# Small Molecule Catalyst for Peptide Synthesis

**DOI:** 10.1021/jacs.5c07242

**Published:** 2025-07-14

**Authors:** Nihar R. Panigrahi, Shahrukh M. Khan, Handoko, Paramjit S. Arora

**Affiliations:** Department of Chemistry, 5894 New York University, 100 Washington Square East, New York, New York 10003, United States

## Abstract

Peptide synthesis is a highly optimized process that has led to the production of new classes of therapeutics and materials. The process of peptide synthesis is straightforward: commercially available, orthogonally protected amino acids can be linked on the solid phase using highly efficient coupling agents. However, the simplicity of peptide synthesis masks a significant drawback of the current method: it is highly wasteful and utilizes a solvent that is facing restrictions on its use. A catalyst that allows solid phase synthesis of peptides in benign solvents without requirement for excess reagents and protected amino acids would have a significant impact. Here, we describe the development of a small molecule catalyst for peptide synthesis. The catalyst design incorporates redox recycling of diselenide and phosphine with air as the ultimate oxidant and phenylsilane as the ultimate reductant. The catalyst affords efficient coupling of amino acids in the solution and solid phase. Significantly, the catalyst functions with acetonitrile, bypassing the need for DMF. The current effort builds on mechanistic analysis of reaction rates and intermediates in our prior work which led to a hydrogen bonding catalyst: [ Handoko; PanigrahiN. R., ; AroraP. S., J. Am. Chem. Soc. 2022, 144, 3637–3643 35188383 10.1021/jacs.1c12798]. Here, we significantly simplified earlier designs to afford an easily accessible small molecule catalyst.

## Graphical abstract

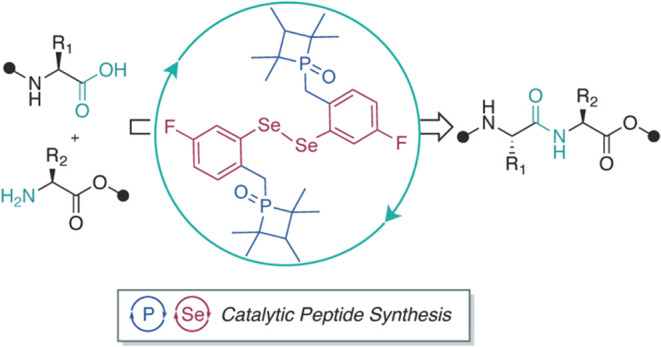

## Introduction

Peptides are gaining momentum as therapeutics with over 100 candidates currently in clinical trials and dozens approved as drugs.^1^ Chemical synthesis remains a mainstay method for the production of these therapeutics. Merrifield’s seminal efforts to develop solid phase peptide synthesis (SPPS),^2^ along with the advent of orthogonal protecting groups^3^ and highly efficient coupling reagents^4^ have allowed automation of peptide synthesis. The success of SPPS relies on the use of excess reagents to drive reactions to completion. However, this inherent advantage of SPPS leads to a resource intensive and wasteful process that consumes excess reagents and solvents. Conventional SPPS methods typically require 3–5 equiv of both protected amino acids and coupling agents for each amide bond formed.^2,5,6^ The requirement for excess reagents is even higher in the emerging flow peptide chemistry technology.^7^ Additionally, the preferred solvent for amino acid coupling in peptide synthesis, *N*,*N*-dimethylformamide (DMF), possesses reprotoxic properties, leading to restrictions to its use.^8,9^ While SPPS remains a vital method, addressing its nonsustainability has become a critical challenge to sustain the success of peptides as therapeutics.^10^

Numerous strategies to achieve catalytic amide bond formation from carboxylic acids and amines have been discussed.^11–18^ Lewis acid catalysts, particularly boron-based reagents, reported by Yamamoto in 1996, have garnered significant attention.^19^ Borate esters^20,21^ and multiboron^22–26^ catalysts enable condensation of a range of amines and carboxylic acids. Progress has also been made in the development of transition metal catalysts, with ZrCl_4_ ^27^ and Ti­(Oi-Pr)_4_ ^28^ emerging as promising options. However, the use of these reagents in SPPS has remained limited because these catalysts require prolonged reaction times, and organometallic catalysts may chelate with oligoamide backbones. An ideal catalyst for peptide synthesis would possess the following attributes: (i) short coupling times, preferably 1–2 h, (ii) compatibility with less toxic solvents, and (iii) compatibility with solid phase conditions.

We recently reported a biomimetic organocatalyst for peptide synthesis.^29–31^ Our approach, inspired by the work of Mukaiyama and others, centers on a redox condensation procedure to activate carboxylic acids as selenoesters in the presence of the diselenide organocatalyst and exogenous phosphine.^32–34^ The catalytic system required four components: the urea–diselenide catalyst **1a**, substoichiometric amount of strained phosphorus reagent **2-[O]**,^35^ silane for recycling of phosphorus­(V) to phosphorus­(III), and oxygen to reoxidize selenol to diselenide.^29^ The system was designed to have an amino acid reversibly attach as a selenoester to the organocatalyst, which features a urea scaffold designed to stabilize the tetrahedral intermediate between amino acids and facilitate amide bond formation (Figure 1A).^31,36–38^ The catalyst enabled coupling of Fmoc-amino acids, without detectable epimerization, and showed promise for oligopeptide synthesis on a solid support. The dual catalytic system leverages silanes as the terminal reductant and oxygen as the terminal oxidant (Figure 1A). Significantly, the reaction proceeded in acetonitrile, allowing us to bypass DMF as a solvent.

**Figure 1:**
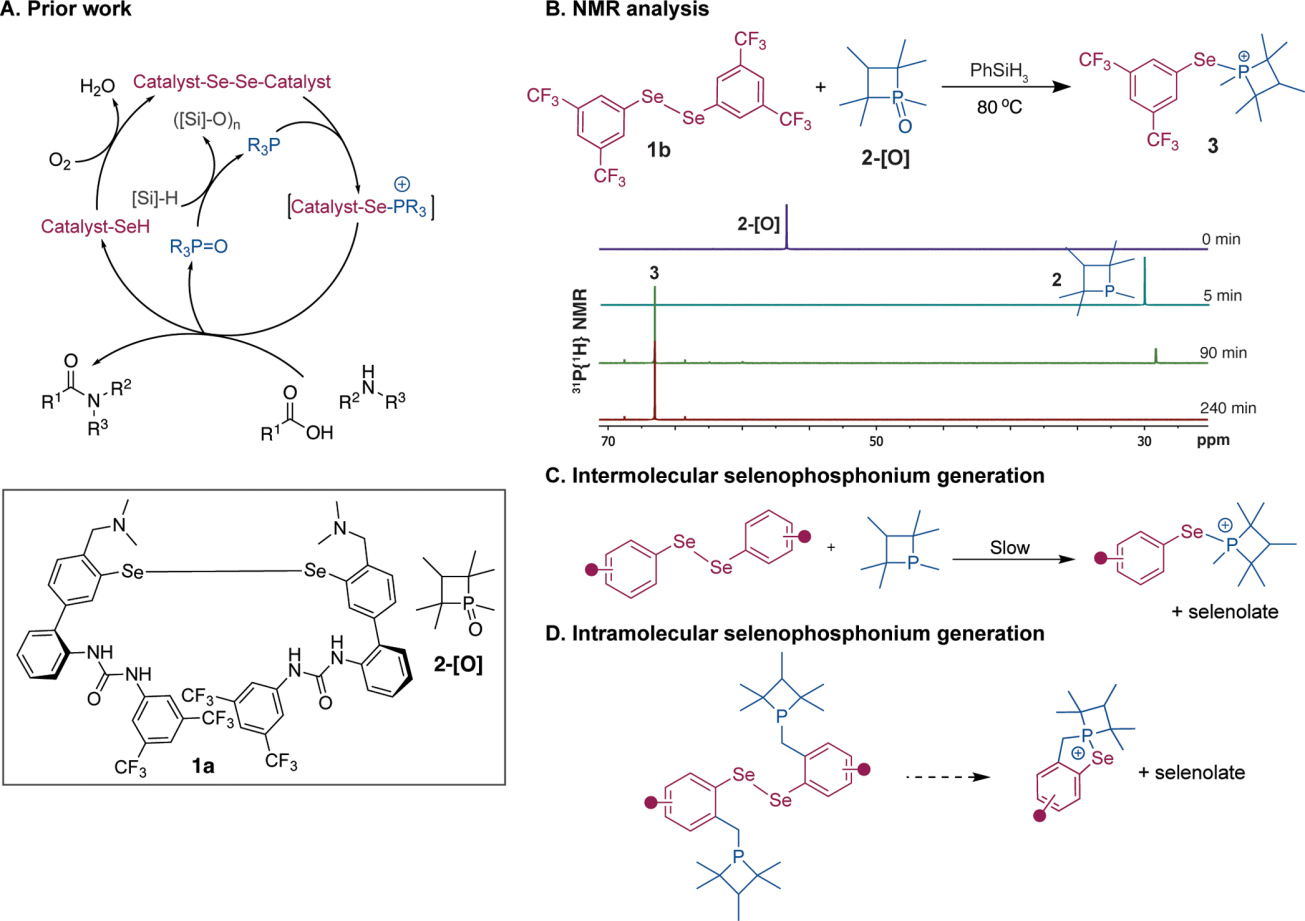
(A) Our previous work on sustainable peptide synthesis showed that a combination of urea diselenide catalyst **1a** and phosphetane oxide **2-[O]** can lead to catalytic formation of the amide bond. This dual catalytic system utilizes phenylsilane as the terminal reductant and molecular oxygen as the terminal oxidant to drive the catalytic cycle. (B) ^31^P NMR analysis of key intermediates reveals that the intermolecular reaction between phosphetane **2** and diselenide **1b** to generate the key selenophosphonium intermediate **3** is sluggish, requiring approximately 4 h for completion. (C) In the prior work, the selenophosphonium intermediate was generated *via* an *intermolecular* reaction between phosphetane **2** and a diaryldiselenide. (D) In the current effort, we developed a small molecule organocatalyst for peptide synthesis wherein the selenophosphonium intermediate is generated through an *intramolecular* reaction.

Here, we build on this previous effort to introduce a simpler and more efficient catalyst for amide bond formation. The new design results from a rigorous analysis of the reaction mechanism and the intermediates. The redesigned catalyst can be synthesized on a multigram scale and enables rapid peptide synthesis, in solution or solid phase, from conventionally protected amino acids with acetonitrile as the solvent.^39^

## Results

### Preliminary Studies Support the *Intramolecular* Catalyst Design

The goal of this project is to reduce the requirement for superstoichiometric amounts of protected amino acids and coupling agents in solid phase peptide synthesis. Our strategy to create an efficient catalyst for amide bond formation builds on a four-component redox recycling mechanism (Figure 1A).^29–31^ We rigorously investigated the mechanism of the catalytic cycle by ^1^H, ^19^F, ^31^P, and ^77^Se nuclear magnetic resonance (NMR) to identify the critical intermediates and enhance its overall efficiency.^29^ The mechanistic analysis with a model diselenide **1b** revealed that the *intermolecular* reaction of sterically hindered phosphetane **2** with **1b** to afford selenophosphonium **3** is slow (Figure 1B). The selenophosphonium adduct constitutes a key intermediate in the catalytic cycle, whose condensation with carboxylic acid affords a selenoester. Based on this insight, we designed a small molecule catalyst in which the selenophosphonium intermediate is obtained from an *intramolecular* reaction (Figure 1C,D).

The reenvisioned catalytic cycle to access the selenophosphonium intermediate *via* an intramolecular reaction is shown in Figure 2A. Reduction of phosphetane oxide-linked diselenide (**I**) with silane is expected to provide intermediate **II**, which is predicted to undergo an intramolecular reaction to yield the desired selenophosphonium adduct **III**. Reaction of carboxylic acid with **III** would yield selenoester **V**, after an acyl transfer step involving intermediate **IV**. Condensation of the selenoester with an amine provides an amide bond and liberates selenol, which is oxidized under aerobic conditions to yield diselenide **I** for the next cycle. The small molecule catalyst design draws inspiration from recent efforts to promote redox organocatalytic reactions.^17,40–42^

**Figure 2:**
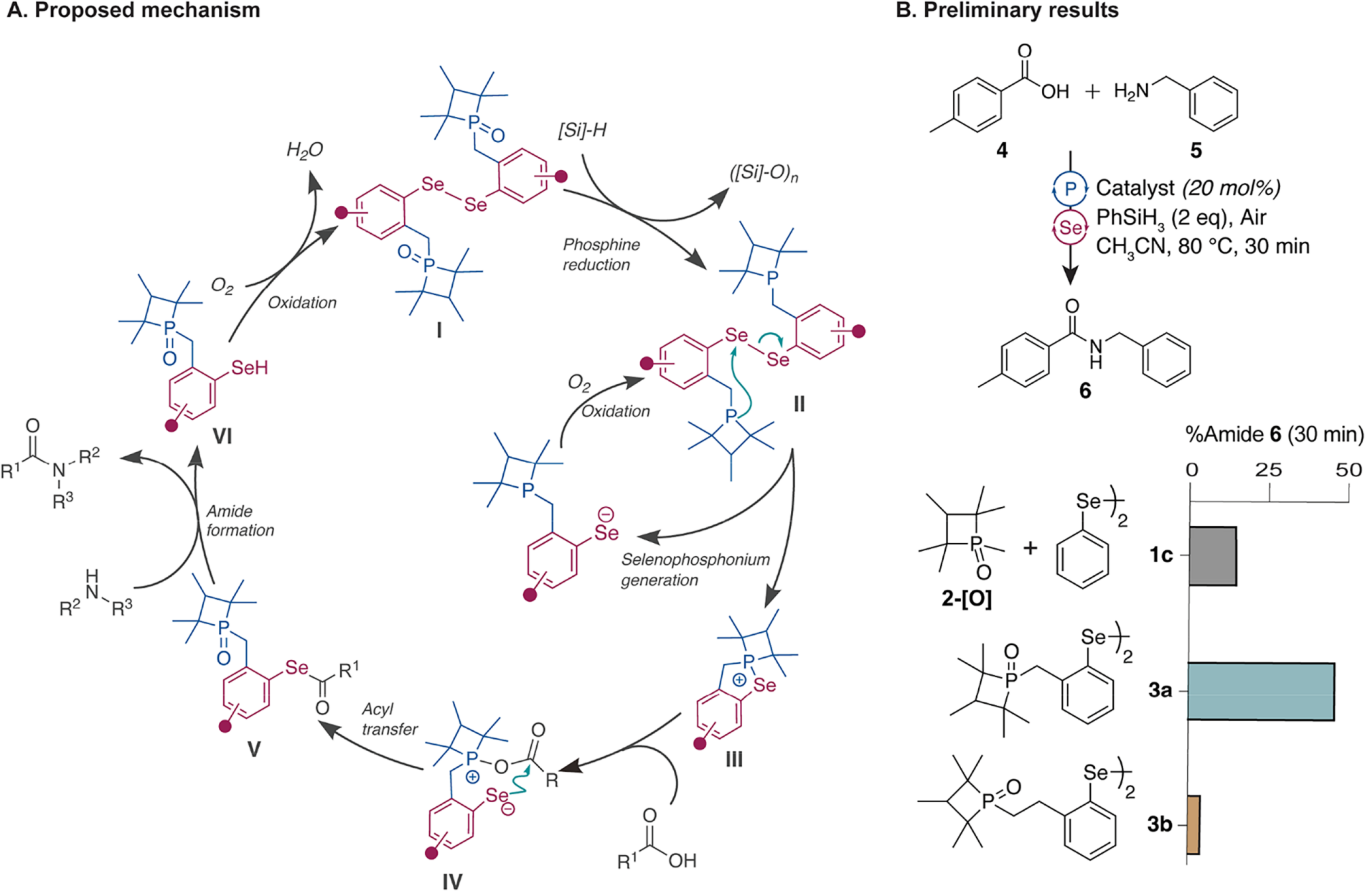
(A) Proposed catalytic cycle for the redesigned organocatalyst for amide bond synthesis. The proposed cycle begins with the reduction of phosphetane oxide to generate **II** which is predicted to undergo an intramolecular reaction to generate selenophosphonium intermediate **III**. The subsequent reaction of carboxylic acid with **III** would lead to the formation of selenoester **V** *via* an acyl transfer step involving intermediate **IV**. Condensation of the selenoester with an amine facilitates amide bond formation, releasing selenol **VI** which is then oxidized under aerobic conditions to regenerate catalyst **I**, completing the catalytic cycle. (B) Preliminary comparison of the catalytic efficiency of the intermolecular catalytic system (**1c** + **2-[O]**) and intramolecular catalytic system **3a** and **3b** for catalytic amide bond formation.

To determine if the switch to an intramolecular reaction to obtain the selenophosphonium adduct leads to an overall increase in the efficiency of amide bond formation, we compared the rates of formation of a model amide product (**6**) from toluic acid (**4**) and benzylamine (**5**). Treatment of the carboxylic acid and amine with 20 mol % diphenyl-diselenide (**1c**) and 20 mol % phosphetane oxide (**2-[O]**), along with phenylsilane (2 equiv) in acetronitrile at 80 °C affords the amide product **6** in 15% yield after 30 min. [Two equiv of phenylsilane are required in the catalytic cycle because one equivalent is needed for absorption of water produced during the reaction.] In contrast, the benzylphosphetane oxide diphenyl-diselenide **3a**, which would enable intramolecular formation of a 5-membered selenophosphonium intermediate, exhibited a substantial increase in the yield of the amide product under identical conditions within 30 min. We next compared the impact of the ring sizes on the reaction progress. Catalyst **3b** features an ethylphosphetane oxide-tethered to diphenyl-diselenide and is designed to generate a 6-membered selenophosphonium intermediate. As expected, the 5-membered ring intermediate leads to the amide product in higher yields as compared to the 6-membered ring intermediate, highlighting the kinetics of the 5-membered ring formation.

The above results support our hypothesis that intramolecular access to the selenophosphonium intermediate would result in a more efficient catalyst. We next focused on optimizing the electronics of the aryl ring to optimize the catalyst. In our proposed catalytic cycle (Figure 2), selenium acts as an electrophile in the formation of selenophosphonium intermediate **III** from **II** but as a nucleophile in the formation of selenoester (**IV → V**). A selenide is also a leaving group in step **II** to **III**. Our foray into catalyst optimization began with the synthesis and screening of electronically diverse analogs of **3a** (Figure 3).

**Figure 3:**
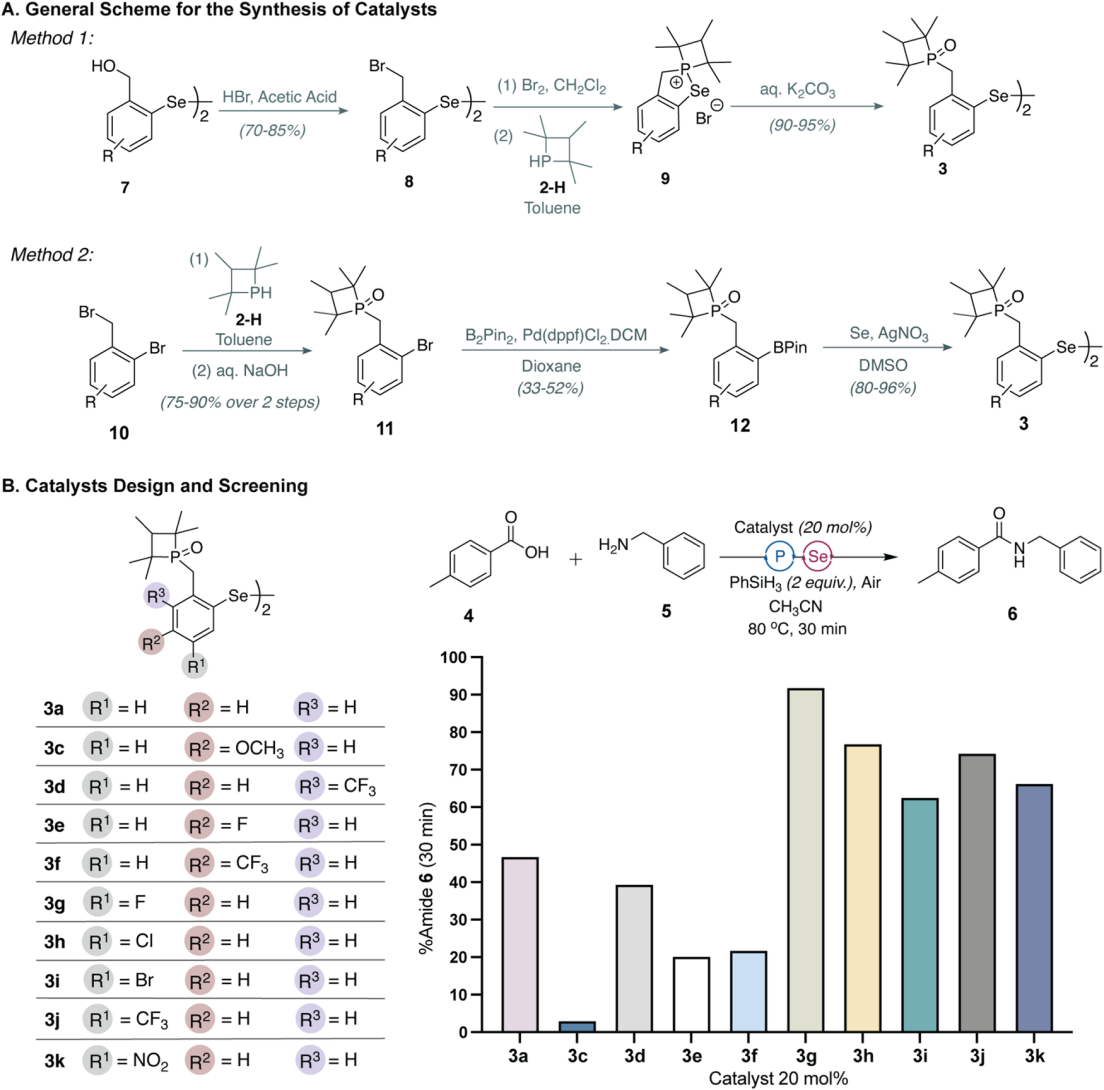
(A) Overview of two distinct synthetic routes developed for phosphetane oxide-tethered aryldiselenide catalysts. (B) Structures of catalysts **3a**–**3k**. These analogs were compared for their efficiency for catalytic amidation for a model reaction between toluic acid **4** and benzylamine **5** to yield **6**. Reaction conditions: **4** (50 μmol), **5** (65 μmol), **3a**–**3k** (20 mol %, 10 μmol), PhSiH_3_ (2 equiv, 100 μmol) in CH_3_CN (1 mL) at 80 °C under air. The %product formation was assessed by HPLC using an internal control (Supporting Information).

### Synthesis of Phosphetane Oxide-Tethered Diselenides

We developed two strategies for the synthesis of phosphetane oxide-tethered diaryl diselenides (Figure 3A). In *Method 1*, bromination of bis­(benzyl alcohol) diselenide **7** afforded bis­(bromobenzyl) diselenide **8** in good yields. Subsequent treatment of **8** with bromine, followed by a double displacement reaction with phosphetane **2-H**, led to the formation of selenophosphonium salt **9**. Finally, aerobic hydrolysis of **9** with aqueous potassium carbonate furnished the bis­(benzylphosphetane oxide) diselenides **3** in near quantitative yields. As an alternative, we explored modification of substituted bromobenzyl bromides to obtain the desired bis­(benzylphosphetane oxide) diselenides. In *Method 2*, phosphetane **2-H** is reacted with benzyl bromide **10** to first afford a phosphonium salt, which is oxidized to phosphetane oxide **11** upon treatment with aqueous sodium hydroxide and exposure to air. We found that **11** can be efficiently converted to aryl diselenides *via* boronate **12**. Borylation of arylhalide with B_2_(Pin)_2_ followed by the treatment of the boronate with elemental selenium and silver nitrate furnishes bis­(benzylphosphetane oxide) diselenides **3** in good-to-excellent yields.

### Evaluation of Phosphetane Oxide-Tethered Diselenides as Catalysts for Amide Bond Formation

Using these methods, we synthesized several analogs of **3a** as illustrated in Figure 3B. These analogs contain electron donating or withdrawing groups at the *meta* (*R* ^1^ and *R* ^3^ substituents on the ring) or *para* (*R* ^2^ substituent) positions relative to the diselenide. Method 1 is suitable for the synthesis of majority of catalysts due to its cost-effectiveness and higher overall yields. However, for some derivatives (such as **3c**), method 2 afforded higher yields, as attempts to isolate the corresponding selenophosphonium salt **9** in method 1 were unsuccessful. Method 2 is a viable alternative for synthesizing all derivatives, but the step to obtain boronate esters **12** from **11** proved to be low yielding for several analogs. We evaluated the effectiveness of catalysts **3a**–**3k** in facilitating amide bond formation between toluic acid **4** and benzylamine **5**. Reactions were conducted at 20 mol % catalyst loading with phenylsilane (2 equiv) at 80 °C in CH_3_CN. Catalyst **3c** bearing an electron donating OMe group at the *R* ^2^ position (*para* to the diselenide group) was initially tested but proved ineffective for amide bond formation. We postulated that the electron donating group in **3c** likely disfavors the nucleophilic attack by the phosphine onto the diselenide, thereby reducing the rate of formation of the selenophosphonium intermediate **III**. Interestingly, the introduction of electron withdrawing groups at the *R* ^3^ (**3d**) or *R* ^2^ position (**3e**–**3f**) also did not enhance the catalytic efficiency compared to the parent **3a**. In contrast, we found that substitution of electron withdrawing substituents at the *R* ^1^ position (**3g**–**3j**) provided a significant improvement in the catalyst performance. Notably, 20 mol % of **3g** afforded near quantitative yields of product **6** within 30 min of reaction time (Figure 3B).

Based on the survey of derivatives in Figure 3B, catalyst **3g** was selected for further exploration. We next assessed reaction parameters including solvents, catalyst loading, additives, and temperature to further optimize reaction yields (Supporting Information, Table S1). Counterions have been shown to impact the stability and reactivity of the selenophosphonium intermediate;^43^ as part of these optimization studies, we evaluated the impact of counterions on the reaction progress. The initial reaction conditions (20 mol % catalyst, acetonitrile as solvent, and high temperature of 80 °C) were chosen based on an extensive optimization with the intermolecular system discussed in Figure 1. For example, the high reaction temperature is required for efficient reduction of the phosphetane oxide.^35^ We found that the initial conditions remain optimal for intramolecular catalyst **3g**.

### Mechanistic Analysis of the Catalytic Cycle by NMR and DFT

To gain insight into the reaction mechanism, we performed single turnover studies with NMR spectroscopy to identify the intermediates. Fluorobenzoic acid **13** was chosen to allow monitoring of the reaction progress by ^19^F NMR. Single turnover studies were designed to assess the involvement of both phosphorus and selenium components in the catalytic cycle for amide bond formation (Figure 4). For these studies, we designed two derivatives of the catalyst, where the diselenide is substituted with selenobromide (**3l**) or selenoether (**3m**). The selenobromide derivative would be expected to yield the same cyclic selenophosphonium intermediate as **3g**, whereas **3m** would not be expected to react. We tracked the formation of amide **15** by reacting equimolar amounts of *p*-fluorobenzoic acid **13**, benzylamine **5**, and the selenium adduct **3m** or **3l**, along with 2 equiv PhSiH_3_ (Supporting Information *)*. Under these single turnover conditions, amide **15** is formed rapidly in the presence of **3l** (Figure 4A). As expected, selenoether **3m** did not lead to product formation.

**Figure 4:**
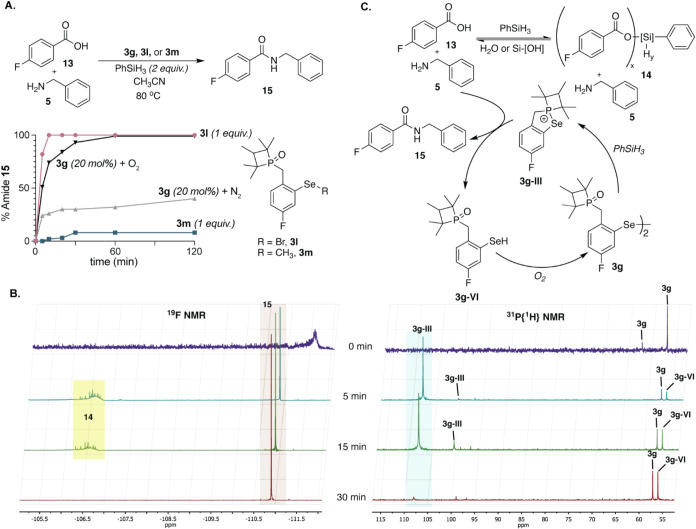
We used a suite of NMR active isotopes (^1^H, ^19^F, ^31^P, and ^77^Se) to assess the proposed mechanism. Carboxylic acid (*p-*fluorobenzoic acid) was chosen to follow product formation by ^19^F NMR for these studies. (A) Analogs **3l** and **3m** were used to confirm that a leaving group on selenium is required for the initiation of the catalytic cycle. Comparison of the performance of **3g** under O_2_ versus N_2_ environments supports the hypothesis that air reoxidation of selenol is needed for the reaction cycle. (B) We simultaneously monitored the ^19^F (left) and ^31^P (right) NMRs to identify the selenophosphonium intermediates **3g-III** and **3g-VI**. (C) Intermediates **14**, **3g-III**, **3g-VI**, and product **15** were identified by NMR.

A combination of ^1^H, ^19^F, ^31^P, and ^77^Se NMRs allowed us to map the presence of other important mechanistic intermediates in catalytic reaction conditions with **3g** (Supporting Information). We were particularly interested in identifying the critical cyclic selenophosphonium adduct **3g-III** (Figure 4C, and the proposed catalytic cycle Figure 2A). Prior to PhSiH_3_ addition, the ^19^F NMR spectrum exhibited a broad signal at 111.65 ppm indicative of salt formation between carboxylic acid **13** and amine **5** (Figure 4B). The corresponding ^31^P NMR spectrum (Figure 4B) displayed two signals at 56.90 (major) and 62.30 (minor), corresponding to diastereomeric catalyst **3g**.

Upon introduction of PhSiH_3_, gas evolution (H_2_) was observed and within 5 min, the ^19^F NMR revealed complex signals between −106 ppm and –107 ppm (Figure 4B left), consistent with the formation of silyl ester intermediates **14** along with a signal at −110.91 ppm corresponding to amide product **15**. The ^31^P NMR spectrum, obtained in tandem, showed two new signals at 107.81 (major) and 100.28 ppm (minor), indicative of the formation of selenophosphonium intermediate **3g-III** (Figure 4B right). After the reaction is completed, the amounts of **3g-III** diminish relative to **3g** (Figure 4B, 30 min plot). Over the course of 30 min, the condensation of **13** and **5** to give **14** was completed as evidenced by the disappearance of the silyl ester signals in the ^19^F NMR spectrum. The ^31^P spectrum displayed a gradual decrease in the intensity of the **3g-III** signals, along with the reappearance of the **3g** signal, confirming the catalyst regeneration. We tentatively assigned **3g-VI** to the peak at 55.96 ppm, but this peak could represent various forms of phosphine oxide intermediates that may form in the catalytic cycle.

In these studies, we also confirmed the involvement of oxygen in the catalytic cycle for amide synthesis. When the model reaction with **3g** (20 mol %) was conducted under a nitrogen environment, product formation was terminated after one cycle (Figure 4A).

We pursued density functional theory (DFT) calculations to understand how the electronics of the aryl group may impact the catalytic cycle and contribute to the enhanced reactivity of **3g** relative to those of other catalysts (Figure 3B). DFT calculations were carried out using the ORCA 6 software package^44^ and visualized with CYLview.^45^ An initial conformer search was performed at the GFN2-*x*TB level^46^ to identify global minimum structures. All geometries were optimized at the r^2^SCAN-3c-SMD­(MeCN) level,^47–49^ and single-point energies were subsequently evaluated at the DLPNO–CCSD­(T)/def2-TZVPP level.^50^ Frequency and thermochemistry calculations were performed at 80 °C to align with the experimental conditions. Additional methodological details and benchmarking are provided in the Supporting Information.

We generated model systems for two key steps in the catalytic cycle, involving selenophosphonium generation and selenoester formation (Figure 5). Our computational results aligned with experimental trends, specifically indicating that the *meta*-fluorine substituted catalyst (**3g**) displays the lowest energy barriers across both steps compared to those of the unsubstituted **3a** and *para*-substituted **3e**. We further analyzed the intermediates using natural bond order (NBO) analysis.^51^ This analysis suggests that the inductive effect of the fluorine substituent is greatest when it is meta to the selenium center, thereby lowering the energy barriers for each step (Supporting Information).^52^ The natural charge on selenium is most negative for **3g** in species **IV**, which facilitates the nucleophilic attack leading to selenoester formation (Figure 5B and Supporting Information). The NBO analysis showed consistent trends across a variety of functionals and basis sets (Supporting Information).^53,54^

**Figure 5:**
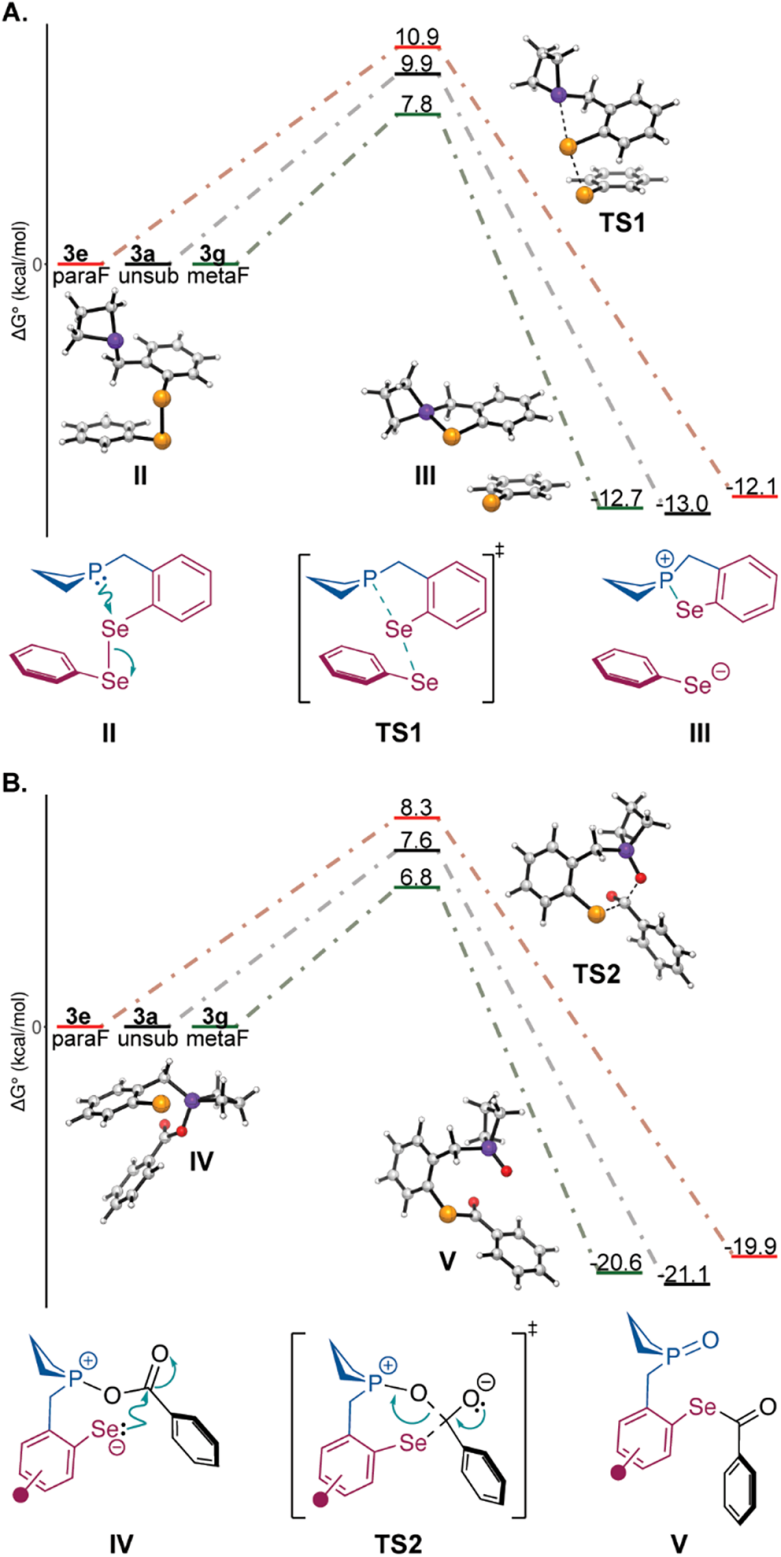
Computational analysis of the impact of ring substituents on the catalyst performance. (A, B) Transition states leading to selenophosphonium adduct **III** and selenoester **V** were calculated. Free energies were calculated at the DLPNO–CCSD­(T)/def2-TZVPP//r^2^SCAN-3c-SMD­(MeCN) level.

### Application of Phosphetane Oxide-Tethered Diselenide **3g** to Dipeptide Synthesis

We next evaluated the efficiency of catalyst **3g** in promoting the coupling of amino acids. Our investigation focused on a diverse set of commercially available Boc-protected amino acids and amino acid esters with results summarized in Scheme 1. Condensation of Boc-Ala-OH with benzylamine and alanine *tert-*butyl ester in acetonitrile provided >90% yields of dipeptides **16a** and **16b** in <1.5 h. Encouraged by these results, we expanded our investigation to include phenylalanine and tryptophan, valine, proline, lysine, aminoisobutyric acid (Boc-Aib), histidine, aspartic acid, and arginine amino acids (**16c**–**16s**). In all cases, we were pleased to observe high yields of dipeptide products within 1–2 h, particularly for the β-branched valine, secondary amine proline, and sterically hindered Boc-Aib. No amide product **16u** was obtained with aniline due to the low nucleophilicity of the aromatic amine. We have previously demonstrated that little to no epimerization is observed with carbamate-protected amino acids activated as arylselenoesters.^29,30^

**Scheme 1:**
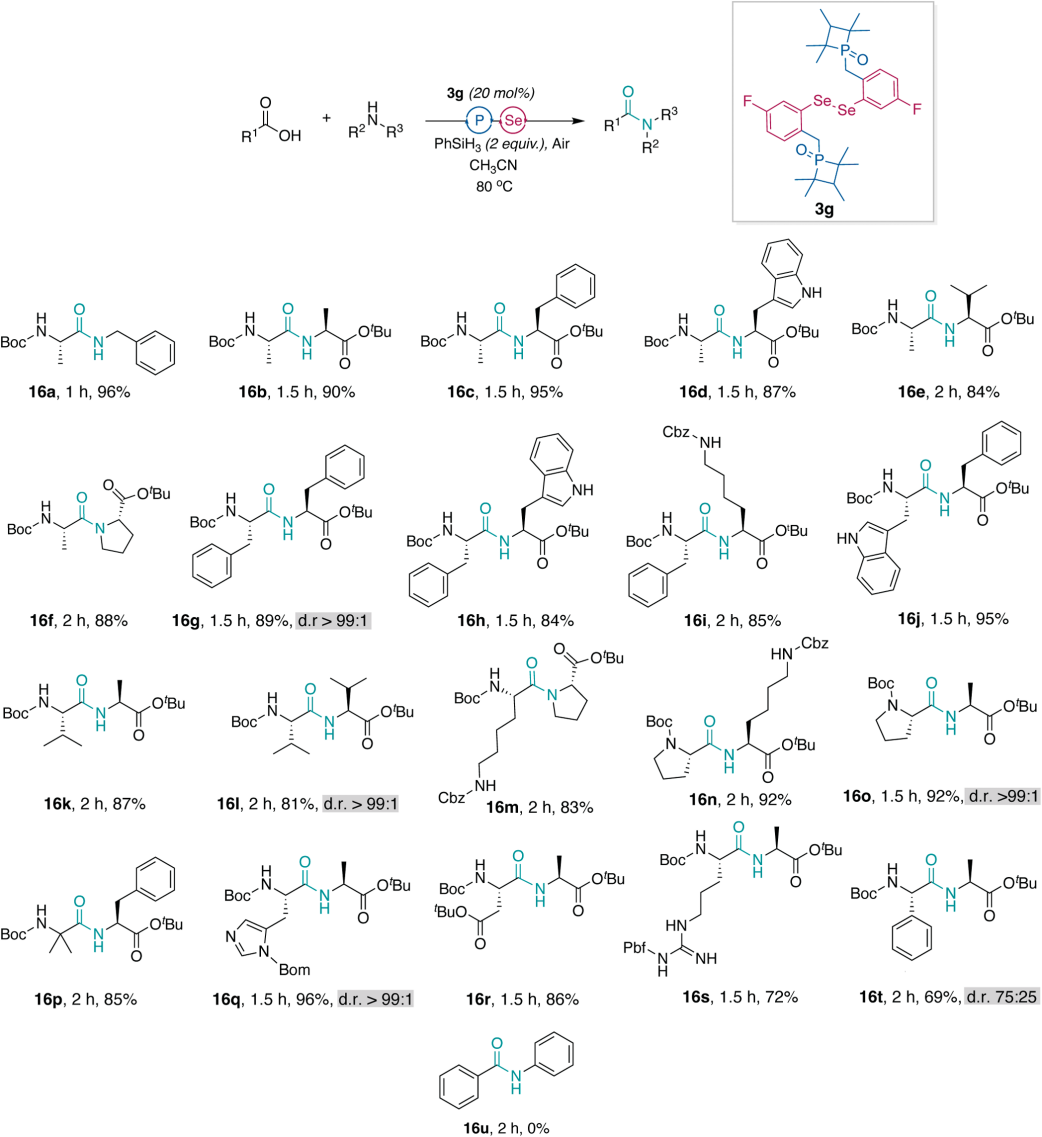
Catalytic Synthesis of Dipeptides^a^

a Reaction condition: R^1^COOH (0.5 mmol), R^2^R^3^NH (0.55 mmol), **3g** (20 mol %), and PhSiH_3_ (2 × 1 equiv every 30 min) in CH_3_CN (5 mL) at 80 °C under air. Progress of the reaction was analyzed by thin layer chromatography and isolated yields are reported. We analyzed the potential epimerization of amino acids in the catalyst-mediated coupling by NMR or HPLC; the diastereomeric ratio (d.r.) is highlighted in gray. Detailed reaction procedures and epimerization analysis are included in Supporting Information.

To determine if diselenide **3g** catalyzed reactions lead to epimerization-free coupling of amino acids, we analyzed crude NMRs or HPLC spectra of dipeptides Boc-l-Val-l-Val-O^*t*^Bu (**16l)**, Boc-l-Phe-l-Phe-O^*t*^Bu (**16g)**, Boc-l-Pro-l-Ala-O^*t*^Bu (**16o**), Boc-l-His-l-Ala-O^*t*^Bu (**16q**), and Boc-l-Phg-l-Ala-O^*t*^Bu (**16t**). Crude spectra used to determine the diastereomeric ratios are included in the Supporting Information. Spectra of the L,L dipeptides were compared to those of the D,L diastereomer as standards. We chose histidine, valine, and phenylglycine because they are known to be sensitive to epimerization as carboxylic acids in peptide synthesis.^55–58^ The catalyzed synthesis of dipeptides resulted in undetectable levels of epimerization for all dipeptides, with the exception of phenylglycine (Scheme 1). The results are in agreement with our previous analyses and show that negligible epimerization of carbamate-protected natural amino acids results in the selenoester-mediated amide bond formation.

### Solid Phase Peptide Synthesis with Catalyst **3g**

The overarching goal of this catalyst design is to improve the sustainability of solid phase peptide synthesis by replacing stoichiometric coupling reagents with a catalytic system while employing an environmentally benign solvent. Fmoc solid phase peptide synthesis (SPPS) is the method of choice for peptide synthesis. For any catalyst to have a practical impact, it would need to be operational under the Fmoc SPPS conditions. The advantage of SPPS is that reactions can be driven to completion by the use of excess reagents. This key attribute of SPPS has led to waste in contemporary peptide synthesis. We sought to determine if catalyst **3g** would provide efficient synthesis of oligopeptides on solid phase with a slight excess of Fmoc-amino acids (1.5 equiv), 20 mol % **3g**, and 2.5 equiv of PhSiH_3_ in acetonitrile. We used Tentagel-S-RAM resin due to its superior swelling properties in acetonitrile.

We tested three different sequences with a range of amino acid residues and side chain protecting groups (Figure 6). We began by synthesizing pentamer Fmoc-KCGFG-NH_2_ (**17a**) using standard Fmoc-amino acids in acetonitrile. Peptide elongation was carried out at 80 °C using 20 mol % of **3g**, 1.5 equiv of Fmoc-amino acids, and 2.5 equiv of PhSiH_3_ in acetonitrile for each coupling step. Kaiser test was used to monitor reaction progress. Cycles of coupling and deprotection (see Supporting Information *)* were repeated with different amino acids until the complete peptide was obtained. HPLC and mass spectrometry analyses of the crude peptide confirmed the formation of a high purity product (Figure 6A). We next synthesized 5-mer Ac-LWFGA-NH_2_ (**17b**) and 11-mer Ac-QCFVAYKCFG-NH_2_(**17c**). While **17b** was obtained with satisfactory purity, **17c** required two coupling cycles per residue beyond the sixth amino acid for complete conversion. Nevertheless, HPLC analysis demonstrated high purity for the final product, as compared to the peptide prepared using standard Fmoc solid phase synthesis procedure with 5 equiv each of HBTU and Fmoc-amino acids (Figure 6D).

**Figure 6:**
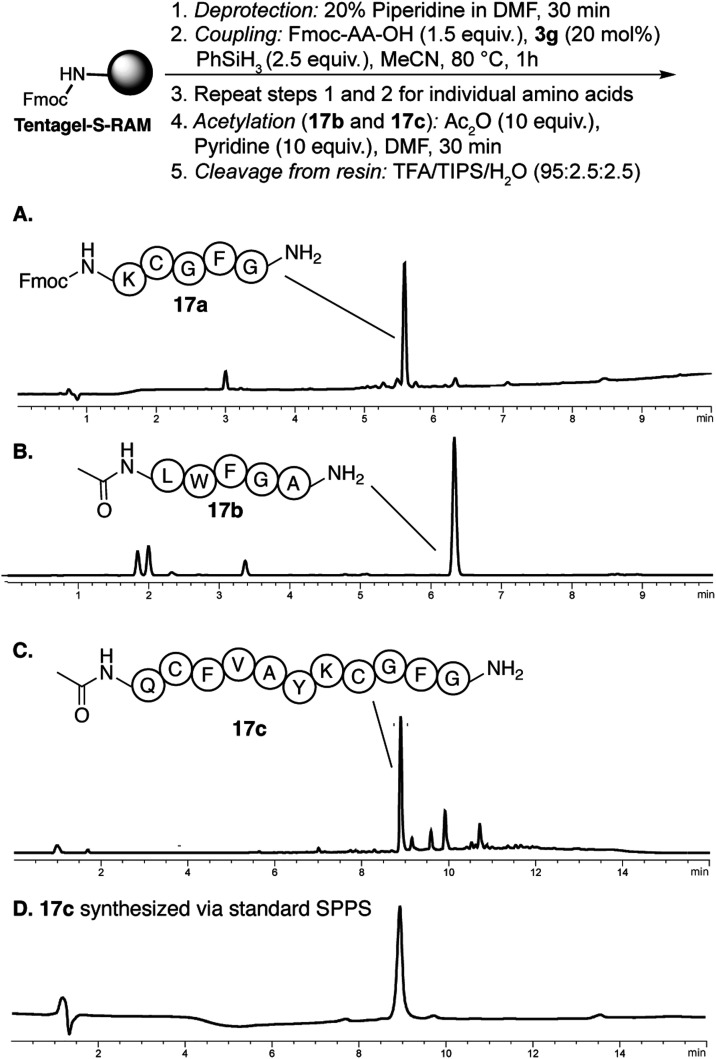
Catalytic solid phase peptide synthesis using **3g**. HPLC chromatograms (λ 220 nm) of crude peptides (A) Fmoc-KCGFG-NH_2_ **(17a**), (B) Ac-LWFGA-NH_2_ (**17b**), (C) Ac-QCFVAYKCGFG-NH_2_ (**17c**) obtained from catalytic SPPS, and (D) **17c** synthesized using standard Fmoc solid phase peptide synthesis (SPPS) using 5 equiv each of HBTU and Fmoc-amino acid per coupling. Detailed reaction conditions and HPLC purification protocols are included in the Supporting Information.

## Conclusions

In this study, we have developed a small molecule organocatalyst for peptide synthesis. The catalyst design incorporates redox recycling of aryldiselenide and phosphetane oxide with air as the ultimate oxidant and phenylsilane as the ultimate reductant. The catalyst can be readily accessed from simple starting materials and provides high yields for a range of dipeptide synthesis and, notably, solid phase synthesis of oligopeptides. Two salient features of the catalyst are that the reaction can be run in air and is compatible with acetonitrile, which is an attractive solvent to DMF both for its lower toxicity and damaging environmental impact.^9^ Despite the current progress, challenges remain, which may limit the practical utility of **3g**. In particular, the sensitivity of catalyst **3g** to water necessitates a need for a higher mol % of the catalyst and phenylsilane. Acetonitrile proved to be a suitable solvent for the solid phase synthesis of short peptides tested herein but its use as a general replacement for DMF remains to be explored.^39^ Our current efforts focus on reducing the amount of catalyst needed for efficient amide bond formation and exploring the effectiveness of the catalyst in automated synthesizers.

## Supplementary Material



## Notes

Synthesis and characterization of compounds, description of DFT analysis (PDF)
