# Supplementary material for: Small Molecule Catalyst for Peptide Synthesis
Source: J Am Chem Soc. 2025 Jul 14;147(29):25682–91. doi: 10.1021/jacs.5c07242 (PMC12291467; doi:10.1021/jacs.5c07242)
Supplement: Supplementary file 1 [file ja5c07242_si_001.pdf]

SUPPORTING INFORMATION

A Small Molecule Catalyst for Peptide Synthesis

Nihar R. Panigrahi, Shahrukh M. Khan, Handoko, Paramjit S. Arora*

Department of Chemistry, New York University, 100 Washington Square East, New York, NY 10003

Table of Contents

1 Methods - General Considerations.......... S1
2 Catalyst Screening and Reaction Tracking.......... S2
3 Reaction Condition Screening.......... S3
4 Mechanistic Studies.......... S4
5 Synthetic Procedures.......... S8
5.1 Synthesis of Catalysts.......... S8
5.2 Solution-Phase Dipeptide Synthesis.......... S45
5.3 Catalytic Solid-Phase Peptide Synthesis.......... S56
6 Analysis of Amino Acid Epimerization.......... S57
7 Computational Methods.......... S61
7.1 General Computational Information.......... S61
7.2 NBO Analysis.......... S61
7.3 Cartesian Coordinates for Optimized Structures.......... S65
8 References.......... S101
9 NMR Spectra of Compounds.......... S102

1 Methods - General Considerations

- All reagents were used as received from commercial suppliers unless otherwise noted.
- All solvents are reagent grade or HPLC grade. Anhydrous acetonitrile (MeCN), dichloromethane (CH_2Cl_2), *N*,*N*-dimethylformamide (DMF), and tetrahydrofuran (THF) were obtained from a dry solvent system (passed through a column of alumina) and stored with activated 3Å molecular sieves for at least 24 hours before use. Dichloroethane (DCE), dioxane, and toluene were stored and dried with activated 3Å molecular sieves (directly from commercially purchased bottle) for at least 48 hours before use.
- ^{1}H NMR, ^{13}C NMR, ^{19}F NMR, ^{31}P and ^{77}Se NMR experiments were performed on Bruker Avance 400, 500 or 600 MHz NMR spectrometer. NMR spectra data are reported as chemical shift (in ppm) (multiplicity, integration, coupling constants (in Hz). Chemical shifts for ^{1}H NMR were calibrated internally against residual peaks of NMR solvents: $CDCl_3$ (7.26), CD_3CN (1.94). CD_3OD (3.31), D_2O (4.80), acetone-*d6* (2.05), DMSO-*d6* (2.50). Chemical shifts for ^{13}C NMR were also calibrated internally against residual peaks of NMR solvents: $CDCl_3$ (77.0), CD_3CN (1.32). CD_3OD (49.0), acetone-*d6* (29.8), DMSO-*d6* (39.5). Chemical shifts for ^{19}F, ^{31}P and ^{77}Se NMR were reported without calibration. Multiplicity is reported as follows: singlet (s), broad (br), doublet (d), doublet of doublet (dd), doublet of triplet (dt), triplet (t), triplet of doublet (td), quartet (q), quintet/pentet (p), and multiplet (m).
- HPLC analyses were performed on Agilent 1200 series or 1260 infinity with appropriate columns and elution conditions.
- Low-resolution mass spectrometry was performed using Agilent 1100 Series Single Quadrupole LC/MS. High-resolution mass spectrometry was performed using Agilent 6224 TOF LC/MS and Waters ACQUITY/Xevo G3 LC-QToF MS.
- Reactions were monitored by thin-layer chromatography (TLC) using TLC silica gel 60 F254 glass plate. Compounds were detected by UV (254 nm) or staining (ninhydrin or potassium permanganate).

2 Catalyst Screening and Reaction Tracking

General procedure

An oven dried 1-dram vial equipped with a magnetic stir-bar was added *p*-toluic acid **4** (6.81 mg, 50 μmol), benzylamine **5** (7.11 μL, 65 μmol), catalyst **3g** (20 mol%, 10 μmol), biphenyl (7.71 mg, 50 μmol, as an internal standard), anhydrous CH_3CN (1.0 mL) and $PhSiH_3$ (12.30 μL, 100 μmol). The vial was placed on a heating block at 80 °C. The progress of the reaction was monitored by analytical HPLC.

Reaction progress monitoring:

From the reaction mixture, 5.0 μL of aliquot was transferred and quenched into a small LCMS vial containing 195 μL of MeCN (5% Water + 0.1% TFA). Ten microliters of the diluted solution was then injected into HPLC column (Poroshell 120 EC-C18 4.6 × 100 mm 2.7 μm column). HPLC condition: 0.1% TFA (v/v) in water (solvent A): acetonitrile (solvent B); gradient 45–100% (solvent B) in 8 min, flow rate = 1.5 mL/min, detection wavelength = 254 nm.

Reaction conversion at any time point was determined through interpolation from a standard curve (**Figure S1**) correlating the ratio of product/biphenyl concentration to that of product/biphenyl peak area:

$$\%Product = \frac{A_p}{A_{is}} \times \frac{C_{is}}{0.4482} \times \frac{1}{C_{sm}} \times 100\% \quad (1)$$

Where A_p is the peak area of the product, A_{is} is the peak area of the internal standard (biphenyl), C_{is} is the concentration of the internal standard, and C_{sm} is the concentration of the starting material (toluic acid **4**).

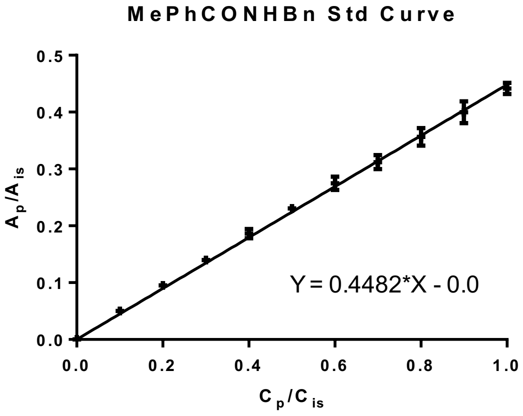

Figure S1. Standard curve for *N*-benzyl-4-methylbenzamide (**6**) showing a linear correlation between the ratio of product/biphenyl concentration (C_p/C_{is}) and product/biphenyl peak area (A_p/A_{is}). All data points were obtained in triplicate.

3 Reaction Condition Screening

Table S1.

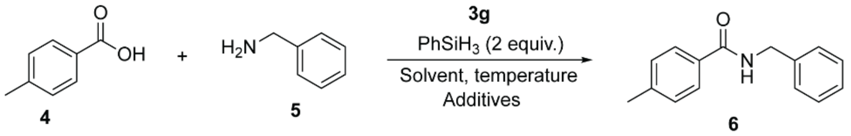

Entry	Solvent	Loading	Additive	Temp, °C	Conversion, %
**1**	CH_3CN	20 (mol%)	—	80	91
**2**	$PhCF_3$	20 (mol%)	—	80	55
**3**	DCE	20 (mol%)	—	80	13
**4**	2-MeTHF	20 (mol%)	—	80	35
**5**	Toluene	20 (mol%)	—	80	51
**6**	DMF	20 (mol%)	—	80	70
**7**	CH_3CN	10 (mol%)	—	80	67
**8**	CH_3CN	5 (mol%)	—	80	58
**9**	CH_3CN	20 (mol%)	—	60	31
**10**	CH_3CN	20 (mol%)	—	70	42
**11**	CH_3CN	20 (mol%)	TBAB (1 equiv.)	80	89
**12**	CH_3CN	20 (mol%)	KPF_6 (1 equiv.)	80	90
**13**	CH_3CN	20 (mol%)	NaBArF (1 equiv.)	80	82
**14**	CH_3CN	10 (mol%)	NaBArF (1 equiv.)	80	37

%Conversions after 30 minutes are reported and were monitored using HPLC using biphenyl as internal standard. TBAB = Tetrabutylammonium bromide, KPF_6 = Potassium hexafluorophosphate and NaBArF = Sodium tetrakis[3,5-bis(trifluoromethyl)phenyl]borate.

4 Mechanistic Studies

A combination of ^{19}F and ^{31}P NMR was used to gain mechanistic insights. As shown in **Figure S2** When **13** and **5** were mixed, the expected ammonium carboxylate salt was generated (^{19}F δ −111.4 ppm). Upon addition of **3g** and $PhSiH_3$ to the mixture, gas evolution was observed and the ^{19}F NMR spectrum of the reaction mixture showed complex signals (^{19}F δ -106 to -107 ppm) due to the formation of silyl ester intermediate **14** along with a signal at ^{19}F δ −110.91 ppm indicating

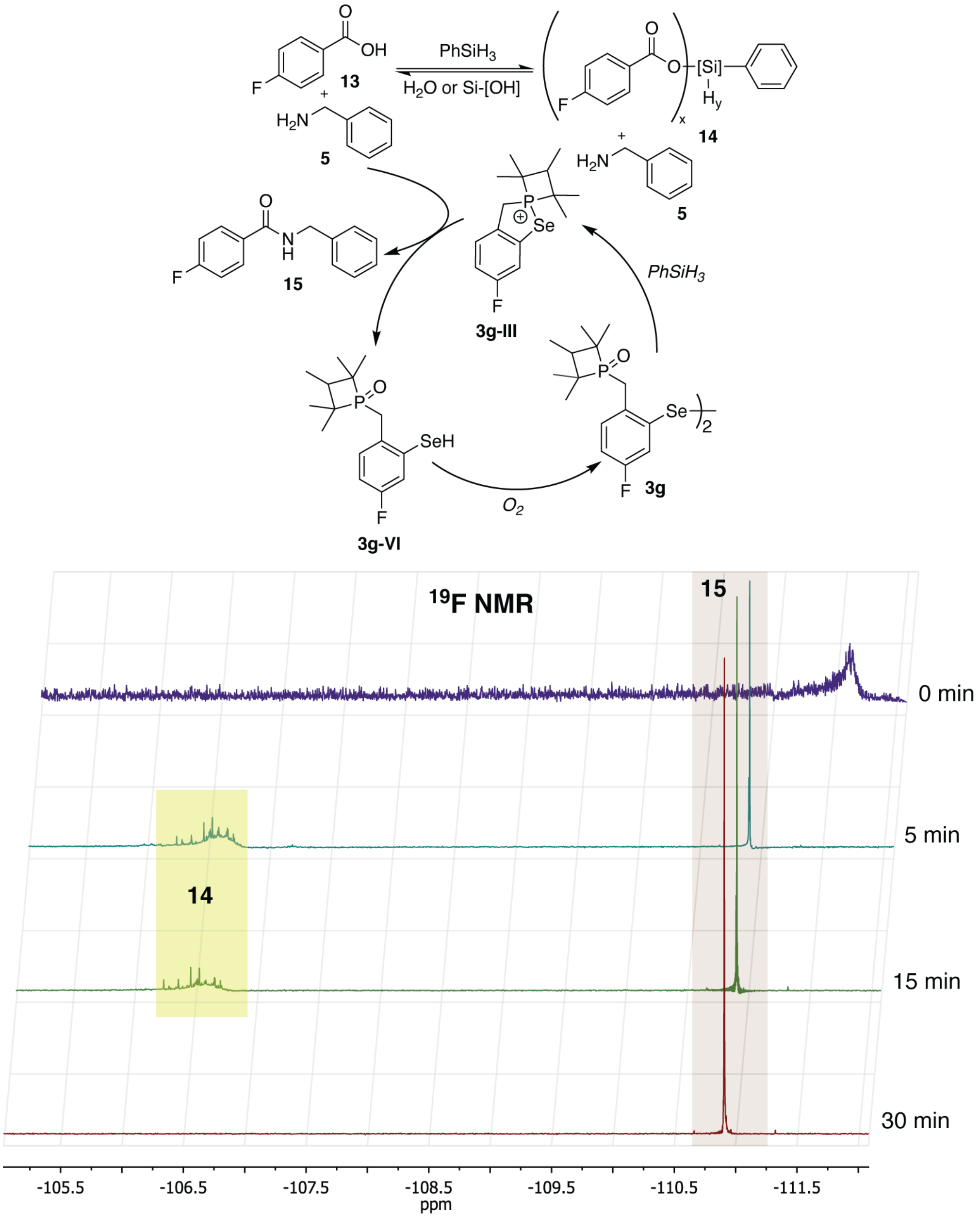

Figure S2. Reaction condition: carboxylic acid **13** (18 µmol), benzylamine **5** (22 µmol), **3g** (20 mol%) and $PhSiH_3$ (40 µmol) in CD_3CN (1 mL) at 80 °C under open air.

amide product **15** formation. Over the course of 30 minutes, the condensation of **13** and **5** to give **15** was completed as evidenced by the disappearance of the silyl ether **14** signal.

We observed two signals with ^{31}P NMR prior to the addition of $PhSiH_3$: at ^{31}P δ 56.90 ppm (major)

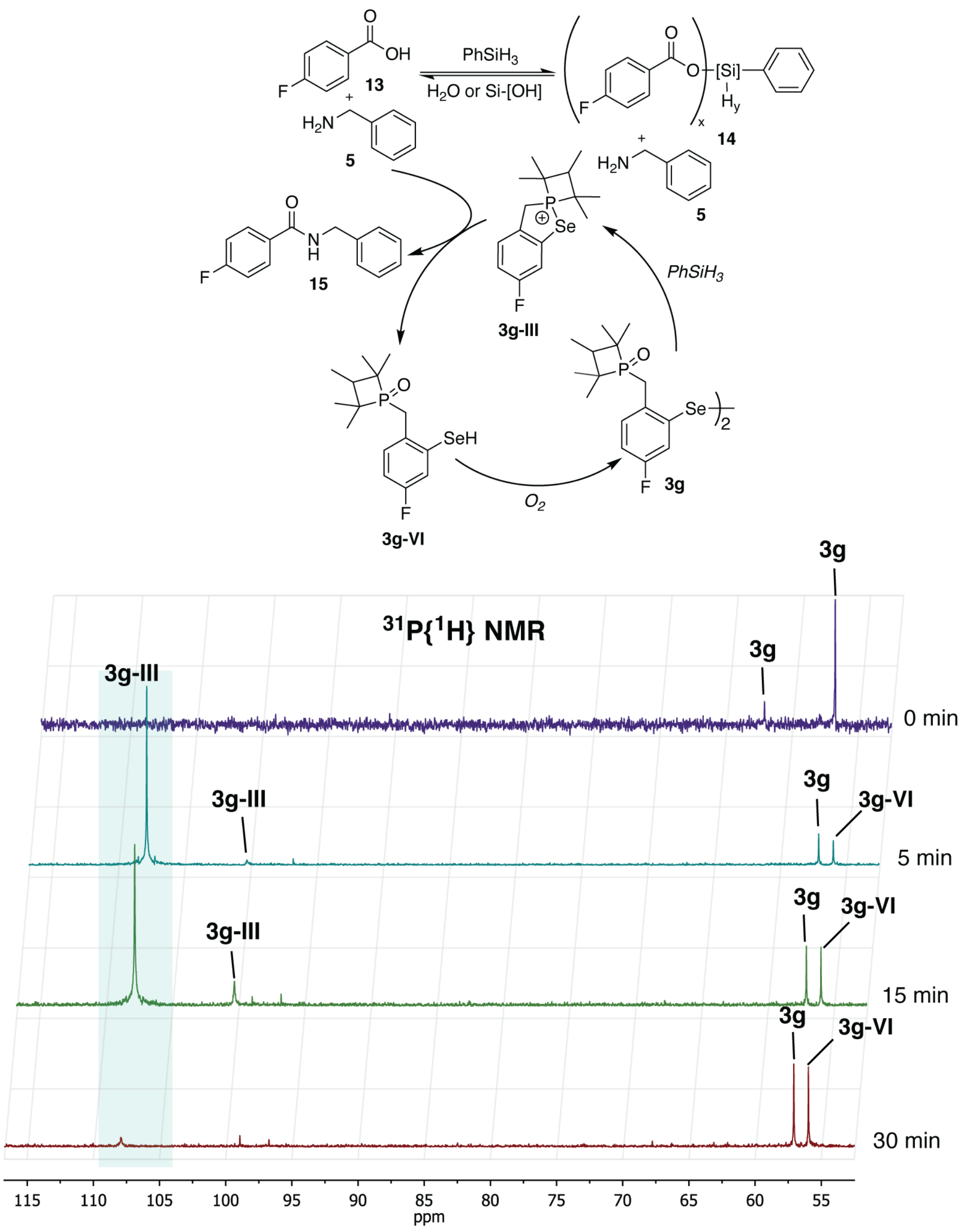

Figure S3. Reaction condition: carboxylic acid **13** (18 µmol), benzylamine **5** (22 µmol), **3g** (20 mol%) and $PhSiH_3$ (40 µmol) in CD_3CN (1 mL) at 80 °C under open air.

and 62.30 ppm (minor) (**Figure S3**). Upon addition of $PhSiH_3$ ^{31}P NMR showed two new signals at ^{31}P δ 107.81 ppm (major) and 100.28 ppm (minor) indicative of selenophosphonium **3g-III**. Over the course of 30 minutes, the ^{31}P spectrum displayed a gradual decrease in the intensity of **3g-III** signals, along with the reappearance of the **3g** signals, confirming catalyst regeneration.

Single Turnover Studies:

To investigate the roles of both phosphine and selenium components in the catalytic cycle for amidation, we conducted single-turnover experiments using compounds **3l** and **3m**. Compound **3l**, a selenolbromide, is anticipated to form the cyclic selenophosphonium intermediate, analogous to the behavior of **3g**. In contrast, compound **3m**, with its selenium protected by a methyl group, is not expected to produce this selenophosphonium intermediate. We opted for **3l** instead of **3g** in the NMR studies to enhance clarity. As depicted in Figure S4, the reaction with 1 equivalent of **3l** resulted in significantly faster formation of product **15**, achieving quantitative conversion within 10 minutes. In contrast, when conducted with **3m**, only a trace amount of **15** was detected, likely attributable to amidation facilitated by phenylsilane and phosphine.

Oxygen Dependent Studies:

In these studies, we also confirmed the involvement of oxygen in the catalytic cycle for amide synthesis. As shown in Figure S4, when the model reaction with **3g** (20 mol%) was conducted under a nitrogen environment, as seen in Figure S4, the formation of product **15** was notably slow. However, when conducted in the presence of air, the same reaction with **3g** (20 mol%) yielded nearly quantitative amounts of **15** within 30 minutes.

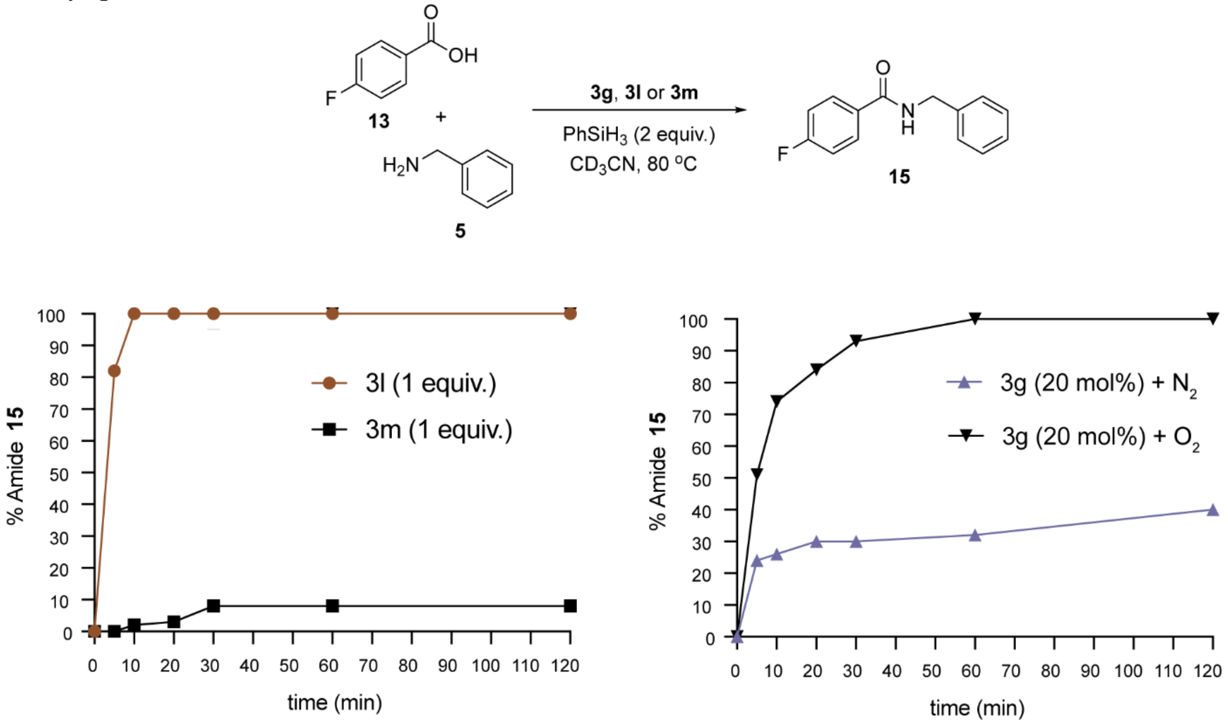

Figure S4. (left) Single turnover studies with **3l** and **3m**. Reaction condition: **13** (18 μmol), **5** (36 μmol), **3l** or **3m** (18 μmol) and $PhSiH_3$ (40 μmol) in CD_3CN (1 mL) at 80 °C (2 equiv. of amine **5** was used to neutralize any HBr formed upon reaction with **3l**. (right) Oxygen dependent studies

with **3g**. Reaction conditions: **13** (18 μmol), **5** (22 μmol), **3g** (20 mol%) and $PhSiH_3$ (40 μmol) in CD_3CN (1 mL) at 80 °C. Reactions were conducted inside 5 mm NMR tubes. Percent conversion was determined by ^{19}F NMR.

<u>Procedure for Single Turnover NMR Studies:</u>

A 5 mm NMR tube consisting of **3l** (7.67 mg, 18 μmol) or **3m** (6.50 mg, 18 μmol) was charged with a solution of carboxylic acid **13** (2.52 mg, 18 μmol) in 1 mL CD_3CN. Benzylamine **5** (3.93 μL, 36 μmol) and $PhSiH_3$ (4.92 μL, 40 μmol) were then added to the NMR tube and the tube was sealed with a cap (2 equiv. of **5** was used to neutralize any HBr formed upon reaction with **3l**). The tube was then placed into NMR spectrometer (temperature set to 80 °C) for data acquisition.

<u>Procedure to Probe Air Dependence</u>:
A 5 mm NMR tube consisting of **3g** (2.49 mg, 3.60 μmol) was charged with a solution of carboxylic acid **13** (2.52 mg, 18 μmol) in 1 mL CD_3CN. The tube was then flushed with nitrogen. Under a positive flow of nitrogen benzylamine **5** (2.40 μL, 22 μmol) and $PhSiH_3$ (4.92 μL, 40 μmol) were added, and the tube was sealed with a cap and then placed into NMR spectrometer (temperature set to 80 °C) for data acquisition.

For reaction exposed to air, a similar procedure was followed except no nitrogen purging was involved and a hole was punched into the NMR tube cap.

5 Synthetic Procedures

5.1 Synthesis of Catalysts

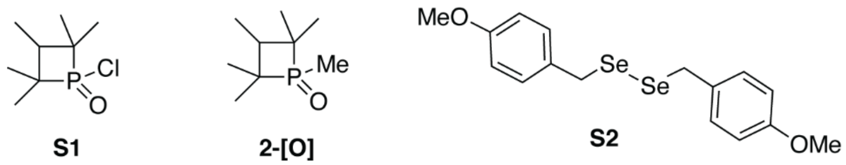

S1 and **2-[O]** used in this report were synthesized following published procedures.[1] Synthesis of **S2** has also been previously described.[2]

General Procedure A (Diaryl diselenide synthesis from aryl boronic acid or aryl boronate ester):

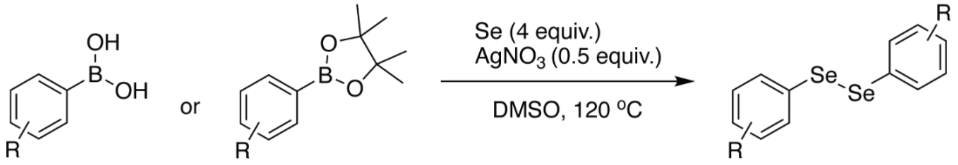

An oven-dried round-bottom flask equipped with a magnetic stir bar was charged with aryl boronic acid or aryl boronate ester (1 equiv.), elemental selenium (4 equiv.), and $AgNO_3$ (0.5 equiv.) in DMSO (0.1 M). The reaction mixture was heated to 120 °C for 12 hours under a steady flow of air. Upon completion, as monitored by TLC, the reaction was quenched with water. The organic compounds were extracted using DCM, and the combined organic layers were dried over Na_2SO_4, filtered, and concentrated under reduced pressure. The crude product was then purified via column chromatography.

General Procedure B (Aryl Se-PMB synthesis from aryl fluoride)

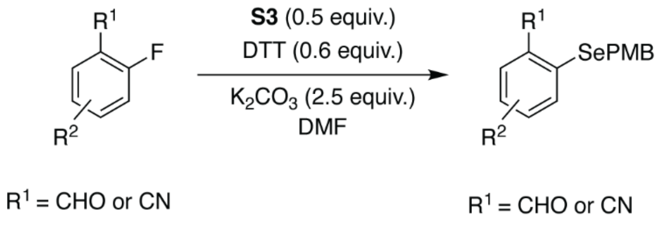

An oven-dried 2-neck flask equipped with a magnetic stir-bar was charged with **S3** (0.5 equiv.) and DMF (0.25 M). The solution in the reaction vessel was purged with N_2 for 30 min. Into it was then added 1,4-dithiothreitol (0.6 equiv.) under a positive flow of nitrogen. The reaction mixture was then stiired for 30 minutes at RT. Aryl fluoride (1 equiv.) was then added under a positive flow of nitrogen and the reaction mixture was stiired at RT for additional 15 minutes. Then K_2CO_3 (2.5 equiv.) was added and stirred at RT. Progress of the reaction was monitored by TLC. After completion, the reaction was quenched by water and the organic compounds were extracted using

diethyl ether. The combined organic layers were washed with brine, dried over Na_2SO_4, filtered and concentrated under reduced pressure. The crude product was then purified via column chromatography.

General Procedure C (Se-PMB deprotection using PIFA)

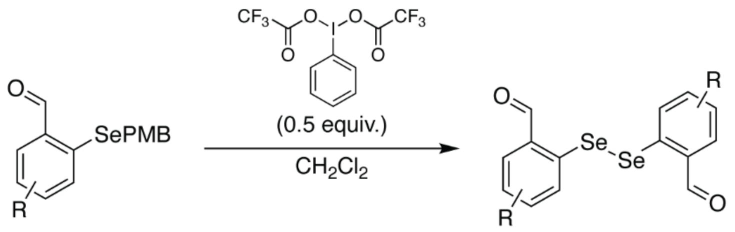

An oven-dried, round-bottom flask equipped with a stir bar was charged with aryl-SePMB (1 equiv.) and CH_2Cl_2 (0.2 M). [Bis(trifluoroacetoxy)iodo]benzene (PIFA) (0.5 equiv.) was added in one portion, and the reaction mixture was stirred at room temperature for 30 minutes. The reaction progress was monitored by TLC. Upon completion, volatiles were removed under reduced pressure, yielding a yellow solid. Hexanes were added to dissolve the by-product, while the desired product remained insoluble. The solid was collected by filtration and further washed with hexanes to afford the pure product

General Procedure D (Dibenzyl alcohol diselenide synthesis from dibenzaldehyde diselenide):

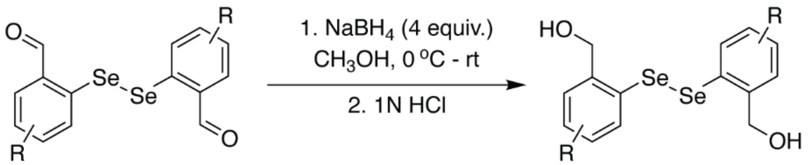

An oven-dried 2-neck flask equipped with a magnetic stir-bar was charged with dibenzaldehyde diselenide (1 equiv.) and CH_3OH (0.2 M). The reaction vessel was cooled to 0 °C and under a positive flow of nitrogen $NaBH_4$ (4 equiv.) was added in small portions (exothermic reaction). After complete addition of $NaBH_4$, the reaction mixture was stirred was 2 hours at RT and then quenched by adding 1N HCl. The organic compounds were extracted using DCM, and the combined layers were washed with brine and dried over Na_2SO_4, filtered, and concentrated under reduced pressure. The resulting product was used for the next step without further purification.

General Procedure E (2-(bromomethyl)benzeneselenenyl bromide derivatives synthesis from

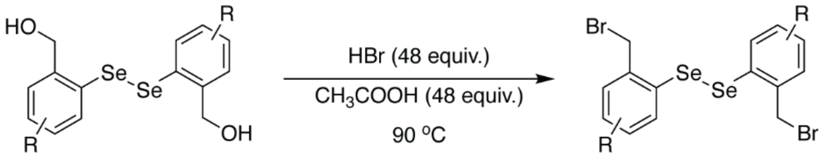

A round-bottom flask equipped with a stir bar and a reflux condenser was charged with dibenzyl alcohol diselenide (1 equiv.), glacial acetic acid (48 equiv.) and 48% HBr (48 equiv.). The reaction vessel was stirred at 90 °C and the progress of the reaction was analyzed by TLC. Upon completion, the reaction mixture was cooled to 0 °C and neutralized by slowly adding saturated solution of K_2CO_3 (exothermic). The organic compounds were extracted using DCM and the combined layers were washed with brine and dried over Na_2SO_4, filtered and concentrated under reduced pressure. The crude product was then purified via column chromatography.

General Procedure F

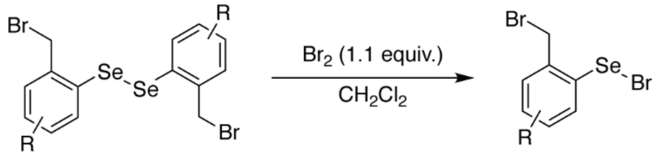

An oven-dried round-bottom flask equipped with a stir bar was charged with dibenzylbromide diselenide (1 equiv.) and CH_2Cl_2 (0.1 M). Into it was then added bromine (1.1 equiv.) and the reaction mixture was stirred for 1 h at RT. After completion, the volatiles were removed under reduced pressure to get the desired product which was used for next step without further purification.

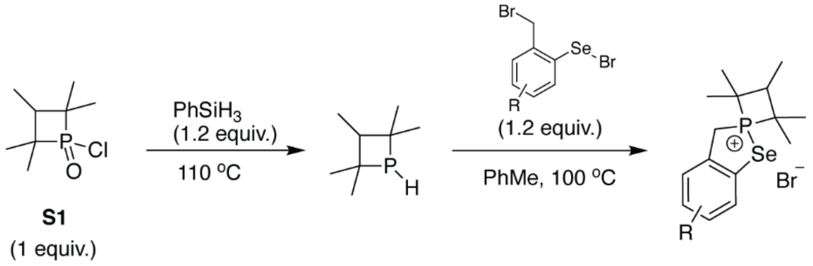

A flame-dried, two-neck round-bottom flask equipped with a stir bar and a condenser was charged with **S1** (1 equiv.). The reaction vessel was evacuated and backfilled with N_2 (3 cycles). $PhSiH_3$ (1.2 equiv.) was added, and the mixture was stirred at 110 °C for 2 h. The vessel was then removed from heat and cooled to RT over 15 min. At RT, selenenyl bromide (0.1 M in PhMe) was added dropwise over 10 min. The reaction mixture was heated to 100 °C and stirred for 4 h, during which the selenophosphonium salt precipitated. After 4 h, the vessel was removed from heat, cooled to RT, and the solid was collected by filtration. The product was washed with PhMe, then hexanes, and dried under vacuum. The obtained product was immediately used for the next step.

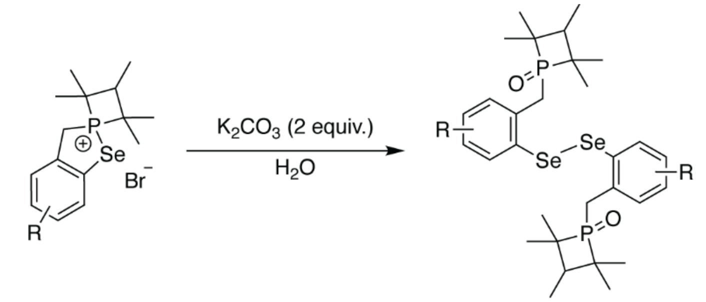

A round-bottom flask equipped with a stir bar was charged with selenophosphonium salt and H_2O (0.1 M). K_2CO_3 (2 equiv.) was added in one portion, instantly turning the suspension dark yellow. The mixture was stirred at RT for 1 h. The organic compounds were then extracted with CH_2Cl_2, and the combined organic layers were washed with brine, dried over Na_2SO_4, filtered, and concentrated under reduced pressure. The crude product was purified by column chromatography.

2,2'-diselanediyldibenzaldehyde (S3)

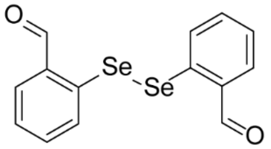

Prepared with general procedure A. The title compound was obtained by purification via silica gel chromatography (0% to 20% EtOAc in hexanes) as a yellow solid (1.84g, 84% yield). $R_f = 0.26$ (20% EtOAc in hexanes, UV).

^{1}H NMR (400 MHz, $CDCl_3$) δ 10.16 (s, 2H), 7.86 – 7.80 (m, 4H), 7.42 – 7.36 (m,4H).

^{13}C NMR (101 MHz, $CDCl_3$) δ 192.80, 135.72, 135.09, 134.78, 134.41, 131.33, 126.49.

^{77}Se NMR (76 MHz, $CDCl_3$) δ 458.08.

HRMS (ESI/Q-TOF) m/z: $[M + H]^+$ calculated for $C_{14}H_{11}O_2Se_2^+$ 370.9084; Found 370.9086

(diselanediylbis(2,1-phenylene))dimethanol (S4)

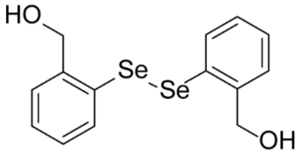

Prepared with general procedure D. The title compound was obtained as a yellow solid (1.21g, 94% yield). $R_f = 0.18$ (40% EtOAc in hexanes, UV).

^{1}H NMR (500 MHz, DMSO) δ 7.65 (d, *J* = 7.6 Hz, 2H), 7.37 (d, *J* = 7.3 Hz, 2H), 7.24 (ddd, *J* = 15.0, 10.4, 4.1 Hz, 4H), 5.51 (t, *J* = 5.3 Hz, 2H), 4.61 (d, *J* = 5.4 Hz, 4H).

^{13}C NMR (101 MHz, DMSO) δ 141.96 (s), 131.54 (s), 129.53 (s), 127.92 (s), 127.29 (d, *J* = 3.1 Hz), 63.16 (s).

^{77}Se NMR (76 MHz, DMSO) δ 398.35.

HRMS (ESI/Q-TOF) m/z: [M - H]$^-$ calculated for $C_{14}H_{13}O_2Se_2^-$ 372.9251; Found 372.9259

1,2-bis(2-(bromomethyl)phenyl)diselane (S5)

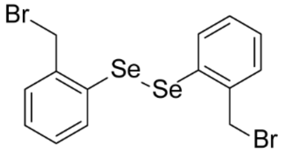

Prepared with general procedure E. The title compound was obtained by purification via silica gel chromatography (0% to 10% EtOAc in hexanes) as a yellow solid (819 mg, 82% yield). R_f = 0.53 (5% EtOAc in hexanes, UV).

^{1}H NMR (400 MHz, $CDCl_3$) δ 7.73 (dd, *J* = 7.7, 1.3 Hz, 2H), 7.37 (dd, *J* = 7.5, 1.5 Hz, 2H), 7.27 (td, *J* = 7.6, 1.6 Hz, 2H), 7.20 (td, *J* = 7.6, 1.6 Hz, 2H), 4.60 (s, 4H).

^{13}C NMR (101 MHz, $CDCl_3$) δ 139.64, 135.82, 132.65, 130.33, 129.79, 129.25, 33.91.

^{77}Se NMR (76 MHz, $CDCl_3$) δ 439.06.

HRMS (ESI/Q-TOF) m/z: [M + H]$^+$ calculated for $C_{14}H_{13}Br_2Se_2^+$ 498.7709; Found 498.7701

1,1'-((diselanediylbis(2,1-phenylene))bis(methylene))bis(2,2,3,4,4-pentamethylphosphetane 1-oxide) (3a)

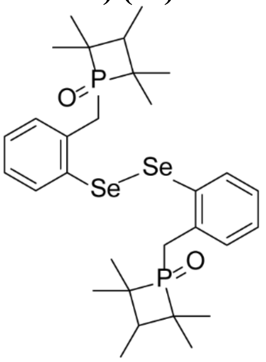

Prepared by following general procedure F. The title compound was obtained by purification via silica gel chromatography (0% to 10% MeOH in CH_2Cl_2) as a yellow solid (322 mg, 84% yield). R_f = 0.36 (5% MeOH in CH_2Cl_2, UV and $KMnO_4$).

^{1}H NMR (400 MHz, $CDCl_3$) δ 7.72 (d, *J* = 8.2 Hz, 2H), 7.62 (d, *J* = 7.7 Hz, 2H), 7.19 (td, *J* = 7.5, 1.3 Hz, 2H), 7.07 (dd, *J* = 10.8, 4.3 Hz, 2H), 3.31 (d, *J* = 10.2 Hz, 4H), 1.63 (qd, *J* = 7.0, 2.1 Hz, 2H), 1.20 (s, 6H), 1.16 (s, 6H), 1.07 (s, 6H), 1.02 (s, 6H), 0.84 (dd, *J* = 7.1, 1.6 Hz, 6H).

^{13}C NMR (101 MHz, $CDCl_3$) δ 136.87 (d, *J* = 1.1 Hz), 135.11 (d, *J* = 6.8 Hz), 134.51 (d, *J* = 6.6 Hz), 129.90 (d, *J* = 4.6 Hz), 129.05 (d, *J* = 1.1 Hz), 127.78 (d, *J* = 1.6 Hz), 46.91 (d, *J* = 56.8 Hz), 43.51 (d, *J* = 5.2 Hz), 31.58 (d, *J* = 33.8 Hz), 25.20 (d, *J* = 3.9 Hz), 24.28 (d, *J* = 4.7 Hz), 19.51 (d, *J* = 1.8 Hz), 17.88 (d, *J* = 4.5 Hz), 7.07 (d, *J* = 23.4 Hz).

^{31}P NMR (162 MHz, $CDCl_3$) δ 63.10 (minor), 58.01 (major).

^{77}Se NMR (76 MHz, $CDCl_3$) δ 469.80.

HRMS (ESI/Q-TOF) m/z: $[M + H]^+$ calculated for $C_{30}H_{45}O_2P_2Se_2^+$ 659.1220; Found 659.1239

Synthesis of (2-(2-bromoethyl)phenoxy)(tert-butyl)dimethylsilane (S6)

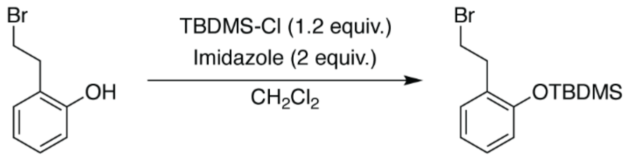

An oven-dried round-bottom flask equipped with a stir bar was charged with 2-(2-bromoethyl)phenol (4.00 g, 19.89 mmol) and CH_2Cl_2 (60 mL). TBDMS-Cl (3.60 g, 23.87 mmol) and imidazole (2.70 g, 39.80 mmol) were added, and the mixture was stirred for 12 h at RT. The reaction was quenched with water, and the organic compounds were extracted with CH_2Cl_2. The combined organic layers were washed with brine, dried over Na_2SO_4, filtered, and concentrated under reduced pressure. The crude product was purified by silica gel column chromatography, eluting with 100% hexanes, to afford the title compound **S6** (5.94 g, 95% yield) as a light brown. R_f = 0.72 (100% hexanes, UV).

^{1}H NMR (500 MHz, CDCl3) δ 7.18 – 7.11 (m, 2H), 6.90 (td, *J* = 7.4, 1.0 Hz, 1H), 6.81 (d, *J* = 8.0 Hz, 1H), 3.54 (t, *J* = 8.0 Hz, 2H), 3.15 (t, *J* = 8.0 Hz, 2H), 1.04 (s, 9H), 0.26 (s, 6H).

^{13}C NMR (101 MHz, $CDCl_3$) δ 153.93, 130.92, 129.56, 128.26, 121.26, 118.58, 35.16, 31.90, 25.91, 18.33, -4.01.

HRMS (ESI/Q-TOF) m/z: $[M + H]^+$ calculated for C14H24BrOSi$^+$ 315.0780; Found 315.0787

Synthesis of 1-(2-((tert-butyldimethylsilyl)oxy)phenethyl)-2,2,3,4,4-pentamethylphosphetane 1-oxide (S7)

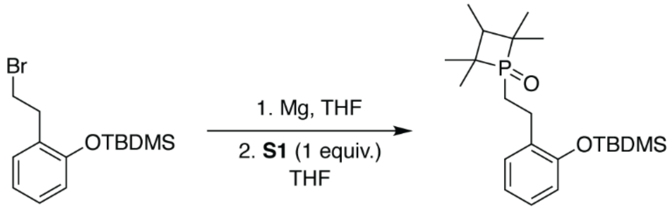

Grignard reagent preparation: A flame-dried round-bottom flask equipped with a stir bar was charged with magnesium turnings (440 mg, 18.1 mmol, 5 equiv.) and a tiny crystal of iodine. **S6** (1.71 g, 5.42 mmol) dissolved in THF (25 mL) was added over 1.5 h using a syringe pump. After addition, the reaction mixture was stirred for 1 h.

Separately, another flame-dried round-bottom flask equipped with a stir bar was charged with **S1** (703 mg, 3.61 mmol) and THF (10 mL). The Grignard reagent was then added dropwise to this mixture at RT over 15 min. After addition, the reaction was stirred for 12 h. Upon completion, the reaction was quenched with water, and the organic compounds were extracted with CH_2Cl_2. The combined organic layers were washed with brine, dried over Na_2SO_4, filtered, and concentrated under reduced pressure. The crude product was purified by silica gel column chromatography. Elution of the column with 100% CH_2Cl_2 followed by 5% MeOH in CH_2Cl_2 gave the title compound **S7** (1.2 g, 84% yield) as a light brown oil. R_f = 0.49 (5% MeOH in CH_2Cl_2, UV, $KMnO_4$).

^{1}H NMR (500 MHz, CDCl3) δ 7.17 (dd, J = 7.5, 1.4 Hz, 1H), 7.08 (td, J = 7.9, 1.6 Hz, 1H), 6.85 (dd, J = 10.8, 4.0 Hz, 1H), 6.77 (d, J = 8.0 Hz, 1H), 4.23 (q, J = 6.8 Hz, 2H), 2.99 (t, J = 7.0 Hz, 2H), 1.47 (qd, J = 7.1, 4.0 Hz, 1H), 1.22 (s, 3H), 1.18 (s, 3H), 1.11 (s, 3H), 1.07 (s, 3H), 1.00 (s, 9H), 0.83 (dd, J = 7.2, 1.4 Hz, 3H), 0.23 (s, 6H).

^{13}C NMR (101 MHz, $CDCl_3$) δ 153.89 (s), 131.41 (s), 128.14 (s), 127.75 (s), 121.00 (s), 118.38 (s), 63.87 (d, J = 7.6 Hz), 50.54 (s), 49.82 (s), 42.28 (d, J_{PC} = 10.5 Hz), 32.45 (d, J_{PC} = 5.9 Hz), 25.88 (s), 23.92 (d, J_{PC} = 6.6 Hz), 18.32 (s), 18.28 (d, J = 2.8 Hz),7.11 (d, J_{PC} = 24.2 Hz), 7.11 (d, J_{PC} = 24.2 Hz), -4.06 (s).

^{31}P NMR (162 MHz, $CDCl_3$) δ 57.61 (major), 56.53 (minor).

HRMS (ESI/Q-TOF) m/z: $[M + H]^+$ calculated for $C_{22}H_{40}O_2PSi^+$ 395.2539; Found 395.2535

Synthesis of 1-(2-hydroxyphenethyl)-2,2,3,4,4-pentamethylphosphetane 1-oxide (S8)

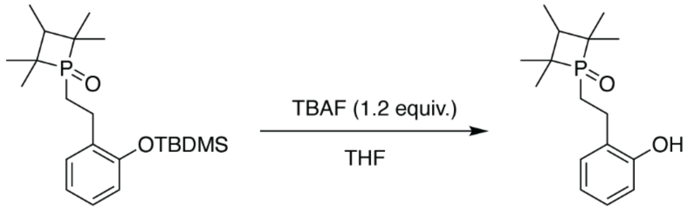

An oven dried round bottom flask equipped with a stir bar was charged with **S7** (500 mg, 1.27 mmol) and THF (10 mL). To this was then added TBAF (1.0 M in THF, 1.52 mL) and the reaction mixture was stirred for 2 hours. After completion, the volatiles were removed under vacuum and the crude product was purified by silica gel chromatography. Elution of the column with 5% MeOH in CH_2Cl_2 gave the title compound **S8** (336 mg, 94% yield) as a white solid. R_f = 0.41 (5% MeOH in CH_2Cl_2, UV, $KMnO_4$).

^{1}H NMR (400 MHz, CDCl3) δ 8.32 (s, 1H), 7.07 (ddd, *J* = 7.3, 4.1, 1.3 Hz, 2H), 6.89 (dd, *J* = 8.5, 1.2 Hz, 1H), 6.76 (td, *J* = 7.4, 1.2 Hz, 1H), 4.28 (q, *J* = 7.2 Hz, 2H), 3.04 (t, *J* = 7.1 Hz, 2H), 1.57 (qd, *J* = 7.2, 4.3 Hz, 1H), 1.25 (s, 3H), 1.21 (s, 3H), 1.17 (s, 3H), 1.12 (s, 3H), 0.87 (dd, *J* = 7.2, 1.6 Hz, 3H).
^{13}C NMR (101 MHz, $CDCl_3$) δ 155.78 (s), 131.06 (s), 128.09 (s), 123.58 (s), 119.48 (s), 115.93 (s), 64.90 (d, J_{PC} = 7.6 Hz), 42.48 (d, J_{PC} = 10.7 Hz), 32.96 (d, J_{PC} = 5.3 Hz), 23.90 (d, J_{PC} = 6.6 Hz), 18.31 (d, J_{PC} = 2.9 Hz), 7.23 (d, J_{PC} = 24.4 Hz).

^{31}P NMR (162 MHz, $CDCl_3$) δ 59.82 (major), 58.86 (minor).

HRMS (ESI/Q-TOF) m/z: $[M - H]^-$ calculated for $C_{16}H_{24}O_2P^-$ 279.1514; Found 279.1512

Synthesis of 2-(2-(2,2,3,4,4-pentamethyl-1-oxidophosphetan-1-yl)ethyl)phenyl trifluoromethanesulfonate (S9)

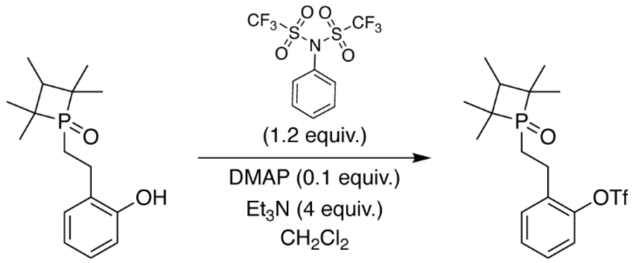

An oven dried round bottom flask equipped with a stir bar was charged with **S8** (1.00 g, 3.56 mmol) and CH_2Cl_2 (30 mL). Into it was added phenyl triflimide (1.53 g, 4.28 mmol), DMAP (44 mg, 0.35 mmol) and Et_3N (2 mL, 14.27 mmol). The reaction mixture was stirred at RT overnight. After completion, all the volatiles were removed under vacuum and the crude product was purified by silica gel chromatography. Elution of the column with 100% CH_2Cl_2 followed by 3% MeOH

in CH_2Cl_2 gave the title compound **S9** (1.31 g, 89% yield) as a light red solid. R_f = 0.30 (3% MeOH in CH_2Cl_2, UV, $KMnO_4$).

^{1}H NMR (500 MHz, $CDCl_3$) δ 7.47 (dd, *J* = 7.4, 1.5 Hz, 1H), 7.36 – 7.26 (m, 3H), 3.14 (dt, *J* = 11.6, 8.0 Hz, 2H), 2.14 (dt, *J* = 11.5, 8.4 Hz, 2H), 1.59 (qd, *J* = 7.0, 1.5 Hz, 1H), 1.29 (s, 3H), 1.26 (s, 3H), 1.14 (s, 3H), 1.11 (s, 3H), 0.88 (dd, *J* = 7.1, 1.4 Hz, 3H).

^{13}C NMR (126 MHz, $CDCl_3$) δ 148.24 (s), 134.61 (d, *J* = 11.4 Hz), 131.80 (s), 128.90 (s), 128.61 (s), 121.45 (s), 119.97 (s), 117.42 (s), 46.06 (d, *J* = 57.1 Hz), 42.90 (d, *J* = 5.7 Hz), 25.31 (d, *J* = 36.6 Hz), 24.83 (d, *J* = 3.6 Hz), 22.56 (d, *J* = 3.3 Hz), 17.83 (d, *J* = 4.4 Hz), 7.10 (d, *J* = 23.0 Hz).

^{19}F NMR (471 MHz, $CDCl_3$) δ -73.60.

^{31}P NMR (202 MHz, $CDCl_3$) δ 64.78 (minor), 59.18 (major).

HRMS (ESI/Q-TOF) m/z: $[M + H]^+$ calculated for $C_{17}H_{25}F_3O_4PS^+$ 413.1158; Found 413.1155

Synthesis of 2,2,3,4,4-pentamethyl-1-(2-(4,4,5,5-tetramethyl-1,3,2-dioxaborolan-2-yl)phenethyl)phosphetane 1-oxide (S10)

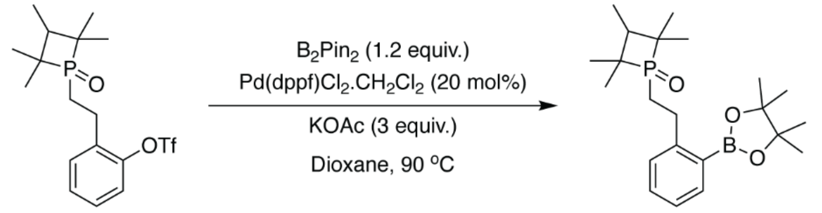

A flame dried 2-neck round bottom flask equipped with a stir bar and a reflux condenser was chaged with **S7** (1.23 g, 2.98 mmol), B_2Pin_2 (909 mg, 3.58 mmol), KOAc (878 mg, 8.94 mmol) and dioxane (30 mL). The reaction vessel was evacuated and backfilled with N_2 (3 cycles). To this was then added $Pd(dppf)Cl_2.CH_2Cl_2$ (487 mg, 0.59 mmol) and the reaction mixture was stirred at 90 °C for 24 hours. After completion, the volatiles were removed under vacuum and the crude product was purified by silica gel chromatography. Elution of the column with 30% EtOAc in hexanes followed by elution of 50% EtOAc in hexanes and 80% EtOAc in hexanes gave the title complound **S8** (877 mg, 75% yield) as a clear oil. R_f = 0.36 (70% EtOAc in hexanes, UV, $KMnO_4$).

^{1}H NMR (400 MHz, $CDCl_3$) δ 7.26 – 7.22 (m, 1H), 7.20 – 7.14 (m, 1H), 7.12 – 7.04 (m, 1H), 3.16 (dt, *J* = 11.1, 7.7 Hz, 2H), 2.04 (dt, *J* = 11.1, 7.7 Hz, 2H), 1.43 (dq, *J* = 7.1, 5.1 Hz, 1H), 1.20 (s, 12H), 1.16 (s, 3H), 1.12 (s, 3H), 1.04 (s, 3H), 1.00 (s, 3H), 0.74 (dd, *J* = 7.1, 1.6 Hz, 3H).

^{13}C NMR (101 MHz, CDCl3) δ 149.07 (s), 148.95 (s), 136.57 (s), 131.49 (s), 129.44 (s), 128.67 (s), 128.14 (s), 125.57 (s), 83.63 (s), 45.60 (d, *J* = 57.2 Hz), 42.87 (d, *J* = 5.6 Hz), 27.77 (d, *J* =

37.5 Hz), 27.25 (d, J = 3.5 Hz), 24.99 (d, J = 3.3 Hz), 24.85 (s), 17.82 (d, J = 4.5 Hz), 7.02 (d, J = 22.7 Hz).

^{31}P NMR (162 MHz, $CDCl_3$) δ 59.76.

HRMS (ESI/Q-TOF) m/z: $[M + H]^+$ calculated for $C_{22}H_{37}BO_3P^+$ 391.2568; Found 391.2549

Synthesis of 1,1'-((diselanediylbis(2,1-phenylene))bis(ethane-2,1-diyl))bis(2,2,3,4,4-pentamethylphosphetane 1-oxide) (3b)

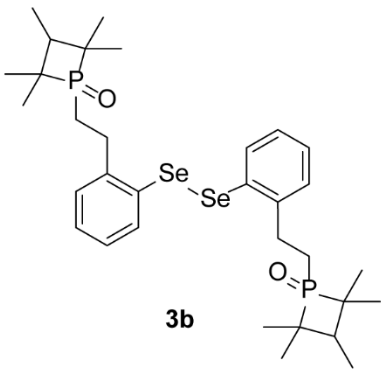

3b

3b was synthesized from **S10** following general procedure A .
Purified by silica gel chromatography. Elution of the column with 0% to 10% MeOH in CH_2Cl_2 gave the title compound **3b** (504 mg, 85% yield) as a yellow solid. R_f = 0.36 (10% MeOH in CH_2Cl_2, UV, $KMnO_4$).

^{1}H NMR (500 MHz, $CDCl_3$) δ 7.60 (d, J = 7.6 Hz, 2H), 7.30 (d, J = 7.0 Hz, 2H), 7.23 (t, J = 7.1 Hz, 2H), 7.12 – 7.07 (m, 2H), 3.21 – 3.13 (m, 4H), 2.13 – 2.05 (m, 4H), 1.54 – 1.49 (m, 2H), 1.28 (d, J = 7.7 Hz, 6H), 1.24 (s, 6H), 1.10 (s, 6H), 1.07 (s, 6H), 0.86 (d, J = 7.1 Hz, 6H).

^{13}C NMR (101 MHz, $CDCl_3$) δ 143.51 (d, J = 11.6 Hz), 135.12 (s), 131.01 (s), 129.76 (s), 129.36 (s), 127.79 (s), 45.91 (d, J = 57.2 Hz), 42.83 (d, J = 5.7 Hz), 28.26 (d, J = 3.4 Hz), 26.41 (s), 26.05 (s), 25.04 (d, J = 3.4 Hz), 17.85 (d, J = 4.4 Hz), 7.07 (d, J = 23.0 Hz).

^{31}P NMR (202 MHz, $CDCl_3$) δ 59.40.

^{77}Se NMR (76 MHz, $CDCl_3$) δ 431.49.

HRMS (ESI/Q-TOF) m/z: $[M + H]^+$ calculated for $C_{32}H_{49}O_2P_2Se_2^+$ 687.1533; Found 687.1533

1,1-bis(2-bromo-5-methoxybenzyl)-2,2,3,4,4-pentamethylphosphetan-1-ium bromide (S11)

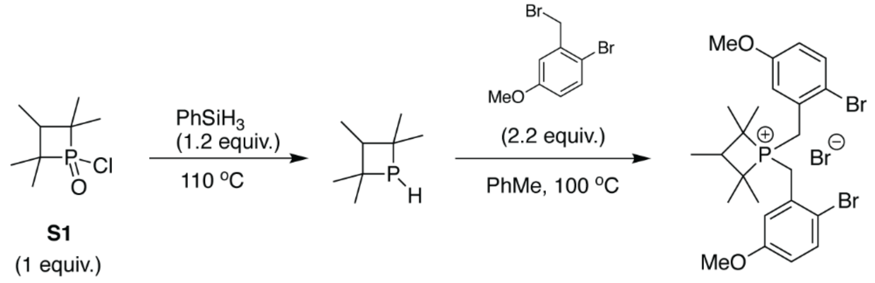

A flame-dried, two-neck round-bottom flask equipped with a stir bar and a condenser was charged with **S1** (560 mg, 2.87 mmol). The reaction vessel was evacuated and backfilled with N_2 (3 cycles). $PhSiH_3$ (428 μL, 3.45 mmol) was added, and the mixture was stirred at 110 °C for 2 h. The vessel was then removed from heat and cooled to RT over 15 min. At RT, 1-bromo-2-(bromomethyl)-4-methoxybenzene (1.77 g, 6.32 mmol) (0.1 M in PhMe) was added dropwise over 10 min. The reaction mixture was heated to 100 °C and stirred for 4 h, during which the product precipitated. After 2 h, the vessel was removed from heat, cooled to RT. The solid was collected by filtration, washed with PhMe, then hexanes, and dried under vacuum to get the title compound **S11** (1.32 g, 75% yield) as a white solid.

^{1}H NMR (400 MHz, CDCl3) δ 7.35 (d, J = 8.8 Hz, 1H), 7.30 – 7.26 (m, 1H), 6.72 – 6.68 (m, 1H), 6.66 – 6.61 (m, 1H), 4.37 (d, J = 13.9 Hz, 2H), 4.25 (d, J = 13.9 Hz, 2H), 3.78 (s, 3H), 3.58 (s, 3H), 2.98 (qd, J = 6.8, 2.1 Hz, 1H), 1.66 (s, 3H), 1.61 (s, 3H), 1.58 (s, 3H), 1.53 (s, 3H), 1.03 (d, J = 6.7 Hz, 3H).

^{13}C NMR (101 MHz, DMSO) δ 158.52 (s), 158.17 (d, J = 1.1 Hz), 133.57 (s), 133.06 (d, J = 1.4 Hz), 130.72 (d, J = 9.2 Hz), 129.63 (d, J = 7.1 Hz), 116.98 (d, J = 5.8 Hz), 116.01 (d, J = 2.0 Hz), 115.37 (d, J = 4.7 Hz), 115.12 (s), 114.64 (d, J = 9.3 Hz), 113.81 (d, J = 6.1 Hz), 55.35 (d, J = 38.4 Hz), 49.40 (d, J = 8.1 Hz), 43.11 (d, J = 40.7 Hz), 25.74 (d, J = 14.7 Hz), 24.80 (d, J = 24.0 Hz), 24.46 (d, J = 4.1 Hz), 18.78 (d, J = 2.1 Hz), 7.98 (d, J = 20.2 Hz).

^{31}P NMR (162 MHz, DMSO) δ 63.29.

HRMS (ESI/Q-TOF) m/z: $[M - Br]^+$ calculated for $C_{24}H_{32}Br_2O_2P^+$ 541.0507; Found 541.0510

1-(2-bromo-5-methoxybenzyl)-2,2,3,4,4-pentamethylphosphetane 1-oxide (S12)

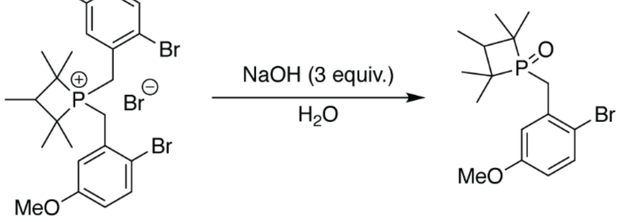

A round-bottom flask equipped with a stir bar was charged with **S11** (500 mg, 0.8 mmol) and H_2O 5 mL). NaOH (96 mg, 2.41 mmol) was added in one portion. The mixture was stirred at RT for 1 h. The organic compounds were then extracted with CH_2Cl_2, and the combined organic layers were washed with brine, dried over Na_2SO_4, filtered, and concentrated under reduced pressure. The crude product was purified by silica gel chromatography. Elution of the column with 30% EtOAc in hexanes followed by 100% EtOAc gave the title complound **S12** (280 mg, 97% yield) as a white solid. R_f = 0.20 (80% EtOAc, UV, $KMnO_4$).

^{1}H NMR (400 MHz, $CDCl_3$) δ 7.70 (dd, *J* = 3.0, 1.5 Hz, 1H), 7.40 (dd, *J* = 8.8, 0.6 Hz, 1H), 6.68 (dd, *J* = 8.8, 2.5 Hz, 1H), 3.79 (s, 3H), 3.44 (d, *J* = 10.5 Hz, 2H), 1.77 (qd, *J* = 7.1, 2.1 Hz, 1H), 1.30 (s, 3H), 1.25 (s, 3H), 1.17 (s, 3H), 1.12 (s, 3H), 0.91 (dd, *J* = 7.1, 1.7 Hz, 3H).

^{13}C NMR (101 MHz, $CDCl_3$) δ 159.05 (d, *J* = 1.0 Hz), 133.55 (d, *J* = 5.9 Hz), 133.33 (s), 115.80 (d, *J* = 1.2 Hz), 115.72 (d, *J* = 4.3 Hz), 115.60 (s), 55.63 (s), 46.87 (d, *J* = 57.4 Hz), 43.42 (d, *J* = 5.5 Hz), 30.70 (d, *J* = 34.8 Hz), 24.90 (d, *J* = 4.0 Hz), 18.10 (d, *J* = 4.4 Hz), 7.25 (d, *J* = 23.5 Hz).

^{31}P NMR (162 MHz, $CDCl_3$) δ 64.29 (minor), 59.32 (major).

HRMS (ESI/Q-TOF) m/z: $[M - Br]^+$ calculated for $C_{16}H_{25}BrO_2P^+$ 359.0776; Found 359.0777

1-(5-methoxy-2-(4,4,5,5-tetramethyl-1,3,2-dioxaborolan-2-yl)benzyl)-2,2,3,4,4-pentamethylphosphetane 1-oxide (S13)

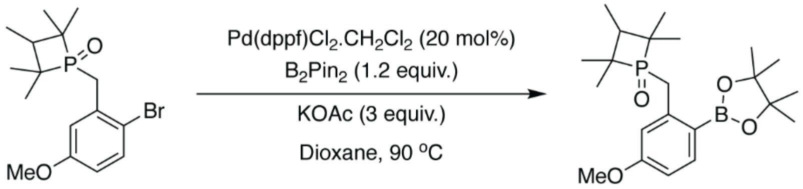

A flame dried 2-neck round bottom flask equipped with a stir bar and a reflux condenser was chaged with **S12** (560 mg, 1.55 mmol), B_2Pin_2 (475 mg, 1.87 mmol), KOAc (460 mg, 4.67 mmol) and dioxane (15 mL). The reaction vessel was evacuated and backfilled with N_2 (3 cycles). To this was then added $Pd(dppf)Cl_2.CH_2Cl_2$ (255 mg, 0.31 mmol) and the reaction mixture was stirred at 90 °C for 24 hours. After completion, the volatiles were removed under vacuum and the crude product was purified by silica gel chromatography. Elution of the column with 30% EtOAc in hexanes followed by elution of 50% EtOAc in hexanes and 80% EtOAc in hexanes gave the title complound **S13** (210 mg, 33% yield) as a clear oil. R_f = 0.24 (80% EtOAc in hexanes, UV, $KMnO_4$).

^{1}H NMR (500 MHz, $CDCl_3$) δ 7.73 (d, *J* = 8.4 Hz, 1H), 7.41 (d, *J* = 1.9 Hz, 1H), 6.77 – 6.73 (m, 1H), 3.84 (d, *J* = 11.2 Hz, 2H), 3.81 (d, *J* = 1.3 Hz, 3H), 1.75 (q, *J* = 7.0 Hz, 1H), 1.33 (s, 12H), 1.25 (s, 3H), 1.22 (s, 3H), 1.15 (s, 3H), 1.11 (s, 3H), 0.89 (d, *J* = 7.2 Hz, 3H).

^{13}C NMR (126 MHz, $CDCl_3$) δ 161.93 (d, *J* = 1.6 Hz), 141.60 (d, *J* = 7.6 Hz), 138.10 (s), 114.96 (d, *J* = 4.4 Hz), 112.51 (s), 83.52 (s), 60.52 (s), 55.27 (s), 47.08 (s), 46.62 (s), 43.48 (d, *J* = 5.2

Hz), 30.20 (d, J = 35.2 Hz), 25.19 (s), 25.05 (d, J = 3.7 Hz), 25.01 (s), 18.25 (d, J = 4.3 Hz), 7.20 (d, J = 23.1 Hz).

^{31}P NMR (162 MHz, $CDCl_3$) δ 58.89.

HRMS (ESI/Q-TOF) m/z: $[M + H]^+$ calculated for $C_{22}H_{37}BO_4P^+$ 407.2523; Found 407.2523

1,1'-((diselanediylbis(5-methoxy-2,1-phenylene))bis(methylene))bis(2,2,3,4,4-pentamethylphosphetane 1-oxide) (3c)

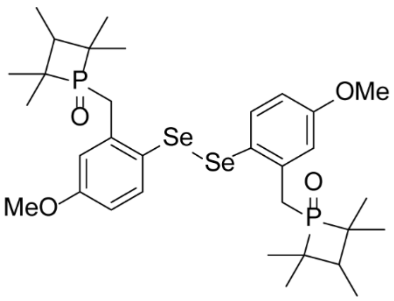

3c was synthesized from **S13** following general procedure A .
Purified by silica gel chromatography. Elution of the column with 0% to 10% MeOH in CH_2Cl_2 gave the title compound **3c** (233 mg, 80% yield) as a yellow solid. R_f = 0.36 (5% MeOH in CH_2Cl_2, UV, $KMnO_4$).

^{1}H NMR (500 MHz, $CDCl_3$) δ 7.59 (d, J = 8.6 Hz, 1H), 7.35 (s, 1H), 6.68 (dd, J = 8.6, 2.5 Hz, 1H), 3.79 (s, 3H), 3.28 (d, J = 10.1 Hz, 2H), 1.67 – 1.62 (m, 1H), 1.23 (s, 3H), 1.20 (s, 3H), 1.05 (s, 3H), 1.01 (s, 3H), 0.87 (dd, J = 7.0, 0.9 Hz, 3H).

^{13}C NMR (126 MHz, CDCl3) δ 160.89 (s), 139.63 (s), 137.89 (d, J = 6.6 Hz), 124.39 (d, J = 6.9 Hz), 115.05 (d, J = 4.2 Hz), 114.46 (s), 55.53 (s), 46.90 (d, J = 56.8 Hz), 43.44 (d, J = 5.1 Hz), 31.55 (d, J = 33.5 Hz), 25.12 (d, J = 3.7 Hz), 18.04 (d, J = 4.3 Hz), 7.23 (d, J = 23.6 Hz).

^{31}P NMR (202 MHz, $CDCl_3$) δ 63.10, 59.24.

^{77}Se NMR (76 MHz, $CDCl_3$) δ 478.15.

HRMS (ESI/Q-TOF) m/z: $[M + H]^+$ calculated for $C_{32}H_{49}O_4P_2Se_2^+$ 719.1437; Found 719.1437

2-((4-methoxybenzyl)selanyl)-6-(trifluoromethyl)benzaldehyde (S14)

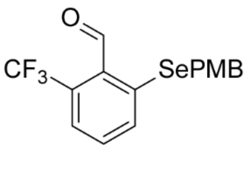

Prepared with general procedure B. The title compound was obtained by purification via silica gel chromatography (0% to 20% EtOAc in hexanes) as a yellow solid (69% yield). R_f = 0.33 (10% EtOAc in hexanes, UV).

^{1}H NMR (400 MHz, $CDCl_3$) δ 10.46 (tt, J = 2.1, 1.1 Hz, 1H), 7.78 (d, J = 8.1 Hz, 1H), 7.61 (d, J = 7.2 Hz, 1H), 7.55 – 7.49 (m, 1H), 7.32 – 7.28 (m, 2H), 6.89 – 6.82 (m, 2H), 4.12 (s, 2H), 3.80 (s, 3H).

^{13}C NMR (101 MHz, $CDCl_3$) δ 189.64 (q, J = 3.1 Hz), 159.00 (s), 142.93 (s), 133.67 (q, J = 31.9 Hz), 132.49 (s), 132.18 (s), 131.04 (d, J = 0.9 Hz), 130.48 (s), 127.77 (s), 125.14 (s), 122.83 (q, J = 6.1 Hz), 122.41 (s), 114.34 (s), 55.42 (s), 30.29 (s).

^{19}F NMR (377 MHz, $CDCl_3$) δ -55.15.

^{77}Se NMR (76 MHz, $CDCl_3$) δ 417.25 (t, J = 8.9 Hz).

HRMS (ESI/Q-TOF) m/z: $[M + Na]^+$ calculated for $C_{16}H_{13}F_3NaO_2Se^+$ 396.9931; Found 396.9935

6,6'-diselanediylbis(2-(trifluoromethyl)benzaldehyde) (S15)

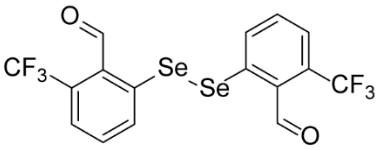

Prepared with general procedure C. The title compound was obtained as a yellow solid (83% yield). R_f = 0.51 (10% EtOAc in hexanes, UV).

^{1}H NMR (400 MHz, $CDCl_3$) δ 10.57 (dt, J = 2.0, 1.4 Hz, 2H), 8.09 (d, J = 8.2 Hz, 2H), 7.70 (d, J = 7.6 Hz, 2H), 7.52 – 7.46 (m, 2H).

^{13}C NMR (101 MHz, $CDCl_3$) δ 190.11 (dd, J = 6.7, 3.3 Hz), 138.36 (s), 135.04 (s), 134.14 (dd, J = 64.3, 32.1 Hz), 133.15 (s), 131.41 (s), 124.72 – 124.35 (m, J = 6.0 Hz), 123.63 (d, J = 275.1 Hz).

^{19}F NMR (377 MHz, $CDCl_3$) δ -54.79.

77Se NMR (76 MHz, $CDCl_3$) δ 517.70.

HRMS (ESI/Q-TOF) m/z: $[M + Na]^+$ calculated for $C_{16}H_8F_6NaO_2Se_2^+$ 528.8657; Found 528.8663

(diselanediylbis(6-(trifluoromethyl)-2,1-phenylene))dimethanol (S16)

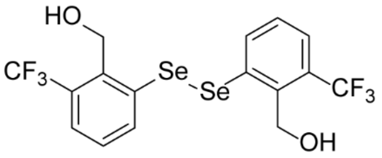

Prepared with general procedure D. The title compound was obtained as a yellow solid (96% yield). R_f = 0.63 (5% MeOH in CH_2Cl_2, UV).

^{1}H NMR (500 MHz, DMSO) δ 8.01 (d, *J* = 8.0 Hz, 2H), 7.61 (d, *J* = 7.7 Hz, 2H), 7.44 (t, *J* = 7.9 Hz, 2H), 5.94 (t, *J* = 4.9 Hz, 2H), 4.78 (d, *J* = 4.7 Hz, 4H).

^{13}C NMR (101 MHz, DMSO) δ 138.93 (s), 135.57 (s), 135.06 (s), 128.71 (s), 126.98 (d, *J* = 29.5 Hz), 125.34 (s), 124.24 (d, *J* = 6.1 Hz), 59.36 (s).

^{19}F NMR (471 MHz, DMSO) δ -56.83.

^{77}Se NMR (76 MHz, $CDCl_3$) δ 438.83.

HRMS (ESI/Q-TOF) m/z: $[M + Na]^+$ calculated for $C_{16}H_{11}F_6O_2Se_2^-$ 508.8994; Found 508.8997

1,2-bis(2-(bromomethyl)-3-(trifluoromethyl)phenyl)diselane (S17)

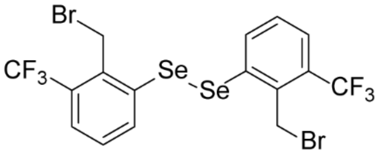

Prepared with general procedure E. The title compound was obtained by purification via silica gel chromatography (0% to 10% EtOAc in hexanes) as a yellow solid (81% yield). R_f = 0.0.58 (10% EtOAc in hexanes, UV).

^{1}H NMR (400 MHz, CDCl3) δ 7.99 (d, *J* = 7.9 Hz, 2H), 7.62 (d, *J* = 7.4 Hz, 2H), 7.34 (td, *J* = 7.9, 0.7 Hz, 2H), 4.78 (s, 4H).

^{13}C NMR (101 MHz, $CDCl_3$) δ 138.11 (s), 136.69 (s), 136.58 (s), 129.65 – 129.41 (m), 129.47 (d, *J* = 30.4 Hz), 126.47 (q, *J* = 5.7 Hz), 123.76 (d, *J* = 274.8 Hz), 28.58 (d, *J* = 2.7 Hz).

^{19}F NMR (377 MHz, $CDCl_3$) δ -59.18.

^{77}Se NMR (76 MHz, $CDCl_3$) δ 442.38.

HRMS (ESI/Q-TOF) m/z: $[M + NH]^+$ calculated for $C_{16}H_{11}Br_2F_6Se_2^+$ 634.7462; Found 634.7463

1,1'-((diselanediylbis(6-(trifluoromethyl)-2,1-phenylene))bis(methylene))bis(2,2,3,4,4-pentamethylphosphetane 1-oxide) (3d)

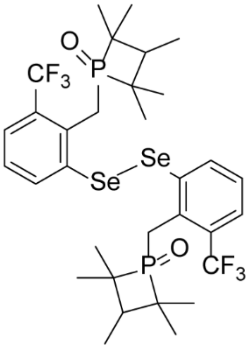

Prepared from **S17** by following general procedure F. The title compound was obtained by purification via silica gel chromatography (100% EtOAc) as a yellow solid (80% yield). R_f = 0.24 (100% EtOAc, UV and $KMnO_4$).

^{1}H NMR (400 MHz, $CDCl_3$) δ 7.78 (d, *J* = 7.8 Hz, 2H), 7.63 (d, *J* = 7.4 Hz, 2H), 7.19 (t, *J* = 7.9 Hz, 2H), 3.48 (d, *J* = 9.3 Hz, 4H), 1.72 (p, *J* = 7.2 Hz, 2H), 1.27 (s, 6H), 1.23 (s, 12H), 1.19 (s, 6H), 0.90 (dd, *J* = 7.2, 1.5 Hz, 6H).

^{13}C NMR (101 MHz, $CDCl_3$) δ 141.26 (s), 138.44 (d, *J* = 4.3 Hz), 135.21 (d, *J* = 8.5 Hz), 129.40 (d, *J* = 4.2 Hz), 129.11 (d, *J* = 4.3 Hz), 127.39 (d, *J* = 2.0 Hz), 127.16 (dd, *J* = 5.7, 1.4 Hz), 47.71 (d, *J* = 55.7 Hz), 45.10 (d, *J* = 5.1 Hz), 27.49 (d, *J* = 27.5 Hz), 26.07 (s), 18.47 (d, *J* = 4.6 Hz), 8.51 (d, *J* = 19.6 Hz).

^{19}F NMR (377 MHz, $CDCl_3$) δ -58.93.

^{31}P NMR (162 MHz, $CDCl_3$) δ 58.76.

^{77}Se NMR (76 MHz, $CDCl_3$) δ 490.33.

HRMS (ESI/Q-TOF) m/z: $[M + NH]^+$ calculated for $C_{32}H_{43}F_6O_2P_2Se_2^+$ 795.0973; Found 795.0973

6,6'-diselanediylbis(3-fluorobenzaldehyde) (S18)

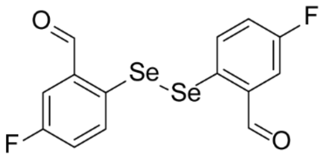

Synthesized following general procedure A. The title compound was obtained by purification via silica gel chromatography (0% to 20% EtOAc in hexanes) as a yellow solid (86% yield). R_f = 0.29 (10% EtOAc in hexanes, UV, $KMnO_4$).

^{1}H NMR (500 MHz, $CDCl_3$) δ 10.12 (s, 2H), 7.78 (dd, J = 8.7, 4.9 Hz, 2H), 7.58 (dd, J = 7.9, 2.6 Hz, 2H), 7.17 (td, J = 8.4, 2.7 Hz, 2H).

^{13}C NMR (101 MHz, $CDCl_3$) δ 191.69 (d, J = 2.0 Hz), 163.23 (s), 160.76 (s), 135.68 (d, J = 5.2 Hz), 133.31 (d, J = 6.8 Hz), 128.95 (d, J = 3.0 Hz), 121.78 (dd, J = 67.0, 21.7 Hz).
^{77}Se NMR (76 MHz, CDCl3) δ 458.25.

^{19}F NMR (471 MHz, $CDCl_3$) δ -116.09.

HRMS (ESI/Q-TOF) m/z: $[M + Na]^+$ calculated for $C_{14}H_8F_2NaO_2Se_2^+$ 428.8720; Found 428.8726

(diselanediylbis(5-fluoro-2,1-phenylene))dimethanol (S19)

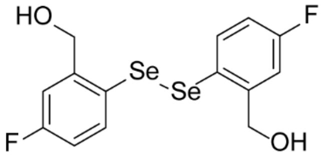

Synthesized follwing general procedure D. The title compound was obtained as a yellow solid (98% yield). R_f = 0.5 (5% MeOH in CH_2Cl_2, UV, $KMnO_4$).

^{1}H NMR (500 MHz, DMSO) δ 7.60 (dd, J = 8.4, 5.9 Hz, 2H), 7.27 – 7.19 (m, 2H), 7.08 (td, J = 8.5, 2.5 Hz, 2H), 5.59 – 5.56 (m, 2H), 4.52 (s, 4H).

^{13}C NMR (101 MHz, DMSO) δ 163.60 (s), 161.16 (s), 145.92 (d, J = 6.9 Hz), 135.31 (d, J = 8.1 Hz), 123.86 (d, J = 2.9 Hz), 114.36 (dd, J = 80.8, 22.1 Hz), 62.74 (s).

^{19}F NMR (471 MHz, DMSO) δ -113.88.

^{77}Se NMR (76 MHz, DMSO) δ 411.35.

HRMS (ESI/Q-TOF) m/z: $[M - Na]^-$ calculated for $C_{14}H_{11}F_2O_2Se_2^-$ 408.9058; Found 408.9060

1,2-bis(2-(bromomethyl)-4-fluorophenyl)diselane (S20)

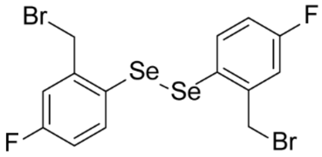

Prepared from **S19** following general procedure E. The title compound was obtained by purification via silica gel chromatography (0% to 10% EtOAc in hexanes) as a dark brown liquid (75% yield). R_f = 0.59 (10% EtOAc in hexanes, UV, $KMnO_4$).

^{1}H NMR (500 MHz, $CDCl_3$) δ 7.61 (dd, *J* = 8.6, 5.8 Hz, 2H), 7.14 (dd, *J* = 9.1, 2.7 Hz, 2H), 6.93 (td, *J* = 8.4, 2.7 Hz, 2H), 4.51 (s, 4H).

^{13}C NMR (101 MHz, CDCl3) δ 164.58 (s), 162.09 (s), 142.61 (d, *J* = 7.8 Hz), 138.79 (d, *J* = 8.1 Hz), 127.04 (d, *J* = 3.5 Hz), 117.17 (dd, *J* = 40.7, 22.0 Hz), 33.16 (s).

^{19}F NMR (471 MHz, $CDCl_3$) δ -110.79.

^{77}Se NMR (76 MHz, $CDCl_3$) δ 451.49.

HRMS (ESI/Q-TOF) m/z: $[M + H]^+$ calculated for $C_{14}H_{11}Br_2F_2Se_2^+$ 534.7526; Found 534.7526

1,1'-((diselanediylbis(5-fluoro-2,1-phenylene))bis(methylene))bis(2,2,3,4,4-pentamethylphosphetane 1-oxide) (3e)

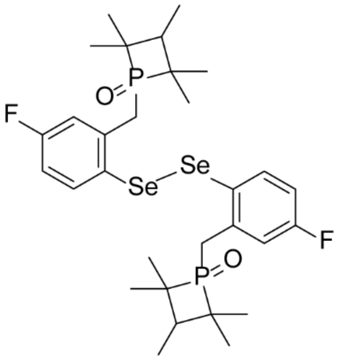

Prepared following general procedure F. The title compound was obtained by purification via silica gel chromatography (100% EtOAc) as a yellow solid (86% yield). R_f = 0.22 (100% EtOAc, UV and $KMnO_4$).

^{1}H NMR (400 MHz, $CDCl_3$) δ 7.67 (dd, *J* = 8.6, 6.0 Hz, 2H), 7.38 (d, *J* = 8.5 Hz, 2H), 6.83 (td, *J* = 8.3, 2.5 Hz, 2H), 3.28 (d, *J* = 10.0 Hz, 4H), 1.67 – 1.61 (m, 2H), 1.23 (s, 6H), 1.19 (s, 6H), 1.10 (s, 6H), 1.05 (s, 6H), 0.87 (d, *J* = 6.7 Hz, 6H).

^{13}C NMR (101 MHz, CDCl3) δ 164.44 (s), 161.96 (s), 139.23 (d, *J* = 8.2 Hz), 138.45 – 137.48 (m), 129.50 (d, *J* = 6.0 Hz), 116.97 (dd, *J* = 22.8, 4.0 Hz), 115.16 (d, *J* = 21.1 Hz), 47.16 (d, *J* = 56.8 Hz), 43.33 (d, *J* = 4.9 Hz), 31.82 (d, *J* = 32.5 Hz), 29.79 (s), 25.16 (d, *J* = 3.7 Hz), 24.35 (s), 19.61 (s), 17.92 (d, *J* = 4.3 Hz), 7.21 (d, *J* = 23.6 Hz).

^{77}Se NMR (76 MHz, $CDCl_3$) δ 477.71.

31P NMR (162 MHz, CDCl3) δ 62.52 (minor), 57.74 (major).

^{19}F NMR (377 MHz, $CDCl_3$) δ -111.05.

HRMS (ESI/Q-TOF) m/z: $[M + H]^+$ calculated for $C_{30}H_{43}F2O_2P_2Se_2^+$ 695.1037; Found 695.1040

2-((4-methoxybenzyl)selanyl)-5-(trifluoromethyl)benzaldehyde (S21)

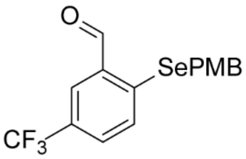

Prepared with general procedure B. The title compound was obtained by purification via silica gel chromatography (0% to 20% EtOAc in hexanes) as a yellow solid (73% yield). R_f = 0.42 (10% EtOAc in hexanes, UV).

^{1}H NMR (400 MHz, $CDCl_3$) δ 10.13 (s, 1H), 8.04 – 8.03 (m, 1H), 7.70 – 7.67 (m, 2H), 7.29 – 7.26 (m, 2H), 6.88 – 6.83 (m, 2H), 4.17 (s, 2H), 3.80 (s, 3H).

^{13}C NMR (101 MHz, $CDCl_3$) δ 191.29 (s), 159.06 (s), 144.10 (d, J = 1.0 Hz), 134.55 (s), 131.02 (q, J = 3.8 Hz), 130.32 (d, J = 5.4 Hz), 129.68 (q, J = 3.5 Hz), 128.52 (s), 128.19 (s), 127.85 (s), 127.73 (s), 127.52 (s), 125.08 (s), 122.38 (s), 119.67 (s), 114.32 (s), 55.36 (s), 30.12 (s).

^{19}F NMR (377 MHz, $CDCl_3$) δ -62.63.

^{77}Se NMR (76 MHz, $CDCl_3$) δ 388.14 (t, J = 9.3 Hz).

HRMS (ESI/Q-TOF) m/z: $[M + H]^+$ calculated for $C_{16}H_{14}F_3O_2Se^+$ 375.0106; Found 375.0105

6,6'-diselanediylbis(3-(trifluoromethyl)benzaldehyde) (S22)

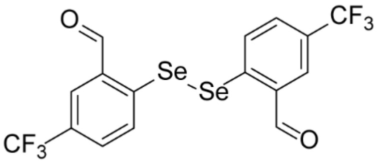

Prepared with general procedure C. The title compound was obtained as a yellow solid (92% yield). R_f = 0.34 (10% EtOAc in hexanes, UV).

^{1}H NMR (400 MHz, $CDCl_3$) δ 10.22 (d, J = 0.5 Hz, 2H), 8.10 (d, J = 1.4 Hz, 2H), 7.95 (d, J = 8.4 Hz, 2H), 7.63 (dd, J = 8.4, 1.6 Hz, 2H).

^{13}C NMR (101 MHz, $CDCl_3$) δ 191.75 (s), 139.46 (s), 135.18 (s), 132.30 (q, J = 3.7 Hz), 132.03 (s), 130.51 (q, J = 3.4 Hz), 129.90 (s), 129.56 (s), 124.96 (s), 122.25 (s).

^{19}F NMR (377 MHz, $CDCl_3$) δ -62.81.

^{77}Se NMR (76 MHz, $CDCl_3$) δ 479.05.

HRMS (ESI/Q-TOF) m/z: $[M + H]^+$ calculated for $C_{16}H_9F_6O_2Se_2^+$ 506.8832; Found 506.8349

(diselanediylbis(5-(trifluoromethyl)-2,1-phenylene))dimethanol (S23)

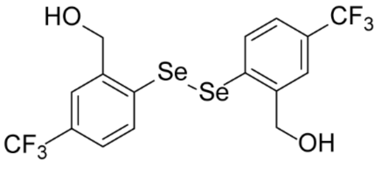

Synthesized follwing general procedure D. The title compound was obtained as a yellow solid (91% yield). R_f = 0.21 (40% EtOAc in Hexanes, UV, $KMnO_4$).

^{1}H NMR (400 MHz, DMSO) δ 7.84 (d, J = 8.1 Hz, 2H), 7.71 (d, J = 1.2 Hz, 2H), 7.58 (dd, J = 8.3, 1.6 Hz, 2H), 5.84 (t, J = 5.4 Hz, 2H), 4.71 (d, J = 5.2 Hz, 4H).

^{13}C NMR (101 MHz, DMSO) δ 142.81 (s), 135.05 (d, *J* = 1.3 Hz), 131.47 (s), 128.53 – 127.17 (m), 125.55 (s), 124.81 (q, *J* = 3.6 Hz), 123.65 (q, *J* = 3.5 Hz), 122.84 (s), 120.14 (s), 62.90 (s).

^{19}F NMR (377 MHz, DMSO) δ -61.05.

^{77}Se NMR (76 MHz, DMSO) δ 402.42.

HRMS (ESI/Q-TOF) m/z: $[M - H]^-$ calculated for $C_{16}H_{11}F_6O_2Se_2^-$ 508.8999; Found 508.8992

1,2-bis(2-(bromomethyl)-4-(trifluoromethyl)phenyl)diselane (S24)

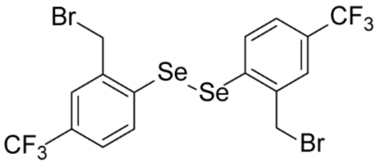

Prepared following general procedure E. The title compound was obtained by purification via silica gel chromatography (0% to 10% EtOAc in hexanes) as a yellow solid (79% yield). R_f = 0.62 (10% EtOAc in hexanes, UV, $KMnO_4$).

^{1}H NMR (400 MHz, $CDCl_3$) δ 7.87 (d, *J* = 8.2 Hz, 2H), 7.60 (s, 2H), 7.47 (d, *J* = 8.2 Hz, 2H), 4.66 (s, 4H).

^{13}C NMR (101 MHz, $CDCl_3$) δ 139.09 (s), 136.75 (s), 135.89 (s), 134.39 (s), 131.06 (q, *J* = 33.1 Hz), 126.90 (q, *J* = 3.7 Hz), 126.50 (q, *J* = 3.6 Hz), 124.95 (s), 122.24 (s), 32.45 (s).

^{19}F NMR (377 MHz, $CDCl_3$) δ -62.76.

^{77}Se NMR (76 MHz, $CDCl_3$) δ 426.75.

HRMS (ESI/Q-TOF) m/z: $[M + H]^+$ calculated for $C_{16}H_{11}Br_2F_6Se_2^+$ 634.7457; Found 634.7458

1,1'-((diselanediylbis(5-(trifluoromethyl)-2,1-phenylene))bis(methylene))bis(2,2,3,4,4-pentamethylphosphetane 1-oxide) (3f)

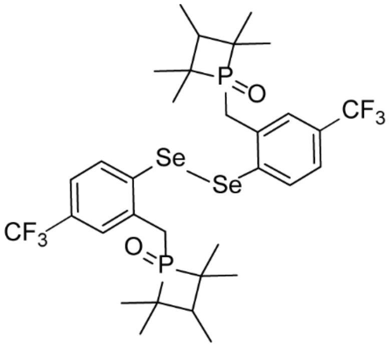

Prepared following general procedure F. The title compound was obtained by purification via silica gel chromatography (5% MeOH in CH_2Cl_2) as a yellow solid (87% yield). R_f = 0.47 (10% MeOH in CH_2Cl_2, UV and $KMnO_4$).

^{1}H NMR (400 MHz, $CDCl_3$) δ 8.20 (s, 2H), 7.62 (d, *J* = 8.4 Hz, 2H), 7.28 (ddd, *J* = 8.4, 1.4, 0.5 Hz, 2H), 3.45 (d, *J* = 10.3 Hz, 4H), 1.71 (qd, *J* = 7.1, 2.0 Hz, 2H), 1.24 (s, 6H), 1.20 (s, 6H), 1.14 (s, 6H), 1.09 (s, 6H), 0.86 (dd, *J* = 7.1, 1.7 Hz, 6H).

^{13}C NMR (101 MHz, $CDCl_3$) δ 133.92 (d, *J* = 6.0 Hz), 133.42 (d, *J* = 0.6 Hz), 130.69 – 129.53 (m), 129.14 (dd, *J* = 7.4, 1.5 Hz), 128.17 (p, *J* = 3.9 Hz), 127.69 (s), 124.98 (d, *J* = 2.1 Hz), 122.27 (s), 119.56 (s), 46.92 (d, *J* = 57.1 Hz), 43.26 (d, *J* = 5.4 Hz), 30.46 (d, *J* = 32.7 Hz), 24.91 (d, *J* = 4.1 Hz), 17.82 (d, *J* = 4.4 Hz), 7.08 (d, *J* = 23.6 Hz).

^{19}F NMR (377 MHz, $CDCl_3$) δ -62.75.

^{31}P NMR (162 MHz, $CDCl_3$) δ 63.13 (minor), 58.51 (major).

^{77}Se NMR (76 MHz, DMSO) δ 458.20.

HRMS (ESI/Q-TOF) m/z: $[M + H]^+$ calculated for $C_{32}H_{43}F_6O_2P_2Se_2^+$ 795.0967; Found 795.0967

2,2'-diselanediylbis(4-fluorobenzaldehyde) (S25)

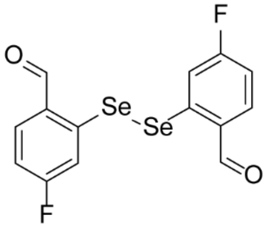

Synthesized following general procedure A. The title compound was obtained by purification via silica gel chromatography (0% to 40% EtOAc in hexanes) as a yellow solid (92% yield). R_f = 0.36 (20% EtOAc in hexanes, UV, $KMnO_4$).

^{1}H NMR (400 MHz, $CDCl_3$) δ 9.97 (s, 2H), 8.05 (dd, *J* = 9.5, 2.2 Hz, 2H), 7.84 (dd, *J* = 8.4, 5.6 Hz, 2H), 7.13 (ddd, *J* = 8.4, 7.6, 2.4 Hz, 2H).

^{13}C NMR (101 MHz, $CDCl_3$) δ 191.34 (s), 167.90 (s), 165.31 (s), 139.73 (d, *J* = 8.4 Hz), 137.80 (d, *J* = 10.2 Hz), 131.20 (d, *J* = 2.4 Hz), 119.39 (d, *J* = 26.3 Hz), 114.34 (d, *J* = 23.0 Hz).

^{19}F NMR (377 MHz, $CDCl_3$) δ -100.70.

^{77}Se NMR (76 MHz, $CDCl_3$) δ 668.24.

HRMS (ESI/Q-TOF) m/z: $[M + H]^+$ calculated for $C_{14}H_9F_2O_2Se_2^+$ 406.8896; Found 406.8892

(diselanediylbis(4-fluoro-2,1-phenylene))dimethanol (S26)

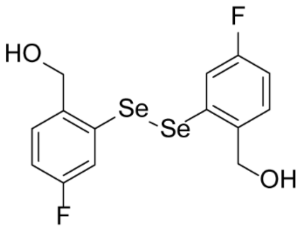

Synthesized follwing general procedure D. The title compound was obtained as a yellow solid (93% yield). R_f = 0.16 (40% EtOAc in Hexanes, UV, $KMnO_4$).

^{1}H NMR (400 MHz, DMSO) δ 7.43 – 7.38 (m, 4H), 7.12 – 7.06 (m, 2H), 5.53 (s, 2H), 4.60 (s, 4H).

^{13}C NMR (101 MHz, DMSO) δ 161.43 (d, *J* = 246.3 Hz), 137.84 (d, *J* = 3.0 Hz), 131.51 (d, *J* = 6.5 Hz), 129.28 (d, *J* = 8.0 Hz), 117.37 (d, *J* = 24.2 Hz), 113.89 (d, *J* = 21.1 Hz), 62.61 (s).

^{19}F NMR (377 MHz, DMSO) δ -114.54.

^{77}Se NMR (76 MHz, DMSO) δ 412.61.

HRMS (ESI/Q-TOF) m/z: $[M - H]^-$ calculated for $C_{14}H_{11}F_2O_2Se_2^-$ 408.9063; Found 408.9056

1,2-bis(2-(bromomethyl)-5-fluorophenyl)diselane (S27)

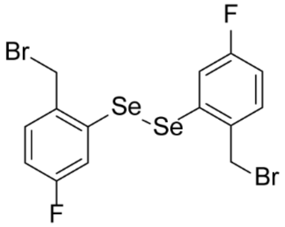

Prepared following general procedure E. The title compound was obtained by purification via silica gel chromatography (0% to 10% EtOAc in hexanes) as a yellow solid (70% yield). R_f = 0.64 (10% EtOAc in hexanes, UV, $KMnO_4$).

^{1}H NMR (400 MHz, $CDCl_3$) δ 7.46 (dd, *J* = 8.5, 2.6 Hz, 2H), 7.35 (dd, *J* = 8.5, 5.5 Hz, 2H), 6.96 (td, *J* = 8.2, 2.6 Hz, 2H), 4.62 (s, 4H).

^{13}C NMR (101 MHz, $CDCl_3$) δ 162.81 (d, *J* = 252.9 Hz), 134.60 (d, *J* = 3.4 Hz), 133.99 (d, *J* = 6.9 Hz), 131.80 (d, *J* = 8.2 Hz), 121.13 (d, *J* = 23.7 Hz), 116.07 (d, *J* = 21.8 Hz), 32.85 (s).

^{19}F NMR (377 MHz, $CDCl_3$) δ -110.50.

^{77}Se NMR (76 MHz, $CDCl_3$) δ 437.28.

HRMS (ESI/Q-TOF) m/z: $[M + H]^+$ calculated for $C_{14}H_{11}Br_2F_2Se_2^+$ 534.7521; Found 534.7549

6-fluoro-2',2',3',4',4'-pentamethyl-3*H*-spiro[benzo[*d*][1,2]selenaphosphole-2,1'-phosphetan]-2-ium bromide (3g-III-Br)

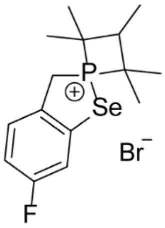

The crude product, **3g-III-Br** (white solid), was synthesized from **S27** following General Procedure F. Analysis by ^{1}H NMR revealed that it was isolated as a 2:1 mixture of diastereomers, accompanied by minor unidentified impurities.

^{1}H NMR (400 MHz, $CDCl_3$) δ 7.74 (dd, *J* = 8.6, 5.3 Hz, 0.66H), 7.62 (dd, *J* = 8.6, 5.3 Hz, 0.33H), 7.44 (dd, *J* = 8.0, 2.3 Hz, 0.33H), 7.37 – 7.32 (m, 0.66H), 7.25 – 7.22 (m, 0.33H), 7.20 – 7.12 (m, 0.66H), 7.07 – 7.01 (m, 0.33H), 7.01 – 6.93 (m, 0.33H), 5.24 (d, *J* = 9.9 Hz, 0.1.32H), 5.03 (d, *J* = 9.9 Hz, 0.66H), 3.53 (dd, *J* = 7.0, 3.5 Hz, 0.66H), 2.90 (p, *J* = 7.1 Hz, 0.33H), 1.75 (s, 2H), 1.74 (s, 1H), 1.69 (s, 2H), 1.68 (s, 1H), 1.62 (s, 1H), 1.56 (s, 3H), 1.50 (s, 2H), 1.15 – 1.07 (m, 3H).

^{19}F NMR (377 MHz, $CDCl_3$) δ -109.81, -110.32.

^{31}P NMR (162 MHz, $CDCl_3$) δ 120.04 (major), 116.19 (minor)

HRMS (ESI/Q-TOF) m/z: $[M - Br]^+$ calculated for $C_{15}H_{21}FPSe^+$ 331.0525; Found 331.0571

1,1'-((diselanediylbis(4-fluoro-2,1-phenylene))bis(methylene))bis(2,2,3,4,4-pentamethylphosphetane 1-oxide) (3g)

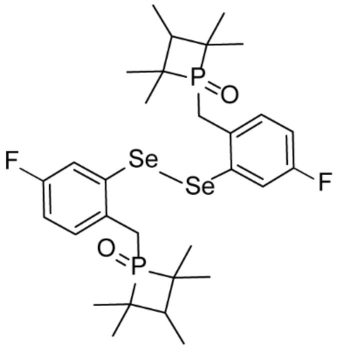

Prepared following general procedure F. The title compound was obtained by purification via silica gel chromatography (5% MeOH in CH_2Cl_2) as a yellow solid (83% yield). R_f = 0.38 (10% MeOH in CH_2Cl_2, UV and $KMnO_4$).

^{1}H NMR (400 MHz, $CDCl_3$) δ 7.61 – 7.56 (m, 2H), 7.52 (dd, *J* = 8.6, 2.7 Hz, 2H), 6.93 (td, *J* = 8.3, 2.7 Hz, 2H), 3.32 (d, *J* = 10.1 Hz, 4H), 1.68 (qd, *J* = 7.0, 2.1 Hz, 2H), 1.25 (s, 6H), 1.21 (s, 6H), 1.14 (s, 6H), 1.10 (s, 6H), 0.89 (dd, *J* = 7.1, 1.6 Hz, 6H).

^{13}C NMR (101 MHz, CDCl3) δ 162.92 (d, *J* = 1.8 Hz), 160.44 (d, *J* = 1.8 Hz), 136.11 (t, *J* = 6.3 Hz), 131.09 (dd, *J* = 7.5, 4.5 Hz), 130.17 (dd, *J* = 7.0, 3.4 Hz), 122.66 (d, *J* = 22.7 Hz), 116.03 (d, *J* = 21.3 Hz), 47.16 (d, *J* = 56.6 Hz), 43.72 (d, *J* = 5.1 Hz), 30.85 (d, *J* = 33.4 Hz), 25.24 (d, *J* = 3.9 Hz), 17.95 (d, *J* = 4.5 Hz), 7.18 (d, *J* = 23.5 Hz).

^{31}P NMR (162 MHz, $CDCl_3$) δ 62.87 (minor), 57.89 (major).

^{19}F NMR (377 MHz, $CDCl_3$) δ -114.40 (d, *J* = 2.9 Hz).

^{77}Se NMR (76 MHz, $CDCl_3$) δ 481.91.

HRMS (ESI/Q-TOF) m/z: $[M + H]^+$ calculated for $C_{30}H_{43}F_2O_2P_2Se_2^+$ 695.1031; Found 695.1040

5-fluoro-2-((2,2,3,4,4-pentamethyl-1-oxidophosphetan-1-yl)methyl)phenyl hypobromoselenoite (3l)

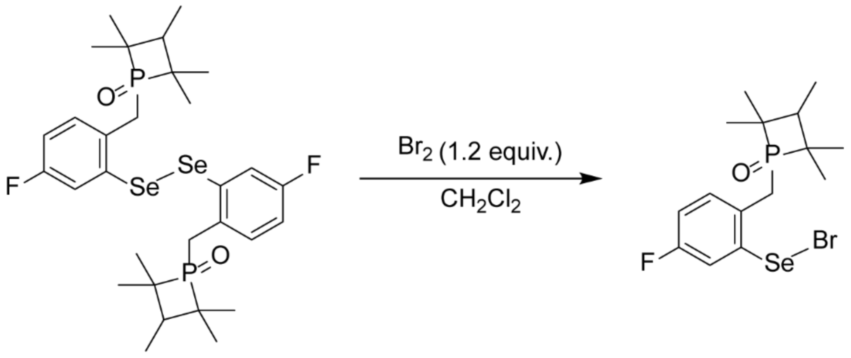

An oven-dried round-bottom flask equipped with a stir-bar was charged with **3g** (200 mg, 0.28 mmol) and CH_2Cl_2 (5 mL). To the solution was then added Br_2 (18 μL, 0.34 mmol). The reaction mixture was stirred for 30 minutes. After completion, the volatiles were removed under reduced pressure to get **3l** as dark brown solid (99% yield)

^{1}H NMR (400 MHz, $CDCl_3$) δ 7.85 (dd, J = 8.6, 2.7 Hz, 1H), 7.21 (dd, J = 7.2, 5.8 Hz, 1H), 6.95 (td, J = 8.2, 2.6 Hz, 1H), 3.54 (d, J = 9.4 Hz, 2H), 1.83 – 1.76 (m, 1H), 1.28 (s, 3H), 1.23 (s, 6H), 1.19 (s, 3H), 0.92 (dd, J = 7.1, 1.6 Hz, 3H).

^{13}C NMR (101 MHz, $CDCl_3$) δ 161.66 (dd, J = 250.3, 2.5 Hz), 139.92 (dd, J = 7.3, 4.6 Hz), 130.74 (dd, J = 7.8, 5.1 Hz), 127.68 (dd, J = 8.7, 3.3 Hz), 124.57 (dd, J = 22.8, 2.1 Hz), 116.53 (dd, J = 21.8, 1.6 Hz), 47.45 (d, J = 55.0 Hz), 43.97 (d, J = 4.7 Hz), 32.01 (d, J = 33.4 Hz), 25.01 (d, J = 3.9 Hz), 17.79 (d, J = 4.9 Hz), 7.31 (d, J = 23.9 Hz).

^{19}F NMR (377 MHz, $CDCl_3$) δ -114.24 (d, J = 3.9 Hz).

^{31}P NMR (162 MHz, $CDCl_3$) δ 63.28 (d, J = 3.5 Hz).

^{77}Se NMR (76 MHz, $CDCl_3$) δ 830.95.

HRMS (ESI/Q-TOF) m/z: $[M + H]^+$ calculated for $C_{15}H_{22}BrFOPSe^+$ 426.9735; Found 426.9730

1-(4-fluoro-2-(methylselanyl)benzyl)-2,2,3,4,4-pentamethylphosphetane 1-oxide (3m)

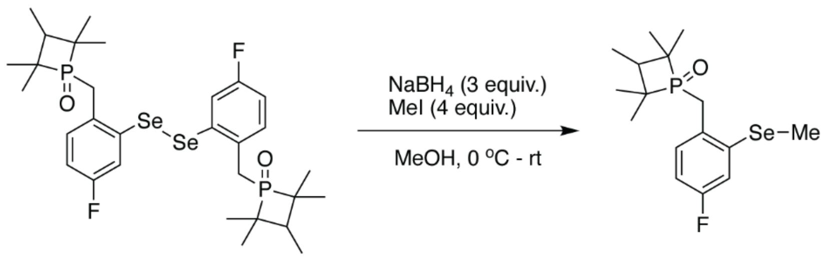

An oven-dried round-bottom flask equipped with a stir-bar was charged with **3g** (300 mg, 0.43 mmol) and MeOH (8 mL). The reaction vessel was cooled to 0 °C. To the solution was then added MeI (81.00 µL, 1.30 mmol) followed by $NaBH_4$ (65.55 mg, 1.73 mmol) under a positive N_2 flow. The reaction mixture was stirred at RT for 1 hour. Progress of the reaction was monitored by TLC. After completion, MeOH was removed under reduced pressure and organic compounds were extracted using DCM. The combined layers were washed with brine and dried over Na_2SO_4, filtered, and concentrated under reduced pressure. The crude product was purified by silica gel column chromatography, eluting with 5% MeOH in CH_2Cl_2, to afford the title compound **3m** (143 mg, 91% yield) as a white solid. R_f = 0.61 (5% MeOH in CH_2Cl_2, UV, $KMnO_4$).

^{1}H NMR (400 MHz, $CDCl_3$) δ 7.86 (ddd, *J* = 8.4, 5.9, 1.5 Hz, 1H), 7.07 (dd, *J* = 9.0, 2.6 Hz, 1H), 6.87 (td, *J* = 8.4, 2.7 Hz, 1H), 3.35 (d, *J* = 10.6 Hz, 2H), 2.30 (s, 3H), 1.73 (qd, *J* = 7.1, 1.9 Hz, 1H), 1.27 (s, 3H), 1.23 (s, 3H), 1.13 (s, 3H), 1.09 (s, 3H), 0.88 (dd, *J* = 7.1, 1.7 Hz, 3H).

^{13}C NMR (101 MHz, $CDCl_3$) δ 163.07 (d, *J* = 1.3 Hz), 160.60 (d, *J* = 1.2 Hz), 135.55 – 135.36 (m), 131.11 (dd, *J* = 7.9, 4.6 Hz), 129.00 (dd, *J* = 6.2, 3.3 Hz), 117.31 (d, *J* = 23.0 Hz), 113.52 (d, *J* = 21.8 Hz), 53.52 (s), 47.45 – 46.19 (m), 43.33 (d, *J* = 5.4 Hz), 29.43 (d, *J* = 35.1 Hz), 24.98 (d, *J* = 4.0 Hz), 18.00 (d, *J* = 4.4 Hz), 8.31 (s), 7.18 (d, *J* = 23.4 Hz).

^{19}F NMR (377 MHz, $CDCl_3$) δ -115.08.

^{31}P NMR (162 MHz, $CDCl_3$) δ 63.66 (d, *J* = 2.5 Hz) (minor), 58.61 (d, *J* = 1.7 Hz) (Major).

^{77}Se NMR (76 MHz, $CDCl_3$) δ 180.42.

HRMS (ESI/Q-TOF) m/z: $[M + H]^+$ calculated for $C_{16}H_{25}FOPSe^+$ 363.0787; Found 363.0795

4-chloro-2-hydroselenobenzaldehyde (S28)

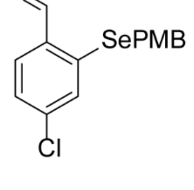

Prepared with general procedure B. The title compound was obtained by purification via silica gel chromatography (0% to 20% EtOAc in hexanes) as a yellow solid (31% yield). R_f = 0.34 (10% EtOAc in hexanes, UV).

^{1}H NMR (500 MHz, CDCl3) δ 9.78 (s, 1H), 7.46 (d, *J* = 8.2 Hz, 1H), 7.05 (d, *J* = 8.2 Hz, 1H), 6.98 (d, *J* = 0.9 Hz, 1H), 6.96 (d, *J* = 8.2 Hz, 2H), 6.55 (d, *J* = 8.4 Hz, 2H), 3.84 (s, 2H), 3.52 (s, 3H).

^{13}C NMR (101 MHz, $CDCl_3$) δ 191.49, 159.07, 140.70, 140.32, 135.03, 133.31, 130.42, 130.37, 128.03, 126.19, 114.32, 55.45, 30.49.

^{77}Se NMR (76 MHz, $CDCl_3$) δ 375.47 (t, *J* = 9.2 Hz).

HRMS (ESI/Q-TOF) m/z: $[M + H]^+$ calculated for $C_{15}H_{13}ClNaO_2Se^+$ 362.9667; Found 362.9664

2,2'-diselanediylbis(4-chlorobenzaldehyde) (S29)

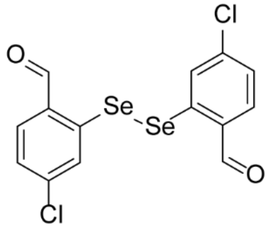

Prepared with general procedure C. The title compound was obtained as a yellow solid (91% yield). R_f = 0.24 (10% EtOAc in hexanes, UV).

^{1}H NMR (500 MHz, $CDCl_3$) δ 10.12 (s, 2H), 7.79 (dd, *J* = 4.6, 3.4 Hz, 4H), 7.40 (dd, *J* = 8.1, 1.8 Hz, 2H).

^{13}C NMR (101 MHz, $CDCl_3$) δ 191.88, 141.99, 136.82, 136.42, 133.33, 130.97, 127.15.

^{77}Se NMR (76 MHz, $CDCl_3$) δ 465.97.

HRMS (ESI/Q-TOF) m/z: $[M + H]^+$ calculated for $C_{14}H_8Cl_2NaO_2Se_2^+$ 460.8129; Found 460.8131

(diselanediylbis(4-chloro-2,1-phenylene))dimethanol (S30)

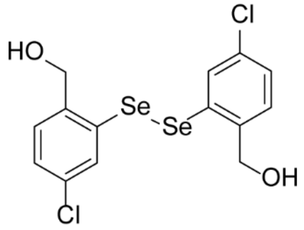

Synthesized follwing general procedure D. The title compound was obtained as a yellow solid (97% yield). R_f = 0.50 (5% MeOH in CH_2Cl_2, UV, $KMnO_4$).

^{1}H NMR (500 MHz, DMSO) δ 7.59 (d, *J* = 1.6 Hz, 2H), 7.39 (d, *J* = 8.1 Hz, 2H), 7.33 (dd, *J* = 8.1, 1.8 Hz, 2H), 5.69 (t, *J* = 5.3 Hz, 2H), 4.59 (d, *J* = 5.3 Hz, 4H).

^{13}C NMR (126 MHz, DMSO) δ 140.87, 132.58, 131.70, 130.37, 129.20, 127.30, 62.82.

^{77}Se NMR (76 MHz, $CDCl_3$) δ 418.25.

HRMS (ESI/Q-TOF) m/z: $[M - H]^-$ calculated for $C_{14}H_{11}Cl_2O_2Se_2^-$ 440.8467; Found 440.8471

1,2-bis(2-(bromomethyl)-5-chlorophenyl)diselane (S31)

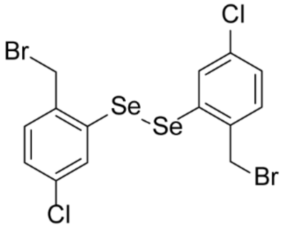

Prepared following general procedure E. The title compound was obtained by purification via silica gel chromatography (0% to 10% EtOAc in hexanes) as a yellow solid (85% yield). R_f = 0.50 (10% EtOAc in hexanes, UV, $KMnO_4$).

^{1}H NMR (400 MHz, $CDCl_3$) δ 7.66 (d, *J* = 2.1 Hz, 2H), 7.29 (d, *J* = 8.2 Hz, 2H), 7.23 (dd, *J* = 8.2, 2.2 Hz, 2H), 4.56 (s, 4H).

^{77}Se NMR (76 MHz, $CDCl_3$) δ 446.29.

^{13}C NMR (101 MHz, $CDCl_3$) δ 137.71, 135.46, 134.88, 133.68, 131.28, 129.42, 32.83.

HRMS (ESI/Q-TOF) m/z: $[M + H]^+$ calculated for $C_{14}H_{11}Br_2Cl_2Se_2^+$ 566.6935; Found 566.6927

1,1'-((diselanediylbis(4-chloro-2,1-phenylene))bis(methylene))bis(2,2,3,4,4-pentamethylphosphetane 1-oxide) (3h)

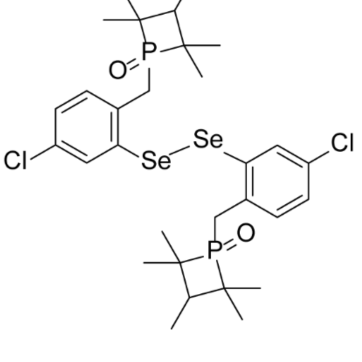

Prepared following general procedure F. The title compound was obtained by purification via silica gel chromatography (100% EtOAc) as a yellow solid (81% yield). R_f = 0.16 (100% EtOAc, UV and $KMnO_4$).

^{1}H NMR (500 MHz, $CDCl_3$) δ 7.79 (d, J = 2.1 Hz, 2H), 7.49 (d, J = 8.3 Hz, 2H), 7.19 (dd, J = 8.3, 2.2 Hz, 2H), 3.29 (d, J = 10.1 Hz, 4H), 1.67 (qd, J = 6.8, 1.4 Hz, 2H), 1.24 (s, 6H), 1.21 (s, 6H), 1.13 (s, 6H), 1.09 (s, 6H), 0.88 (dd, J = 7.0, 1.1 Hz, 6H).

^{13}C NMR (101 MHz, $CDCl_3$) δ 136.52 (d, J = 6.0 Hz), 136.13 (s), 133.36 (d, J = 2.1 Hz), 132.97 (d, J = 7.2 Hz), 130.76 (d, J = 4.4 Hz), 128.98 (s), 47.18 (d, J = 56.5 Hz), 43.42 (d, J = 5.1 Hz), 31.06 (d, J = 32.8 Hz), 25.21 (d, J = 4.1 Hz), 17.96 (d, J = 4.4 Hz), 7.24 (d, J = 23.6 Hz).

^{77}Se NMR (76 MHz, CDCl3) δ 489.40.

^{31}P NMR (202 MHz, $CDCl_3$) δ 63.02 (minor), 58.01 (major).
HRMS (ESI/Q-TOF) m/z: $[M + H]^+$ calculated for $C_{30}H_{43}Cl_2O_2P_2Se_2^+$ 727.0446; Found 727.0451

4-bromo-2-((4-methoxybenzyl)selanyl)benzonitrile (S32)

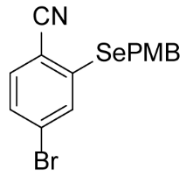

Prepared with general procedure B. The title compound was obtained by purification via silica gel chromatography (0% to 20% EtOAc in hexanes) as a yellow solid (67% yield). R_f = 0.42 (15% EtOAc in hexanes, UV).

^{1}H NMR (400 MHz, $CDCl_3$) δ 7.63 (s, 1H), 7.43 (d, J = 0.7 Hz, 2H), 7.17 (d, J = 8.4 Hz, 2H), 6.80 (d, J = 8.5 Hz, 2H), 4.23 (s, 2H), 3.77 (s, 3H).

^{13}C NMR (101 MHz, $CDCl_3$) δ 159.18, 137.05, 136.83, 134.46, 130.73, 130.30, 128.33, 127.57, 115.90, 114.25, 55.35, 32.43.

^{77}Se NMR (76 MHz, $CDCl_3$) δ 396.17 (t, J = 11.3 Hz).

HRMS (ESI/Q-TOF) m/z: $[M + Na]^+$ calculated for $C_{15}H_{12}BrNNaOSe^+$ 403.9160; Found 403.9172

4-bromo-2-((4-methoxybenzyl)selanyl)benzoic acid (S33)

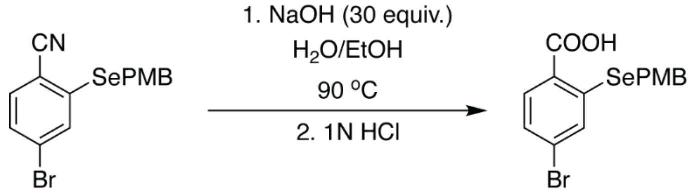

A round-bottom flask equipped with a stir bar and a reflux condenser was charged with **S32** (2.00 g, 5.25 mmol), H_2O (25 mL) and EtOH (25 mL). The reaction vessel was cooled to 0 °C and NaOH (6.30 g, 157.42 mmol) was added in one portion. After stirring for 30 minutes at 0 °C, the reaction vessel was heated to 90 °C and stirred for 12 hours. After completion, the volatiles were removed under vacuum and and neutralized by slow addition of 1N HCl at 0 °C. The organic compounds were then extracted with EtOAc, and the combined organic layers were washed with brine, dried over Na_2SO_4, filtered, and concentrated under reduced pressure to get the titled compound **S33** (2.02 g, 96% yield) as white solid. R_f = 0.57 (5% MeOH in CH_2Cl_2, UV, $KMnO_4$).

^{1}H NMR (400 MHz, $CDCl_3$) δ 7.95 (d, J = 8.4 Hz, 1H), 7.64 (d, J = 1.8 Hz, 1H), 7.37 (dd, J = 8.4, 1.8 Hz, 1H), 7.35 – 7.31 (m, 2H), 6.89 – 6.84 (m, 2H), 4.11 (s, 2H), 3.80 (s, 3H).

^{13}C NMR (101 MHz, $CDCl_3$) δ 170.55, 159.21, 143.07, 133.81, 131.12, 130.59, 129.31, 128.08, 127.89, 125.80, 114.52, 55.49, 30.32.

^{77}Se NMR (76 MHz, $CDCl_3$) δ 392.83.

HRMS (ESI/Q-TOF) m/z: $[M - H]^-$ calculated for $C_{15}H_{12}BrO_3Se^-$ 398.9141; Found 398.9142

2,2'-diselanediyldibenzoic acid (S34)

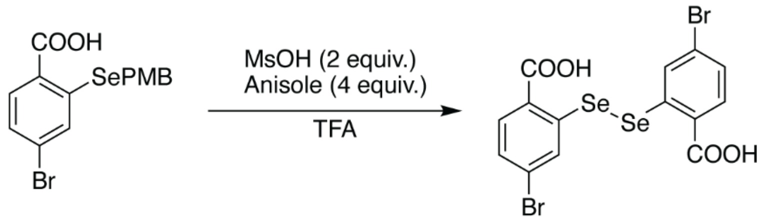

A round-bottom flask equipped with a stir-bar was charged with **S33** (1.00 g, 2.50 mmol), anisole (1.1 mL, 10 mmol) and TFA (20 mL). The flask was cooled to 0 °C and MsOH (325 µL, 5 mmol) was added dropwise. The reaction mixture was stirred overnight at RT. Next day, all the volatiles were removed under reduced pressure and the organic compounds were extracted with EtOAc and the combined organic layers were washed with brine, dried over Na_2SO_4 and filtered. Evaporation of the volatiles followed by precipitation with hexanes afforded the title compound **S34** (681 mg, 98% yield) as a pale yellow solid.

^{1}H NMR (400 MHz, DMSO) δ 7.95 (d, *J* = 8.3 Hz, 2H), 7.76 (d, *J* = 1.9 Hz, 2H), 7.59 (dd, *J* = 8.3, 1.9 Hz, 2H).

^{13}C NMR (101 MHz, DMSO) δ 167.90, 135.37, 133.24, 131.72, 129.79, 128.25, 127.92.

^{77}Se NMR (76 MHz, DMSO) δ 460.31.

HRMS (ESI/Q-TOF) m/z: [M - H]$^-$ calculated for $C_{14}H_7Br_2O_4Se_2^-$ 556.7047; Found 556.7041

1,1'-((diselanediylbis(4-bromo-2,1-phenylene))bis(methylene))bis(2,2,3,4,4-pentamethylphosphetane 1-oxide) (3i)

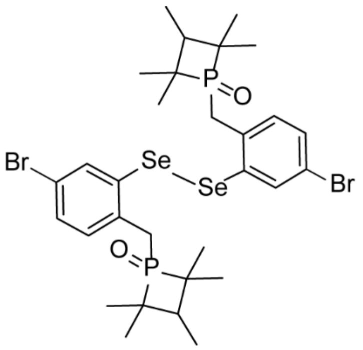

Prepared following general procedure F. The title compound was obtained by purification via silica gel chromatography (5% MeOH in CH_2Cl_2) as a yellow solid (85% yield). R_f = 0.41 (10% MeOH in CH_2Cl_2, UV and $KMnO_4$).

^{1}H NMR (400 MHz, $CDCl_3$) δ 7.93 (d, *J* = 2.0 Hz, 2H), 7.43 (dd, *J* = 8.3, 1.3 Hz, 2H), 7.33 (dd, *J* = 8.2, 2.1 Hz, 2H), 3.29 (d, *J* = 10.1 Hz, 4H), 1.68 (qd, *J* = 7.0, 1.7 Hz, 2H), 1.24 (s, 6H), 1.20 (s, 6H), 1.14 (s, 6H), 1.09 (s, 6H), 0.89 (dd, *J* = 7.1, 1.5 Hz, 6H).

^{13}C NMR (101 MHz, $CDCl_3$) δ 139.04 (s), 137.02 (d, *J* = 5.9 Hz), 133.51 (d, *J* = 7.3 Hz), 131.88 (s), 131.11 (d, *J* = 4.5 Hz), 121.39 (d, *J* = 2.4 Hz), 47.23 (d, *J* = 56.4 Hz), 43.45 (d, *J* = 5.0 Hz), 31.23 (d, *J* = 32.6 Hz), 25.28 (d, *J* = 4.0 Hz), 17.97 (d, *J* = 4.5 Hz), 7.27 (d, *J* = 23.7 Hz).

^{31}P NMR (162 MHz, $CDCl_3$) δ 57.95.

^{77}Se NMR (76 MHz, $CDCl_3$) δ 493.81.
HRMS (ESI/Q-TOF) m/z: [M + H]$^+$ calculated for $C_{30}H_{43}Br_2O_2P_2Se_2^+$ 814.9430; Found 814.9429

2-((4-methoxybenzyl)selanyl)-4-(trifluoromethyl)benzaldehyde (S35)

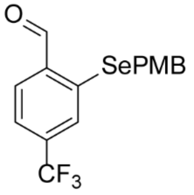

Prepared with general procedure B. The title compound was obtained by purification via silica gel chromatography (0% to 20% EtOAc in hexanes) as a yellow solid (57% yield). R_f = 0.37 (10% EtOAc in hexanes, UV).

^{1}H NMR (500 MHz, $CDCl_3$) δ 10.18 (s, 1H), 7.92 (d, J = 8.0 Hz, 1H), 7.79 (s, 1H), 7.60 (d, J = 7.9 Hz, 1H), 7.20 (d, J = 8.6 Hz, 2H), 6.82 (d, J = 8.6 Hz, 2H), 4.15 (s, 2H), 3.79 (s, 3H).

^{13}C NMR (101 MHz, $CDCl_3$) δ 191.88 (s), 159.13 (s), 139.04 (s), 137.42 (s), 135.13 (s), 134.80 (s), 133.73 (s), 130.40 (s), 128.24 (d, J = 3.9 Hz), 128.06 (s), 122.92 (d, J = 3.6 Hz), 114.34 (s), 55.45 (s), 30.84 (s).

^{19}F NMR (471 MHz, $CDCl_3$) δ -63.21.

^{77}Se NMR (76 MHz, $CDCl_3$) δ 377.47.

HRMS (ESI/Q-TOF) m/z: $[M + Na]^+$ calculated for $C_{16}H_{13}F_3NaO_2Se^+$ 396.9931; Found 396.9937

2,2'-diselanediylbis(4-(trifluoromethyl)benzaldehyde) (S36)

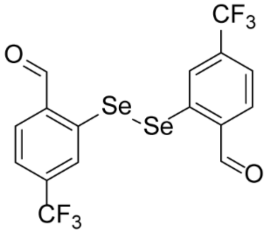

Prepared with general procedure C. The title compound was obtained as a yellow solid (75% yield). R_f = 0.21 (10% EtOAc in hexanes, UV).

^{1}H NMR (400 MHz, $CDCl_3$) δ 10.26 (d, J = 0.6 Hz, 2H), 8.04 (s, 2H), 8.00 (d, J = 7.8 Hz, 2H), 7.67 (dd, J = 7.9, 1.0 Hz, 2H).

^{13}C NMR (101 MHz, $CDCl_3$) δ 192.23 (s), 137.15 (s), 136.12 (s), 135.89 (s), 135.79 (s), 135.51 (s), 135.46 (s), 135.14 (s), 128.35 (dd, J = 7.8, 3.9 Hz), 124.45 (s), 123.71 (dd, J = 7.3, 3.6 Hz), 121.73 (s).

^{19}F NMR (377 MHz, $CDCl_3$) δ -63.43.

^{77}Se NMR (76 MHz, $CDCl_3$) δ 473.53.

HRMS (ESI/Q-TOF) m/z: $[M + Na]^+$ calculated for $C_{16}H_8F_6NaO_2Se_2^+$ 528.8657; Found 528.8661

(diselanediylbis(4-(trifluoromethyl)-2,1-phenylene))dimethanol (S37)

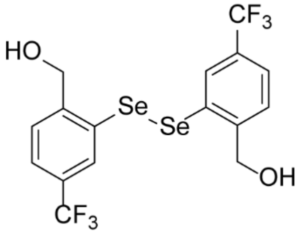

Prepared follwing general procedure D. The title compound was obtained as a yellow solid (98% yield). R_f = 0.46 (5% MeOH in CH_2Cl_2, UV, $KMnO_4$).

^{1}H NMR (400 MHz, DMSO) δ 7.87 (d, J = 1.5 Hz, 2H), 7.67 – 7.58 (m, 4H), 5.80 (t, J = 5.4 Hz, 2H), 4.67 (d, J = 5.2 Hz, 4H).

^{13}C NMR (101 MHz, DMSO) δ 147.02 (s), 129.03 (s), 128.71 (s), 128.40 (s), 128.23 (dd, J = 4.9, 2.9 Hz), 128.01 (s), 125.09 (s), 124.46 (d, J = 3.8 Hz).

^{19}F NMR (377 MHz, DMSO) δ -61.30.

^{77}Se NMR (76 MHz, DMSO) δ 418.90.

HRMS (ESI/Q-TOF) m/z: $[M - H]^-$ calculated for $C_{16}H_{11}F_6O_2Se_2^-$ 508.8994; Found 508.8989

1,2-bis(2-(bromomethyl)-5-(trifluoromethyl)phenyl)diselane (S38)

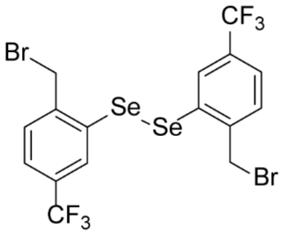

Prepared following general procedure E. The title compound was obtained by purification via silica gel chromatography (0% to 10% EtOAc in hexanes) as a dark brown liquid (75% yield). R_f = 0.51 (10% EtOAc in hexanes, UV, $KMnO_4$).

^{1}H NMR (400 MHz, $CDCl_3$) δ 7.93 (d, *J* = 1.3 Hz, 2H), 7.57 – 7.47 (m, 4H), 4.62 (s, 4H).

^{13}C NMR (101 MHz, $CDCl_3$) δ 143.36 (s), 133.10 (s), 132.47 (d, *J* = 4.1 Hz), 131.81 (d, *J* = 33.0 Hz), 126.21 (d, *J* = 3.8 Hz), 123.29 (d, *J* = 272.9 Hz), 32.35 (s).

^{19}F NMR (377 MHz, $CDCl_3$) δ -62.94.

^{77}Se NMR (76 MHz, $CDCl_3$) δ 450.01.

HRMS (ESI/Q-TOF) m/z: $[M + H]^+$ calculated for $C_{16}H_{11}Br_2F_6Se_2^+$ 634.7462; Found 634.7465

1,1'-((diselanediylbis(4-(trifluoromethyl)-2,1-phenylene))bis(methylene))bis(2,2,3,4,4-pentamethylphosphetane 1-oxide) (3j)

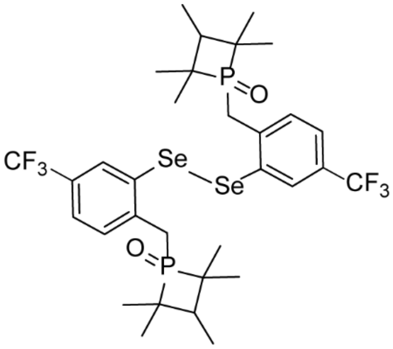

Prepared by following general procedure F. The title compound was obtained by purification via silica gel chromatography (100% EtOAc) as a yellow solid (82% yield). R_f = 0.26 (100% EtOAc, UV and $KMnO_4$).

^{1}H NMR (400 MHz, $CDCl_3$) δ 8.03 (s, 2H), 7.60 (d, *J* = 8.0 Hz, 2H), 7.43 (dd, *J* = 8.1, 1.5 Hz, 2H), 3.42 (d, *J* = 10.1 Hz, 4H), 1.69 (qd, *J* = 7.0, 1.8 Hz, 2H), 1.24 (s, 6H), 1.20 (s, 6H), 1.15 (s, 6H), 1.10 (s, 6H), 0.89 (dd, *J* = 7.1, 1.5 Hz, 6H).

^{13}C NMR (101 MHz, $CDCl_3$) δ 138.47 (d, *J* = 7.7 Hz), 136.43 (d, *J* = 5.6 Hz), 133.29 (s), 130.10 (d, *J* = 4.4 Hz), 125.32 (s), 123.66 (d, *J* = 272.6 Hz), 47.42 (d, *J* = 56.4 Hz), 43.48 (d, *J* = 5.0 Hz), 31.94 (d, *J* = 31.4 Hz), 25.16 (d, *J* = 4.1 Hz), 17.92 (d, *J* = 4.5 Hz), 7.24 (d, *J* = 23.8 Hz).

^{19}F NMR (377 MHz, $CDCl_3$) δ -62.61.

^{31}P NMR (162 MHz, $CDCl_3$) δ 63.11 (minor), 58.35 (major).

^{77}Se NMR (76 MHz, $CDCl_3$) δ 489.72.

HRMS (ESI/Q-TOF) m/z: $[M + H]^+$ calculated for $C_{32}H_{43}F6O2P_2Se_2^+$ 795.0973; Found 795.0972

2,2'-diselanediylbis(4-nitrobenzaldehyde) (S39)

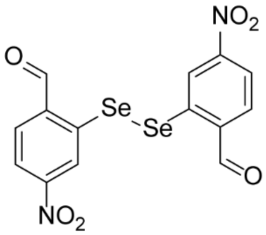

Synthesized following general procedure A. The title compound was obtained by purification via silica gel chromatography (0% to 40% EtOAc in hexanes) as a yellow solid (63% yield). $R_f = 0.26$ (20% EtOAc in hexanes, UV, $KMnO_4$).

^{1}H NMR (400 MHz, $CDCl_3$) δ 10.36 (d, $J = 0.6$ Hz, 2H), 8.46 (d, $J = 2.1$ Hz, 2H), 8.41 (d, $J = 8.3$ Hz, 2H), 8.34 (dd, $J = 8.3$, 2.1 Hz, 2H).

^{13}C NMR (101 MHz, $CDCl_3$) δ 194.36, 150.49, 138.23, 137.74, 133.96, 124.95, 121.98.

^{77}Se NMR (76 MHz, $CDCl_3$) δ 481.68.

HRMS (ESI/Q-TOF) m/z: $[M + H]^+$ calculated for $C_{14}H_9N_2O_6Se_2^+$ 460.8786; Found 460.8781

(diselanediylbis(4-nitro-2,1-phenylene))dimethanol (S40)

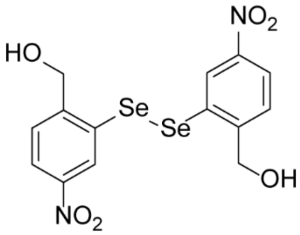

Synthesized follwing general procedure D. The title compound was obtained as a yellow solid (92% yield). $R_f = 0.43$ (5% MeOH in CH_2Cl_2, UV, $KMnO_4$).

^{1}H NMR (400 MHz, DMSO) δ 8.44 (d, $J = 2.3$ Hz, 2H), 8.12 (dd, $J = 8.4$, 2.3 Hz, 2H), 7.66 (d, $J = 8.4$ Hz, 2H), 4.74 (s, 4H).

^{13}C NMR (101 MHz, DMSO) δ 149.48, 147.06, 131.07, 128.23, 126.06, 122.49, 62.96.

^{77}Se NMR (76 MHz, DMSO) δ 425.77.

HRMS (ESI/Q-TOF) m/z: $[M - H]^-$ calculated for $C_{14}H_{11}N_2O_6Se_2^-$ 462.8953; Found 462.8940

1,2-bis(2-(bromomethyl)-5-nitrophenyl)diselane (S41)

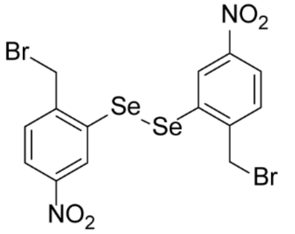

Prepared following general procedure E. The title compound was obtained by purification via silica gel chromatography (0% to 10% EtOAc in hexanes) as a dark brown liquid (70% yield). $R_f = 0.47$ (10% EtOAc in hexanes, UV, $KMnO_4$).

^{1}H NMR (400 MHz, $CDCl_3$) δ 8.57 (d, $J = 2.3$ Hz, 2H), 8.10 (dd, $J = 8.4, 2.3$ Hz, 2H), 7.55 (d, $J = 8.4$ Hz, 2H), 4.69 (s, 4H).

^{13}C NMR (101 MHz, $CDCl_3$) δ 148.29, 145.40, 133.90, 131.01, 129.46, 123.83, 31.53.

^{77}Se NMR (76 MHz, $CDCl_3$) δ 452.17.

HRMS (ESI/Q-TOF) m/z: $[M + H]^+$ calculated for $C_{14}H_{11}Br_2N_2O_4Se_2^+$ 588.7411; Found 588.7420

1,1'-((diselanediylbis(4-nitro-2,1-phenylene))bis(methylene))bis(2,2,3,4,4-pentamethylphosphetane 1-oxide) (3k)

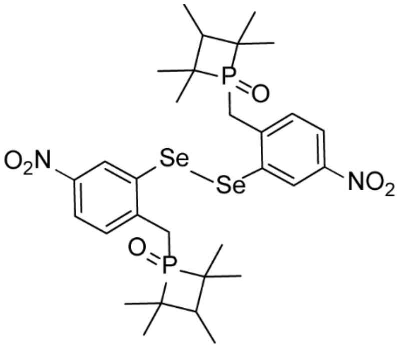

Prepared following general procedure F. The title compound was obtained by purification via silica gel chromatography (100% EtOAc) as a yellow solid (67% yield). $R_f = 0.16$ (100% EtOAc, UV and $KMnO_4$).

^{1}H NMR (400 MHz, $CDCl_3$) δ 8.64 (d, $J = 2.3$ Hz, 2H), 8.01 (dd, $J = 8.4, 2.3$ Hz, 2H), 7.65 (dd, $J = 8.5, 1.4$ Hz, 2H), 3.57 (d, $J = 10.2$ Hz, 4H), 1.75 (qd, $J = 6.9, 1.8$ Hz, 2H), 1.26 (s, 6H), 1.22 (s, 6H), 1.21 (s, 6H), 1.17 (s, 6H).

^{13}C NMR (101 MHz, $CDCl_3$) δ 147.13 (d, $J = 2.1$ Hz), 141.25 (d, $J = 7.5$ Hz), 137.17 (d, $J = 5.3$ Hz), 130.61 (d, $J = 4.4$ Hz), 130.18 (d, $J = 1.3$ Hz), 122.88 (d, $J = 1.3$ Hz), 47.74 (d, $J = 56.0$ Hz),

43.54 (d, J = 5.1 Hz), 32.40 (d, J = 29.8 Hz), 25.23 (d, J = 4.1 Hz), 17.90 (d, J = 4.5 Hz), 7.30 (d, J = 23.9 Hz).

^{31}P NMR (162 MHz, $CDCl_3$) δ 63.71 (minor), 59.09 (major).

^{77}Se NMR (76 MHz, $CDCl_3$) δ 497.64.

HRMS (ESI/Q-TOF) m/z: $[M + H]^+$ calculated for $C_{30}H_{43}N_2O_6P_2Se_2^+$ 749.0921; Found 749.0946

5.2 Solution-Phase Dipeptide Synthesis

All commercially available amino acid hydrochloride (HCl) salts were transformed into their corresponding free amine forms and dried. Stock solutions were prepared in anhydrous CH_3CN in the presence of 4Å molecular sieves and stored overnight before being utilized in dipeptide synthesis.

In a 10 mL vial/round-bottom flask equipped with a stir bar was charged with 5 mL of solution of Boc-Xaa-OH (0.50 mmol) in anhydrous CH_3CN, H-Xaa-O^{t}Bu or H-Xaa-NH_2 (0.55 mmol), and catalyst **3g** (20 mol %). The vial was then placed in a heat block at 80 °C and 1 equiv. of $PhSiH_3$ was added followed by another 1 equiv. after 30 minutes. Progress of the reaction was monitored by TLC. After completion of the reaction, acetonitrile was evaporated under vacuum and 2.0 mL of CH_2Cl_2 was added. The solution was then loaded directly into a chromatographic column for purification.

tert-butyl (tert-butoxycarbonyl)-L-alanyl-L-alaninate (16b)

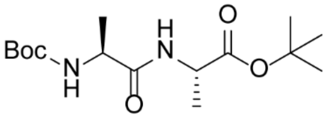

Purified by silica gel chromatography using gradient elution of 10% to 30% EtOAc in hexanes
TLC (UV, Ninhydrin)

White foam (90% yield)

^{1}H NMR (400 MHz, $CDCl_3$) δ 6.70 (d, J = 7.2 Hz, 1H), 5.16 (s, 1H), 4.42 – 4.38 (m, 1H), 4.17 (s, 1H), 1.44 (s, 9H), 1.42 (s, 9H), 1.34 (d, 7.2 Hz, 3H), 1.33 (d, 7.0 Hz, 3H).

^{13}C NMR (101 MHz, $CDCl_3$) δ 172.20, 172.02, 82.02, 77.16, 48.73, 28.40, 28.04, 18.68, 18.57.

HRMS (ESI/Q-TOF) m/z: $[M + H]^+$ calculated for $C_{15}H_{29}N_2O_5^+$ 317.2071; Found 317.2062

***tert*-butyl (*tert*-butoxycarbonyl)-*L*-alanyl-*L*-phenylalaninate (16c)**

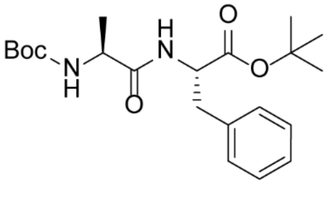

Purified by silica gel chromatography using gradient elution of 10% to 30% EtOAc in hexanes
TLC (UV, Ninhydrin)
White foam (95% yield)

^{1}H NMR (400 MHz, $CDCl_3$) δ 7.28 – 7.20 (m, 3H), 7.15 – 7.13 (m, 2H), 6.58 (d, *J* = 7.6 Hz, 1H), 5.06 (s, 1H), 4.72 – 4.67 (m, 1H), 4.15 (s, 1H), 3.08 – 3.06 (m, 2H), 1.42 (s, 9H), 1.38 (s, 9H), 1.31 (d, *J* = 7.1 Hz, 3H).

^{13}C NMR (101 MHz, $CDCl_3$) δ 172.21, 170.39, 155.38, 136.21, 129.62, 128.42, 127.01, 82.39, 80.03, 53.68, 50.21, 38.12, 28.38, 28.00, 18.56.

HRMS (ESI/Q-TOF) m/z: $[M + Na]^+$ calculated for $C_{21}H_{32}N_2NaO_5^+$ 415.2203; Found 415.2208

***tert*-butyl ((*S*)-1-(((*S*)-1-amino-3-(1*H*-indol-3-yl)-1-oxopropan-2-yl)amino)-1-oxopropan-2-yl)carbamate (16d)**

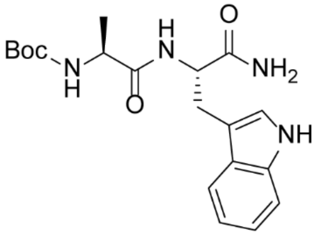

Purified by silica gel chromatography using gradient elution of 50% to 90% EtOAc in hexanes
TLC (UV, Ninhydrin)

White foam (87% yield)

^{1}H NMR (500 MHz, DMSO) δ 10.79 (s, 1H), 7.69 (d, *J* = 8.0 Hz, 1H), 7.58 (d, *J* = 7.9 Hz, 1H), 7.36 – 7.29 (m, 2H), 7.11 (d, *J* = 2.1 Hz, 1H), 7.09 – 7.03 (m, 2H), 7.02 – 6.95 (m, 2H), 4.47 (dd, *J* = 12.7, 6.9 Hz, 1H), 3.92 (dd, *J* = 13.7, 6.7 Hz, 1H), 3.14 (dd, *J* = 14.6, 5.1 Hz, 1H), 3.02 (dd, *J* = 14.6, 7.6 Hz, 1H), 1.36 (s, 9H), 1.13 (d, *J* = 7.2 Hz, 3H).

^{13}C NMR (101 MHz, DMSO) δ 206.57, 173.28, 172.43, 155.26, 136.04, 127.51, 123.49, 120.84, 118.53, 118.24, 111.24, 109.97, 78.30, 54.95, 52.91, 50.26, 39.52, 30.73, 28.20, 27.62, 17.93.

HRMS (ESI/Q-TOF) m/z: $[M + H]^+$ calculated for $C_{19}H_{27}N_4O_4^+$ 375.2027; Found 375.2022

***tert*-butyl (*tert*-butoxycarbonyl)-*L*-alanyl-*L*-valinate (16e)**

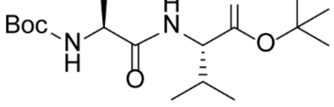

Purified by silica gel chromatography using gradient elution of 10% to 30% EtOAc in hexanes
TLC (UV, Ninhydrin)

Dense liquid (84%)

^{1}H NMR (600 MHz, $CDCl_3$) δ 6.68 (d, *J* = 7.8 Hz, 1H), 5.22 (s, 1H), 4.36 (dd, *J* = 8.8, 4.7 Hz, 1H), 4.17 (s, 1H), 2.13 – 2.07 (m, 1H), 1.41 (s, 9H), 1.39 (s, 9H), 1.30 (d, *J* = 7.2 Hz, 3H), 0.87 (d, *J* = 7.2 Hz, 3H), 0.85 (d, *J* = 6.6 Hz, 3H),

^{13}C NMR (151 MHz, $CDCl_3$) δ 172.61, 170.84, 155.54, 81.85, 79.88, 57.43, 57.26, 50.10, 31.42, 28.35, 28.07, 18.92, 18.07, 17.56.

HRMS (ESI/Q-TOF) m/z: $[M + H]^+$ calculated for $C_{17}H_{33}N_2O_5^+$ 345.2384; Found 345.2399

***tert*-butyl (*tert*-butoxycarbonyl)-*L*-alanyl-*L*-prolinate (16f)**

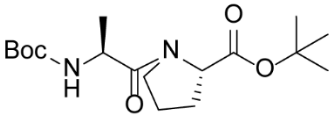

Purified by silica gel chromatography using gradient elution of 10% to 50% EtOAc in hexanes TLC (UV, Ninhydrin)

Dense liquid (88%)

^{1}H NMR (400 MHz, $CDCl_3$) δ 5.36 (d, *J* = 8.0 Hz, 1H), 4.40 – 4.30 (m, 2H), 3.63 – 3.57 (m, 1H), 3.52 – 3.46 (m, 1H), 2.19 – 2.07 (m, 1H), 2.01 – 1.80 (m, 3H), 1.36 (s, 9H), 1.34 (s, 9H), 1.27 (d, *J* = 6.9 Hz, 3H).
^{13}C NMR (101 MHz, $CDCl_3$) δ 171.29, 170.96, 155.17, 81.20, 79.34, 59.82, 59.57, 47.68, 46.73, 46.24, 31.21, 28.97, 28.34, 28.30, 27.92, 27.76, 24.85, 22.06, 19.61, 18.37.

HRMS (ESI/Q-TOF) m/z: $[M + H]^+$ calculated for $C_{17}H_{31}N_2O_5^+$ 343.2227; Found 343.2225

***tert*-butyl (*tert*-butoxycarbonyl)-L-phenylalanyl-L-phenylalaninate (16g)**

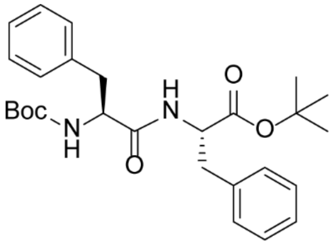

Purified by silica gel chromatography using gradient elution of 10% to 30% EtOAc in hexanes TLC (UV, Ninhydrin)

White foam (89%)

^{1}H NMR (600 MHz, CDCl3) δ 7.28 – 7.26 (m, 2H), 7.25 – 7.22 (m, 3H), 7.20 – 7.18 (m, 3H), 7.07 – 7.06 (m, 2H), 6.41 (br s, 1H), 5.03 (br s, 1H), 4.65 (d, *J* = 4.2 Hz, 1H), 4.36 (br s, 1H), 3.08 – 2.99 (m, 4H), 1.40 (s, 9H), 1.36 (s, 9H).

^{13}C NMR (151 MHz, $CDCl_3$) δ 170.72, 170.07, 155.34, 136.64, 136.10, 129.59, 129.43, 128.68, 128.37, 126.98, 82.37, 80.15, 55.80, 53.79, 38.44, 38.25, 28.33, 27.96.

HRMS (ESI/Q-TOF) m/z: $[M + H]^+$ calculated for $C_{27}H_{37}N_2O_5^+$ 469.2697; Found 469.2696

tert-butyl ((*S*)-1-(((*S*)-1-amino-3-(1*H*-indol-3-yl)-1-oxopropan-2-yl)amino)-1-oxo-3-phenylpropan-2-yl)carbamate (16h)

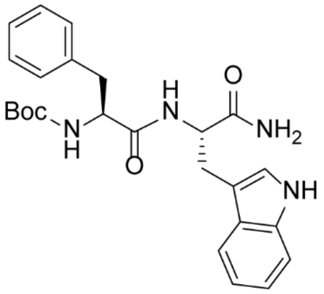

Purified by silica gel chromatography using gradient elution of 0% to 10% MeOH in CH_2Cl_2
TLC (UV, Ninhydrin)

White foam (84% yield)

^{1}H NMR (400 MHz, $CDCl_3$) δ 8.75 (s, 1H), 7.33 – 7.26 (m, 4H), 7.20 – 7.12 (m, 4H), 6.99 – 6.93 (m, 2H), 6.81 (d, *J* = 7.7 Hz, 1H), 6.31 (s, 1H), 5.76 (s, 1H), 4.98 (d, *J* = 5.0 Hz, 1H), 4.76 (dt, *J* = 8.3, 5.8 Hz, 1H), 4.28 (dd, *J* = 12.0, 5.9 Hz, 1H), 3.40 – 3.35 (m, 1H), 3.01 – 2.96 (m, 3H), 1.16 (s, 9H).
^{13}C NMR (101 MHz, $CDCl_3$) δ 174.02, 171.32, 155.83, 136.30, 136.18, 129.43, 128.96, 127.53, 127.46, 123.92, 122.21, 119.76, 118.34, 111.65, 109.39, 80.67, 56.18, 53.60, 37.49, 28.29, 27.99, 27.06.

HRMS (ESI/Q-TOF) m/z: $[M + H]^+$ calculated for $C_{29}H_{38}N_3O_5^+$ 508.2806; Found 508.2808

***tert*-butyl *N*[6]-((benzyloxy)carbonyl)-*N*[2]-((*tert*-butoxycarbonyl)-*L*-phenylalanyl)-*L*-lysinate (16i)**

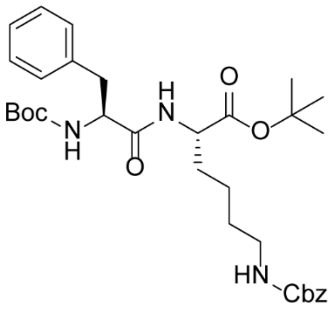

Purified by silica gel chromatography using gradient elution of 0% to 40% EtOAc in hexanes
TLC (UV, Ninhydrin)

Dense clear liquid (85% yield)

^{1}H NMR (400 MHz, $CDCl_3$) δ 7.36 – 7.30 (m, 5H), 7.28 – 7.22 (m, 3H), 7.20 – 7.15 (m, 2H), 6.52 (d, *J* = 7.2 Hz, 1H), 5.15 – 5.00 (m, 3H), 4.44 – 4.33 (m, 2H), 3.16 (dd, *J* = 12.7, 6.4 Hz, 2H), 3.09 – 2.96 (m, 2H), 1.83 – 1.73 (m, 1H), 1.67 – 1.58 (m, 1H), 1.55 – 1.47 (m, 2H), 1.43 (s, 9H), 1.39 (s, 9H), 1.31 – 1.20 (m, 3H).

^{13}C NMR (101 MHz, $CDCl_3$) δ 171.10, 171.03, 156.61, 136.75, 129.46, 129.39, 128.75, 128.65, 128.26, 128.22, 127.07, 82.32, 66.76, 52.51, 40.74, 38.30, 32.29, 29.84, 29.30, 28.38, 28.10, 22.01.

HRMS (ESI/Q-TOF) m/z: $[M + Na]^+$ calculated for $C_{32}H_{45}N_3NaO_7^+$ 606.3150; Found 606.3142

***tert*-butyl (*tert*-butoxycarbonyl)-*L*-tryptophyl-*L*-phenylalaninate (16j)**

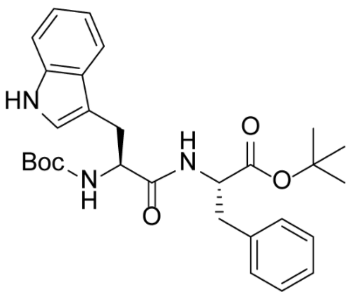

Purified by silica gel chromatography using gradient elution of 0% to 25% EtOAc in hexanes
TLC (UV, Ninhydrin)

White solid (95% yield)

^{1}H NMR (600 MHz, $CDCl_3$) δ 8.58 (s, 1H), 7.68 (d, *J* = 7.9 Hz, 1H), 7.36 (dd, *J* = 8.0, 5.7 Hz, 1H), 7.23 – 7.12 (m, 5H), 7.03 – 6.99 (m, 1H), 6.95 (d, *J* = 6.8 Hz, 2H), 6.47 (d, *J* = 4.3 Hz, 1H),

5.18 (s, 1H), 4.66 (s, 1H), 4.52 (s, 1H), 3.32 (d, *J* = 4.9 Hz, 1H), 3.22 (dd, *J* = 14.4, 6.4 Hz, 1H), 3.03 – 2.94 (m, 2H), 1.45 (s, 9H), 1.37 (s, 9H).

^{13}C NMR (151 MHz, $CDCl_3$) δ 171.27, 170.05, 155.49, 153.51, 136.36, 136.04, 129.50, 129.45, 128.38, 128.29, 127.58, 126.88, 123.44, 122.20, 119.65, 118.83, 111.38, 110.29, 82.28, 80.09, 55.29, 53.77, 38.03, 28.32, 27.92.

HRMS (ESI/Q-TOF) m/z: $[M + H]^+$ calculated for $C_{29}H_{38}N_3O_5^+$ 508.2806; Found 508.2802

tert-butyl (tert-butoxycarbonyl)-L-valyl-L-alaninate (16k)

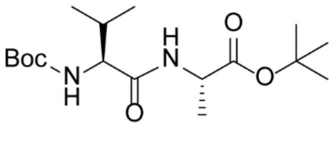

Purified by silica gel chromatography using gradient elution of 10% to 30% EtOAc in hexanes TLC (UV, Ninhydrin)

White foam (87%)

^{1}H NMR (500 MHz, $CDCl_3$) δ 6.59 (d, *J* = 7.0 Hz, 1H), 5.18 (d, *J* = 8.5 Hz, 1H), 4.41 (p, *J* = 7.1 Hz, 1H), 3.95 – 3.93 (m, 1H), 2.08 (dd, *J* = 12.3, 6.0 Hz, 1H), 1.42 (s, 9H), 1.40 (s, 9H), 1.33 (d, *J* = 7.2 Hz, 3H), 0.94 (d, *J* = 6.8 Hz, 3H), 0.89 (d, *J* = 6.9 Hz, 3H).

^{13}C NMR (126 MHz, CDCl3) δ 172.00, 171.12, 155.95, 81.95, 79.78, 77.16, 59.80, 48.72, 31.21, 28.39, 28.01, 19.31, 18.48, 17.80.

HRMS (ESI/Q-TOF) m/z: $[M + H]^+$ calculated for $C_{17}H_{33}N_2O_5^+$ 345.2384; Found 345.2381

tert-butyl (tert-butoxycarbonyl)-L-valyl-L-valinate (16l)

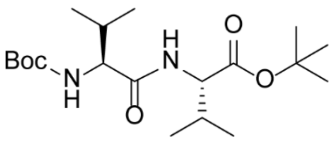

Purified by silica gel chromatography using gradient elution of 10% to 30% EtOAc in hexanes TLC (UV, Ninhydrin)

White foam (81%)

^{1}H NMR (400 MHz, $CDCl_3$) δ 6.41 (d, *J* = 7.9 Hz, 1H), 5.15 (d, *J* = 8.4 Hz, 1H), 4.37 (dd, *J* = 8.7, 4.8 Hz, 1H), 3.91 – 3.89 (m, 1H), 2.13 – 2.03 (m, 2H), 1.41 (s, 9H), 1.39 (s, 9H), 0.93 – 0.85 (m, 12H).

^{13}C NMR (101 MHz, $CDCl_3$) δ 171.61, 170.80, 155.90, 81.92, 79.73, 60.20, 57.57, 31.40, 30.87, 28.36, 28.07, 19.34, 18.93, 18.00, 17.77.

HRMS (ESI/Q-TOF) m/z: $[M + H]^+$ calculated for $C_{19}H_{37}N_2O_5^+$ 373.2697; Found 373.2673

tert-butyl _N_^{6}-((benzyloxy)carbonyl)-_N_^{2}-(_tert_-butoxycarbonyl)-L-lysyl-L-prolinate (16m)

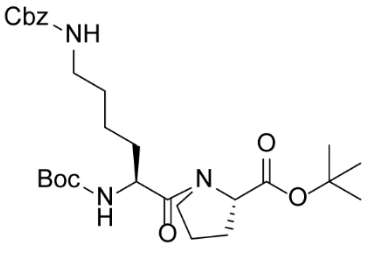

Purified by silica gel chromatography using gradient elution of 10% to 40% EtOAc in hexanes TLC (UV, Ninhydrin)

White solid (83%)

^1H NMR (400 MHz, $CDCl_3$) δ 7.33 – 7.30 (m, 5H), 5.39 – 5.36 (m, 2H), 5.07 (s, 2H), 4.45 – 4.36 (m, 2H), 3.71 – 3.65 (m, 1H), 3.58 – 3.50 (m, 1H), 3.25 – 3.16 (m, 3H), 2.22 – 2.13 (m, 1H), 2.03 – 1.85 (m, 4H), 1.79 – 1.71 (m, 1H), 1.66 – 1.55 (m, 3H), 1.40 (s, 4H).

^{13}C NMR (101 MHz, $CDCl_3$) δ 171.26, 170.83, 156.60, 155.68, 136.86, 128.50, 128.47, 128.21, 128.01, 81.53, 79.58, 66.48, 59.62, 51.51, 47.00, 40.56, 32.28, 29.13, 29.03, 28.40, 27.96, 24.96, 21.74.

HRMS (ESI/Q-TOF) m/z: $[M + H]^+$ calculated for $C_{28}H_{44}N_3O_7^+$ 534.3174; Found 534.3174

tert-butyl (_S_)-2-(((_S_)-6-(((benzyloxy)carbonyl)amino)-1-(_tert_-butoxy)-1-oxohexan-2-yl)carbamoyl)pyrrolidine-1-carboxylate (16n)

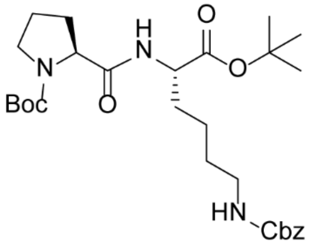

Purified by silica gel chromatography using gradient elution of 10% to 40% EtOAc in hexanes TLC (UV, Ninhydrin)

White solid (92%)

^{1}H NMR (600 MHz, CDCl3) δ 7.40 – 7.27 (m, 5H), 5.08 (s, 3H), 4.51 – 4.16 (m, 2H), 3.56 – 3.27 (m, 2H), 3.17 (s, 2H), 2.29 – 2.02 (m, 1H), 1.99 – 1.77 (m, 4H), 1.68 – 1.50 (m, 3H), 1.45 (s, 18H), 1.34 (S, 3H).

^{13}C NMR (151 MHz, $CDCl_3$) δ 172.59, 171.95, 171.29, 171.15, 156.51, 155.39, 154.63, 136.75, 128.50, 128.36, 128.12, 128.05, 81.89, 80.68, 80.21, 66.53, 61.23, 59.99, 52.54, 52.06, 47.08, 40.74, 32.73, 32.13, 31.06, 29.22, 28.35, 28.03, 24.63, 23.77, 22.16, 21.08.

HRMS (ESI/Q-TOF) m/z: $[M + H]^+$ calculated for $C_{28}H_{44}N_3O_7^+$ 534.3174; Found 534.3152

***tert*-butyl (*S*)-2-(((*S*)-1-(*tert*-butoxy)-1-oxopropan-2-yl)carbamoyl)pyrrolidine-1-carboxylate (16o)**

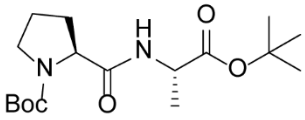

Purified by silica gel chromatography using gradient elution of 10% to 40% EtOAc in hexanes
TLC (UV, Ninhydrin)

Dense clear liquid (92%)

^{1}H NMR (400 MHz, $CDCl_3$) δ 6.81 (d, J = 171.2 Hz, 1H), 4.34 (p, J = 7.0 Hz, 1H), 4.17 (s, 1H), 3.39 (s, 2H), 2.23 – 1.93 (m, 2H), 1.90 – 1.74 (m, 2H), 1.40 (s, 18H), 1.28 (d, J = 7.1 Hz, 3H).

^{13}C NMR (101 MHz, $CDCl_3$) δ 171.92, 154.99, 81.71, 80.34, 60.64, 60.27, 48.67, 47.02, 28.36, 27.99, 24.10, 20.92, 18.61.

HRMS (ESI/Q-TOF) m/z: $[M + H]^+$ calculated for $C_{17}H_{31}N_2O_5^+$ 343.2227; Found 343.2287

***tert*-butyl (2-((*tert*-butoxycarbonyl)amino)-2-methylpropanoyl)-*L*-phenylalaninate (16p)**

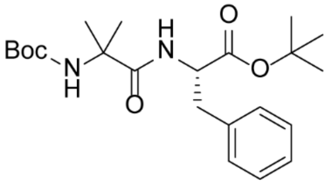

Purified by silica gel chromatography using gradient elution of 10% to 40% EtOAc in hexanes
TLC (UV, Ninhydrin)

White foam (85%)

^{1}H NMR (400 MHz, CDCl3) δ 7.27 – 7.20 (m, 3H), 7.17 – 7.15 (m, 2H), 6.81 (d, *J* = 6.5 Hz, 1H), 4.97 (s, 1H), 4.70 (dt, *J* = 7.4, 6.0 Hz, 1H), 3.09 (dd, *J* = 6.0, 3.4 Hz, 2H), 1.44 (d, *J* = 9.6 Hz, 6H), 1.41 (s, 9H), 1.38 (s, 9H).

^{13}C NMR (101 MHz, $CDCl_3$) δ 174.15, 170.71, 154.56, 136.50, 129.71, 128.35, 126.93, 82.26, 56.75, 53.81, 38.28, 28.39, 28.03, 25.46.

HRMS (ESI/Q-TOF) m/z: $[M + H]^+$ calculated for $C_{22}H_{35}N_2O_5^+$ 407.2540; Found 407.2544

***tert*-butyl *N^α^*-(*tert*-butoxycarbonyl)-*N^τ^*-(((4-methoxybenzyl)oxy)methyl)-*L*-histidyl-*L*-alaninate (16q)**

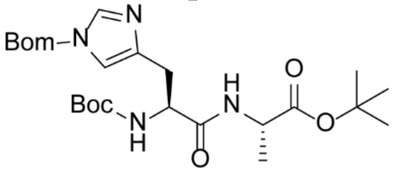

Purified by silica gel chromatography using gradient elution of 0% to 5% MeOH in CH_2Cl_2
TLC (UV, Ninhydrin)

White foam (96%)

^{1}H NMR (600 MHz, $CDCl_3$) δ 7.45 (s, 1H), 7.37 – 7.34 (m, 2H), 7.32 – 7.31 (m, 3H), 6.91 (d, *J* = 5.8 Hz, 1H), 6.88 (s, 1H), 5.54 (d, *J* = 7.3 Hz, 1H), 5.30 (s, 2H), 4.50 (s, 2H), 4.41 (s, 1H), 4.38 – 4.34 (m, 1H), 3.15 – 3.12 (m, 1H), 3.07 – 3.04 (m, 1H), 1.45 (s, 9H), 1.41 (s, 9H), 1.28 (d, *J* = 7.1 Hz, 3H).
^{13}C NMR (151 MHz, $CDCl_3$) δ 171.56, 170.35, 155.43, 138.29, 136.11, 129.28, 128.69, 128.31, 128.11, 127.31, 81.89, 80.11, 73.14, 69.95, 53.84, 48.72, 28.27, 27.93, 26.84, 18.32.

HRMS (ESI/Q-TOF) m/z: $[M + H]^+$ calculated for $C_{18}H_{30}N_4O_5^+$ 382.2211; Found 382.2217

***tert*-butyl (*S*)-4-(((*S*)-1-(*tert*-butoxy)-1-oxopropan-2-yl)amino)-3-((*tert*-butoxycarbonyl)amino)-4-oxobutanoate (16r)**

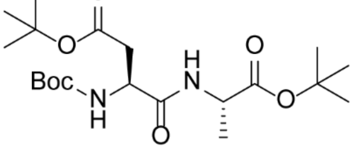

Purified by silica gel chromatography using gradient elution of 30% to 90% EtOAc in hexanes
TLC (UV, Ninhydrin)

Clear dense liquid (86%)

^{1}H NMR (600 MHz, $CDCl_3$) δ 6.26 (d, *J* = 7.1 Hz, 1H), 5.69 (d, *J* = 7.5 Hz, 1H), 4.42 – 4.37 (m, 2H), 2.81 – 2.78 (m, 1H), 2.63 (dd, *J* = 15.4, 3.9 Hz, 1H), 1.43 (s, 9H), 1.42 (s, 9H), 1.40 (s, 9H), 1.32 (d, *J* = 7.1 Hz, 3H).

^{13}C NMR (151 MHz, $CDCl_3$) δ 172.16, 170.44, 169.49, 155.78, 82.15, 82.03, 79.67, 51.04, 48.77, 38.16, 28.41, 28.03, 28.00, 18.71.

HRMS (ESI/Q-TOF) m/z: $[M + H]^+$ calculated for $C_{20}H_{37}N_2O_7^+$ 417.2595; Found 417.2594

tert-butyl _N^2_-(_tert_-butoxycarbonyl)-_N$^\omega$_-((2,2,4,6,7-pentamethyl-2,3-dihydrobenzofuran-5-yl)sulfonyl)-_L_-arginyl-_L_-alaninate (16s)

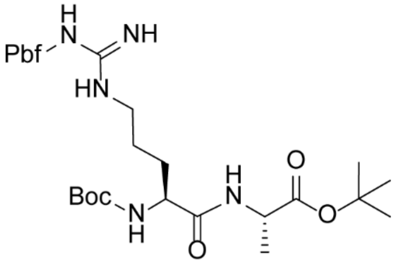

Purified by silica gel chromatography using gradient elution of 50% to 90% EtOAc in hexanes
TLC (UV, Ninhydrin)

Clear dense liquid (72%)

^{1}H NMR (400 MHz, $CDCl_3$) δ 7.33 – 7.24 (m, 1H), 6.43 – 6.05 (m, 3H), 5.59 (s, 1H), 4.35 (dd, *J* = 13.8, 6.8 Hz, 1H), 4.22 (s, 1H), 3.23 (s, 2H), 2.94 (s, 2H), 2.53 (d, *J* = 27.3 Hz, 6H), 2.08 (s, 3H), 1.85 (s, 1H), 1.69 – 1.52 (m, 3H), 1.45 (s, 6H), 1.42 (s, 9H), 1.39 (s, 9H), 1.33 (d, *J* = 6.7 Hz, 3H).

^{13}C NMR (101 MHz, $CDCl_3$) δ 172.34, 158.83, 156.53, 156.05, 138.47, 133.04, 132.37, 124.67, 117.57, 86.45, 82.18, 81.97, 79.99, 53.70, 49.06, 43.36, 40.60, 30.32, 28.71, 28.43, 28.06, 25.35, 19.42, 18.77, 18.06, 17.78, 12.57.

HRMS (ESI/Q-TOF) m/z: $[M + H]^+$ calculated for $C_{31}H_{52}N_5O_8S^+$ 654.3531; Found 654.3532

tert-butyl ((S)-2-((tert-butoxycarbonyl)amino)-2-phenylacetyl)-L-alaninate (16t)

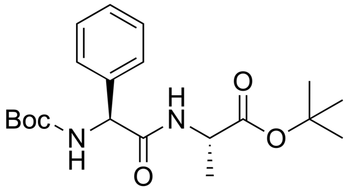

Purified by silica gel chromatography using gradient elution of 10% to 30% EtOAc in hexanes
TLC (UV, Ninhydrin)

White Foam (69%, 3:1 mixture of diastereomers)

^{1}H NMR (400 MHz, $CDCl_3$) δ 7.37 – 7.15 (m, 5H), 6.34 – 6.28 (d, J = 7.0 Hz, 1H), 5.74 (s, 1H), 5.14 (s, 1H), 4.46 – 4.26 (m, 1H), 1.45 – 1.25 (m, 21H).

^{13}C NMR (101 MHz, $CDCl_3$) δ 171.96, 171.58, 169.48, 155.21, 149.10, 148.42, 138.53, 138.29, 129.16, 128.52, 128.07, 127.39, 120.47, 82.28, 80.21, 58.78, 49.21, 49.02, 42.71, 31.06, 28.42, 27.99, 18.70, 18.44.

HRMS (ESI/Q-TOF) m/z: $[M + H]^+$ calculated for $C_{20}H_{31}N_2O_5^+$ 379.2233; Found 379.2229

5.3 Catalytic Solid-Phase Peptide Synthesis

Peptides were synthesized using Tentagel S-RAM resin (0.05 mmol, 208 mg, 0.24 mol/g).

Fmoc deprotection: To a properly washed resin in a SPPS tube was added 2 mL of 20% piperidine in DMF solution. One mililiter of tetrabutylammonium fluoride (1.0 M in THF) was also added during the deprotection step to scavenge any silane polymers formed after the coupling reactions. Afterward, the deprotection solution was drained and the resin was washed with DMF, dry acetonitrile and dry CH_2Cl_2 (3x).

Amino acid coupling: The resin was transferred to a 1-dram glass vial equipped with a stir-bar. Fmoc-Xaa-OH (0.075 mmol) was added to the washed resin along with **3g** (20 mol%, 0.01 mmol). The contents in the vial were dried under reduced pressure for 30 minutes. After that 500 μL of acetonitrile was added. Subsequently, $PhSiH_3$ (0.125 mmol, 2.5 equiv.) was added, and the vial was capped, and reaction mixture was stirred at 80 °C. After one hour of reaction, the resin was transferred to a SPPS tube and the solution was then drained, and the resin was washed with DMF, dry acetonitrile and dry CH_2Cl_2 (3x). Completion of coupling was assessed using Kaiser test or chloranil test (for proline). If necessary, the coupling was repeated to ensure the completion of the reaction.

Global deprotection: After all amino acids were coupled, the resin was treated with a cocktail mixture consisting of TFA/Triisopropylsilane/Water (95:2.5:2.5) for one hour. Subsequently, the resin was filtered. The collected TFA solution was then evaporated, and the resulting residue was precipitated with cold diethyl ether. The precipitate was then triturated with cold diethyl ether 3× to give the crude peptide as white powder. It was then directly analyzed with HPLC and HRMS.

HPLC conditions and Mass analysis:

Fmoc-KCGFG-NH_2: Poroshell 120 EC-C18 4.6 × 100 mm 2.7 μm column; 0.1% TFA (v/v) in water (solvent A)/acetonitrile (solvent B); gradient 5−100% (solvent B) in 10 min; flow rate = 1.5 mL/min; detection wavelength= 220. m/z: $[M + H]^+$ calculated for $C_{37}H_{46}N_7O_7S^+$ 732.3174; Found 732.3171.

Ac-LWFGA-NH_2: Poroshell 120 EC-C18 4.6 × 100 mm 2.7 μm column; 0.1% TFA (v/v) in water (solvent A)/acetonitrile (solvent B); gradient 5−100% (solvent B) in 10 min; flow rate = 1.5 mL/min; detection wavelength= 220. $[M + H]^+$ calculated for $C_{33}H_{44}N_7O_6^+$ 634.3348; Found 634.3396.

Ac-QCFVAYKCGFG-NH_2: Poroshell 120 EC-C18 4.6 × 100 mm 2.7 μm column; 0.1% TFA (v/v) in water (solvent A)/acetonitrile (solvent B); gradient 5−100% (solvent B) in 15 min; flow rate = 1.2 mL/min; detection wavelength= 220. $[M + H]^+$ calculated for $C_{58}H_{83}N_{14}O_{14}S_2^+$ 1263.5649; Found 1263.5772.

6 Analysis of Amino Acid Epimerization

Boc-Val-Val-O^{t}Bu

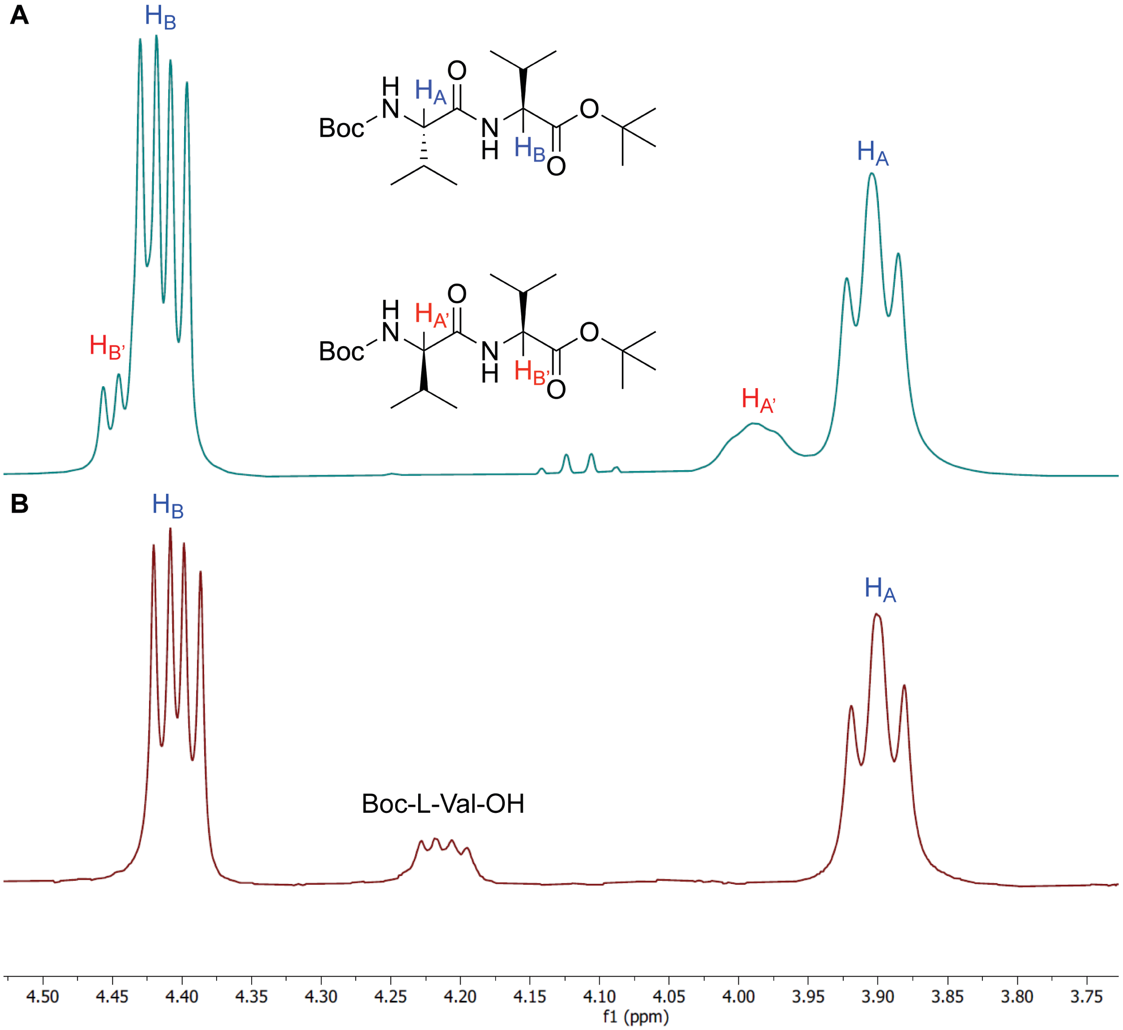

Figure S5. (A) Cropped ^{1}H NMR spectrum of diastereomeric 4:1 LL:DL mixture of Boc-Val-Val-O^{t}Bu standard. (B) Cropped ^{1}H NMR spectrum of crude Boc-Val-Val-O^{t}Bu obtained from the catalytic reaction. Peaks corresponding to Hα-Val protons, BocNHC**H**$(CH_3)_2$ (H_A/$H_{A'}$) and NHC**H**$(CH_3)_2CO_2$ (H_B/$H_{B'}$), are shown.

Boc-Phe-Phe-O*ᵗ*Bu

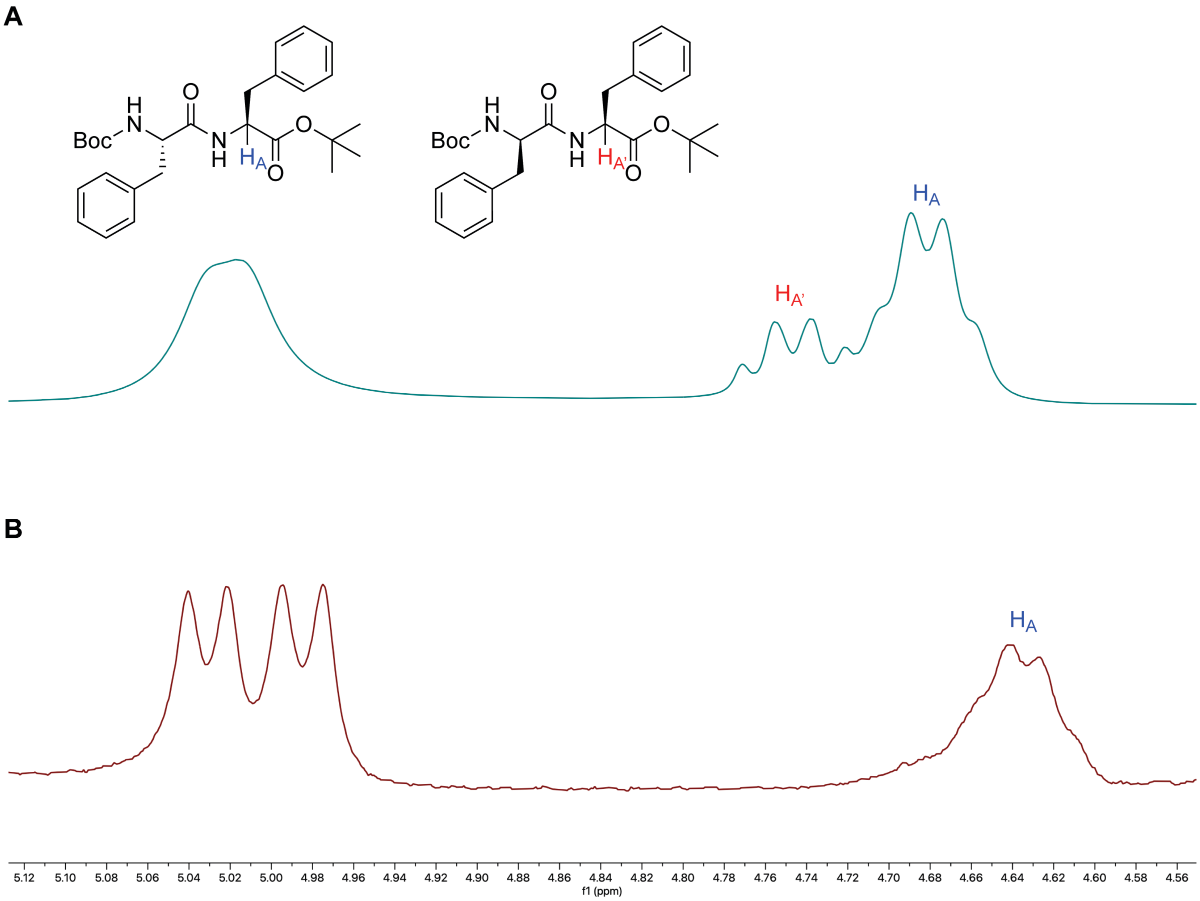

Figure S6. (A) Cropped ^{1}H NMR spectrum of diastereomeric 7:3 LL:DL mixture of Boc-Phe-Phe-O*ᵗ*Bu standard. (B) Cropped ^{1}H NMR spectrum of crude Boc-Phe-Phe-O*ᵗ*Bu obtained from the catalytic reaction. Peaks corresponding to Hα-Phe protons, NHC**<u>H</u>**CO_2 (H_A/$H_{A'}$), are shown.

Boc-Pro-Ala-O^{t}Bu

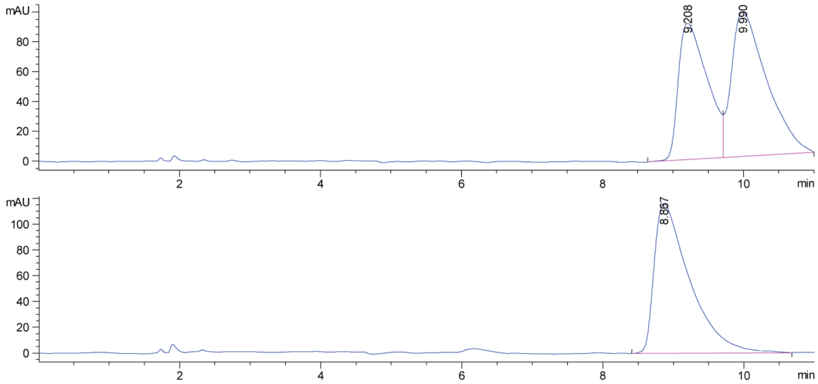

Signal 4: DAD1 D, Sig=210,4 Ref=360,100

Peak #	RetTime [min]	Type	Width [min]	Area [mAU*s]	Height [mAU]	Area %
1	9.208	BV	0.4234	2611.77026	91.38644	43.6024
2	9.990	VBA	0.5029	3378.19580	96.86017	56.3976

Totals : 5989.96606 188.24660

Signal 4: DAD1 D, Sig=210,4 Ref=360,100

Peak #	RetTime [min]	Type	Width [min]	Area [mAU*s]	Height [mAU]	Area %
1	8.867	BB	0.5146	4066.96802	116.12817	100.0000

Totals : 4066.96802 116.12817

Figure S7. (Top) Chromatogram (210 nm) of diastereomeric LL/DL mixture of Boc-Pro-Ala-O^{t}Bu standard. (Bottom) Chromatogram (210 nm) of Boc-Pro-Ala-O^{t}Bu obtained from catalytic reaction. HPLC condition: Chiracel OD; isocratic elution of hexanes:isopropanol = 98:2; flow rate = 1 mL/min

Boc-His(Bom)-Ala-O^{t}Bu

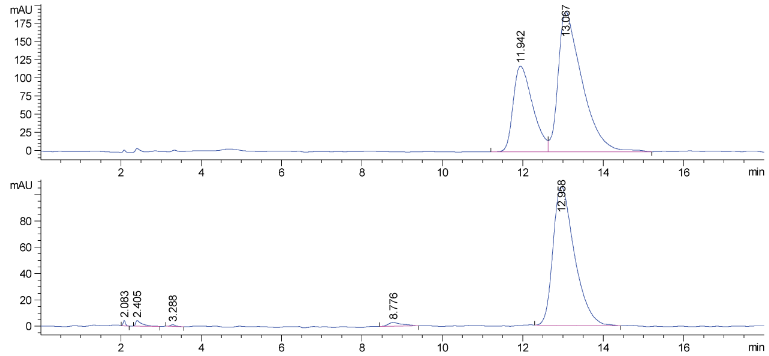

Signal 4: DAD1 D, Sig=210,4 Ref=360,100

Peak #	RetTime [min]	Type	Width [min]	Area [mAU*s]	Height [mAU]	Area %
1	11.942	BV	0.5172	3976.87939	117.98415	33.2919
2	13.067	VB	0.5826	7968.61768	193.42322	66.7081

Signal 4: DAD1 D, Sig=210,4 Ref=360,100

Peak #	RetTime [min]	Type	Width [min]	Area [mAU*s]	Height [mAU]	Area %
1	12.958	BB	0.5577	3915.74585	105.32915	100.0000

Totals : 3915.74585 105.32915

Figure S8. (Top) Chromatogram (210 nm) of diastereomeric LL/DL mixture of Boc-His(Bom)-Ala-O^{t}Bu standard. (Bottom) Chromatogram (210 nm) of Boc-His(Bom)-Ala-O^{t}Bu obtained from catalytic reaction. HPLC condition: Chiracel OD; isocratic elution of hexanes:isopropanol = 97:3; flow rate = 1 mL/min

Boc-Phg-Ala-O^{t}Bu

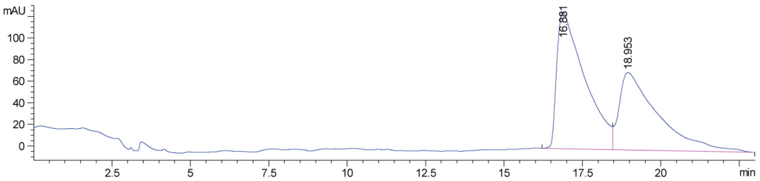

Signal 4: DAD1 D, Sig=210,4 Ref=360,100

Peak #	RetTime [min]	Type	Width [min]	Area [mAU*s]	Height [mAU]	Area %
1	16.881	BV	0.8447	7833.52930	126.44956	55.8942
2	18.953	VBA	1.1450	6181.40381	71.61491	44.1058

Totals : 1.40149e4 198.06447

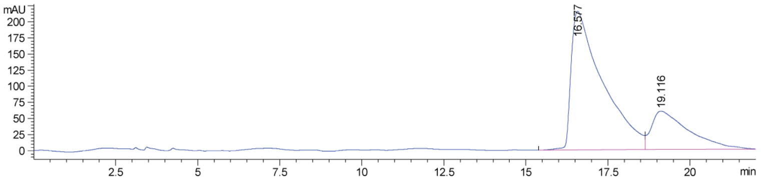

Signal 4: DAD1 D, Sig=210,4 Ref=360,100

Peak #	RetTime [min]	Type	Width [min]	Area [mAU*s]	Height [mAU]	Area %
1	16.577	BV	0.9278	1.44213e4	215.20694	75.2828
2	19.116	VBA	1.0661	4734.84131	59.64796	24.7172

Totals : 1.91561e4 274.85490

Figure S9. (Top) Chromatogram (210 nm) of diastereomeric LL/DL mixture of Boc-Phg-Ala-O^{t}Bu standard. (Bottom) Chromatogram (210 nm) of Boc-Phg-Ala-O^{t}Bu obtained from catalytic reaction. HPLC condition: Chiracel OD; isocratic elution of hexanes:isopropanol = 98:2; flow rate = 0.8 mL/min

7 Computational Methods

7.1 General Computational Information

All calculations were performed using the ORCA 6.0.0/6.0.1 software package[3] on the NYU Greene High-Performance Computing (HPC) cluster. Conformational sampling was carried out using ORCA's GOAT module to identify the lowest-energy conformer for each intermediate. Geometry optimizations were conducted at the r2SCAN-3c level of theory[4], including solvation effects via the SMD model[5,6] for acetonitrile (dielectric constant $\varepsilon = 35.7$). Thermochemical corrections were evaluated at the same level of theory at 80 °C (353.15 K) to reflect experimental conditions. Frequency calculations confirmed that each stationary point was either a minimum (zero imaginary frequencies) or a first-order saddle point (one imaginary frequency) on the potential energy surface.

Electronic single-point energies (SPEs) were computed at the DLPNO-CCSD(T)/def2-TZVPP level of theory[7], employing the corresponding auxiliary basis sets. Final Gibbs Free Energies were obtained by summing the thermochemical and solvation contributions (from the r2SCAN-3c optimizations) with the DLPNO-CCSD(T) electronic energies. Transition states were located using the NEB-TS method[8] as implemented in ORCA 6, and intrinsic reaction coordinate (IRC) calculations were performed to confirm that each transition state connects the expected product and reactant minima. All optimized structures were visualized with CYLview.[9]

7.2 NBO Analysis

Natural Bond Orbital (NBO) analyses were performed to gain deeper insight into the electronic structures of key intermediates and transition states. Using the NBO module[10-11] implemented in ORCA 6, the molecular wavefunctions were recast into localized orbitals that align closely with classical bonding concepts. This approach allowed us to quantify donor–acceptor interactions (via second-order perturbation energies) and to evaluate charge distribution and bond polarization effects. Relevant NBO results, including population analyses and significant donor-acceptor interactions, are presented and discussed below.

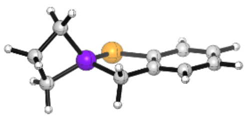

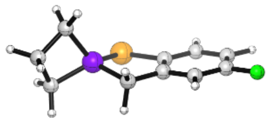

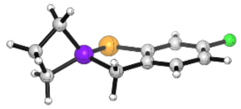

3a-III* **3e-III*** **3g-III***

wB97X-D4/def2-TZVPPD (Grimme et al.)[12] NBO Analysis of (3x)-III* (cation only)

Calculated Property	Unsubstituted (3a)	para-F (3e)	meta-F (3g)
Natural Charge on P	1.31683	1.31393	1.31863
E2 Stabilization Energy (Se LP → Aromatic π*)	16.97	15.74	18.02
Mayer Bond Order (Se-P)	0.8367	0.8244	0.8192

wB97X-3c (Grimme et al.)[12] NBO Analysis of (3x)-III* (cation only)

Calculated Property	Unsubstituted (3a)	para-F (3e)	meta-F (3g)
Natural Charge on P	1.31582	1.31591	1.31736
E2 Stabilization Energy (Se LP → Aromatic π*)	16.98	16.64	17.88
Mayer Bond Order (Se-P)	1.1130	1.1122	1.1048

B3LYP-D4/def2-TZVPPD[13] NBO Analysis of (3x)-III* (cation only)

Calculated Property	Unsubstituted (3a)	para-F (3e)	meta-F (3g)
Natural Charge on P	1.29508	1.29413	1.29900
E2 Stabilization Energy (Se LP → Aromatic π*)	13.25	12.38	13.92
Mayer Bond Order (Se-P)	0.8169	0.8020	0.8009

In the cationic selenophosphonium intermediates (3x-III*), the natural population analyses reveal that the partial positive charge on phosphorus remains nearly the same across all substituents (from +1.29 to +1.32) and only slightly favors the meta-F system. Although this difference is modest, the meta-F derivative consistently shows the highest E2 stabilization energies (Se lone pair → π* of the aromatic ring). Concurrently, its Se–P Mayer bond order is marginally lower than in the unsubstituted and para-F variants. This combination of enhanced donor–acceptor interaction and a more labile Se–P bond suggests that the meta-F substituent better stabilizes the intermediate and facilitates the nucleophilic attack by the carboxylic acid, potentially contributing to the observed higher reactivity.

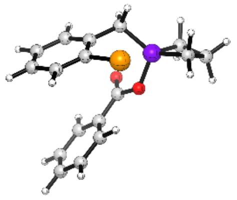

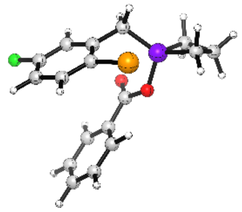

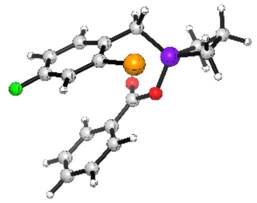

3a-IV **3e-IV** **3g-IV**

wB97X-D4/def2-TZVPPD (Grimme et al.)[12] NBO Analysis of (3x)-IV

Calculated Property	Unsubstituted (3a)	para-F (3e)	meta-F (3g)
**Natural Charge on Se**	-0.58548	-0.57190	-0.59585
**E2 Stabilization Energy (Se LP → Aromatic π*)**	18.97	16.04	26.87
**Mayer Bond Order (Se-P)**	0.4162	0.4307	0.3571

wB97X-3c (Grimme et al.)[12] NBO Analysis of (3x)-IV

Calculated Property	Unsubstituted (3a)	para-F (3e)	meta-F (3g)
**Natural Charge on Se**	-0.57644	-0.56233	-0.58412
**E2 Stabilization Energy (Se LP → Aromatic π*)**	19.06	16.45	26.25
**Mayer Bond Order (Se-P)**	0.2838	0.3111	0.2066

B3LYP-D4/def2-TZVPPD NBO Analysis of (3x)-IV

Calculated Property	Unsubstituted (3a)	para-F (3e)	meta-F (3g)
**Natural Charge on Se**	-0.53665	-0.52211	-0.55057
**E2 Stabilization Energy (Se LP → Aromatic π*)**	14.58	12.82	19.33
**Mayer Bond Order (Se-P)**	0.3927	0.4028	0.3576

In the selenolate–phosphetane-carboxylate intermediates (3x-IV), the partial negative charge on selenium is consistently highest for the meta-F derivative (0.02 to 0.03 lower than others), suggesting a more nucleophilic site for the subsequent attack on the carbonyl carbon. In parallel, the meta-F variant exhibits significantly larger E2 stabilization energies (Se lone pair → π* of the aromatic ring), indicating stronger donor–acceptor interactions. At the same time, its Se–P Mayer bond order is notably lower than those of the unsubstituted and para-F analogues, implying a weaker Se–P interaction. Taken together, these electronic factors support a mechanism wherein the

meta-F substituent enhances the nucleophilicity of selenium and facilitates subsequent bond reorganization steps for formation of the selenoester (3x-V), ultimately contributing to the enhanced reactivity observed experimentally.

Overall, these NBO findings highlight how the meta-F substituent electronically enhances both the cationic selenophosphonium (3x-III*) and the selenolate–phosphetane-carboxylate (3x-IV) intermediates. In the former, the higher E2 stabilization and more labile Se–P interaction facilitates the carboxylic acid attack, while in the latter, the increased negative charge on selenium promotes nucleophilic engagement with the carbonyl. Collectively, these trends provide a coherent explanation for the superior reactivity observed for the meta-F system.

7.3 Cartesian Coordinates for Optimized Structures

The optimized Cartesian coordinates (in xyz format) for each stationary point are provided in Å (angstrom) units at the r2SCAN-3c level of theory.

Unsubstituted

3a-II

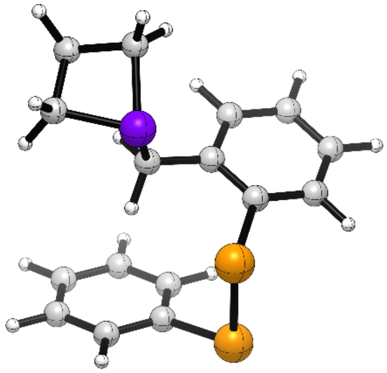

Final Gibbs Free Energy (Eh): -5759.70302603405

# of Imaginary Frequencies: 0

Coordinates:

C	-0.15827153143827	-0.83439530697388	-2.45418788939757
C	-1.00070388300216	0.27807013820911	-2.31656740655469
C	-0.61189454447854	1.54247627467540	-2.74330447940966
C	0.64414549338202	1.72980824813293	-3.31717779987403
C	1.50228586514439	0.64519808686069	-3.45693330513801
C	1.10953013518083	-0.62539352531694	-3.02967591938260
Se	2.33320315614073	-2.10488383840591	-3.28377926008901
P	0.22943261415517	-2.55826240889428	-0.30644881477322
C	-0.61246663975958	-2.16685251029083	-1.94349333713743
C	-0.87836120786977	-1.59011087968566	0.88120983903432
C	-1.90875487442231	-2.74425888519405	0.90833664660298
C	-0.97716942518878	-3.85291045457703	0.36179855470334

H	-1.98482145390207	0.13464362367657	-1.87688070800028
H	-1.29183113770205	2.38260947864360	-2.63002825785058
H	0.95678743458829	2.71464993246751	-3.65281051066313
H	2.48195204110415	0.77944600213692	-3.90691656782462
H	-0.33073772207625	-2.97245775249652	-2.63171276092929
H	-1.70096154451542	-2.17525938082173	-1.82366061404042
H	-0.36109830219627	-1.46865766141173	1.83924812541481
H	-1.23209804391313	-0.61061804580577	0.54469612314057
H	-2.34806533357810	-2.95066035657220	1.89183616512486
H	-2.72823460004470	-2.54789676777831	0.21045489236836
H	-0.49541770076837	-4.42747437087557	1.16039630861961
H	-1.40208115766547	-4.54780318417308	-0.36938325215634
C	-0.00197967489529	-4.54652347709669	-5.15690399028383
C	-1.29451925882204	-5.03757618618282	-4.99859242957583
C	-2.39174836158041	-4.19094907625771	-5.15672543702374
C	-2.19667332831709	-2.85121314237738	-5.48282699531835
C	-0.90594384461585	-2.34992483153326	-5.64257965365938
C	0.18869878651073	-3.19940577714866	-5.47194880878144
Se	1.97159213262783	-2.46817155150527	-5.60690944462830
H	0.85420274779818	-5.20142510329217	-5.02246616270094
H	-1.44463523484043	-6.08463517200089	-4.74950614383538
H	-3.39882448797225	-4.57790907366471	-5.02735600422040
H	-3.04902400255425	-2.18891299623725	-5.60834069303454
H	-0.75061311051345	-1.30256006823237	-5.88556000872575

3a-TS1

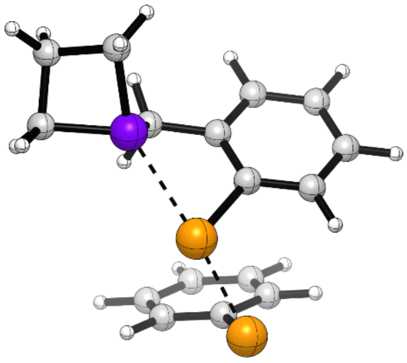

Final Gibbs Free Energy (Eh): -5759.68730780329

# of Imaginary Frequencies: 1

Coordinates:

C	-0.25787111323310	1.07288387180417	0.25841461197314
C	-0.48229601875011	2.25745689985959	-0.44636612136387
C	0.54344193490466	3.17748432477880	-0.64543343598950
C	1.80794969993799	2.92920459153944	-0.11801816557029
C	2.04881911732340	1.75805898317764	0.59595678039878
C	1.02677564454724	0.82921392060346	0.77257665340128
Se	1.35535689469208	-0.84623748545593	1.72225654109994
P	-1.23261202397781	-0.41235569806253	2.29693240477648
C	-1.36453169400669	0.08982775588329	0.51736510937103
C	-2.59735046780539	0.55376129138487	3.15452933995776
C	-3.58597236864114	-0.58330011590856	2.78675423365967
C	-2.55054270794348	-1.71065979768740	2.52181442365260
H	-1.47624889011413	2.45321175800780	-0.84129794852359
H	0.35078505733528	4.09224192594915	-1.19902211689547
H	2.61025886805099	3.65007204630300	-0.25005319036615

H	3.03493039084655	1.56480287728108	1.00689440565777
H	-1.21567574943653	-0.82469492160508	-0.07526806400303
H	-2.34547714641370	0.50721845933874	0.27021338021102
H	-2.40155228329834	0.60954824477619	4.22971848506508
H	-2.80527889582286	1.55309696244172	2.76357619161953
H	-4.31529981662471	-0.81638249864891	3.57028023318268
H	-4.12475468960346	-0.33904768507573	1.86710198384669
H	-2.34551994219715	-2.30726359006667	3.41657265051776
H	-2.73580992800472	-2.37376314187719	1.67311715069956
C	1.66719084291590	-2.44764966670802	-1.64576483209524
C	0.99093253536379	-2.42497102260946	-2.86233264137456
C	1.21164140837823	-1.39105639874614	-3.77167056983262
C	2.11588755681287	-0.37862046941775	-3.45923720514257
C	2.79237016356681	-0.39126138025023	-2.24050014259631
C	2.56922248850519	-1.42684522107024	-1.32827517134159
Se	3.46735632509560	-1.41070871855558	0.37636481810494
H	1.49040374834606	-3.25370784564492	-0.93894826062056
H	0.29035380513980	-3.22103926448470	-3.10098545322984
H	0.68145006181116	-1.37627097671435	-4.71997983867250
H	2.29406245885493	0.43110447624658	-4.16211323276504
H	3.48760473344477	0.40664750921388	-1.99517300681300

3a-III

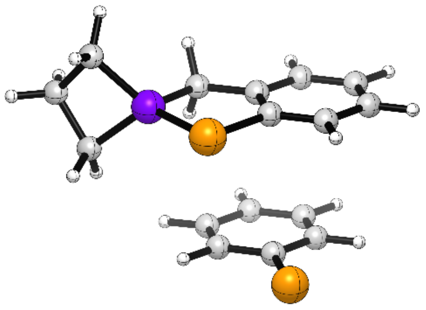

Final Gibbs Free Energy (Eh): -5759.72373880352

# of Imaginary Frequencies: 0

Coordinates:

C	-0.00372183124486	1.45635553838449	0.06626397673071
C	0.07413335440897	2.32529518396042	-1.02341397504404
C	1.24358180624985	3.03337872240808	-1.28049180655283
C	2.34927044745928	2.88325902792436	-0.44670569428058
C	2.29242069160529	2.02523336372748	0.64829793002773
C	1.11698752754274	1.31981338809832	0.88843138181599
Se	1.01933086640113	0.10187343639232	2.39415188287737
P	-1.09073612069855	-0.31415270343314	1.86927102767327
C	-1.26412122472570	0.68057585265053	0.34066011721173
C	-2.49048993111792	-0.15792220410642	3.03948790060699
C	-3.08668851552106	-1.43747275097556	2.37557113626870
C	-1.77209024312798	-2.00720731482529	1.75584563676409
H	-0.79032912764234	2.43660142312220	-1.67301102554002
H	1.29100284021085	3.70248072178226	-2.13470854243292
H	3.26365517452928	3.43569688199691	-0.64323773934314

H	3.15054873218994	1.90743988495277	1.30262668073338
H	-1.49140846064966	-0.01199093091244	-0.48049802584619
H	-2.12842457904961	1.34392327431049	0.46880124610397
H	-2.14528219072417	-0.34367717152280	4.06137142655374
H	-3.06534366380053	0.76890387882847	2.99153245358105
H	-3.56148765089235	-2.11570777225032	3.08751025326645
H	-3.79815731772239	-1.16971963096680	1.59064778273269
H	-1.24164400315202	-2.67136398558144	2.44545082441428
H	-1.82026627151596	-2.44937016132041	0.75942934926784
C	1.25242343632972	-2.07756960410543	-0.76891803855511
C	0.16624810092366	-1.95969431920820	-1.63316168241004
C	0.22684869003093	-1.11617220698278	-2.74160716720659
C	1.39671286126080	-0.39461617240827	-2.97748841427050
C	2.48797612792321	-0.51350772442591	-2.12134885125079
C	2.43898109043062	-1.36034500421840	-1.00094636056280
Se	3.94676925770860	-1.52648122878208	0.17621750918343
H	1.18471379244384	-2.73927068849322	0.09125419015877
H	-0.73619967044251	-2.53386908795722	-1.43648342983154
H	-0.62336912495718	-1.02422725753911	-3.41200019819035
H	1.46231633279550	0.27175963662667	-3.83467398545448
H	3.39159365689115	0.05677746169338	-2.32118954091497

para-F

3e-II

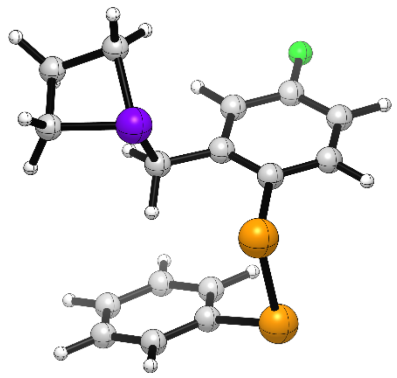

Final Gibbs Free Energy (Eh): -5858.84820627765

# of Imaginary Frequencies: 0

Coordinates:

C	-0.14365704166336	-0.82800742527112	-2.47104547879512
C	-0.99422438193793	0.27476868560641	-2.32319280147726
C	-0.57509684326474	1.52125699860672	-2.74802209793109
C	0.66617446824984	1.75056672401719	-3.31679989017590
C	1.51745746339571	0.66139928719584	-3.45732127924427
C	1.12730234773767	-0.61387759771000	-3.04182129730659
Se	2.35126628046600	-2.08515563904039	-3.30643596992842
P	0.22976008434885	-2.55051105839060	-0.32010240368313
C	-0.59709063967599	-2.16216185805030	-1.96706380425725
C	-0.90397067835220	-1.59993742288225	0.85719906844630
C	-1.92397567743706	-2.76352549719428	0.86340820851625
C	-0.97487238410401	-3.85977246659796	0.32210274476199
H	-1.98342713588535	0.15468607542525	-1.89097803254086
F	-1.42492994524288	2.56925292880170	-2.60139595789623

H	0.95535375254375	2.74652909134328	-3.63677502193711
H	2.49681558085626	0.80441082473748	-3.90418047410078
H	-0.29957418798385	-2.96335349688375	-2.65311008063848
H	-1.68659418294606	-2.17811804550749	-1.86075023716773
H	-0.40048352968650	-1.47985325949464	1.82272372992934
H	-1.26180718332617	-0.62139029544248	0.52238124317486
H	-2.37346135273807	-2.98048109569337	1.83996604133061
H	-2.73645454915135	-2.57040548141885	0.15652193325078
H	-0.49833404663509	-4.43528394645517	1.12314318353573
H	-1.38307898292421	-4.55363307362966	-0.41948390776796
C	-0.00792510109605	-4.54613031229101	-5.12127371366413
C	-1.29774906899368	-5.03504533018617	-4.93665654329873
C	-2.39745318261187	-4.19040398696342	-5.08835383112364
C	-2.20781176997999	-2.85516403349764	-5.43524920736003
C	-0.91980614851775	-2.35592547925594	-5.62104372603281
C	0.17753480647313	-3.20302975065036	-5.45605312216731
Se	1.95737531169472	-2.47162703553442	-5.62222222294115
H	0.85037772292625	-5.19920946247815	-4.99155687176290
H	-1.44383365334343	-6.07878332891105	-4.67173293279414
H	-3.40230953068037	-4.57557376990442	-4.93806044461912
H	-3.06216760850591	-2.19458054468305	-5.55609620836411
H	-0.76849787430625	-1.31173940035941	-5.87964033611888

3e-TS1

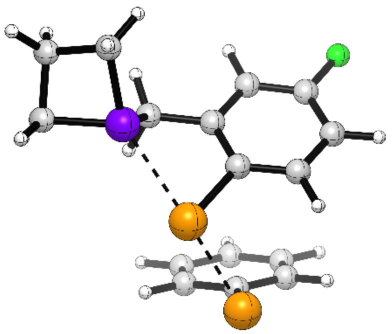

Final Gibbs Free Energy (Eh): -5858.83082771578

# of Imaginary Frequencies: 1

Coordinates:

C	-0.33846046165054	0.96648780446453	0.23777143382198
C	-0.58680709117110	2.11935299012098	-0.50833601539059
C	0.45267447192355	3.00153427648856	-0.74460929820816
C	1.73099161948279	2.80673811085352	-0.25414224650999
C	1.97408686534781	1.66213377174393	0.50021237392177
C	0.95705792741331	0.74116974673546	0.73585452284369
Se	1.30500336176197	-0.89635296500965	1.73933827124254
P	-1.26708543042624	-0.43929247929349	2.34848967909519
C	-1.44242802567239	-0.00127055090240	0.55641549048270
C	-2.58610116398639	0.58087630878819	3.21196203796554
C	-3.60341347871826	-0.55506021727788	2.92766157688761
C	-2.59534585638576	-1.70713199219057	2.66257319699592
H	-1.57882337528074	2.32507403223699	-0.89977197072325
F	0.19681986746559	4.11661602556809	-1.47825639144063
H	2.51415133698963	3.53354202370982	-0.44575945552921

H	2.96979677895425	1.48606822884885	0.89464060889818
H	-1.30655811822421	-0.93625125854829	-0.00677755725672
H	-2.42742353668630	0.41008794048527	0.31620183248878
H	-2.35041302747306	0.67870116689955	4.27609797089512
H	-2.79444999645638	1.56620879097885	2.78724799051566
H	-4.30116213074864	-0.74875891357117	3.74954821695503
H	-4.17765156623005	-0.33734943066836	2.02300992300217
H	-2.36608420366541	-2.27873813574595	3.56769276681768
H	-2.82215308935823	-2.39399762247964	1.84335284234577
C	1.57121753908183	-2.25667927431126	-1.67276025104302
C	0.96521923225273	-2.14912193425301	-2.92128749731157
C	1.39719579757904	-1.18762589743610	-3.83462165435309
C	2.44023325992437	-0.33084983331967	-3.49162997601924
C	3.04354780024909	-0.42372324823767	-2.23851787771806
C	2.61137987822778	-1.38904690681638	-1.32433827829105
Se	3.43125519468658	-1.47888548519991	0.41658577279963
H	1.23219680939559	-3.00880482551847	-0.96577000523519
H	0.15456953662341	-2.82443928550747	-3.18267672375595
H	0.92236621449713	-1.10802832471705	-4.80864428777759
H	2.78260539453049	0.42299974117033	-4.19579621005389
H	3.84399854901528	0.25922081113233	-1.96770965134680

3e-III

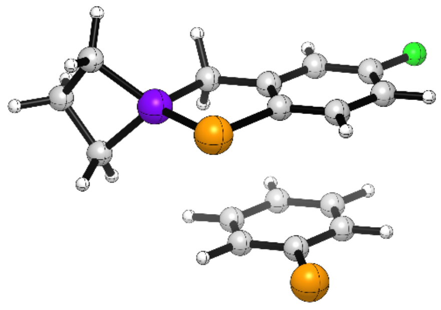

Final Gibbs Free Energy (Eh): -5858.86745103011

# of Imaginary Frequencies: 0

Coordinates:

C	-0.00590945609003	1.45066697056161	0.08394073600356
C	0.07364065470008	2.28027619153411	-1.03448636309278
C	1.25653854180017	2.95443217456689	-1.27538214269879
C	2.36702564193056	2.85295966859283	-0.45539051461244
C	2.28819992332044	2.02670668817475	0.66157314484919
C	1.10917323388618	1.33222679529018	0.91683344891903
Se	1.00014395159410	0.14978008483988	2.44786355058116
P	-1.09407867712318	-0.31023193279059	1.89554486790575
C	-1.27660438076323	0.70133038725126	0.37821376414976
C	-2.51032067813605	-0.19403641746073	3.04889737377751
C	-3.07472160334339	-1.47517400596798	2.36019637511601
C	-1.74142050145323	-2.01448487152537	1.75358606145983
H	-0.77047852118798	2.39025840347134	-1.70887374851592
F	1.32782424753724	3.75052558668059	-2.37242106735996
H	3.27365345958230	3.40397467337271	-0.68397653570477

H	3.14383240295069	1.92682660983566	1.32151677473776
H	-1.54256672850158	0.02748121515618	-0.44620193094672
H	-2.11778216792719	1.38810894172533	0.53547195098901
H	-2.17501559275912	-0.38791334161767	4.07256542177870
H	-3.10046752768093	0.72335274056748	3.00592427227501
H	-3.54766416766497	-2.17028822928375	3.05688071284160
H	-3.77916368854945	-1.20966209939608	1.56822418916241
H	-1.20858959976488	-2.67558967637227	2.44432444532504
H	-1.76727459009822	-2.44665468286928	0.75209096340712
C	1.22766934839186	-2.02756529609141	-0.77223194513762
C	0.15161034553849	-1.90877547378147	-1.64910247176077
C	0.23189406919022	-1.07839358437594	-2.76611291138368
C	1.41133122153770	-0.37119611348021	-2.99825427116049
C	2.49244040802392	-0.49118101375948	-2.12958687398739
C	2.42347651537380	-1.32455154013749	-1.00006130842264
Se	3.91832388016014	-1.49330851912852	0.19291261247408
H	1.14562997168650	-2.67984029214248	0.09392290072769
H	-0.75855528990437	-2.47172712016236	-1.45573394038728
H	-0.61052795663462	-0.98575361914886	-3.44619279120343
H	1.49243251772874	0.28438503468500	-3.86252519041683
H	3.40400079264929	0.06723566318617	-2.32684955968873

meta-F

3g-II

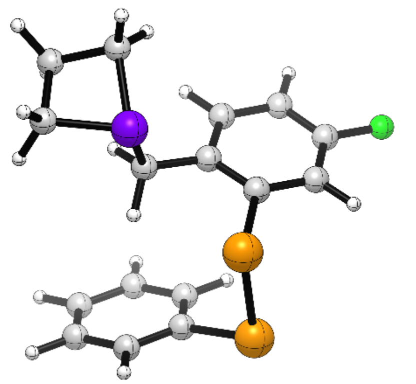

Final Gibbs Free Energy (Eh): -5858.84729807596

# of Imaginary Frequencies: 0

Coordinates:

C	-0.16697766019477	-0.84896017847726	-2.44934727849482
C	-1.01332753410766	0.26146160609053	-2.32245012252978
C	-0.63886848447688	1.52762101913129	-2.75566333233086
C	0.61669230936257	1.67629576155428	-3.31912276201041
C	1.49902299943936	0.62312583917579	-3.46215950773680
C	1.10162552046882	-0.64158074080619	-3.02295741355461
Se	2.32915876735326	-2.11958445401406	-3.26609466250610
P	0.23162474823043	-2.55918733236613	-0.29377654710592
C	-0.61754227728218	-2.17932938697636	-1.92914514263288
C	-0.87012514455219	-1.58050408374939	0.89057275265434
C	-1.89972778708903	-2.73503389402747	0.93343706722834
C	-0.97076287649036	-3.84833195475082	0.39176901404055
H	-1.99828740387695	0.12210253976273	-1.88477077125126
H	-1.30470646876380	2.38005694674420	-2.66233945199577

F	1.00132673311387	2.90792981227914	-3.74650739577376
H	2.47322161722490	0.78387095147786	-3.91326549073619
H	-0.33952420751122	-2.98799505660487	-2.61535550782505
H	-1.70553583920217	-2.18761494933540	-1.80553860414172
H	-0.34845043824057	-1.44952547708390	1.84491283548581
H	-1.22619247992457	-0.60450587735031	0.54636736786755
H	-2.33286663835136	-2.93288873693695	1.92139765482182
H	-2.72336821613222	-2.54533069534009	0.23864444731395
H	-0.48505251782538	-4.41621284106190	1.19270410436544
H	-1.39925164766146	-4.54931664548509	-0.33144692726986
C	-0.00041478145331	-4.53928158960542	-5.17912370700010
C	-1.29682576041297	-5.02423127077718	-5.03431755900966
C	-2.38787426452512	-4.16885669903099	-5.18787672287188
C	-2.18295365263594	-2.82634071942460	-5.49612876474348
C	-0.88838222164933	-2.33112433124112	-5.64274441759243
C	0.19992846258836	-3.18957956625342	-5.47646809470160
Se	1.98753614295802	-2.46744293399160	-5.59357094543434
H	0.85104462210856	-5.20098550342767	-5.04815485202333
H	-1.45481103438901	-6.07335024906946	-4.79922390760195
H	-3.39801497261131	-4.55113244977334	-5.06892052458788
H	-3.03057534426254	-2.15736935521386	-5.61819322159935
H	-0.72514970381408	-1.28191743650840	-5.87223109805275

3g-TS1

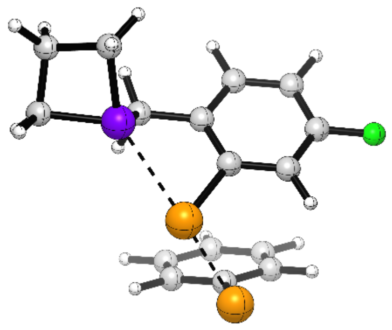

Final Gibbs Free Energy (Eh): -5858.83486061856

# of Imaginary Frequencies: 1

Coordinates:

C	-0.34377843733755	0.93713321275391	0.27780498103333
C	-0.60879195587669	2.11016488072250	-0.43194646111494
C	0.39177721451956	3.03712266190314	-0.70969139203228
C	1.66410252395369	2.76888809667448	-0.23890652430865
C	1.97983688348490	1.63164863226349	0.48185024205688
C	0.96789597420646	0.70823049096298	0.73138519783380
Se	1.36366876532103	-0.94342987299605	1.69135206692075
P	-1.28195272443521	-0.46912809381812	2.39664915582962
C	-1.43603365672770	-0.04359075321270	0.59729991019796
C	-2.57392319691044	0.61589453443950	3.22485363728667
C	-3.63849437908886	-0.47661780698691	2.94561611807036
C	-2.67880332320218	-1.67251245409444	2.70304792075162
H	-1.62100136281566	2.29752351756779	-0.78041267972850
H	0.18905249354510	3.94564400030283	-1.26778436282520
F	2.65482606114975	3.66877055021070	-0.48157120509468

H	2.99627571427306	1.47029065852832	0.82624656348910
H	-1.28832423668210	-0.98230986625109	0.04328129900802
H	-2.42243201019603	0.35670028073922	0.34311106230985
H	-2.34874635694701	0.72140647246583	4.29071413170359
H	-2.73669083841675	1.60356840086440	2.78523585188539
H	-4.35259135432191	-0.62942311074435	3.76241839103089
H	-4.19591364686353	-0.24511512036771	2.03378041796810
H	-2.48798263222407	-2.24860441671814	3.61424305716867
H	-2.92553836926735	-2.35522630588144	1.88577660693363
C	1.63484104384633	-2.29068756870226	-1.73382727414296
C	1.04143751651474	-2.19140496206003	-2.98887306575048
C	1.45542201905449	-1.20930158972609	-3.88870117256303
C	2.46640381721349	-0.32282196751332	-3.52650886815811
C	3.05711714995808	-0.40710198214285	-2.26666998617983
C	2.64301506057313	-1.39398991486673	-1.36802474251271
Se	3.45203435182022	-1.47407516906801	0.37952041266604
H	1.31117060713141	-3.05821738754832	-1.03631846768111
H	0.25560161647016	-2.88914059039680	-3.26629694948515
H	0.99041628059462	-1.13652516063992	-4.86797614164896
H	2.79323651925869	0.44708509260252	-4.22051945208155
H	3.83345870695978	0.29696834105198	-1.98029091403903

3g-III

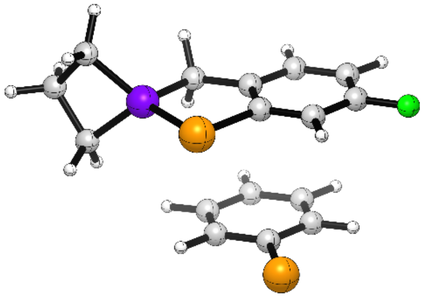

Final Gibbs Free Energy (Eh): -5858.86756630442

# of Imaginary Frequencies: 0

Coordinates:

C	-0.00745197606401	1.45064008030812	0.07097563860175
C	0.07499229861352	2.31766090449120	-1.01943469094015
C	1.24112187589781	3.02571687785158	-1.28737880934204
C	2.32034457599715	2.84894354400351	-0.43853187525254
C	2.29285586720519	2.00816596358822	0.66032548391194
C	1.11199979941124	1.31021318423421	0.89495119558183
Se	1.01635221925357	0.09396466287932	2.39710605177015
P	-1.09652777730145	-0.32010134765738	1.87000332329961
C	-1.26904657812312	0.67642443802664	0.34309898730401
C	-2.49530574737212	-0.16698307423248	3.04085404262264
C	-3.09010501228363	-1.44582510574646	2.37380728706100
C	-1.77448358780099	-2.01349202313855	1.75378284675508
H	-0.78604403614271	2.43291526444579	-1.67206893530577
H	1.31457582230038	3.69705904156555	-2.13676528842760
F	3.46502782782336	3.53108679187944	-0.69047048210268

H	3.16396655001057	1.90422126170748	1.29854808859798
H	-1.49678313842171	-0.01624566846271	-0.47785308702441
H	-2.13319098460875	1.33958436382515	0.47320902073194
H	-2.14983694568908	-0.35480500468136	4.06227506530673
H	-3.07059865494709	0.75964087557850	2.99471257055321
H	-3.56434127125970	-2.12580135690733	3.08441010972078
H	-3.80137902276189	-1.17719350953057	1.58904419867171
H	-1.24371602373549	-2.67790299869937	2.44289464636086
H	-1.82118066406531	-2.45393738854114	0.75652475973560
C	1.26490997612572	-2.06159296024614	-0.77362053191392
C	0.17784199585799	-1.94942218400456	-1.63739941215327
C	0.23246167261454	-1.10270498202223	-2.74381065813098
C	1.39710279802532	-0.37224664008839	-2.97793707680998
C	2.48910437010401	-0.48492901425016	-2.12188252805517
C	2.44608667039591	-1.33477191253997	-1.00353554267597
Se	3.95447277036972	-1.49283724487501	0.17378467508048
H	1.20204711522080	-2.72586949849193	0.08493200525775
H	-0.72045720969275	-2.53054261787695	-1.44207275047376
H	-0.61832331051317	-1.01522512192929	-3.41408846431893
H	1.45786391590149	0.29656721267232	-3.83357844338501
H	3.38884381965466	0.09202518686496	-2.32061142061286

Unsubstituted

3a-IV

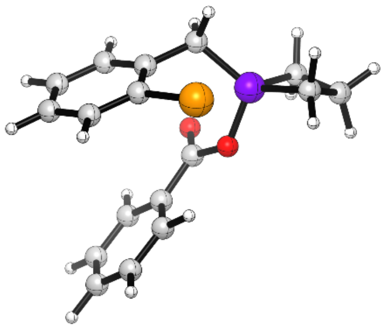

Final Gibbs Free Energy (Eh): -3547.86726067410

# of Imaginary Frequencies: 0

Coordinates:

Se	0.26722836046515	3.09952698029922	0.23405546223705
C	-0.75778979714143	1.80089931978296	1.18485761275770
C	-1.70191115993598	1.01208129072609	0.49588762165473
C	-2.42410536057828	0.02975255660245	1.18040755796681
C	-2.23233442638615	-0.18082399474404	2.54124877704727
C	-1.29978182791104	0.59817552452963	3.22797399421808
C	-0.57371444779427	1.57608702771255	2.55848771361706
C	-1.93096325153865	1.20401292806774	-0.97586168323495
H	-3.13905548984978	-0.57927549018146	0.63251940420686
H	-2.79997709851708	-0.94837579789550	3.05952684409837
H	-1.13370525205042	0.44103905461452	4.29094194022862
H	0.15558328868579	2.17273182940684	3.10009335734087
H	-2.25767214000069	2.22550636466833	-1.21790191863888
H	-2.68006817438905	0.50016889251895	-1.35898946962388

C	0.44267546503863	2.10578753342407	-3.09108472003094
C	0.36297588800784	1.06017034712914	-4.24543134399788
C	-0.74532212171214	0.15493733523744	-3.63368577226909
H	-0.19367971016307	2.98281355257817	-3.25411393249092
H	1.42765025624925	2.42345730913921	-2.74503888158539
H	0.09681134913890	1.49538410302089	-5.21095324452924
H	1.30612920782625	0.51693803818457	-4.33907933290696
H	-1.74925717375853	0.43115437824658	-3.97705921728003
H	-0.61653470784670	-0.92618401029824	-3.70685171033700
P	-0.43777315171859	0.94131894235384	-2.00738021558965
O	0.67705495118346	0.07932487610359	-1.16906193845784
C	0.27939722272225	-1.11952663186209	-0.58211111650208
O	-0.68246860546405	-1.72627759701737	-0.99177254339037
C	1.14914054934034	-1.50198123843182	0.53775406407997
C	2.14423625760726	-0.64579985807167	1.02714721660091
C	2.90000849806913	-1.03173341478927	2.12661308360652
C	2.67053172638283	-2.26394670988127	2.73627640785342
C	1.67959137932400	-3.11649114809079	2.24909286607482
C	0.91616099281516	-2.73851162716664	1.15436923411126
H	2.31101387582630	0.31996244803428	0.56189683196151
H	3.66749138851088	-0.36727942106354	2.51246310825171
H	3.26419324106168	-2.56068377391013	3.59663739061382
H	1.50161291594773	-4.07504321052793	2.72753745713720
H	0.13662606653283	-3.38929770859421	0.77059010063721

3a-TS2

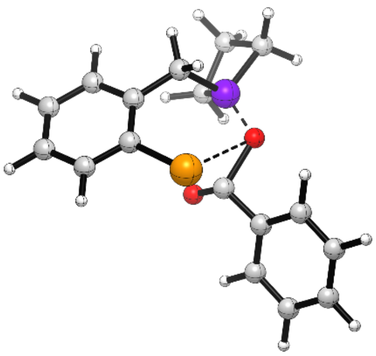

Final Gibbs Free Energy (Eh): -3547.85516637430

# of Imaginary Frequencies: 1

Coordinates:

Se	1.44367668030567	-0.42881857000790	1.75318232074416
C	0.66176627797180	1.31158222778474	1.92781423515856
C	0.59946059449894	2.19855391575207	0.83583313085067
C	-0.05481553727299	3.42415690058662	0.99188410143682
C	-0.62687431479951	3.78709967039758	2.20726139486167
C	-0.54565175712637	2.91784115874520	3.29166571159132
C	0.09002609235143	1.68816395530914	3.14813550452896
C	1.24020832090489	1.88276480433519	-0.49064771579057
H	-0.10611357539959	4.10458474613596	0.14483298085714
H	-1.12946911134513	4.74520427465793	2.30555353606525
H	-0.98502790740438	3.18869317672963	4.24810273893494
H	0.13635547993033	1.00231030851078	3.98937448738557
H	2.23236531445234	1.42901817259354	-0.36316169472481
H	1.35457057285135	2.79903337073938	-1.08409383717039
C	-1.28827826009202	1.48422188240162	-2.06132290300306

C	-0.53647358413025	2.07247532929179	-3.29792203319612
C	0.66877525624970	1.07510869368631	-3.31164181686450
H	-1.98687242151107	0.69221998596237	-2.34964233497373
H	-1.76925707396415	2.18066895881849	-1.37160503616729
H	-1.12365621624605	2.06235625518309	-4.21816971584215
H	-0.19547732038026	3.09128259018451	-3.09841307248173
H	0.44873692827810	0.18278013997226	-3.90705738775977
H	1.65711666463570	1.46561833887280	-3.56154557579261
P	0.30048560524027	0.74674079364608	-1.56302831127270
O	0.41479744928701	-0.75793061108425	-1.12848051507425
C	-0.27651879141843	-1.15865836901379	0.29554418999464
O	-1.30934633629073	-0.55484331049587	0.51384191192664
C	-0.09491379965204	-2.63697186186431	0.39032362954080
C	1.05669959346149	-3.27977572791494	-0.07174784018454
C	1.18681095748396	-4.65562090454000	0.07241415205480
C	0.17438675054002	-5.39777897724277	0.68126635373915
C	-0.97229921026069	-4.75639752246650	1.14289588170791
C	-1.11070045725963	-3.37862724307983	0.99806317107475
H	1.84819257368413	-2.70027597974472	-0.53604603706736
H	2.08222200627227	-5.15219724739609	-0.29121195565672
H	0.28167112884042	-6.47294432514622	0.79579287314214
H	-1.76442491957808	-5.32986544566486	1.61674472362061
H	-2.00215365310848	-2.87177355463503	1.35521075380576

3a-V

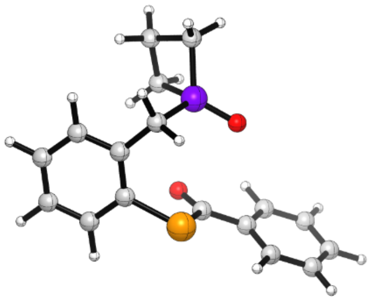

Final Gibbs Free Energy (Eh): -3547.90093048447

# of Imaginary Frequencies: 0

Coordinates:

Se	1.67214327517271	-2.18617547606076	-1.85856741088206
C	0.27036185429204	-1.76059301165985	-0.60757180592828
C	-0.99096457176148	-1.31767266267798	-1.03928521850133
C	-1.94908008255705	-1.03327662062282	-0.05588868426122
C	-1.67135419504064	-1.17757470220966	1.29784537698700
C	-0.41288766059051	-1.60936272742309	1.70786672195698
C	0.55587982090219	-1.89614497670252	0.75225260832588
C	-1.36933170806330	-1.14323905022883	-2.48240337302419
H	-2.93067598032723	-0.68691690381560	-0.37016873083924
H	-2.43745634656943	-0.94546340804526	2.03269782350759
H	-0.18233926152214	-1.72068784729738	2.76361525715032
H	1.54130858485353	-2.23300176789109	1.06045726806360
H	-0.53621847094115	-0.74635828746209	-3.07407648466728
H	-2.20191569527387	-0.43491002070290	-2.55373559205869
C	-2.94807873014936	-3.65218241291752	-2.20287452442083

C	-4.16430557263159	-2.79210347984510	-2.65762684211265
C	-3.59567624880964	-2.31793153922463	-4.02704539348224
H	-3.02989388706384	-4.68630770293769	-2.55637675365347
H	-2.69011039861412	-3.64368863925162	-1.14161261776884
H	-5.10092375262976	-3.35034488202835	-2.73499296977887
H	-4.31517153578206	-1.94438003226769	-1.98308966774277
H	-3.83958117478951	-3.02340315859141	-4.82936029303572
H	-3.82385488886855	-1.29787545296261	-4.34605130381877
P	-1.90854586622921	-2.68826151383408	-3.37803695156444
O	-0.83952403322021	-3.29997657527258	-4.24224646851087
C	1.14832312872900	-4.09211138106342	-2.07673647187632
O	0.23620104204890	-4.55377212391414	-1.43444079382859
C	1.95272799349575	-4.86611163576387	-3.05087501570672
C	3.13464682595261	-4.37449892826711	-3.61385648553626
C	3.84806126503508	-5.14846013750286	-4.52137070024112
C	3.38360098545219	-6.41294574262817	-4.87809978837890
C	2.20408910449813	-6.90631635325054	-4.32084865629467
C	1.49183919967225	-6.14052780789412	-3.40826537663106
H	3.51294009835994	-3.39265903853523	-3.34218749783384
H	4.76851142612791	-4.76381806294842	-4.95070482662027
H	3.94097432138283	-7.01437961665824	-5.59101107484172
H	1.83930474510381	-7.89051803099329	-4.60096554315222
H	0.57057638894130	-6.51294829410471	-2.97136175818482

para-F

3e-IV

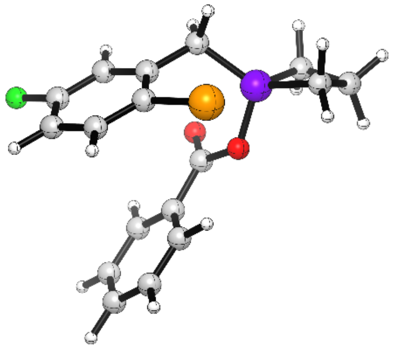

Final Gibbs Free Energy (Eh): -3647.01317932793

# of Imaginary Frequencies: 0

Coordinates:

Se	0.26294044501099	3.07840254315144	0.21547836453738
C	-0.76169982632045	1.78898094112598	1.17922555704507
C	-1.70968947416534	1.00279751913672	0.49374108811490
C	-2.43787269731768	0.02087088345133	1.17316255675355
C	-2.21593443191931	-0.15792455680928	2.52402823510173
C	-1.29478308725333	0.59376671000045	3.23487769469952
C	-0.57263725819196	1.56570330546723	2.55153166484208
C	-1.93622379629031	1.19106720388031	-0.97764600110146
H	-3.16028473870566	-0.60092457732432	0.65266452088350
F	-2.92635667858695	-1.11932859234711	3.17999614053206
H	-1.14379074217314	0.41928880707426	4.29612321034824
H	0.15949346259644	2.15822534238125	3.09264206477521
H	-2.27578239027195	2.20900965860870	-1.21695329287662
H	-2.67246999060011	0.47722577383310	-1.36620538106548

C	0.44340507470211	2.11645044964705	-3.09357149462822
C	0.36297767675592	1.06709446393029	-4.24353573371294
C	-0.74217247662822	0.16355941015569	-3.62499391406514
H	-0.19706910834102	2.99057528328381	-3.25647530722612
H	1.42791720427667	2.43871166946084	-2.75018836571141
H	0.09406598234880	1.49843881865732	-5.21007843013650
H	1.30689349359917	0.52513105055980	-4.33732182791363
H	-1.74717162366920	0.43921058170644	-3.96586859399272
H	-0.61403275578275	-0.91776699249856	-3.69689418212046
P	-0.42975131942152	0.95426327565339	-1.99989684141495
O	0.68522074636819	0.08410442475192	-1.16841892614608
C	0.28419738504501	-1.11314763054766	-0.58122648236151
O	-0.68260271810547	-1.71513492851134	-0.98661283603962
C	1.15335438673937	-1.49865406793018	0.53819130847721
C	2.16108551481279	-0.65202986301126	1.01822407266294
C	2.91459651331902	-1.03935933294528	2.11873691380314
C	2.66967812943724	-2.26310687105335	2.73937970149723
C	1.66581753398445	-3.10594022672878	2.26184833311151
C	0.90514966055394	-2.72681403350647	1.16560234909425
H	2.33981133335626	0.30746272735117	0.54458829532446
H	3.69237592885698	-0.38252239991721	2.49694101700734
H	3.26134624647068	-2.56074712395057	3.60080317798245
H	1.47552450024682	-4.05770746498902	2.74903869946077
H	0.11534997776043	-3.36975738344752	0.78963680579664

3e-TS2

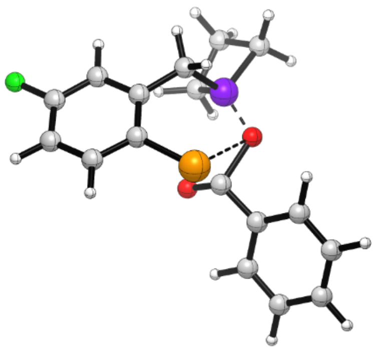

Final Gibbs Free Energy (Eh): -3646.99991297936

# of Imaginary Frequencies: 1

Coordinates:

Se	1.53446499248125	-0.37939637182154	1.70520552790835
C	0.69887298402333	1.33174884701986	1.90084127525694
C	0.56579995253415	2.20841618150302	0.80630029500975
C	-0.12897788138369	3.41027886247754	0.96653961243226
C	-0.65809744538257	3.72372413792829	2.20490473105534
C	-0.52850274416694	2.89676773363946	3.30515291669763
C	0.15074241648497	1.69399168723685	3.13580146694821
C	1.17663525748801	1.91134789605445	-0.53732398316084
H	-0.24643584002551	4.10032767891332	0.13567809205886
F	-1.32800115534094	4.89892459237069	2.34242368320816
H	-0.95523151033430	3.17800128739247	4.26301605070404
H	0.24914735677288	1.01932116062281	3.98093440926531
H	2.18665216007702	1.49245050827297	-0.43342743756425
H	1.24357785615924	2.82838125361207	-1.13628273513407
C	-1.36327852930425	1.42873008307020	-2.06447127174466

C	-0.64360015910182	2.02605328485299	-3.31612333275140
C	0.59051623853603	1.06443908790202	-3.33585551000044
H	-2.04630289615493	0.61753751890761	-2.33611691735006
H	-1.85098965706294	2.11894770950178	-1.37321604023331
H	-1.24146312598168	1.98752218566569	-4.22863627410217
H	-0.33116647624782	3.05668898536471	-3.13171606705481
H	0.39180269253615	0.16232509484090	-3.92389957884009
H	1.56447022912030	1.48263156582670	-3.59730190825327
P	0.25057411266255	0.73755124774677	-1.58194700812290
O	0.41534267167097	-0.75905990189563	-1.13577678592862
C	-0.22559803069611	-1.15671614352541	0.31008662060305
O	-1.25754706024655	-0.56235085668802	0.55696703046823
C	-0.02121565494078	-2.63073045915263	0.41401288828146
C	1.13268552355247	-3.25984659846138	-0.06128196080161
C	1.28511332517178	-4.63246418004366	0.09060690674882
C	0.29325649126310	-5.38468494951304	0.72090642038537
C	-0.85530248809555	-4.75675028853485	1.19599378756352
C	-1.01631462015018	-3.38227174227797	1.04325840354175
H	1.90896526899700	-2.67197989611348	-0.54079944729308
H	2.18210215330184	-5.11879333461735	-0.28282651394975
H	0.41761402530220	-6.45745211287406	0.84053144232993
H	-1.63117457006135	-5.33806046880888	1.68684944416331
H	-1.90913586345734	-2.88555128639524	1.41099176765504

3e-V

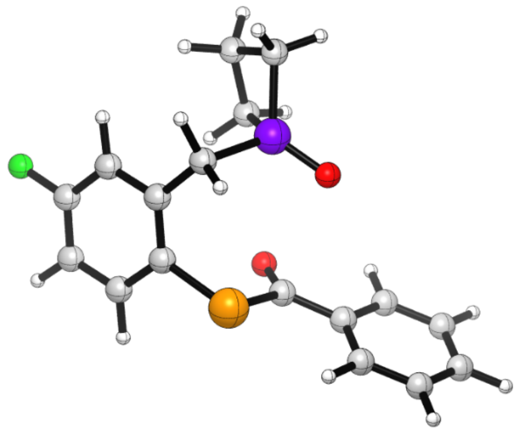

Final Gibbs Free Energy (Eh): -3647.04496148915

# of Imaginary Frequencies: 0

Coordinates:

Se	1.67374976071391	-2.18243708985697	-1.85845030112483
C	0.27250372008241	-1.75916362520393	-0.61178175515248
C	-0.99000144025111	-1.31643337907239	-1.04438547525941
C	-1.95704767669199	-1.03586207420654	-0.07084082285593
C	-1.64946852416225	-1.19537996729759	1.26635068145723
C	-0.41071793108773	-1.62065856597811	1.71135883379645
C	0.55252446041546	-1.89921990763547	0.74866554364607
C	-1.36661394258220	-1.14119971392893	-2.48665106009605
H	-2.94542976290600	-0.68957075093511	-0.35886127882947
F	-2.60869192185490	-0.91268451675931	2.18379474555211
H	-0.20596586532813	-1.72711453164605	2.77181864282076
H	1.53605426174872	-2.23553325143579	1.06196230440942

H	-0.52885056735117	-0.75081129430498	-3.07559299561983
H	-2.19545822602598	-0.42929798681739	-2.56129706188051
C	-2.94819012650437	-3.64874173706729	-2.19981634264104
C	-4.16301827548134	-2.78529342550441	-2.65208061757441
C	-3.59680950727723	-2.31452743335832	-4.02379705445633
H	-3.03379779791818	-4.68262779845456	-2.55308088860021
H	-2.68759897017797	-3.64133438024046	-1.13922264302438
H	-5.10131454322748	-3.34100867341578	-2.72639656256951
H	-4.30966559954495	-1.93623768898995	-1.97828994389156
H	-3.84445618473364	-3.02076441782270	-4.82428129374152
H	-3.82266795016755	-1.29434199536832	-4.34383411379395
P	-1.90976744961788	-2.68897483314839	-3.37878229014726
O	-0.84127531952535	-3.30103808321190	-4.24257804279088
C	1.14997758011092	-4.09018416132708	-2.07551559025551
O	0.23784360557948	-4.54959226149699	-1.43188576377549
C	1.95385380596092	-4.86505869186074	-3.04873683973027
C	3.13085199964709	-4.36996533735183	-3.61900560781955
C	3.84376679045367	-5.14410578181457	-4.52670725027386
C	3.38372143120479	-6.41224167102072	-4.87612987538535
C	2.20931132542424	-6.90920064173488	-4.31134757118113
C	1.49746155904074	-6.14318230541749	-3.39872142576542
H	3.50580686825591	-3.38518951231453	-3.35338172409330
H	4.76023649825851	-4.75673339850289	-4.96202136936015
H	3.94061893364390	-7.01378038865700	-5.58931890051187
H	1.84818117081237	-7.89636870847776	-4.58565489869417
H	0.58020015621929	-6.51847661577479	-2.95590123348084

meta-F

3g-IV

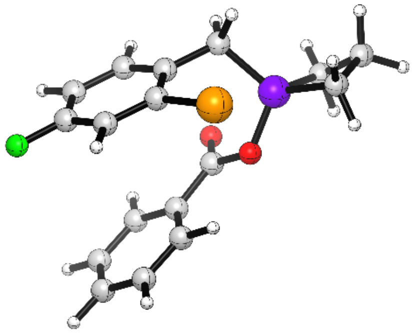

Final Gibbs Free Energy (Eh): -3647.01206850849

# of Imaginary Frequencies: 0

Coordinates:

Se	2.44752864977635	1.88408412558919	0.34137867933712
C	0.84133549345278	1.49252567765199	1.28870698578719
C	-0.42303421668824	1.67376753932898	0.68351840683797
C	-1.58643208654882	1.34265044848677	1.38399796982714
C	-1.54019443355244	0.82972356786279	2.67493890030024
C	-0.28677021052549	0.65883529288043	3.24095715593402
C	0.89012097826922	0.97907332226313	2.59533083246857
C	-0.54233539792799	2.19375435987963	-0.72206245370396
H	-2.55102335869146	1.47608484780509	0.90145795621564
H	-2.43961511328874	0.56339708708277	3.22031642045832
F	-0.20989983651486	0.13954015616134	4.49836039631444
H	1.83994674559550	0.81492600432902	3.09489845765524
H	-0.00543459630384	3.14204700062551	-0.86212228328839
H	-1.59239225279950	2.33839619078239	-1.00029615503610

C	-0.78650564665678	0.98226579651978	-3.53467069825592
C	0.30894640421415	1.88081399387228	-4.18884640928217
C	1.43847427015284	1.65453876899576	-3.13191003710017
H	-0.77603614458229	-0.03862813105180	-3.93037948876247
H	-1.80966001976213	1.36216228769005	-3.50633764261822
H	0.58758180453144	1.56821723791474	-5.19683842830146
H	0.00018581277395	2.92841040411514	-4.20352859633668
H	2.11380812259411	0.83346808194694	-3.39556615506421
H	2.01346168531880	2.52058542391084	-2.80081635196872
P	0.16700954873136	1.06234566464052	-1.98237783933899
O	0.49807488619661	-0.38109765649663	-1.29207463563669
C	-0.56219001301996	-1.04250532397973	-0.66470711805882
O	-1.70304784239051	-0.82676696631779	-1.00174171384805
C	-0.10550533938256	-1.95607986173711	0.38687515105095
C	1.25227602988483	-2.11292428332692	0.69542269509779
C	1.62444220523305	-2.94177034393941	1.74528774224780
C	0.65234748600059	-3.60703821490868	2.49083979661030
C	-0.69949008288560	-3.45008087095953	2.18485424811247
C	-1.08186472957952	-2.62800314102333	1.13502162009686
H	2.00737492901041	-1.57926339195789	0.12842923184467
H	2.67573382796565	-3.06292917861088	1.98907801486151
H	0.94886236379976	-4.24871196788688	3.31605957628522
H	-1.45474626591932	-3.96604799967189	2.77021692188465
H	-2.13105932024321	-2.48917008166400	0.89324693921439

3g-TS2

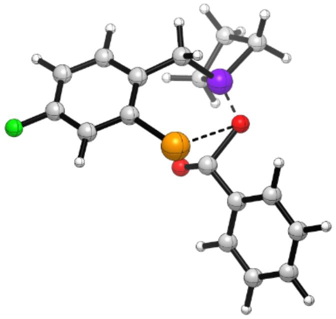

Final Gibbs Free Energy (Eh): -3647.00120028873

# of Imaginary Frequencies: 1

Coordinates:

Se	1.69676034301332	0.26155791239160	1.55034841804563
C	0.79850875295704	1.92114852061801	1.23036385026470
C	0.51774148892326	2.36554650236937	-0.07706628554051
C	-0.20402282073045	3.54912709666240	-0.25252804672653
C	-0.63570212627527	4.31122157224239	0.82859598712587
C	-0.32522352136105	3.85365808956444	2.09588410647668
C	0.37008514589636	2.68027024825998	2.32410109279916
C	0.99589743723518	1.62225770072014	-1.29614237397701
H	-0.42186631843392	3.89171246812777	-1.26102049718072
H	-1.18942379310914	5.23451325091010	0.69191554240071
F	-0.72971335102018	4.58209301132381	3.17100275319070
H	0.56668111451575	2.35742450875754	3.34150852764302
H	2.02978659660789	1.27162437427765	-1.17633055396232
H	0.95605830135813	2.27544311971190	-2.17669094600642
C	-1.68536933889849	0.62408934969613	-2.19763431461813

C	-1.18116151084607	0.76236194320262	-3.67019854029317
C	0.10267085618611	-0.12436704710941	-3.54743773407529
H	-2.35470781678005	-0.23281855009330	-2.07322575391566
H	-2.10892185292431	1.50739399844657	-1.71527122375829
H	-1.89168117533481	0.39644776939772	-4.41378139720889
H	-0.91981061366873	1.79821743236493	-3.89893911217330
H	-0.10435445204427	-1.17500095898972	-3.77490375314207
H	1.00409120891057	0.20716598442885	-4.06668927336083
P	0.01677503509811	0.15399392634654	-1.75376240472473
O	0.32769006954135	-1.09595581007347	-0.85719579622308
C	-0.12363413522082	-0.99317151414383	0.72536617051123
O	-1.16813096420171	-0.39364883093021	0.88395104616260
C	0.20170454565318	-2.34092922354624	1.27808629205149
C	1.34929790113979	-3.04700379974233	0.90749731562159
C	1.61221717500688	-4.28683222586477	1.47727145084783
C	0.73814231261333	-4.82669251424475	2.42145936695330
C	-0.40278670302534	-4.12030921818240	2.79288692579855
C	-0.67389470069301	-2.87925124354452	2.22337596391233
H	2.03361128600891	-2.62156034708993	0.18023824506611
H	2.50297771919151	-4.83586121808068	1.18438078029422
H	0.94838658530194	-5.79574217255788	2.86581069291691
H	-1.08731535551910	-4.53610741739214	3.52721691798899
H	-1.56136332507188	-2.32201668823488	2.50755656081529

3g-V

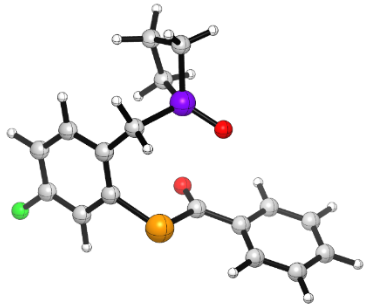

Final Gibbs Free Energy (Eh): -3647.04496148915

# of Imaginary Frequencies: 0

Coordinates:

Se	1.67880859631640	-2.17064055257961	-1.87258957396740
C	0.28165256236827	-1.75817464572939	-0.61341756521930
C	-0.98793897973473	-1.32968213720683	-1.03478307508292
C	-1.94381406780327	-1.06273761456850	-0.04453665125475
C	-1.66739662957330	-1.20441049061115	1.30931112361277
C	-0.39905033984987	-1.62029260570812	1.67098088886463
C	0.58555226449352	-1.89762371004184	0.74158199537872
C	-1.37828778554575	-1.15498810097883	-2.47486055466353
H	-2.93273546397563	-0.72932455930774	-0.34789081586697
H	-2.41518088229234	-0.99048562252287	2.06665603454905
F	-0.10419304300867	-1.76000763010588	2.98946776096519
H	1.56795861265472	-2.22240401359125	1.06864833806947
H	-0.55194491717297	-0.75310375698700	-3.07244058020177
H	-2.21333158130222	-0.44881004784748	-2.53821835636939
C	-2.98977285754184	-3.64090973128389	-2.19921252278111

C	-4.18816172881890	-2.76882951254742	-2.67821997906461
C	-3.59399571874968	-2.31331596627558	-4.04319660736786
H	-3.08045176742702	-4.67661496355895	-2.54590477628395
H	-2.74780451158497	-3.62779092568826	-1.13407599405095
H	-5.13066454259434	-3.31567760047991	-2.76440381335020
H	-4.33759958404665	-1.91369365368082	-2.01274654405401
H	-3.83577177511208	-3.02210564759360	-4.84319331076535
H	-3.80420448195717	-1.29298711099661	-4.37350448260463
P	-1.92144174099748	-2.69985338079420	-3.36704404380538
O	-0.84725955866999	-3.33263803271606	-4.20929924929578
C	1.16422741444243	-4.08442275024008	-2.07038169967265
O	0.26352721233787	-4.54557058487944	-1.41307311357670
C	1.96379688535838	-4.85841300735298	-3.04728506655687
C	3.13510741601406	-4.36101583571654	-3.62687865939520
C	3.84445871139402	-5.13556394426629	-4.53699929257436
C	3.38615946303703	-6.40620105146679	-4.87951644223376
C	2.21721876711205	-6.90533176337578	-4.30536613686257
C	1.50905753114533	-6.13904992034488	-3.39015122721696
H	3.50903917496964	-3.37447093940524	-3.36627154815114
H	4.75684343482519	-4.74662854490994	-4.97940154040614
H	3.94024465701812	-7.00808670921783	-5.59459296068932
H	1.85740371588328	-7.89442932584786	-4.57437292602150
H	0.59598479849624	-6.51592576773434	-2.94013230836994

8 References

(1) Trevor V. Nykaza; Julian C. Cooper; Alexander T. Radosevich. Anti-1,2,2,3,4,4-Hexamethylphosphetane 1-Oxide. *Org. Synth.* **2019**, *96*, 418–435. https://doi.org/10.15227/orgsyn.096.0418.

(2) Handoko; Satishkumar, S.; Panigrahi, N. R.; Arora, P. S. Rational Design of an Organocatalyst for Peptide Bond Formation. *J. Am. Chem. Soc.* **2019**, *141* (40), 15977–15985. https://doi.org/10.1021/jacs.9b07742.

(3) Neese, F. Software Update: The ORCA Program System—Version 5.0. *WIREs Computational Molecular Science* **2022**, *12* (5), e1606. https://doi.org/10.1002/wcms.1606.

(4) Grimme, S.; Hansen, A.; Ehlert, S.; Mewes, J.-M. r2SCAN-3c: A "Swiss Army Knife" Composite Electronic-Structure Method. *The Journal of Chemical Physics* **2021**, *154* (6), 064103. https://doi.org/10.1063/5.0040021.

(5) Marenich, A. V.; Cramer, C. J.; Truhlar, D. G. Universal Solvation Model Based on Solute Electron Density and on a Continuum Model of the Solvent Defined by the Bulk Dielectric Constant and Atomic Surface Tensions. *J. Phys. Chem. B* **2009**, *113* (18), 6378–6396. https://doi.org/10.1021/jp810292n.

(6) Garcia-Ratés, M.; Neese, F. Effect of the Solute Cavity on the Solvation Energy and Its Derivatives within the Framework of the Gaussian Charge Scheme. *Journal of Computational Chemistry* **2020**, *41* (9), 922–939. https://doi.org/10.1002/jcc.26139.

(7) Riplinger, C.; Sandhoefer, B.; Hansen, A.; Neese, F. Natural Triple Excitations in Local Coupled Cluster Calculations with Pair Natural Orbitals. *The Journal of Chemical Physics* **2013**, *139* (13), 134101. https://doi.org/10.1063/1.4821834.

(8) Ásgeirsson, V.; Birgisson, B. O.; Bjornsson, R.; Becker, U.; Neese, F.; Riplinger, C.; Jónsson, H. Nudged Elastic Band Method for Molecular Reactions Using Energy-Weighted Springs Combined with Eigenvector Following. *J. Chem. Theory Comput.* **2021**, *17* (8), 4929–4945. https://doi.org/10.1021/acs.jctc.1c00462.

(9) Legault, C. Y. CYLview, 2009. https://www.cylview.org/.

(10) E. D. Glendening, J. K. Badenhoop, A. E. Reed,; J. E. Carpenter, J. A. Bohmann, C. M. Morales, P. Karafiloglou,; C. R. Landis, and F. Weinhold. NBO 7.0, 2018.

(11) Glendening, E. D.; Landis, C. R.; Weinhold, F. NBO 7.0: New Vistas in Localized and Delocalized Chemical Bonding Theory. *Journal of Computational Chemistry* **2019**, *40* (25), 2234–2241. https://doi.org/10.1002/jcc.25873.

(12) Müller, M.; Hansen, A.; Grimme, S. ωB97X-3c: A Composite Range-Separated Hybrid DFT Method with a Molecule-Optimized Polarized Valence Double-ζ Basis Set. *The Journal of Chemical Physics* **2023**, *158* (1), 014103. https://doi.org/10.1063/5.0133026.

(13) Caldeweyher, E.; Mewes, J.-M.; Ehlert, S.; Grimme, S. Extension and Evaluation of the D4 London-Dispersion Model for Periodic Systems. *Phys. Chem. Chem. Phys.* **2020**, *22* (16), 8499–8512. https://doi.org/10.1039/D0CP00502A.

9 NMR Spectra of Compounds

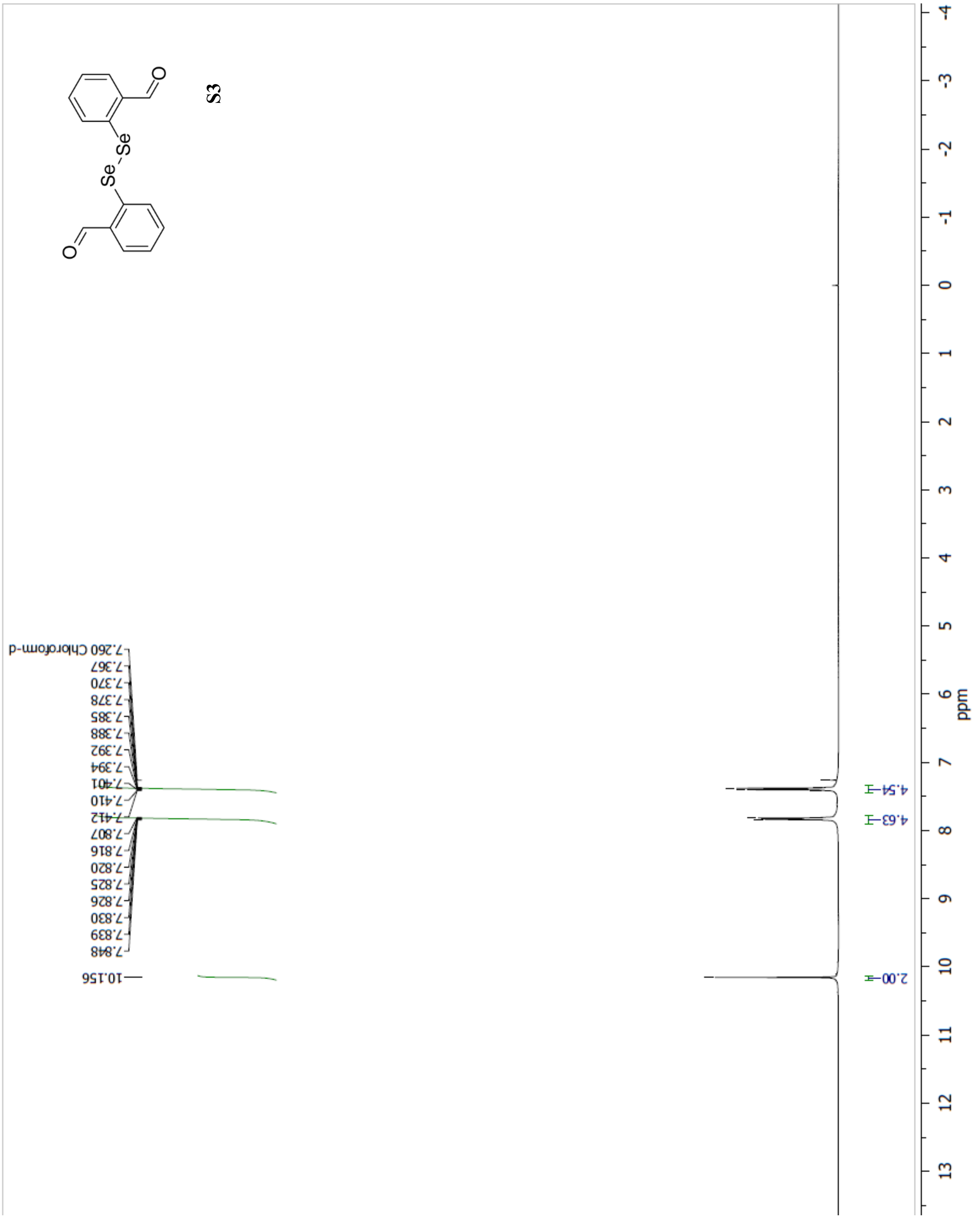

^{1}H NMR in $CDCl_3$

^{13}C NMR in $CDCl_3$

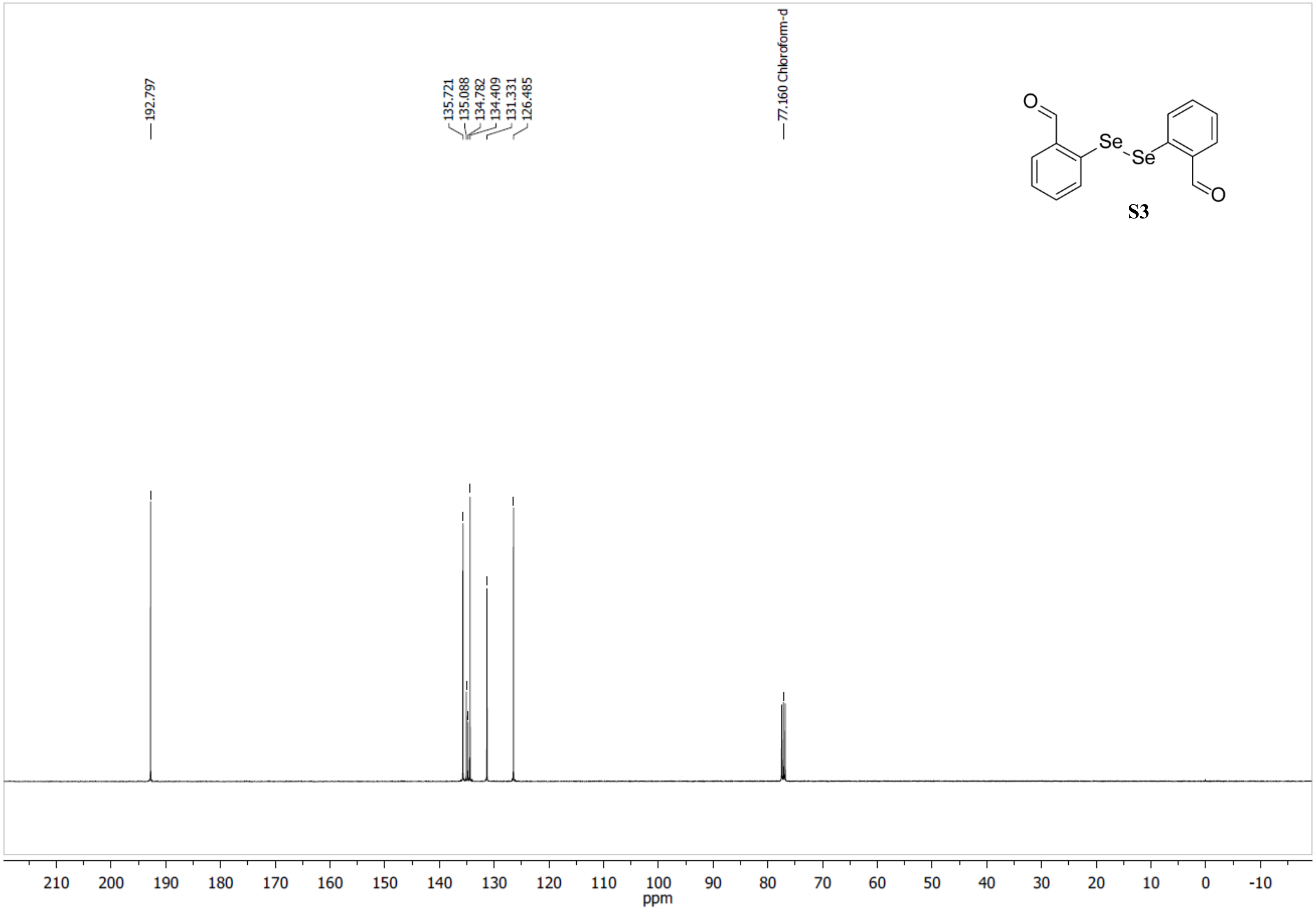

S3

^{77}Se NMR in $CDCl_3$

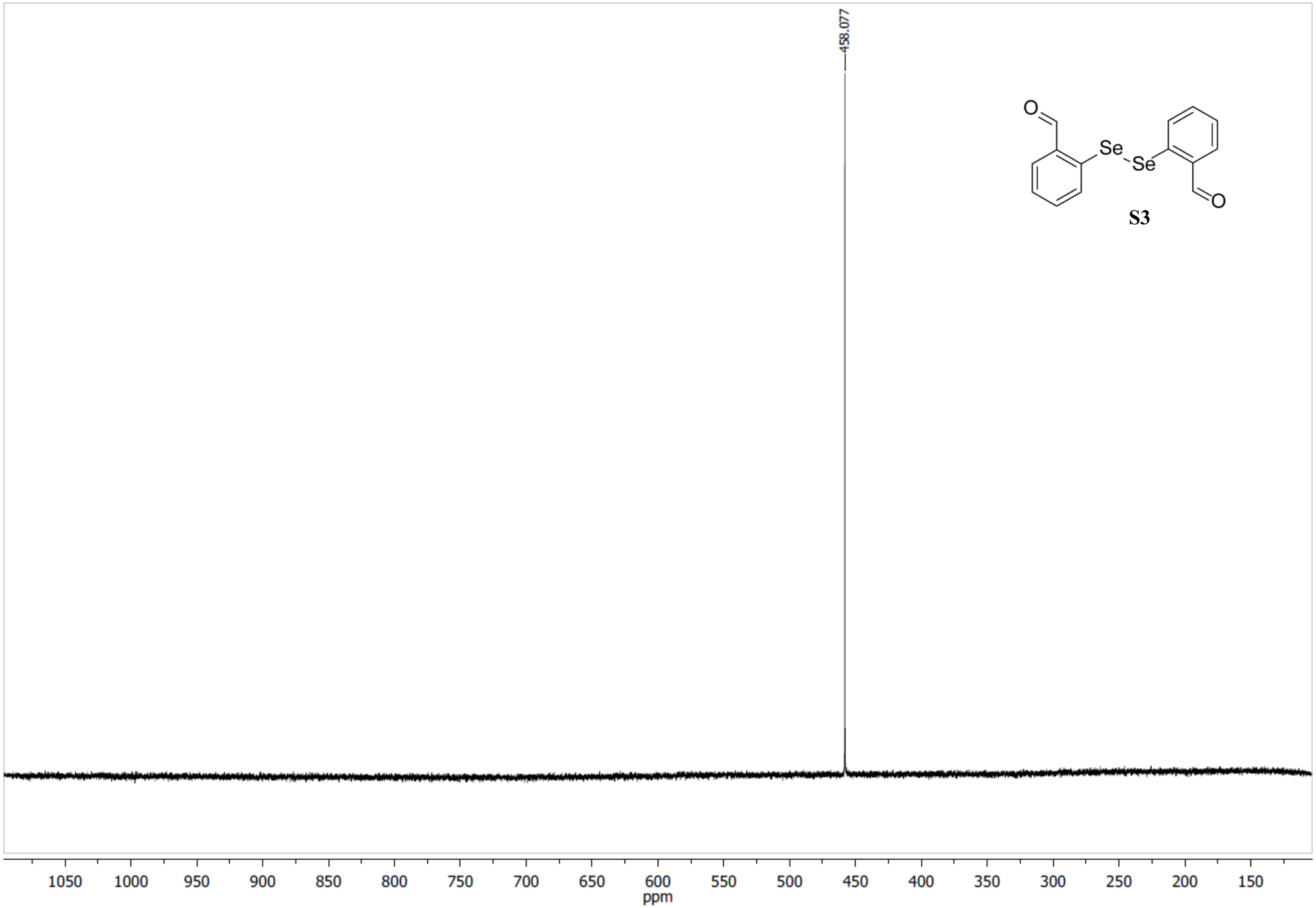

^1H NMR in DMSO-d_6

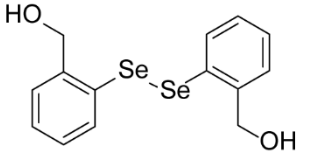

S4

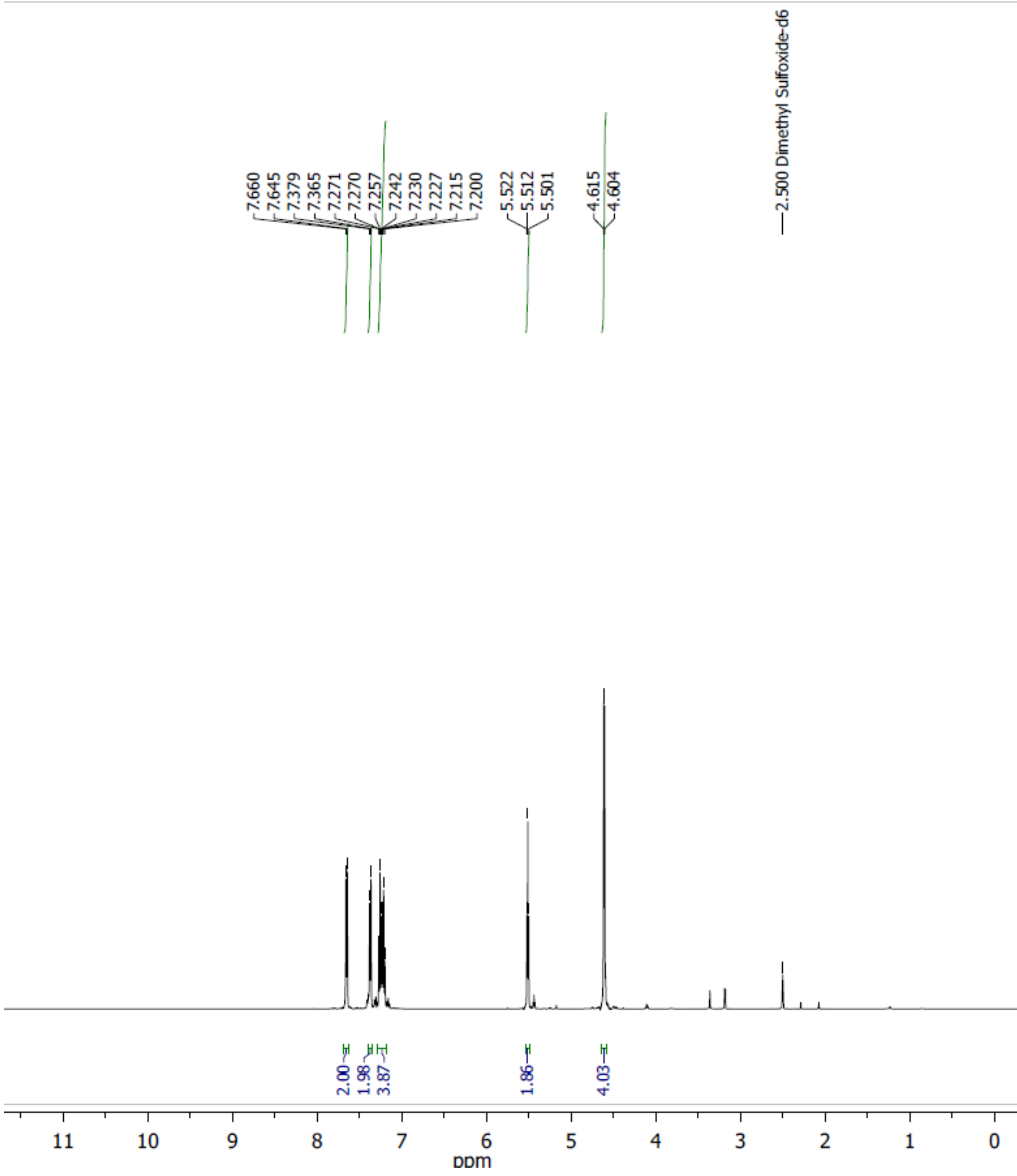

^{13}C NMR DMSO-d_6

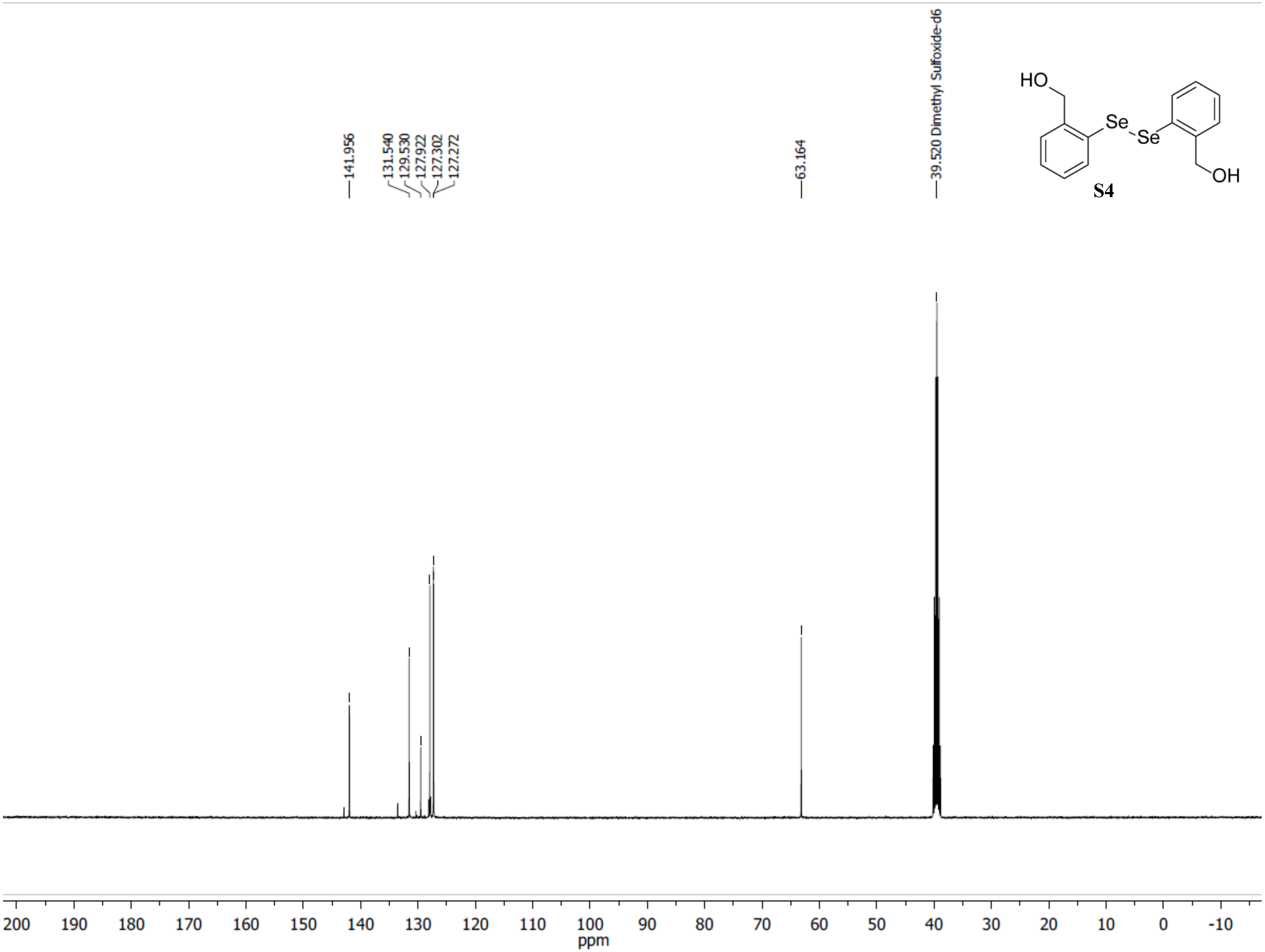

S4

^{77}Se NMR DMSO-d_6

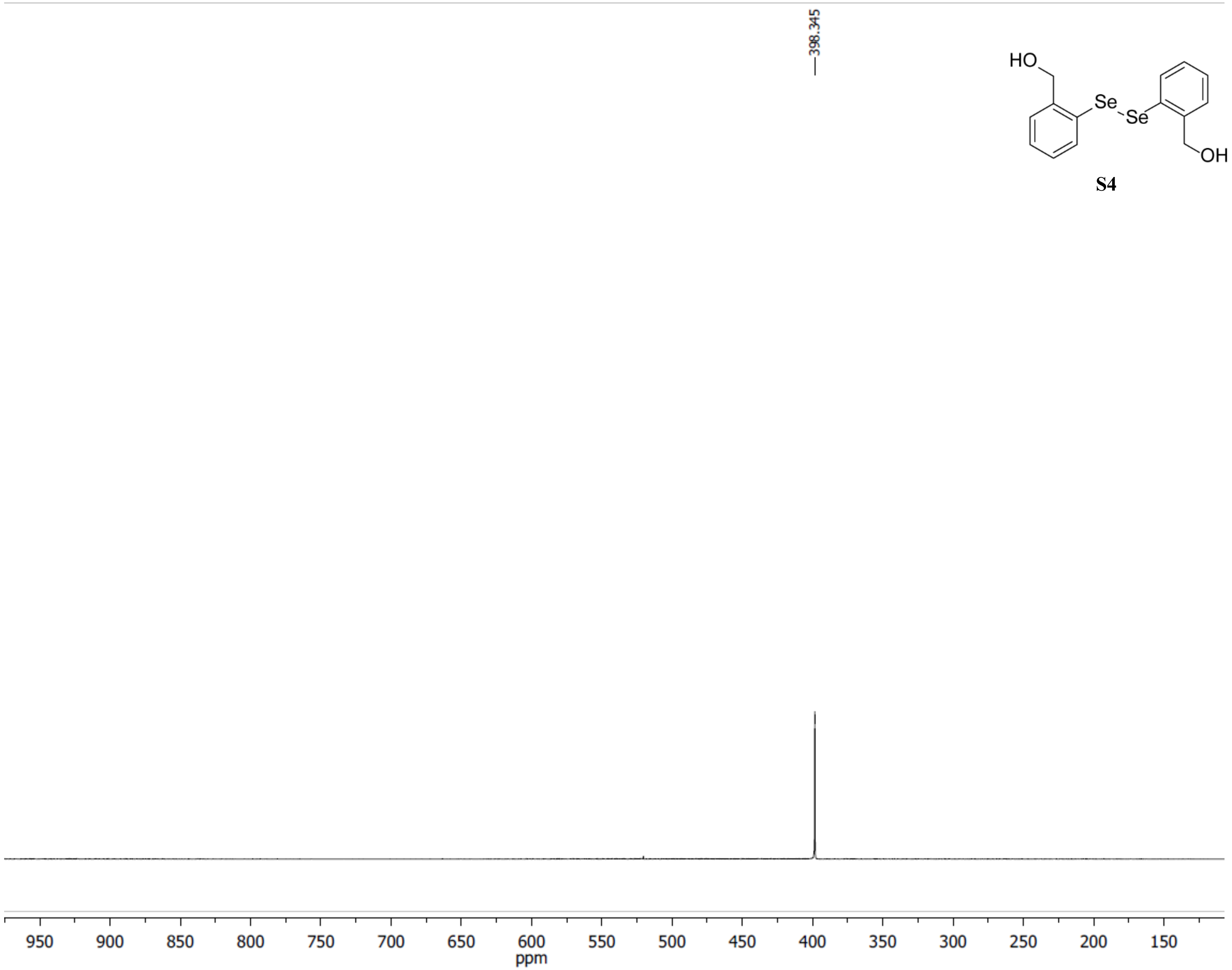

^{1}H NMR in $CDCl_3$

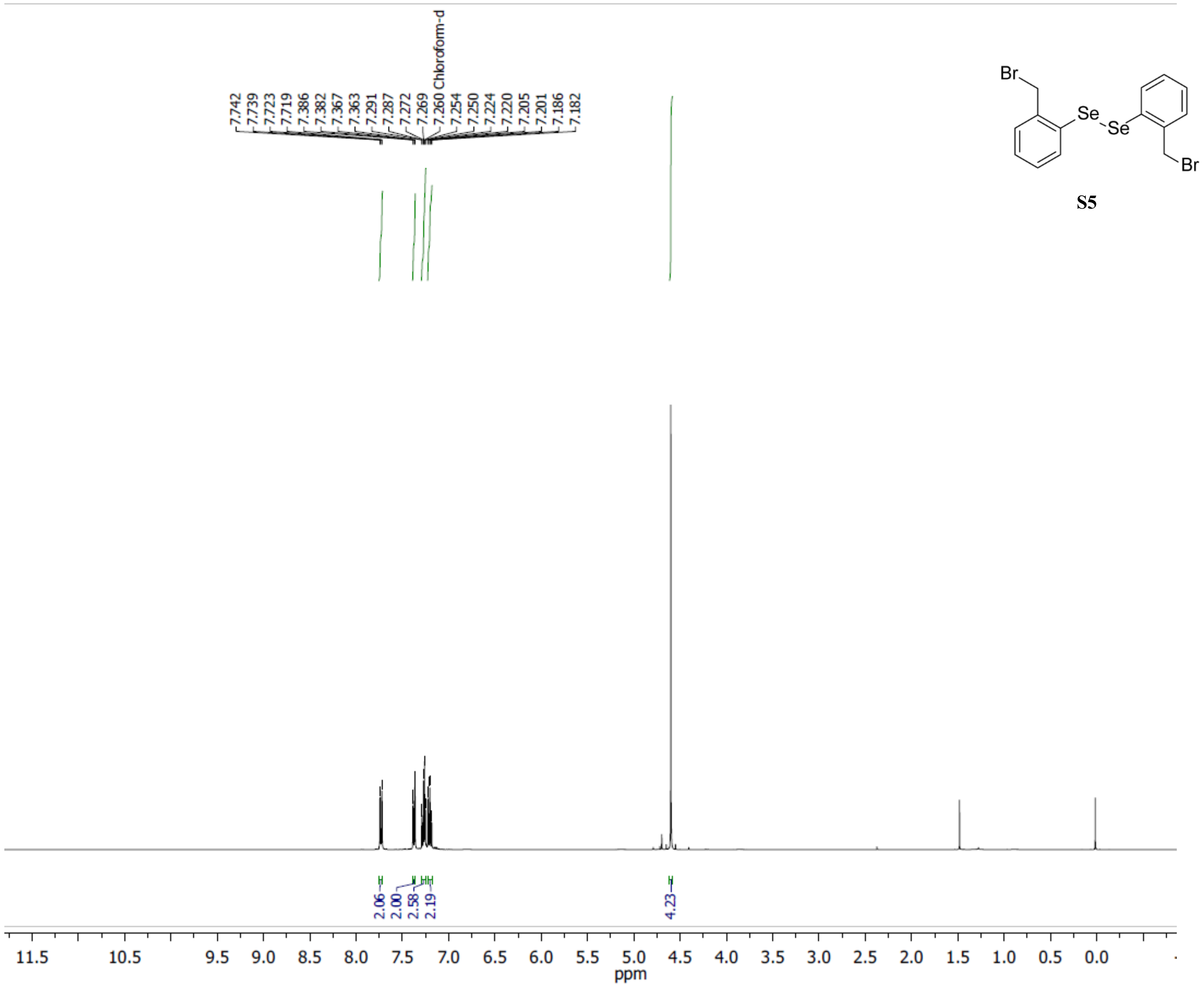

S5

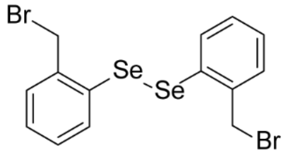

S5

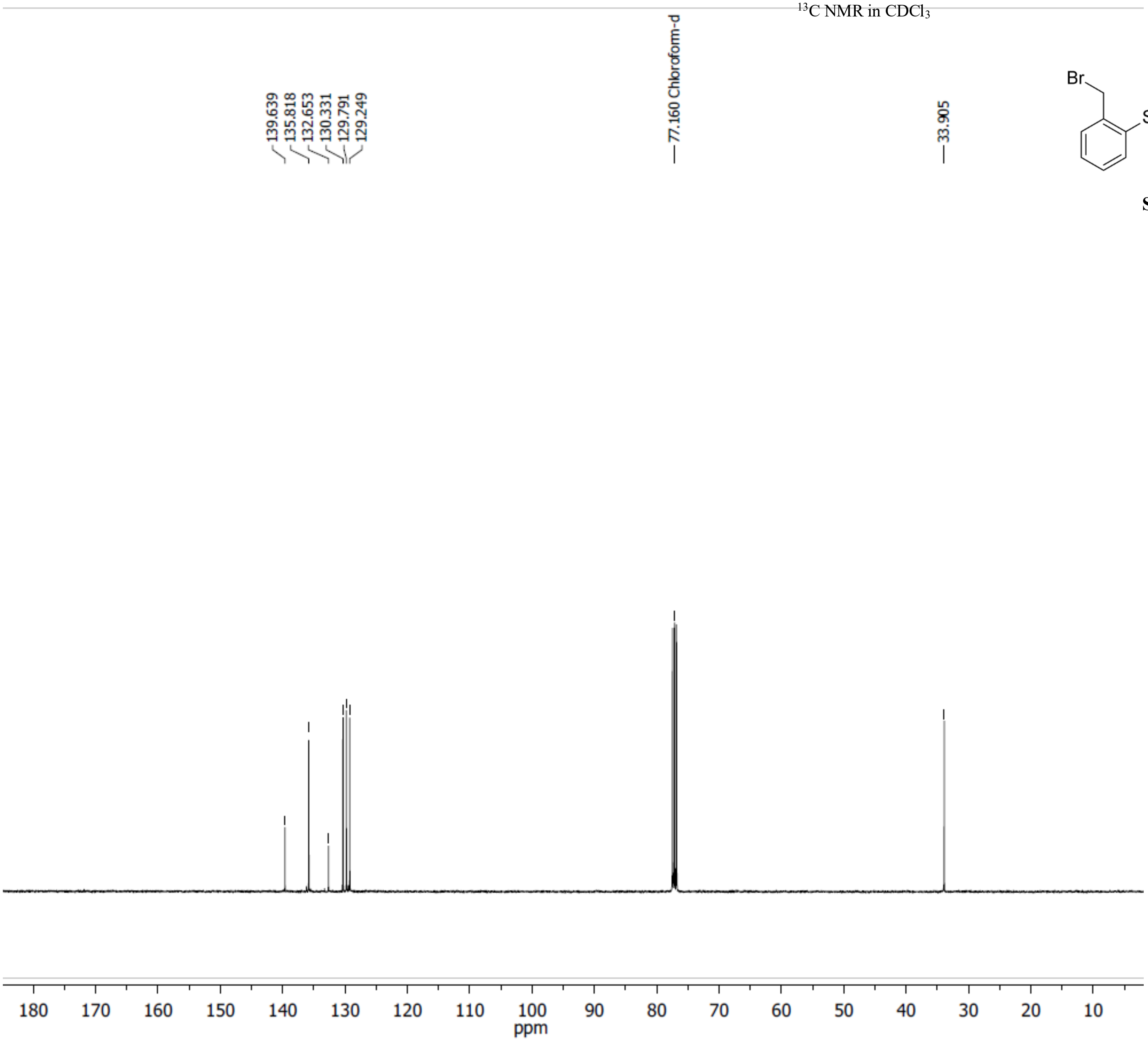

^{13}C NMR in $CDCl_3$
139.639
135.818
132.653
130.331
129.791
129.249
77.160 Chloroform-d
33.905
180
170
160
150
140
130
120
110
100
90
80
70
60
50
40
30
20
10
ppm

^{77}Se NMR $CDCl_3$

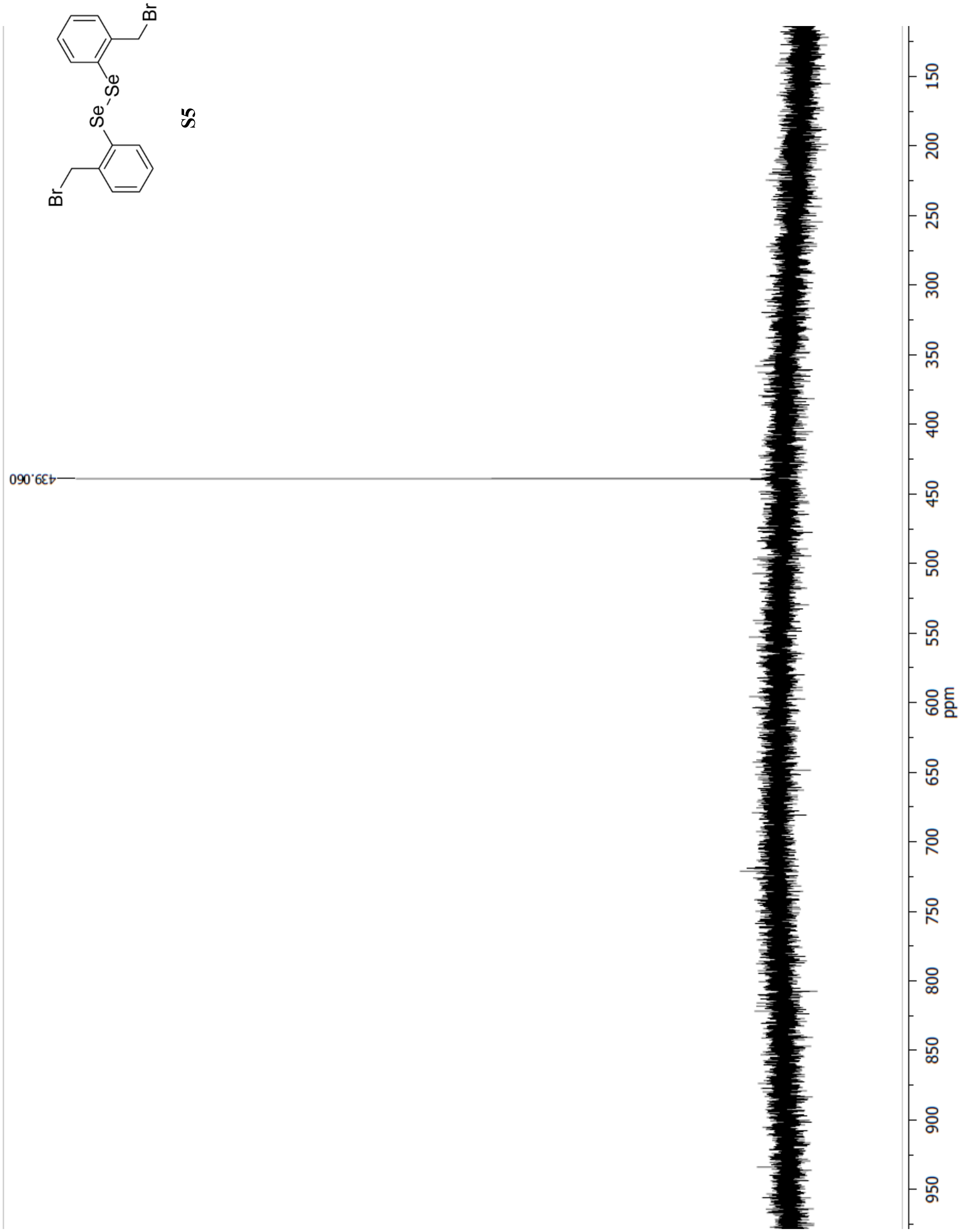

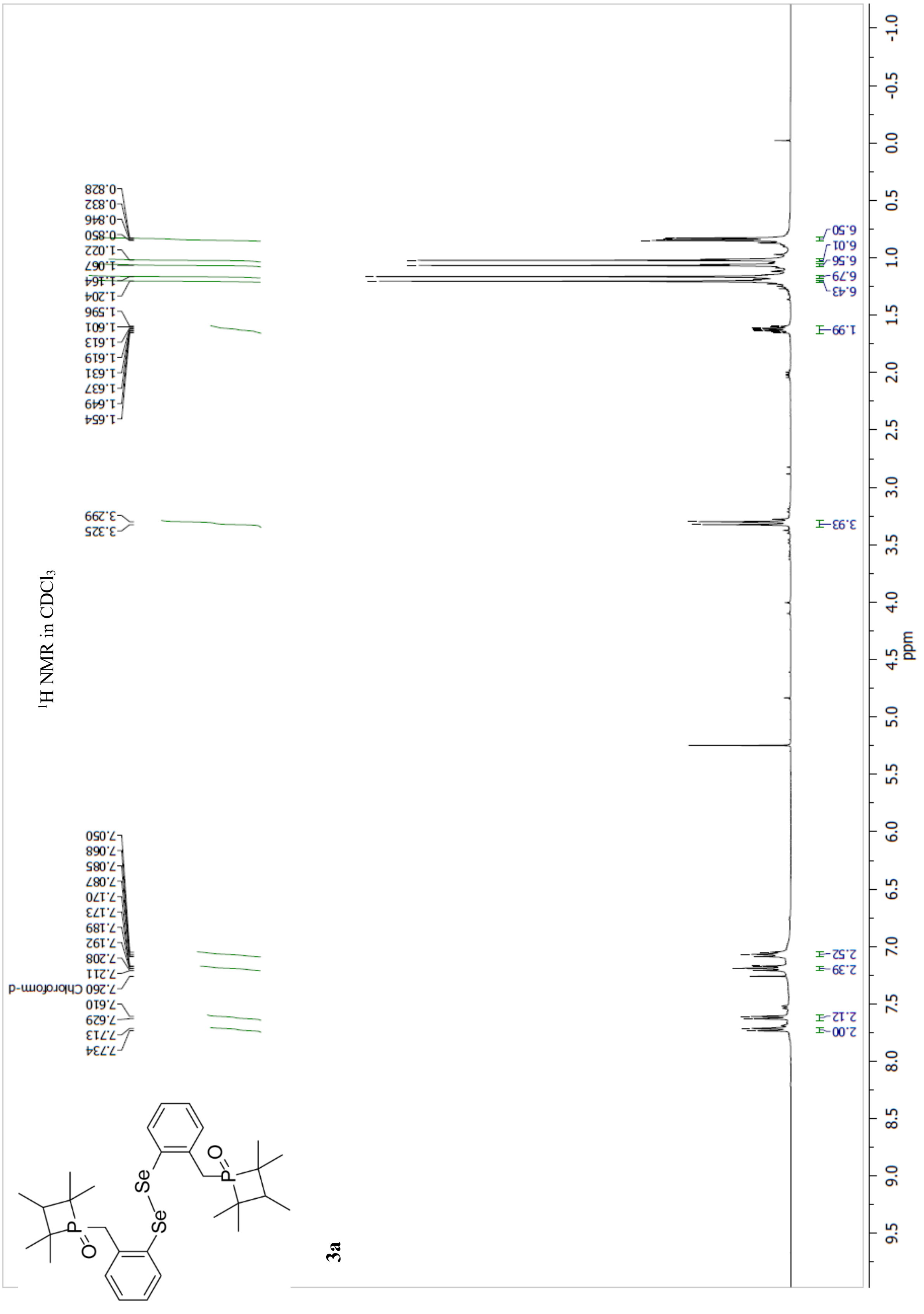
1H NMR in CDCl3
3a
ppm
7.734
7.713
7.629
7.610
7.260 Chloroform-d
7.211
7.208
7.192
7.189
7.173
7.170
7.087
7.085
7.068
7.050
3.325
3.299
1.654
1.649
1.637
1.631
1.619
1.613
1.601
1.596
1.204
1.164
1.067
1.022
0.850
0.846
0.832
0.828
2.00
2.12
2.39
2.52
3.93
1.99
6.43
6.79
6.56
6.01
6.50
Se
Se
P
O
P
O

^{13}C NMR in $CDCl_3$

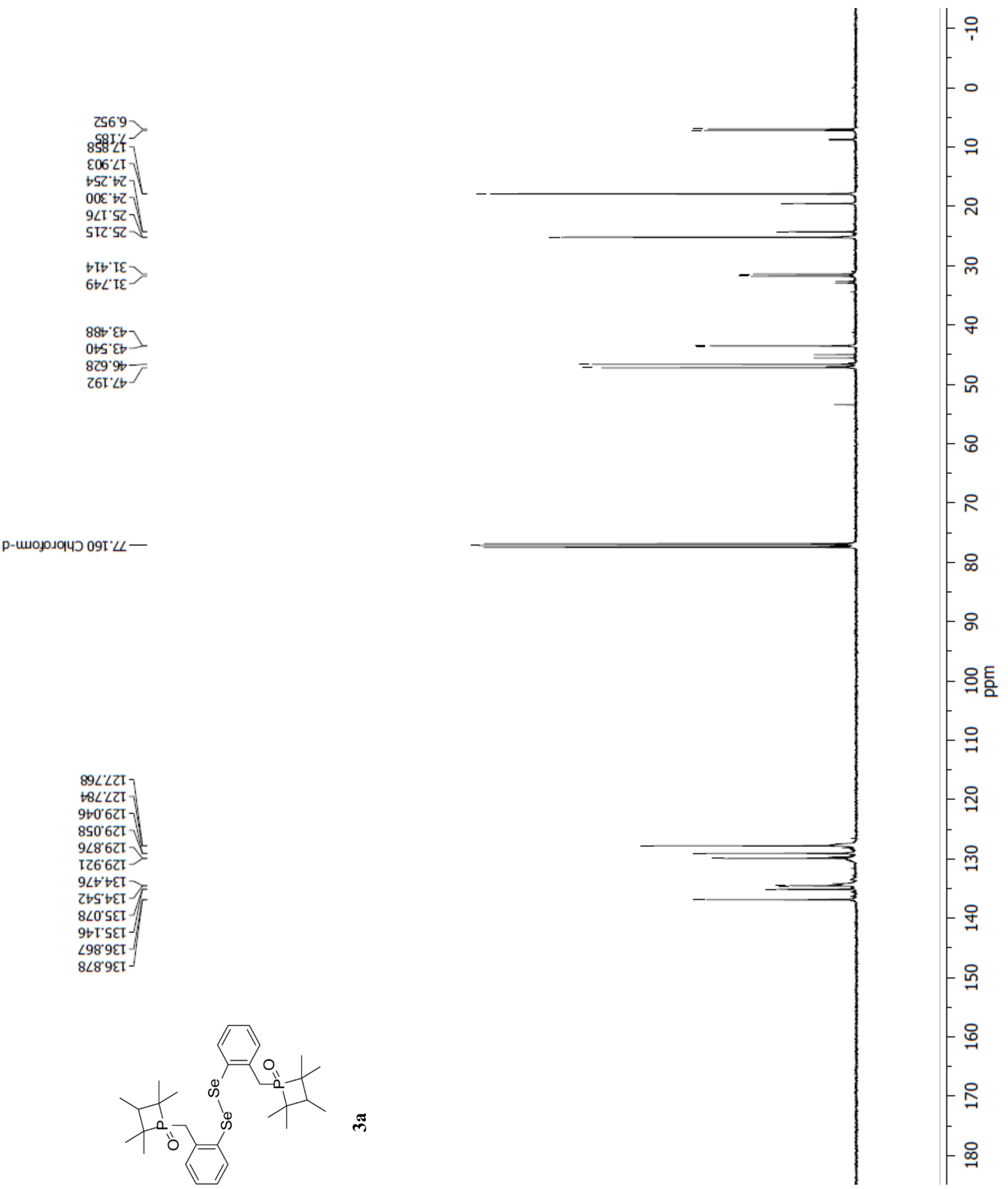

3a

^{31}P NMR in $CDCl_3$

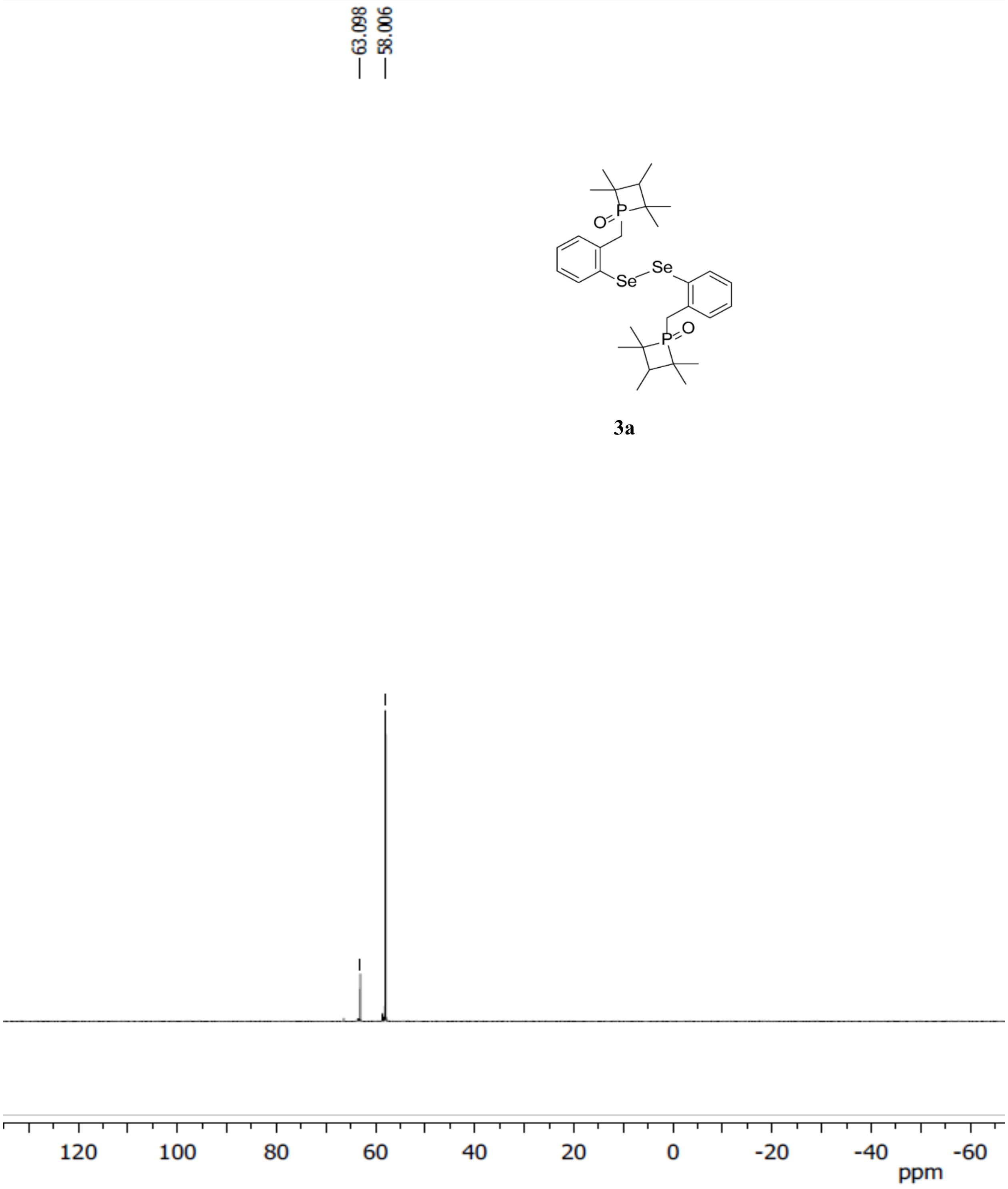

3a

^{77}Se NMR in $CDCl_3$

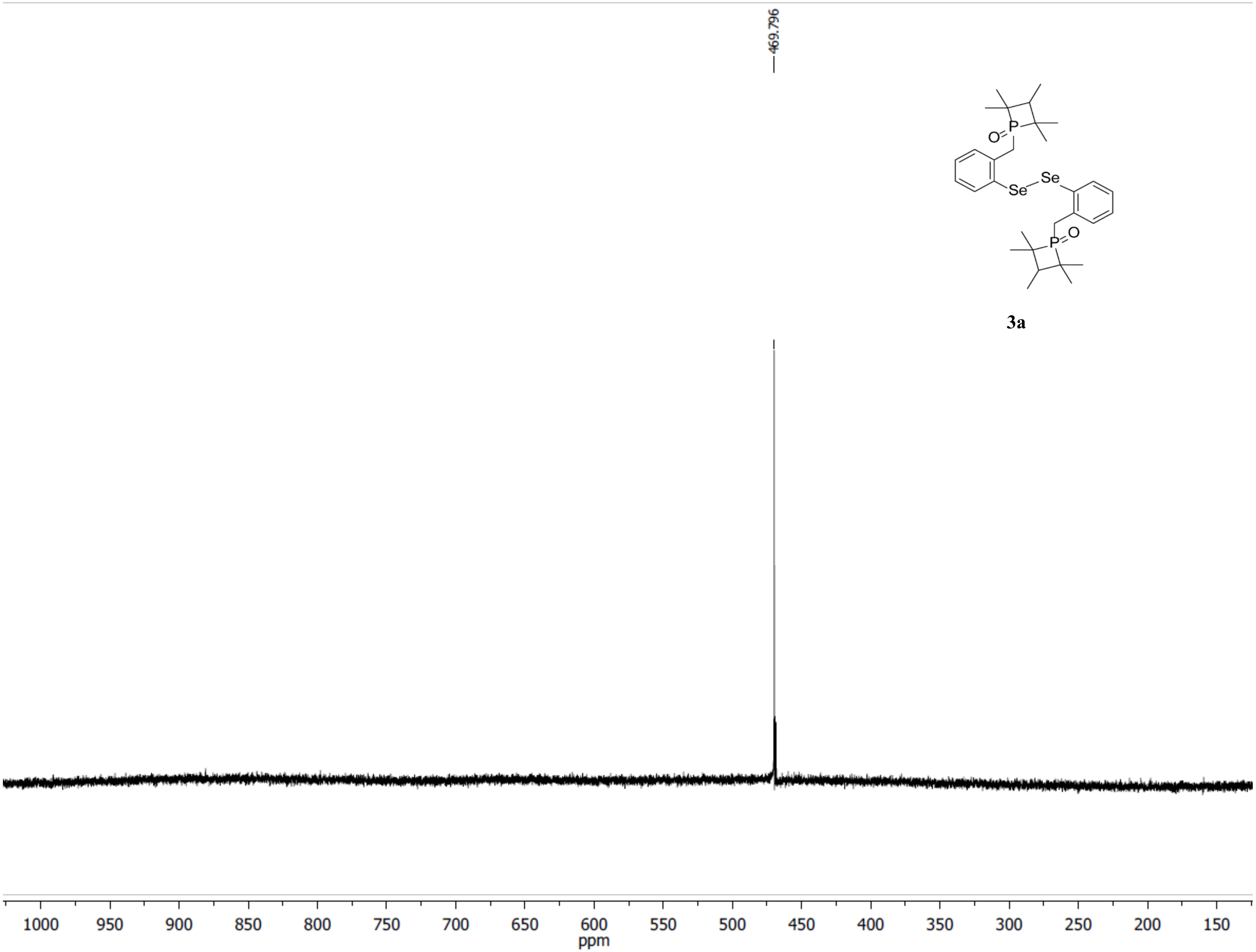

3a

^{1}H NMR in $CDCl_3$

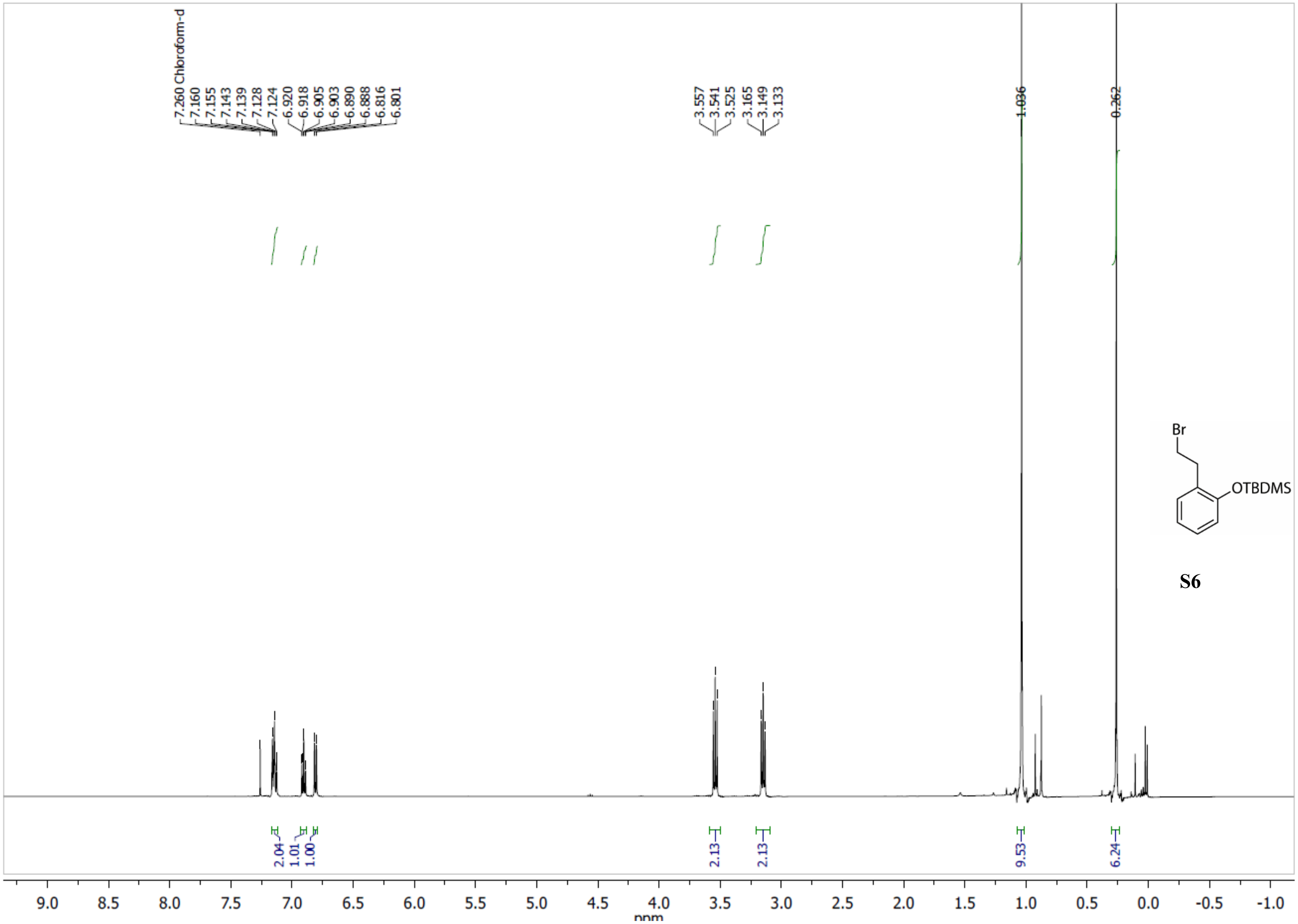

^{13}C NMR in $CDCl_3$

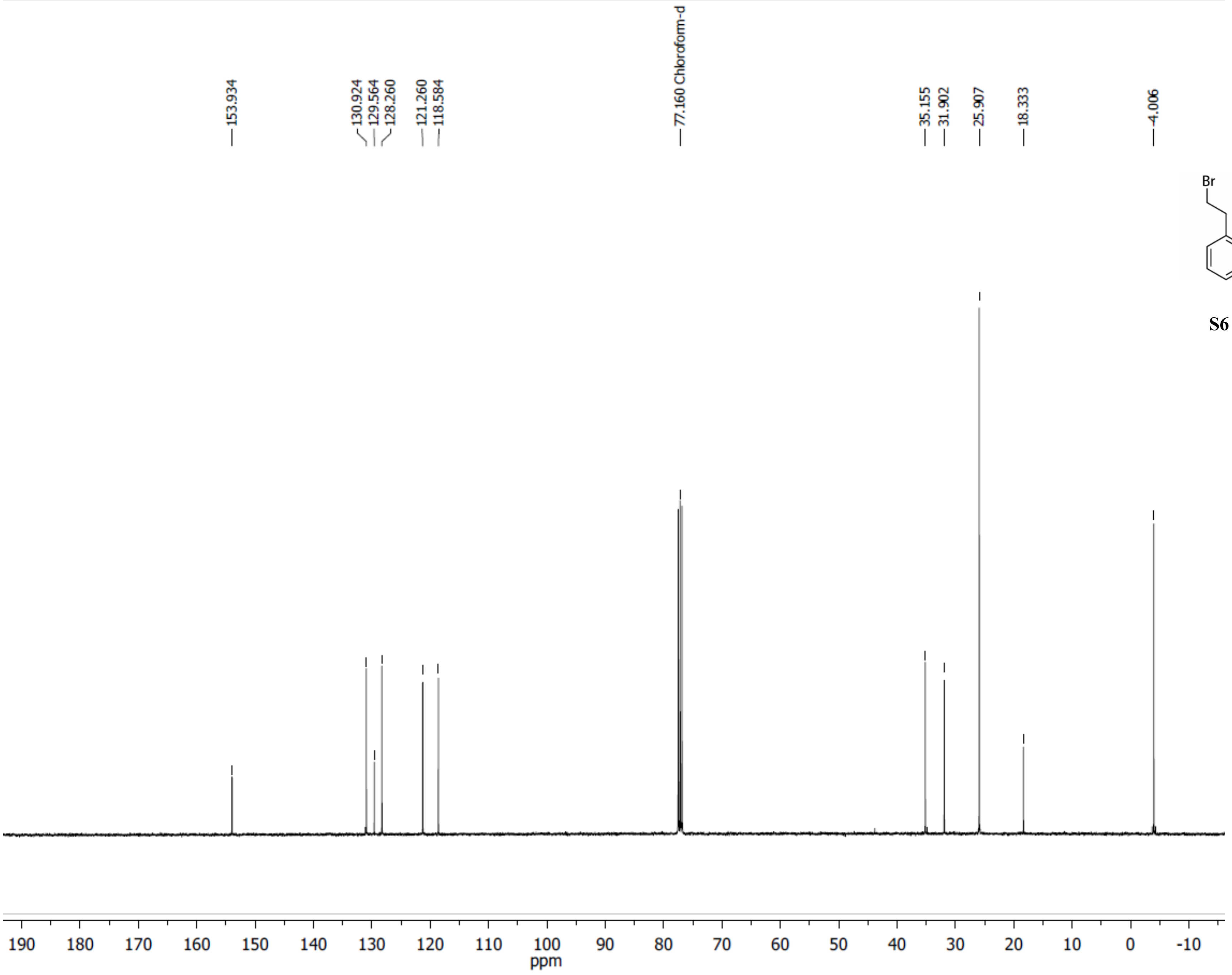

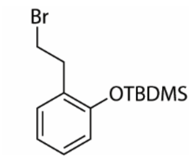

S6

^{1}H NMR in $CDCl_3$

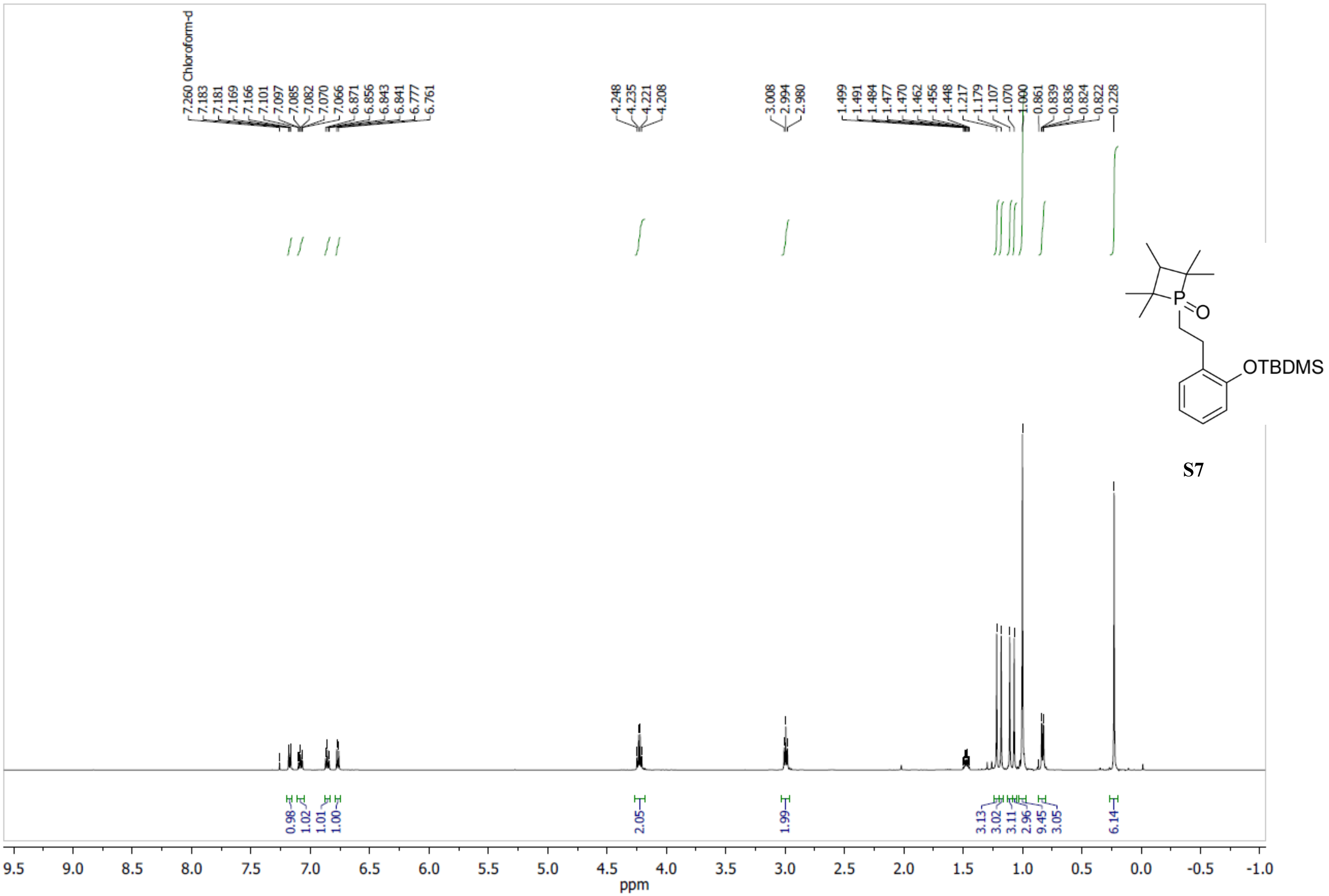

S7

^{13}C NMR in $CDCl_3$

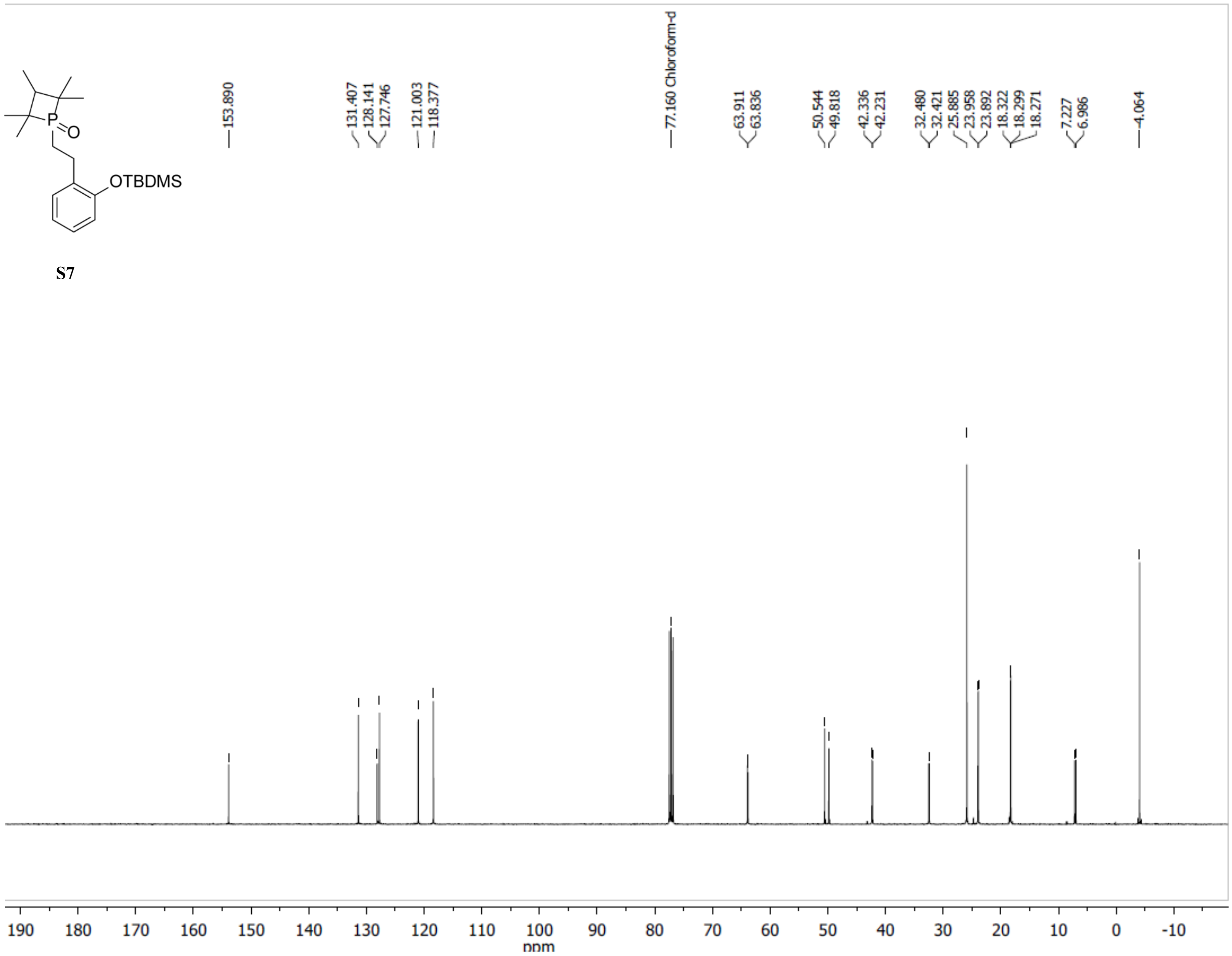

S7

^{31}P NMR in $CDCl_3$

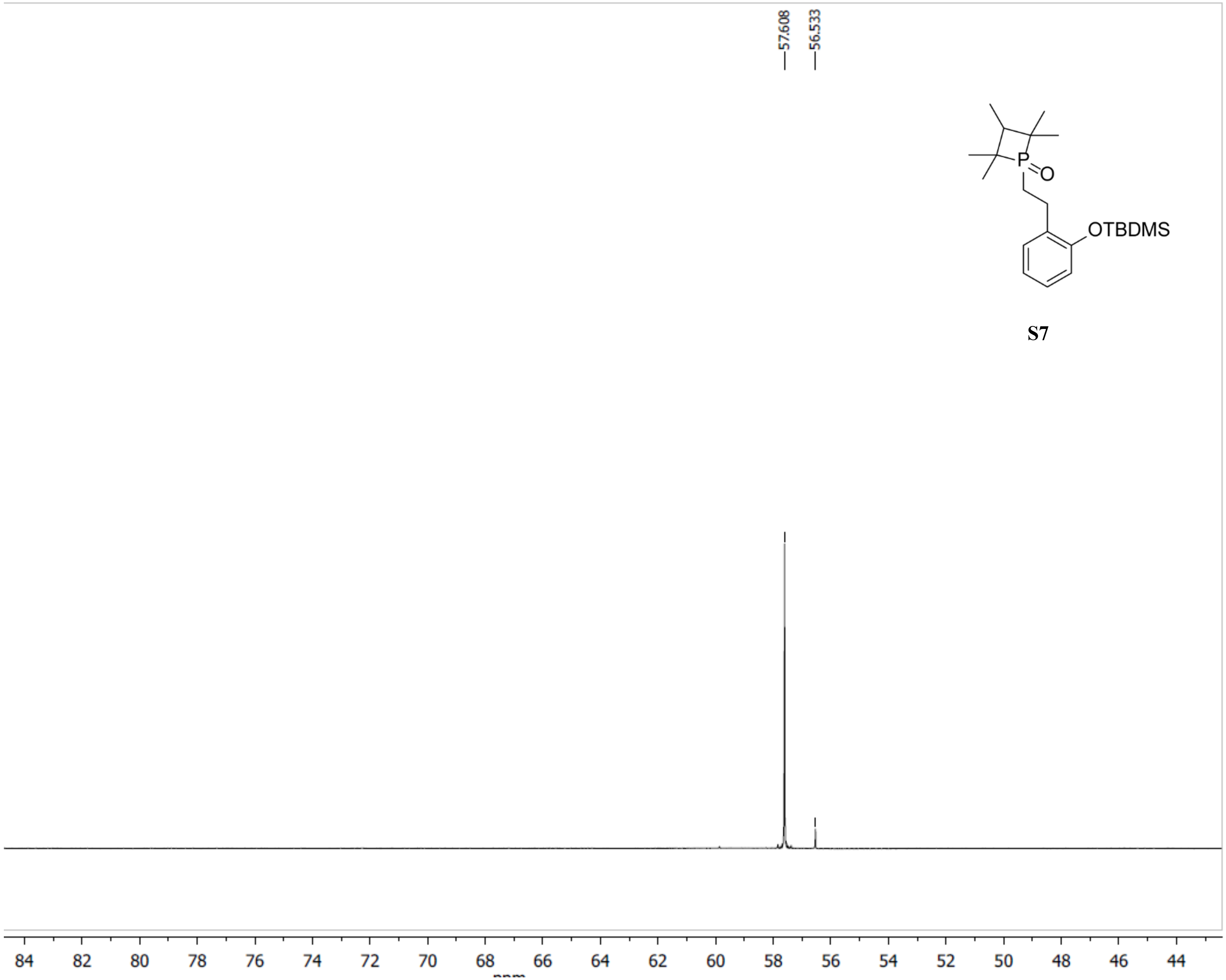

S7

^1H NMR in $CDCl_3$

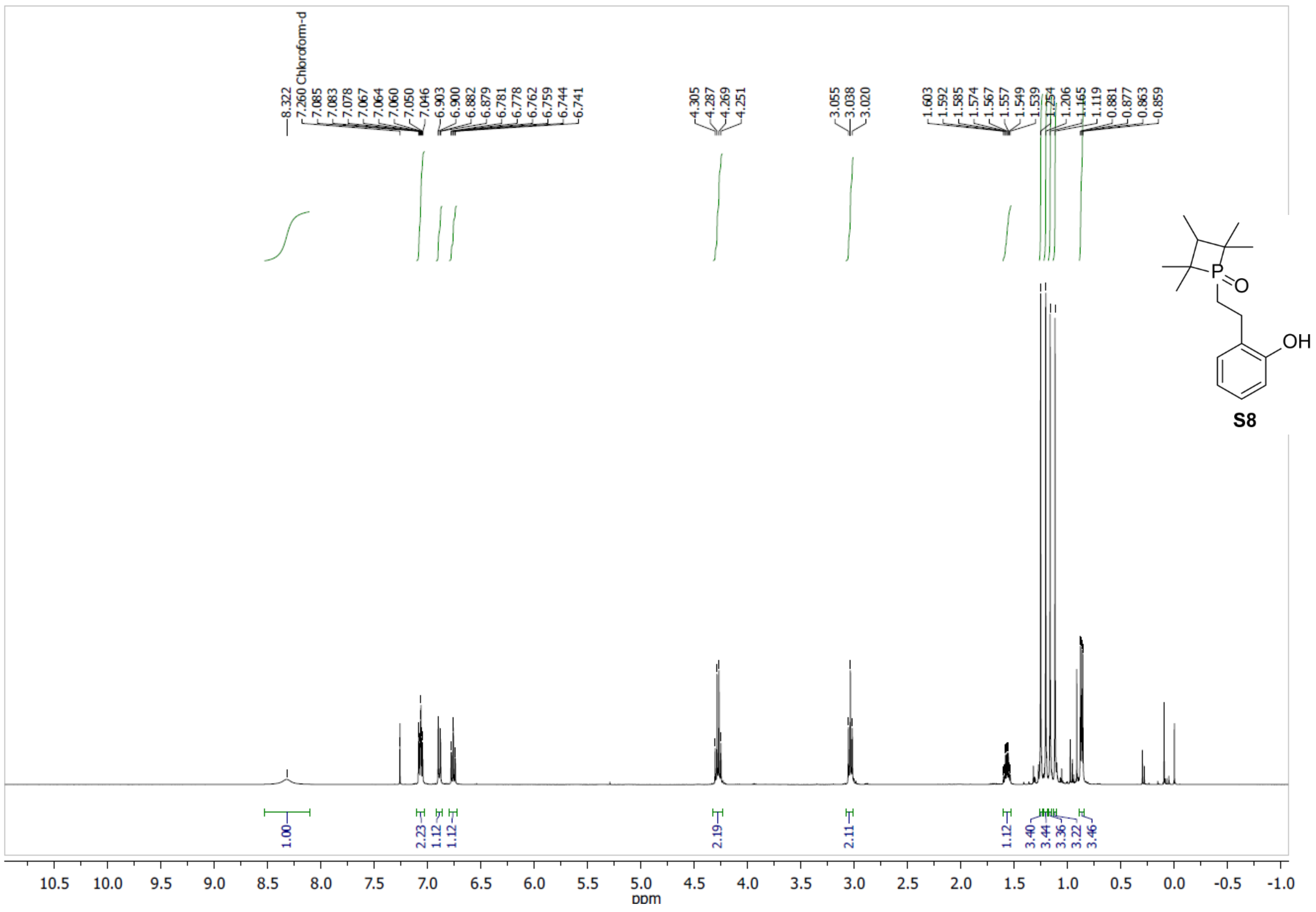

^{13}C NMR in $CDCl_3$

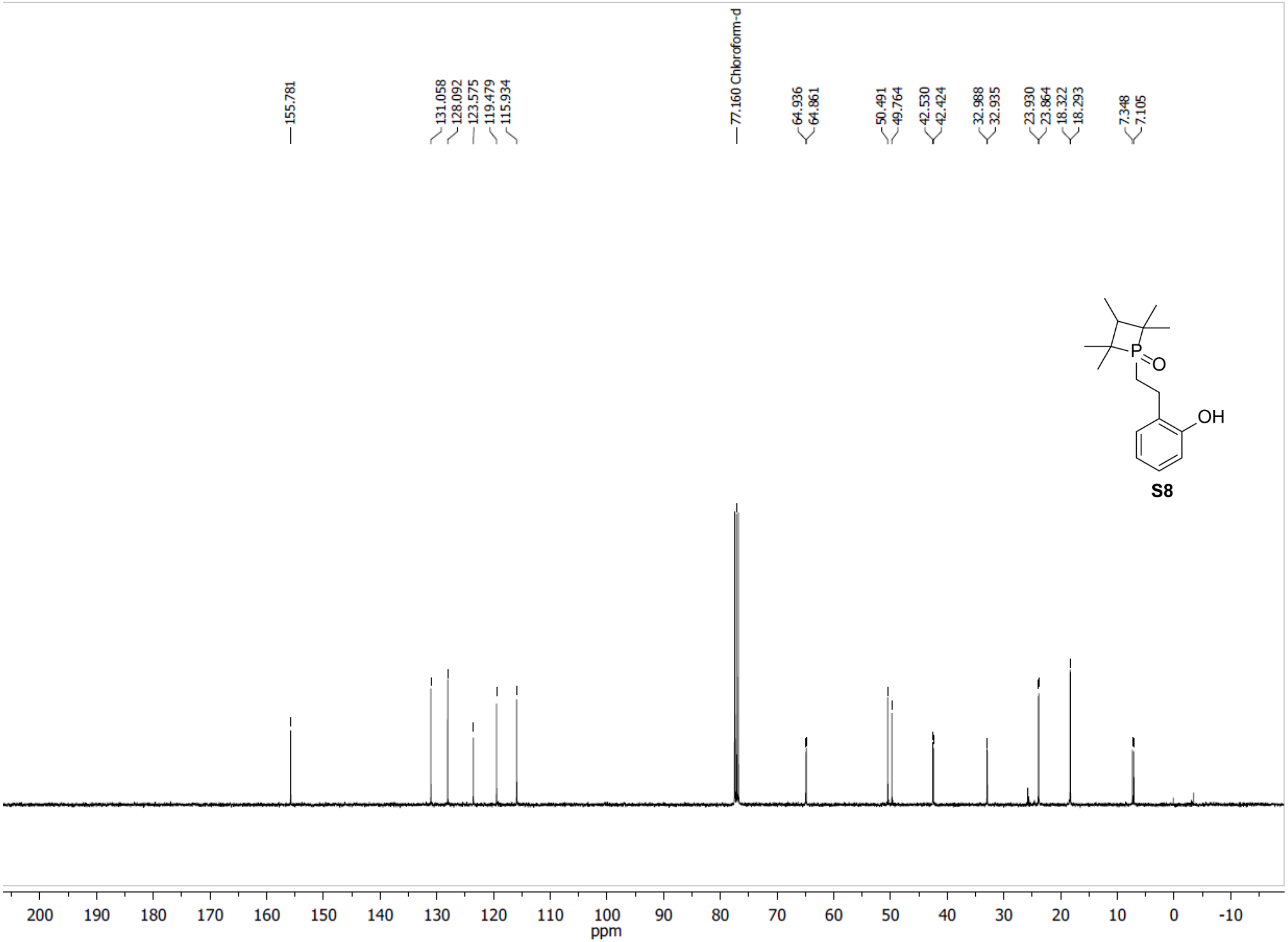

^{31}P NMR in $CDCl_3$

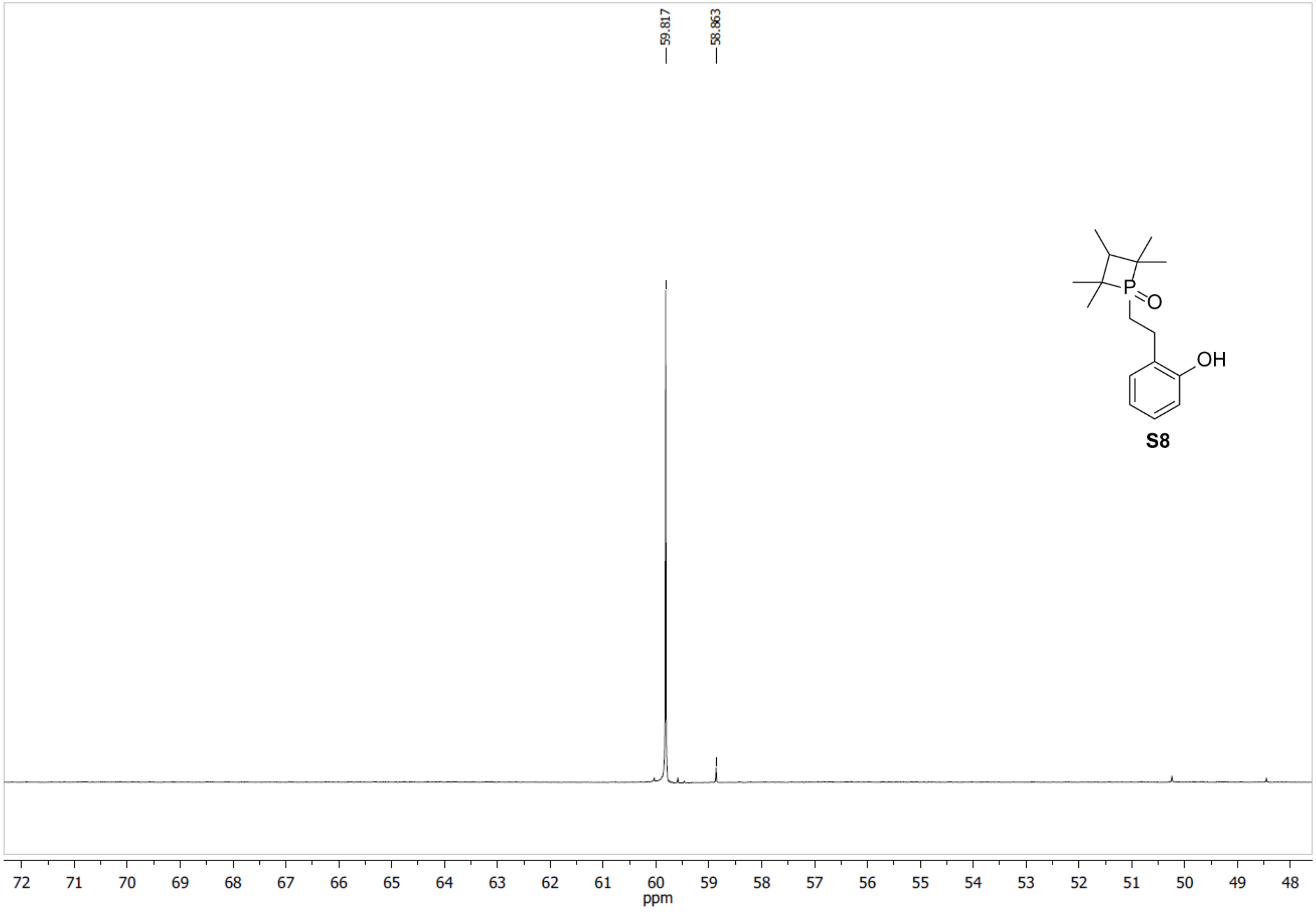

S8

^{1}H NMR in $CDCl_3$

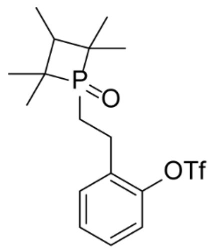

S9

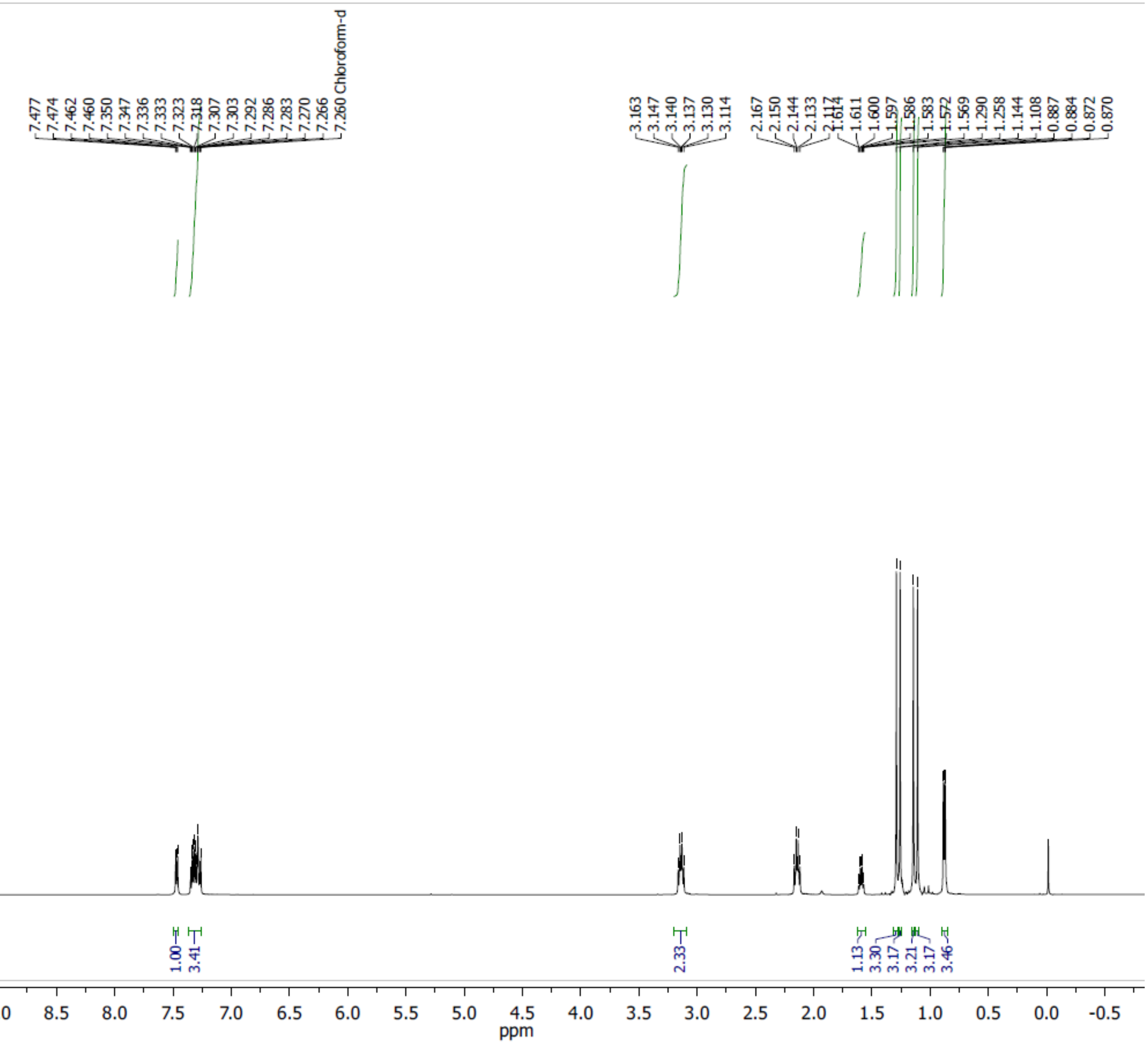

^{13}C NMR in $CDCl_3$

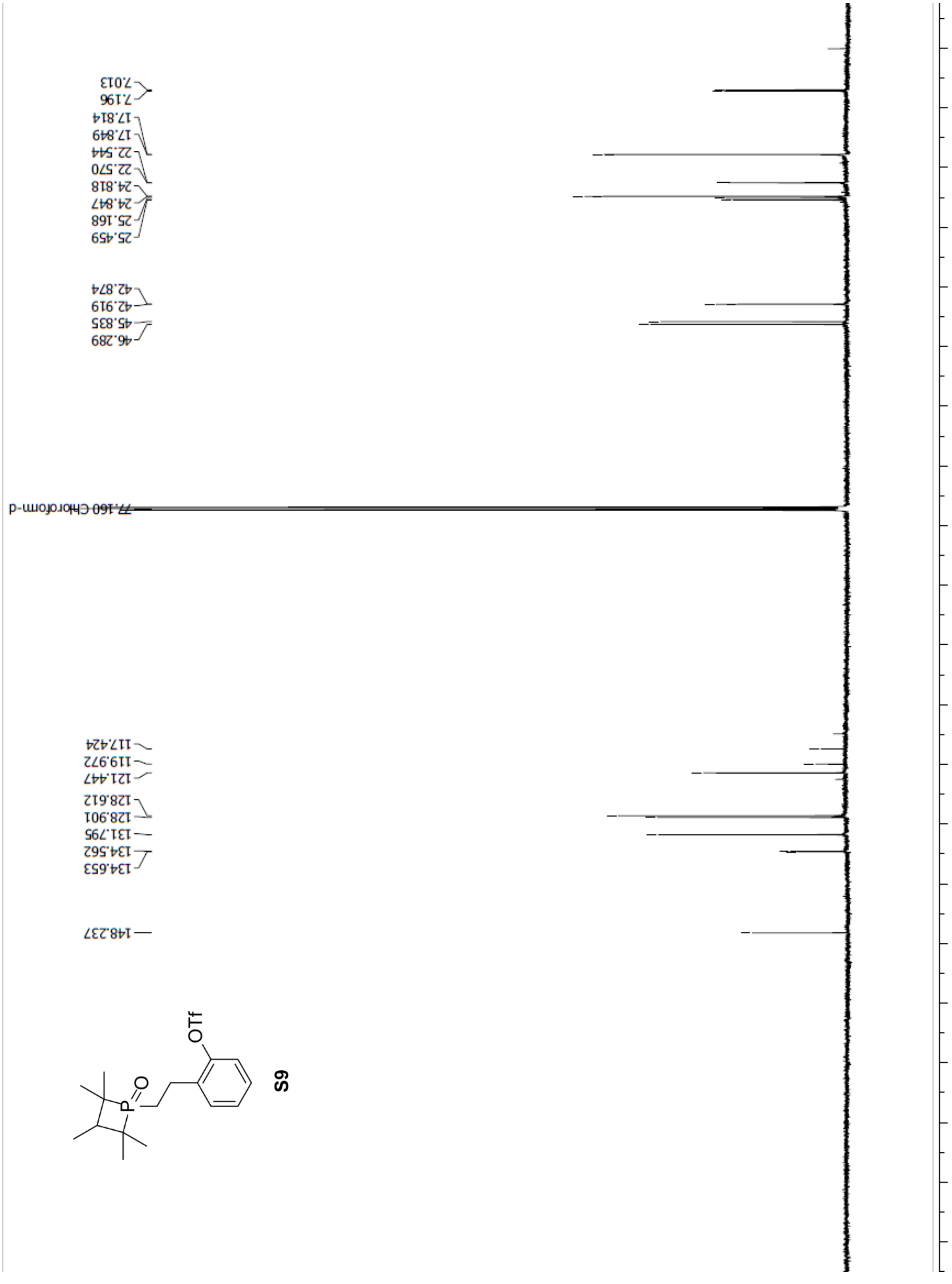

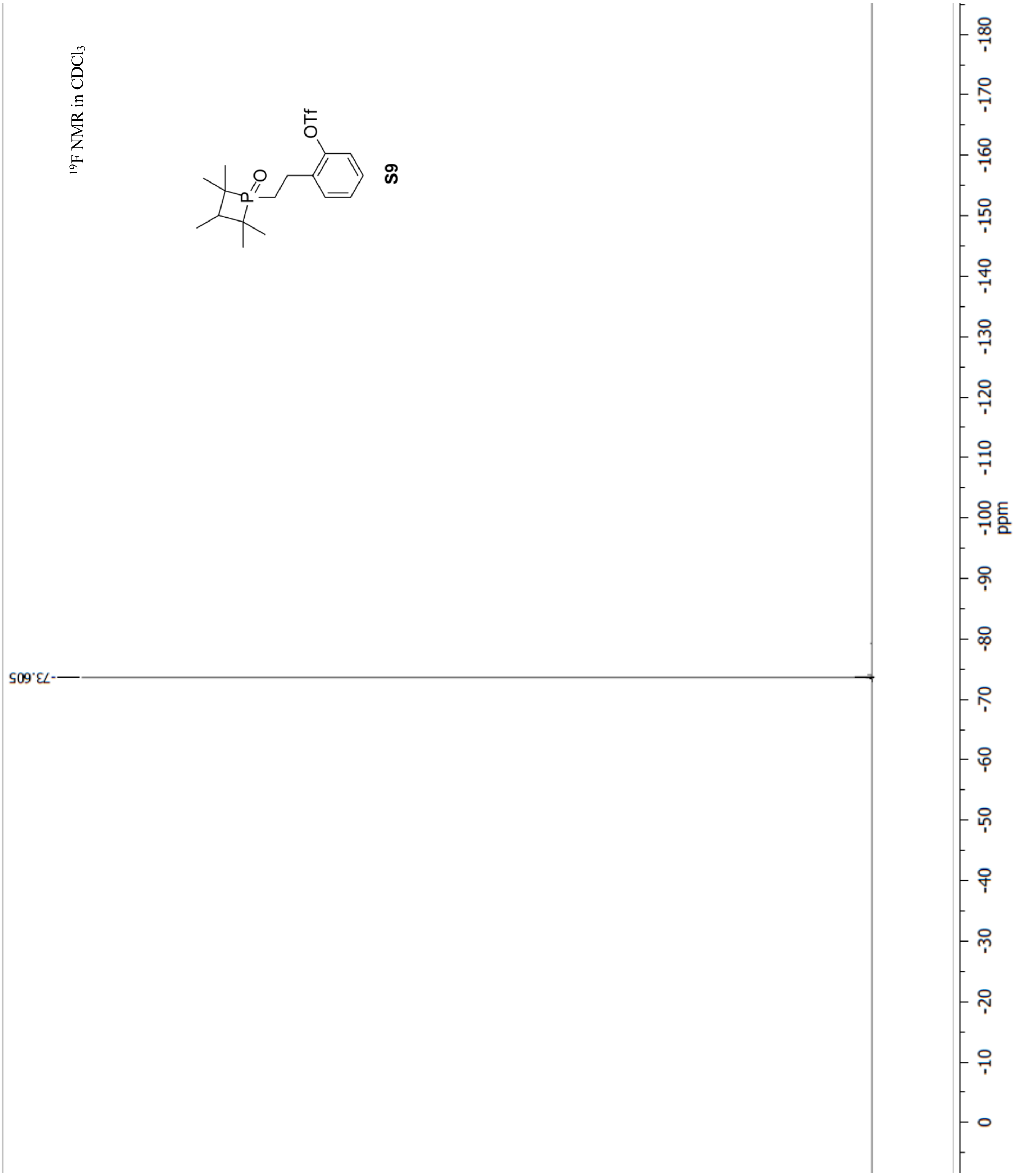
^{19}F NMR in CDCl$_3$
P=O
OTf
S9
-73.605
-180 -170 -160 -150 -140 -130 -120 -110 -100 -90 -80 -70 -60 -50 -40 -30 -20 -10 0
ppm

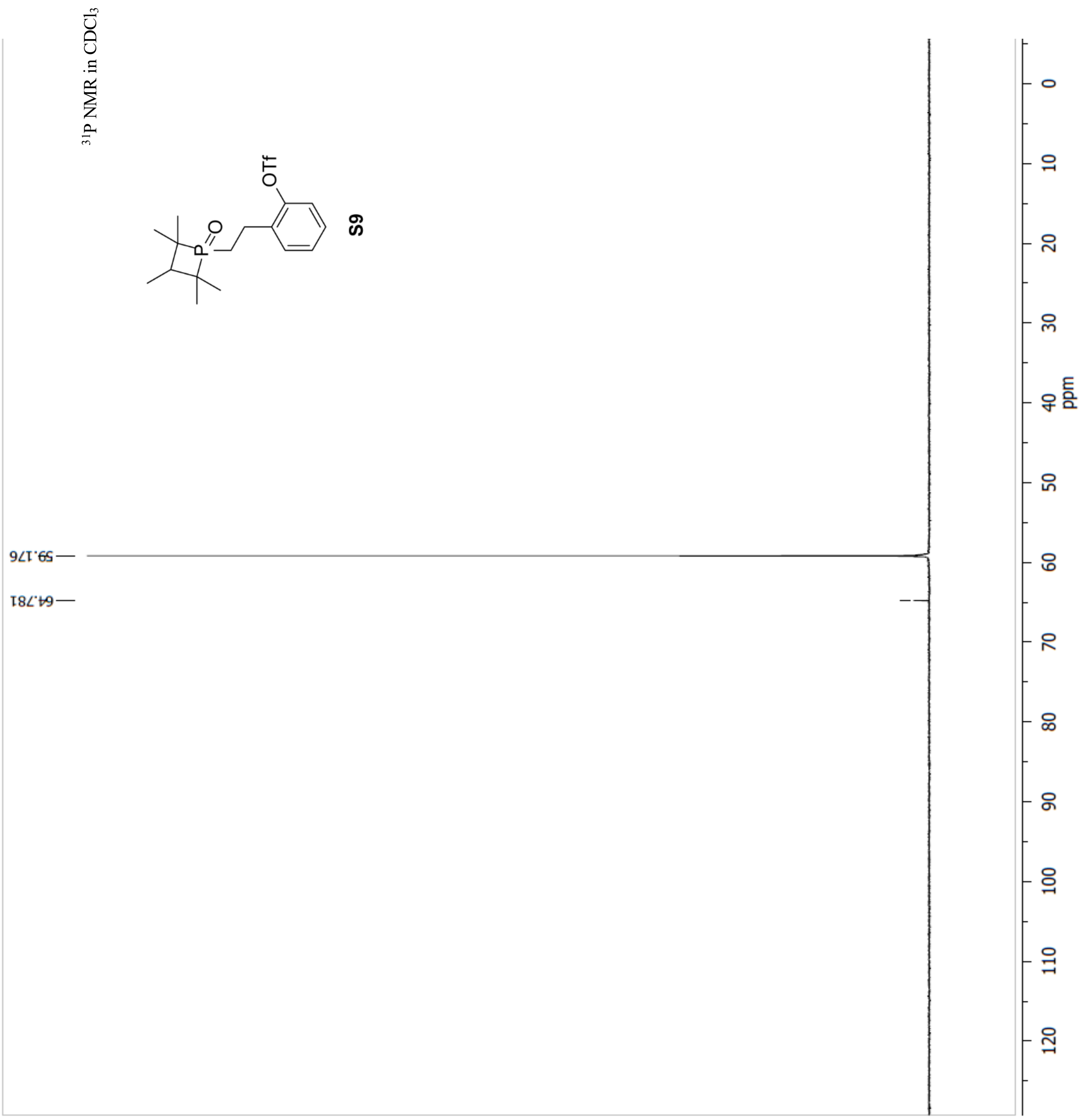

^{31}P NMR in CDCl$_3$
OTf
S9
59.176
64.781
ppm

^{1}H NMR in $CDCl_3$

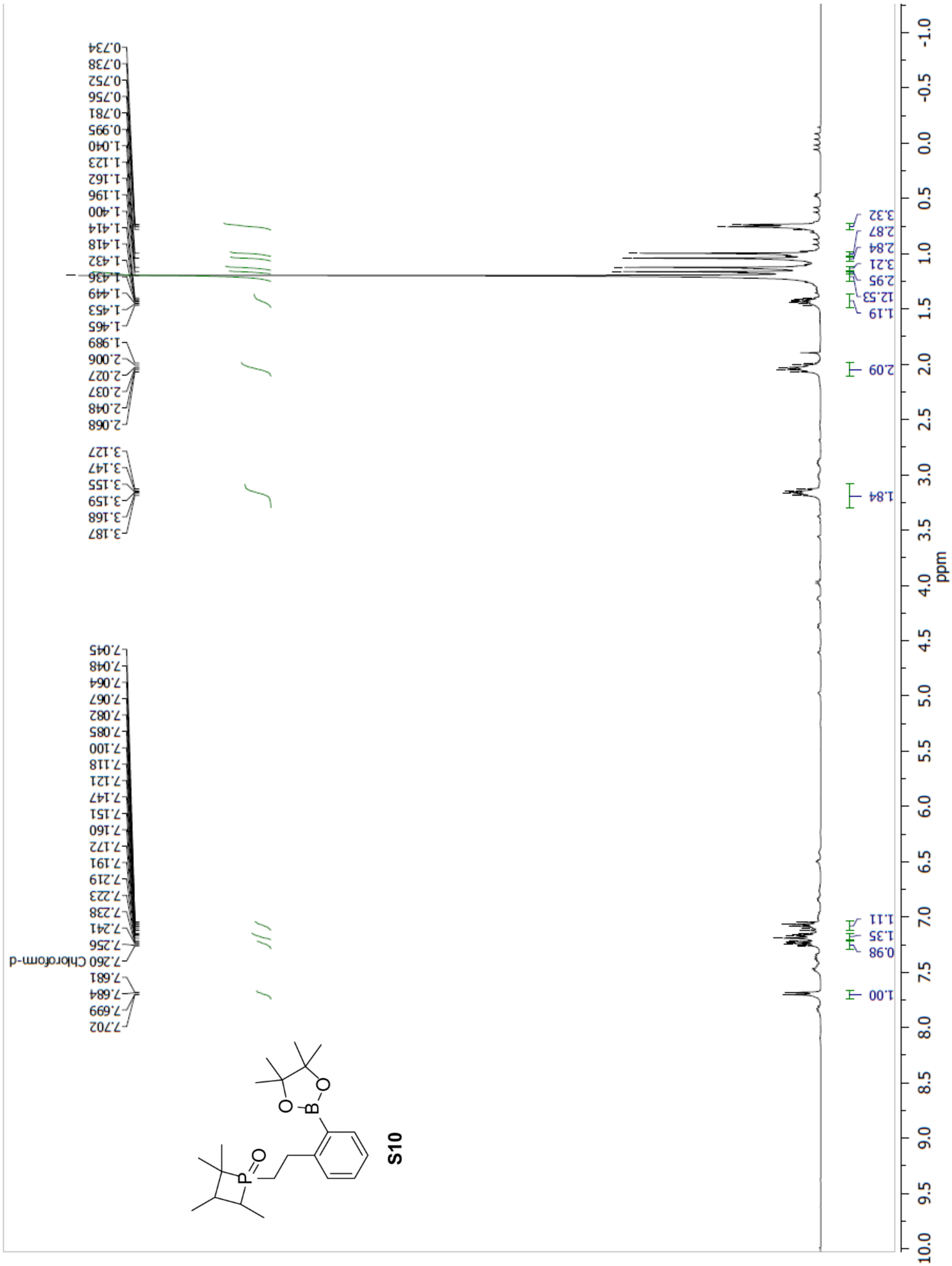

^{13}C NMR in $CDCl_3$

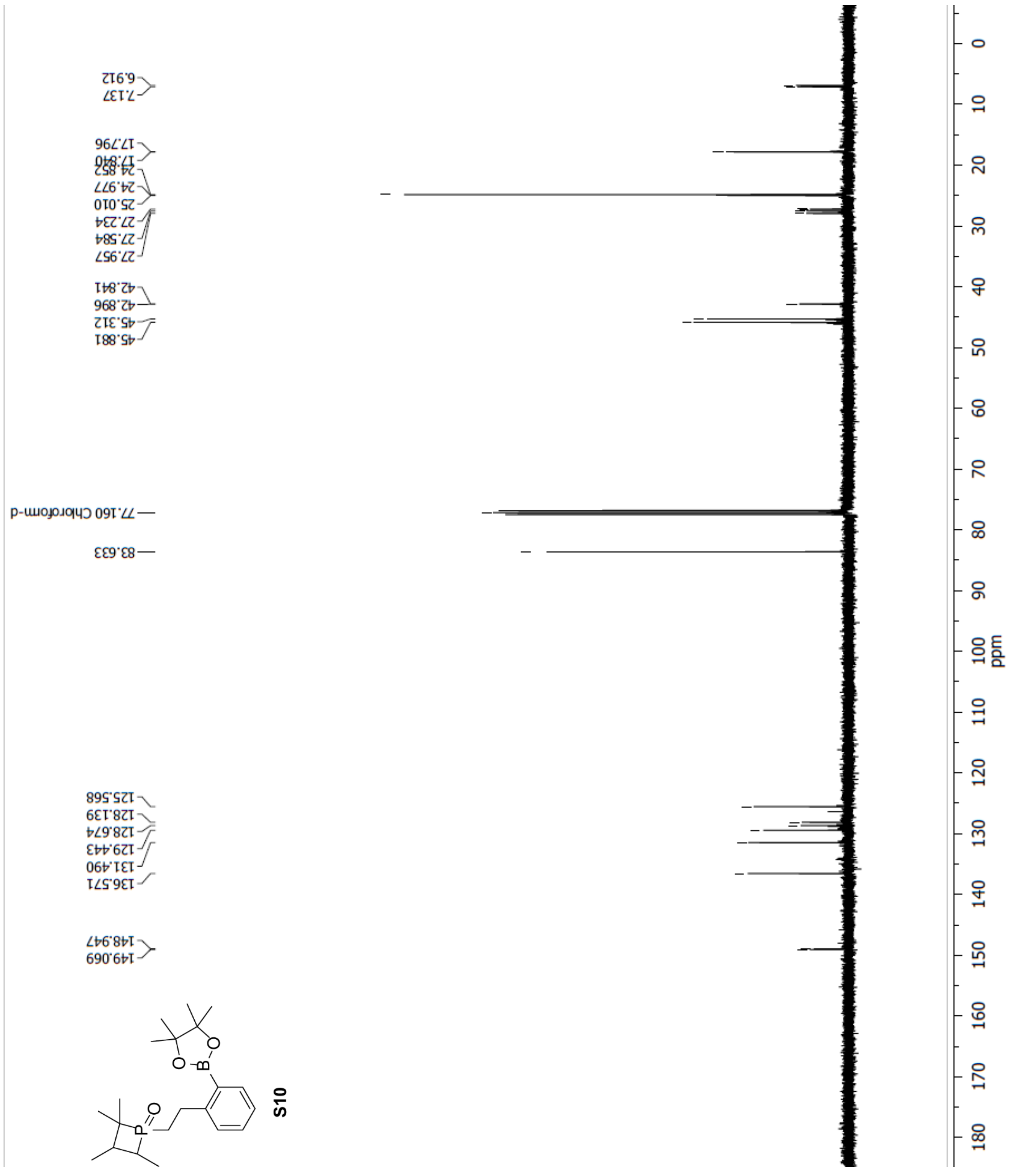

S10

^{31}P NMR in $CDCl_3$

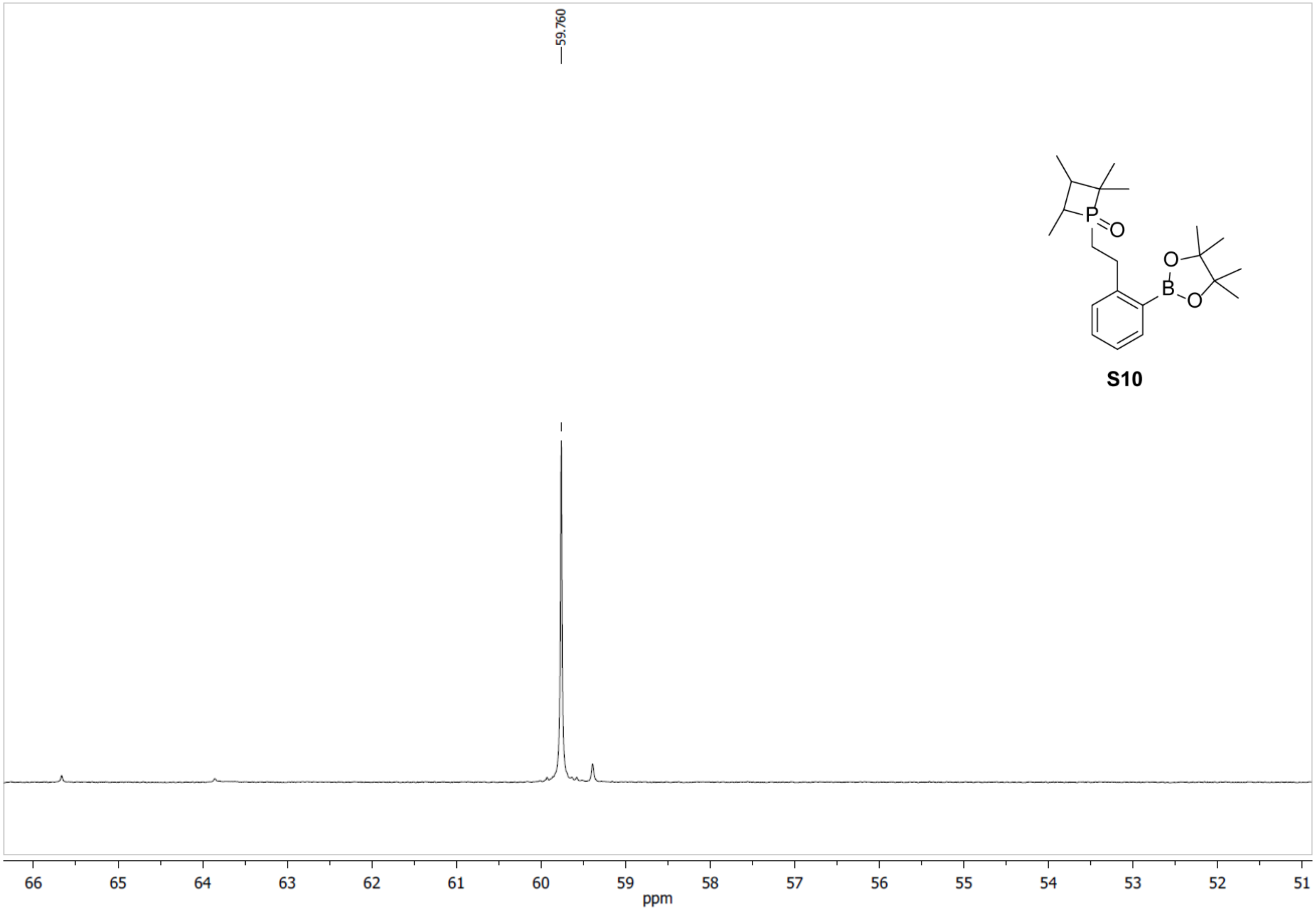

^{1}H NMR in $CDCl_3$

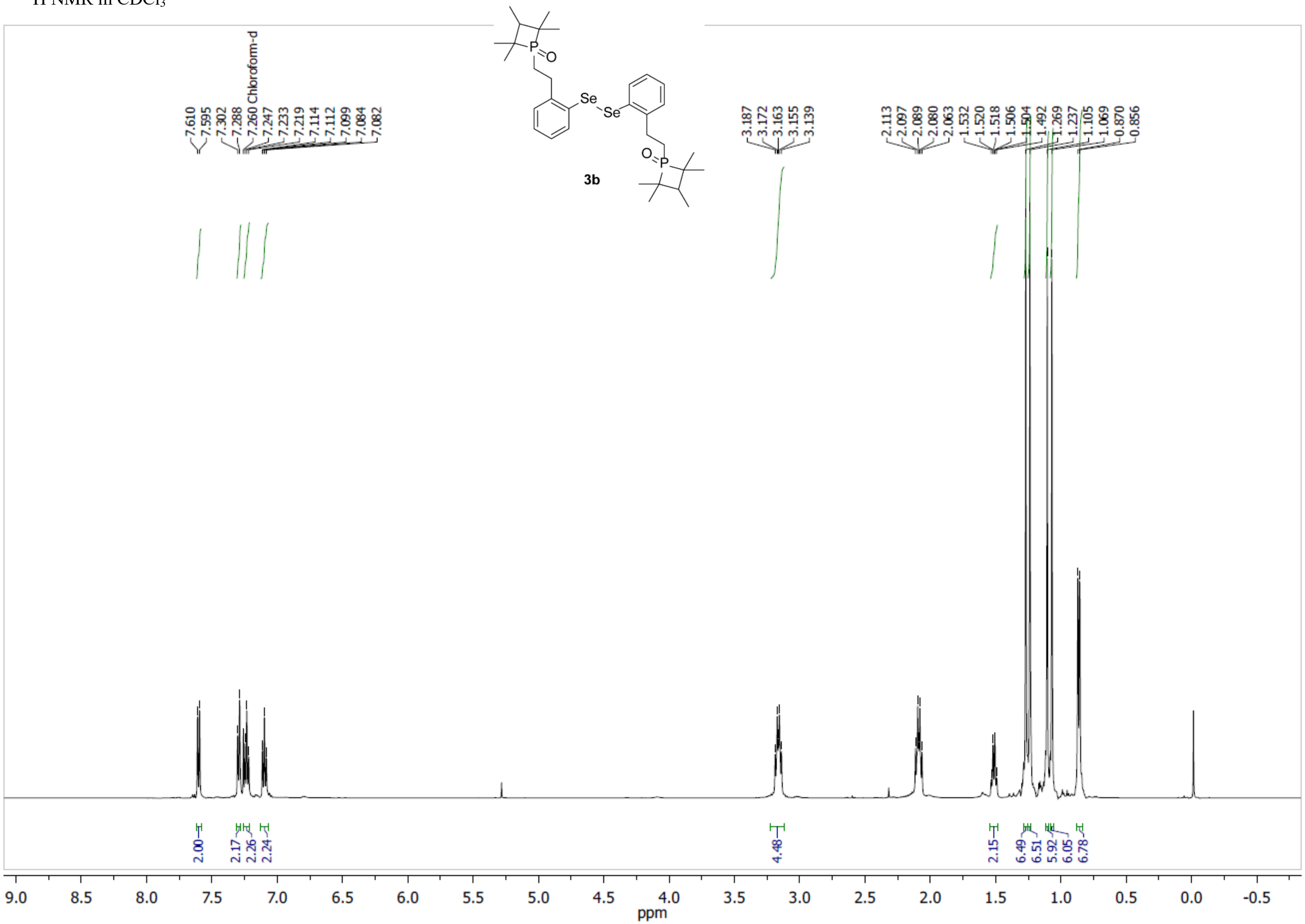

^{13}C NMR in $CDCl_3$

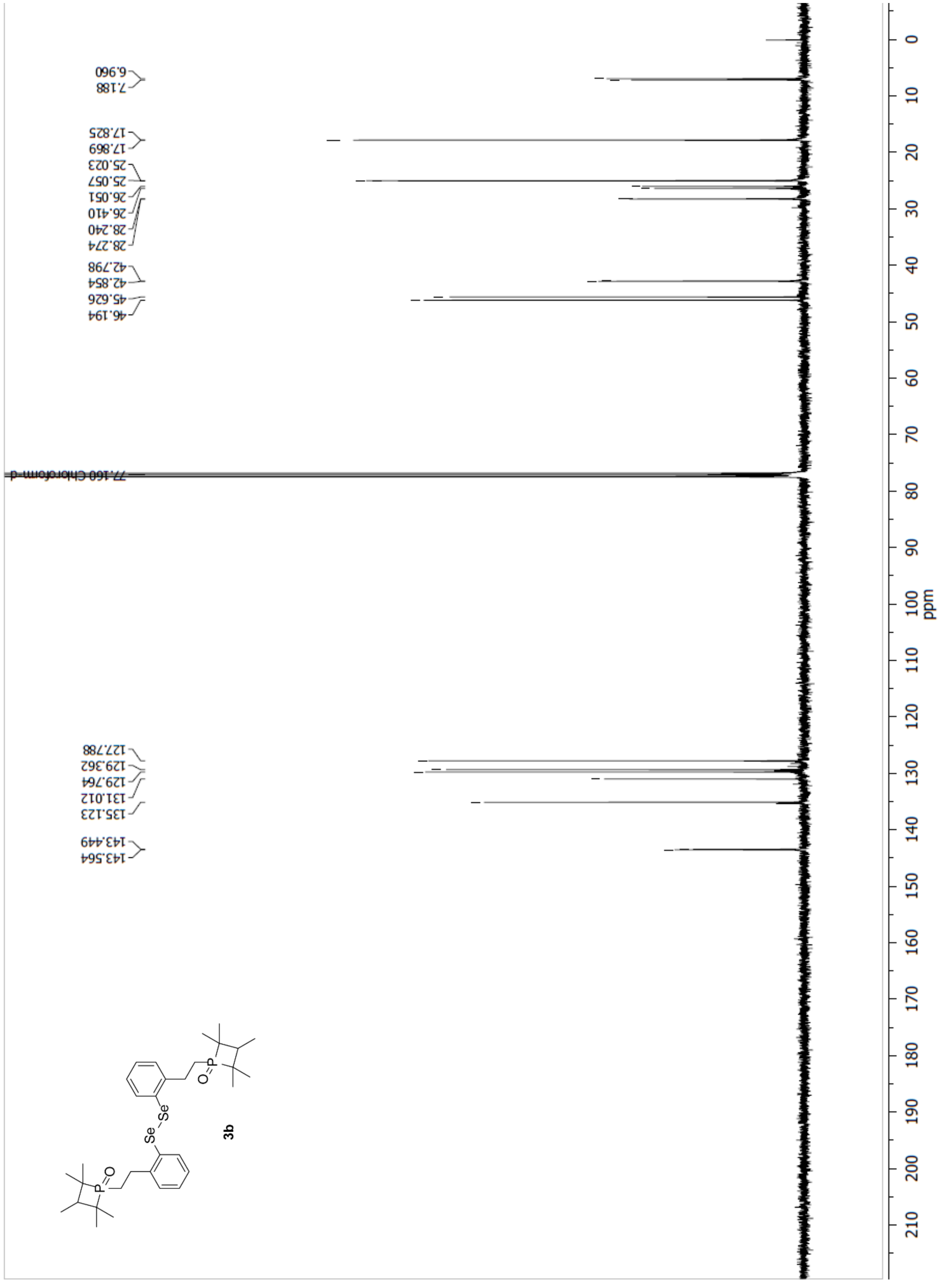

^{31}P NMR in $CDCl_3$

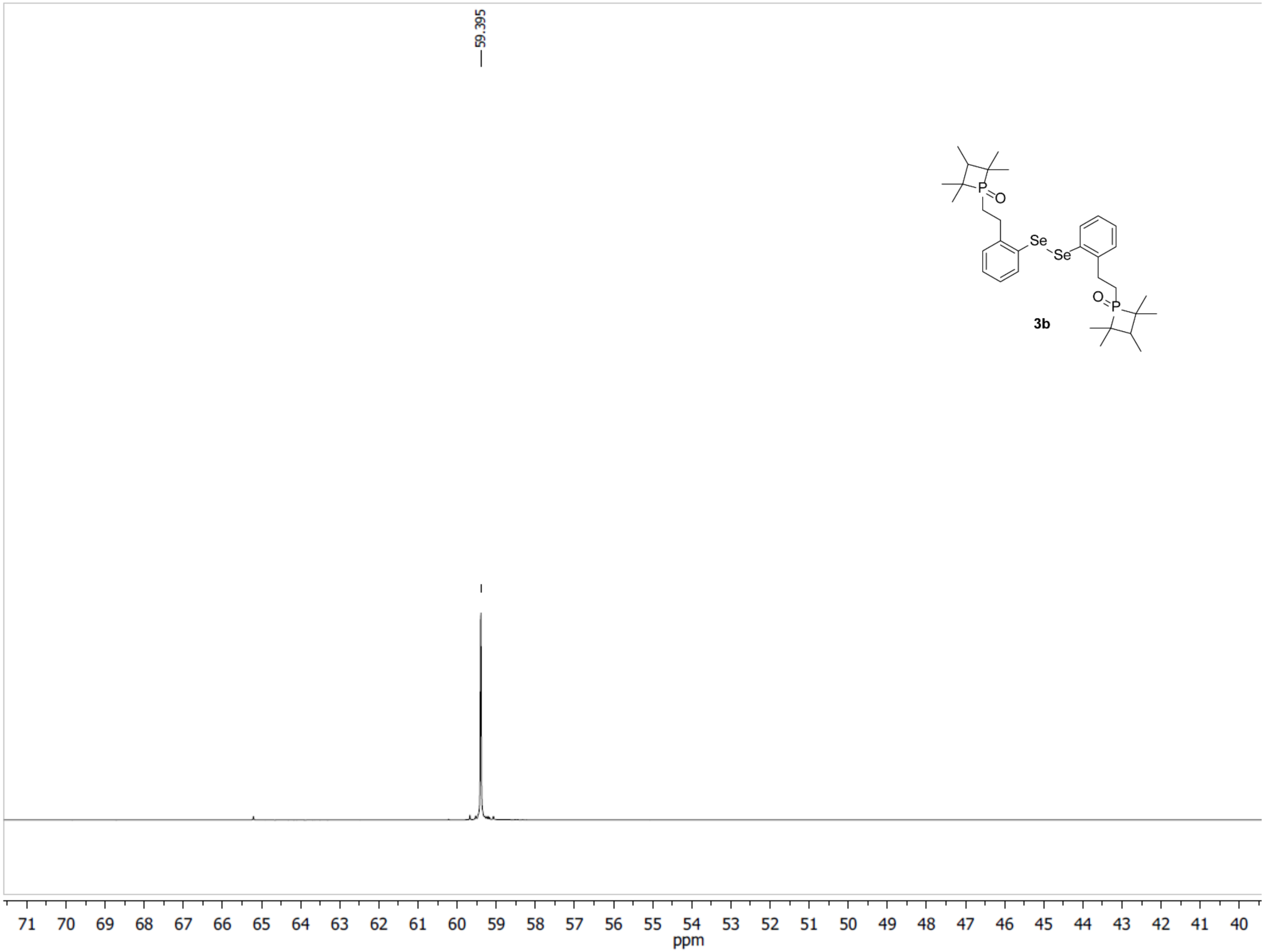

^{77}Se NMR in $CDCl_3$

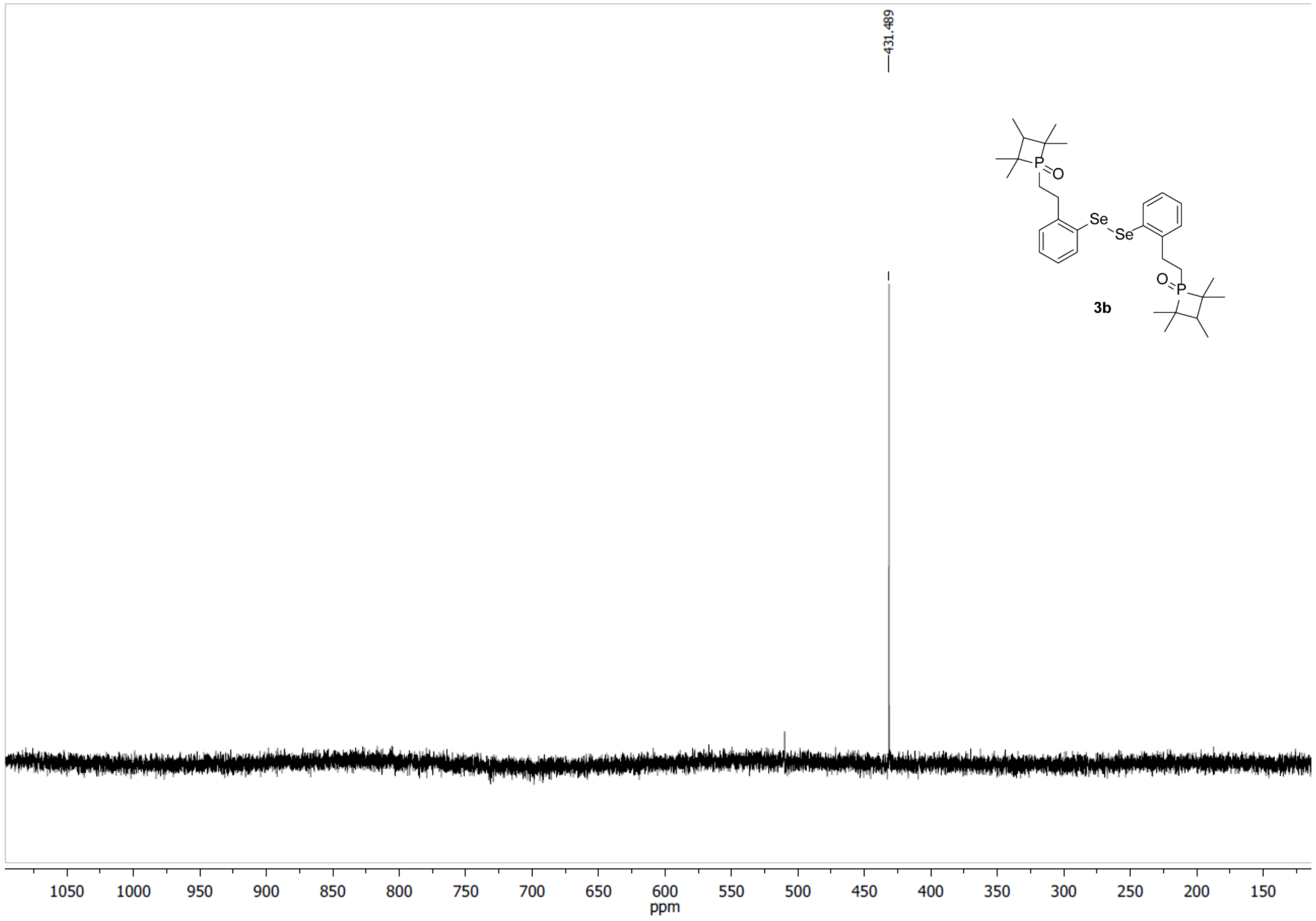

^{1}H NMR in CDCl$_3$

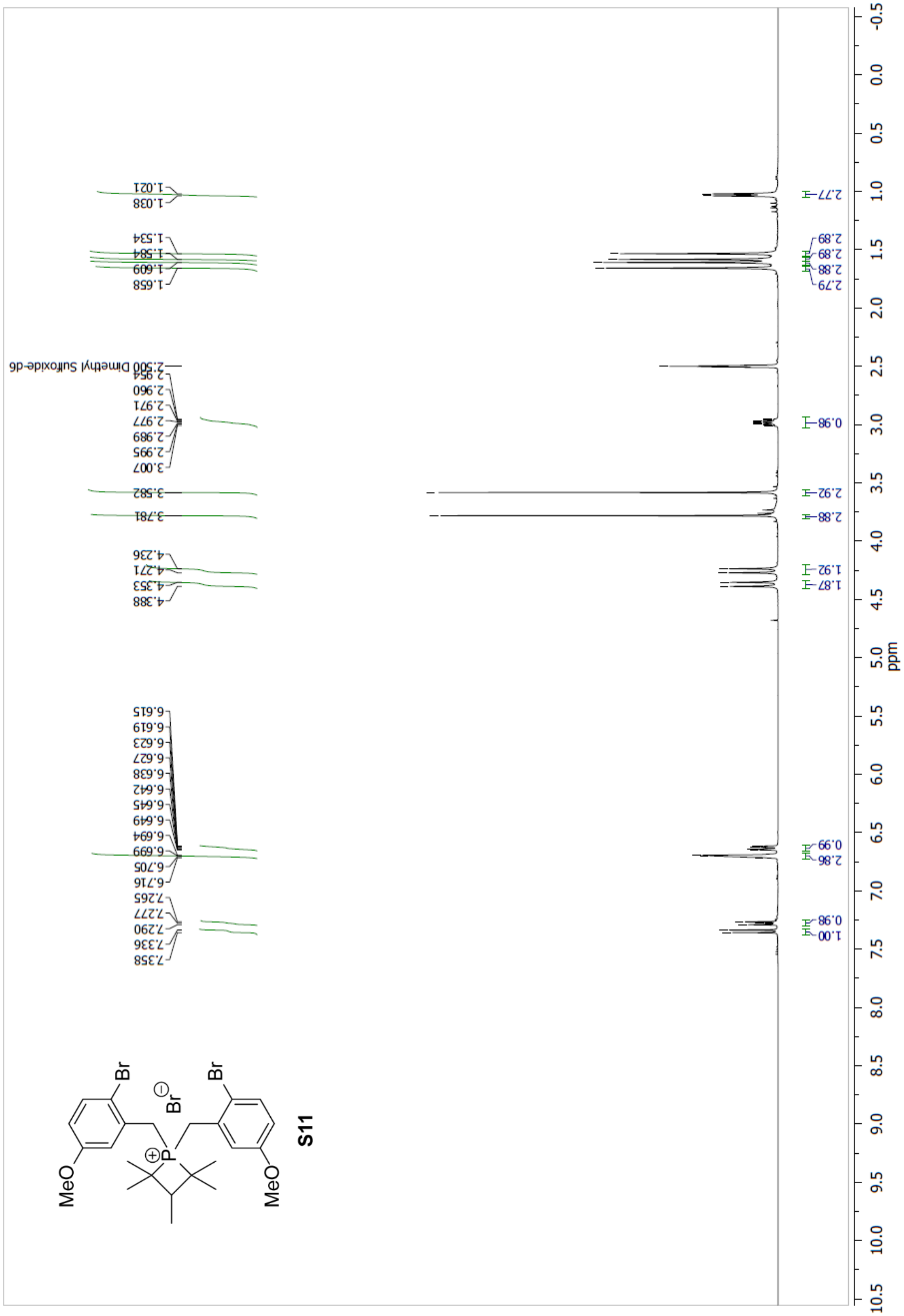

^{13}C NMR in $CDCl_3$

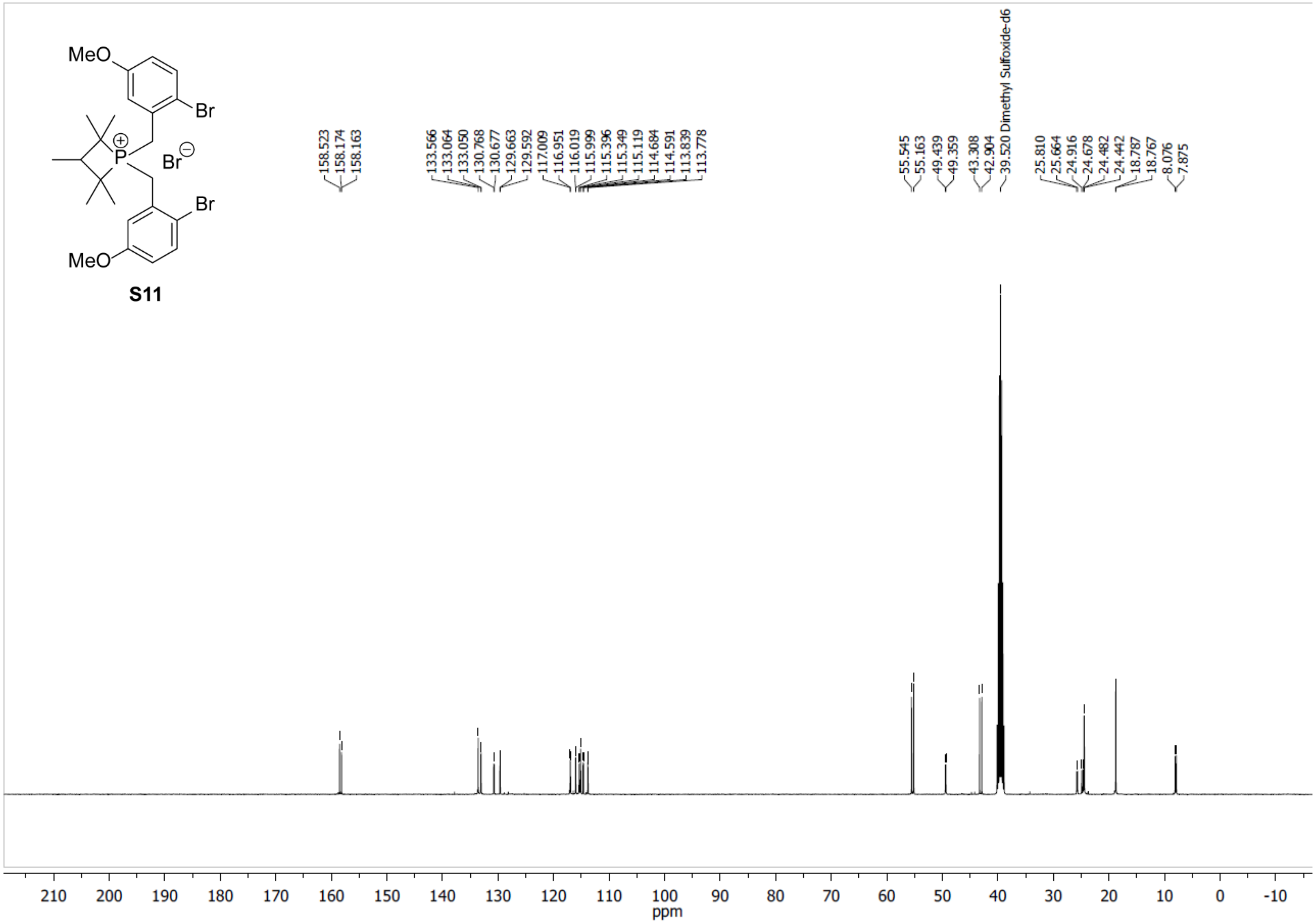

^{31}P NMR in $CDCl_3$

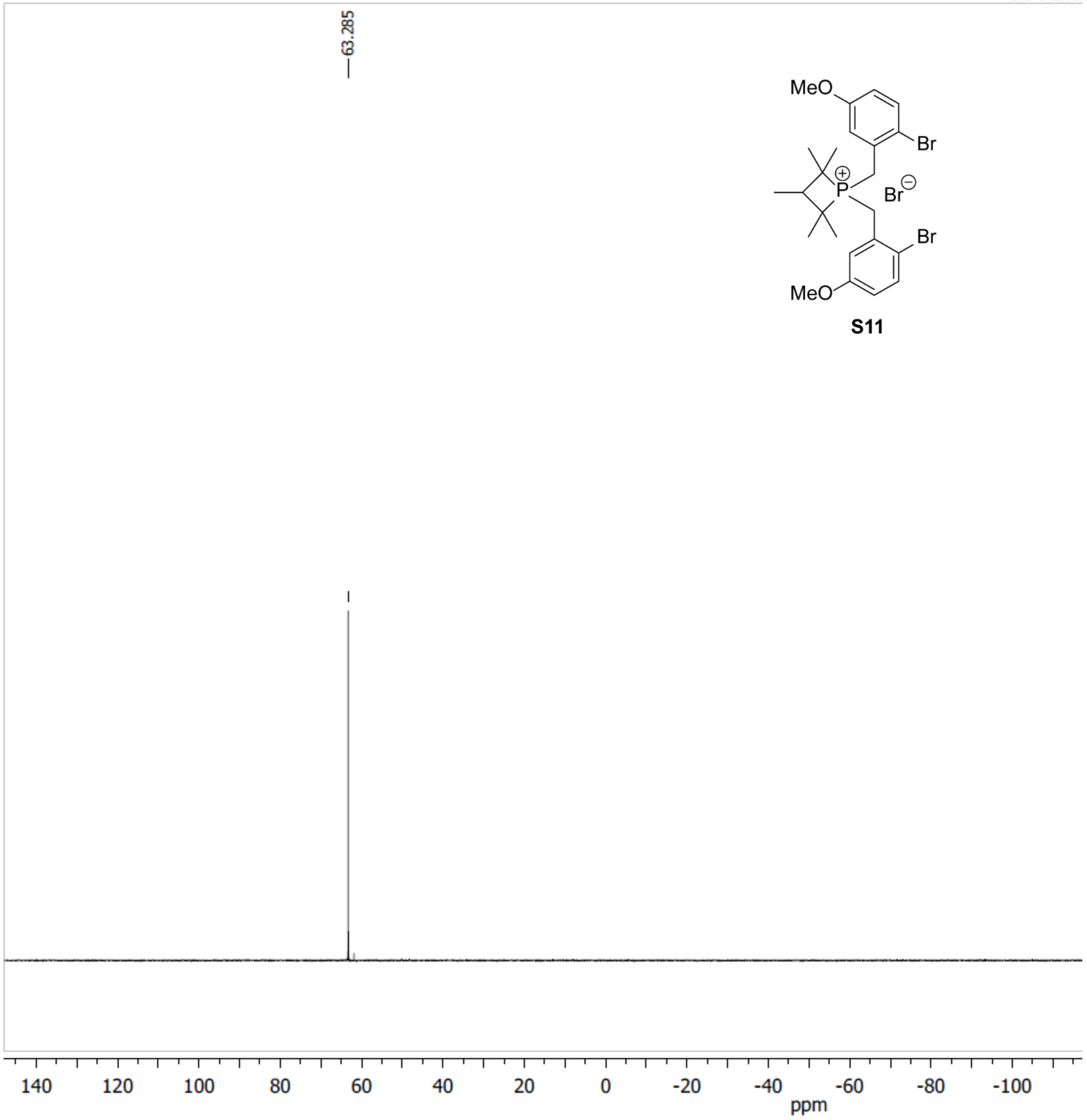

^{1}H NMR in $CDCl_3$

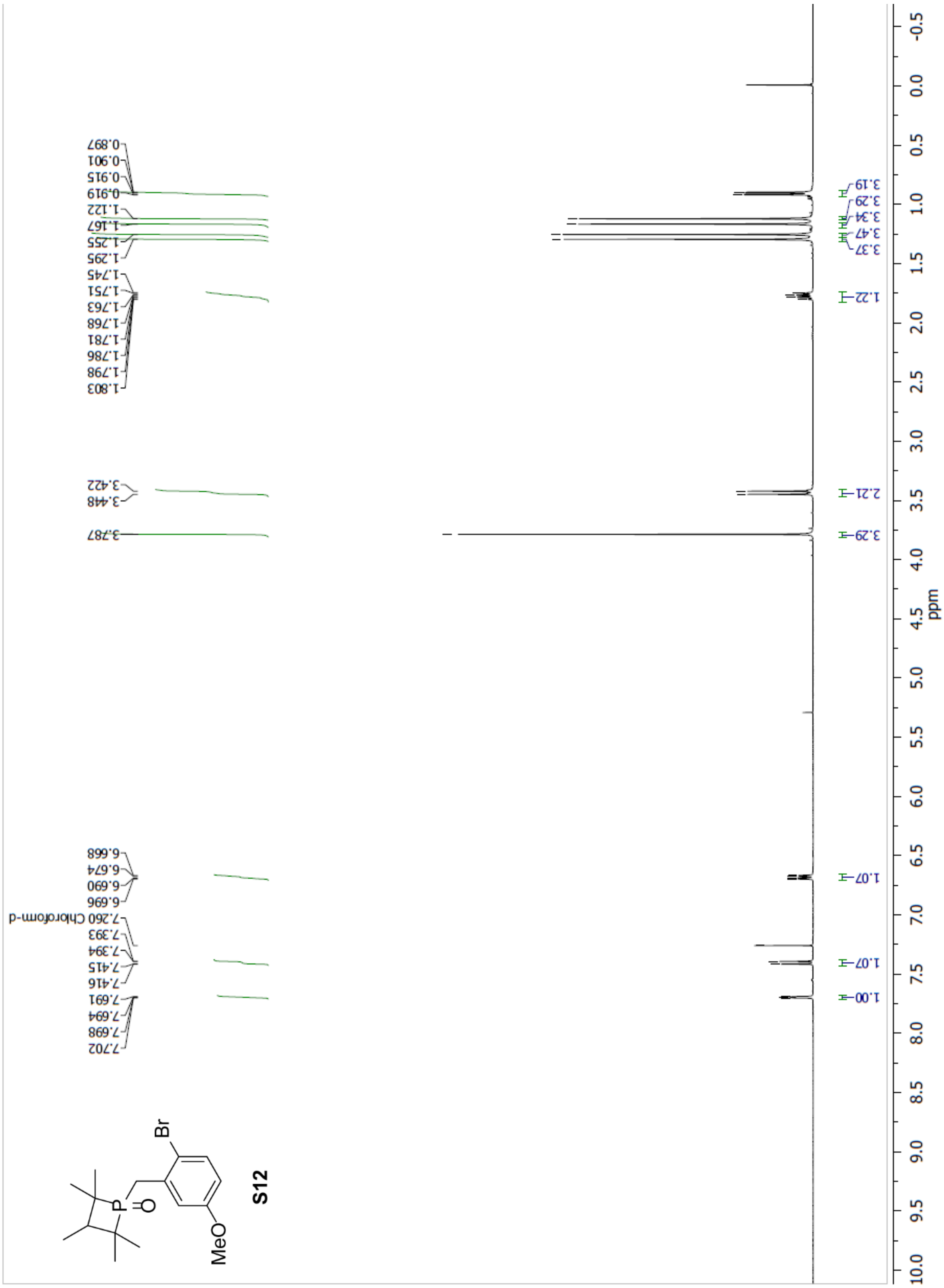

S12

^{13}C NMR in $CDCl_3$

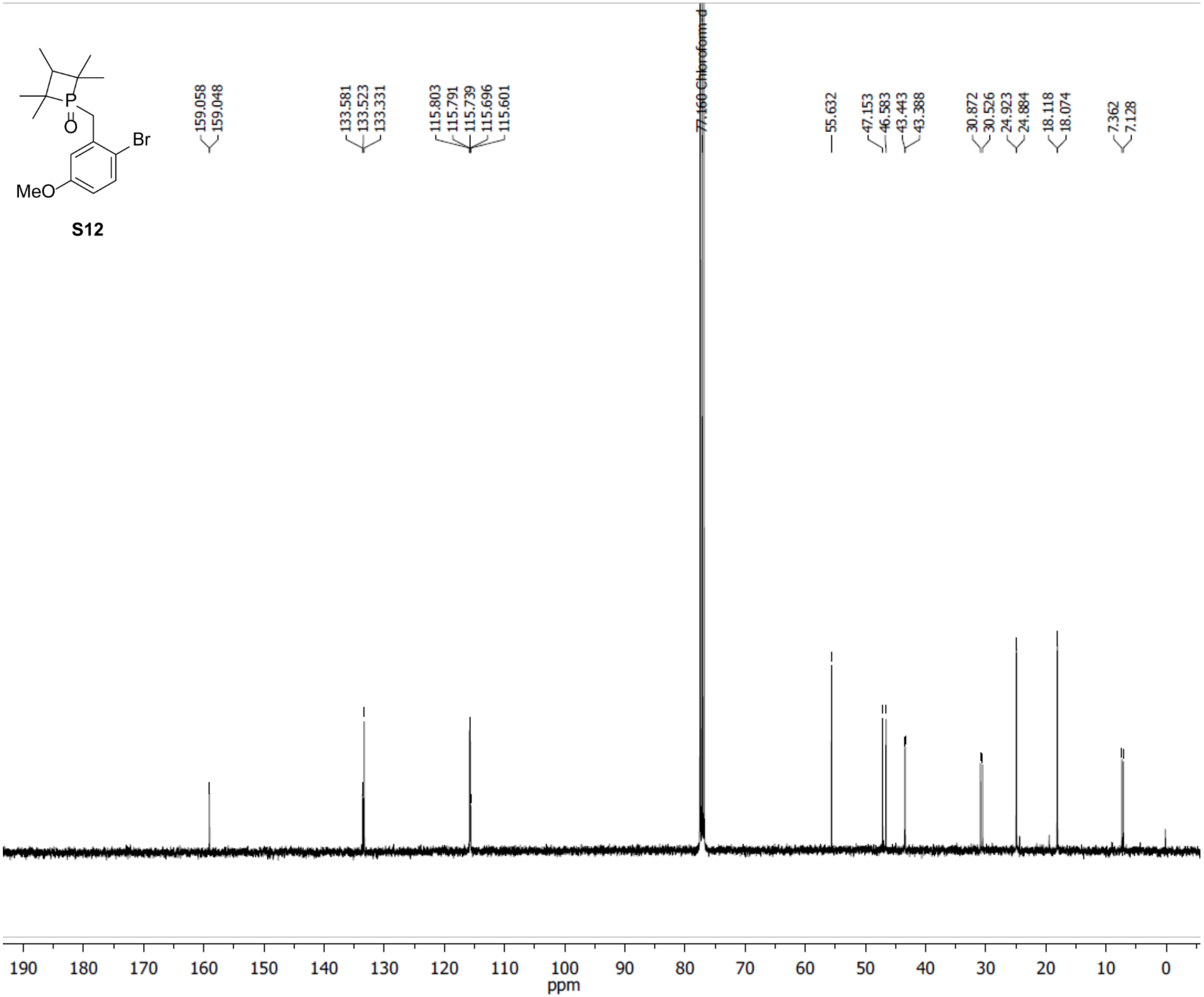

S12

^{31}P NMR in $CDCl_3$

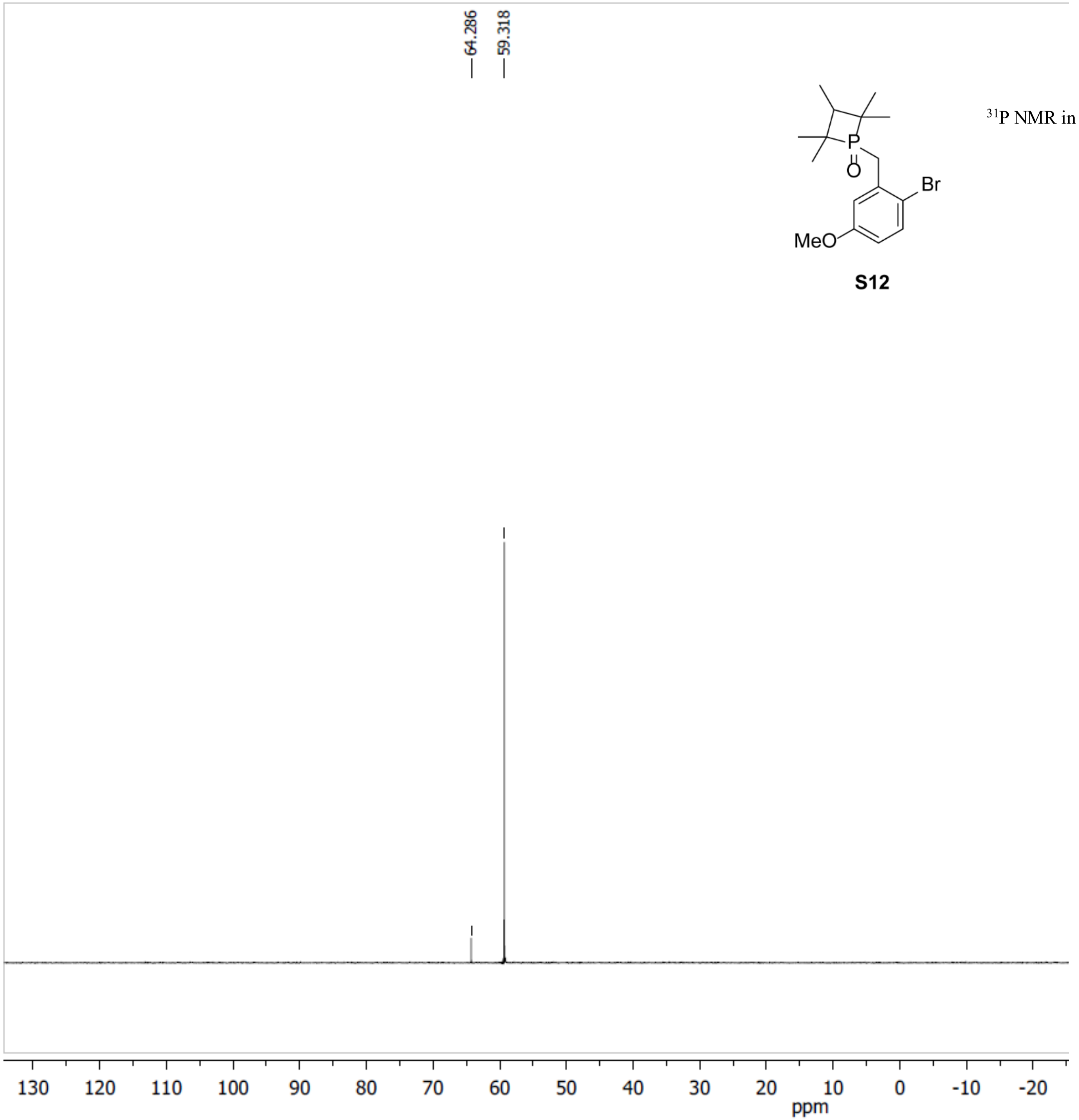

S12

^{1}H NMR in CDCl$_3$

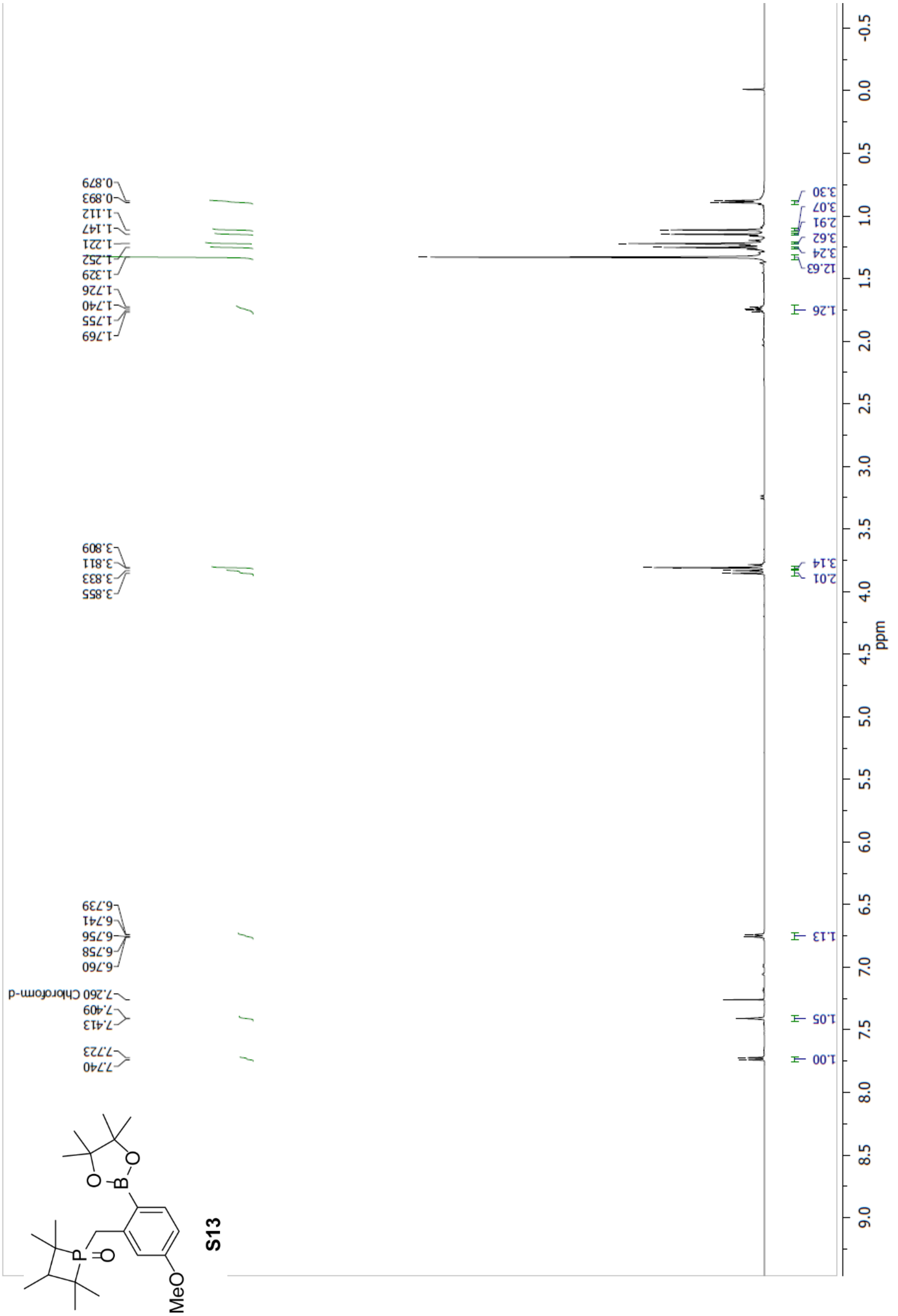

S13

^{13}C NMR in $CDCl_3$

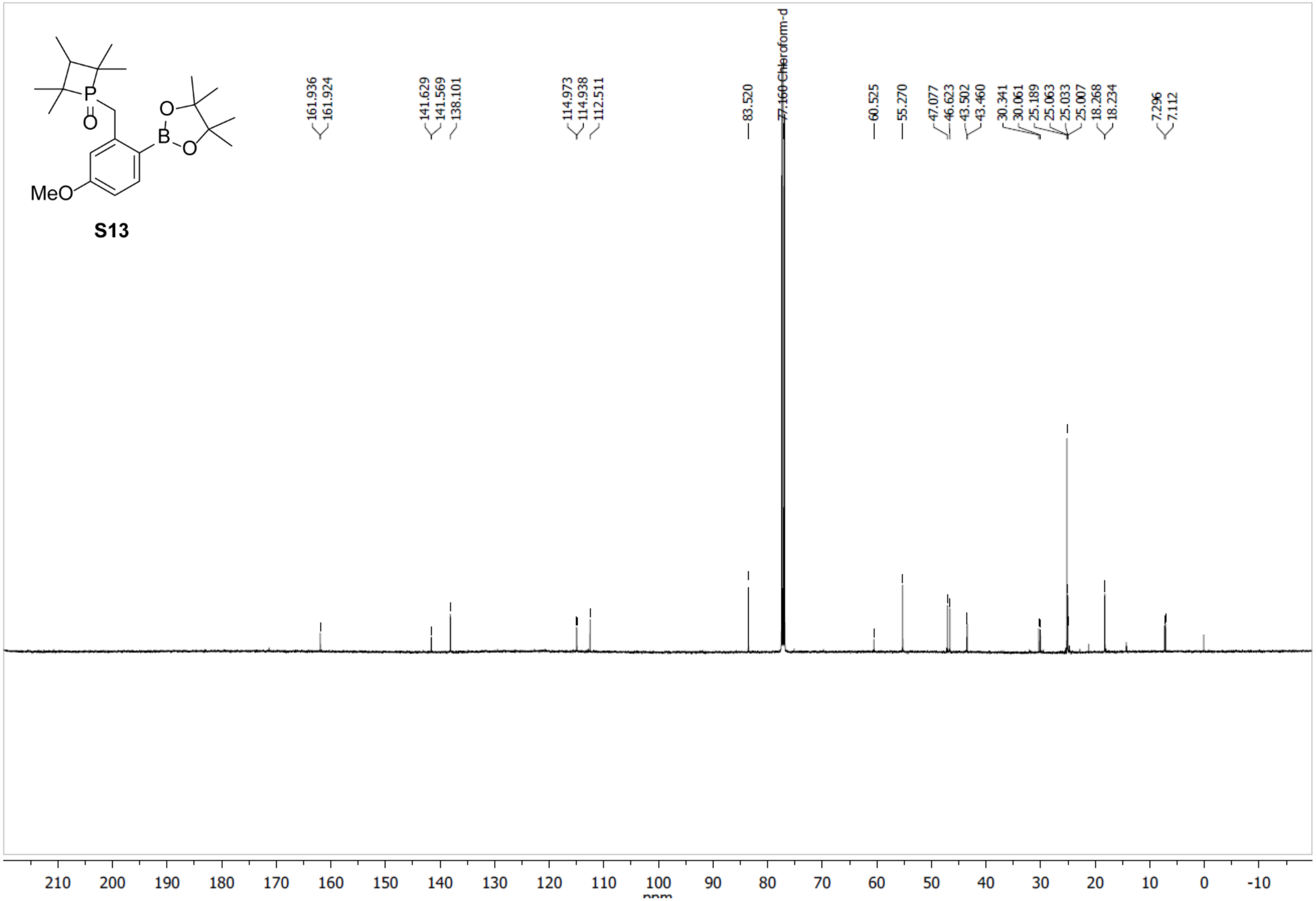

S13

^{31}P NMR in $CDCl_3$

S13

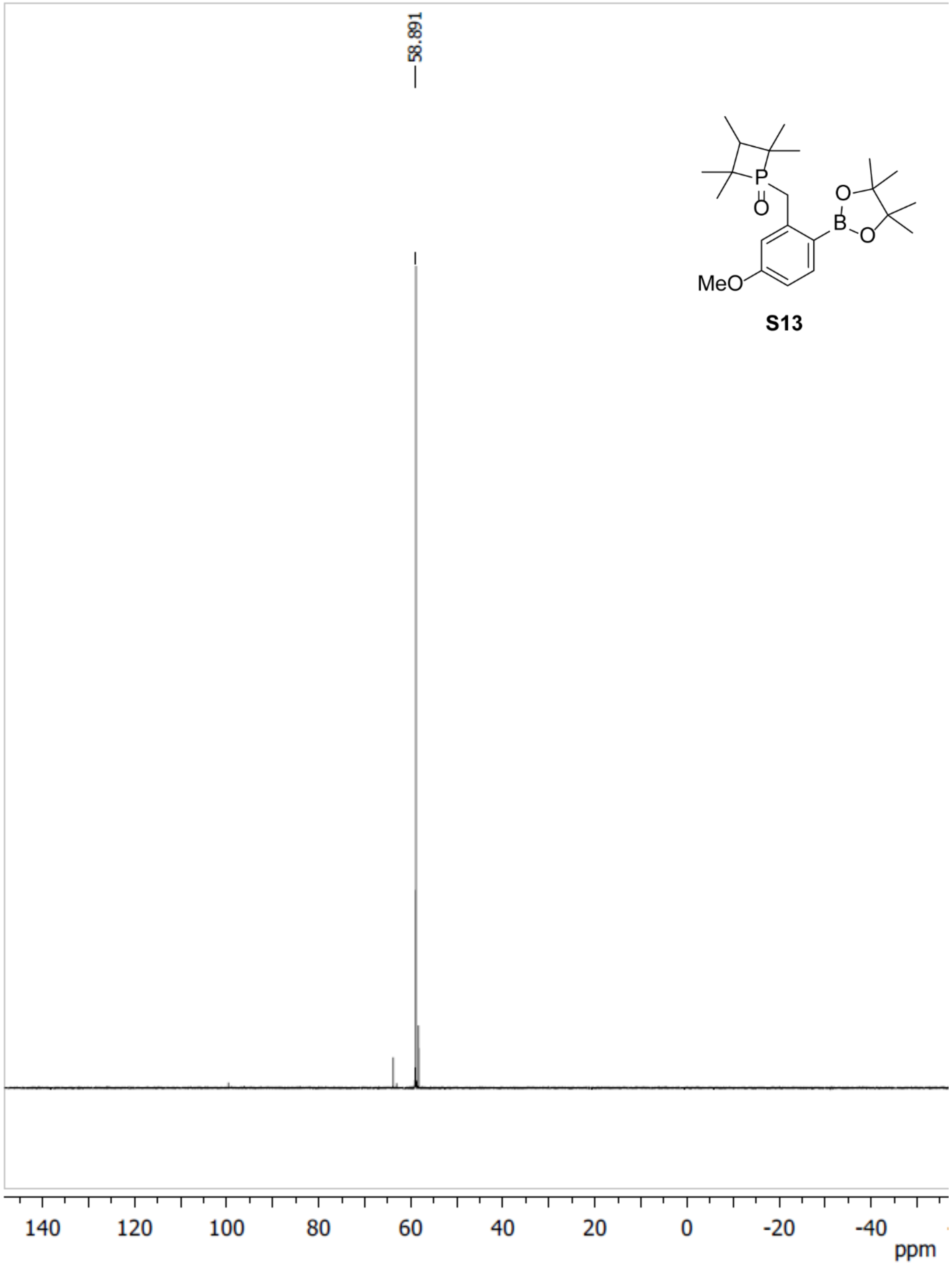

^{1}H NMR in $CDCl_3$

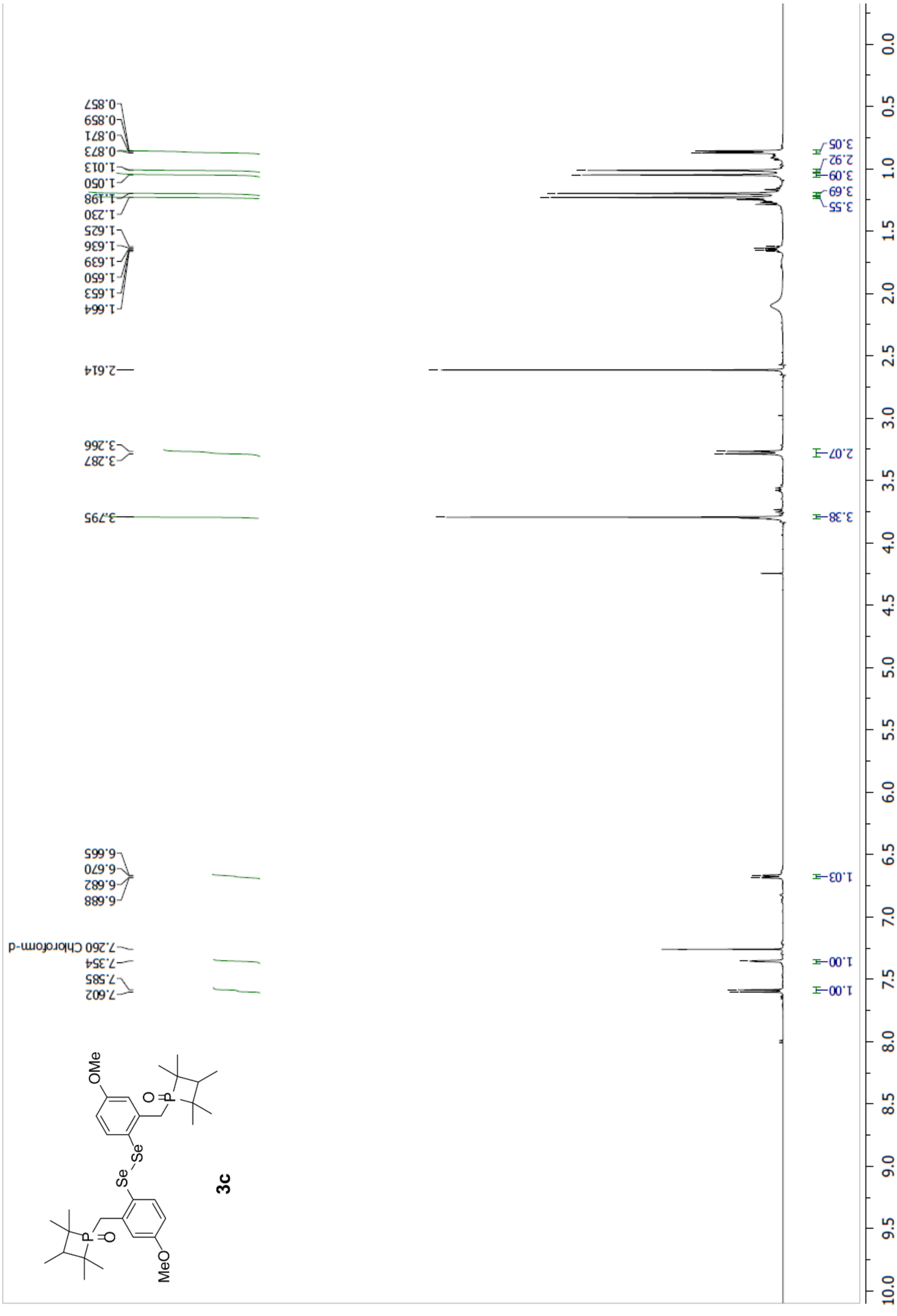

^{13}C NMR in $CDCl_3$

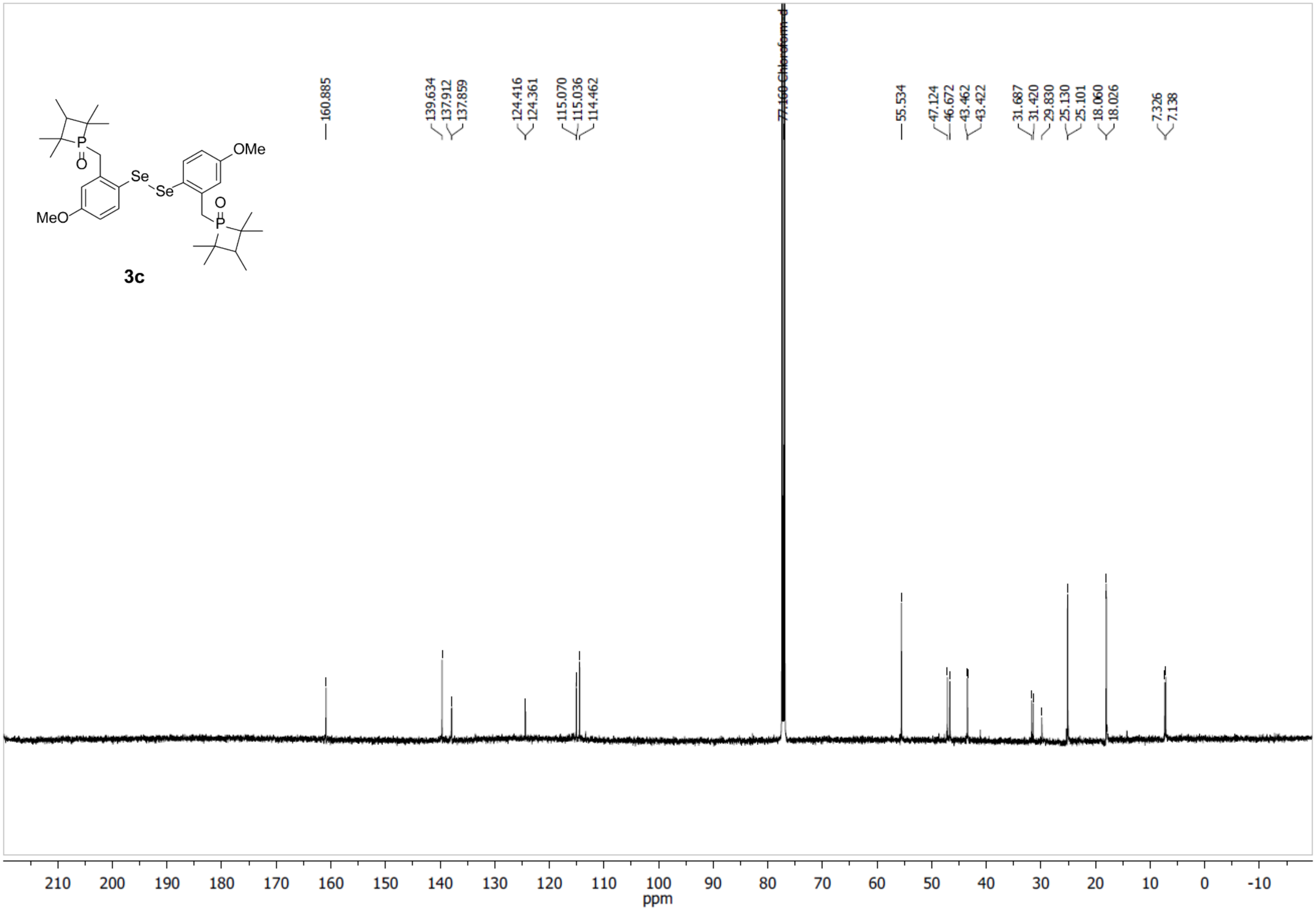

^{31}P NMR in $CDCl_3$

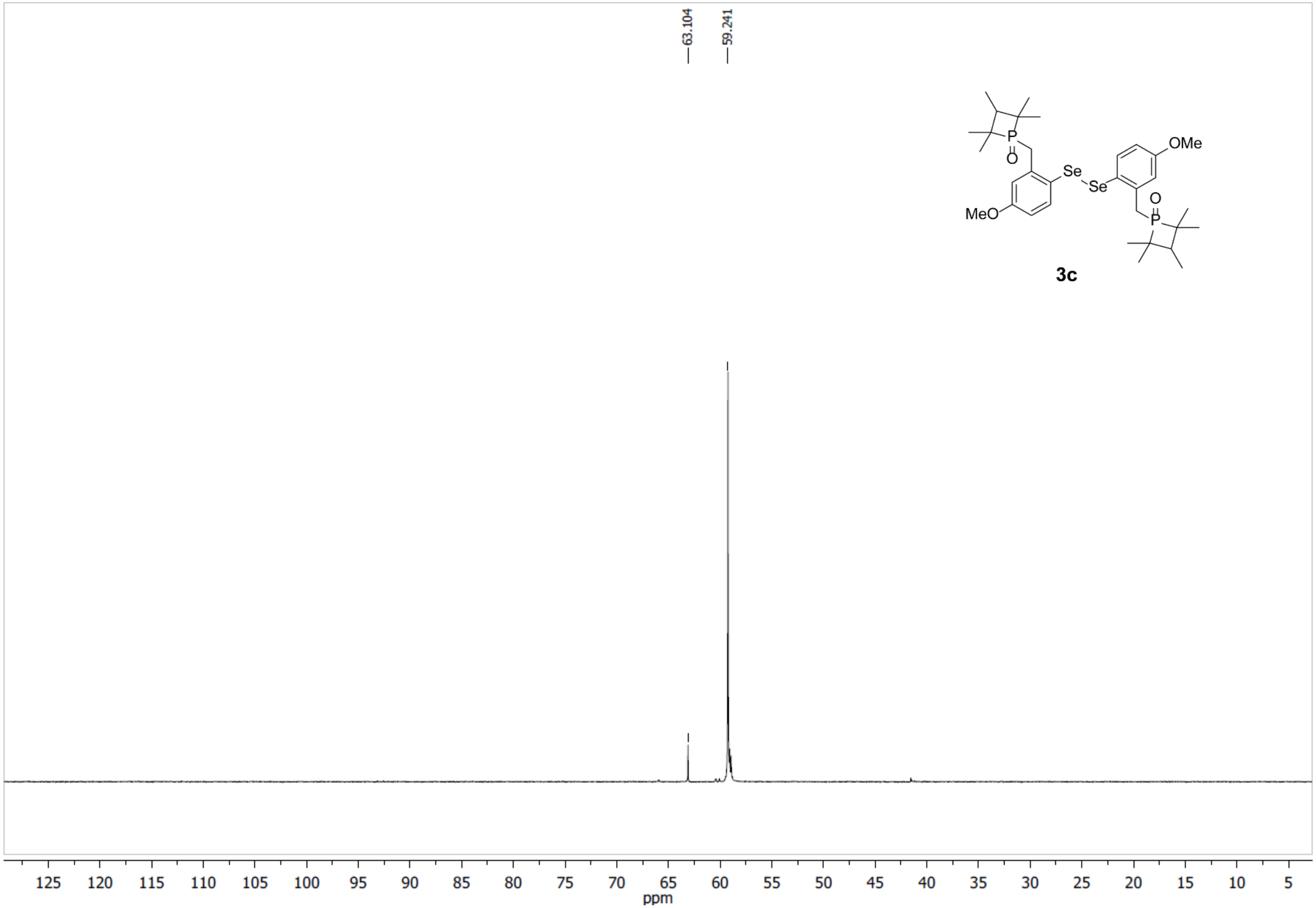

^{77}Se NMR in CDCl$_3$

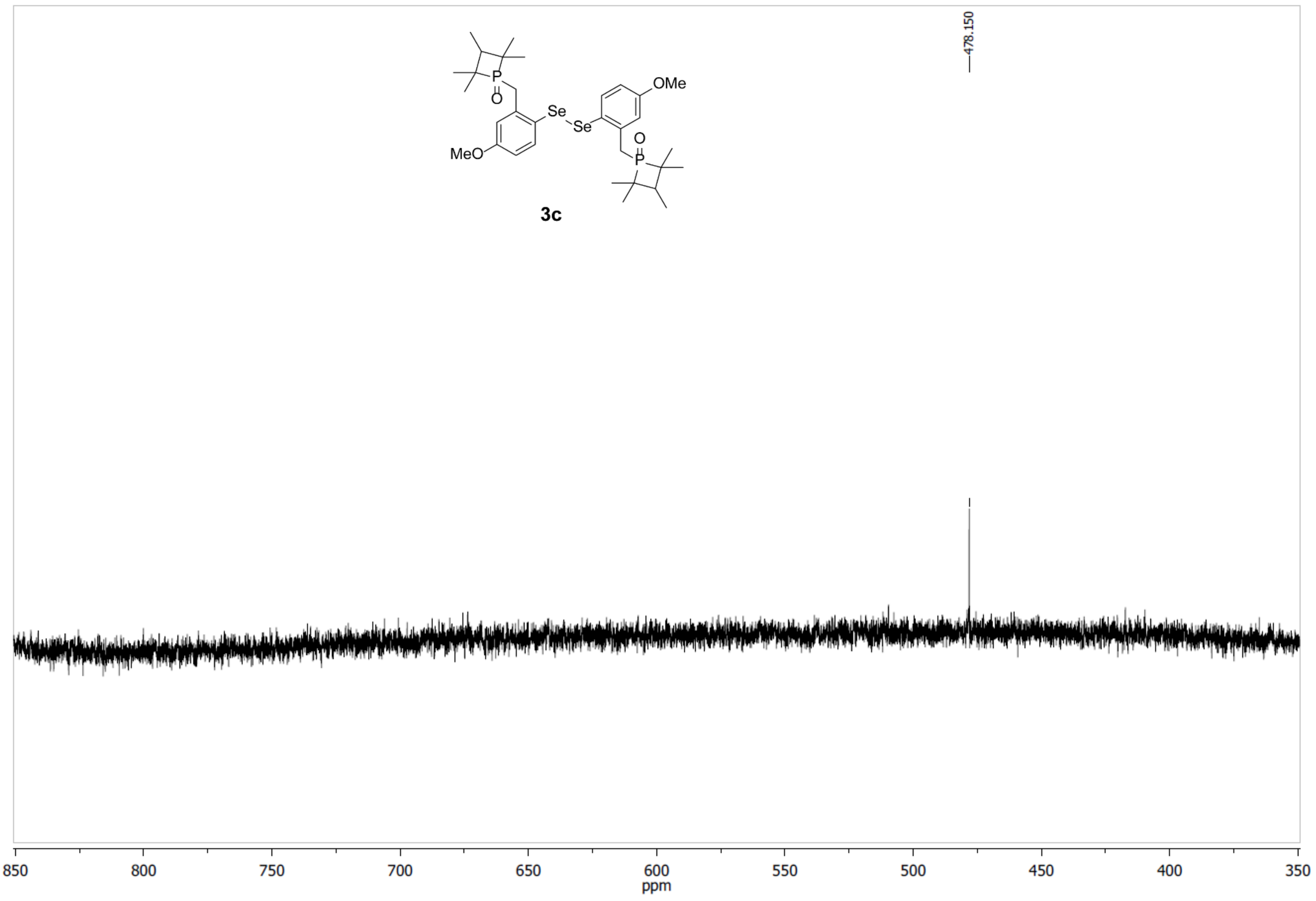

^{1}H NMR in CDCl$_3$

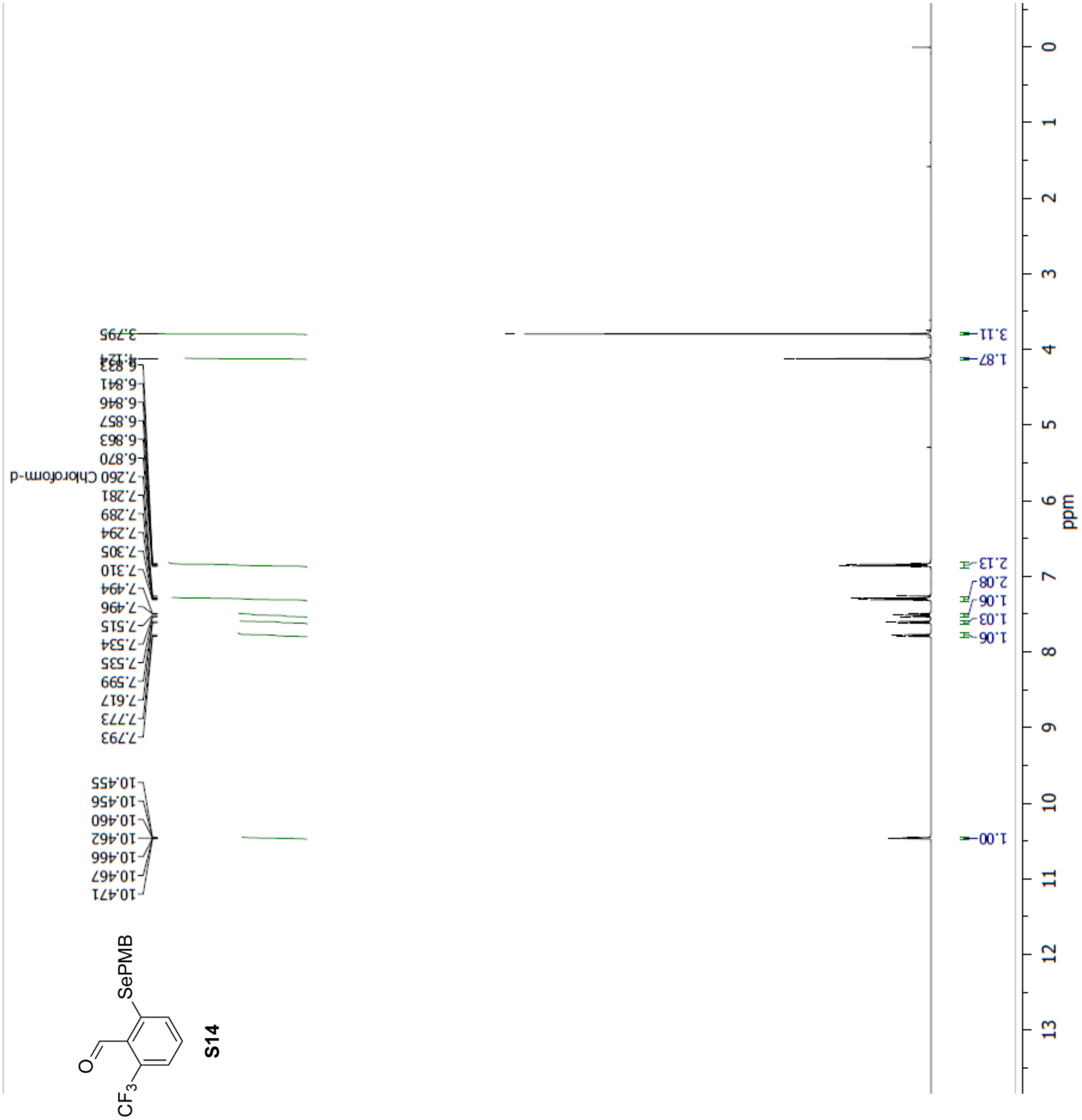

S14

^{13}C NMR in $CDCl_3$

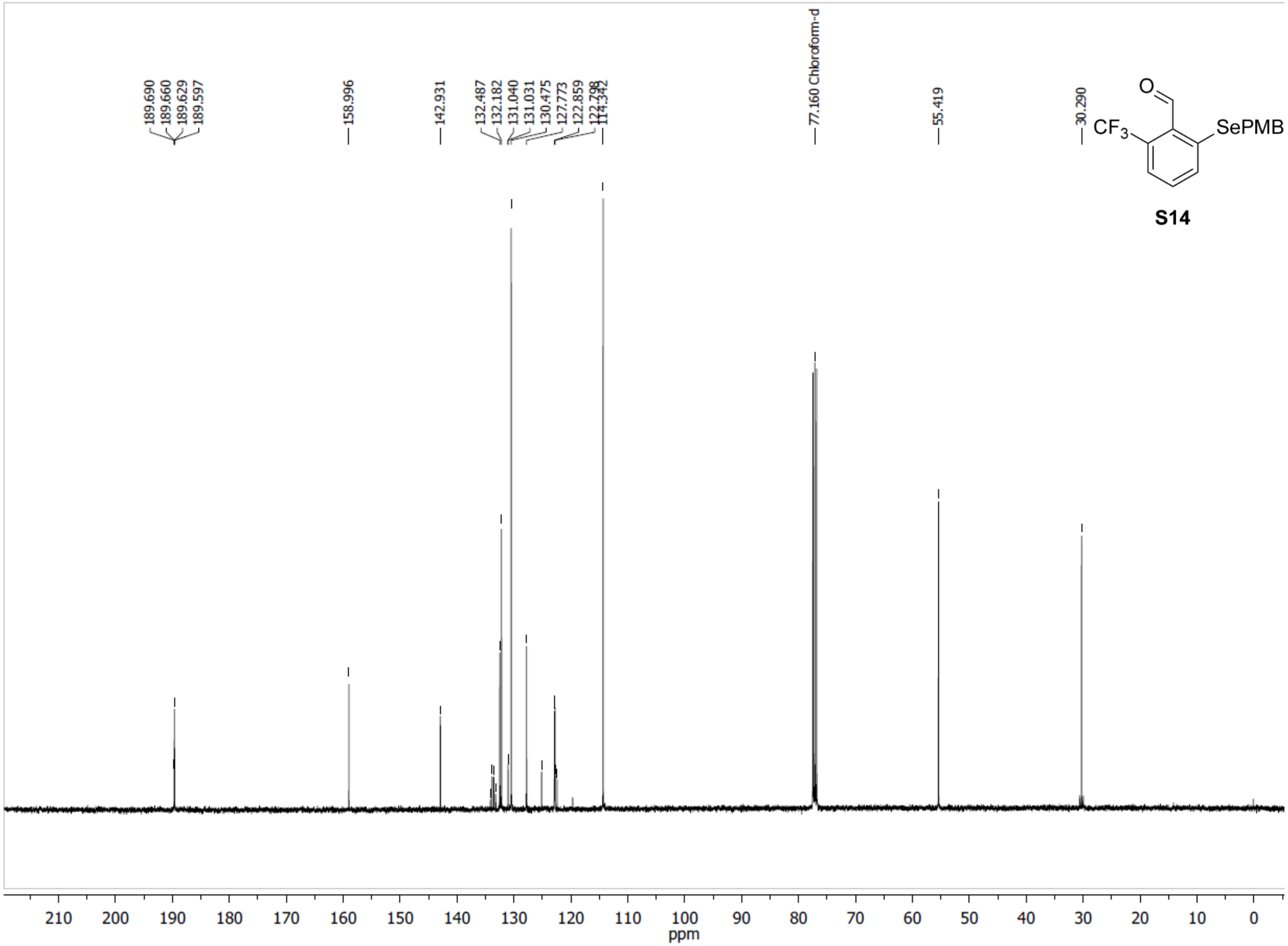

^{19}F NMR in $CDCl_3$

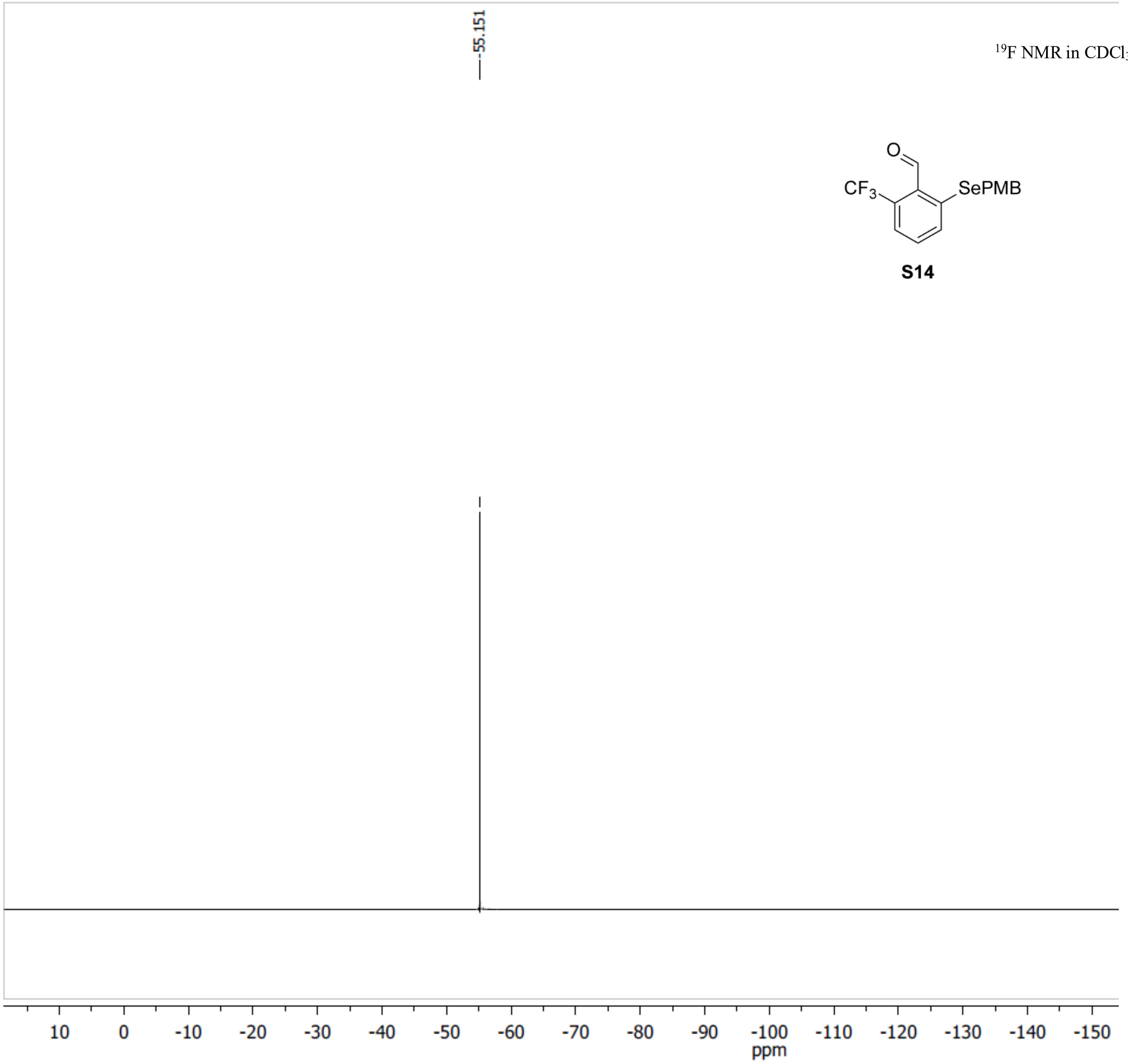

S14

^{77}Se NMR in $CDCl_3$

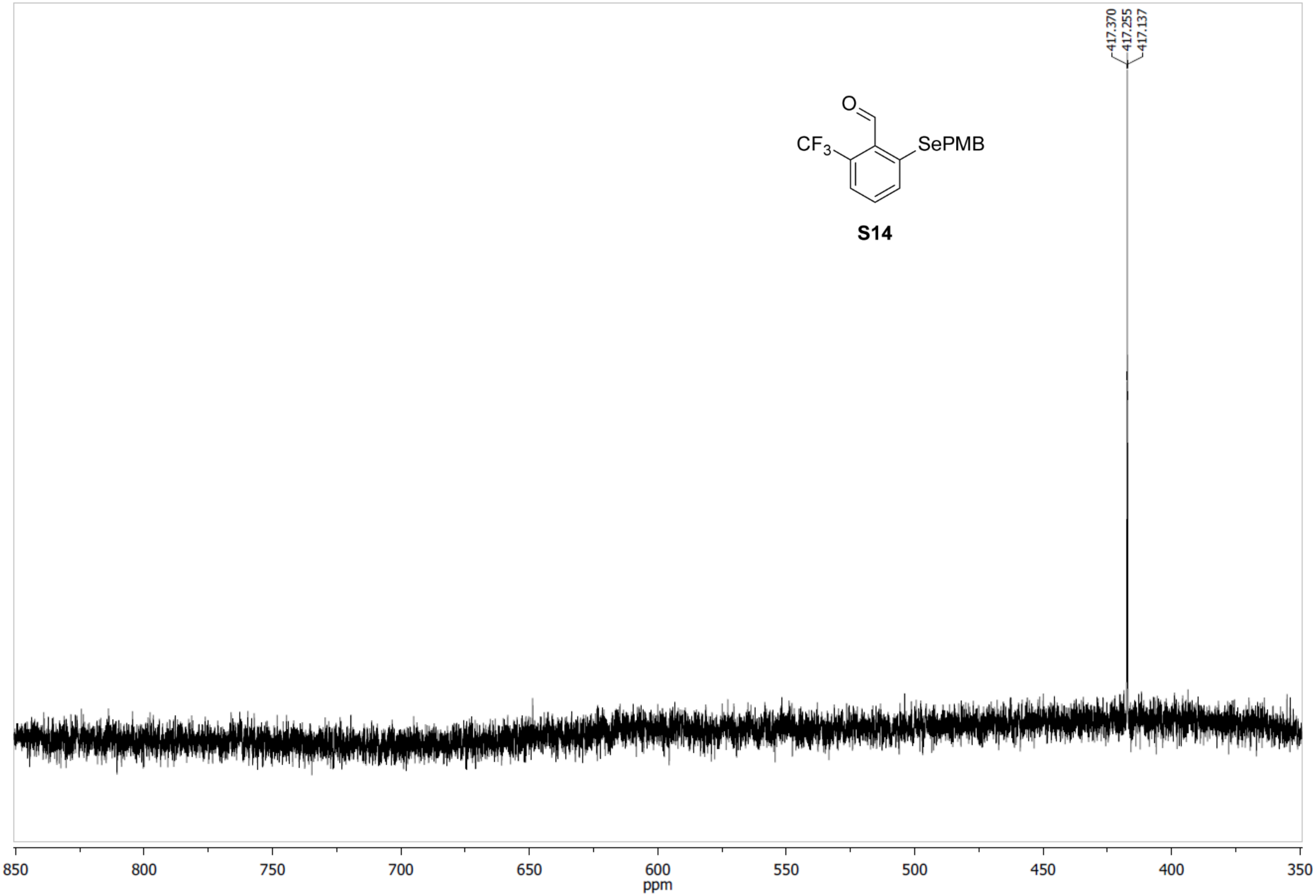

^1H NMR in $CDCl_3$

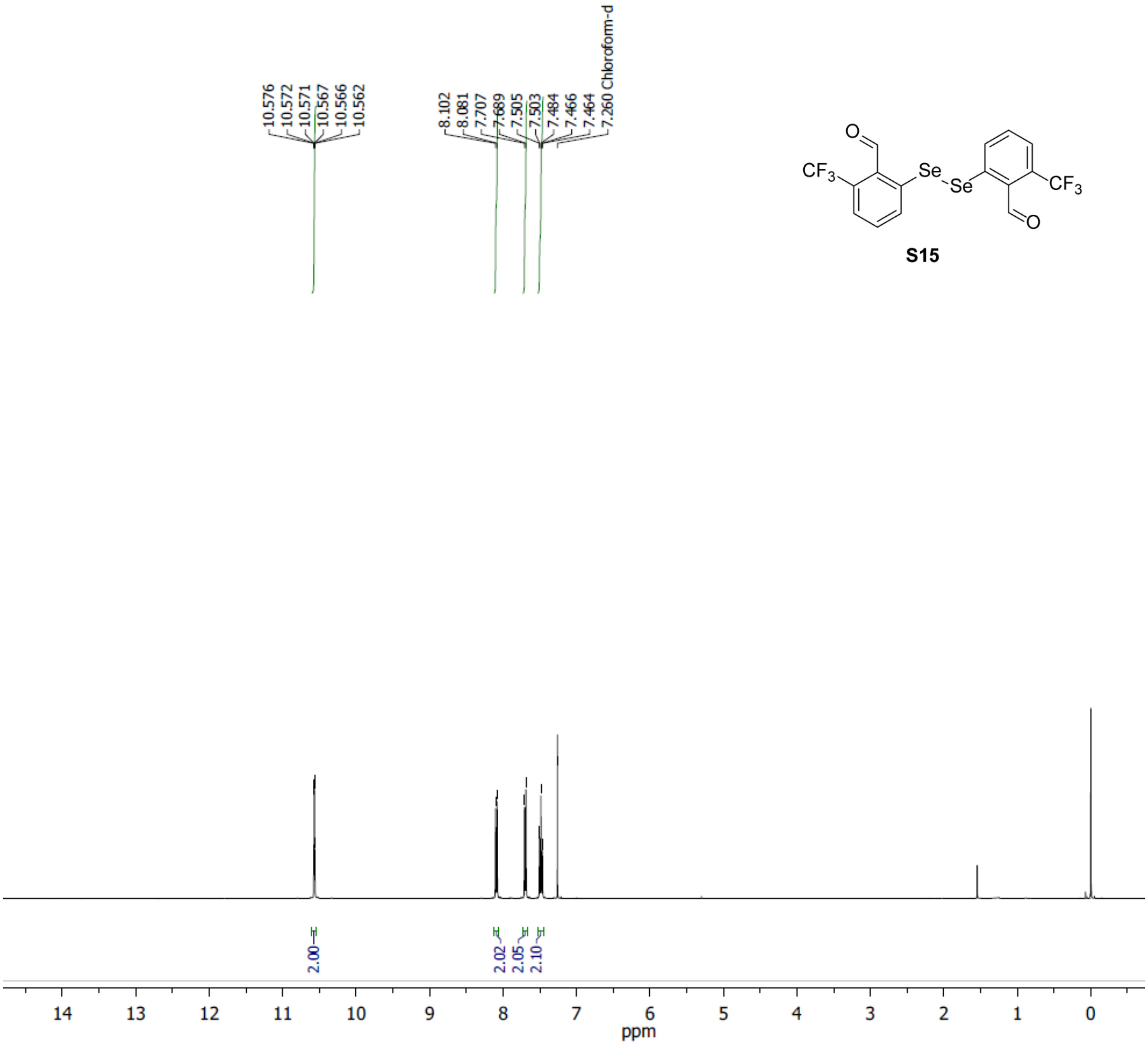

^{13}C NMR in $CDCl_3$

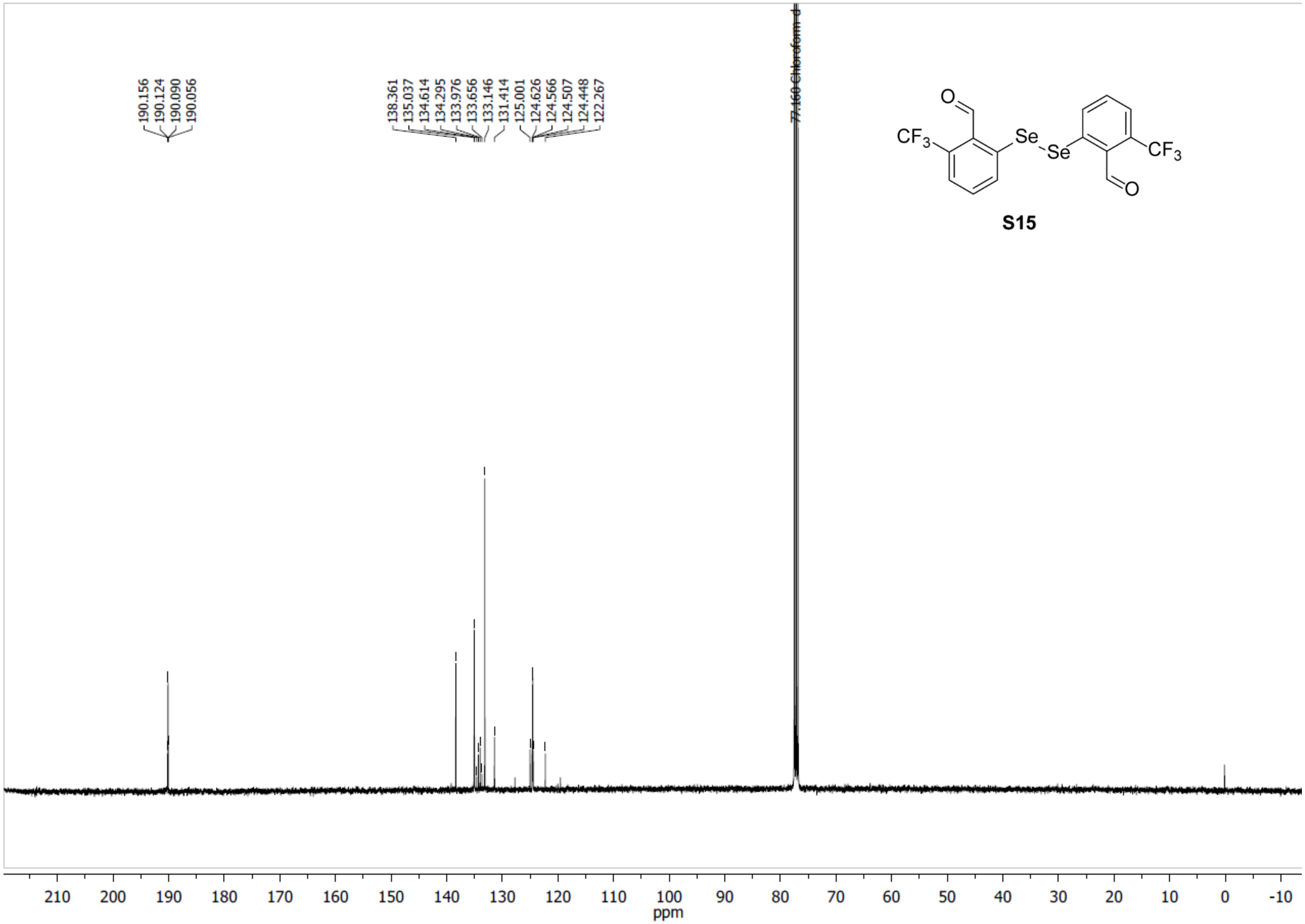

^{19}F NMR in $CDCl_3$

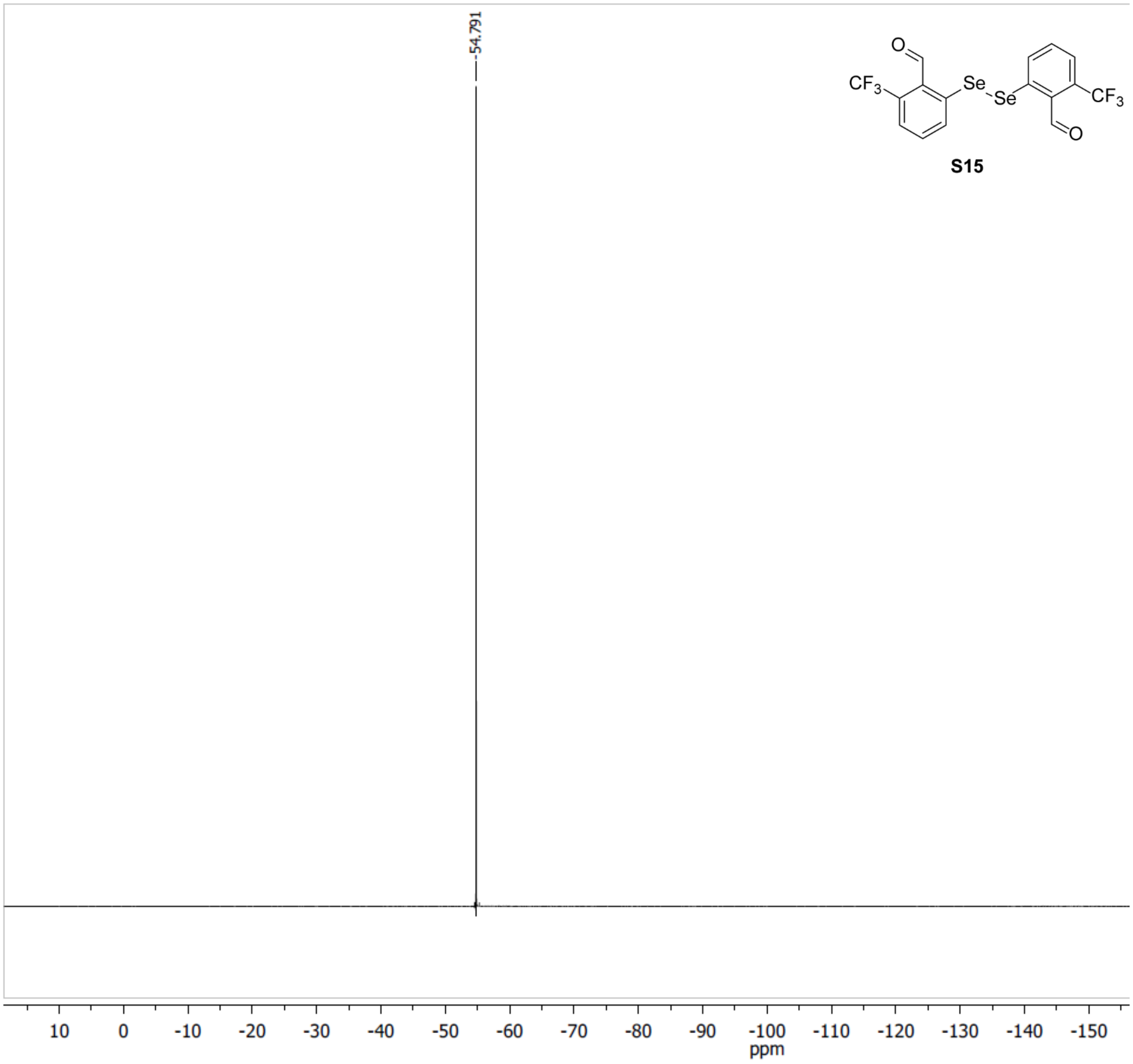

^{77}Se NMR in CDCl$_3$

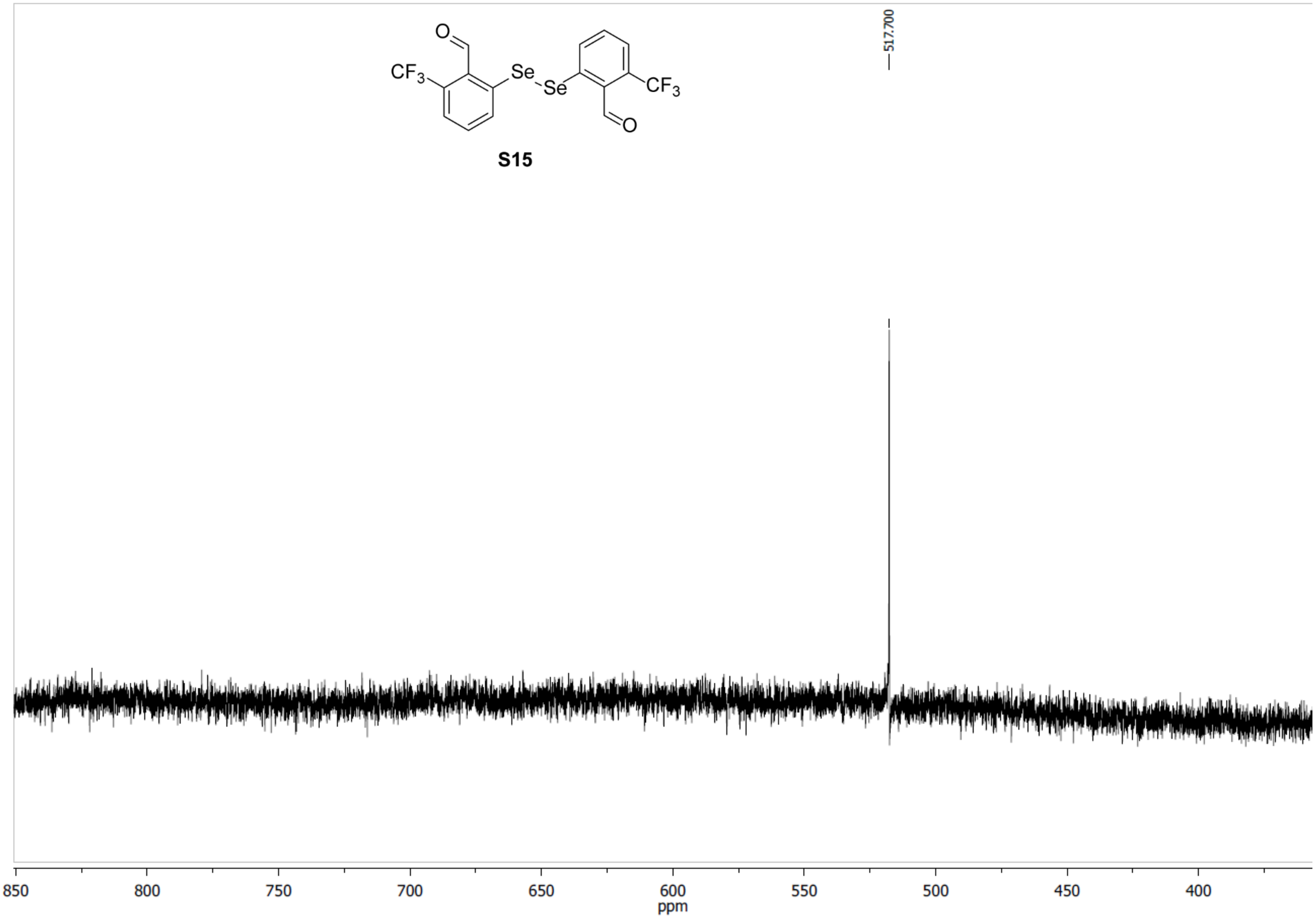

^{1}H NMR in DMSO-d$_6$

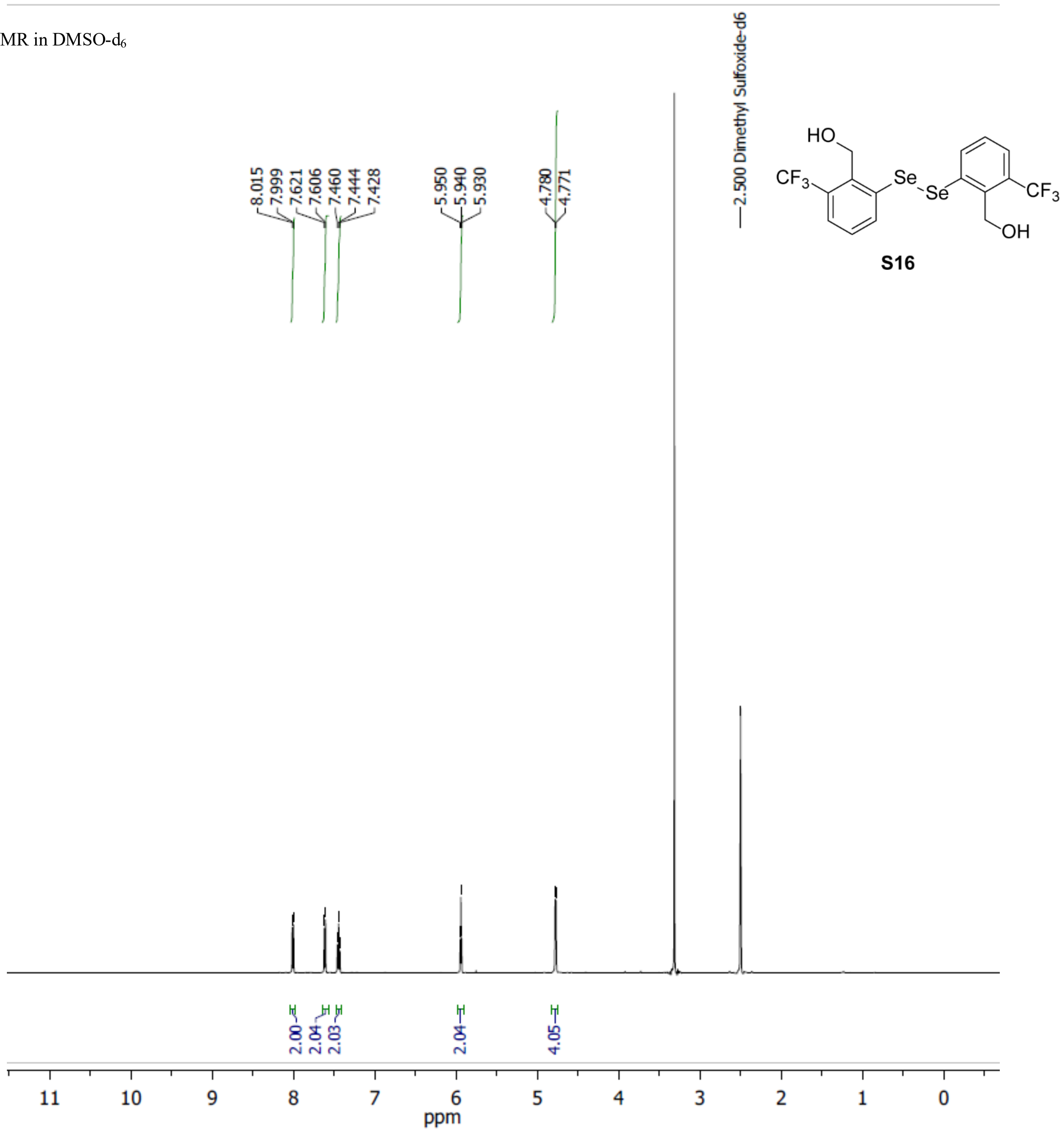

^{13}C NMR in DMSO-d$_6$

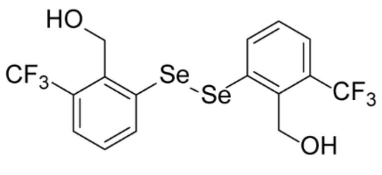

S16

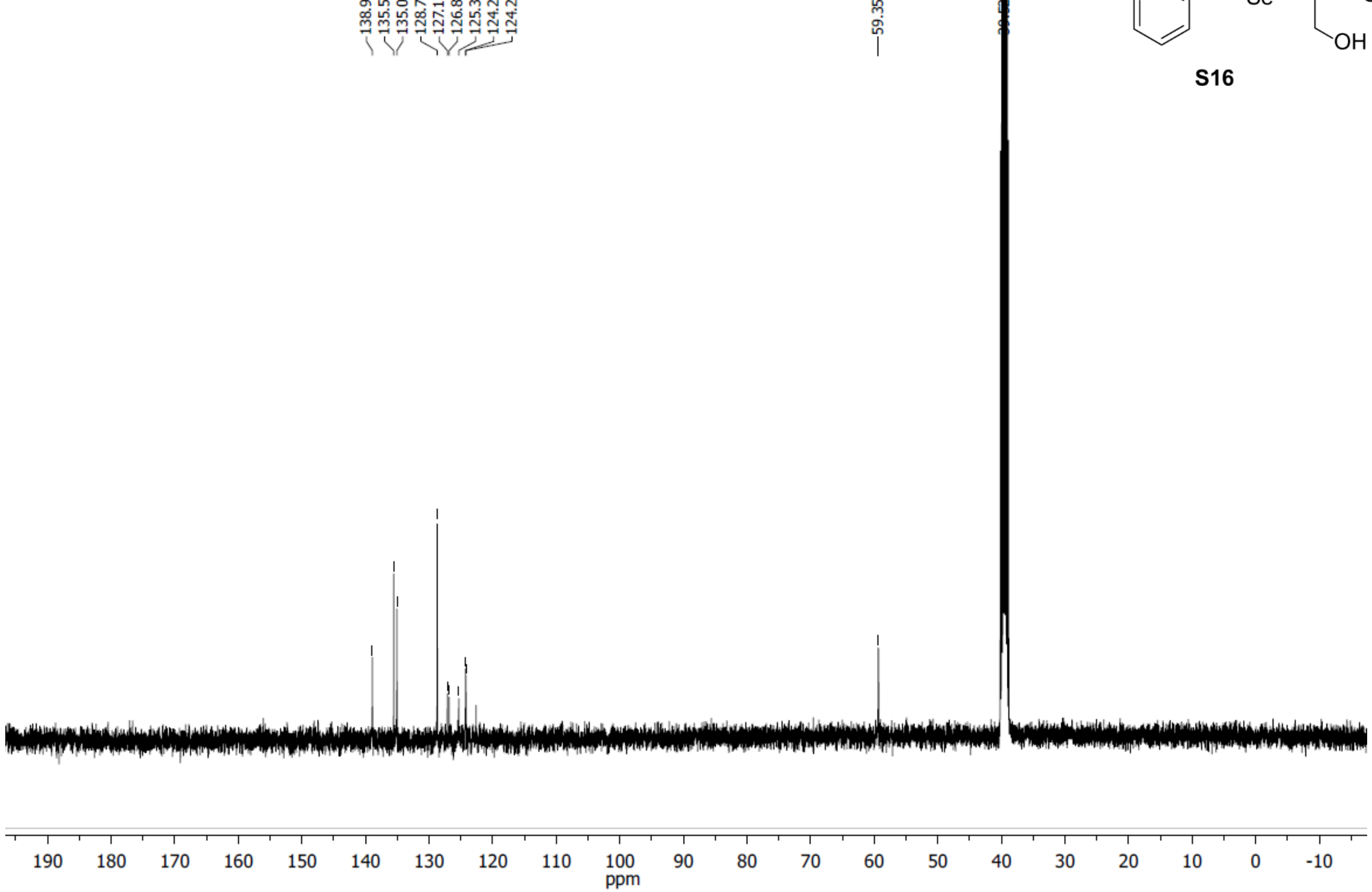

^{19}F NMR in DMSO-d_6

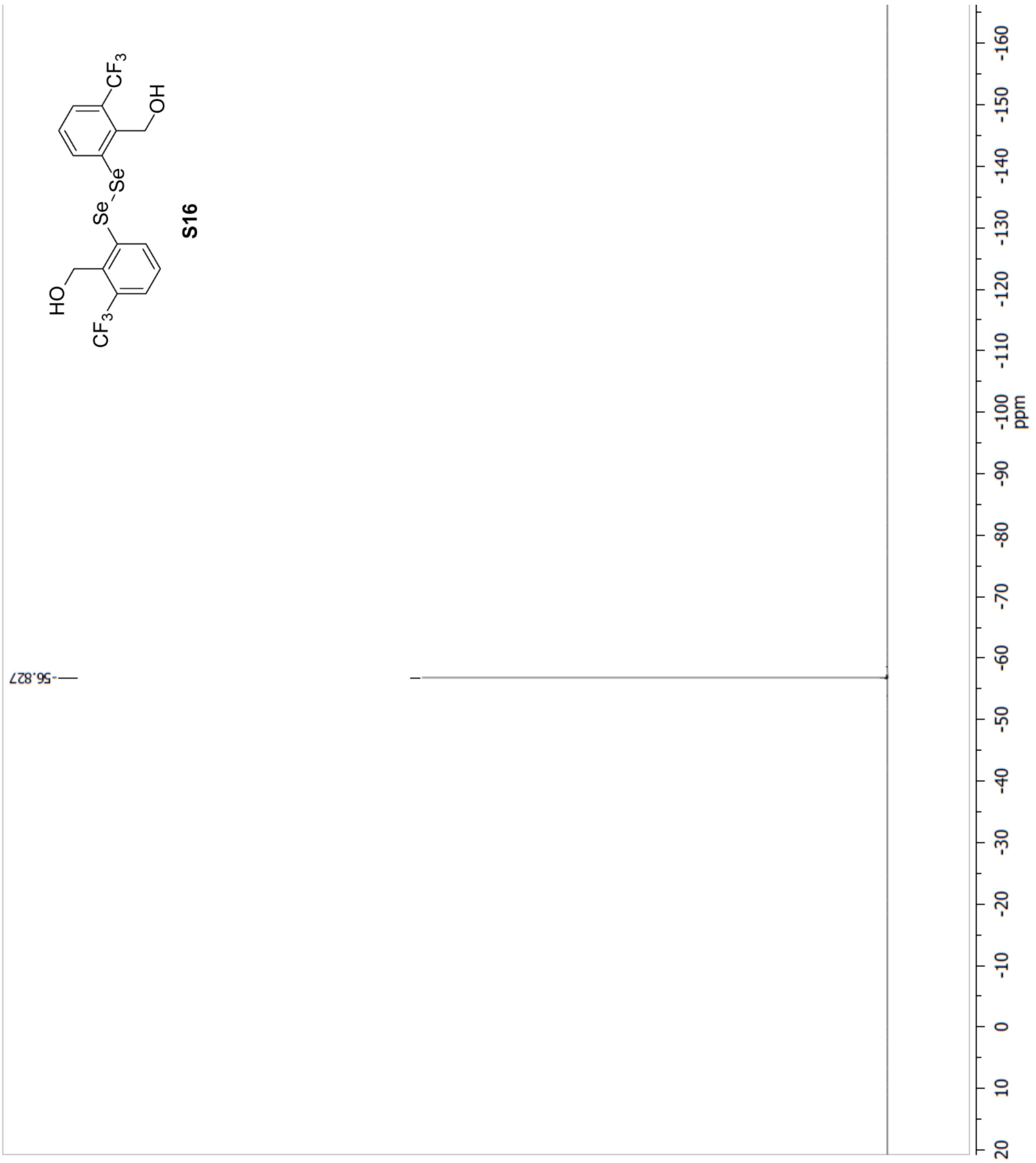

^{77}Se NMR in DMSO-d_6

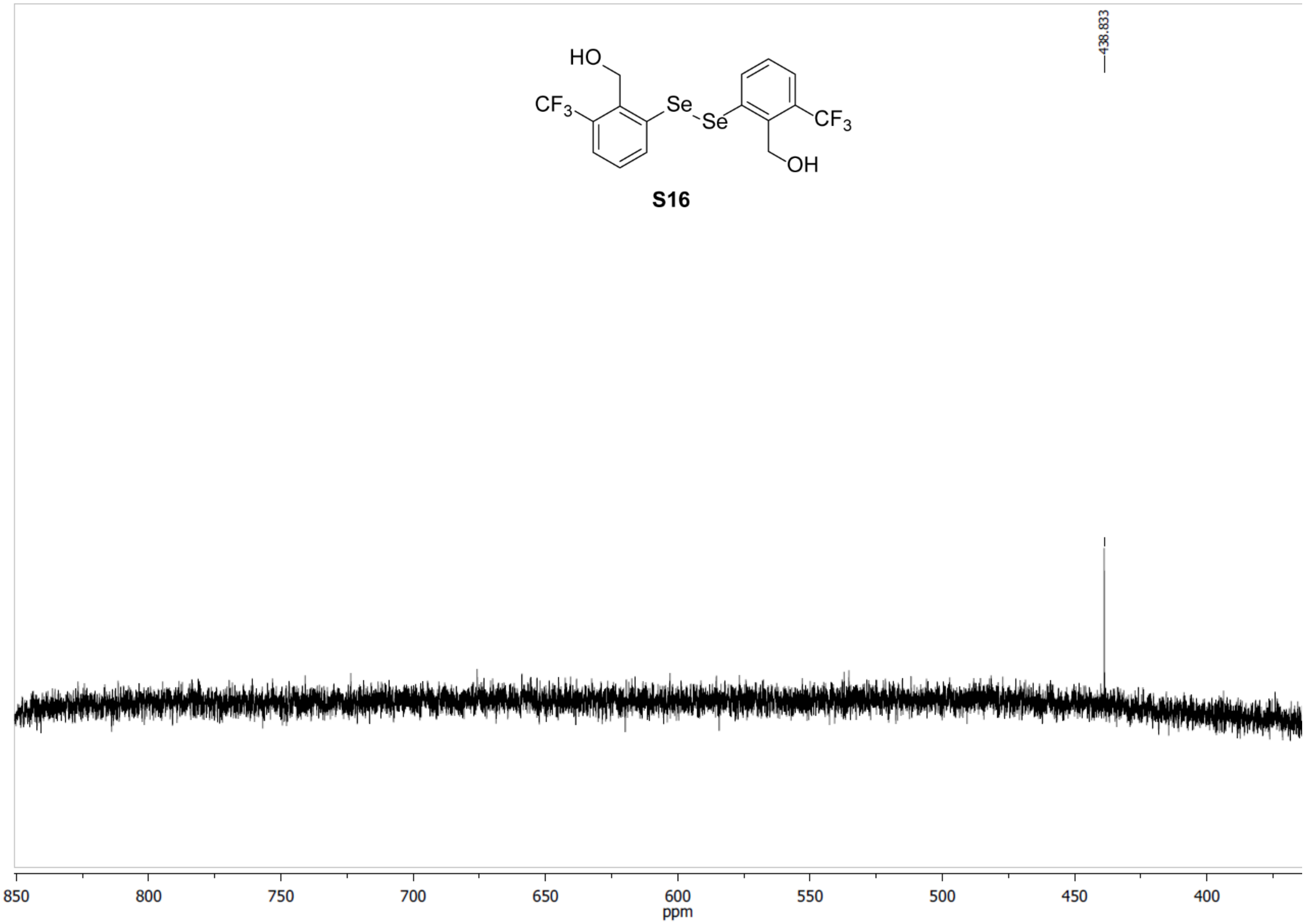

^{1}H NMR in $CDCl_3$

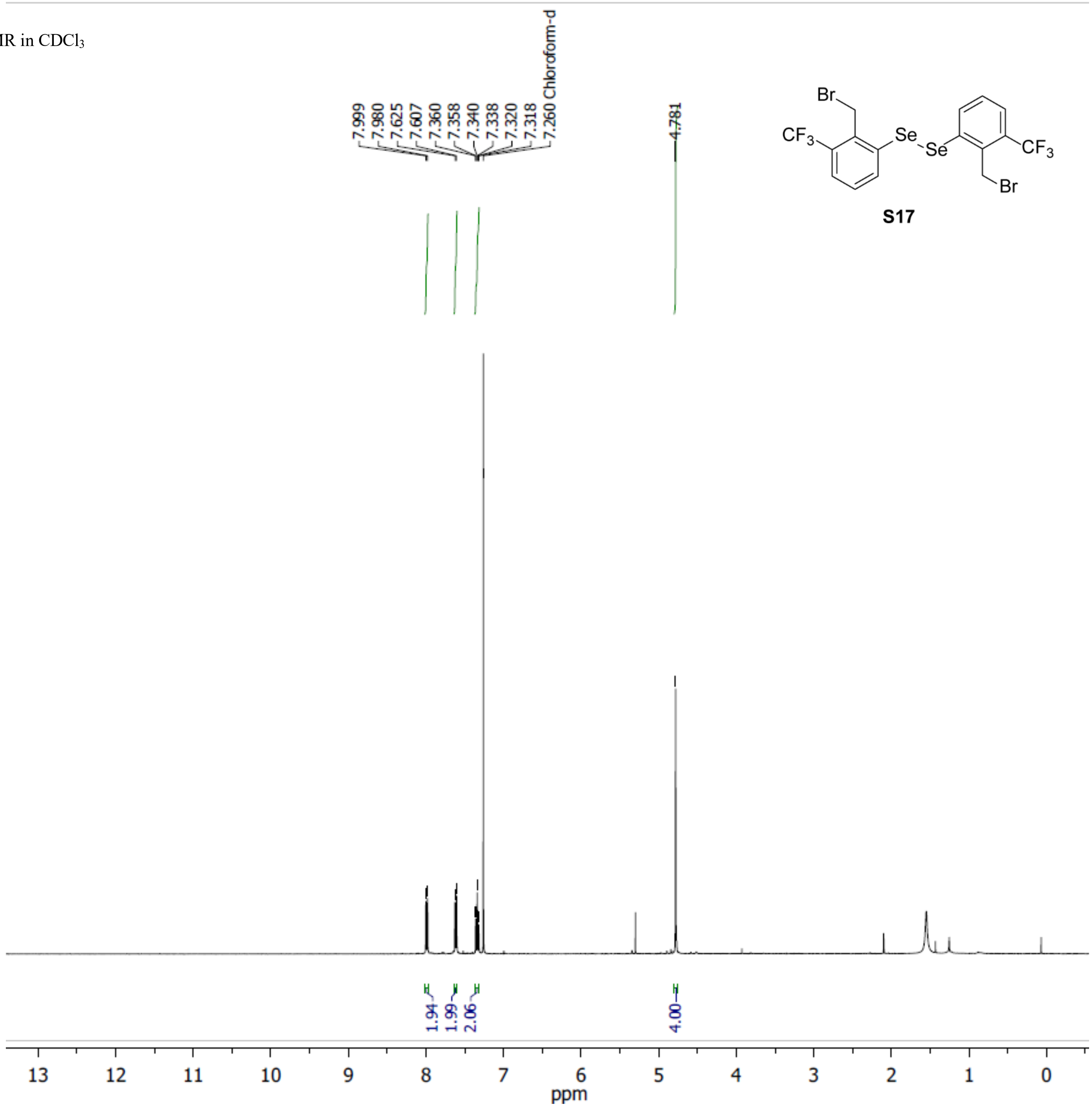

S17

^{13}C NMR in $CDCl_3$

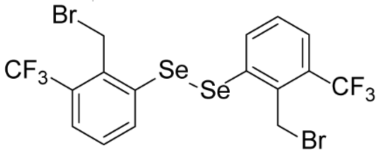

S17

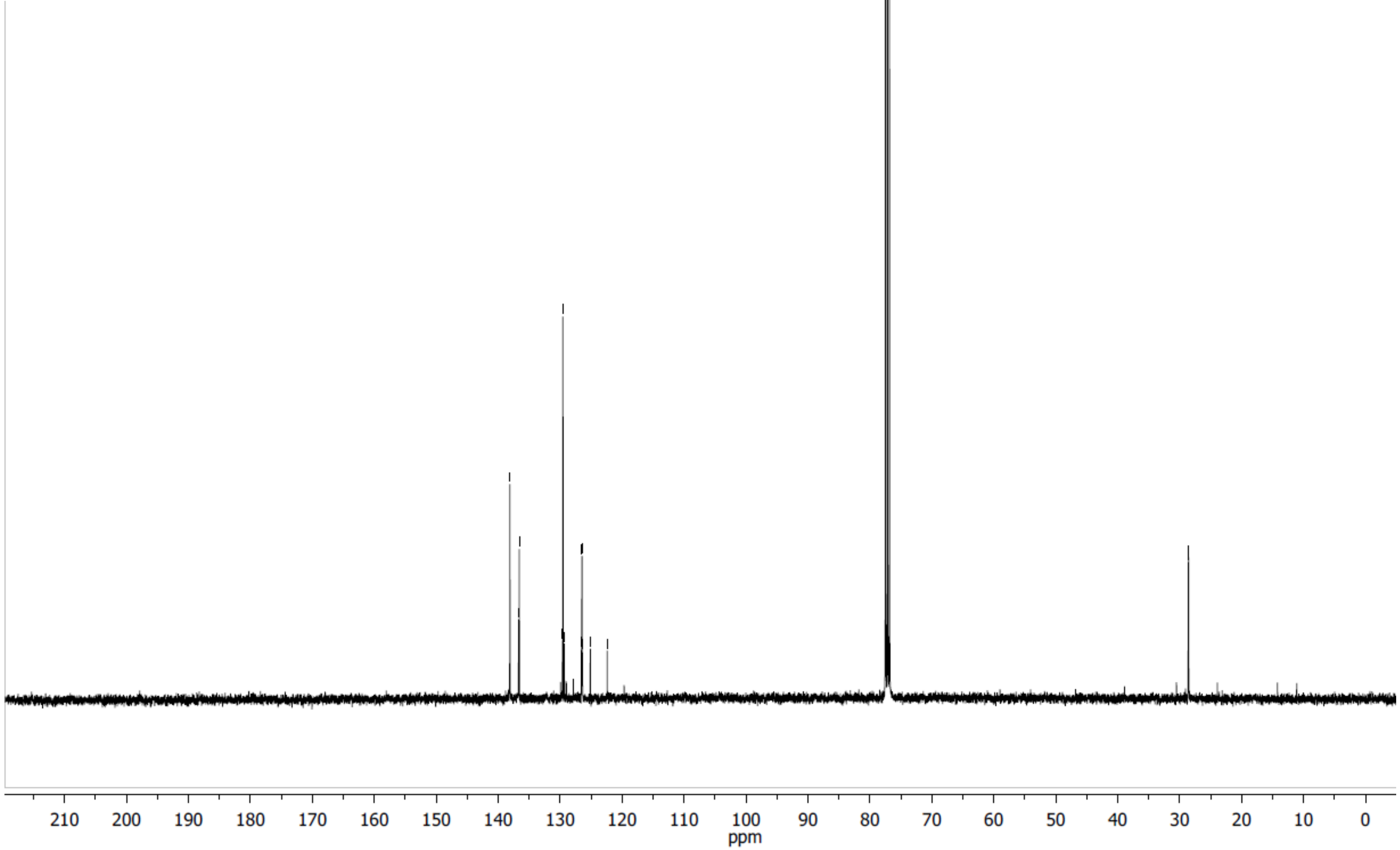

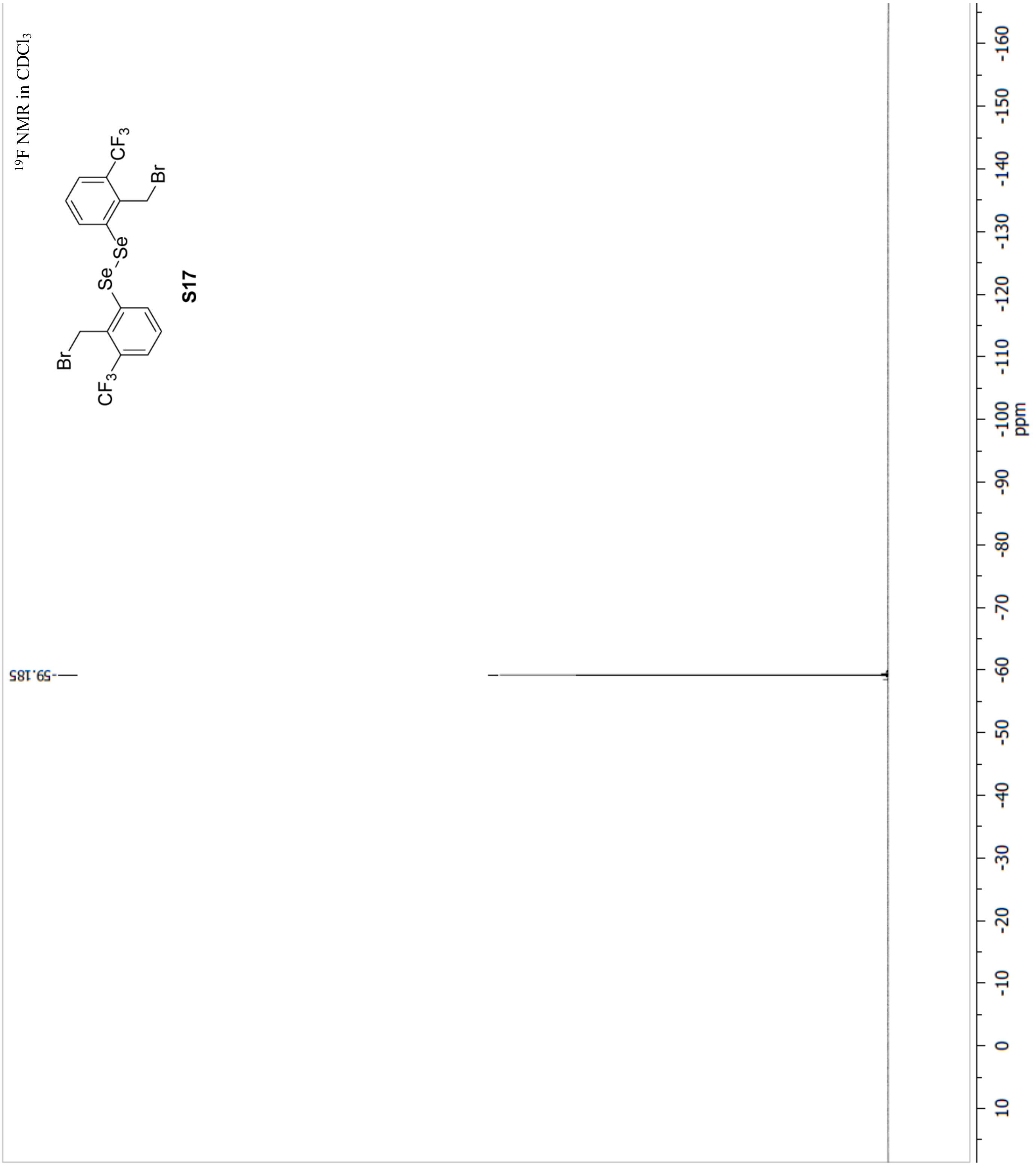
19F NMR in CDCl3
CF3
Br
Se
Se
Br
CF3
S17
-59.185
ppm

^{77}Se NMR in $CDCl_3$

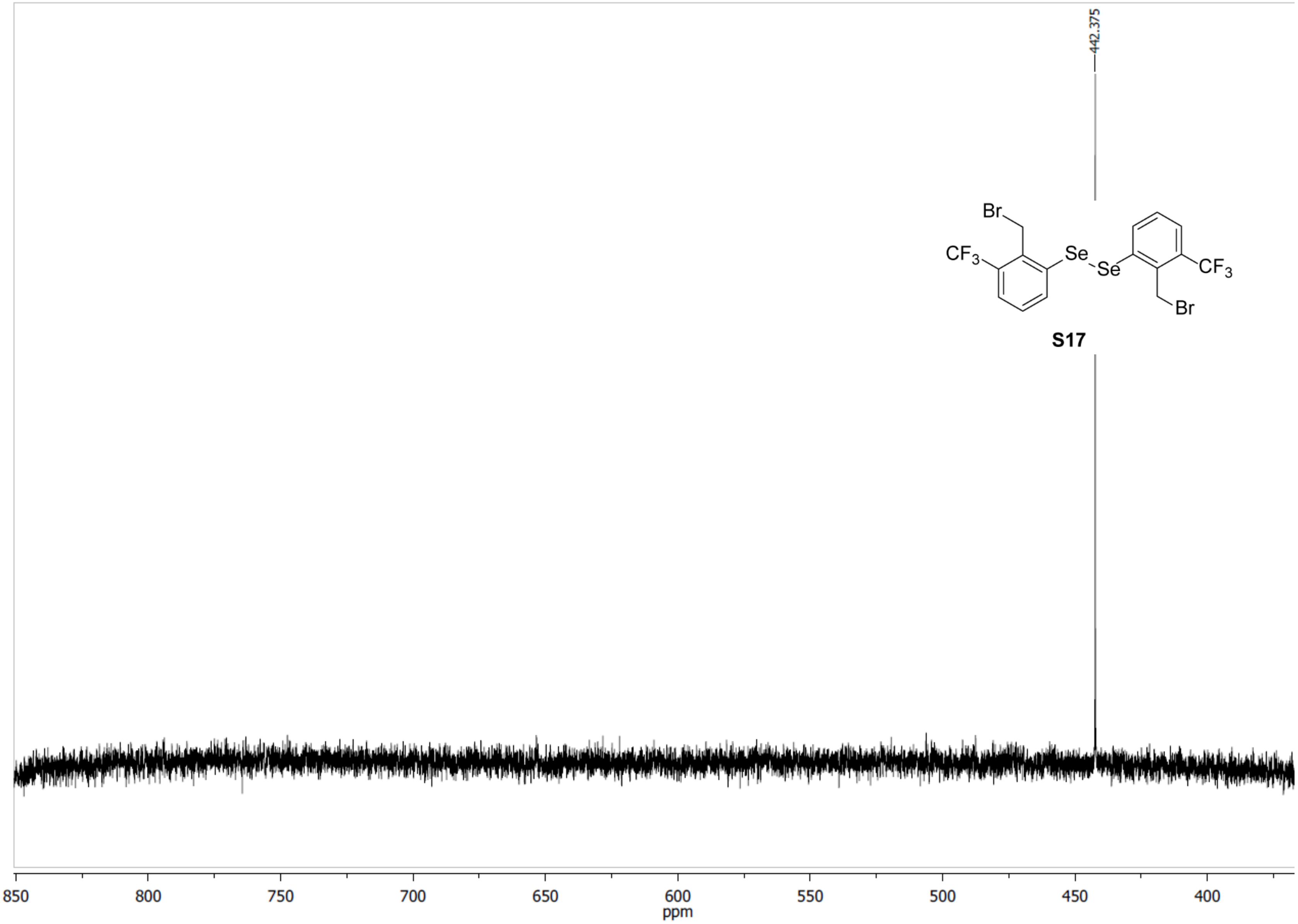

^{1}H NMR in $CDCl_3$

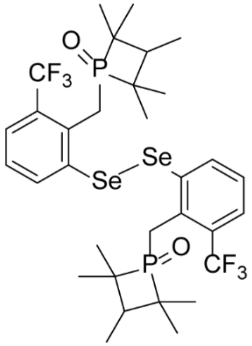

3d

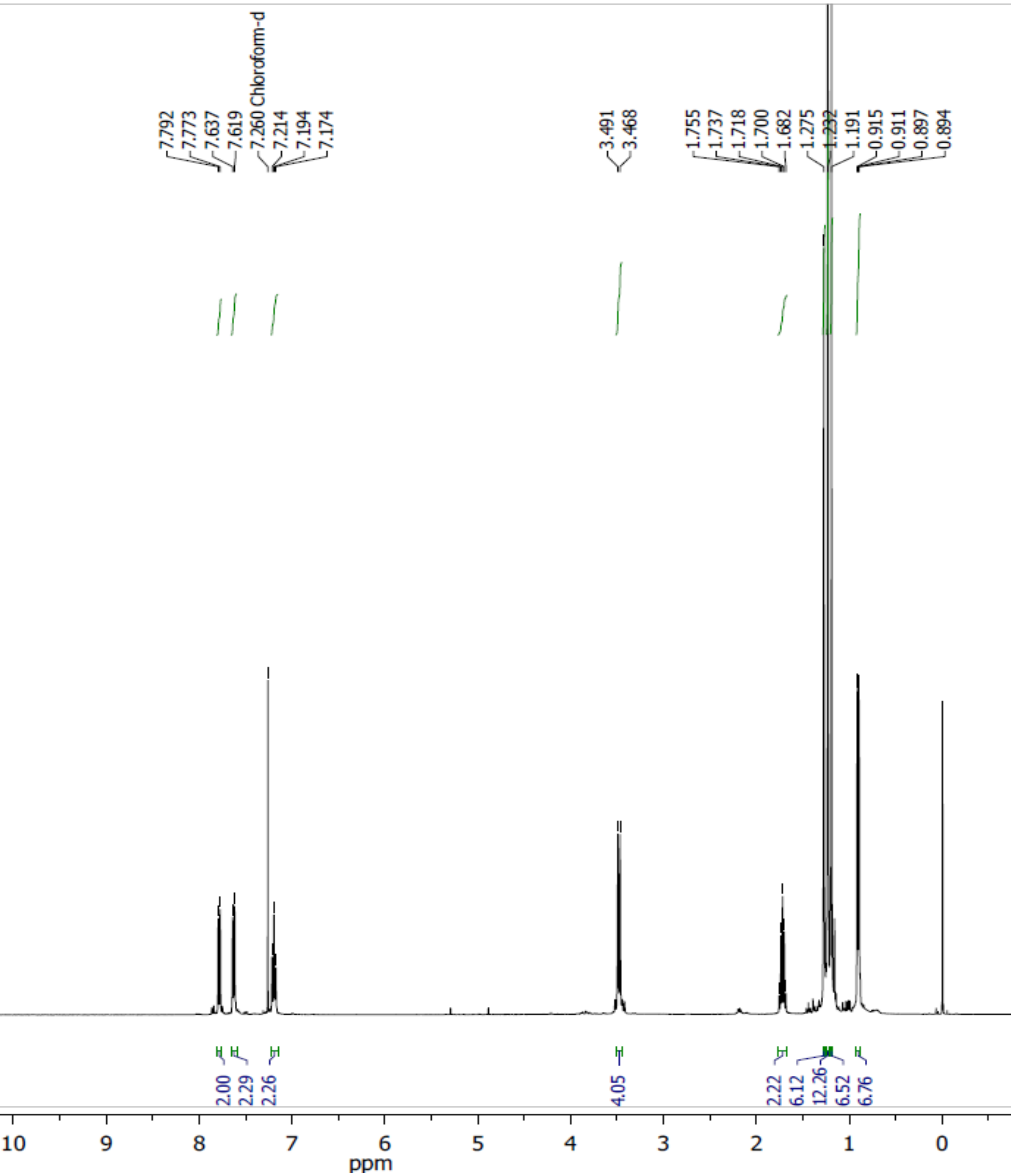

^{13}C NMR in $CDCl_3$

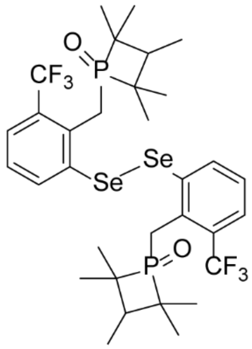

3d

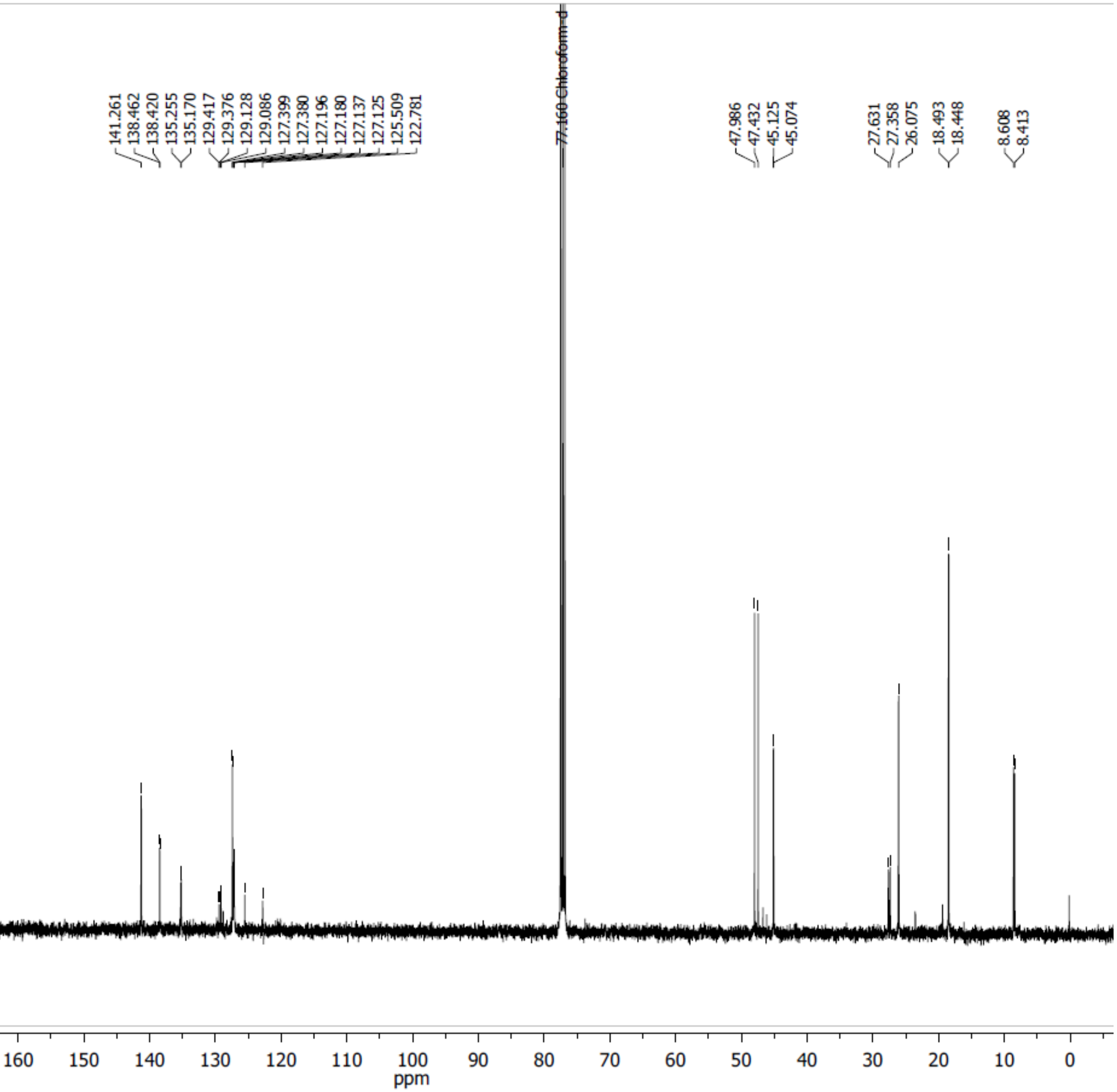

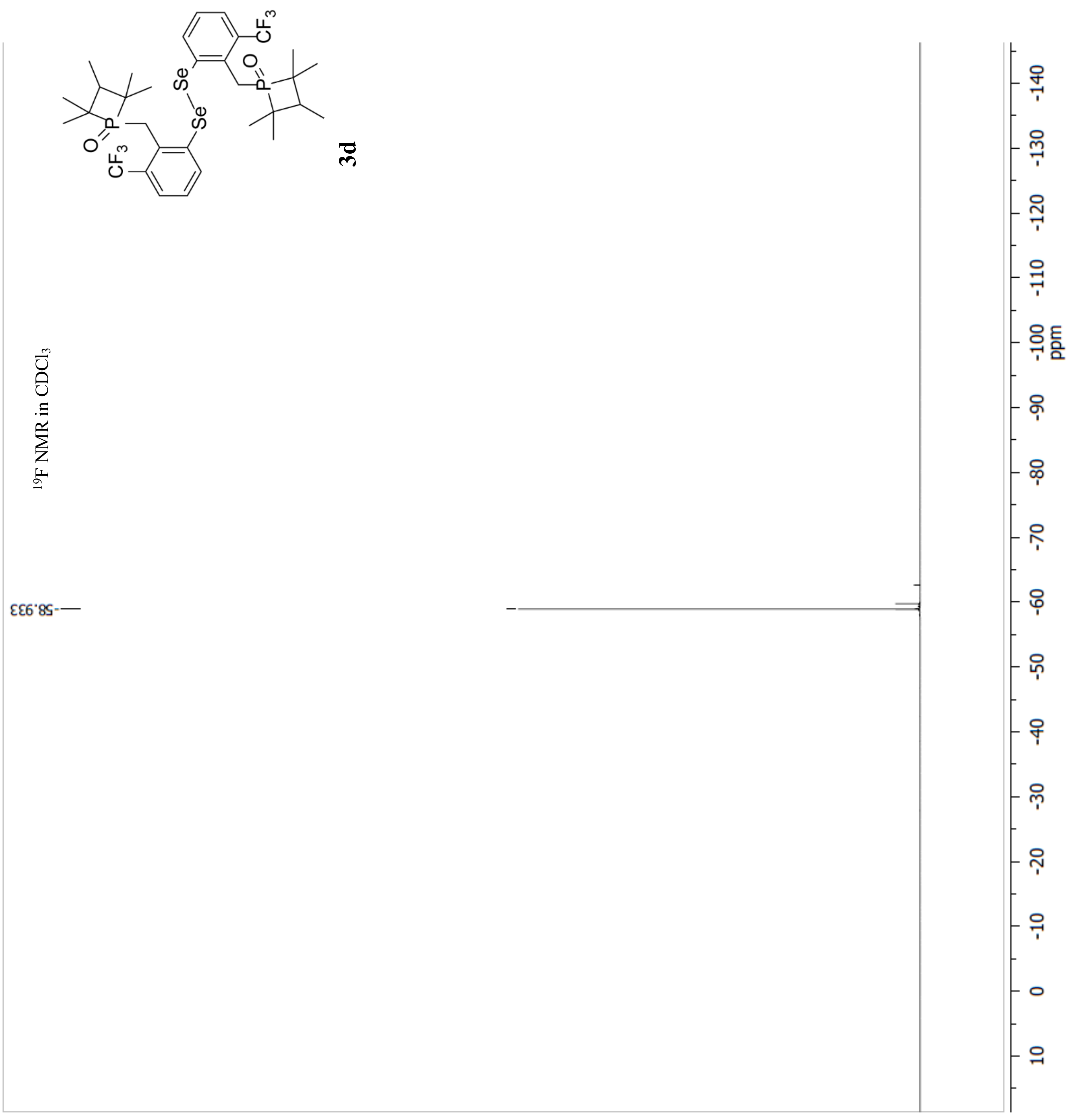

^{19}F NMR in $CDCl_3$
3d
-58.933
ppm
10 0 -10 -20 -30 -40 -50 -60 -70 -80 -90 -100 -110 -120 -130 -140

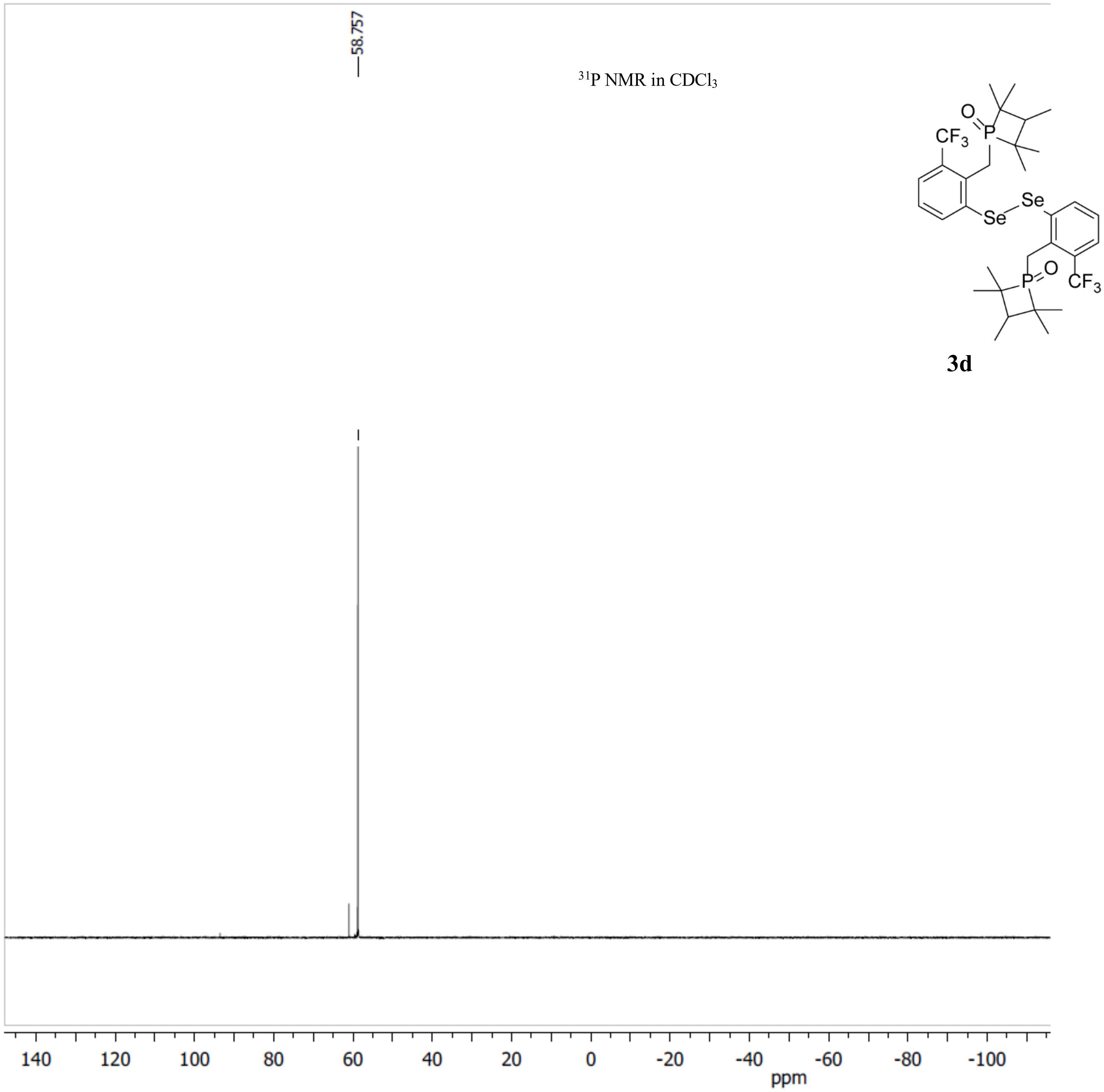

58.757
^{31}P NMR in $CDCl_3$
3d
140 120 100 80 60 40 20 0 -20 -40 -60 -80 -100
ppm

^{77}Se NMR in $CDCl_3$

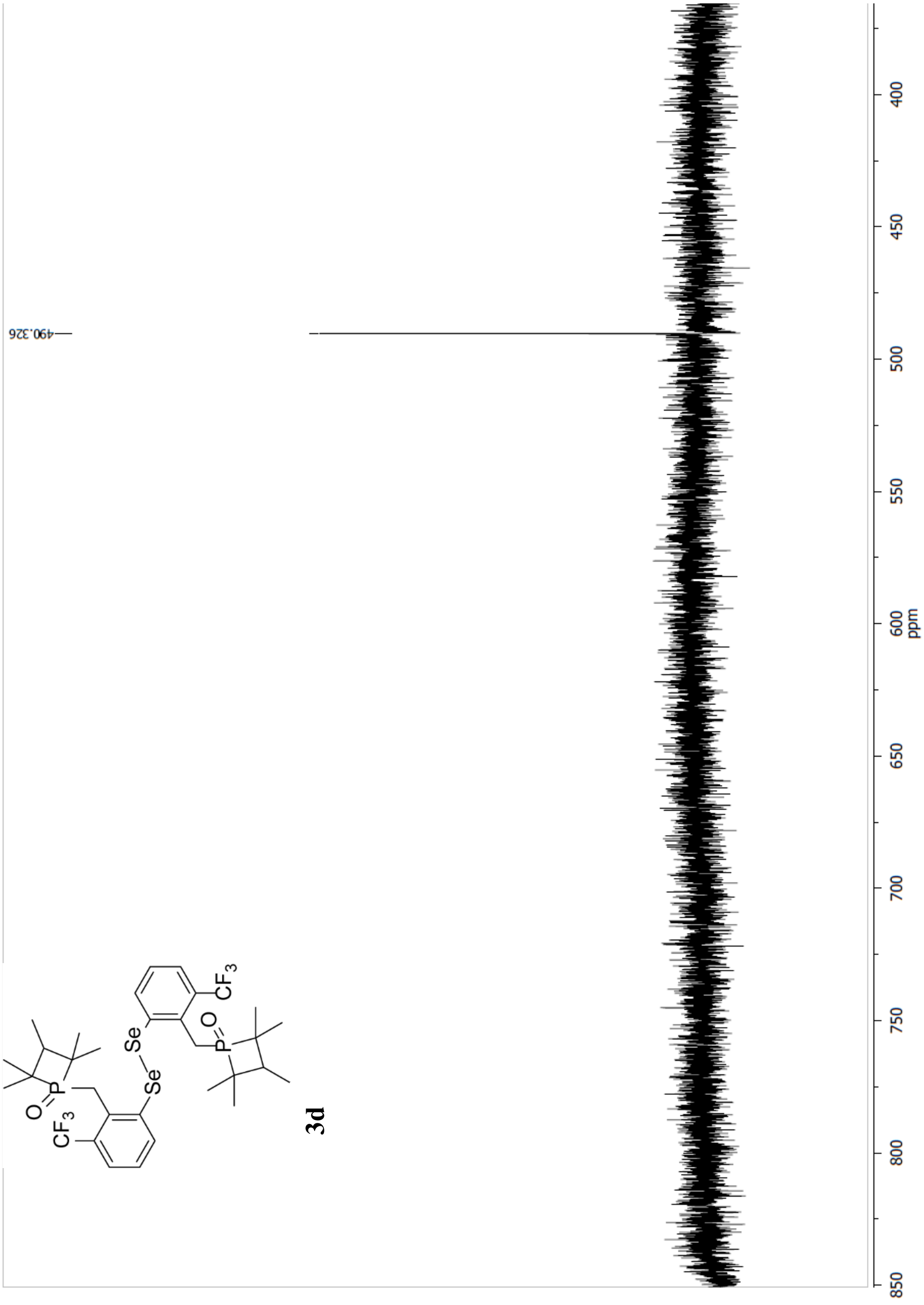

^{1}H NMR in $CDCl_3$

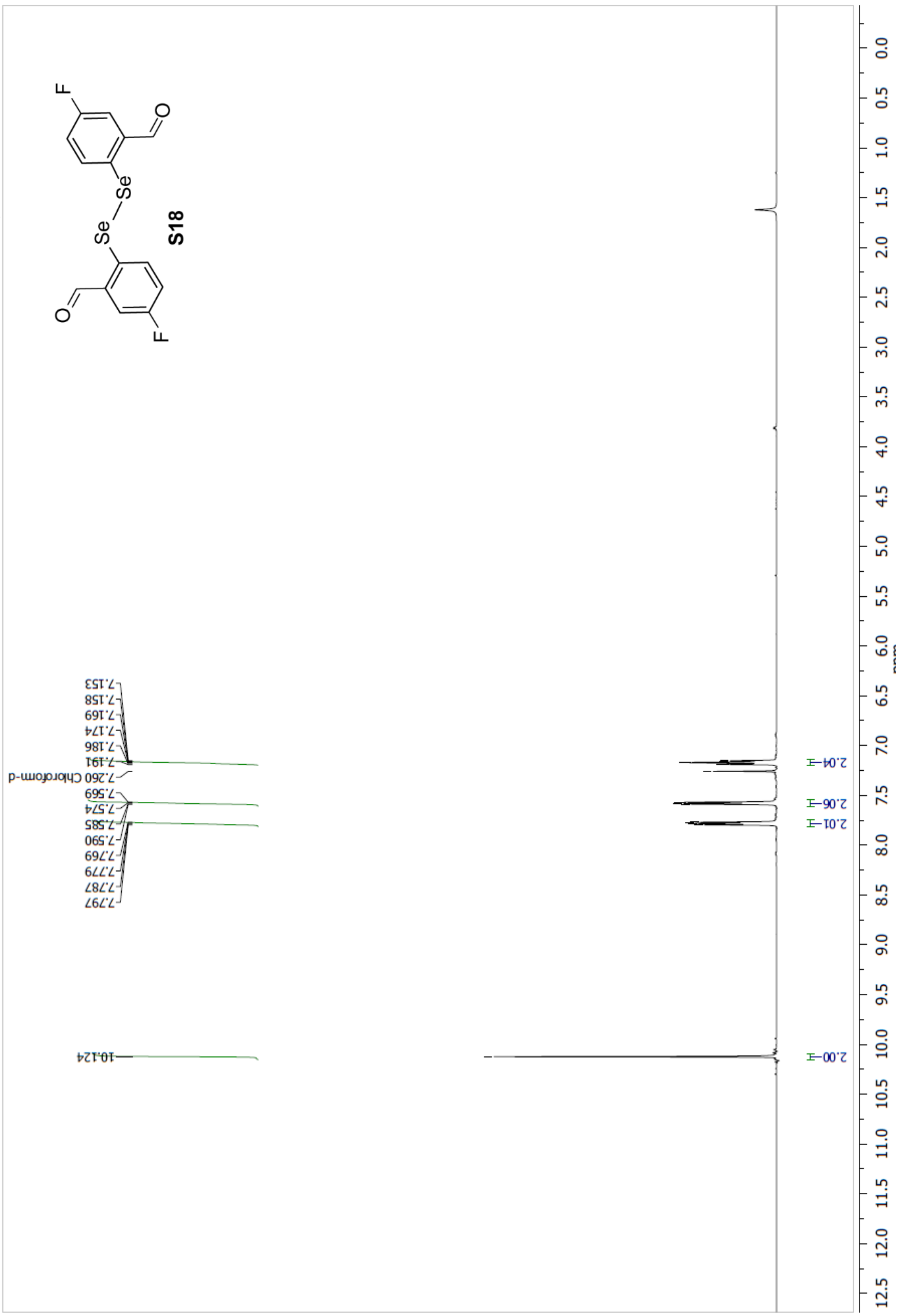

^{13}C NMR in $CDCl_3$

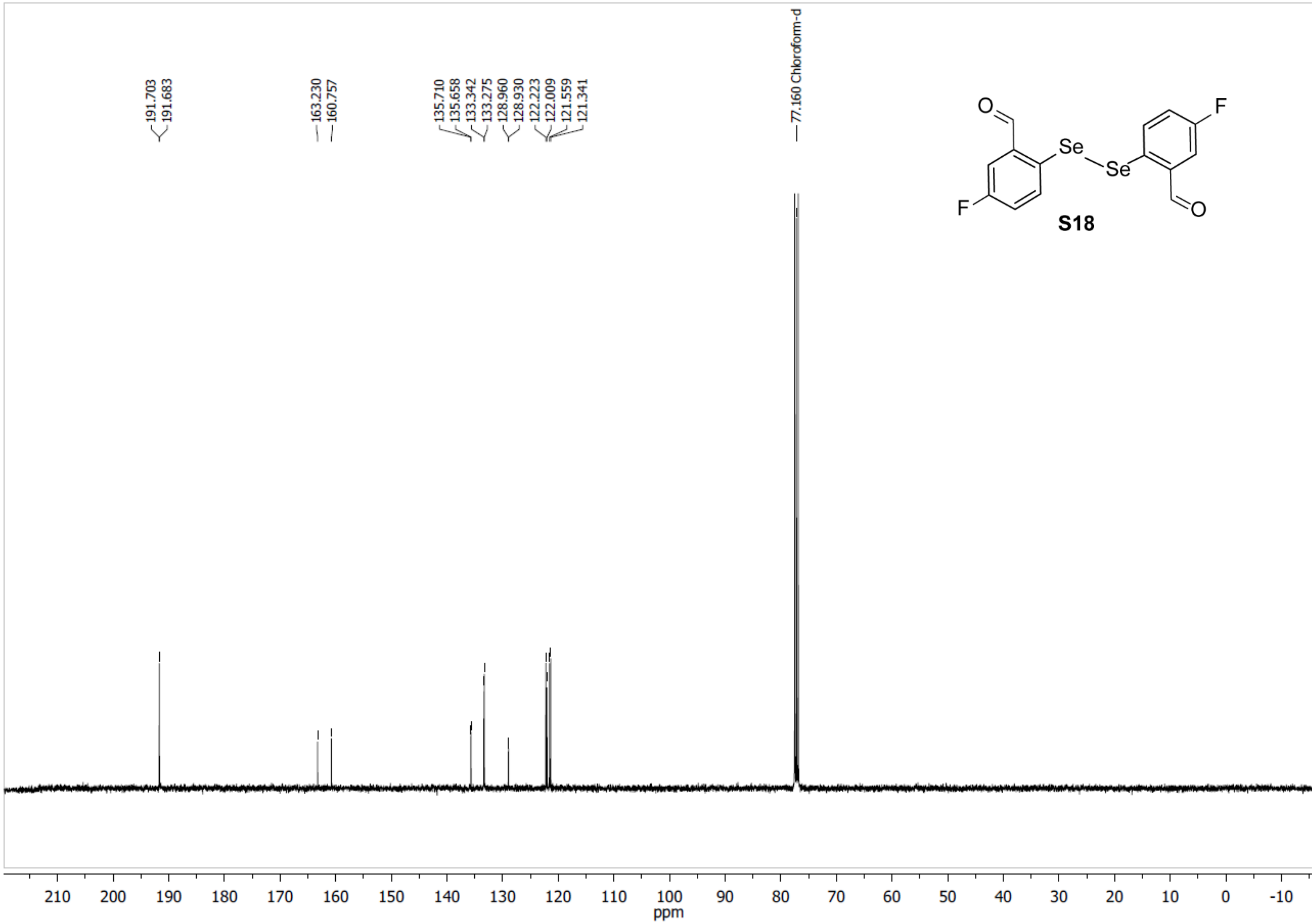

^{19}F NMR in $CDCl_3$

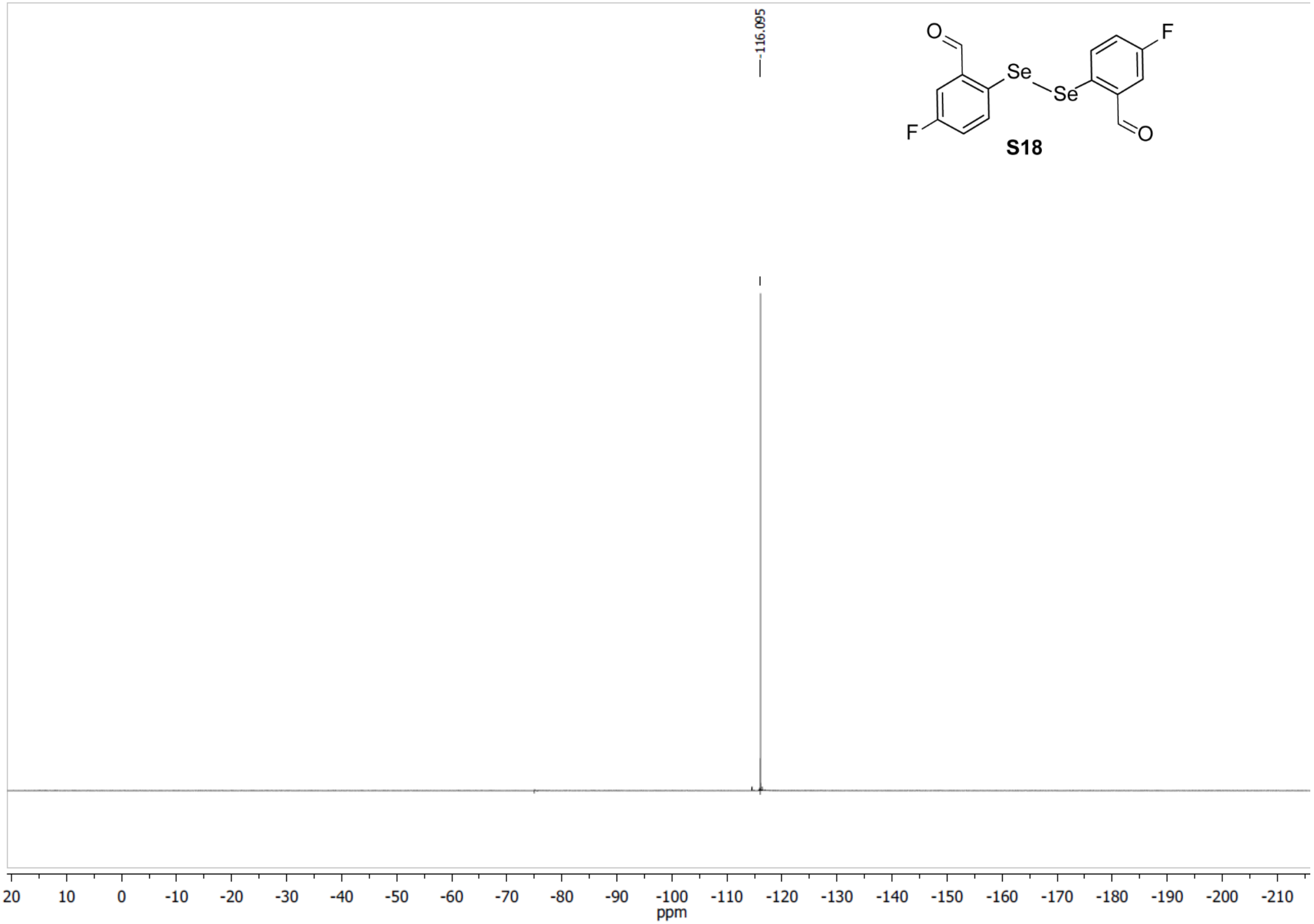

^{77}Se NMR in $CDCl_3$

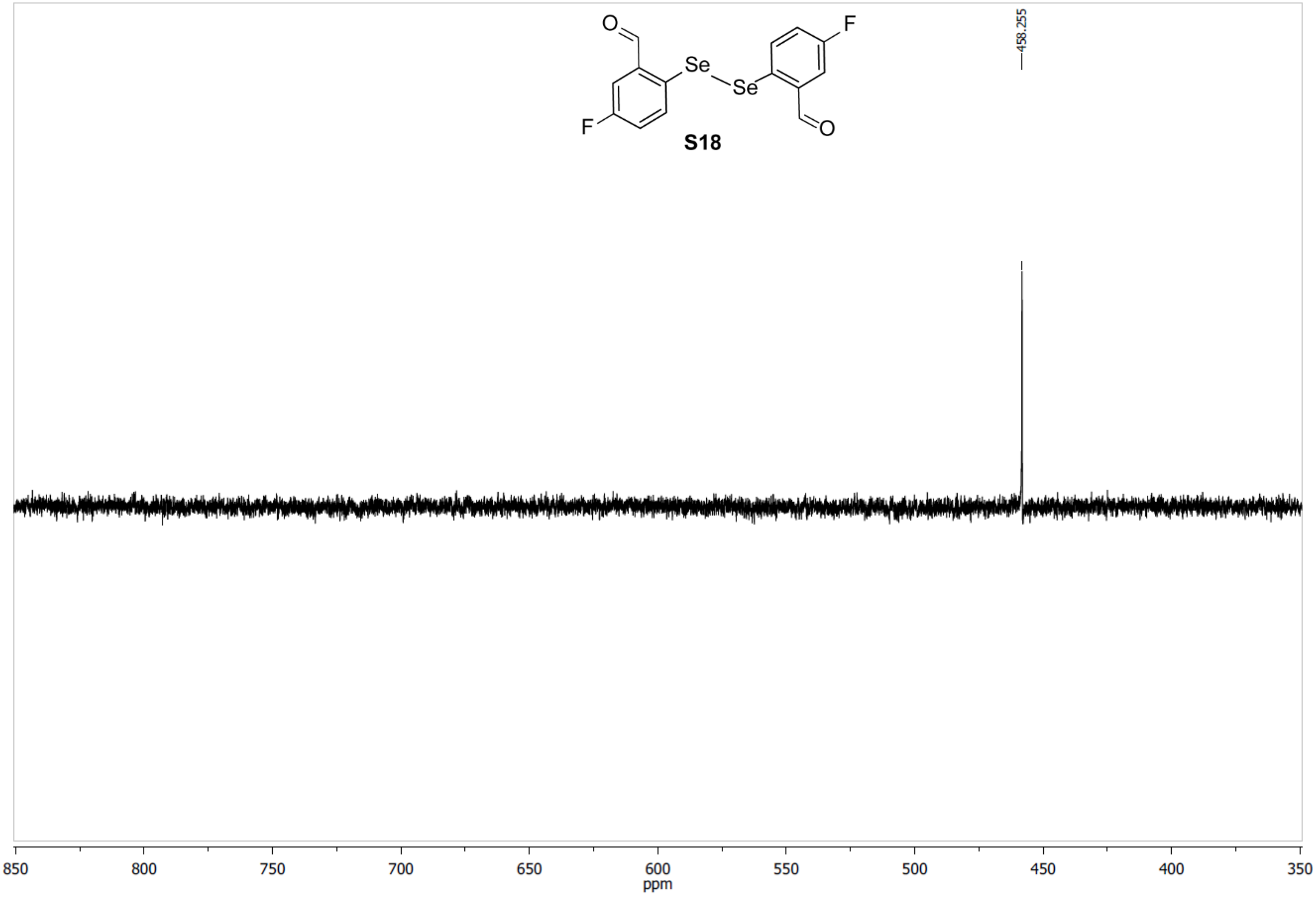

^{1}H NMR in DMSO-d_6

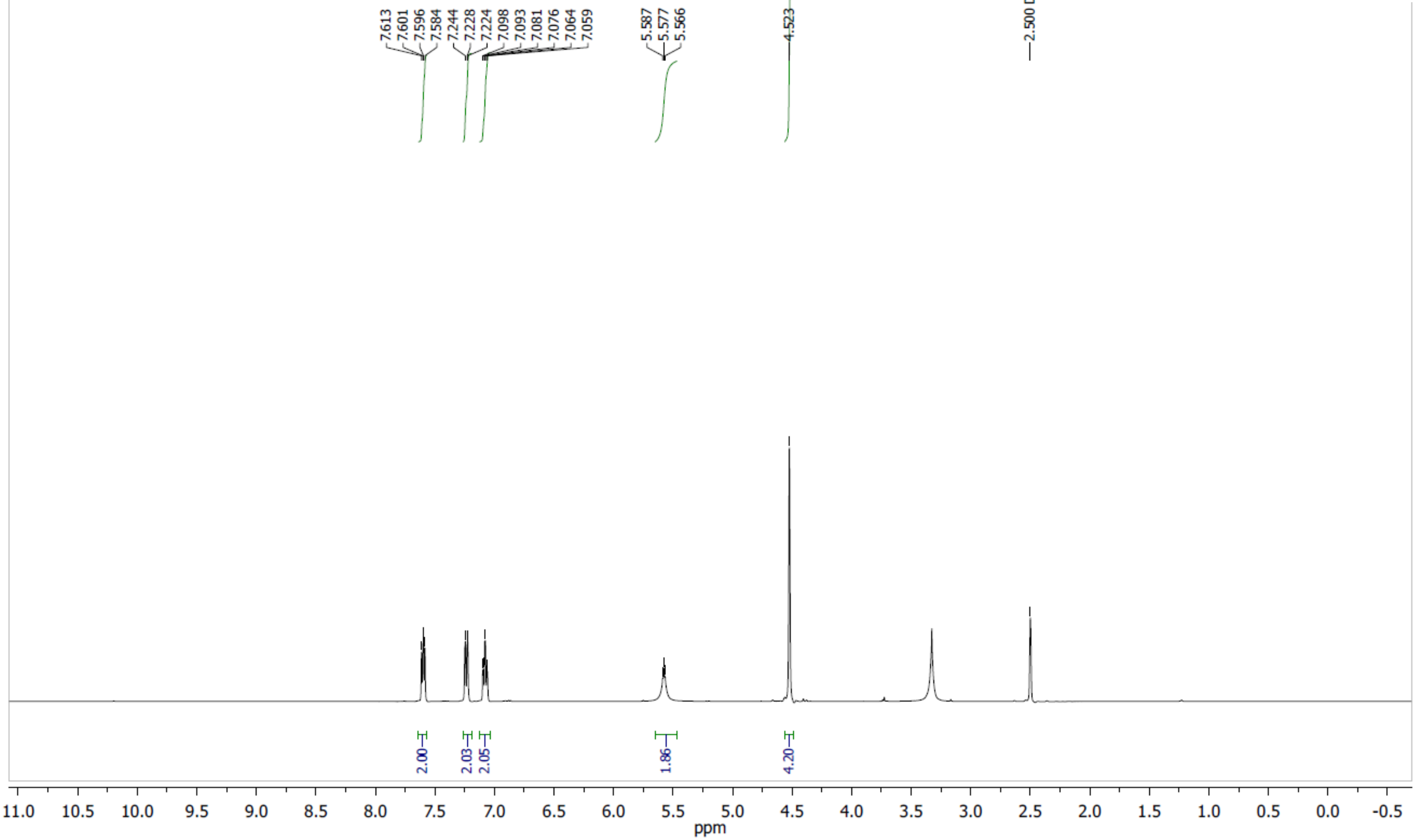

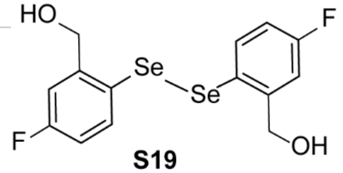

S19

^{13}C NMR in DMSO-d_6

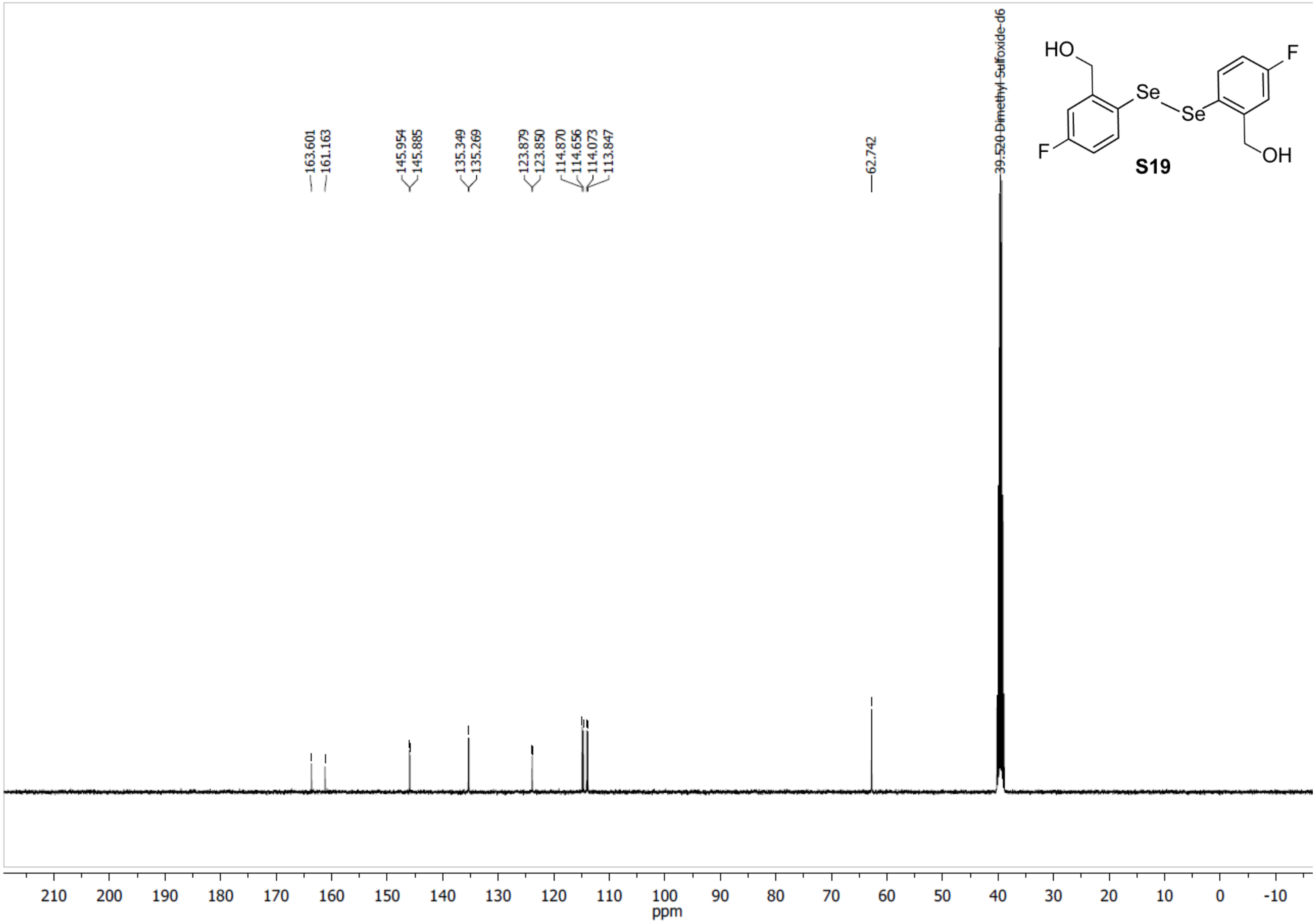

^{19}F NMR in DMSO-d_6

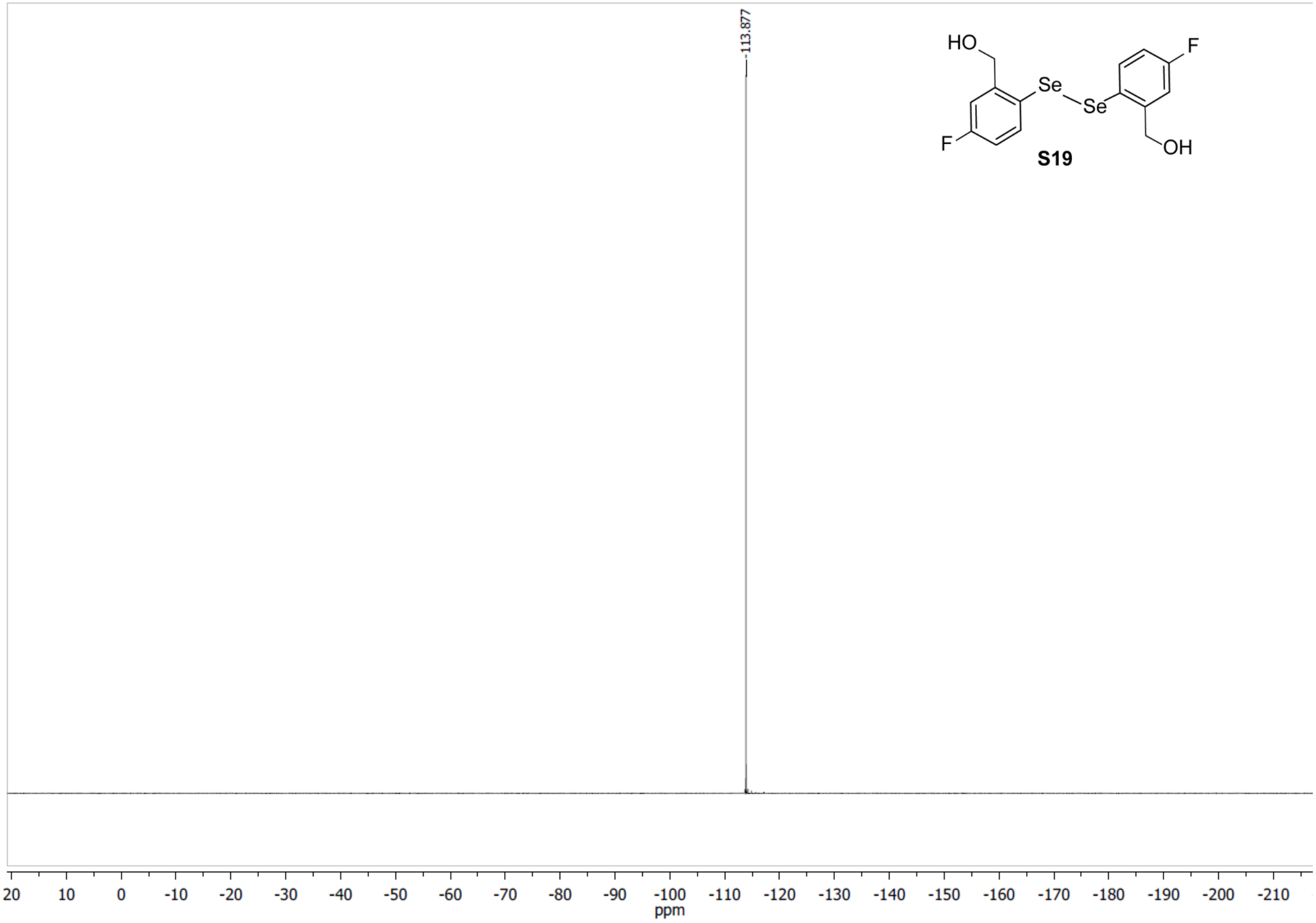

^{77}Se NMR in DMSO-d$_6$

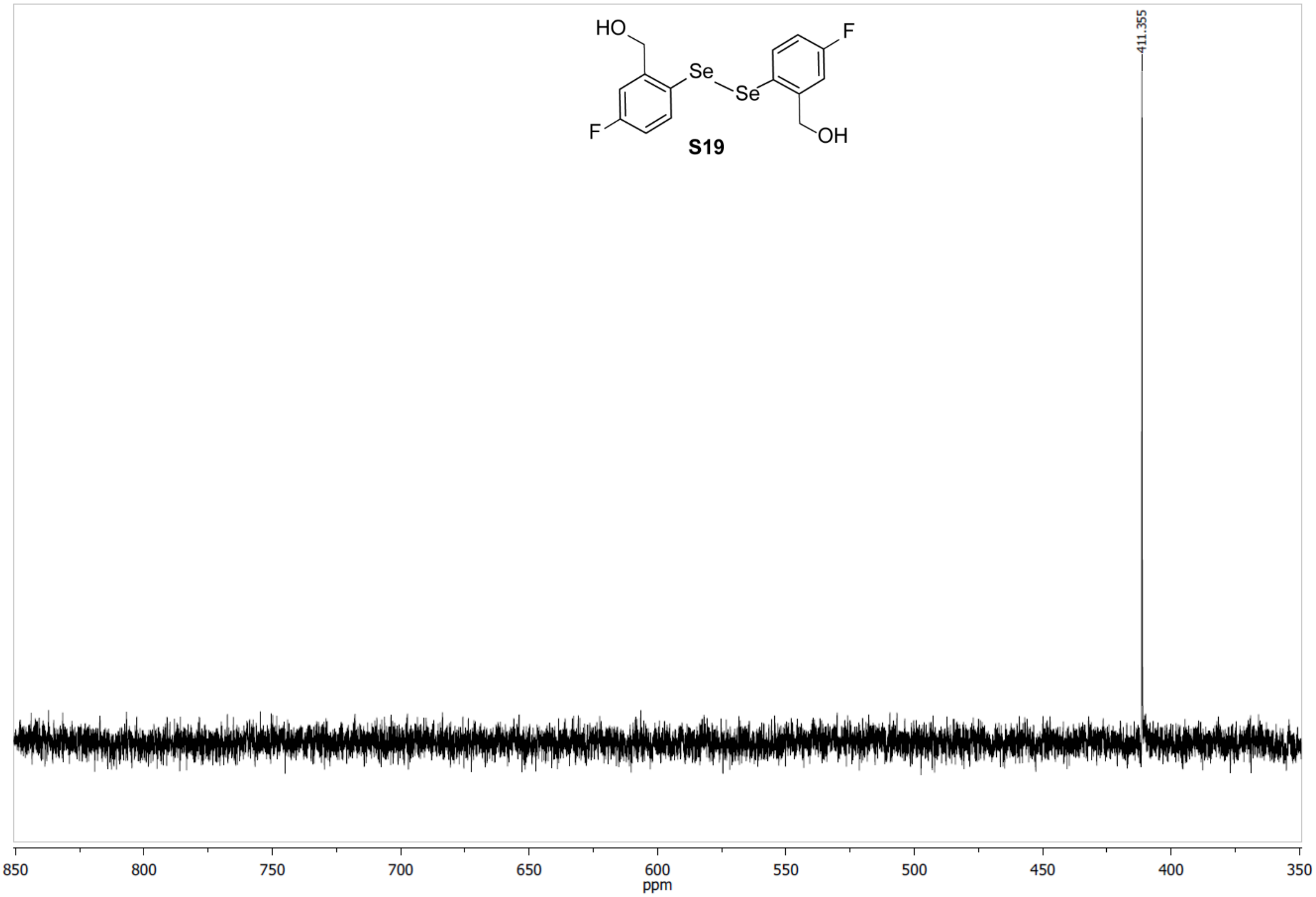

^1H NMR in $CDCl_3$

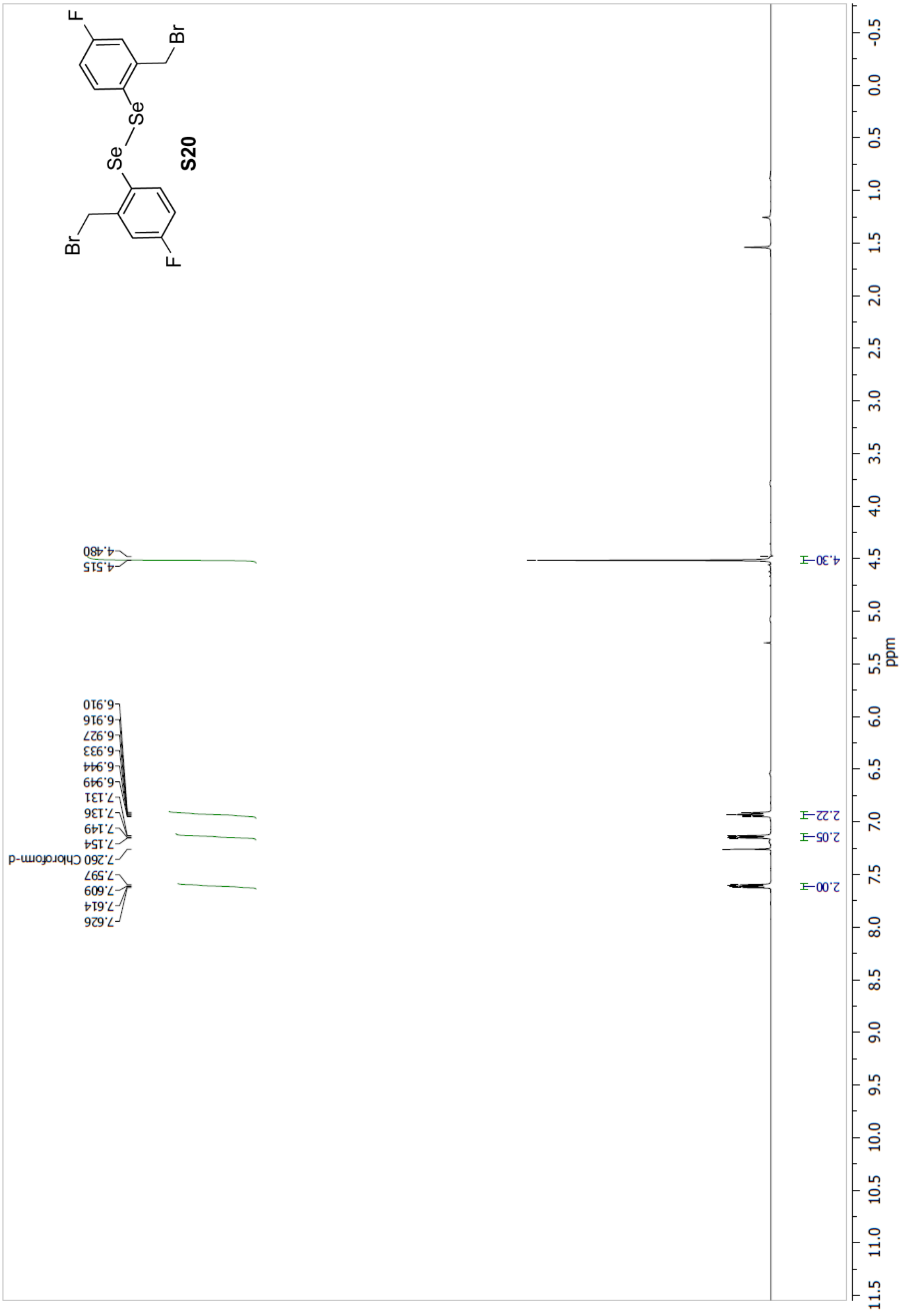

^{13}C NMR in CDCl$_3$

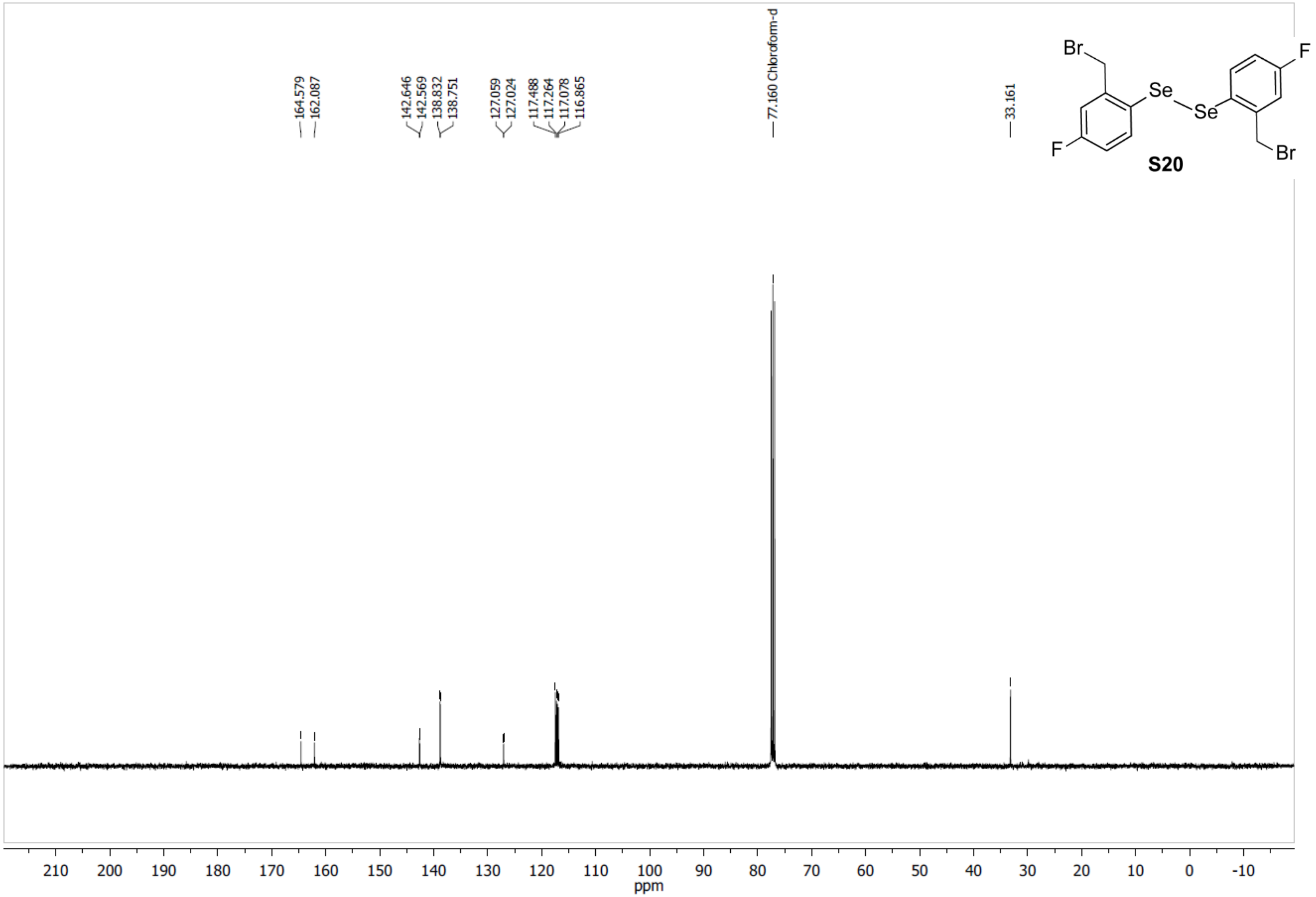

^{19}F NMR in $CDCl_3$

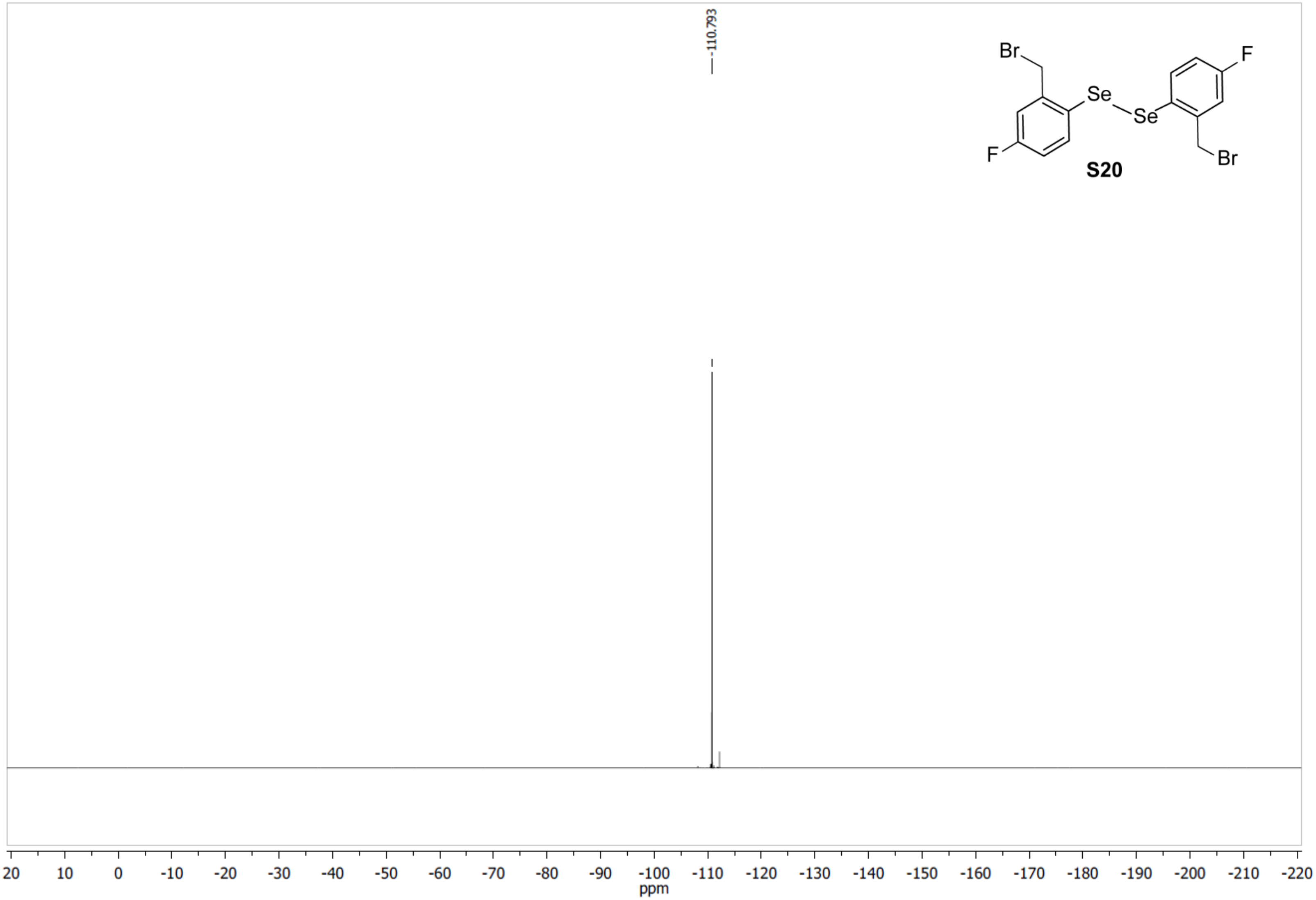

^{77}Se NMR in $CDCl_3$

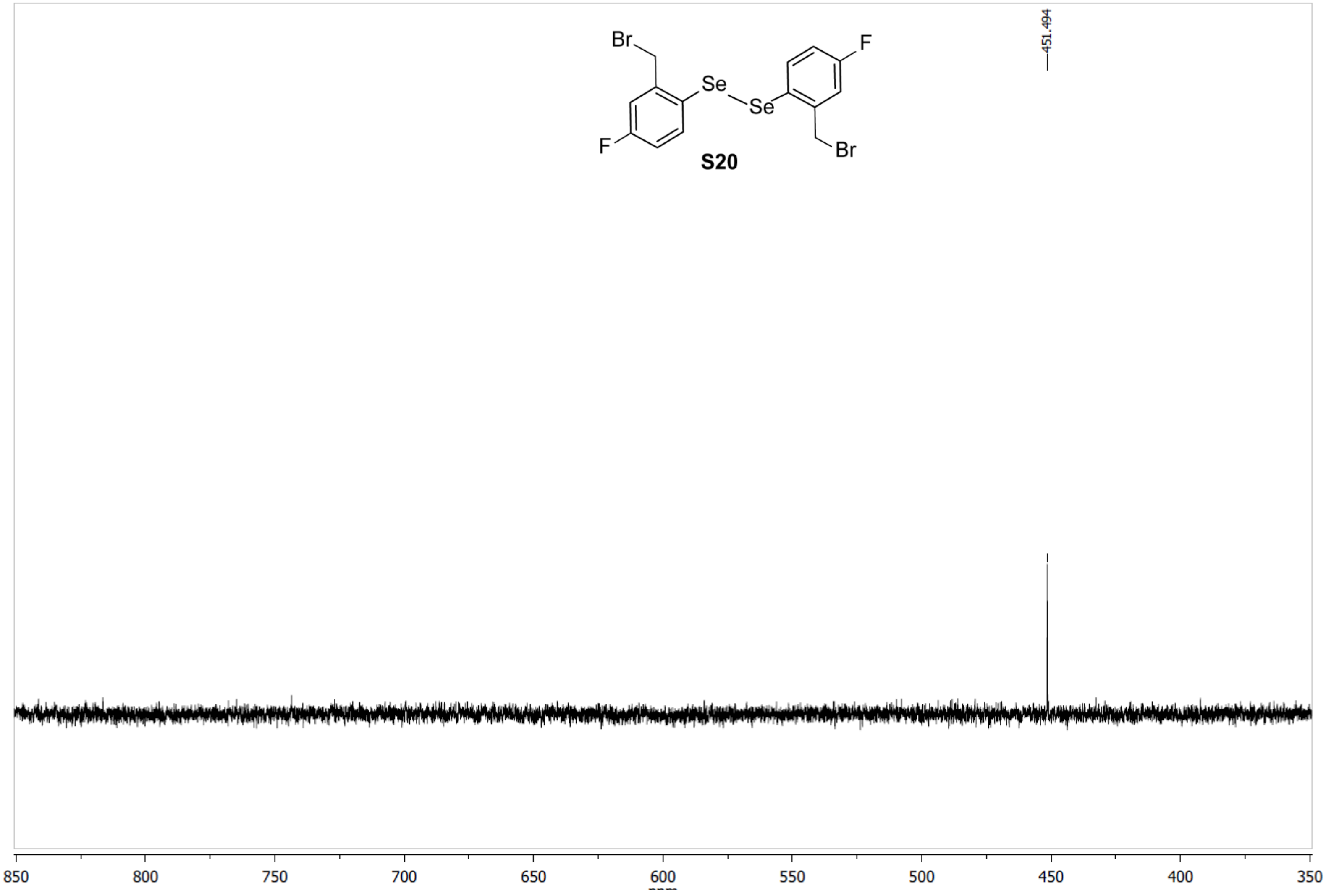

S20

^{1}H NMR in $CDCl_3$

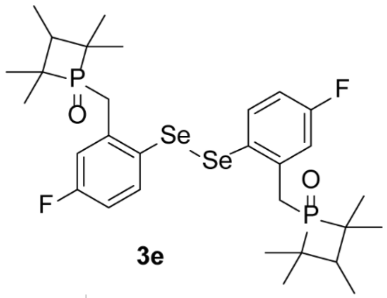

3e

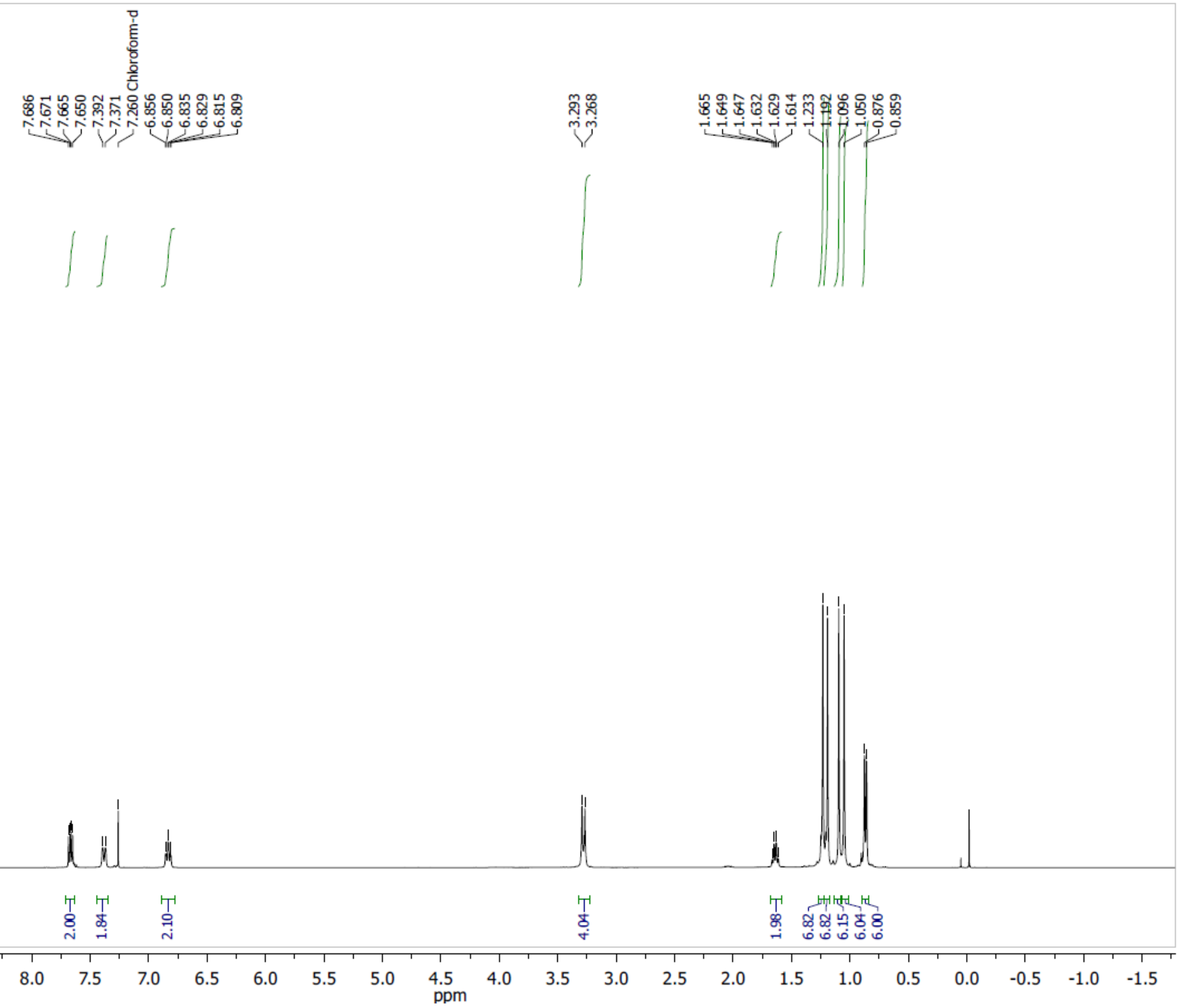

^{13}C NMR in $CDCl_3$

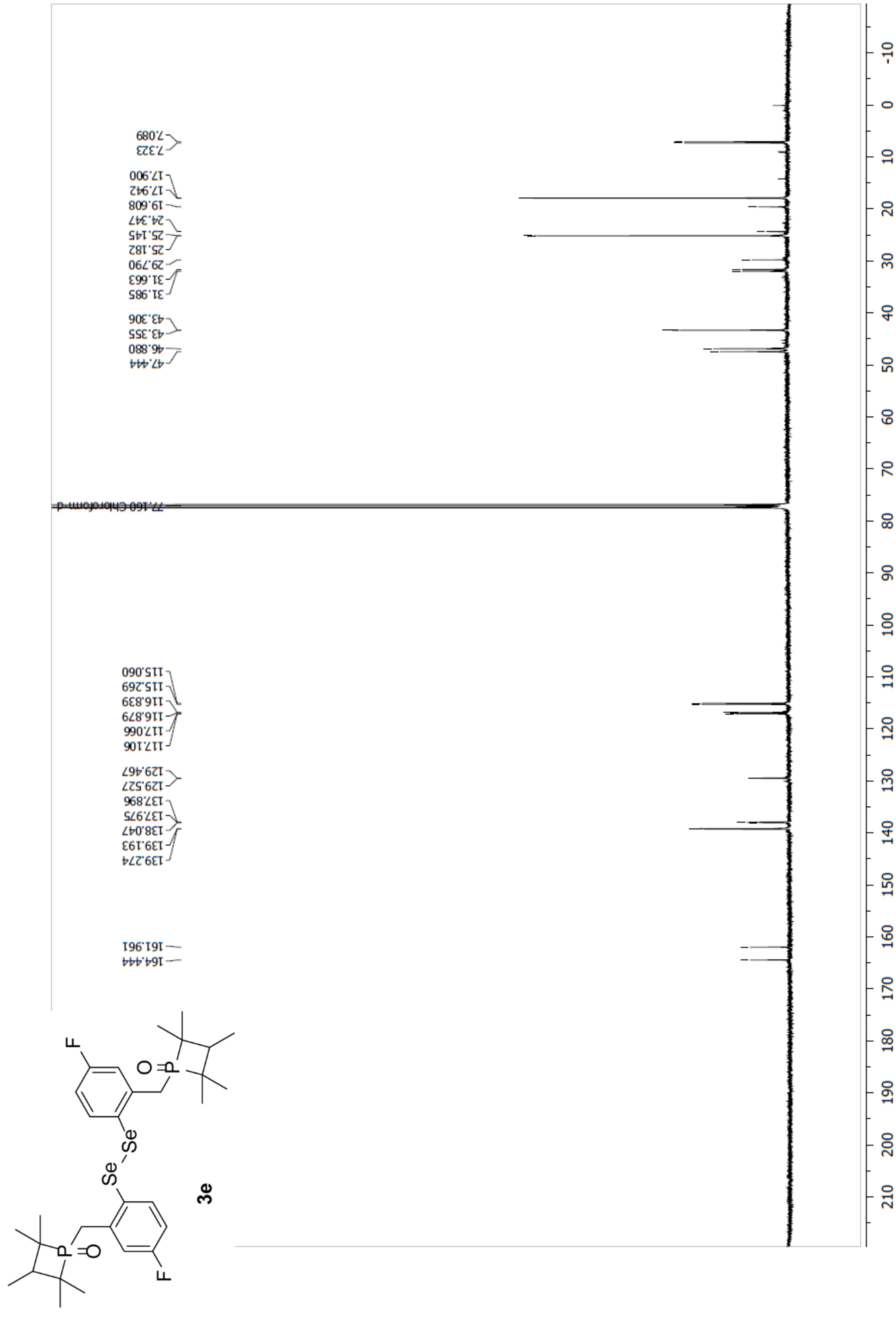

3e

^{19}F NMR in $CDCl_3$

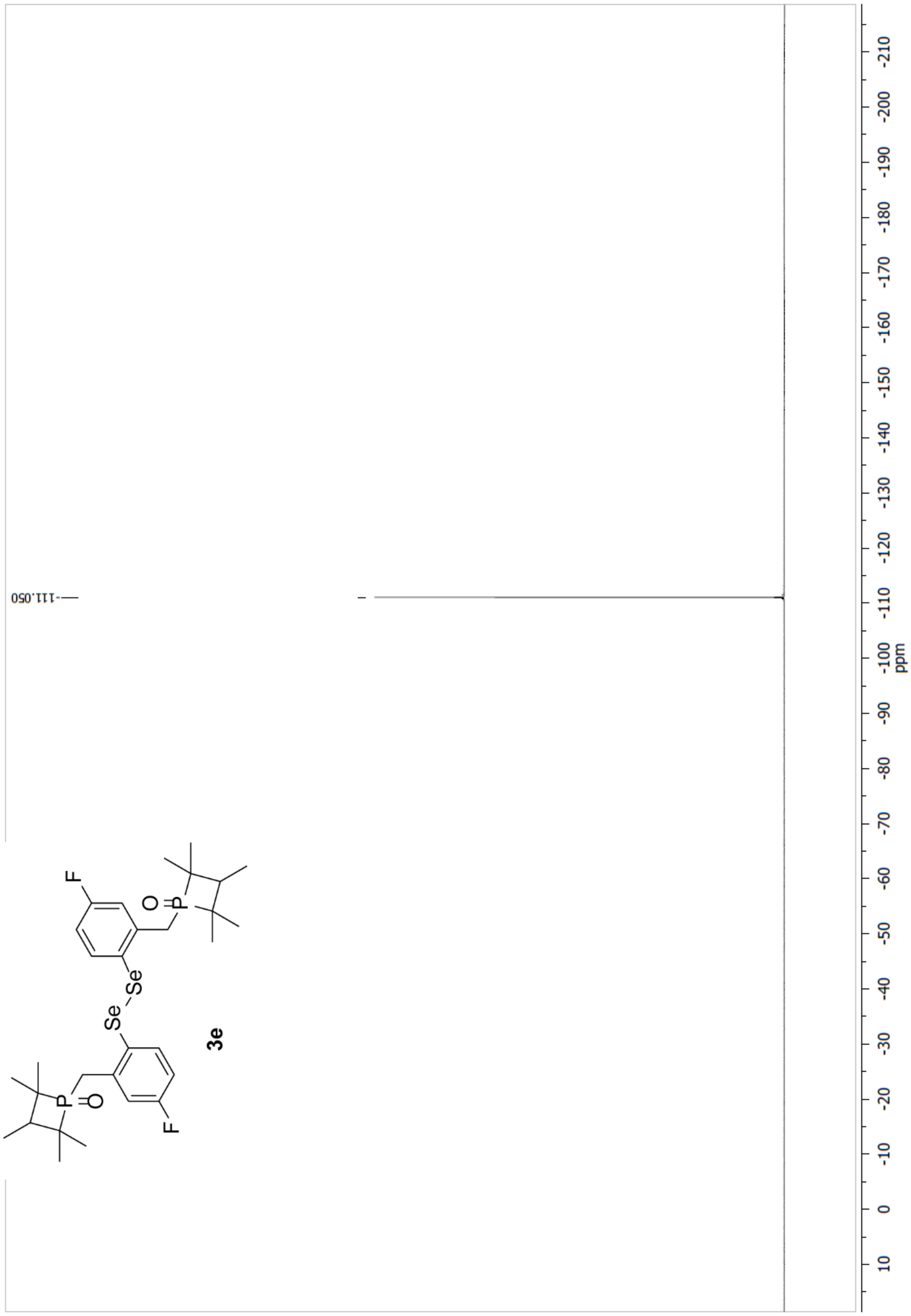

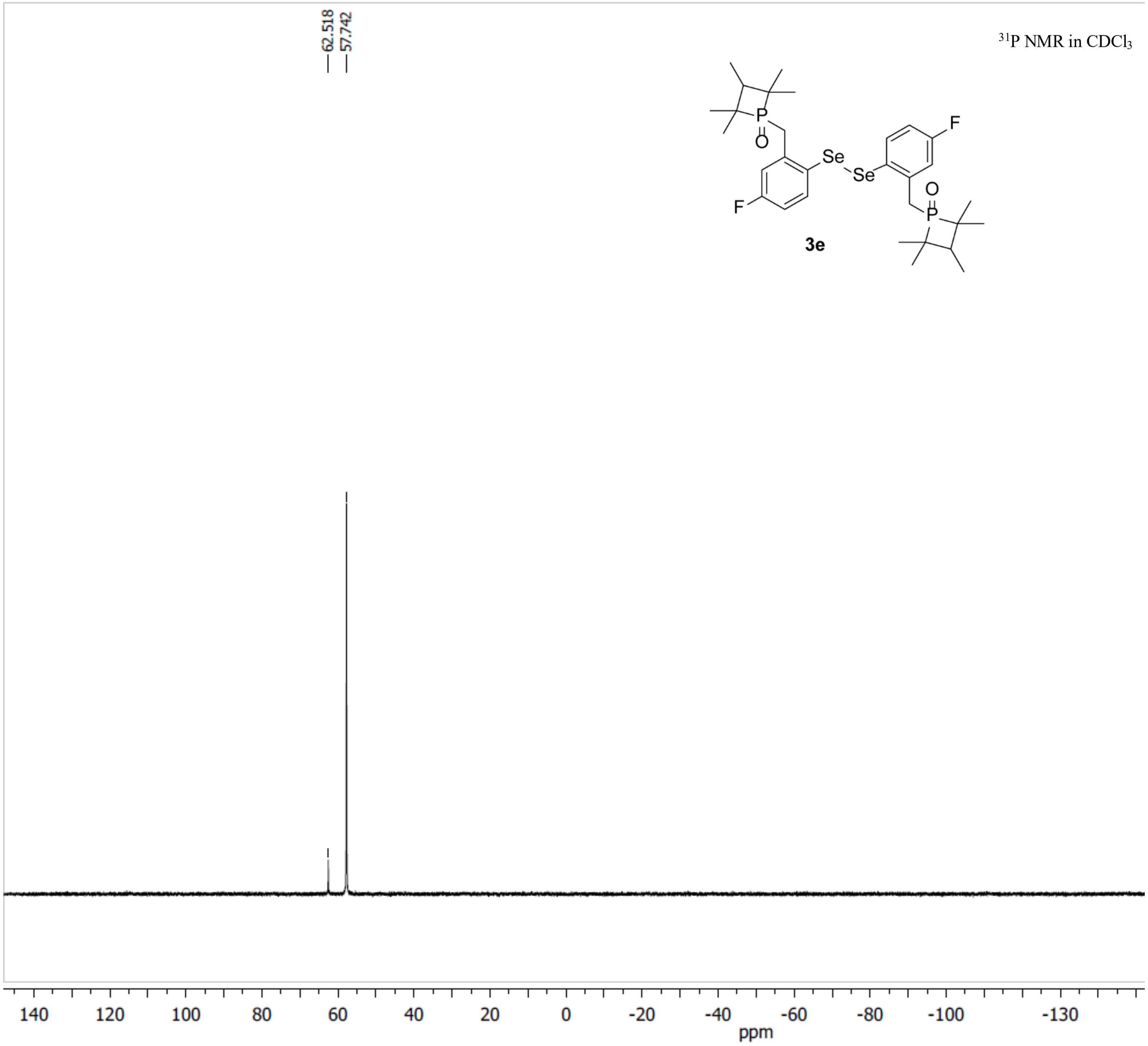

31P NMR in CDCl3
3e
62.518
57.742
140
120
100
80
60
40
20
0
-20
-40
-60
-80
-100
-130
ppm

^{77}Se NMR in $CDCl_3$

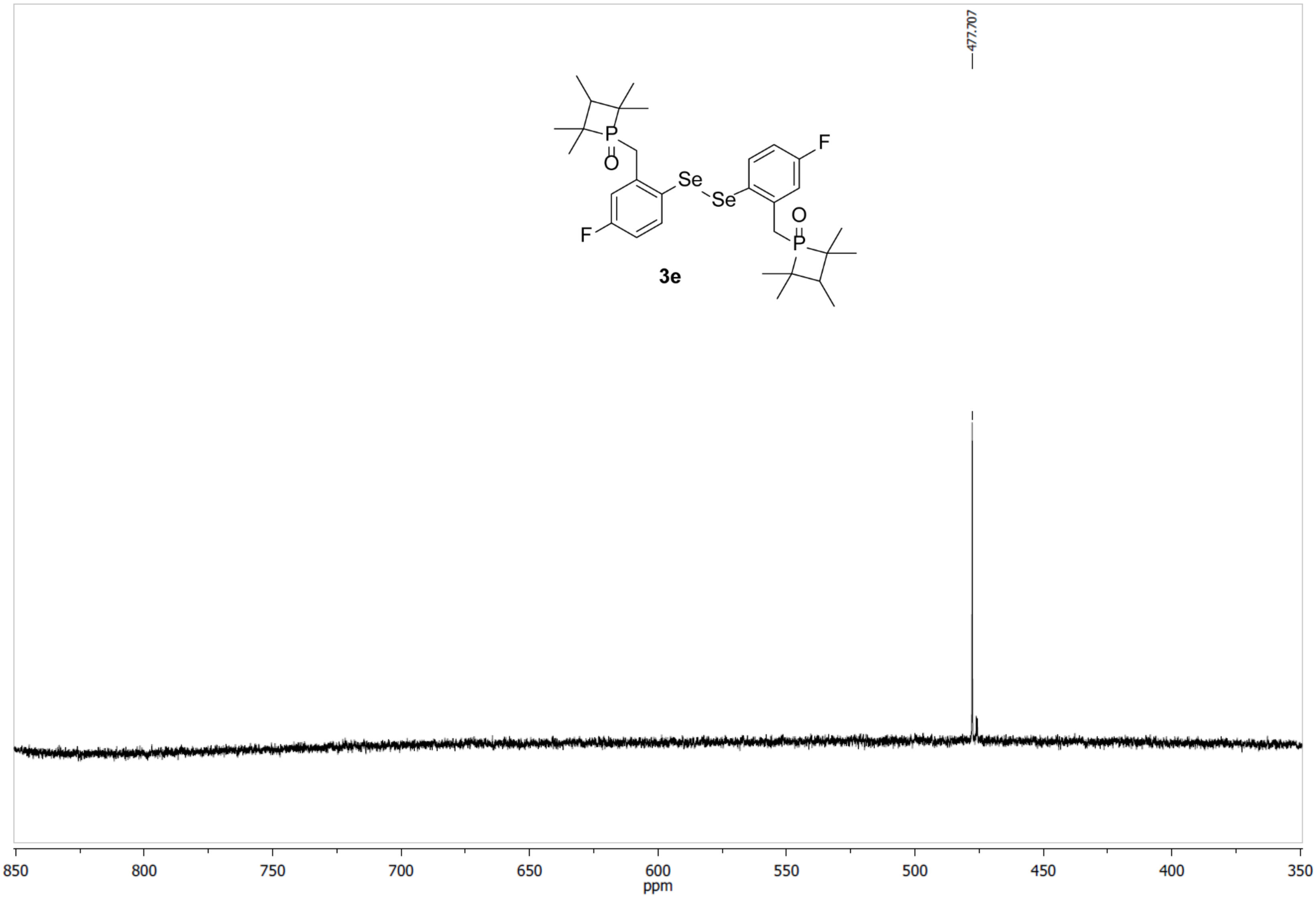

^{1}H NMR in CDCl$_3$

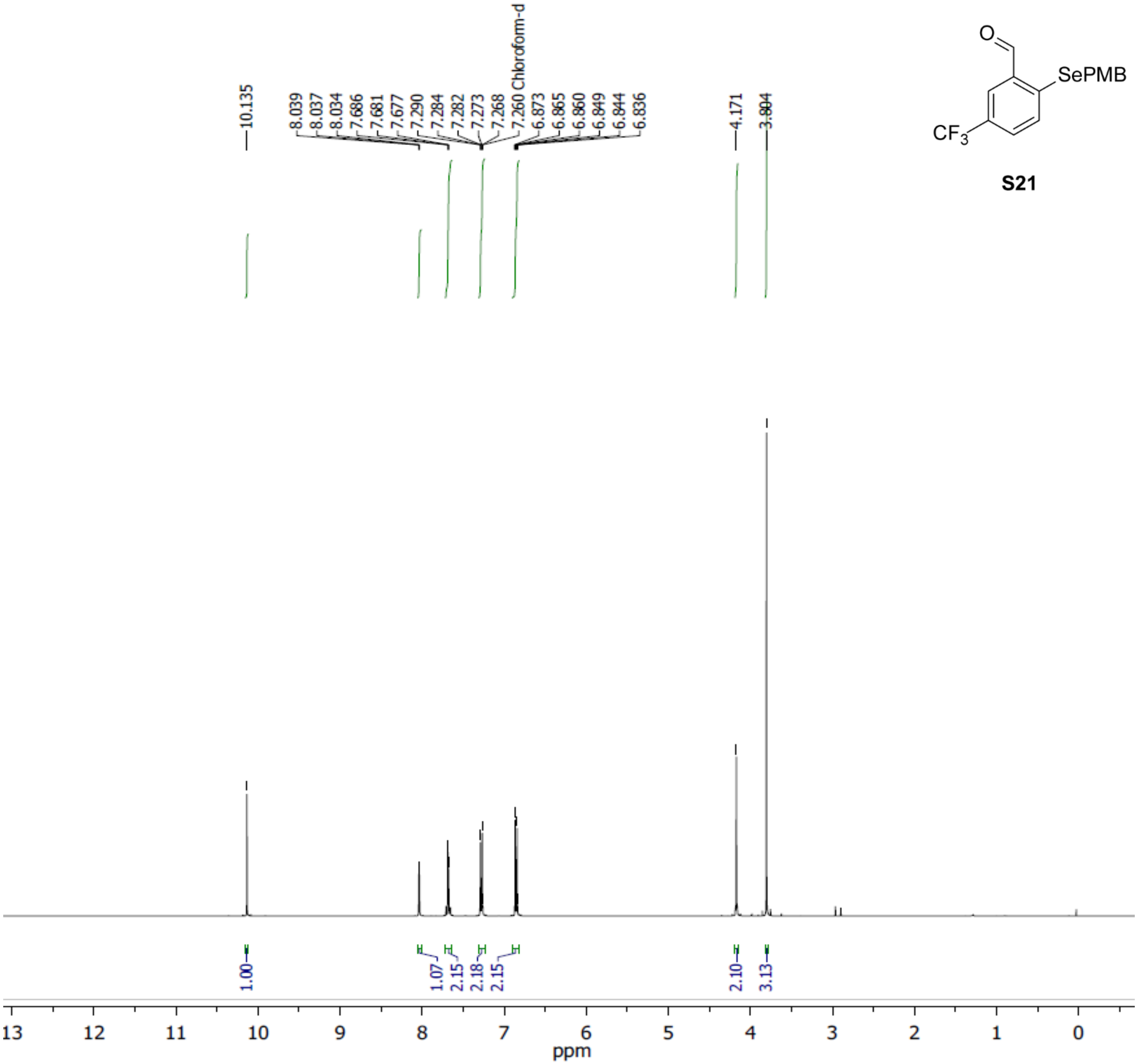

^{13}C NMR in $CDCl_3$

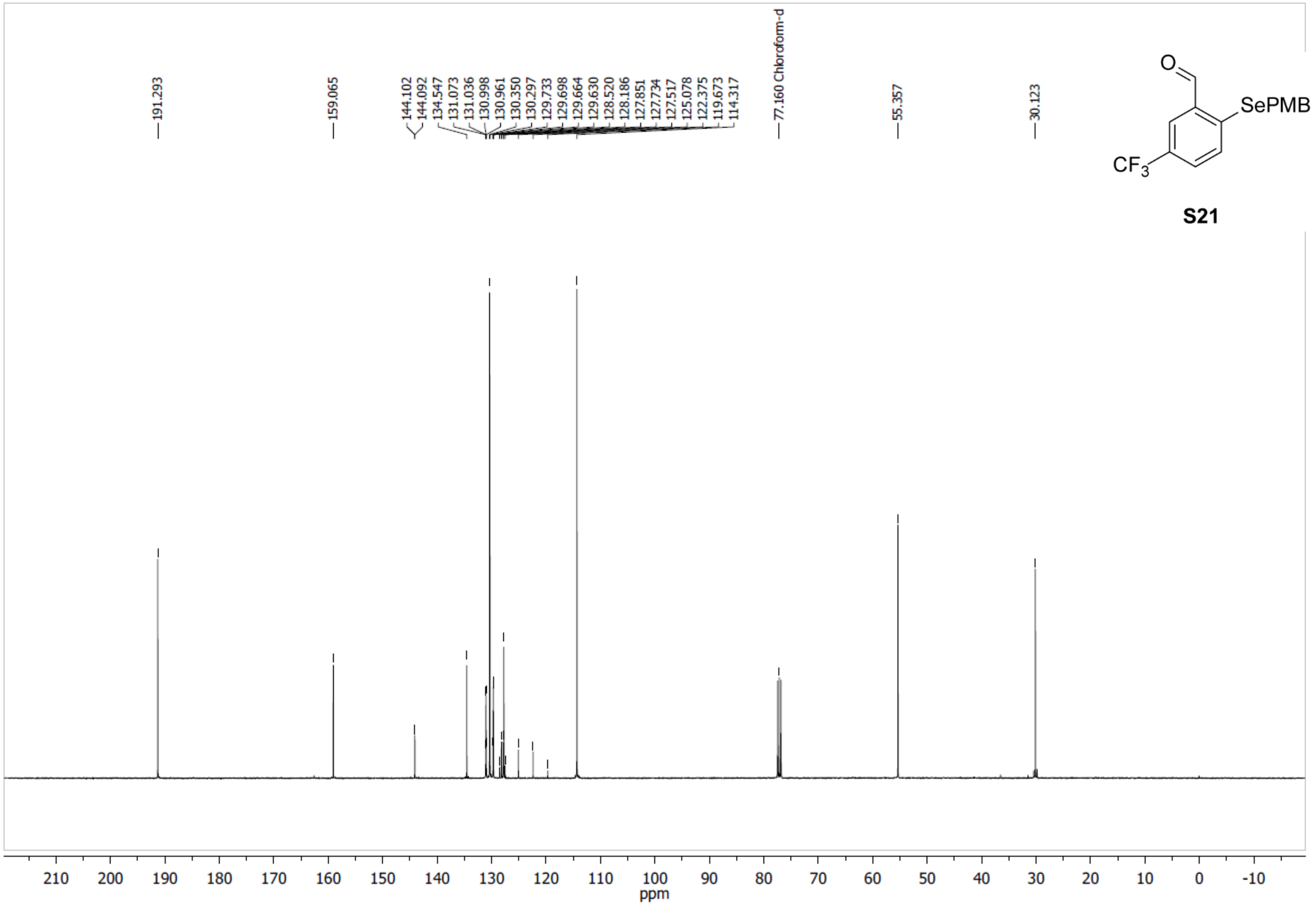

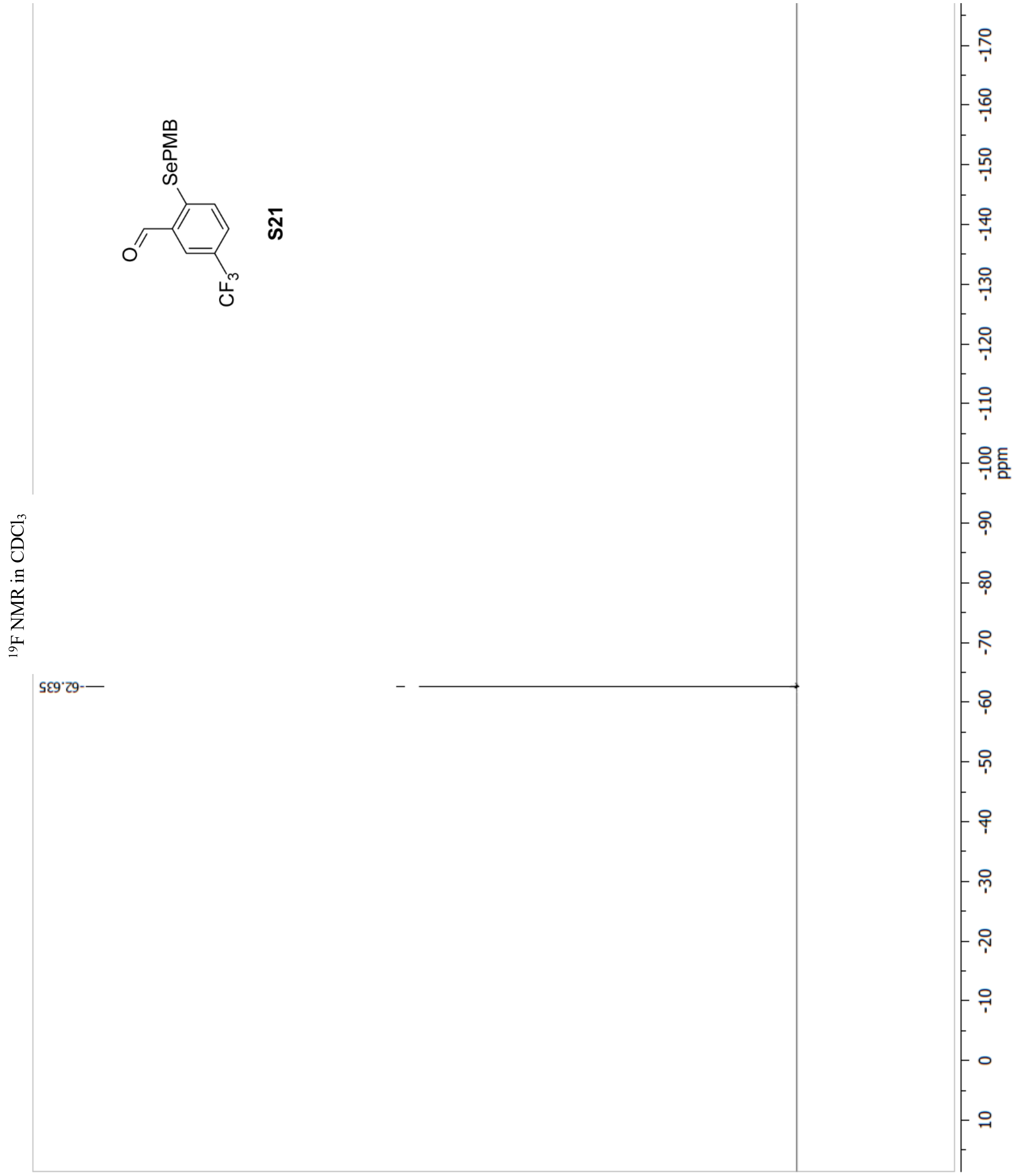

^{19}F NMR in CDCl$_3$
SePMB
O
CF$_3$
S21
-62.635
ppm

^{77}Se NMR in $CDCl_3$

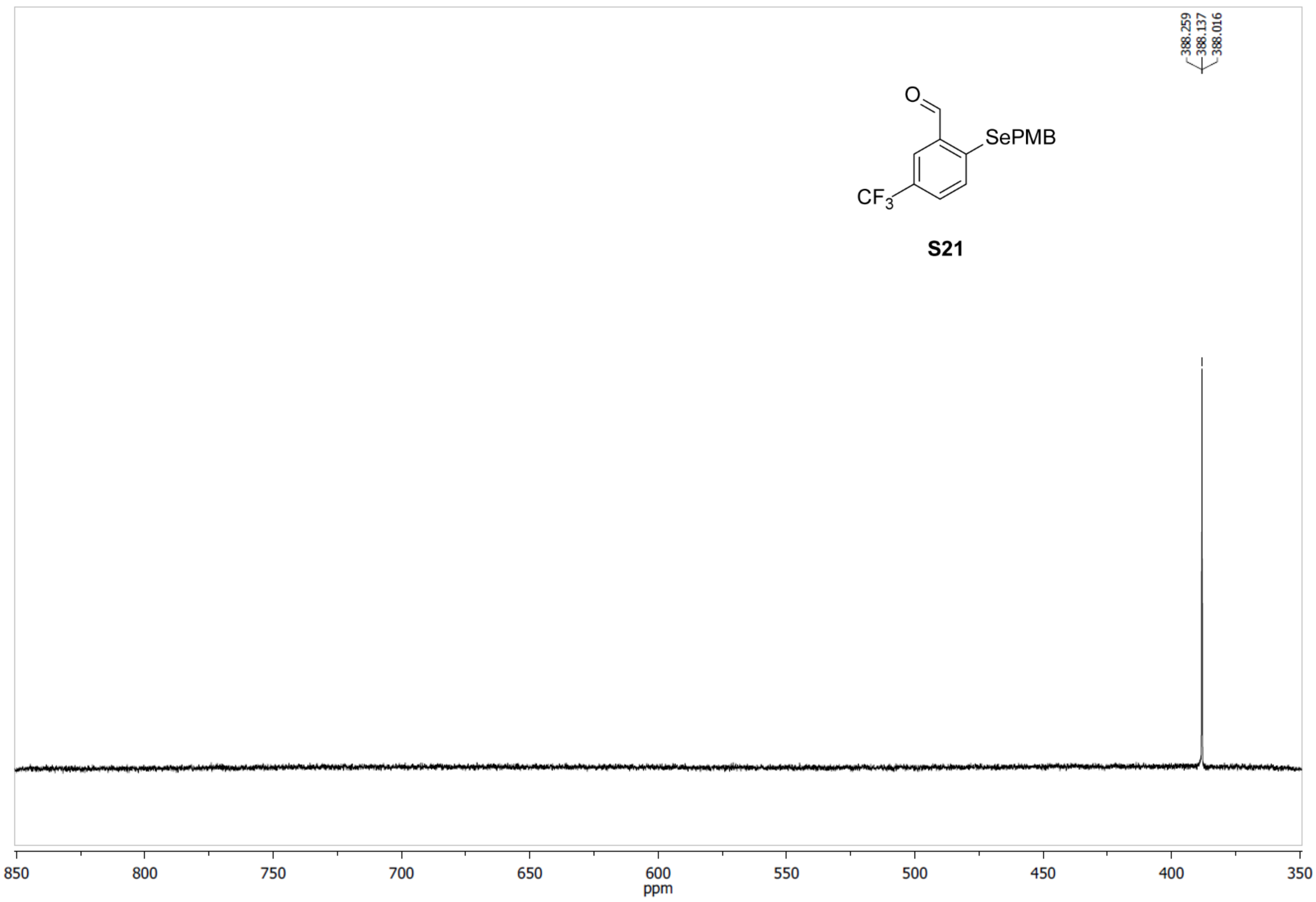

^{1}H NMR in $CDCl_3$

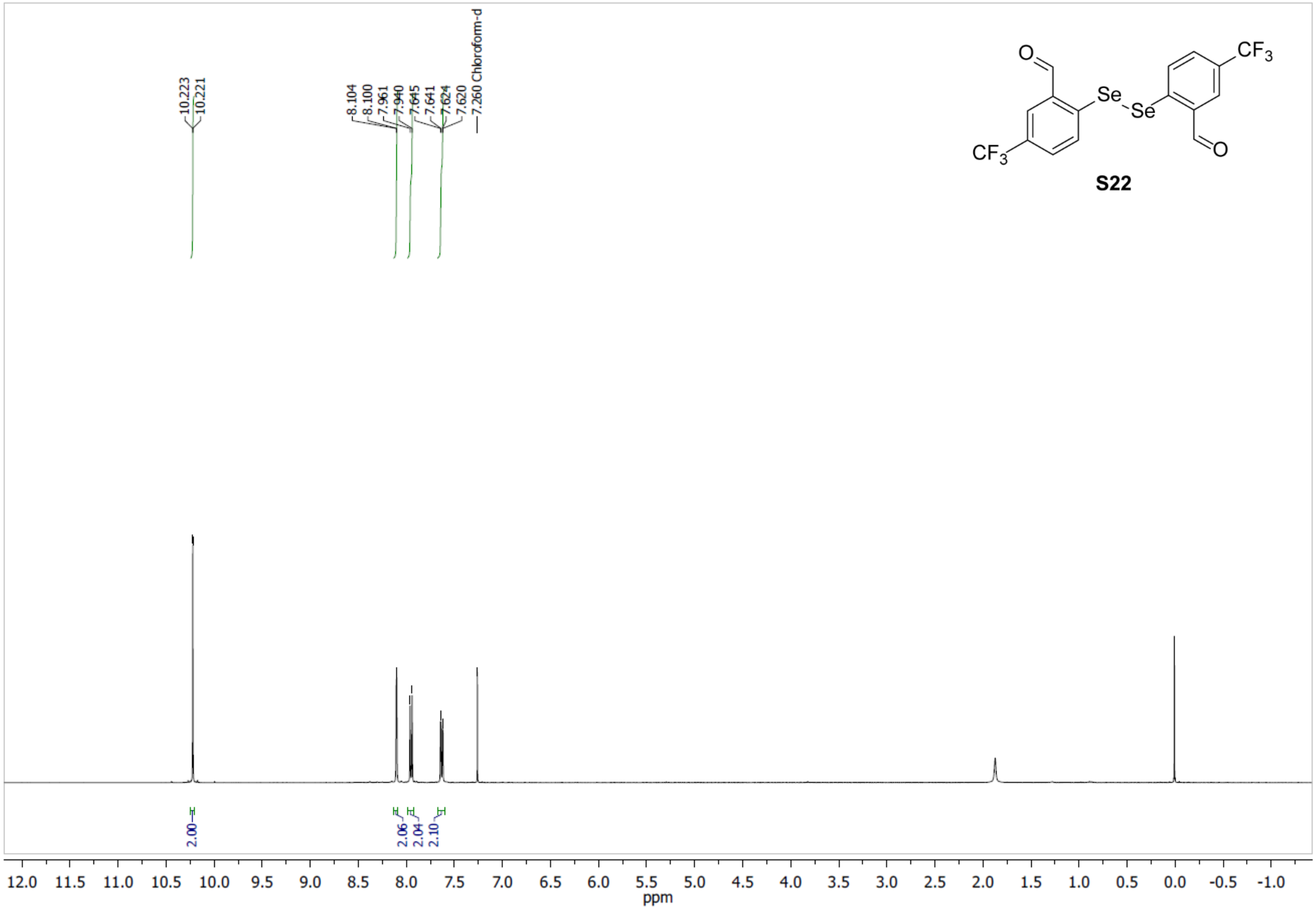

^{13}C NMR in $CDCl_3$

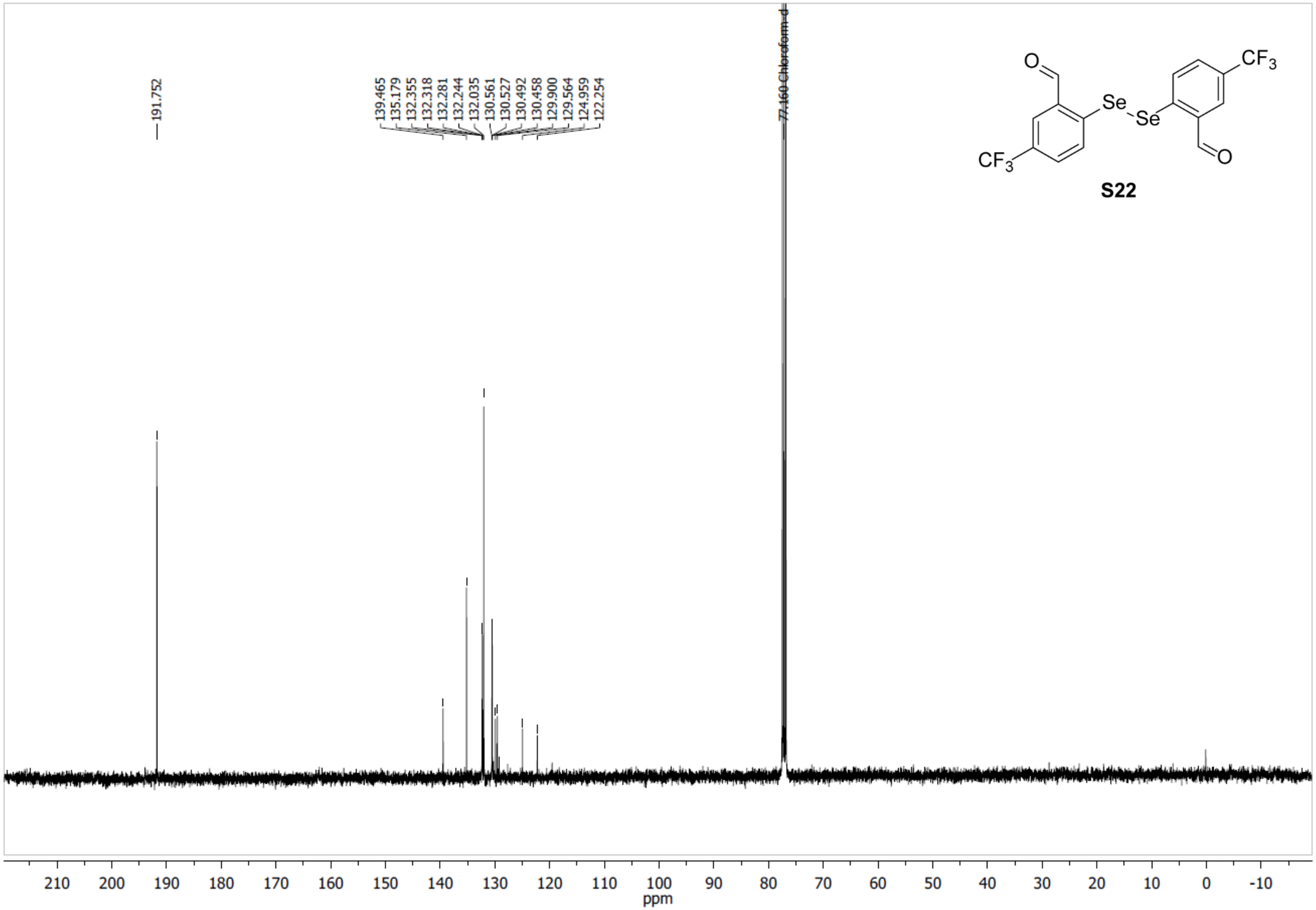

^{19}F NMR in $CDCl_3$

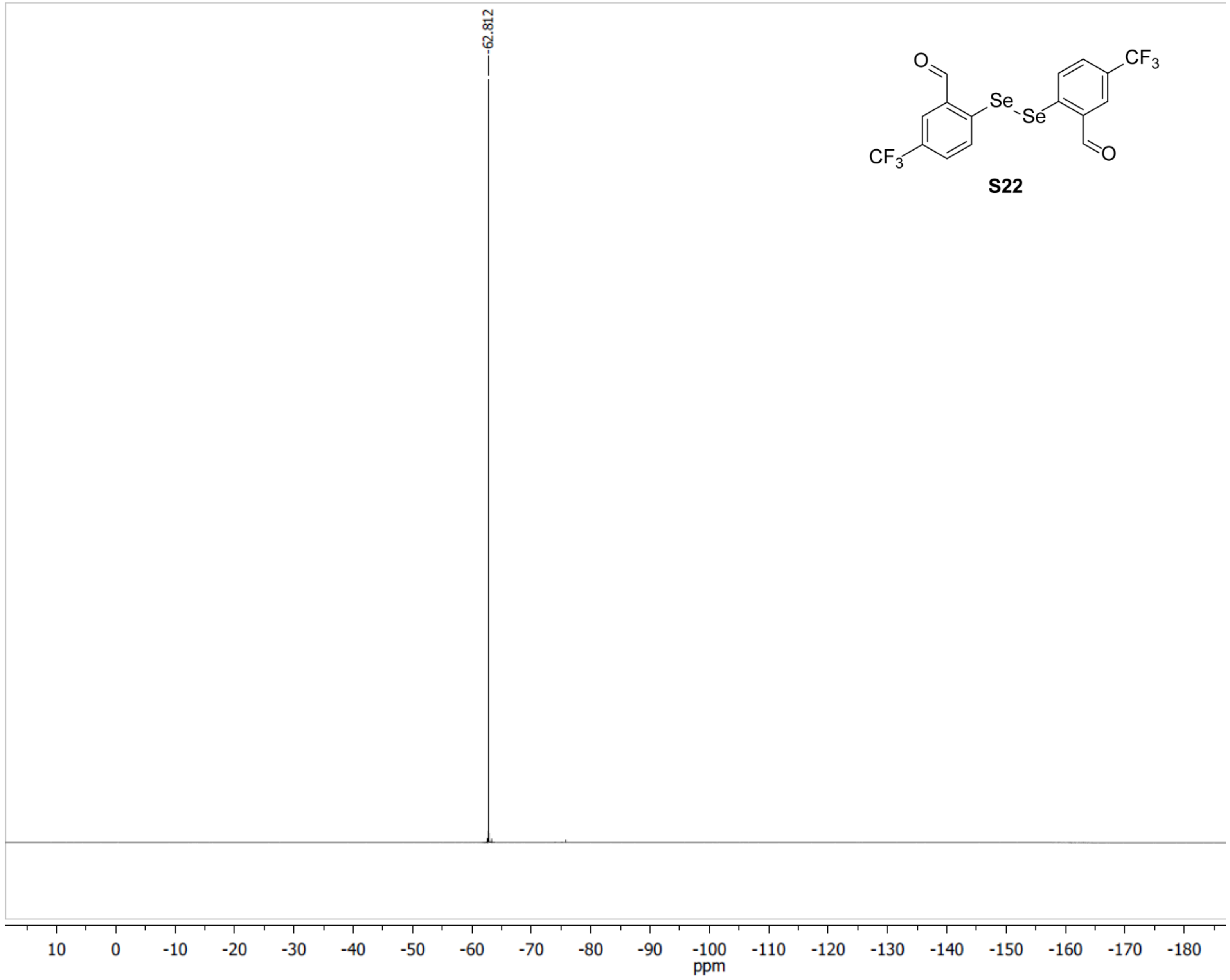

^{77}Se NMR in $CDCl_3$

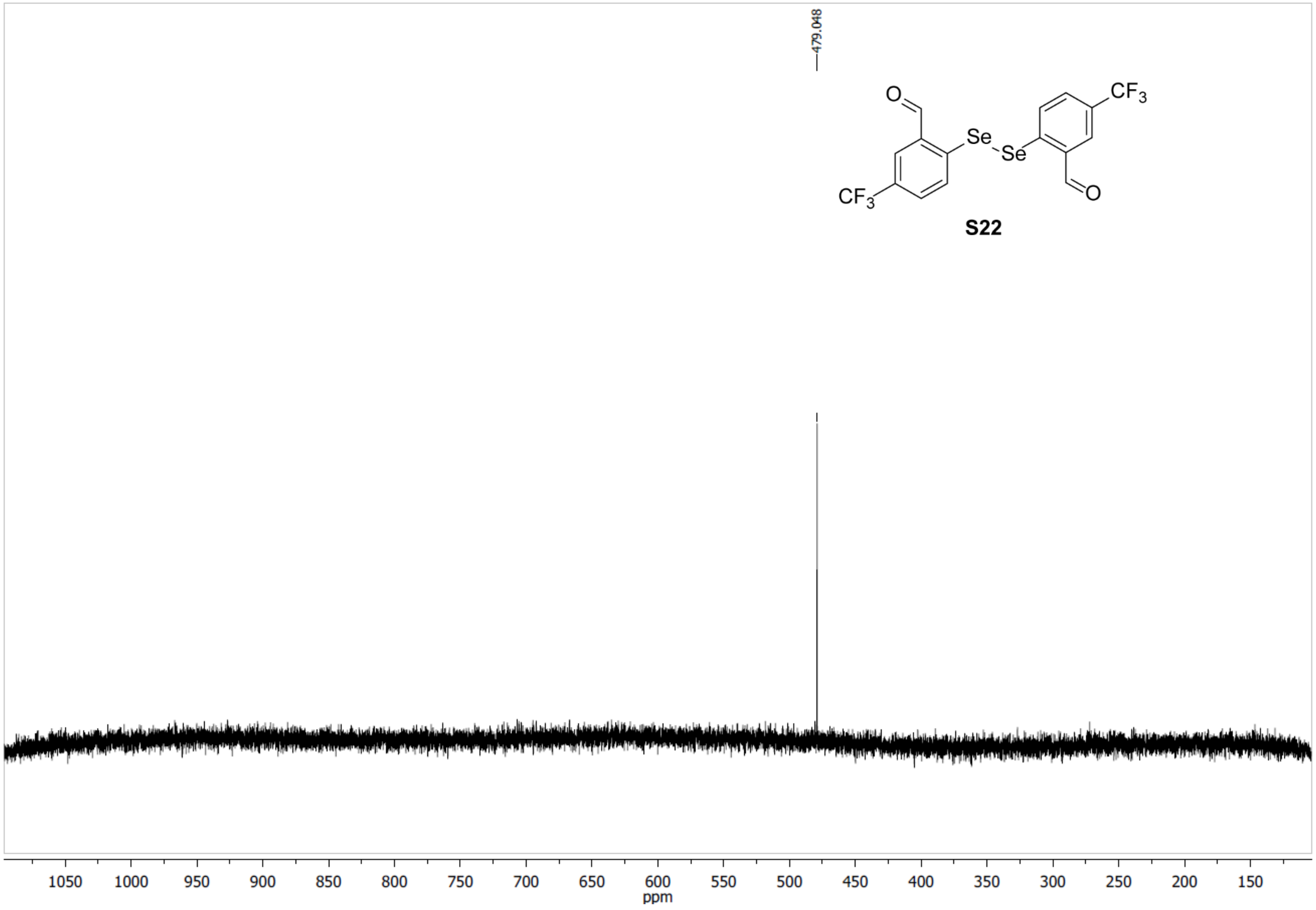

S22

^{1}H NMR in DMSO-d_6

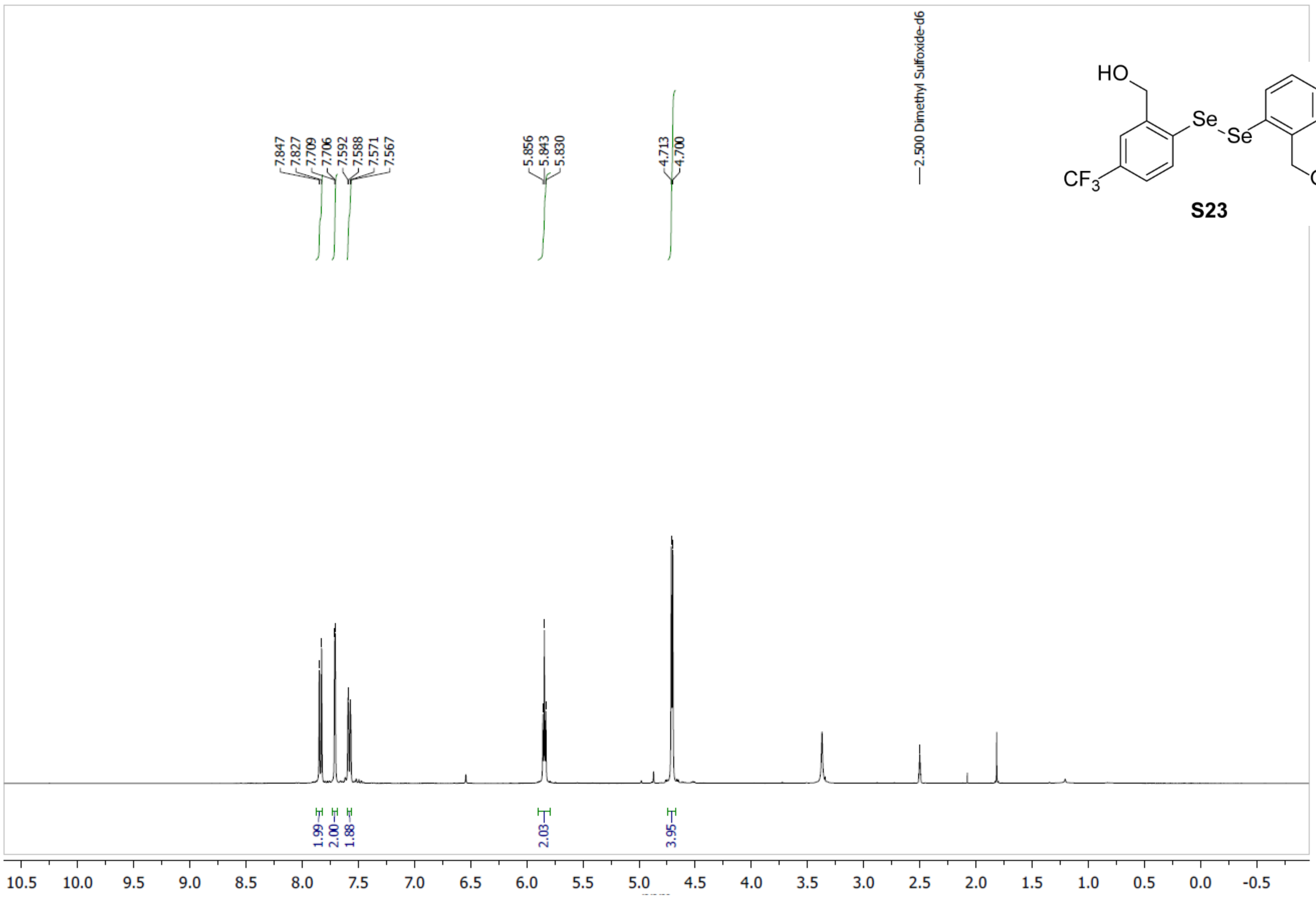

S23

^{13}C NMR in DMSO-d_6

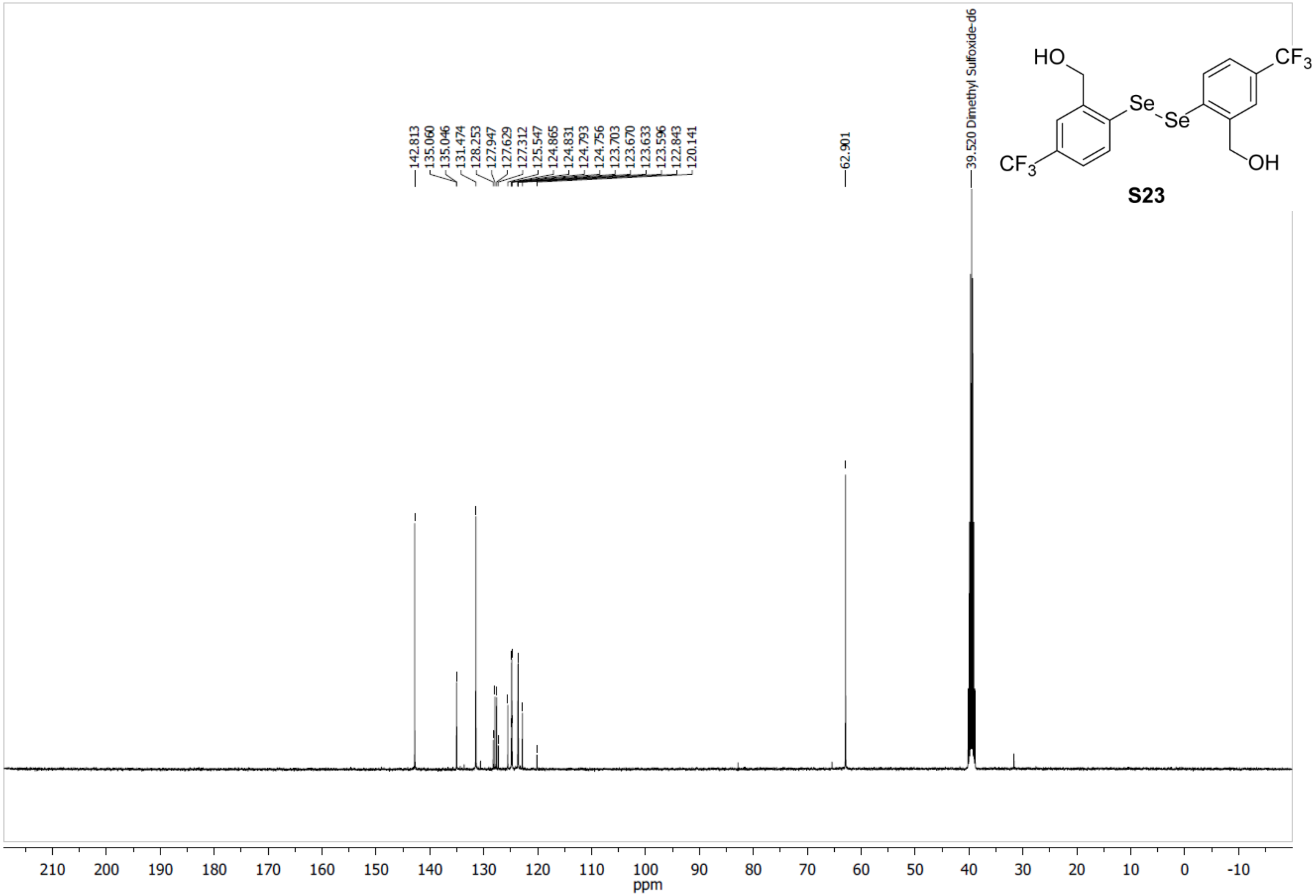

S23

^{19}F NMR in DMSO-d_6

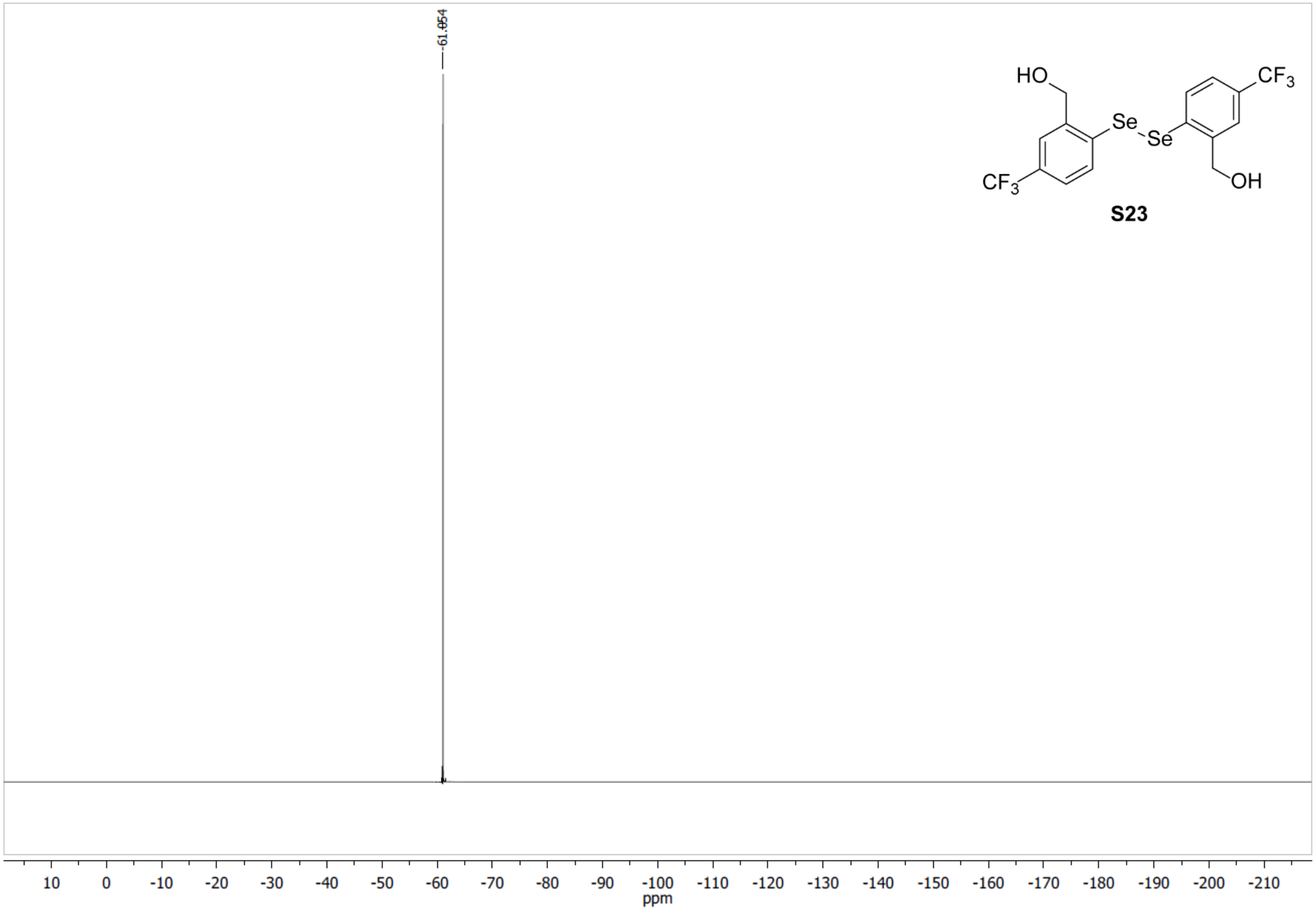

^{77}Se NMR in DMSO-d_6

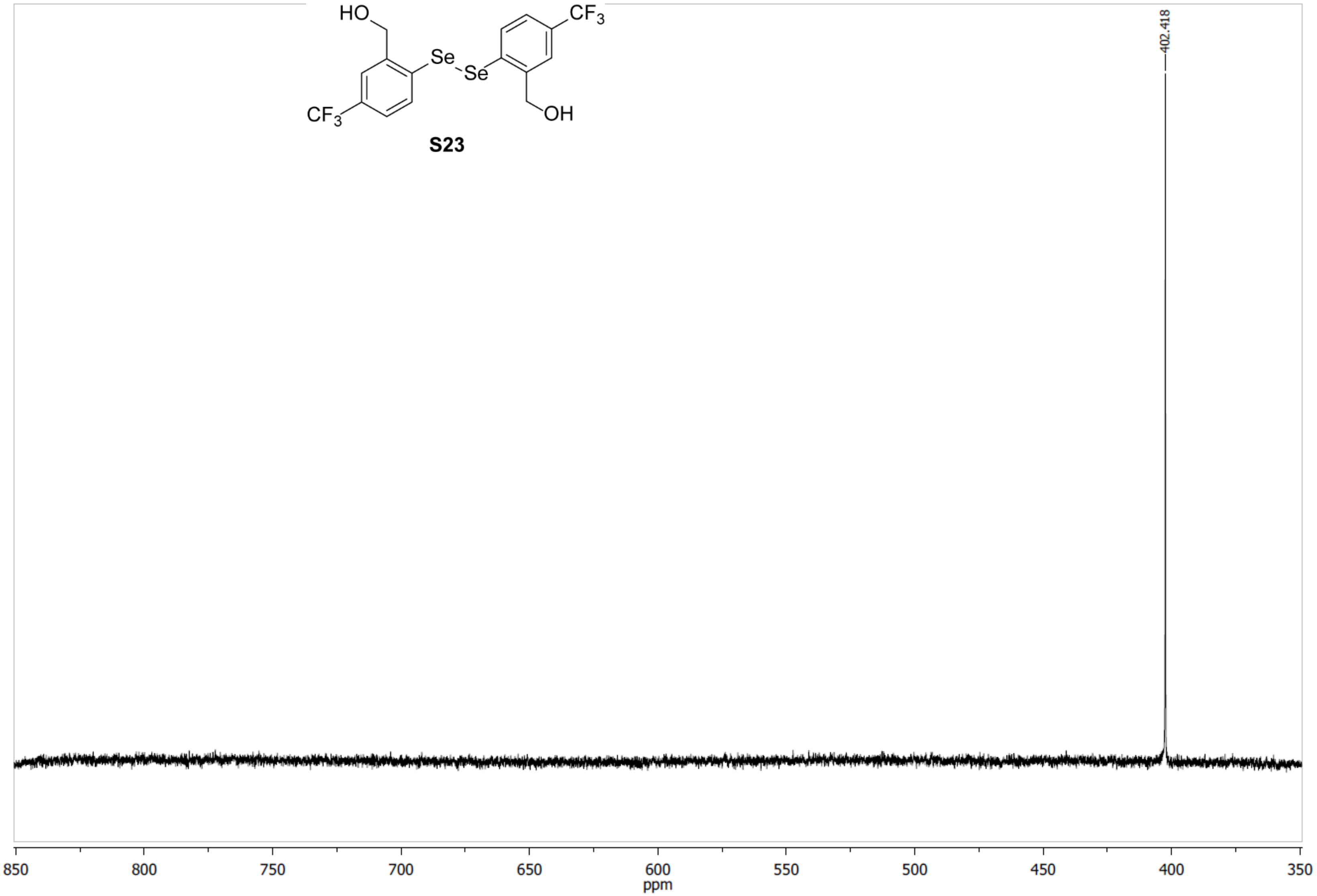

^{1}H NMR in $CDCl_3$

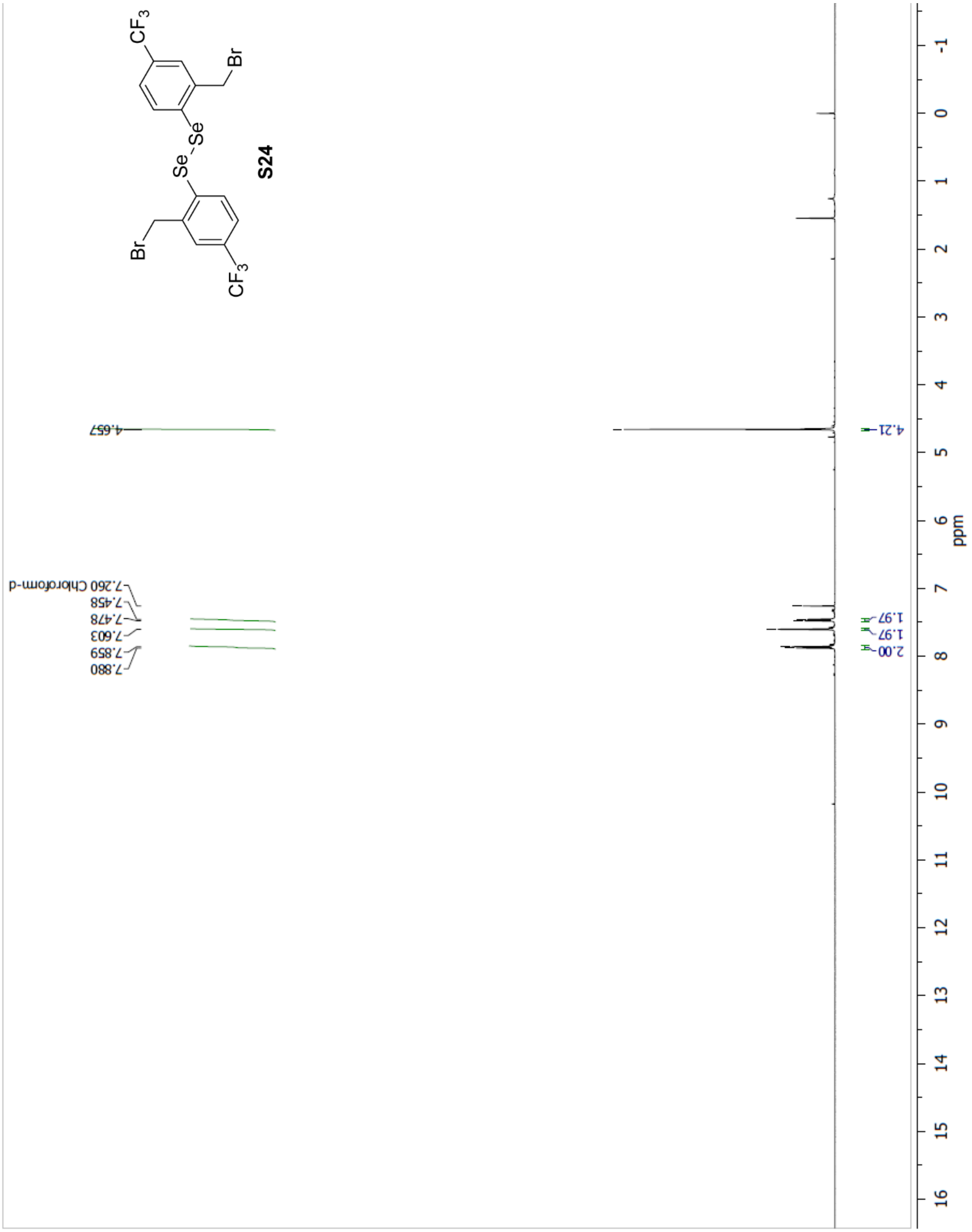

^{13}C NMR in $CDCl_3$

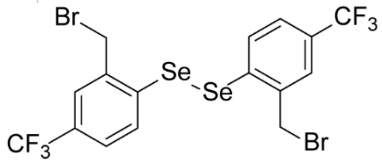

S24

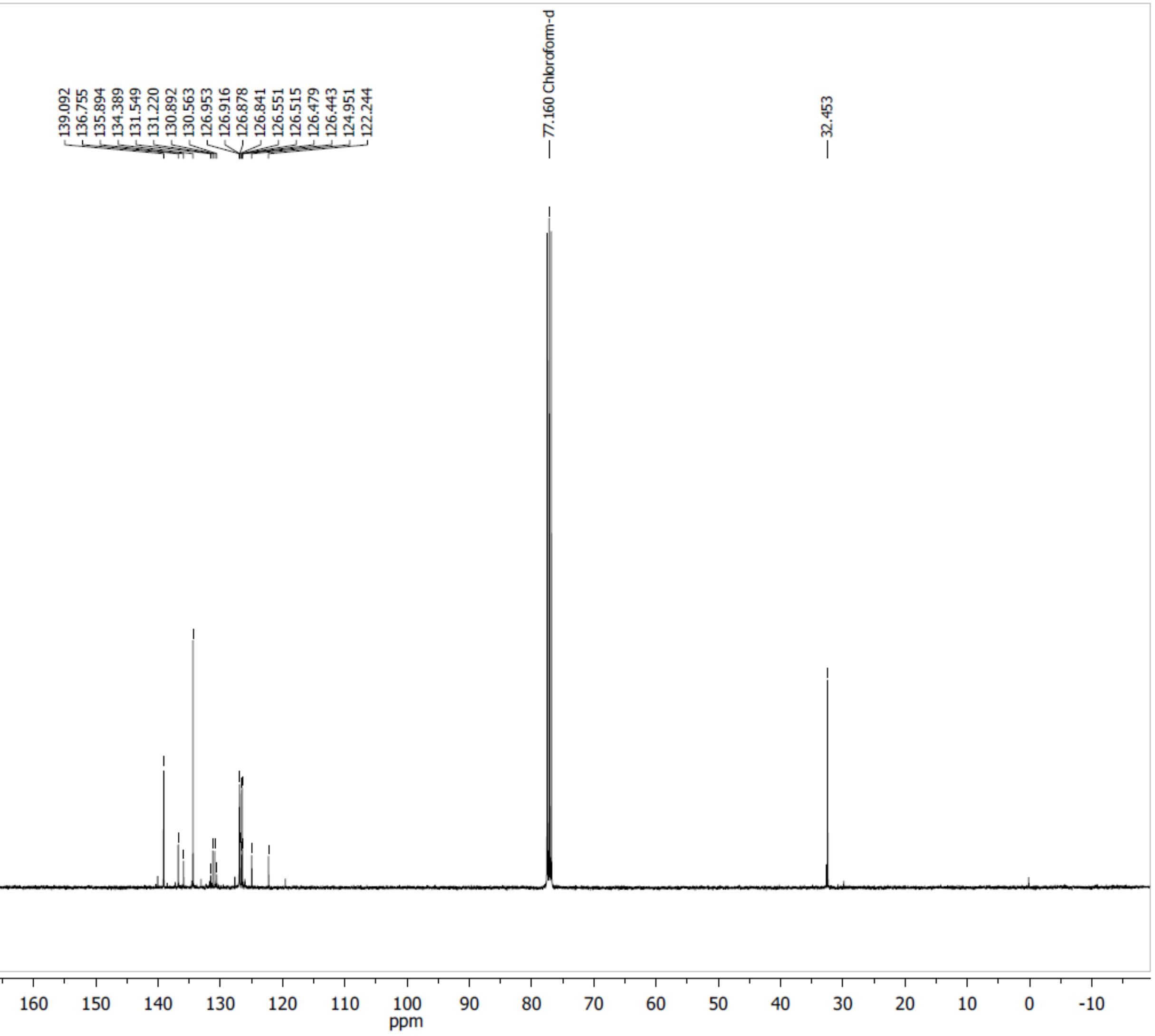

^{19}F NMR in $CDCl_3$

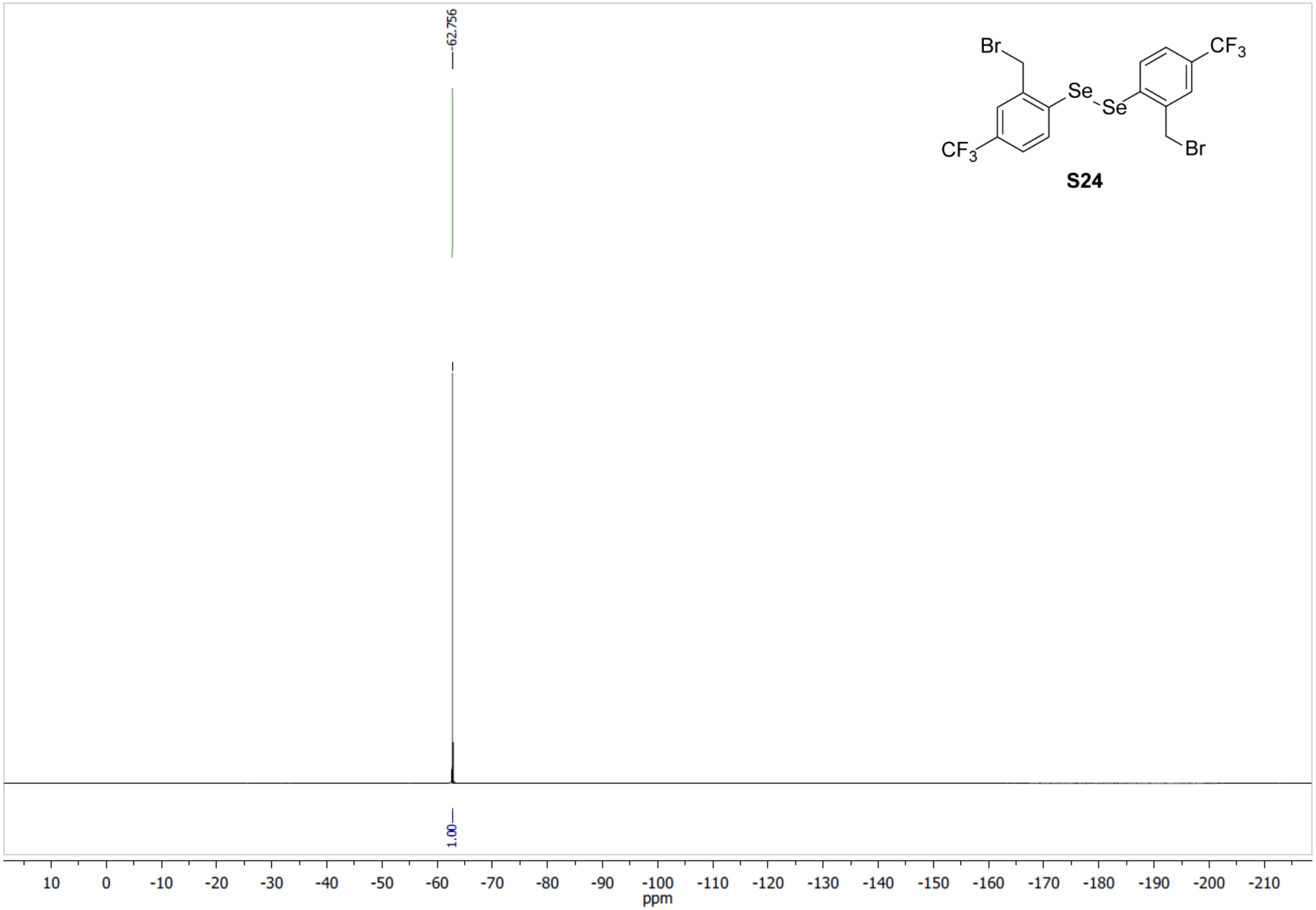

^{77}Se NMR in $CDCl_3$

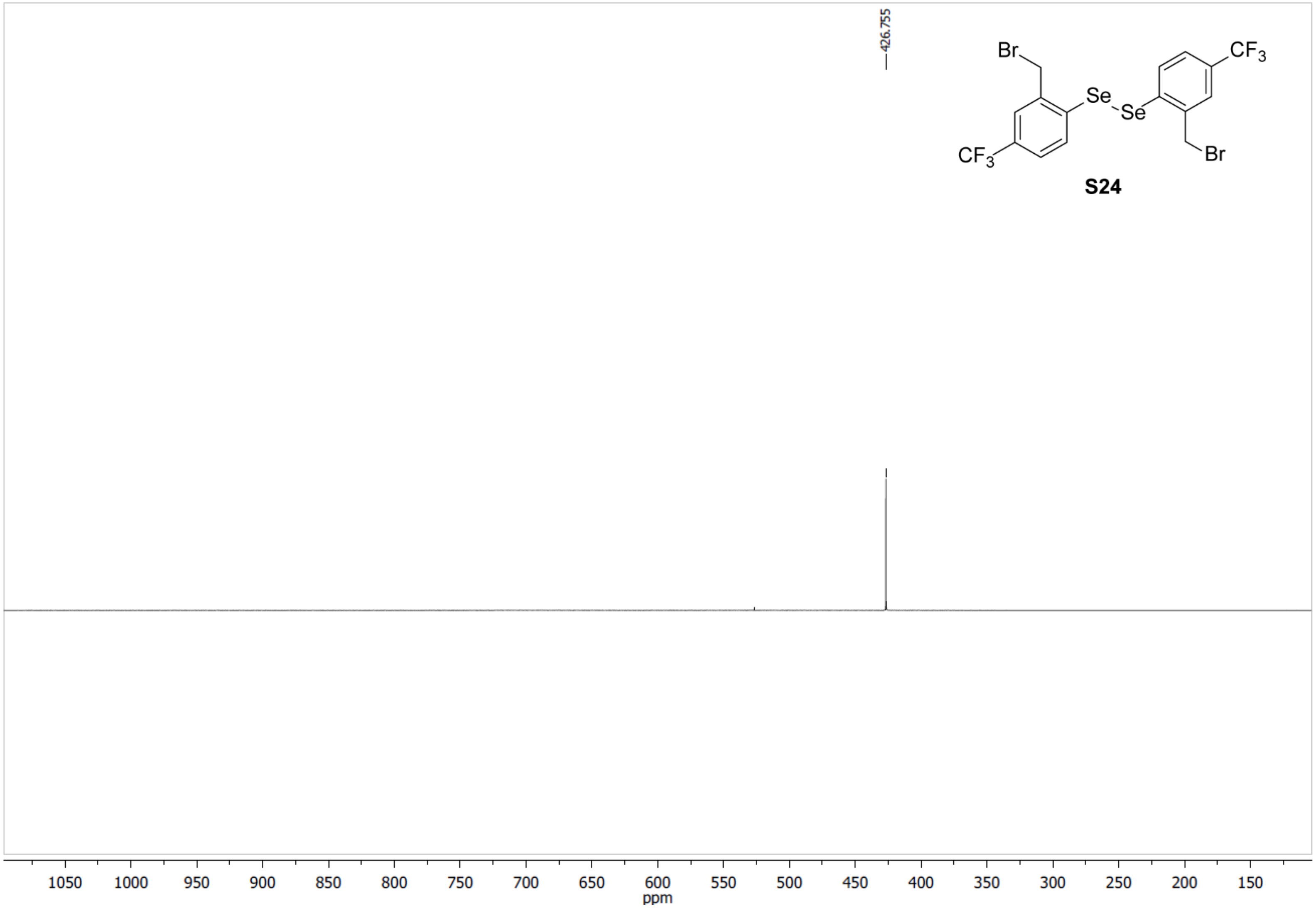

^{1}H NMR in $CDCl_3$

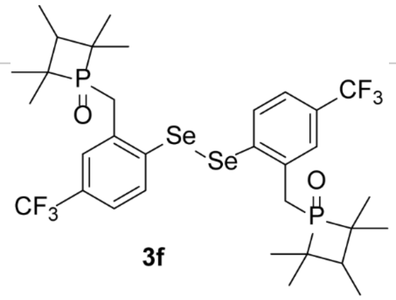

3f

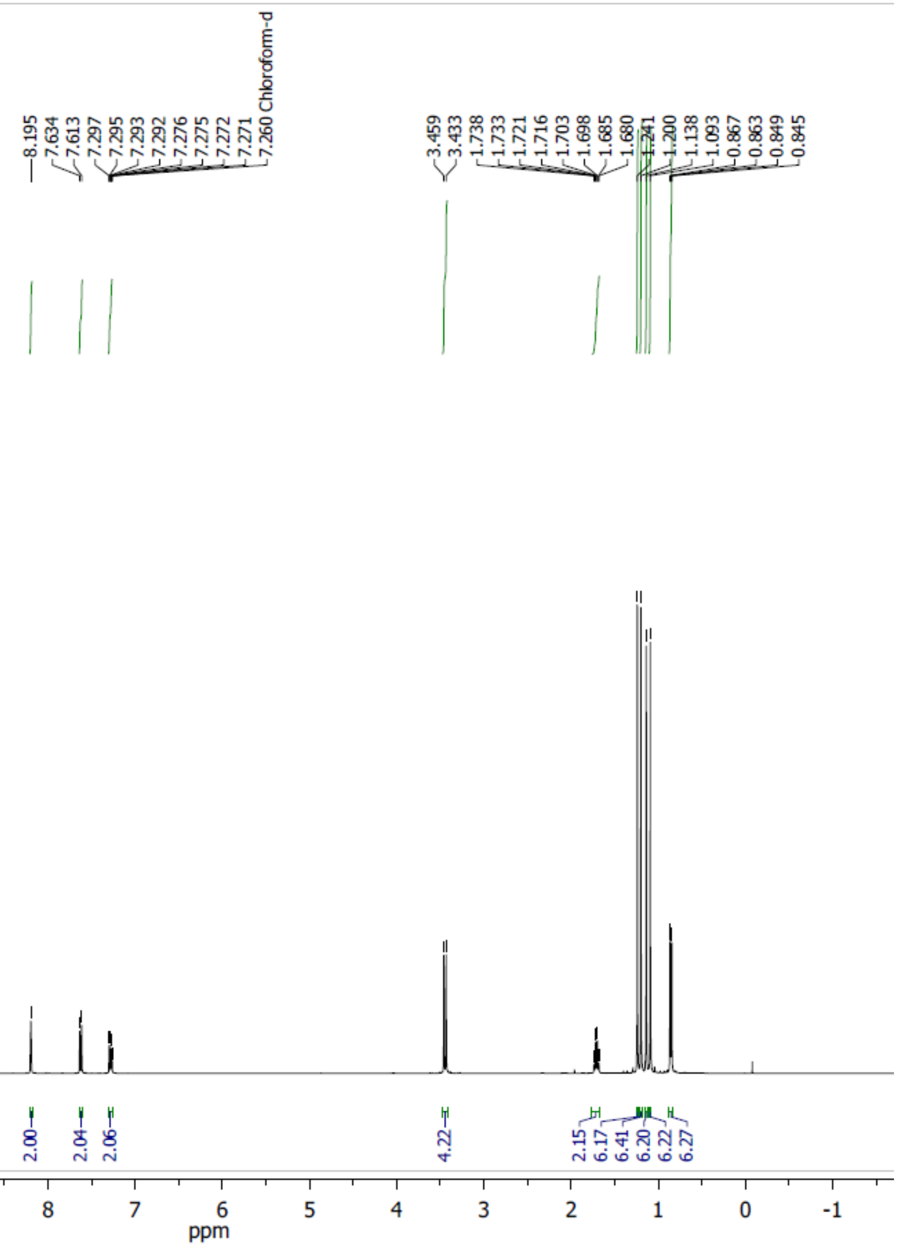

^{13}C NMR in $CDCl_3$

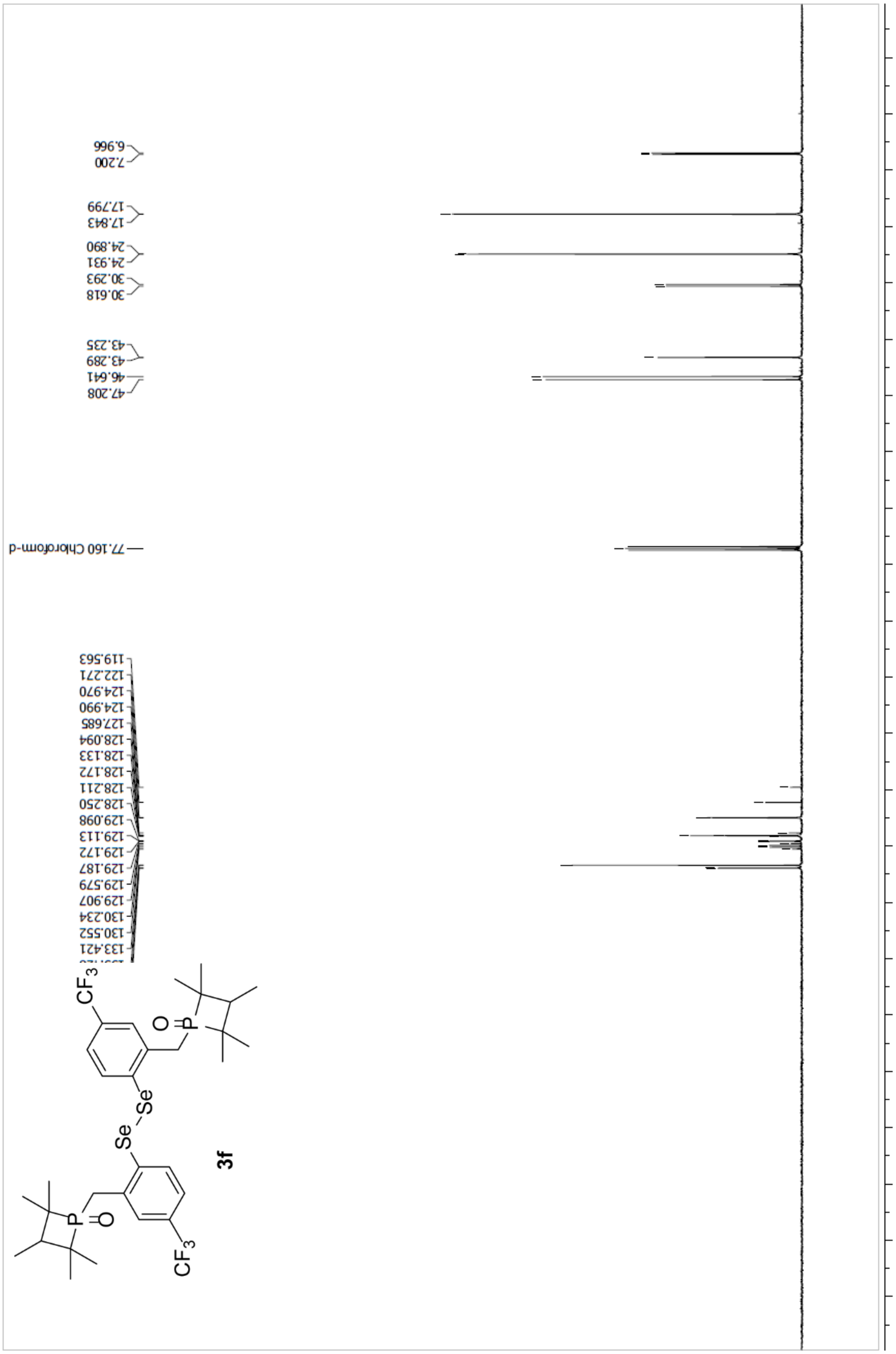

^{19}F NMR in $CDCl_3$

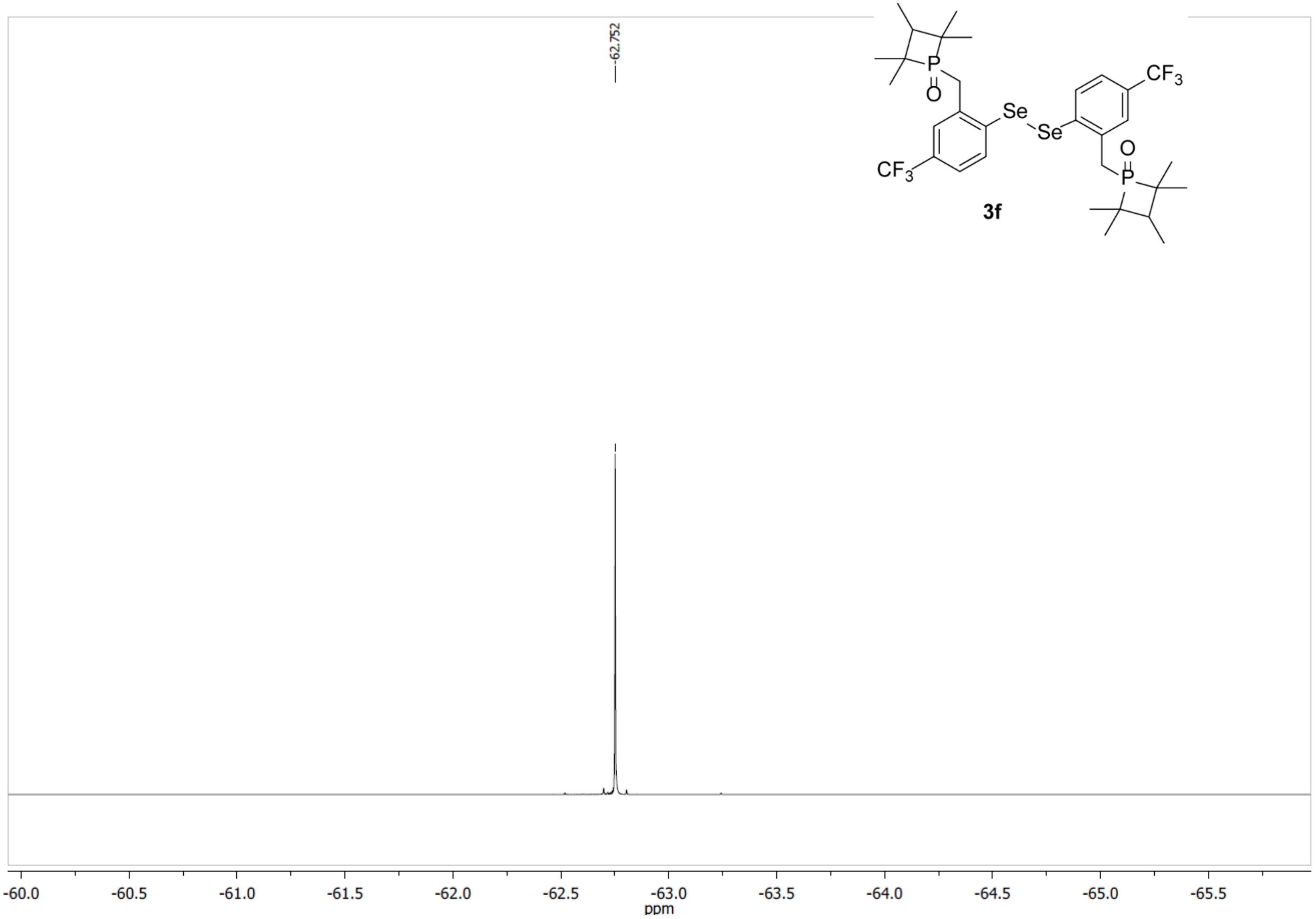

^{31}P NMR in $CDCl_3$

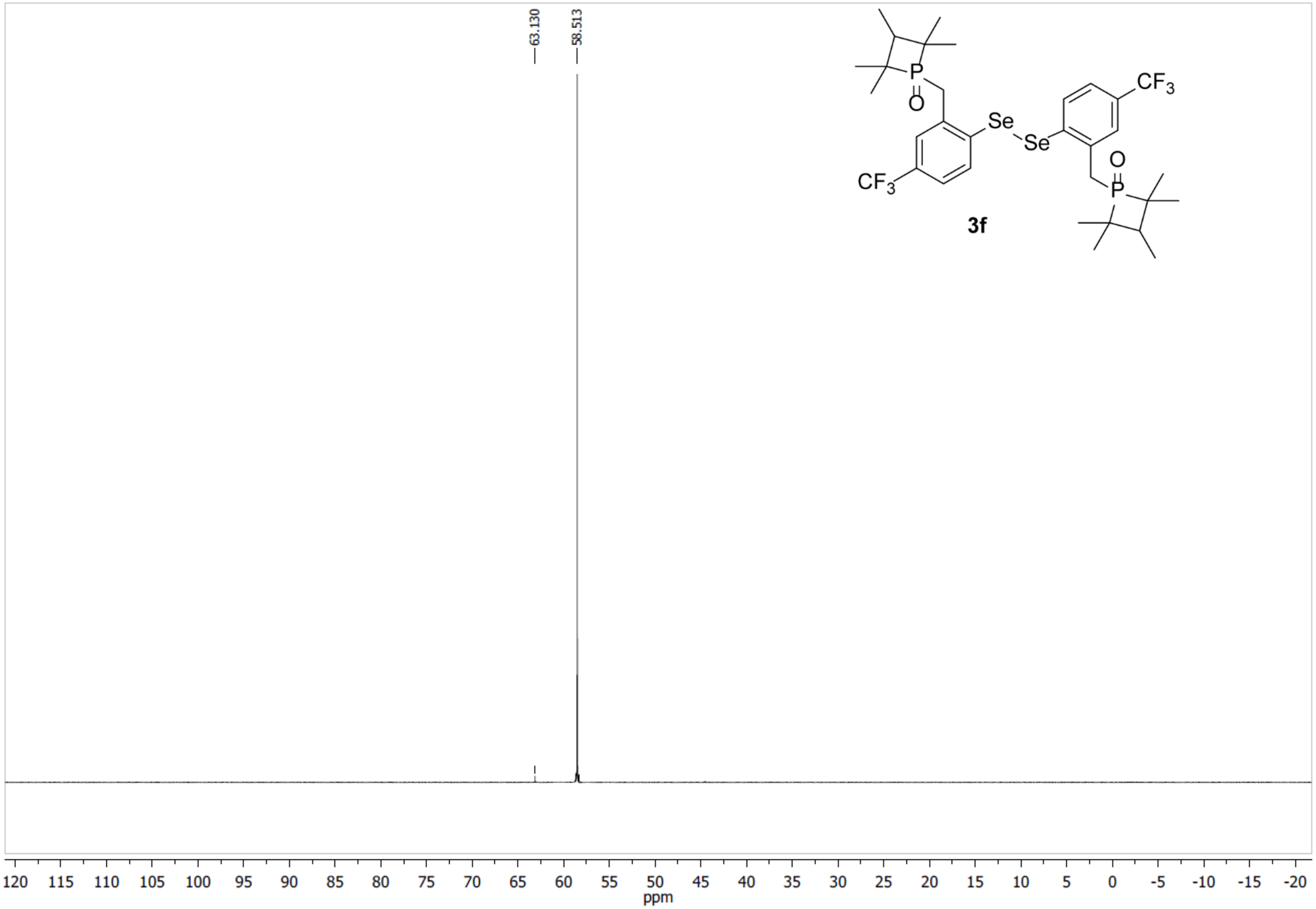

3f

^{77}Se NMR in $CDCl_3$

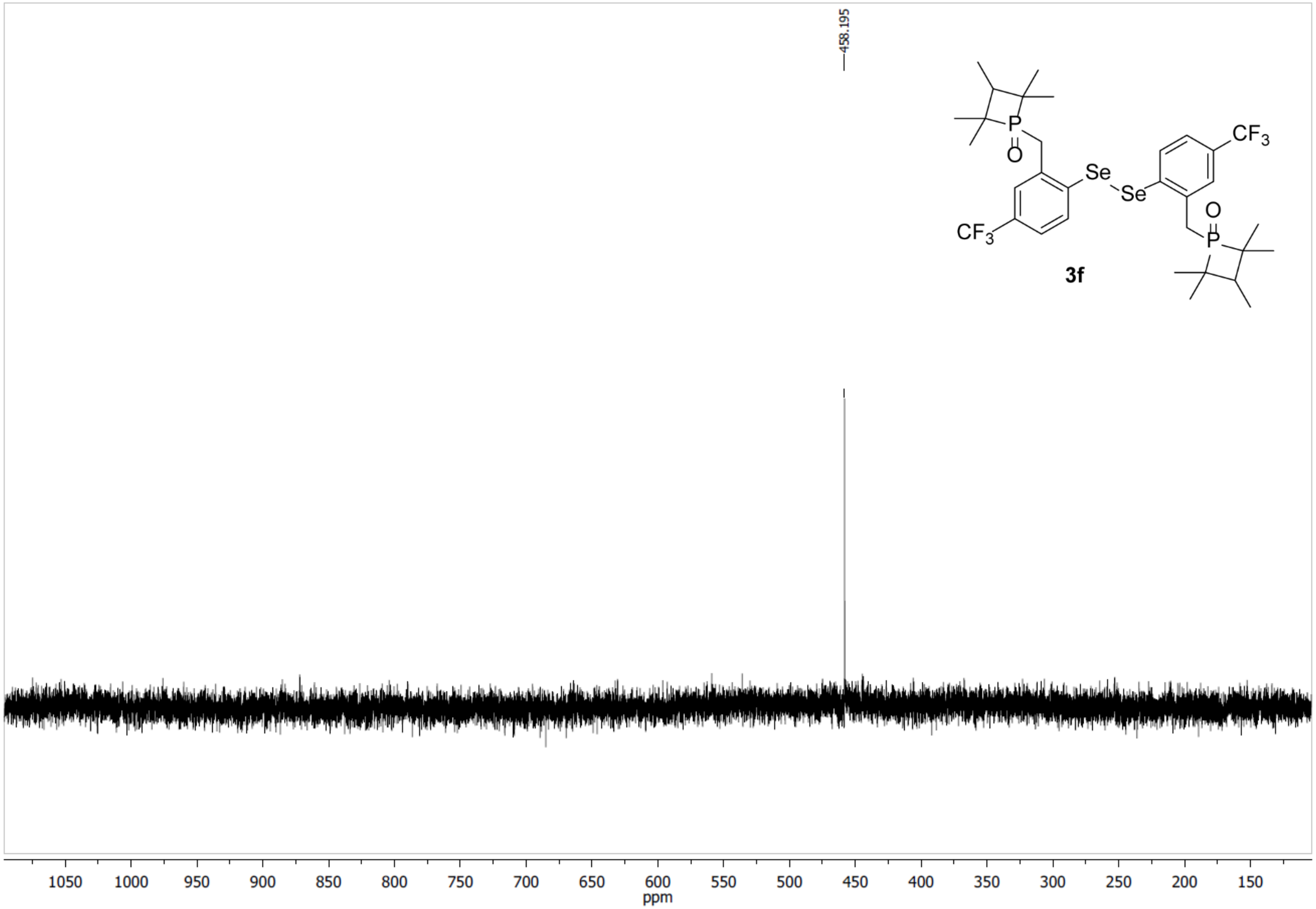

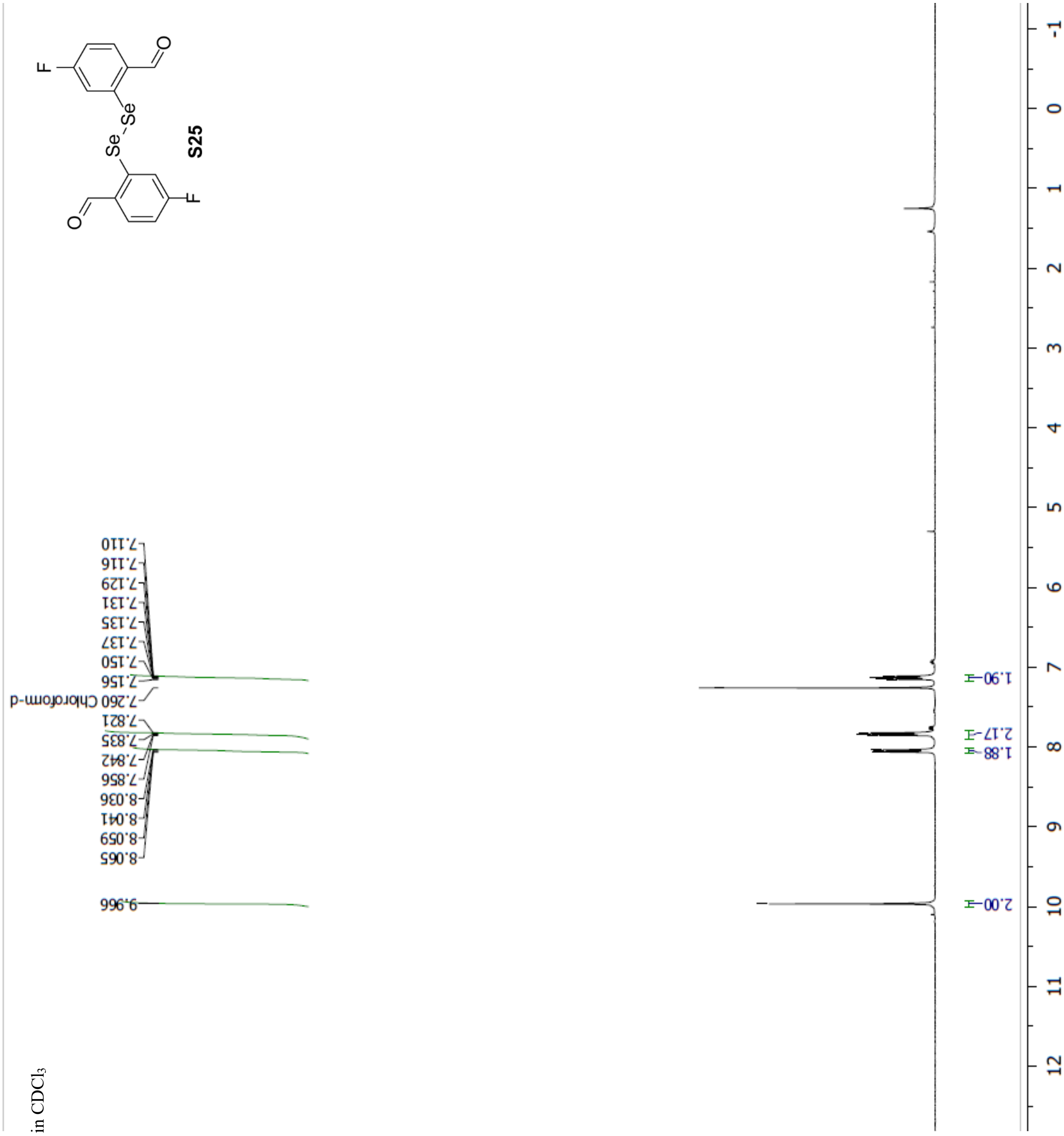

^1H NMR in $CDCl_3$

^{13}C NMR in $CDCl_3$

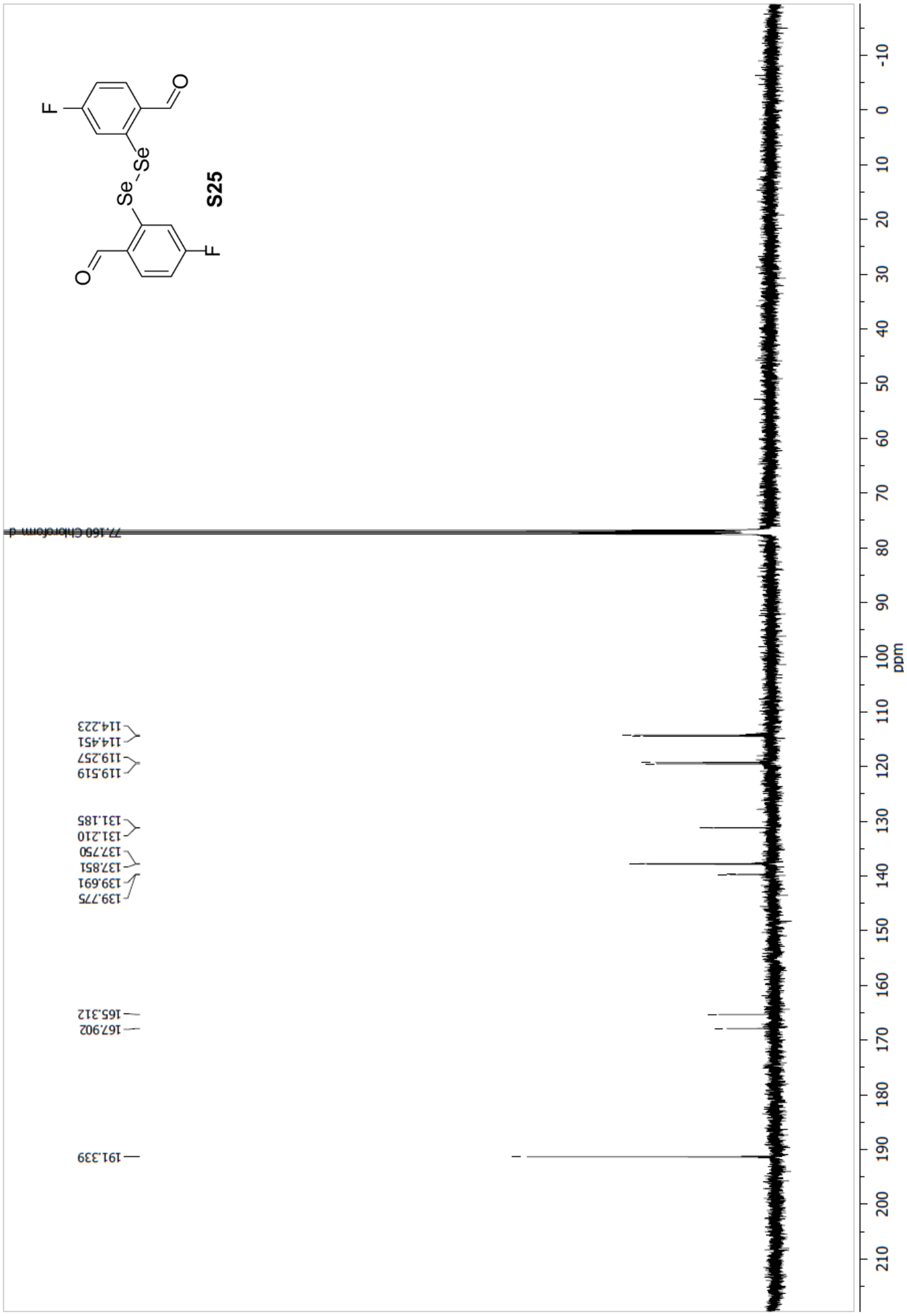

^{19}F NMR in $CDCl_3$

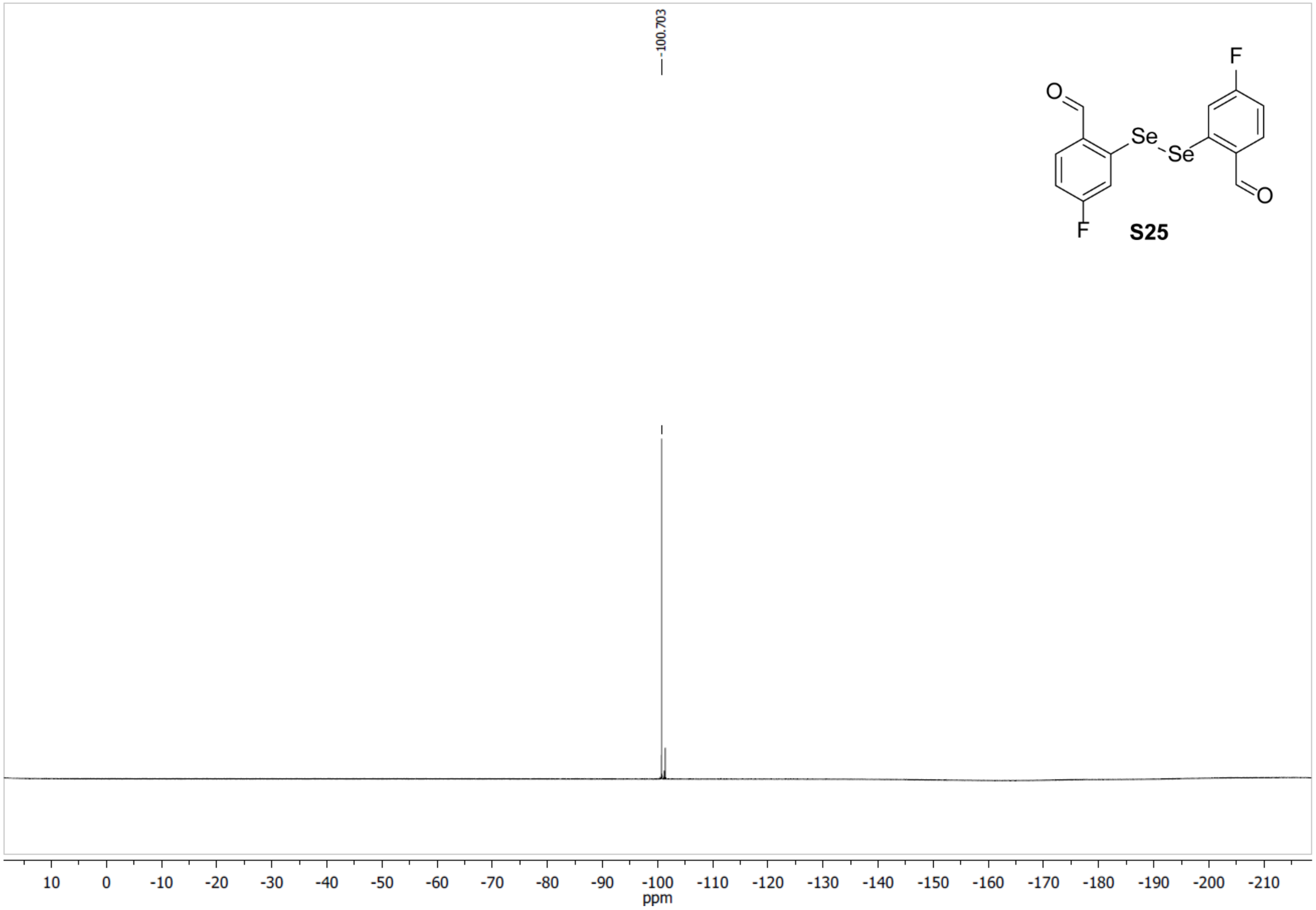

^{77}Se NMR in $CDCl_3$

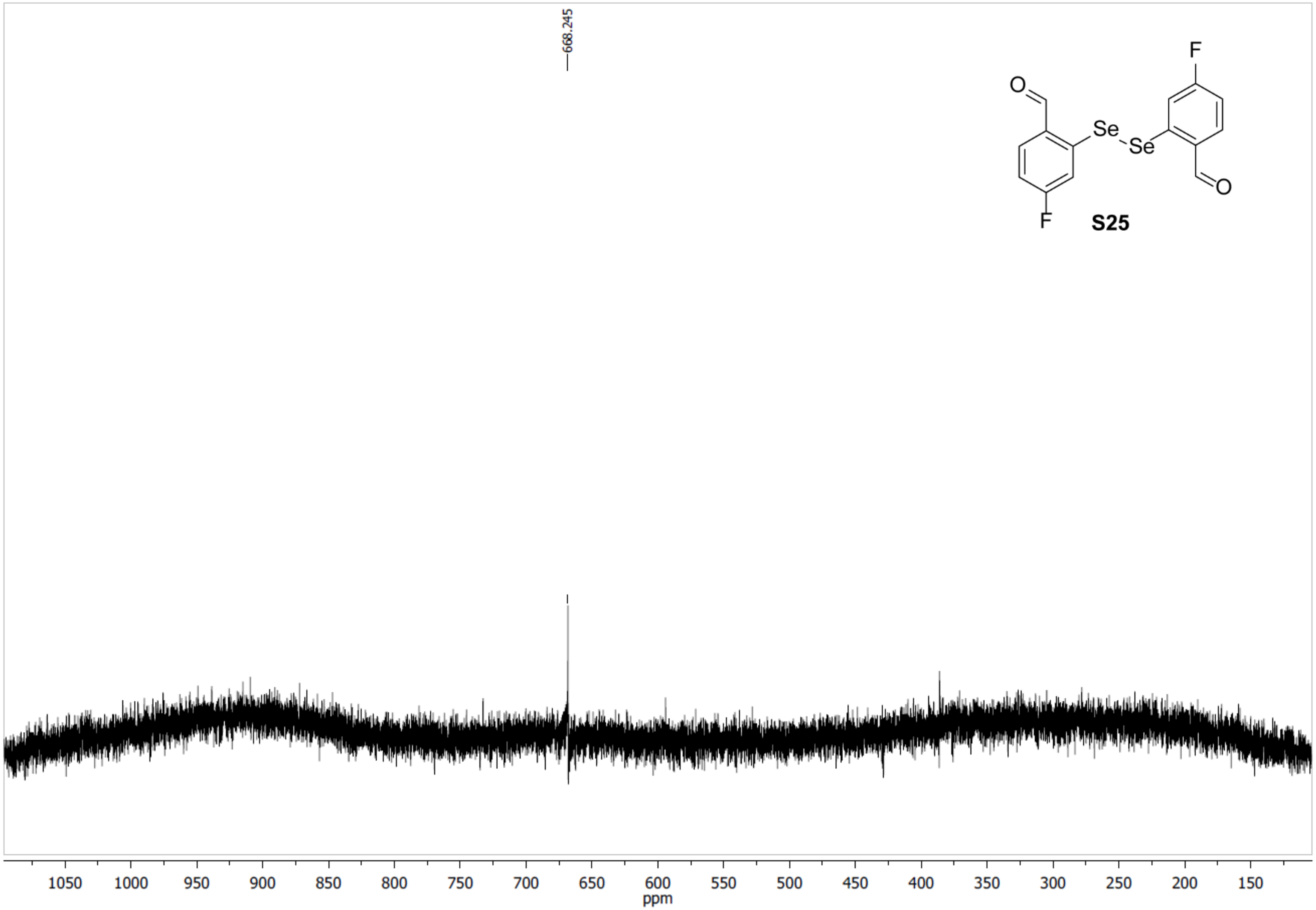

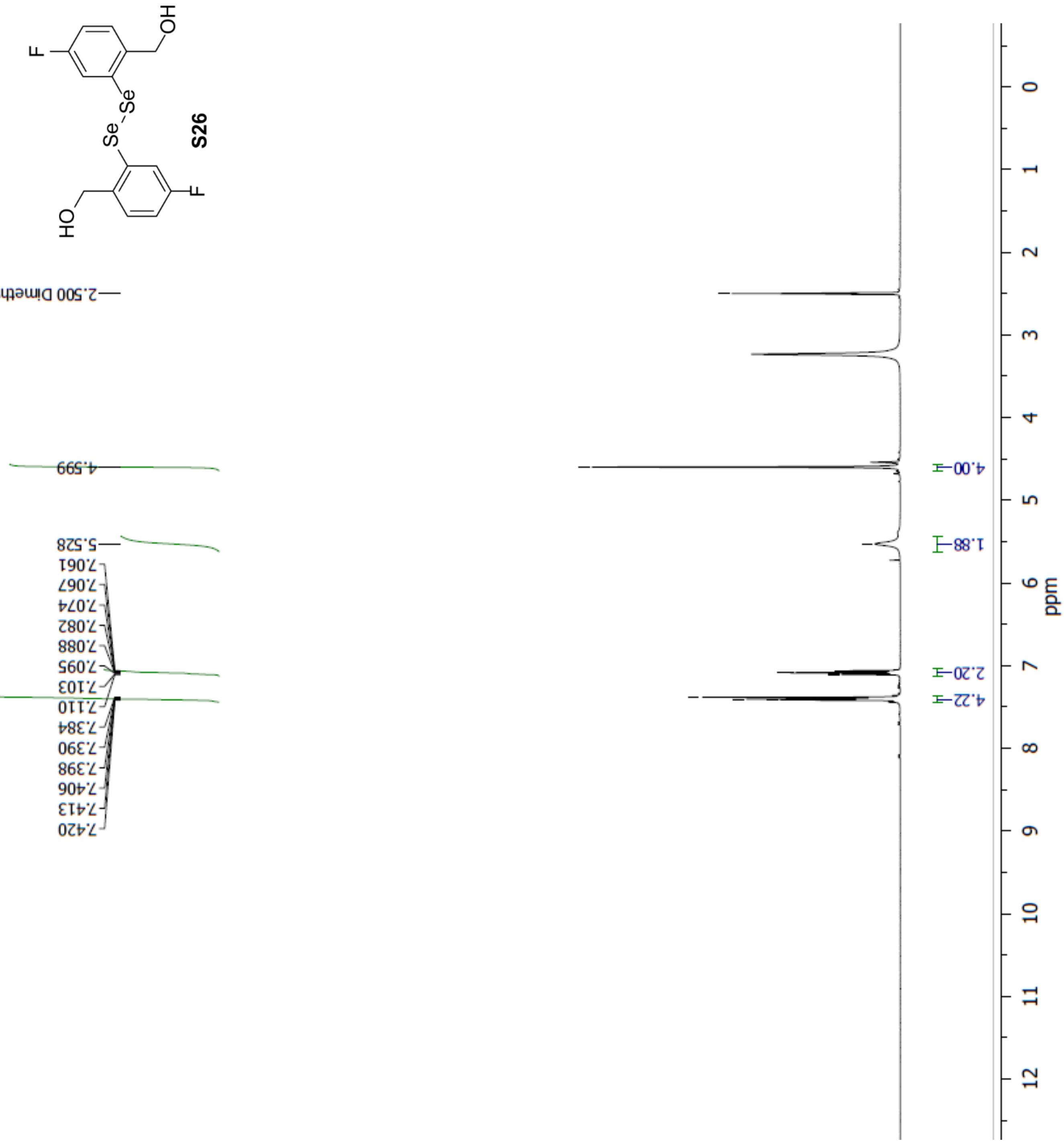

^1H NMR in DMSO-d_6

^{13}C NMR in DMSO-d_6

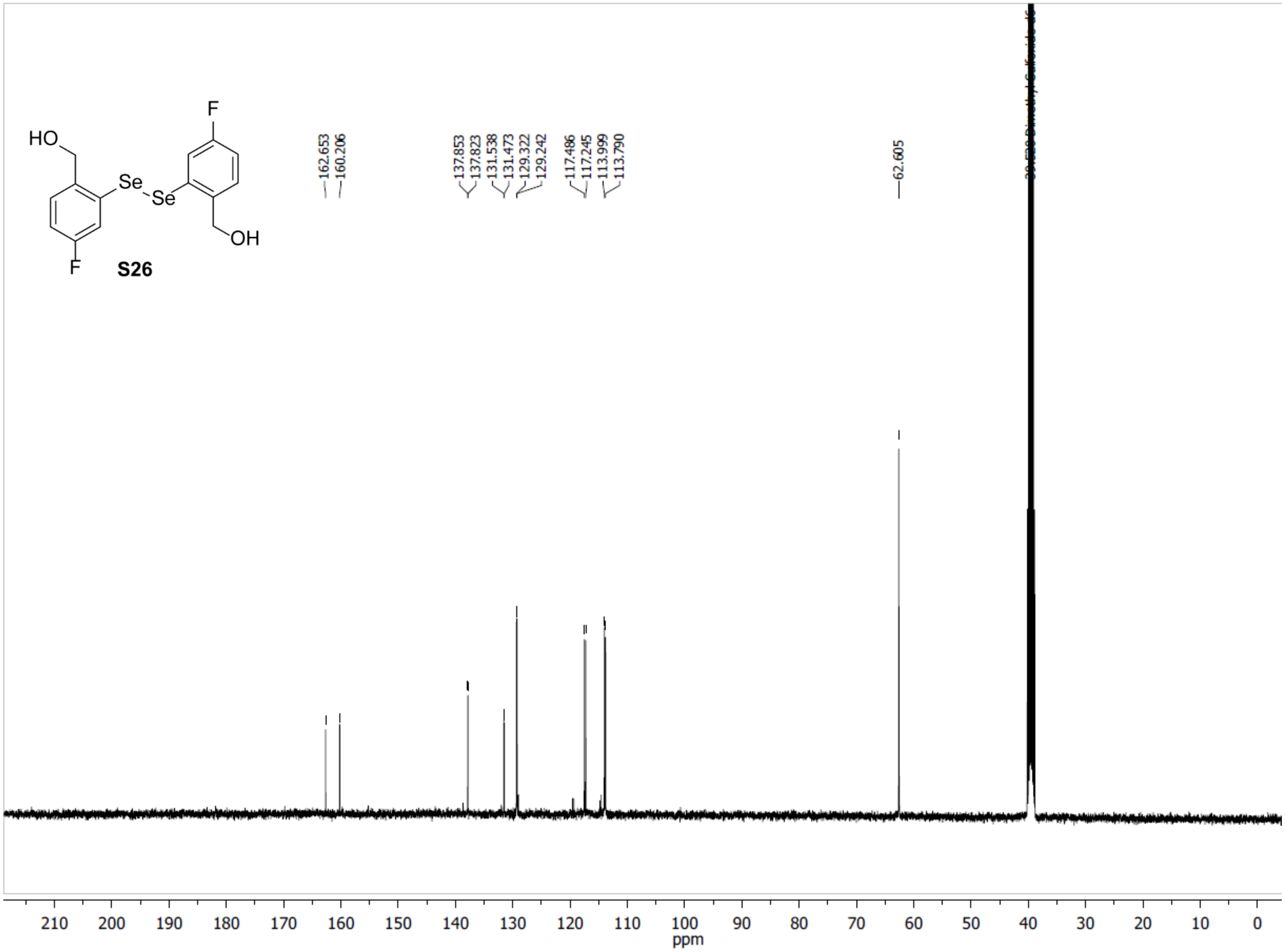

^{19}F NMR in DMSO-d$_6$

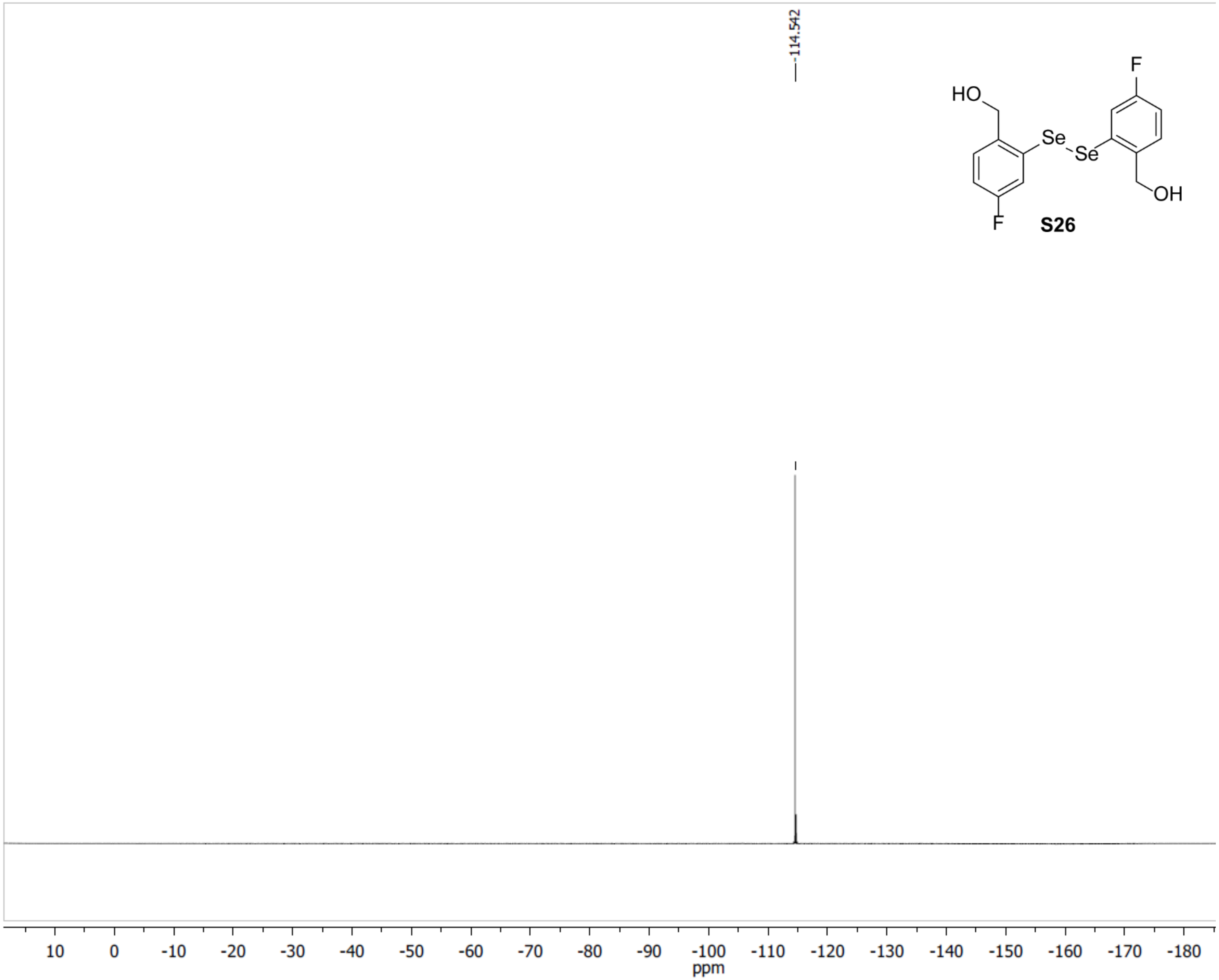

^{77}Se NMR in DMSO-d_6

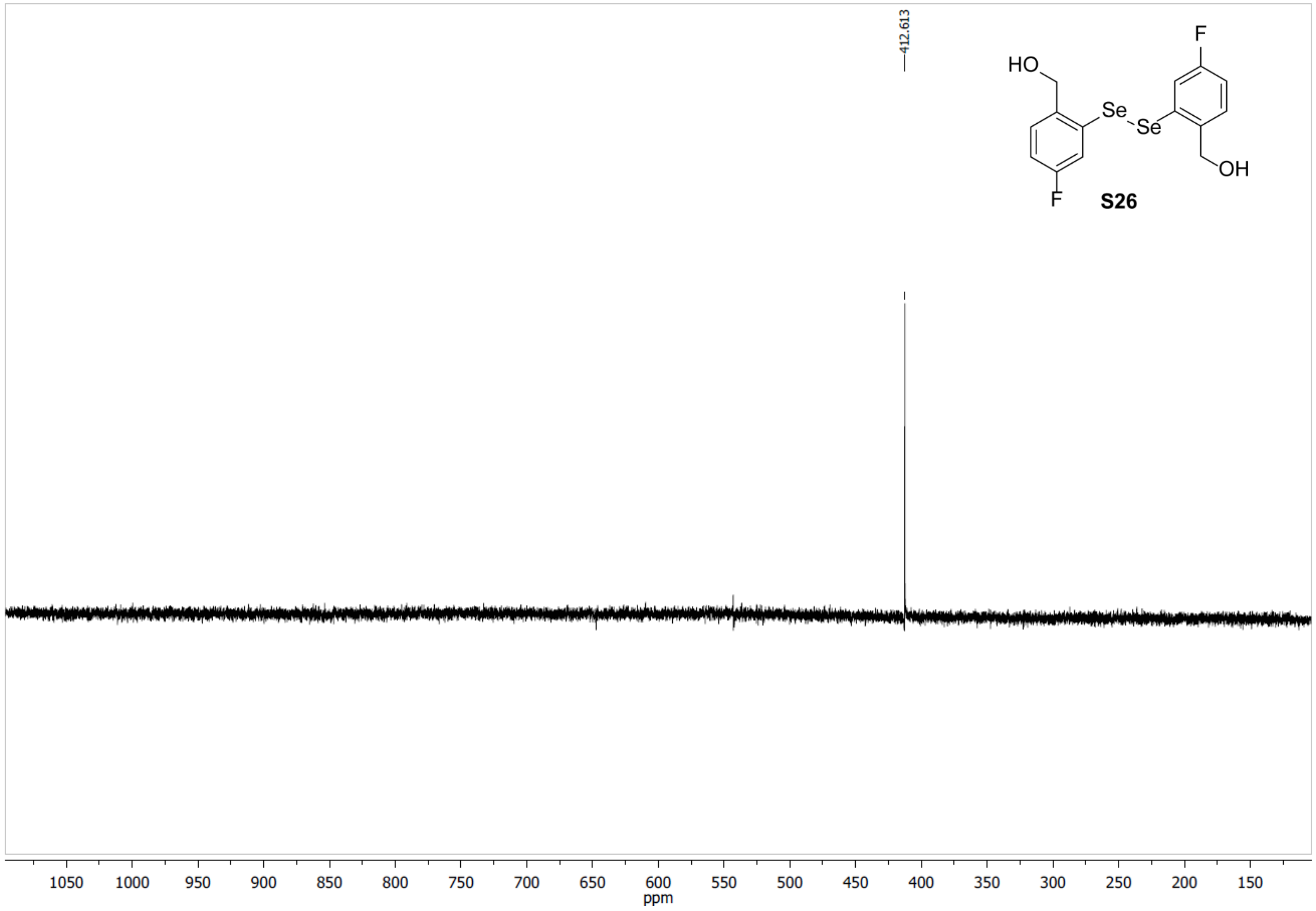

^1H NMR in $CDCl_3$

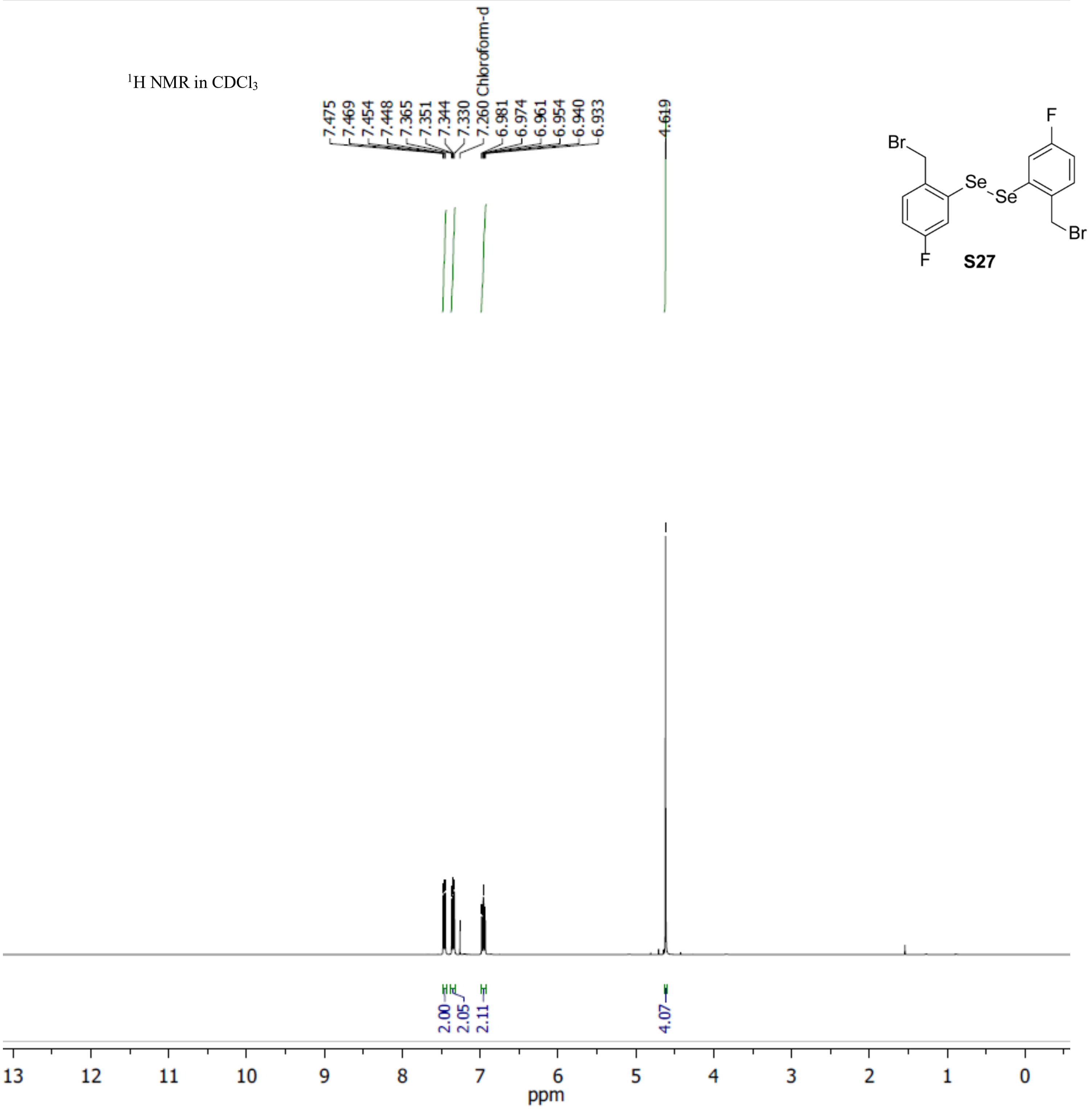

^{13}C NMR in $CDCl_3$

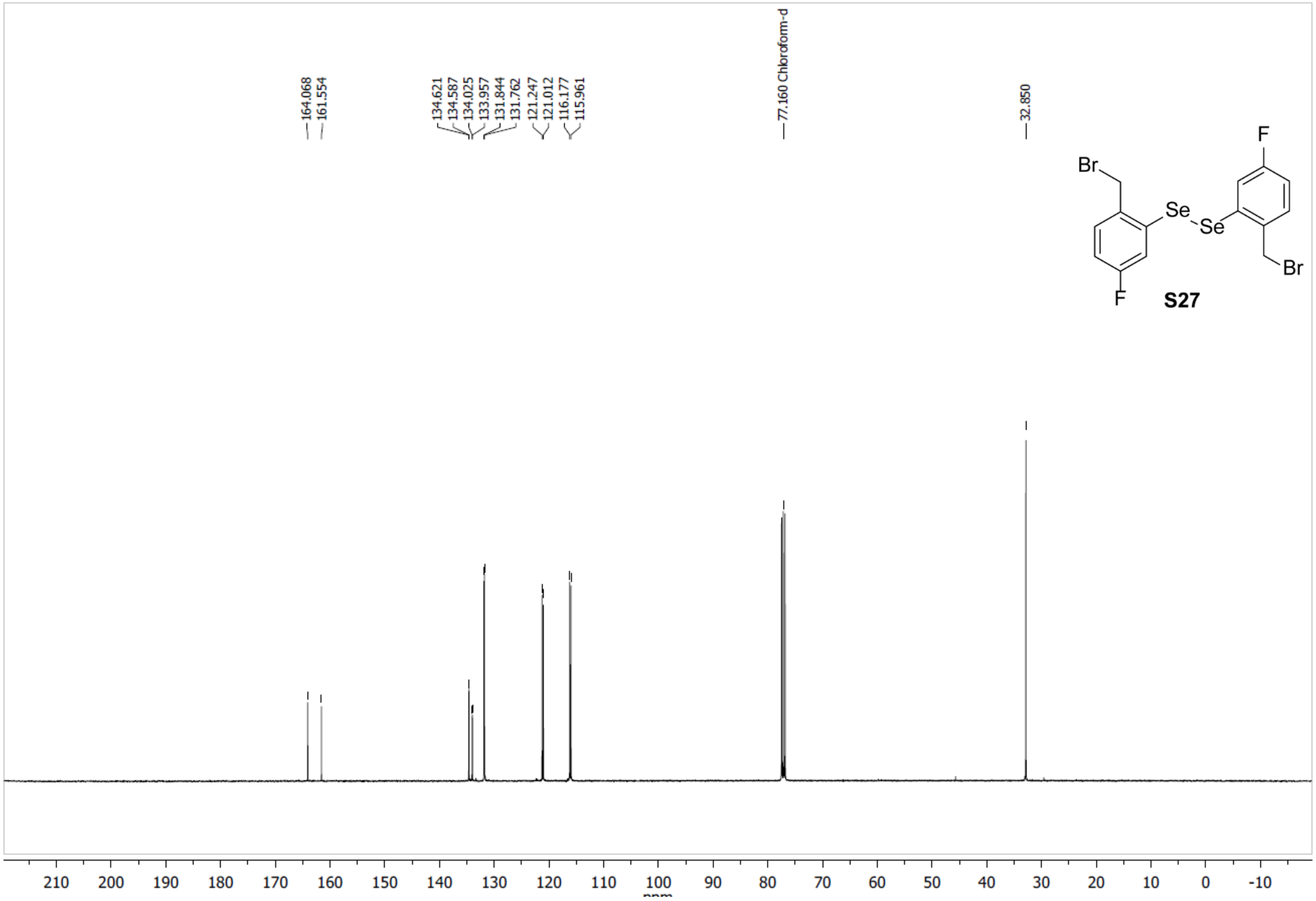

^{19}F NMR in $CDCl_3$

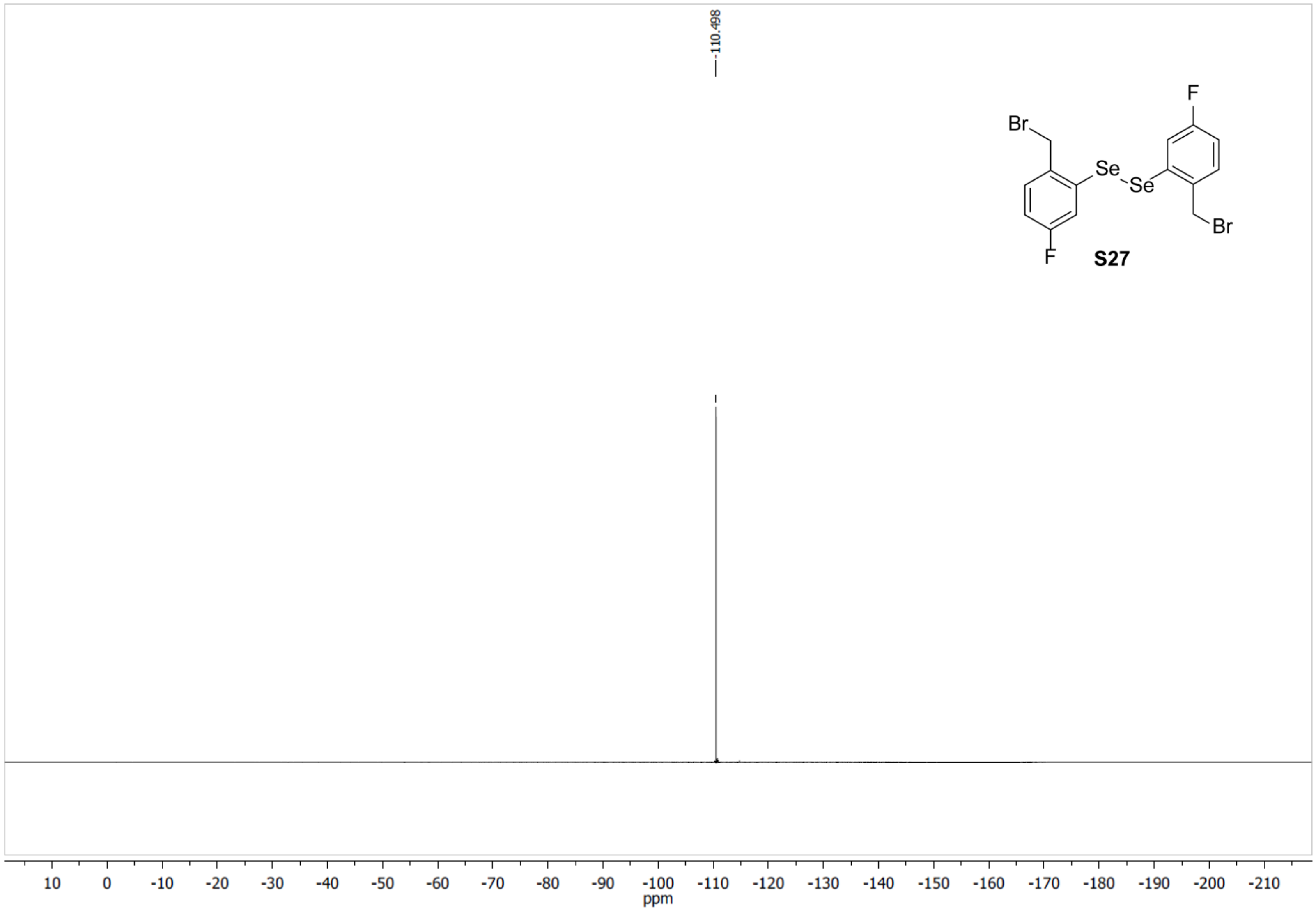

^{77}Se NMR in $CDCl_3$

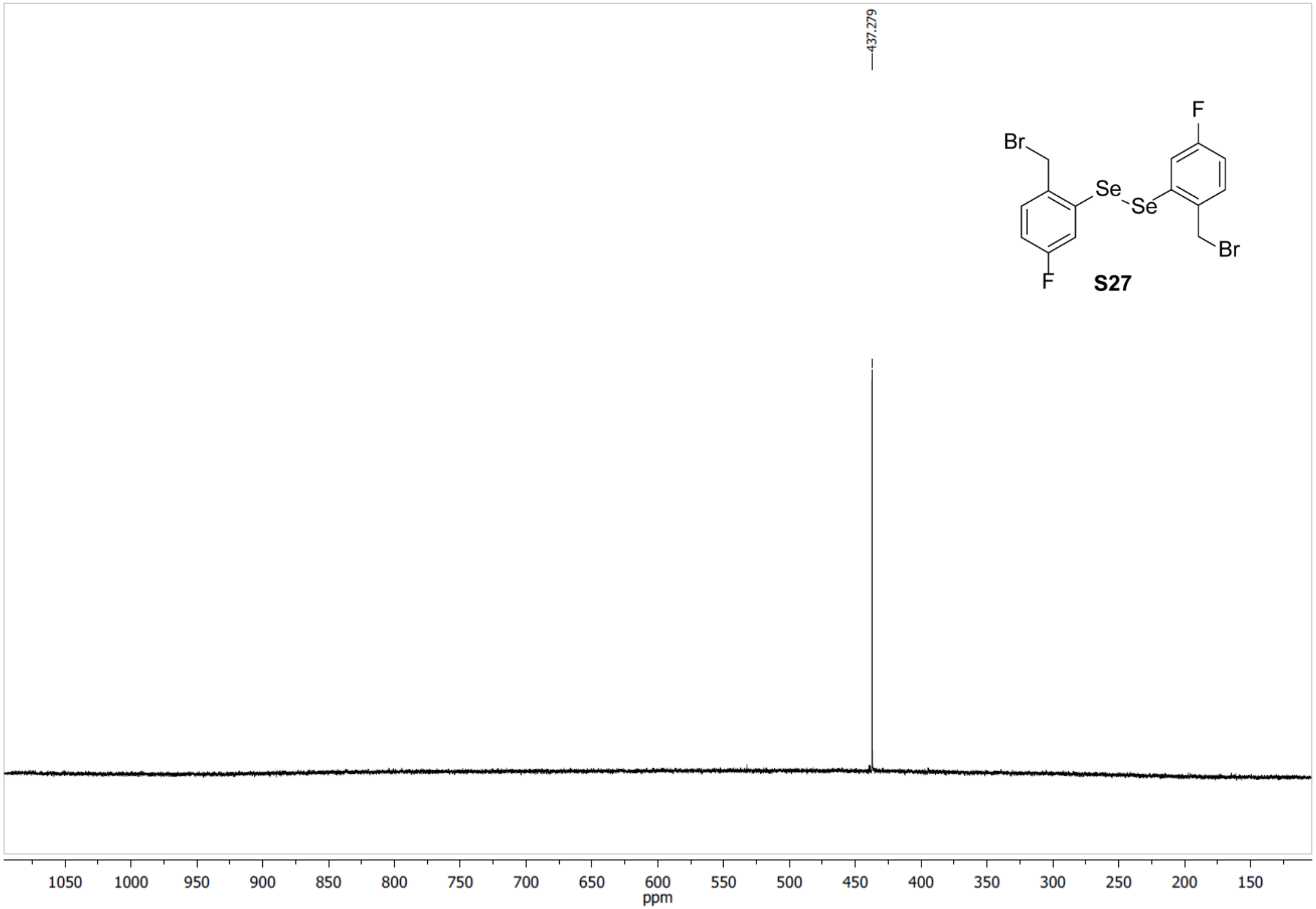

^{1}H NMR in $CDCl_3$

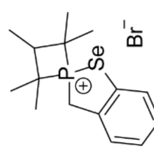

3g-III-Br

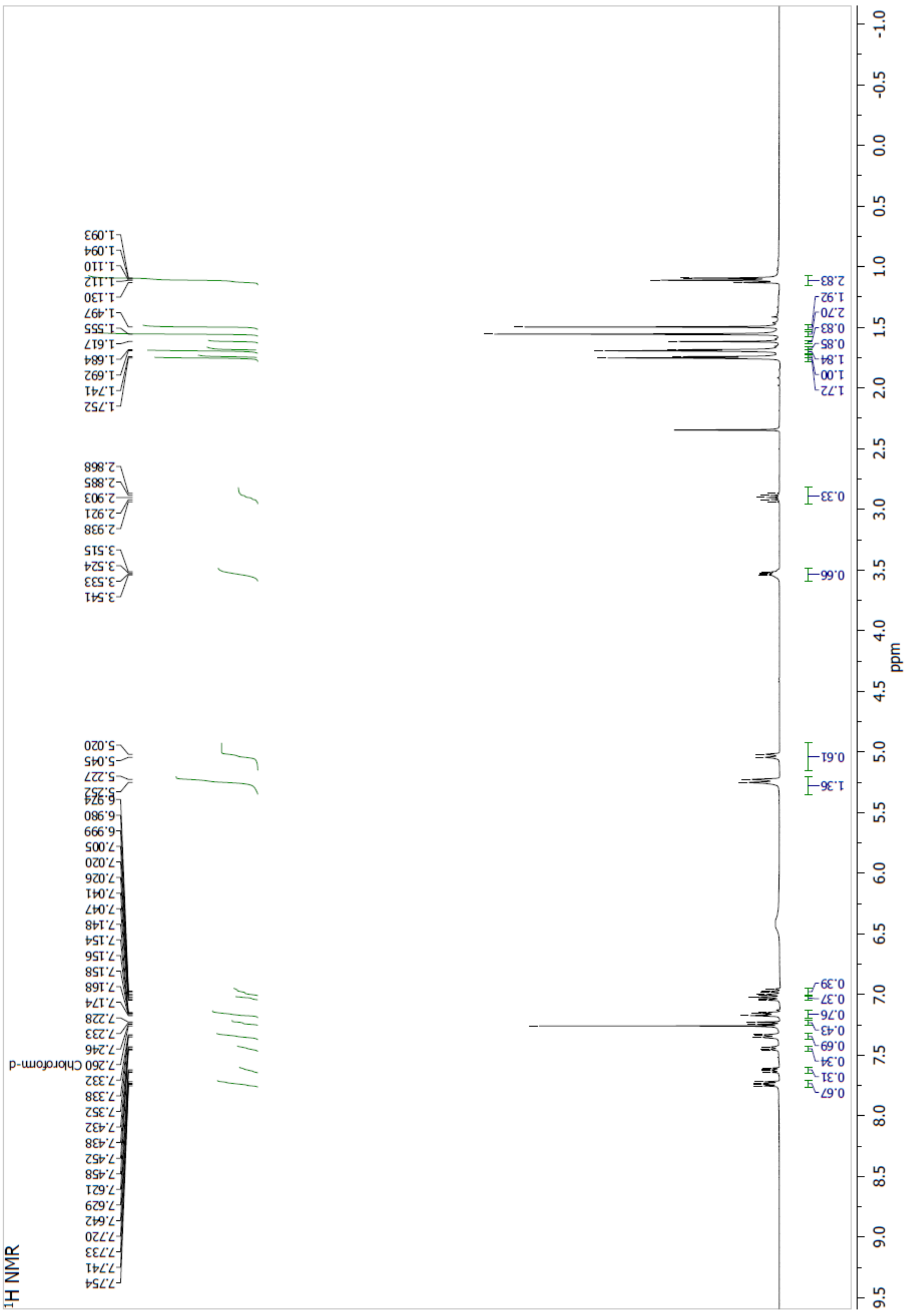

^{19}F NMR in $CDCl_3$

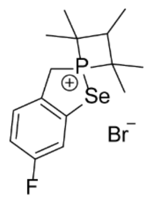

3g-III-Br

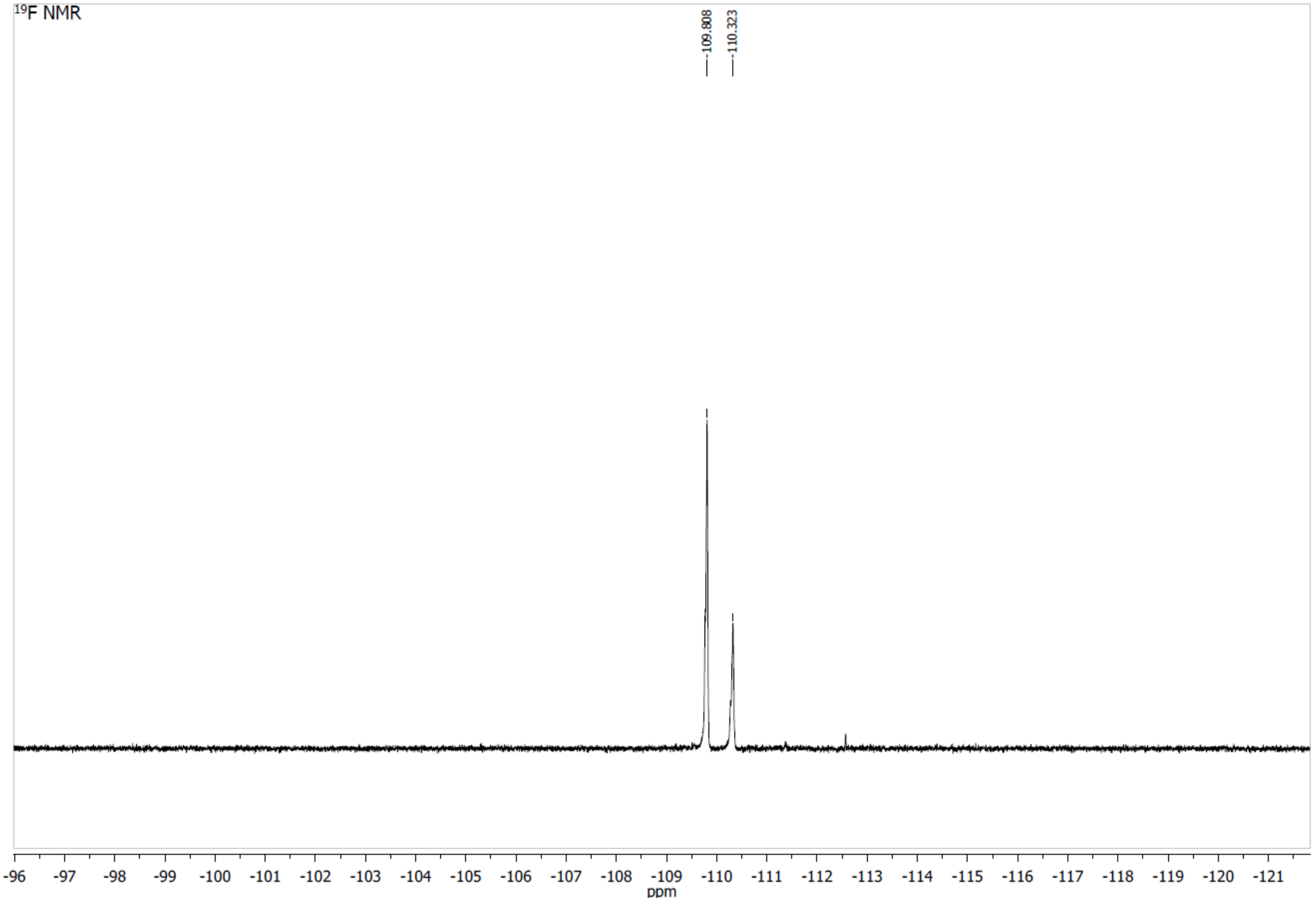

^{31}P NMR in $CDCl_3$

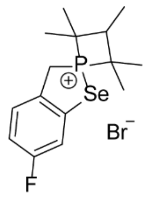

3g-III-Br

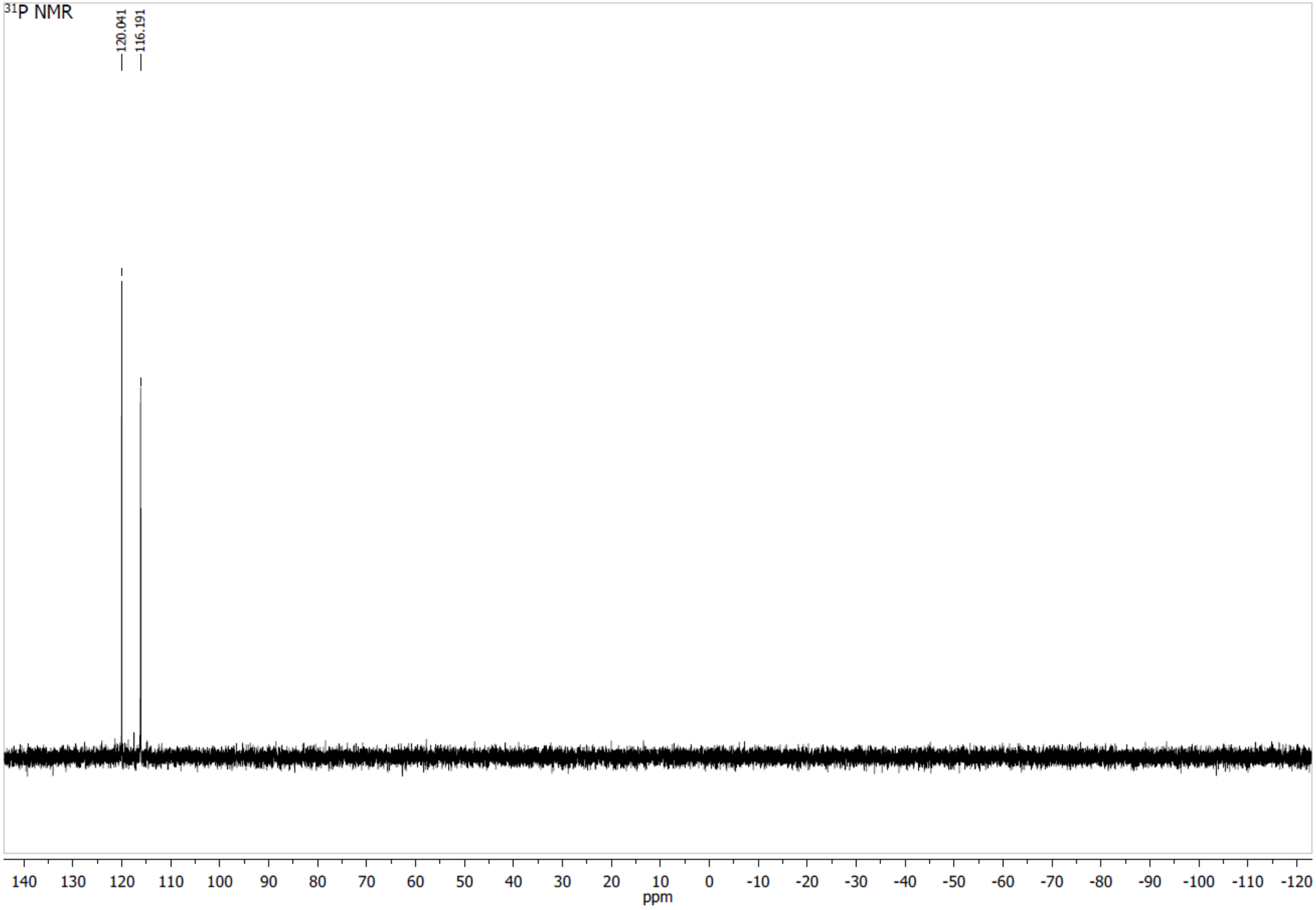

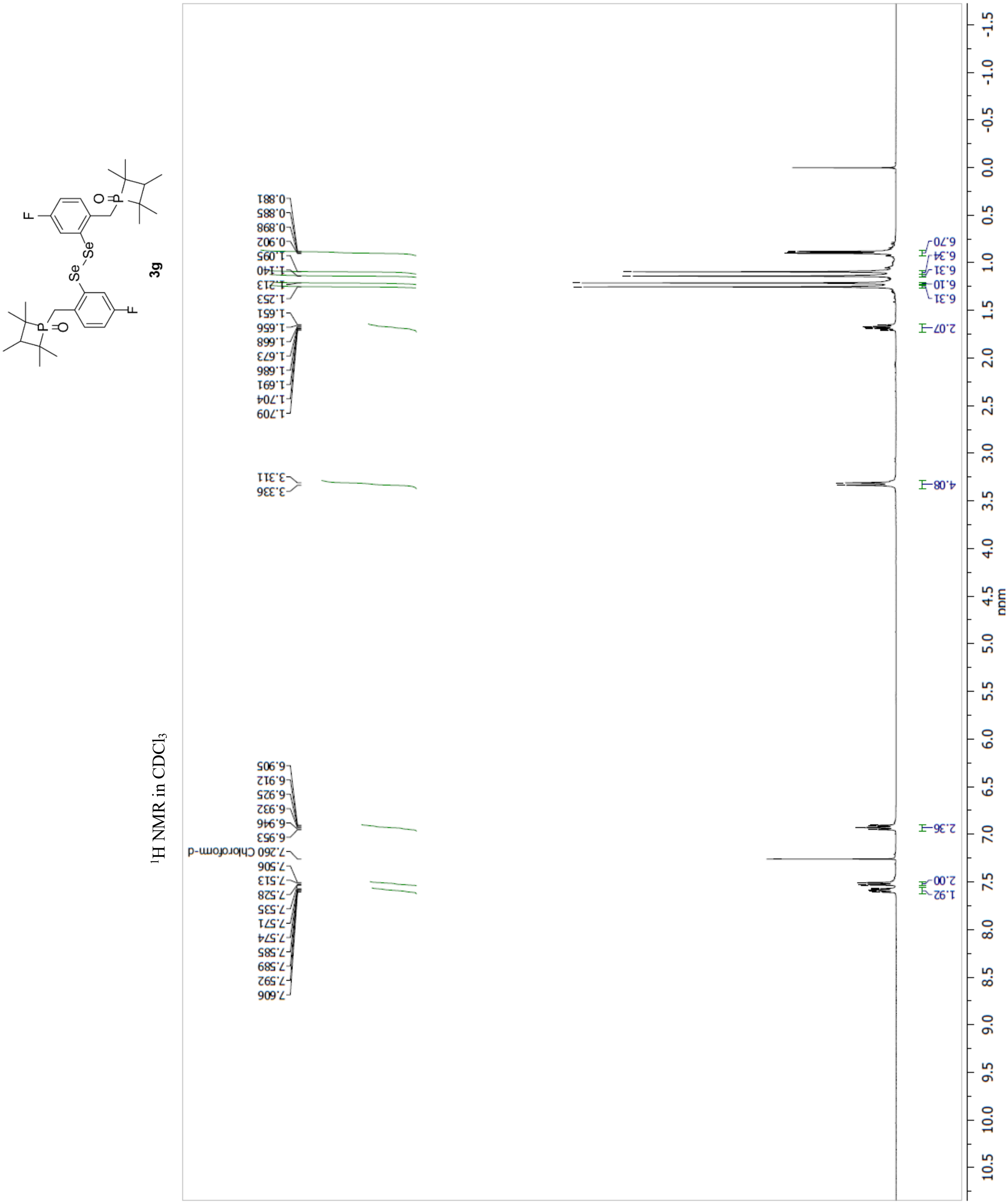

3g

^{1}H NMR in $CDCl_3$

^{13}C NMR in $CDCl_3$

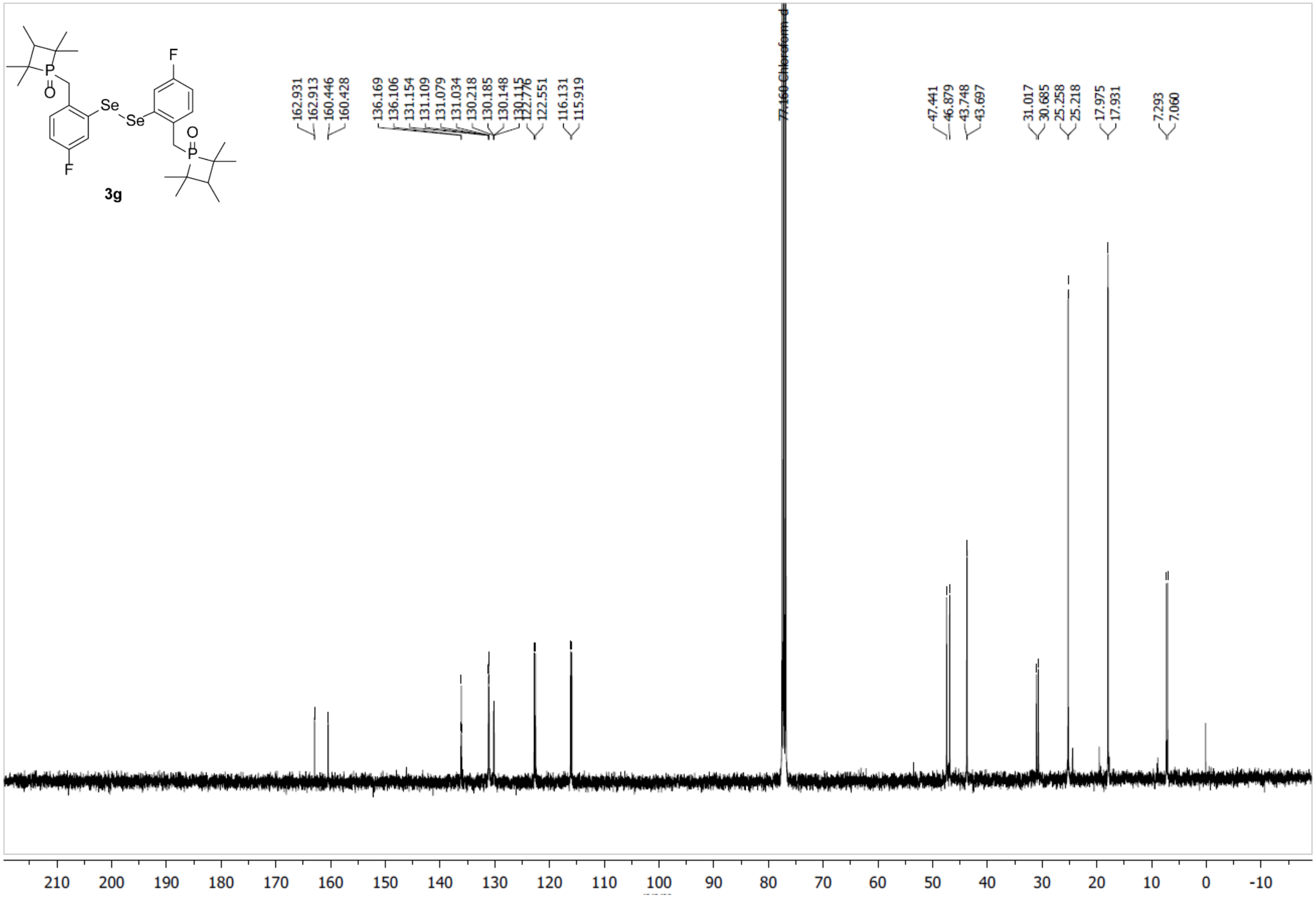

^{19}F NMR in CDCl$_3$

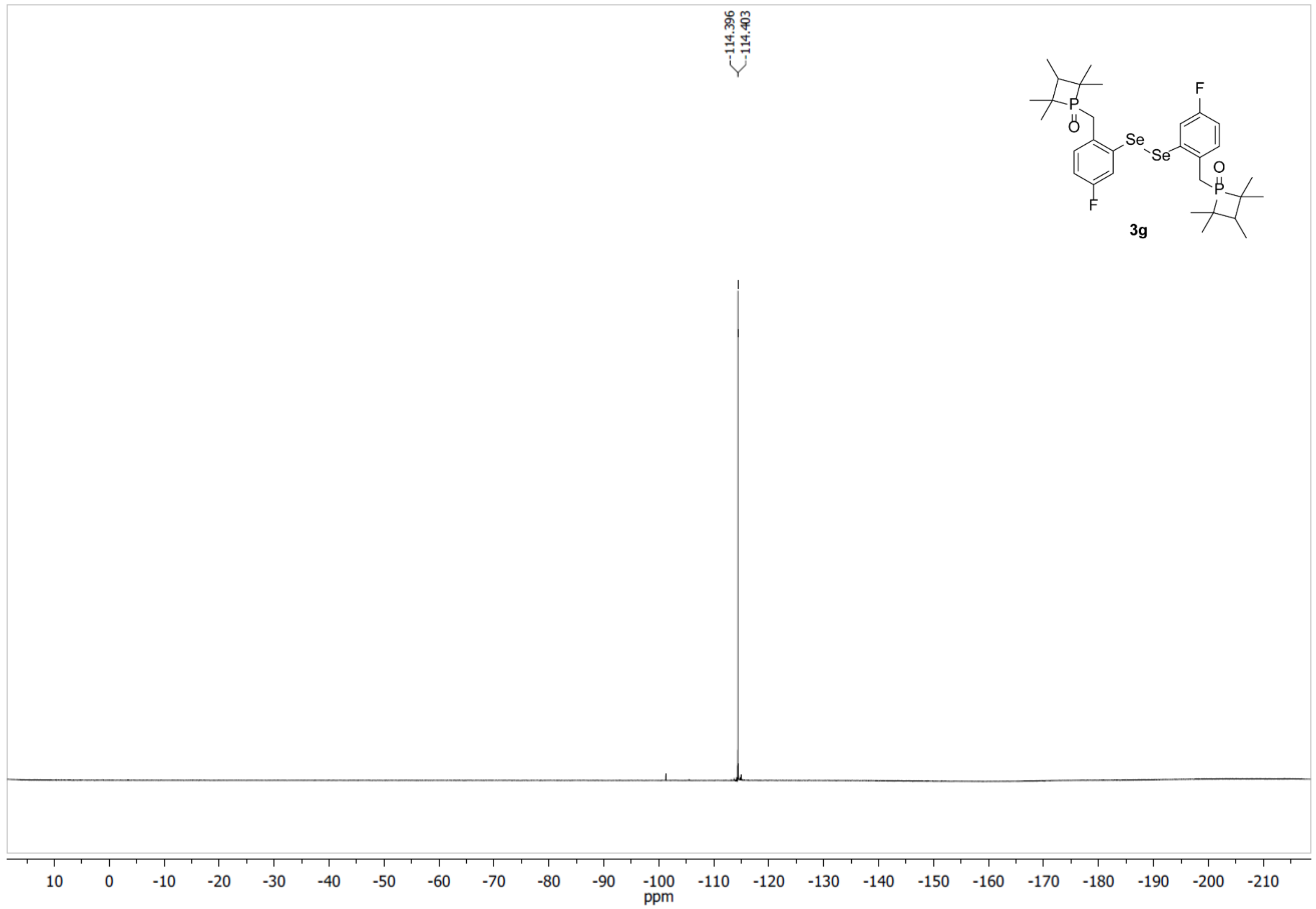

^{31}P NMR in $CDCl_3$

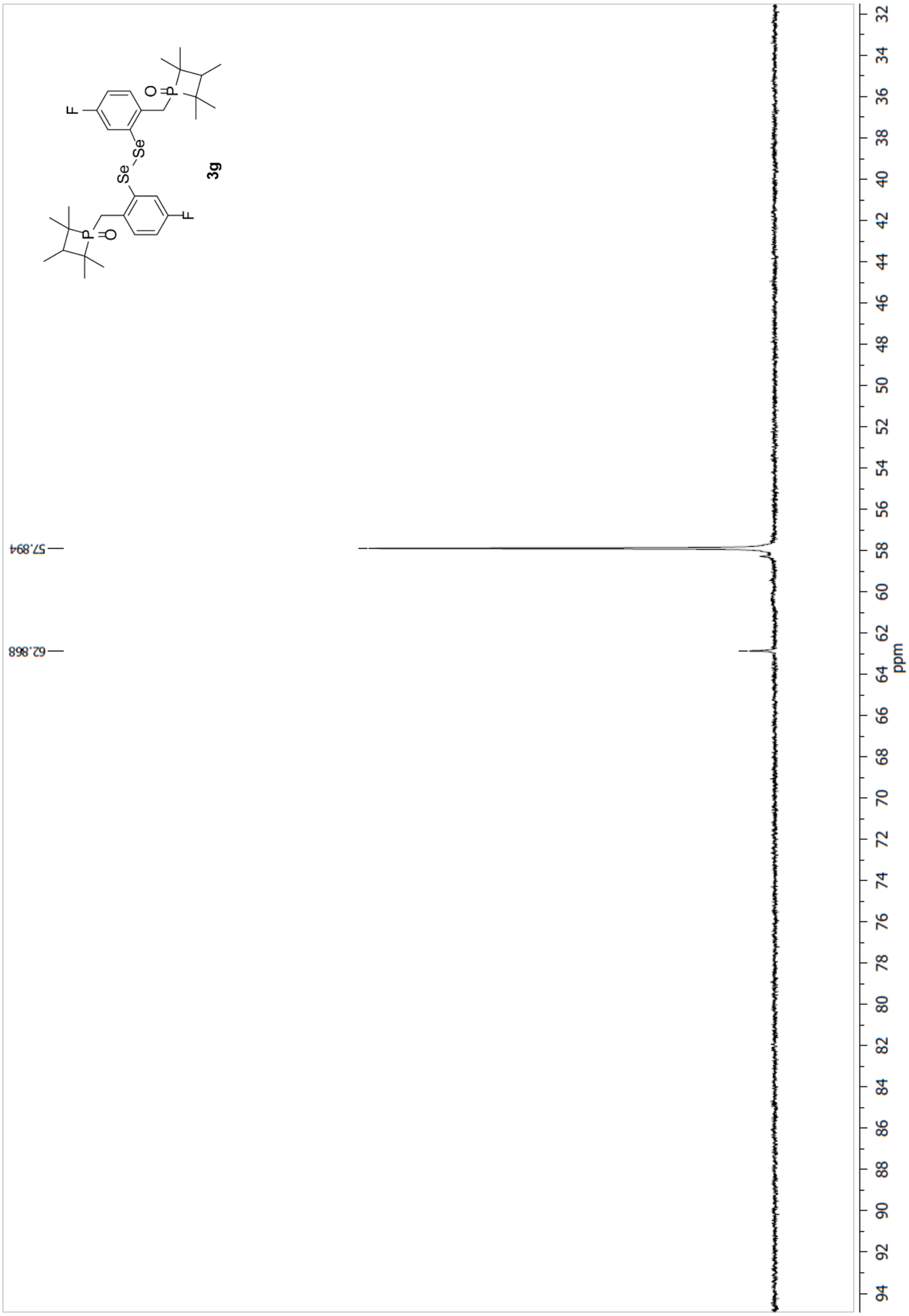

^{77}Se NMR in CDCl$_3$

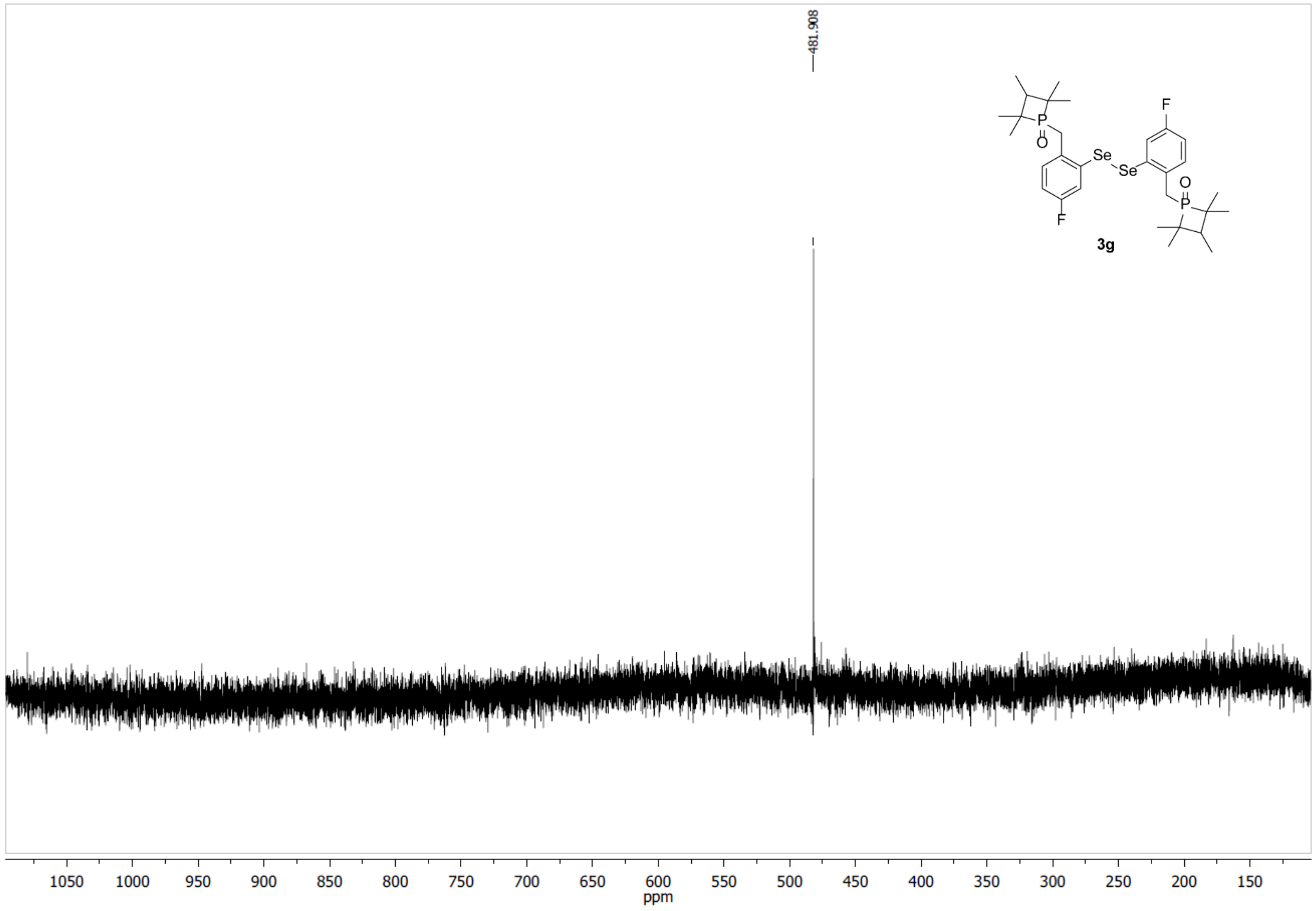

^{1}H NMR in $CDCl_3$

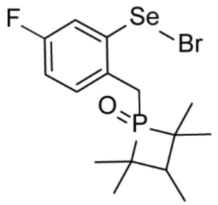

3l

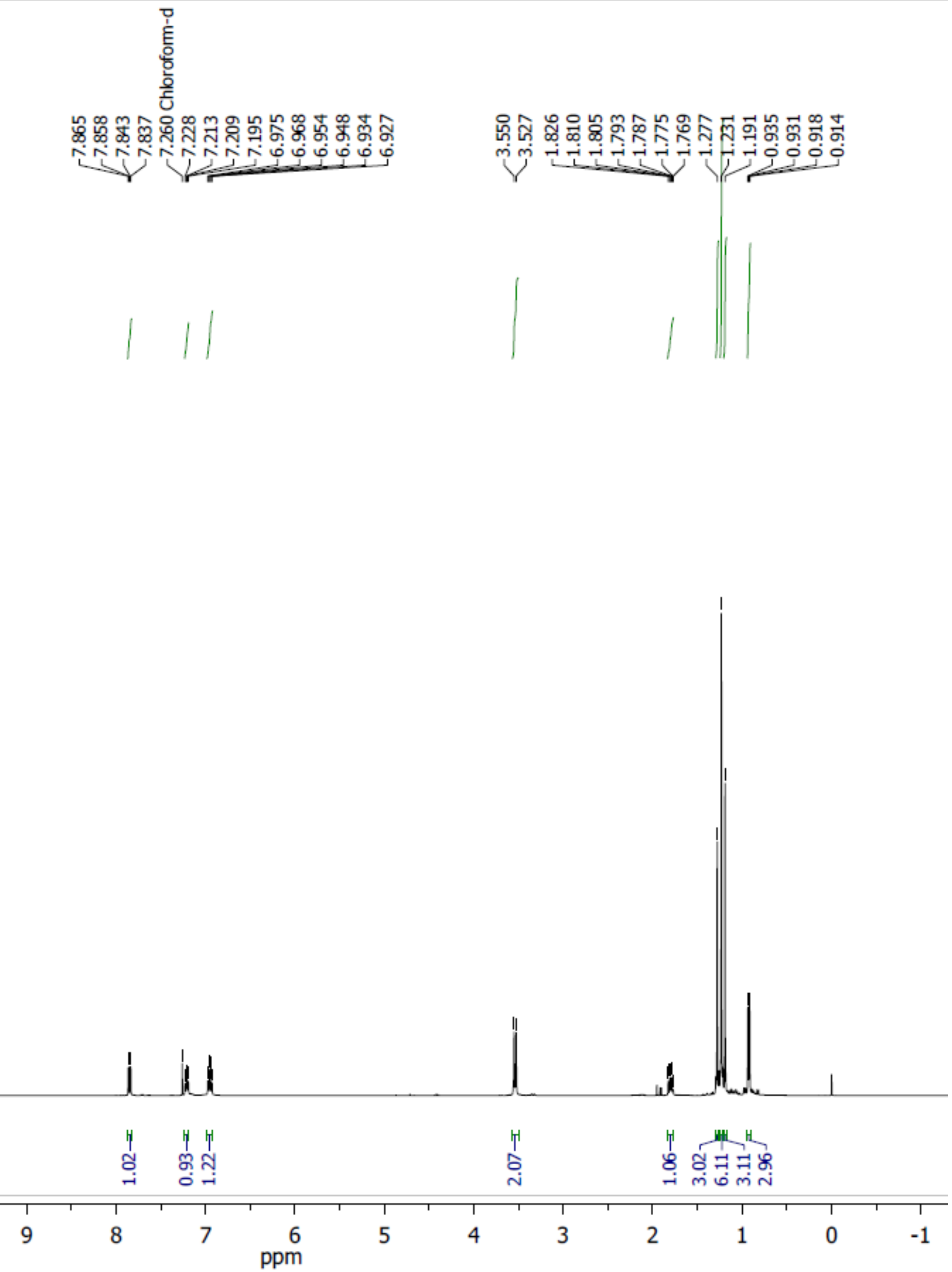

^{13}C NMR in $CDCl_3$

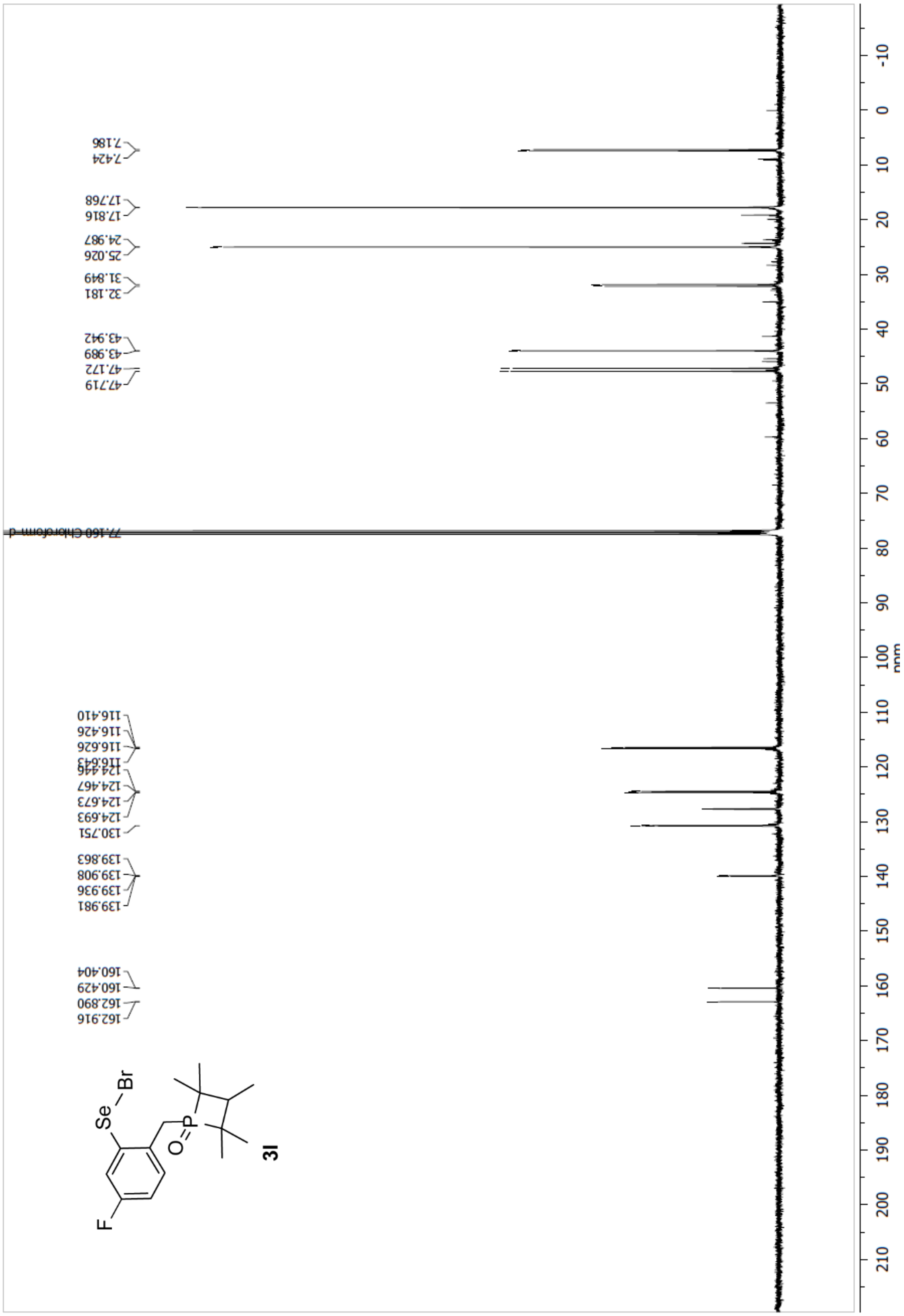

^{19}F NMR in $CDCl_3$

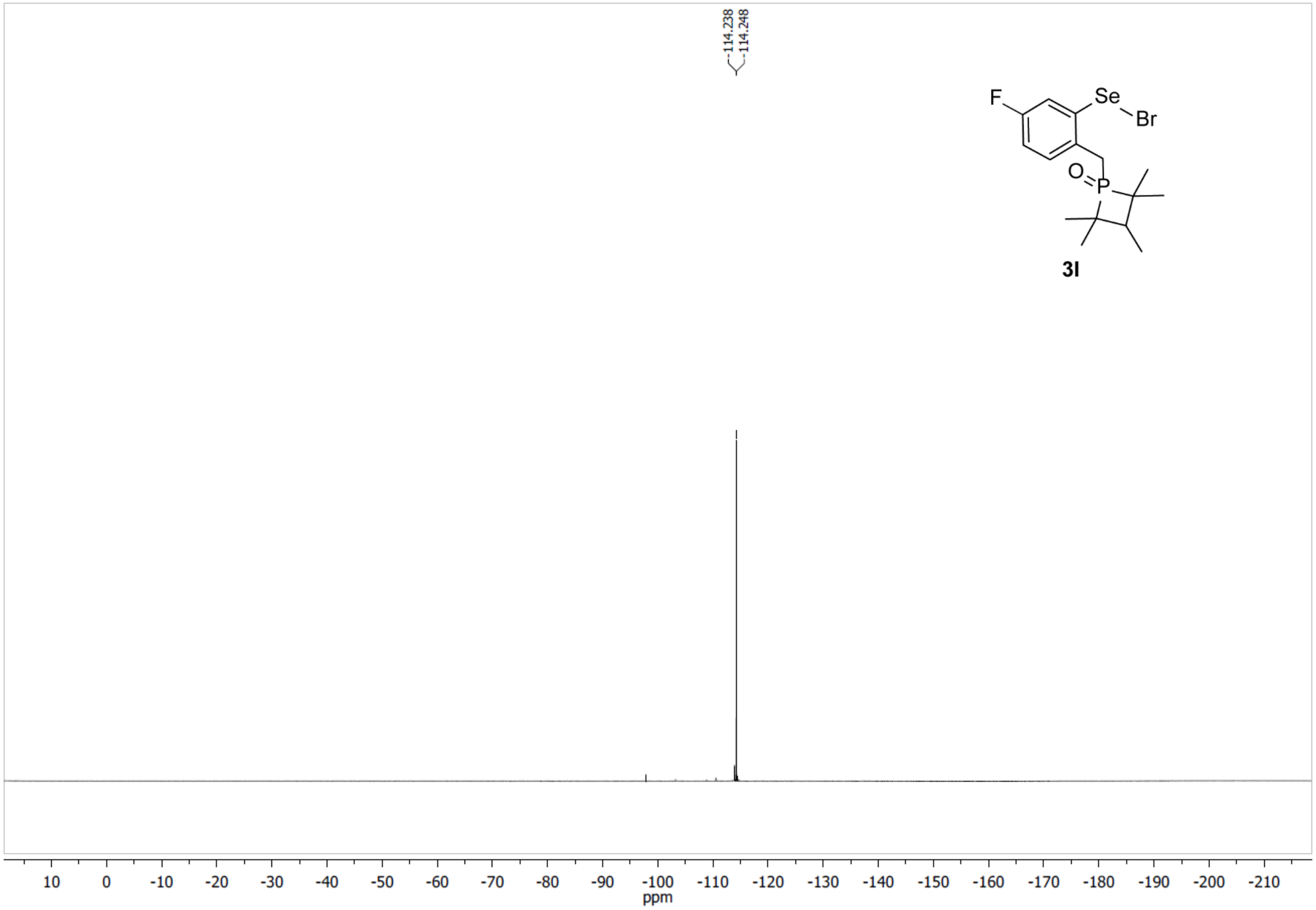

^{31}P NMR in $CDCl_3$

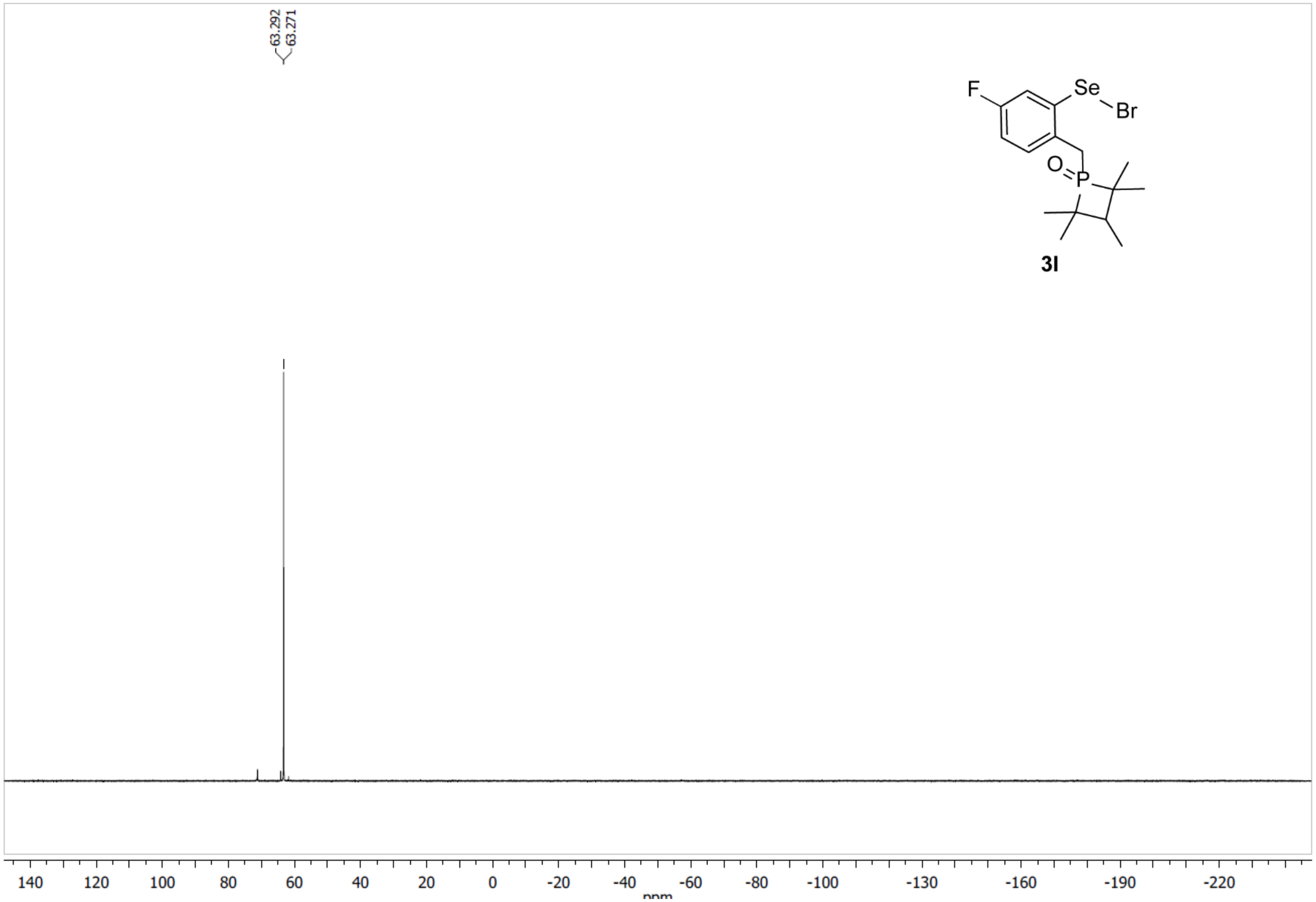

^{77}Se NMR in CDCl$_3$

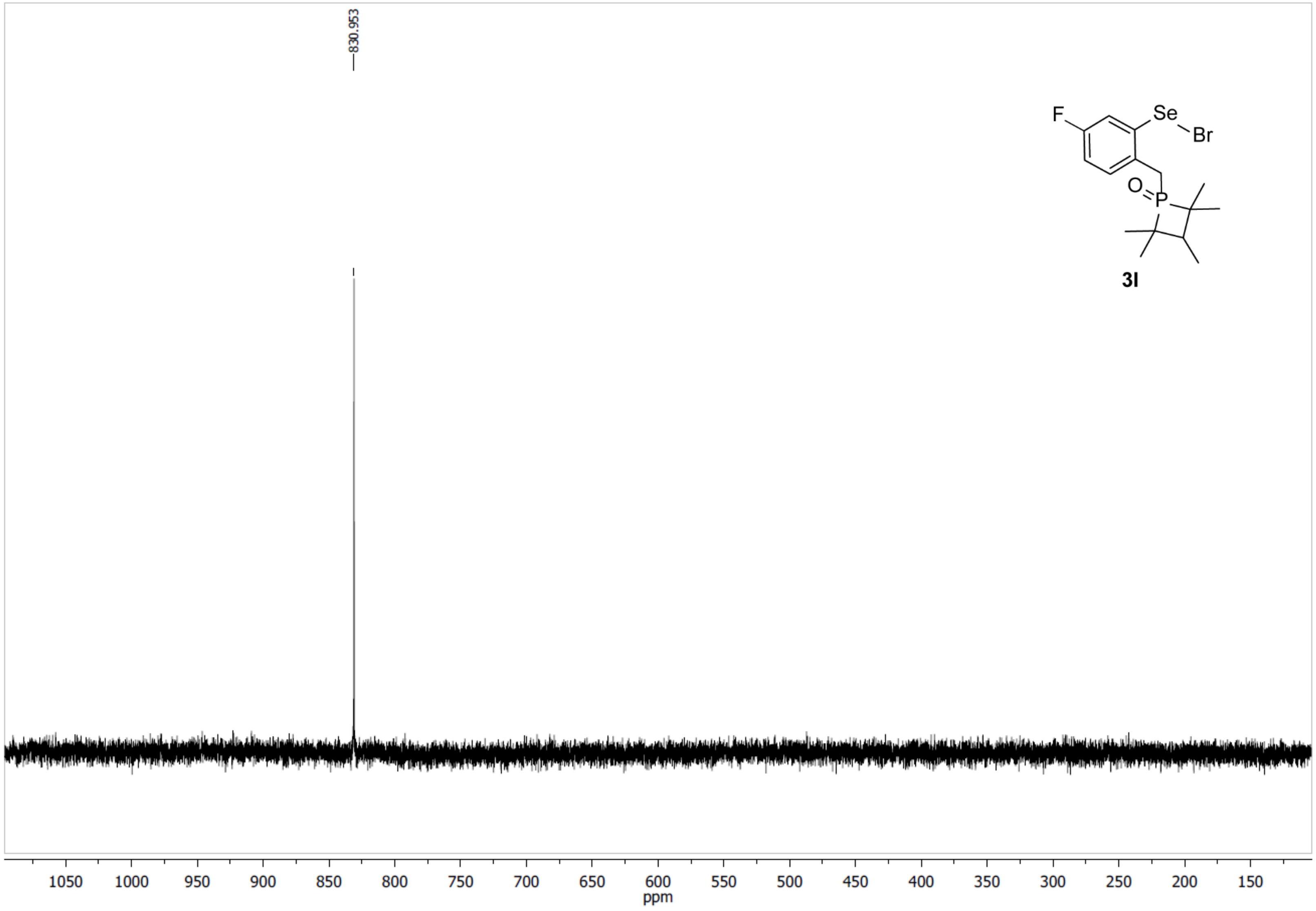

3l

^1H NMR in CDCl$_3$

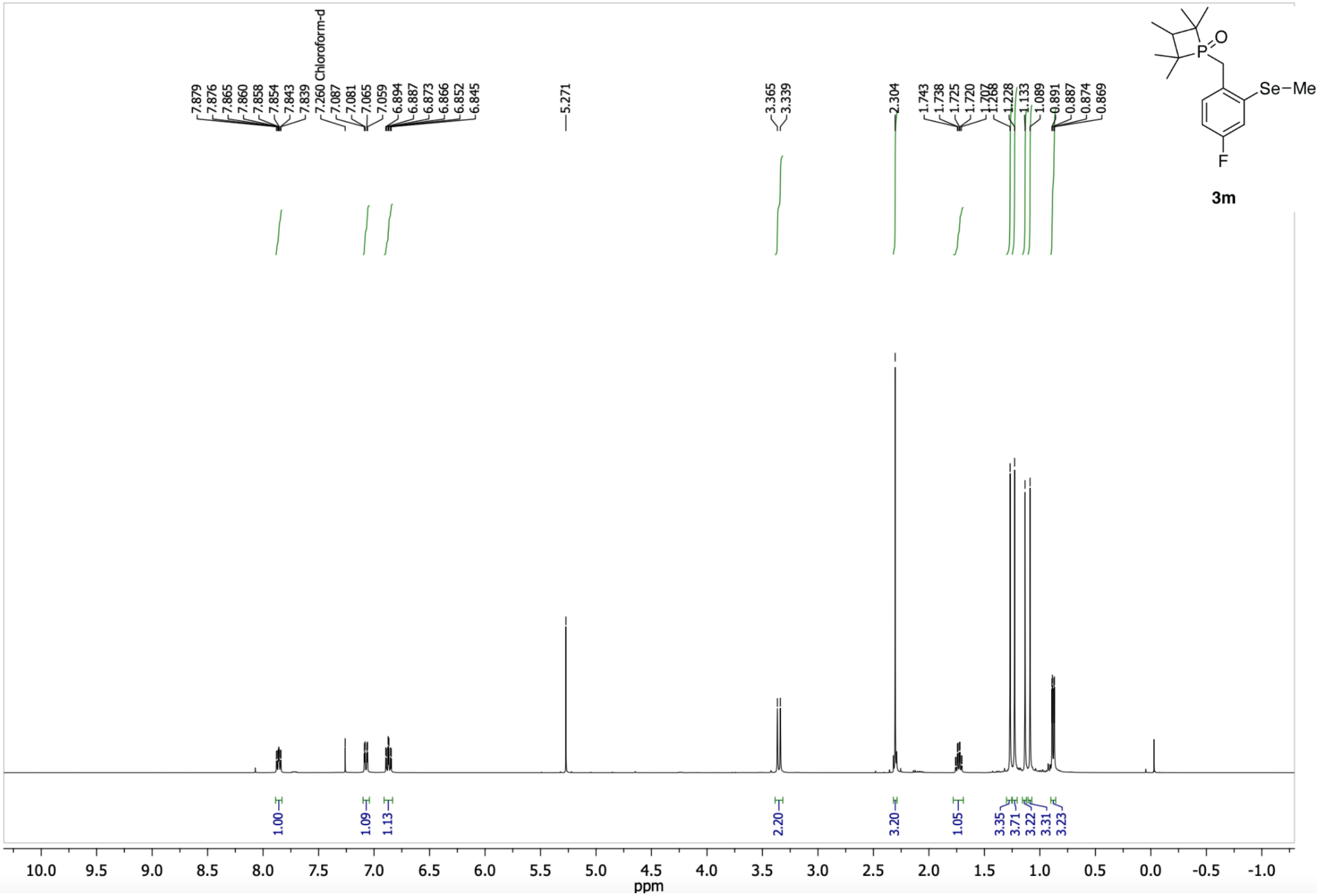

3m

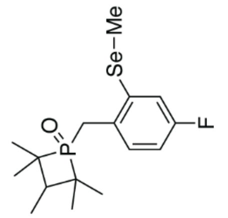

3m

^{13}C NMR in $CDCl_3$

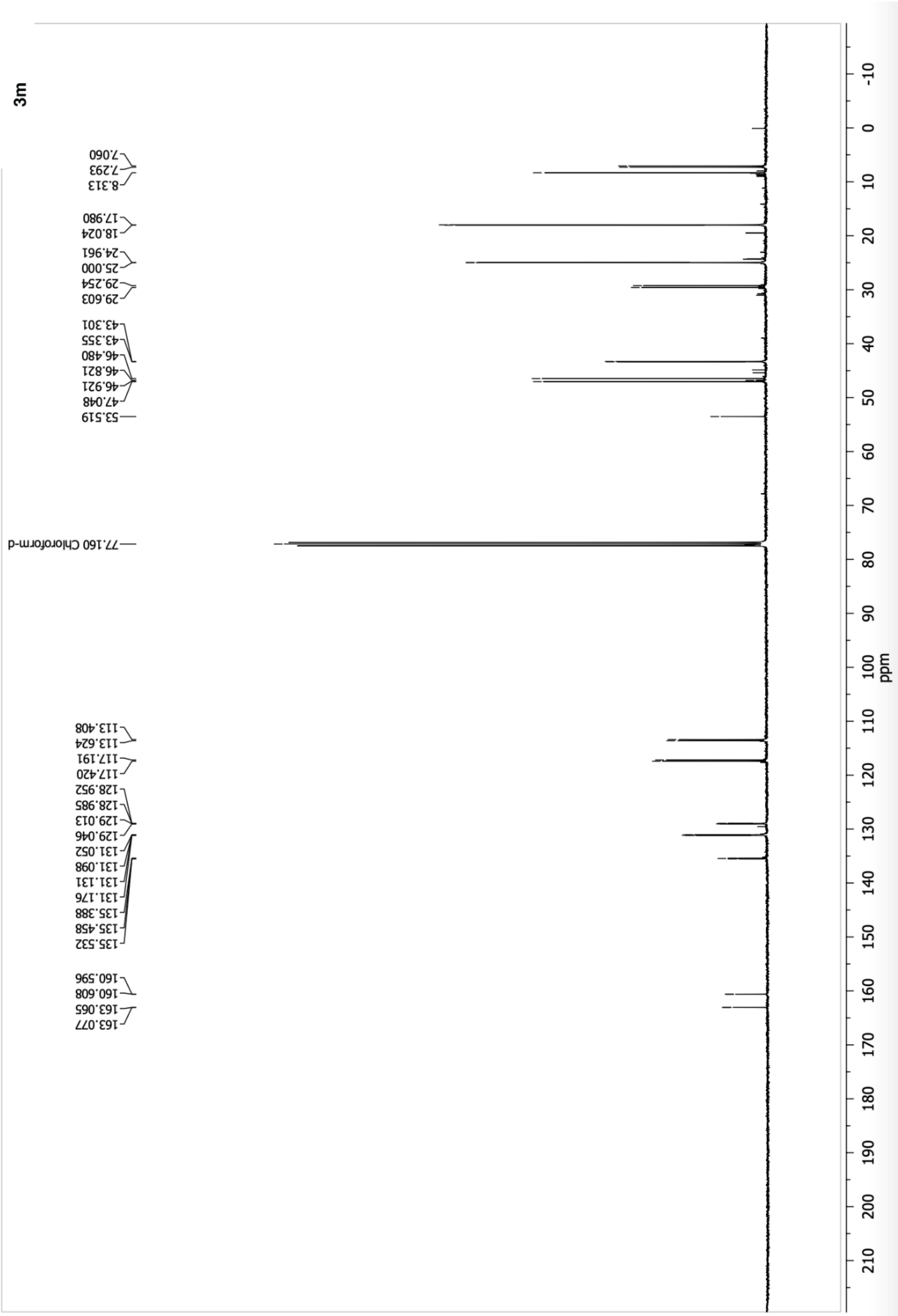

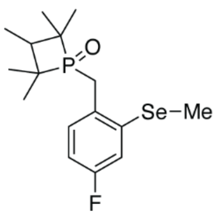

3m

^{19}F NMR in $CDCl_3$

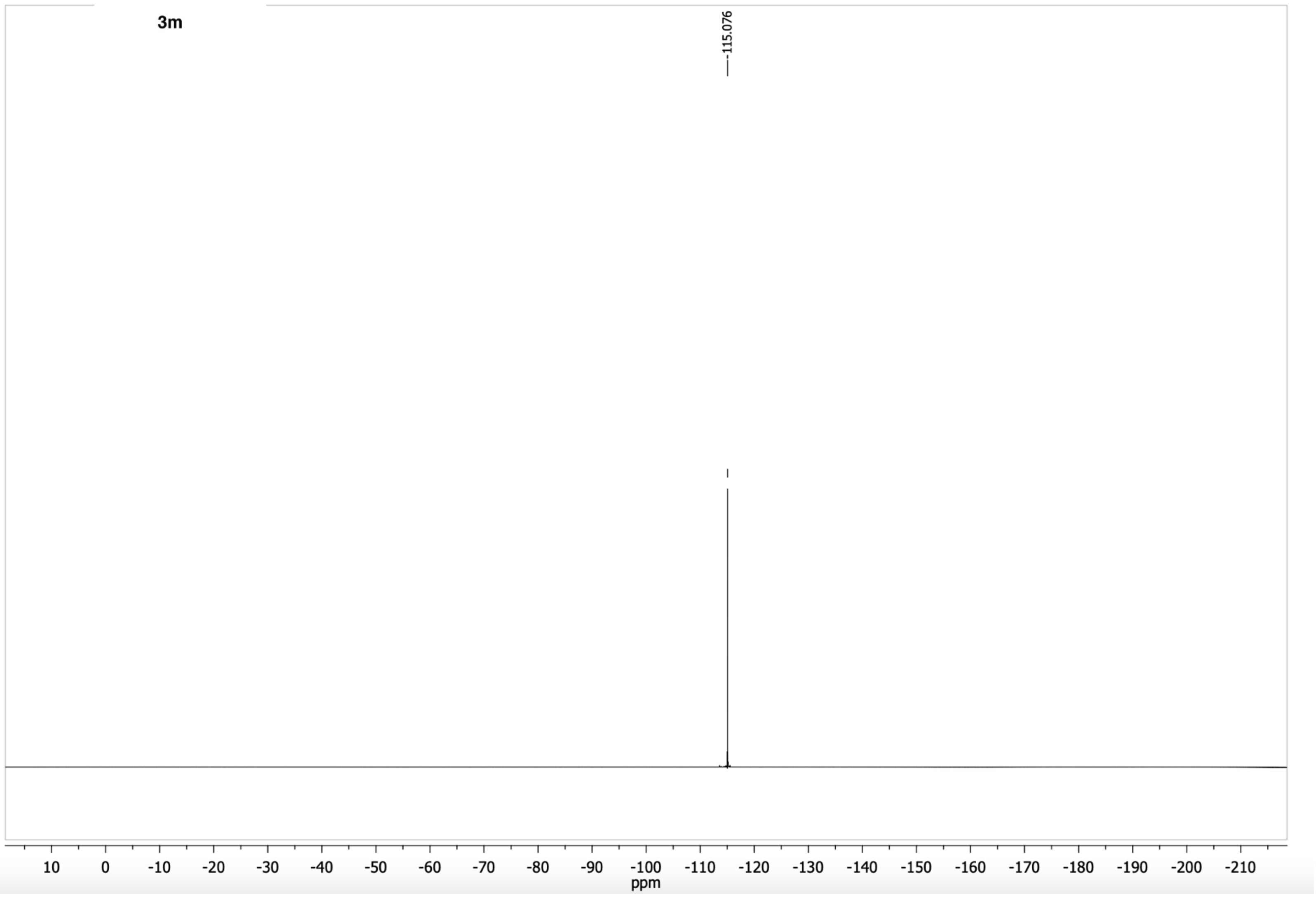

^{31}P NMR in $CDCl_3$

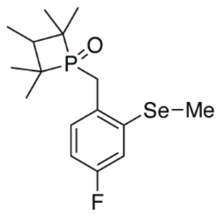

3m

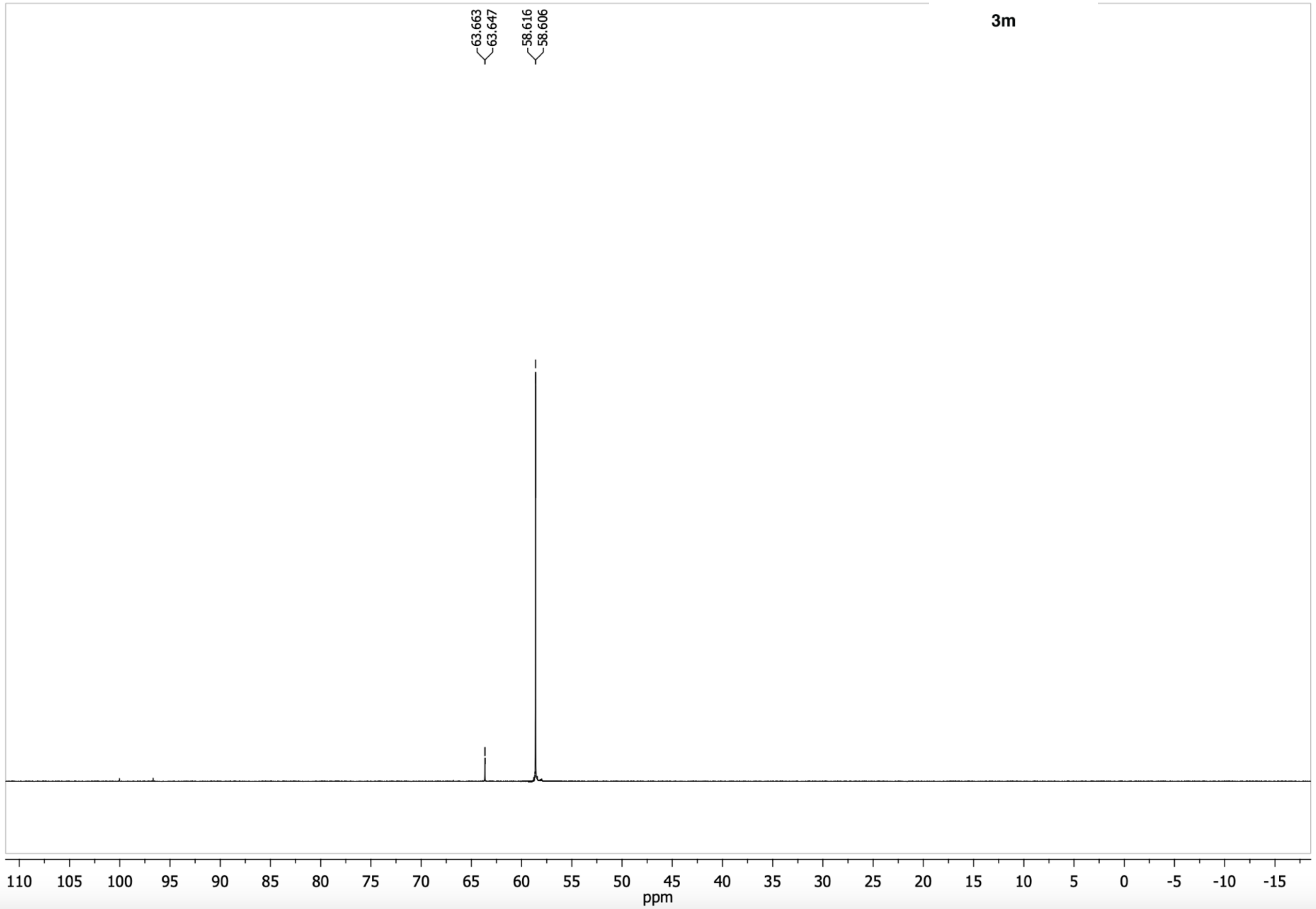

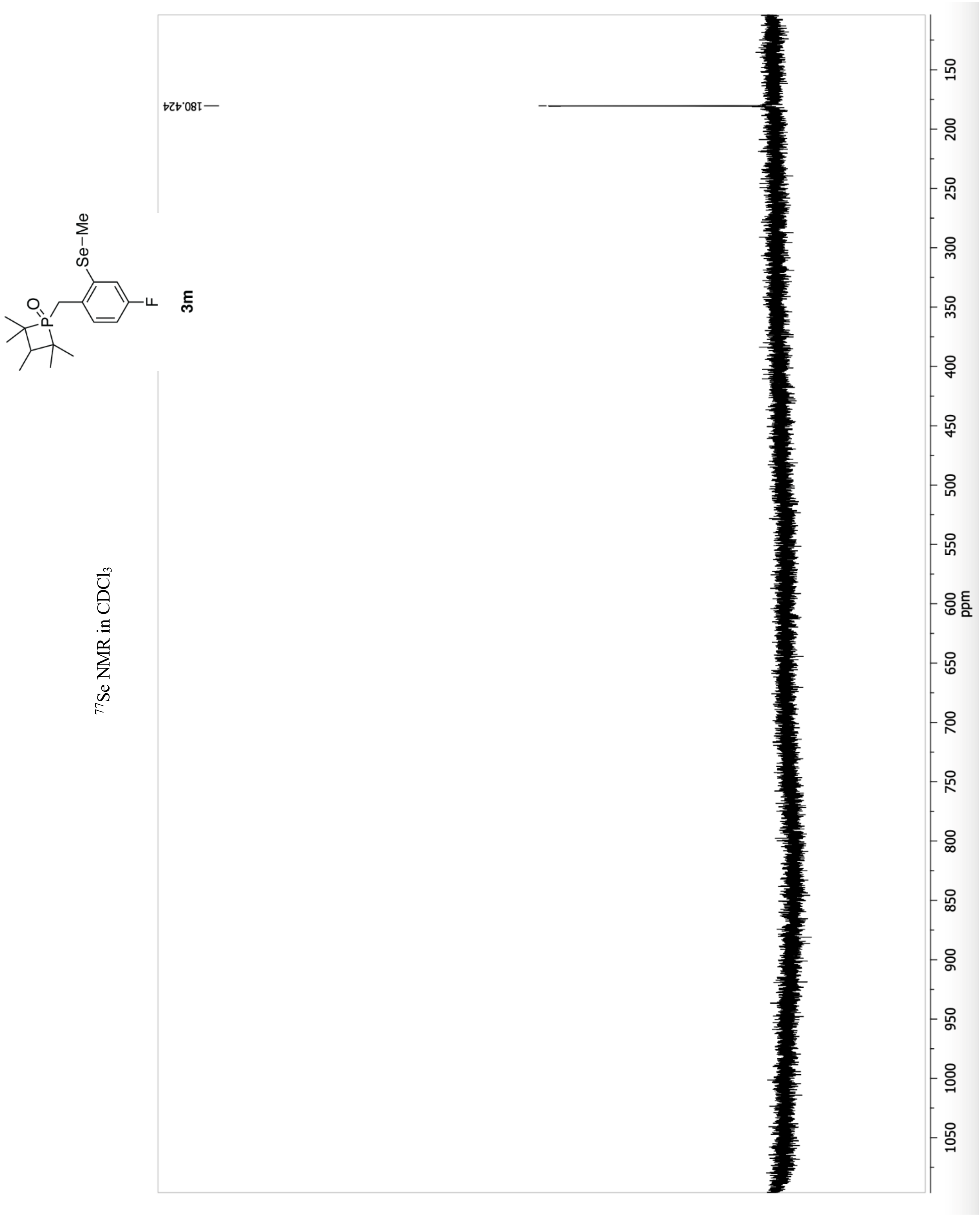

O
P
Se−Me
F
3m
77Se NMR in CDCl3
180.424
150
200
250
300
350
400
450
500
550
600
650
700
750
800
850
900
950
1000
1050
ppm

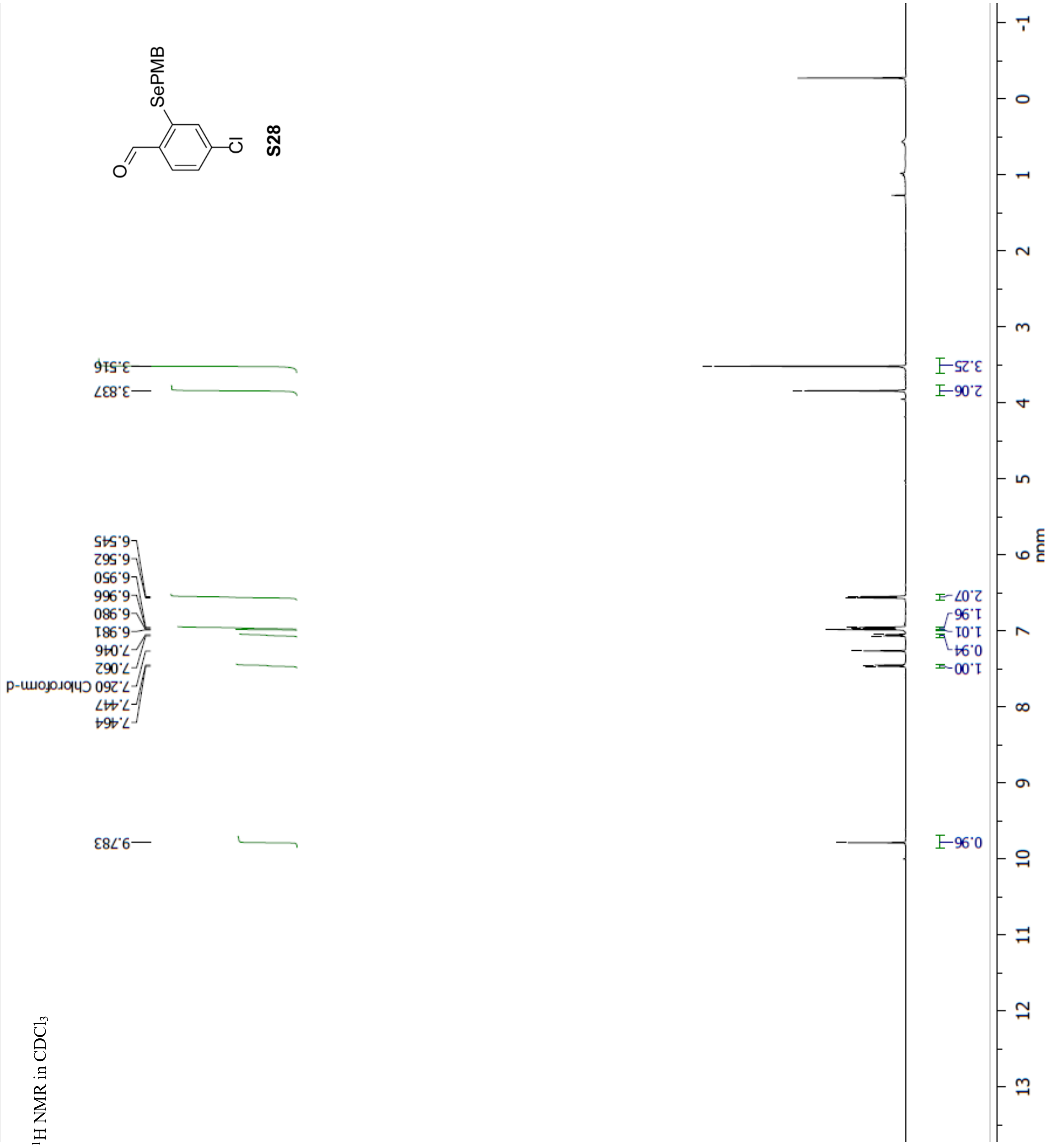

^{1}H NMR in $CDCl_3$

^{13}C NMR in $CDCl_3$

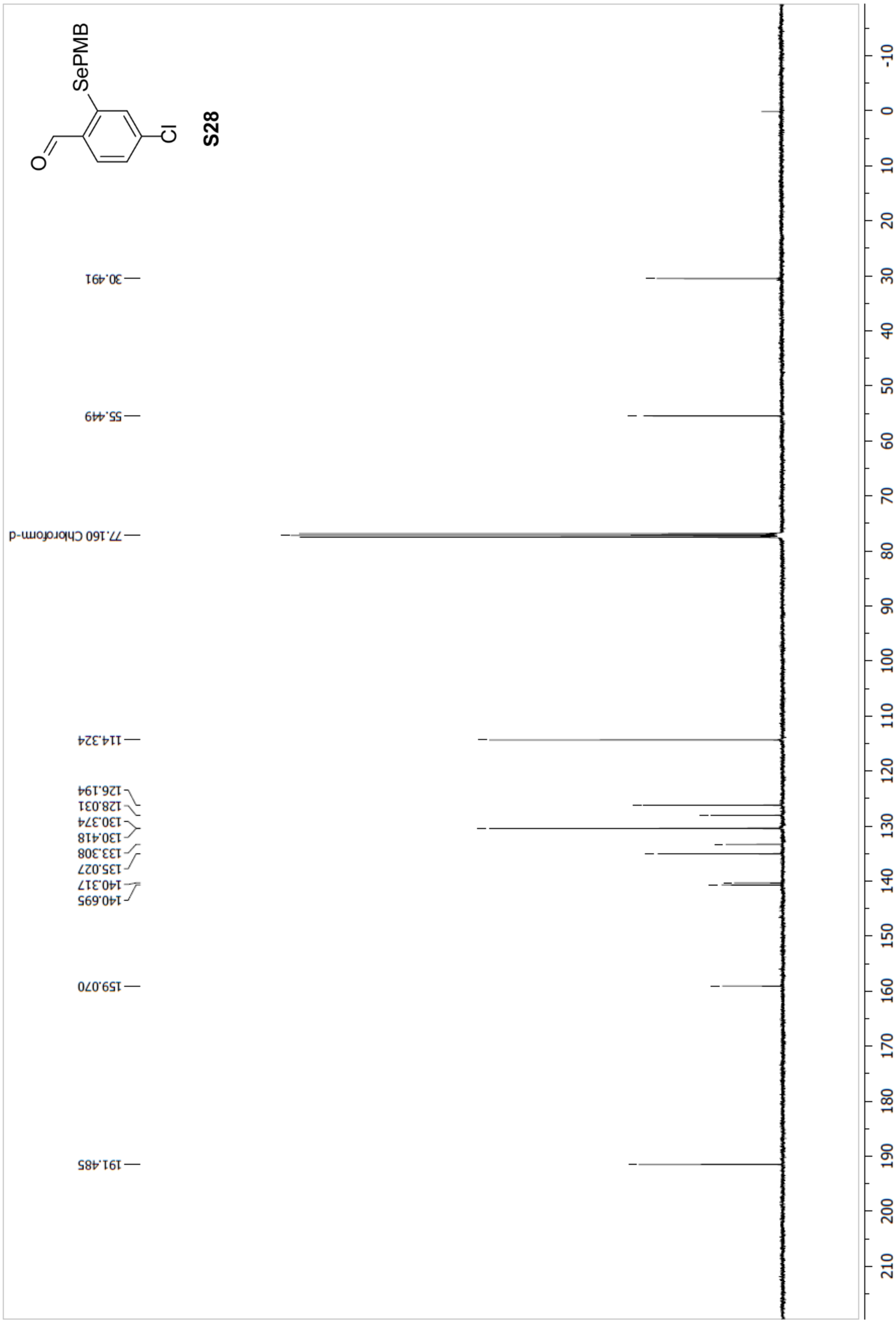

^{77}Se NMR in $CDCl_3$

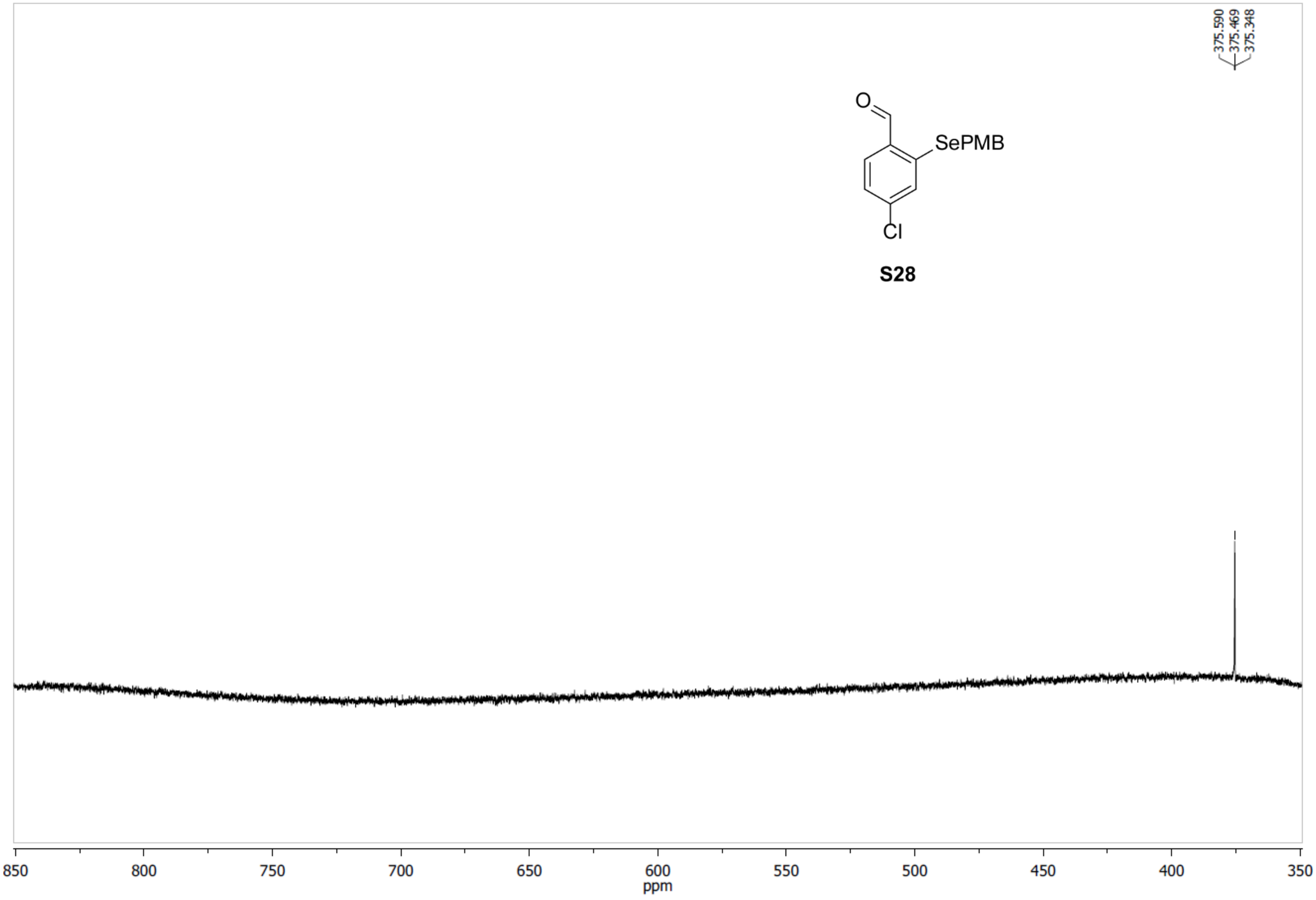

^{1}H NMR in $CDCl_3$

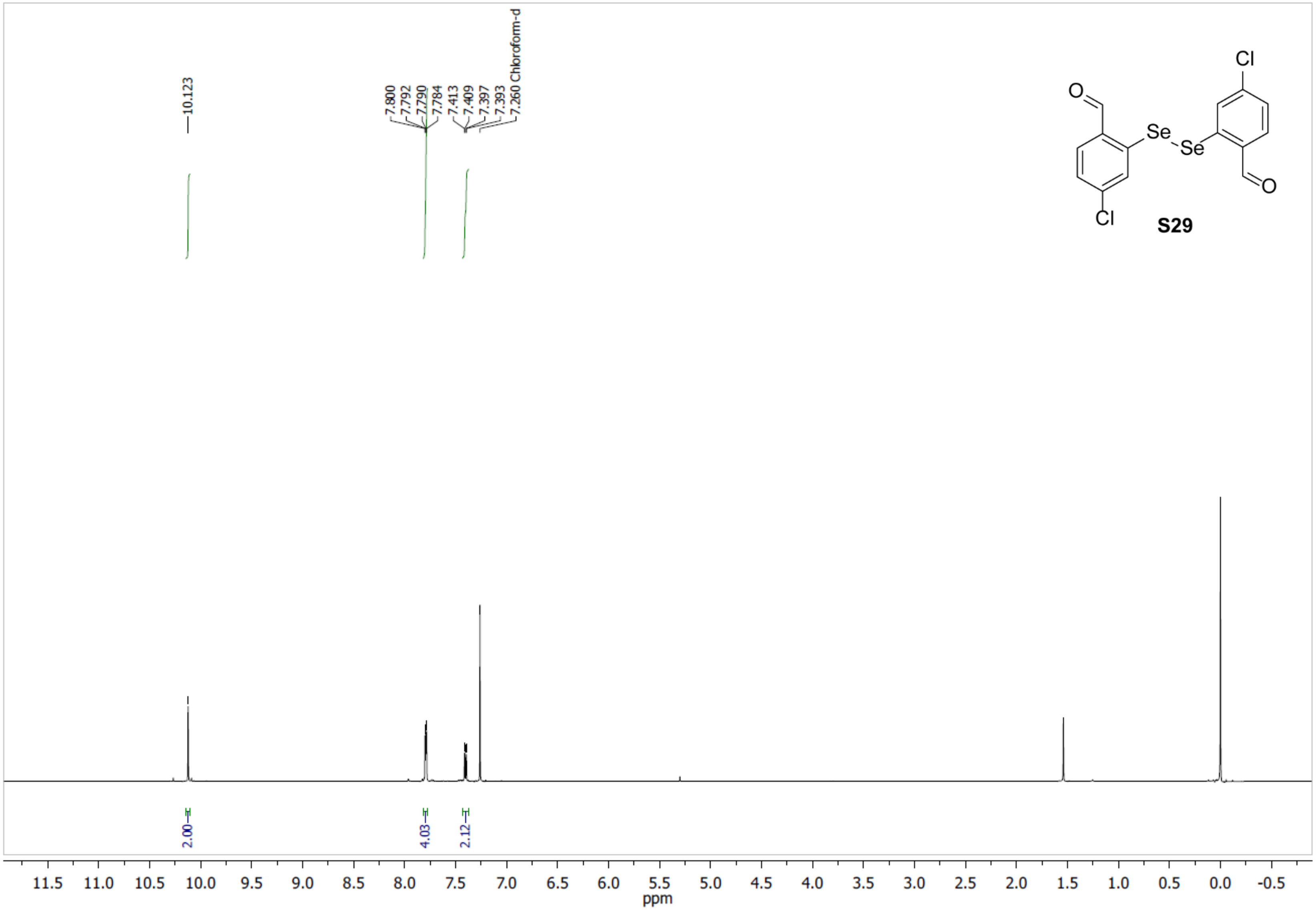

^{13}C NMR in $CDCl_3$

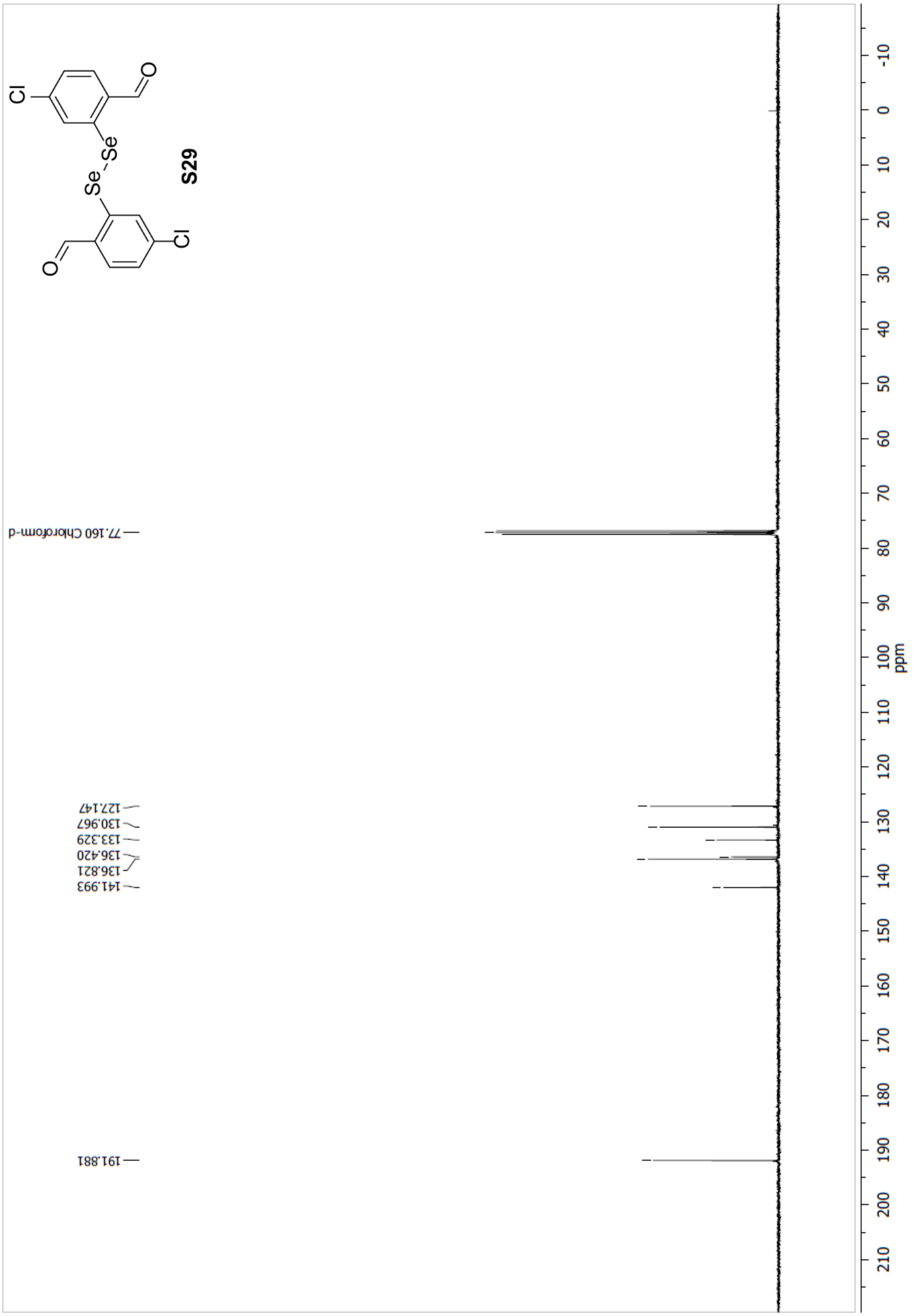

^{77}Se NMR in $CDCl_3$

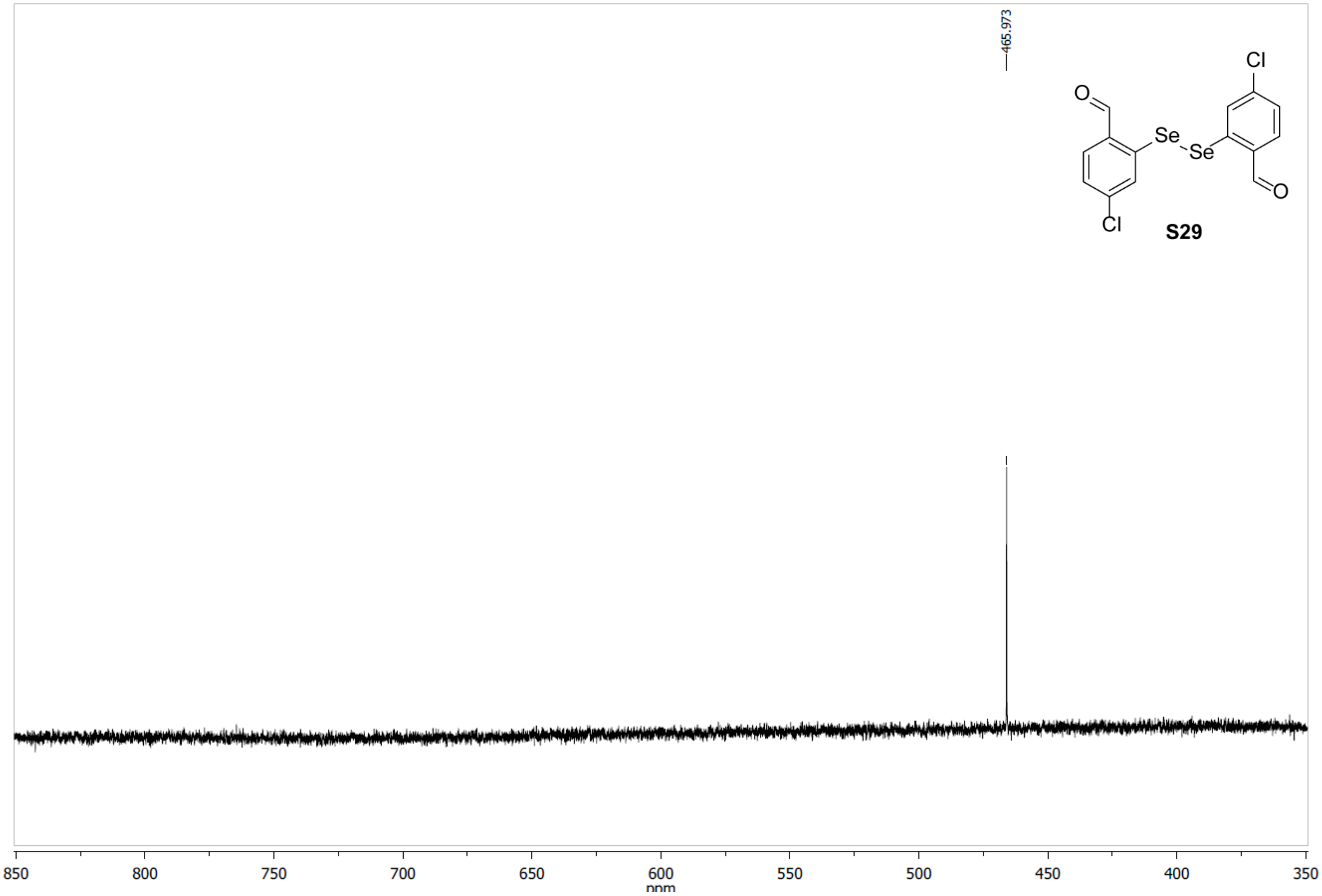

^{1}H NMR in DMSO-d_6

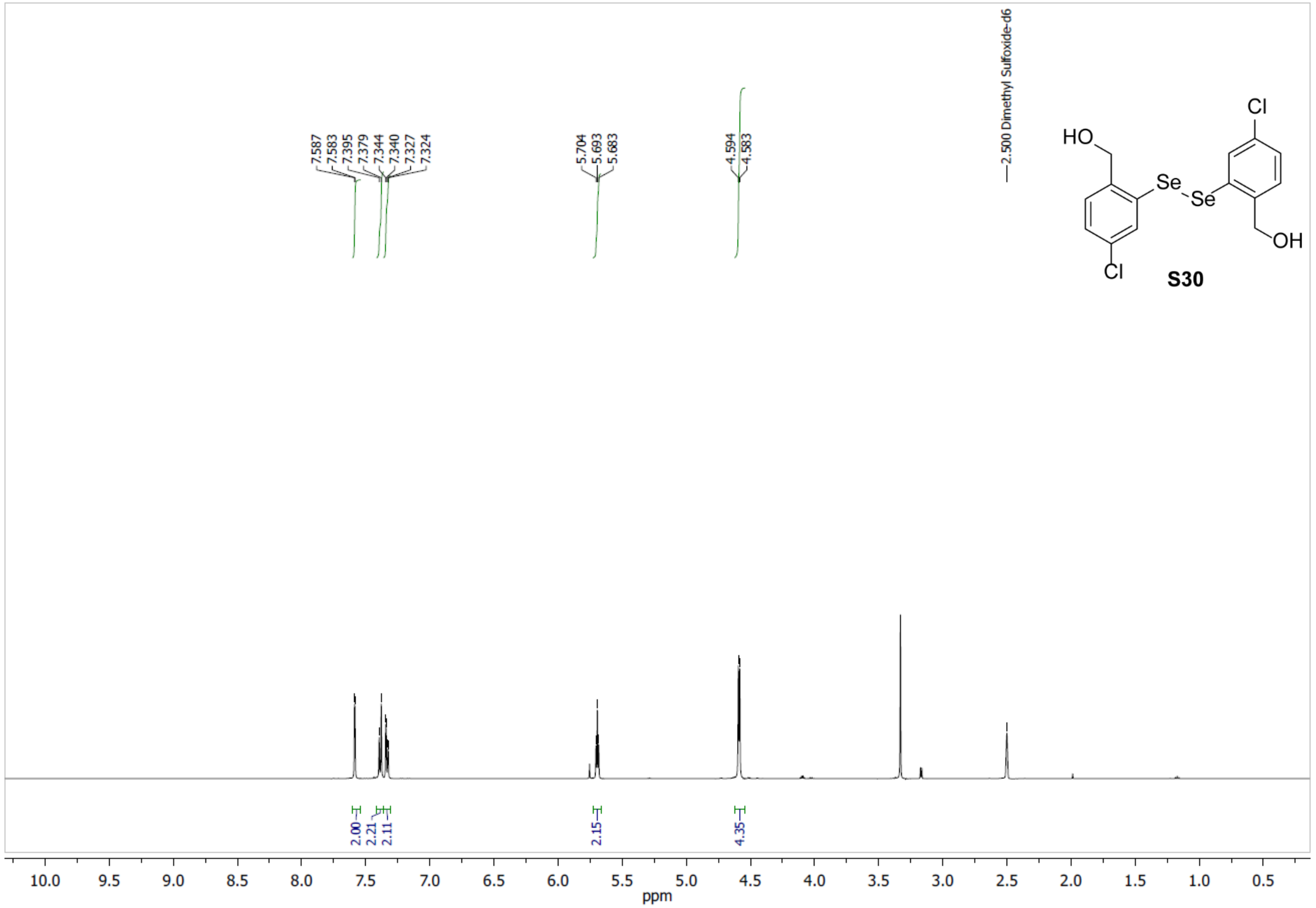

^{13}C NMR in DMSO-d_6

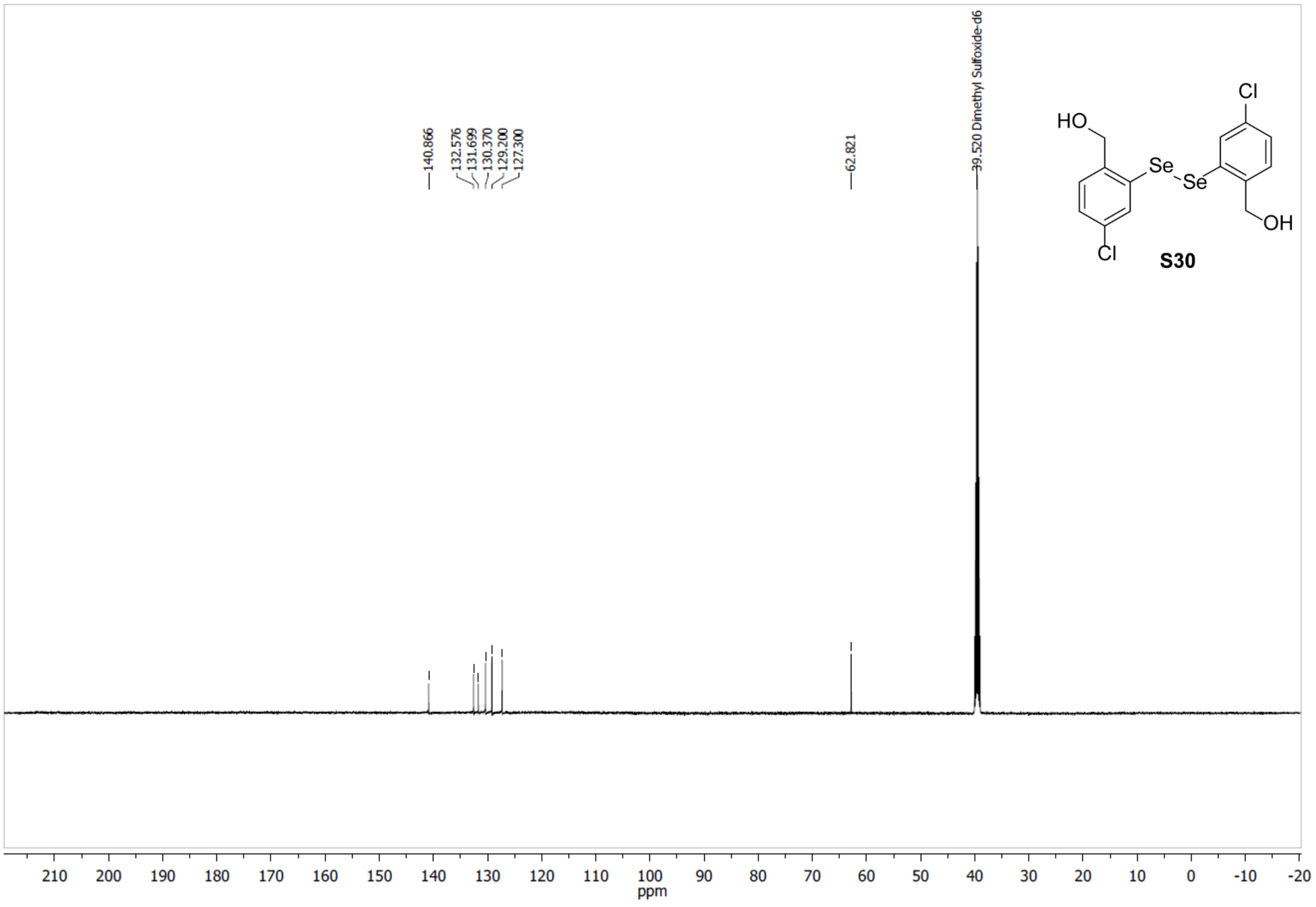

S30

^{77}Se NMR in DMSO-d_6

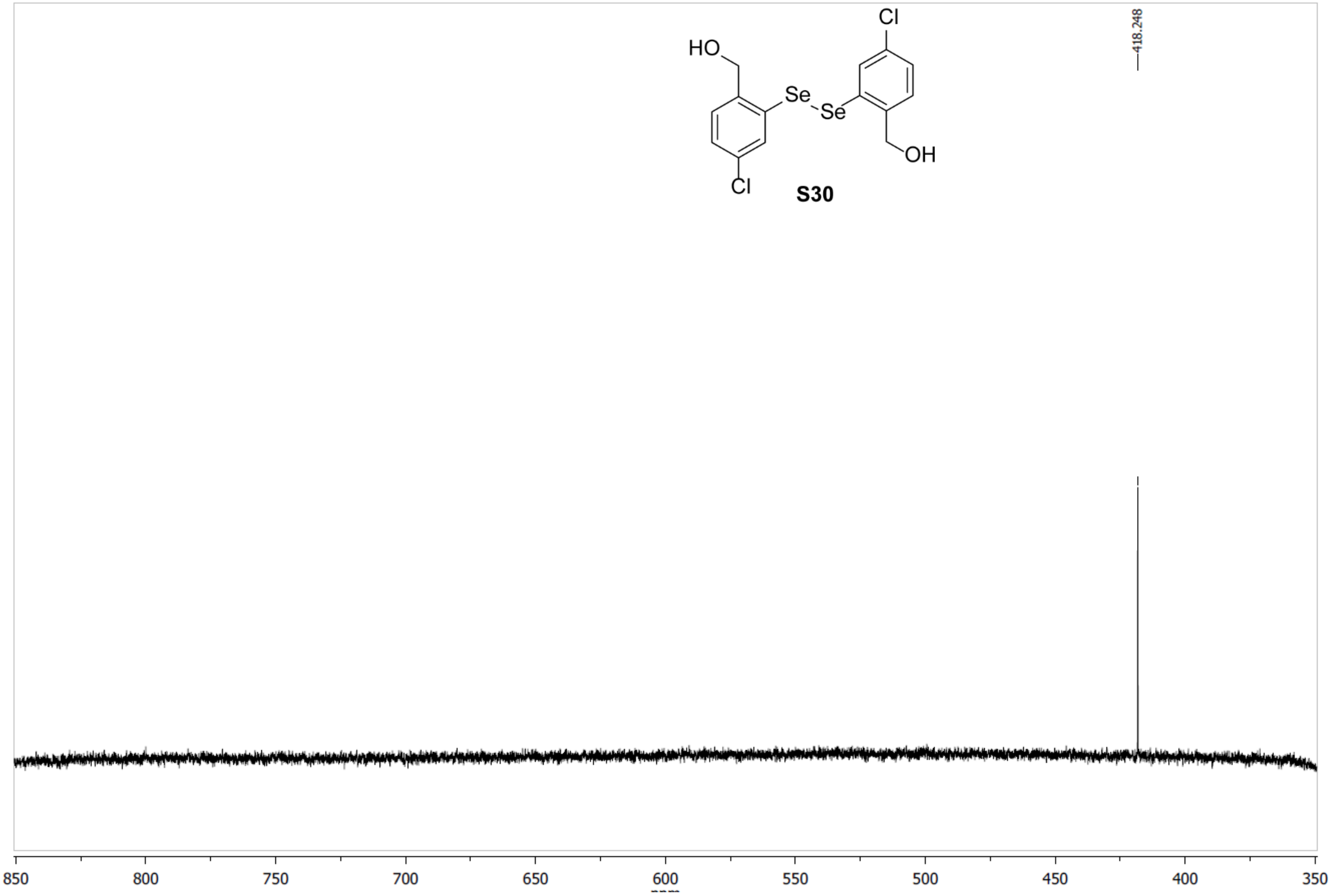

^{1}H NMR in $CDCl_3$

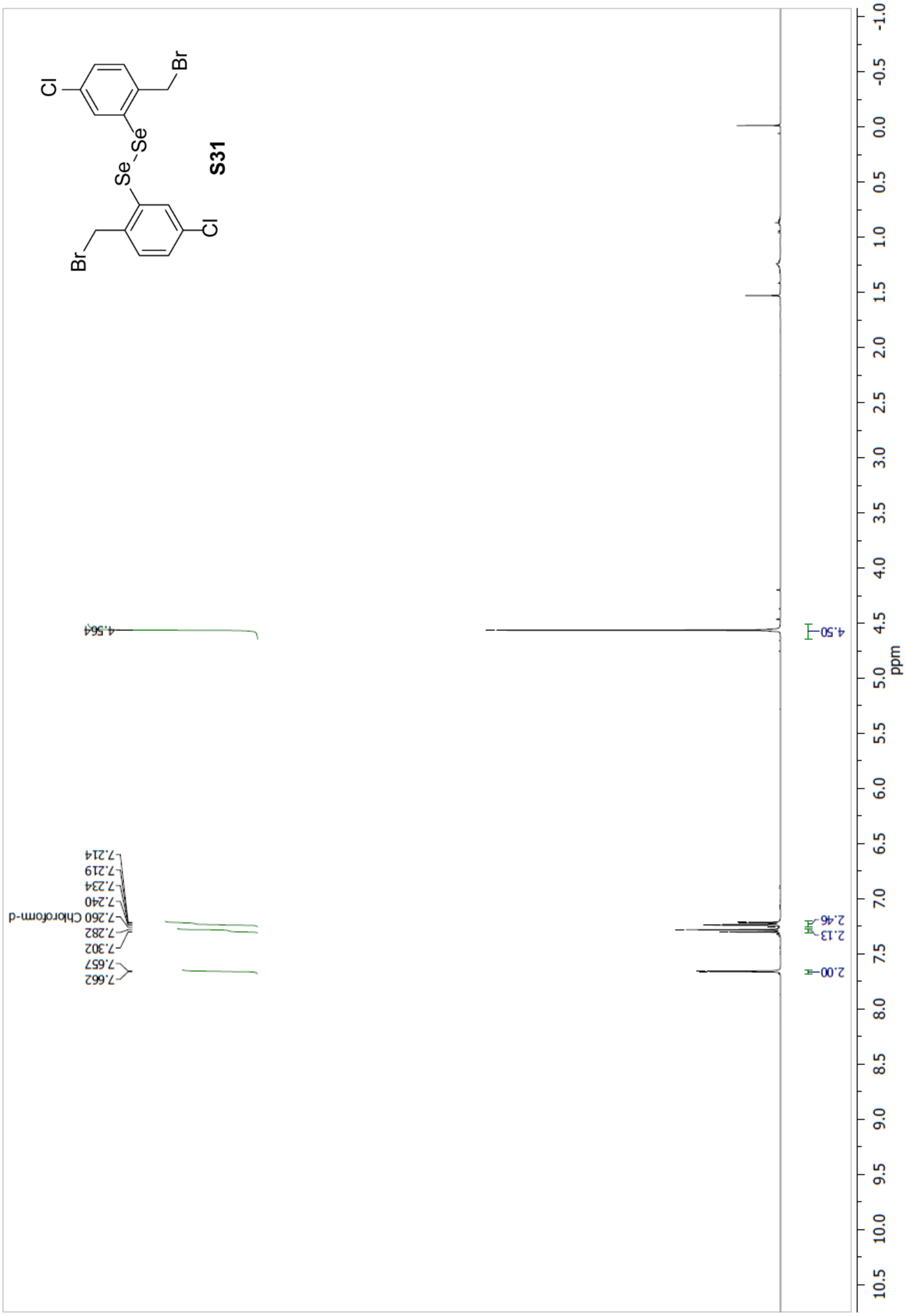

^{13}C NMR in $CDCl_3$

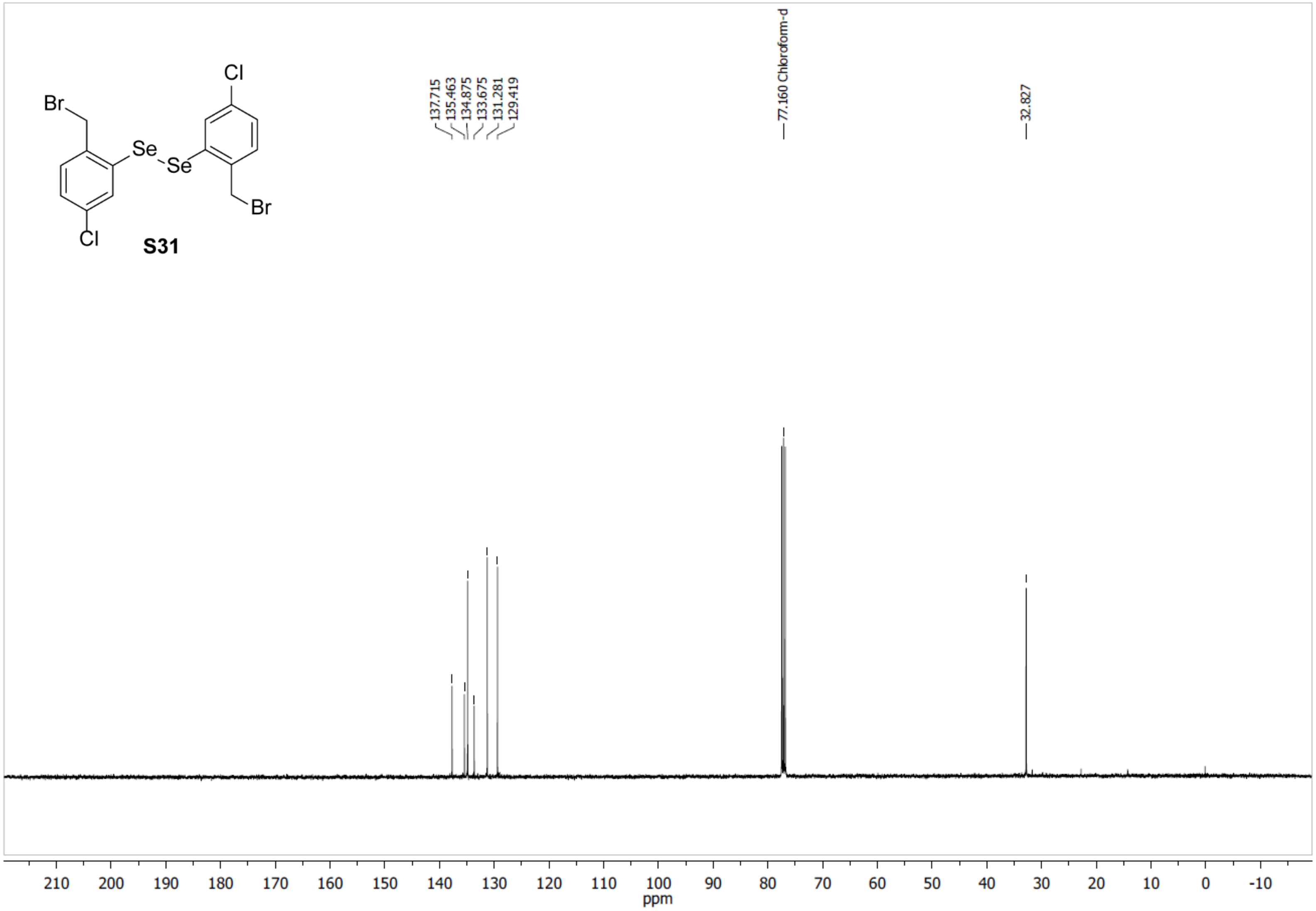

S31

^{77}Se NMR in $CDCl_3$

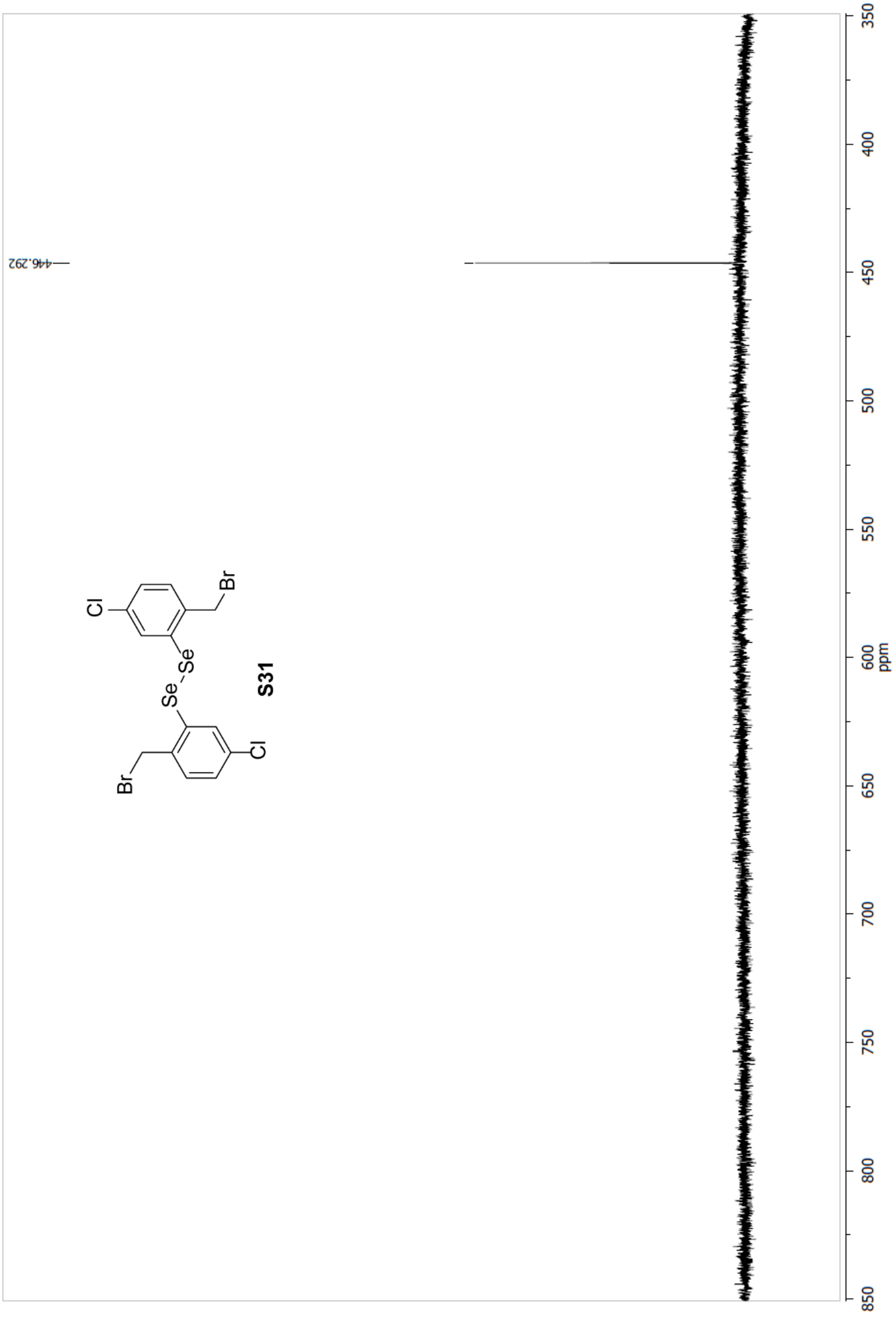

^{1}H NMR in $CDCl_3$

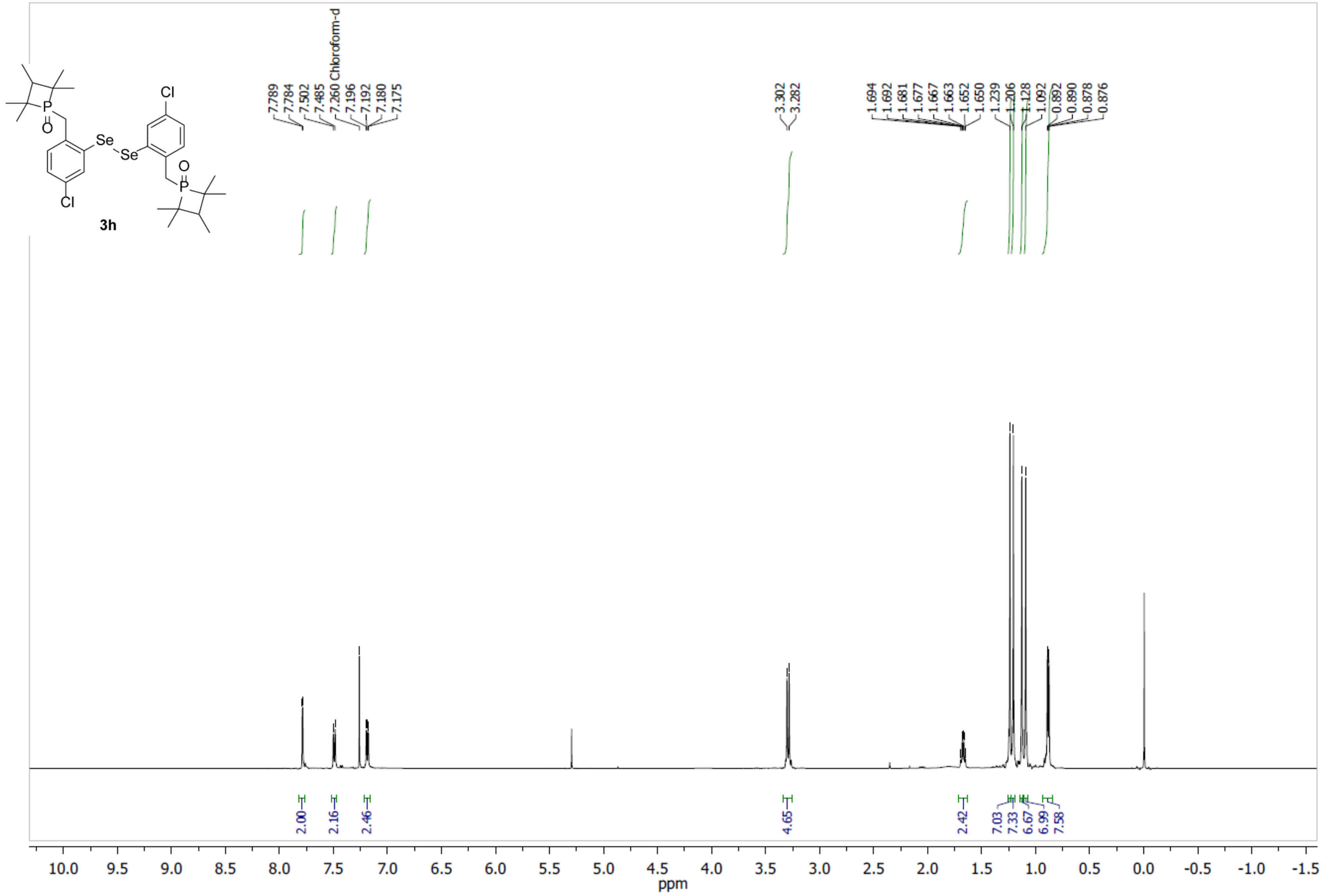

^{13}C NMR in $CDCl_3$

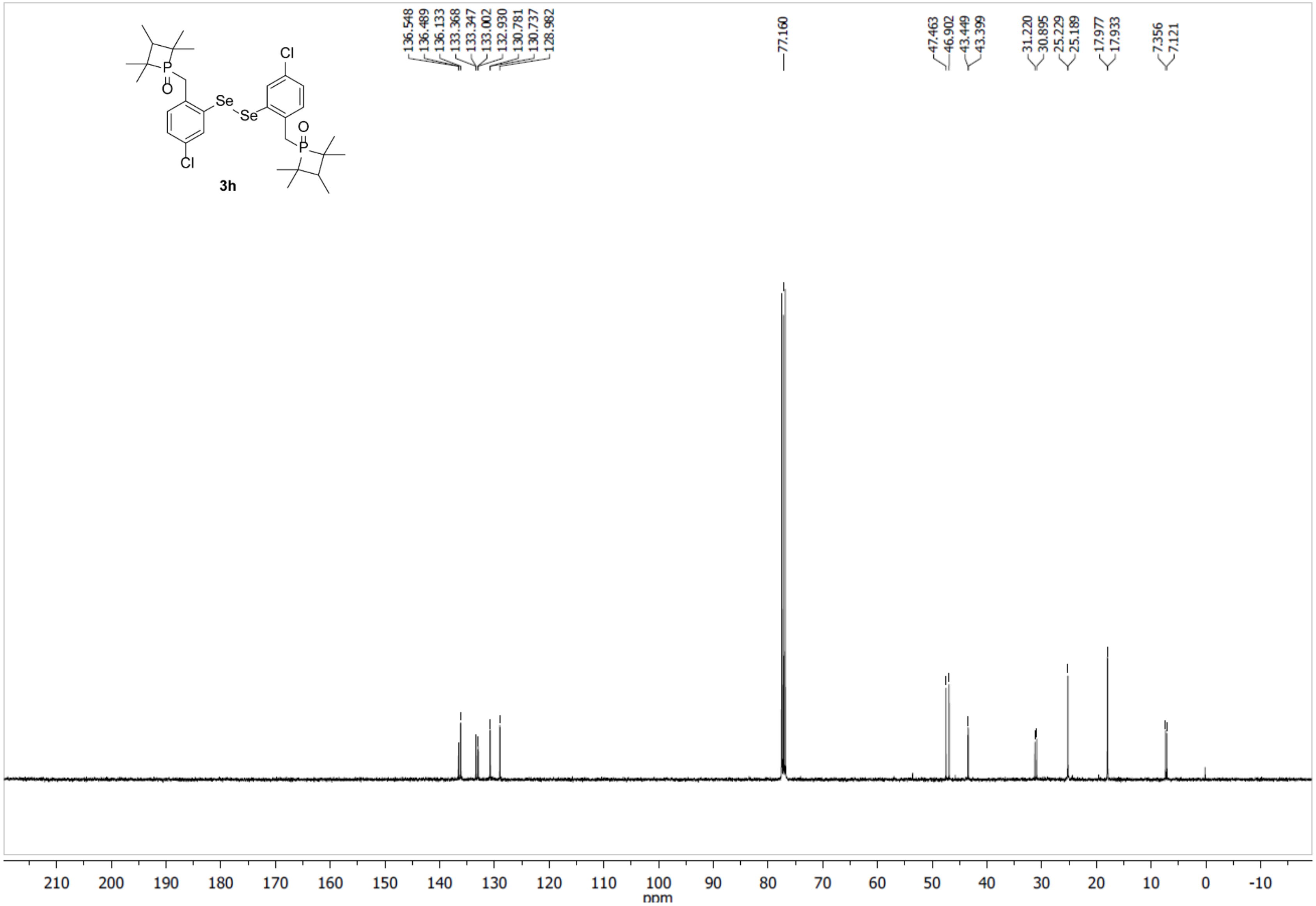

^{31}P NMR in $CDCl_3$

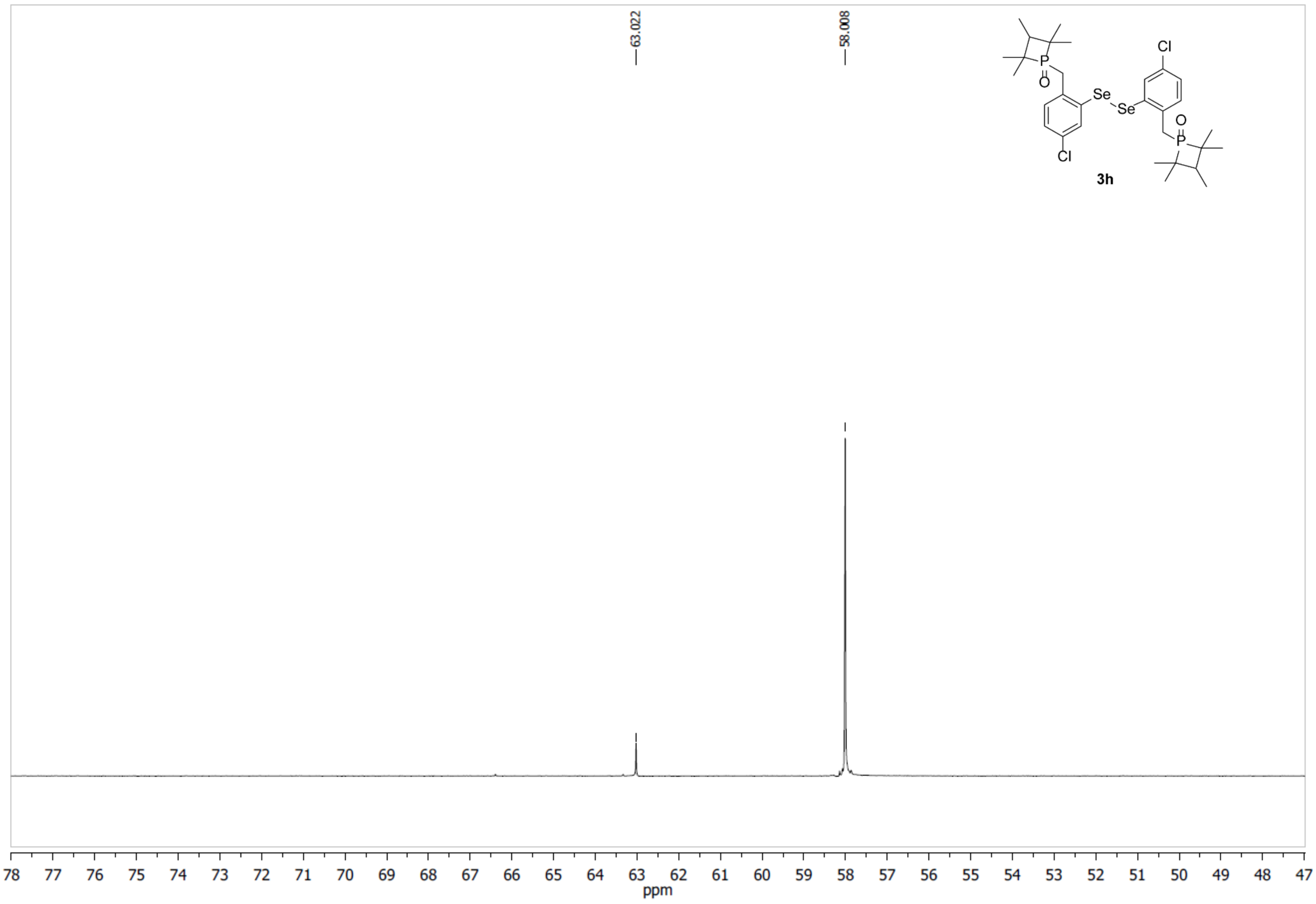

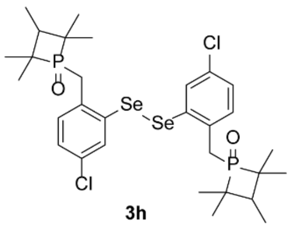

3h

^{77}Se NMR in CDCl$_3$

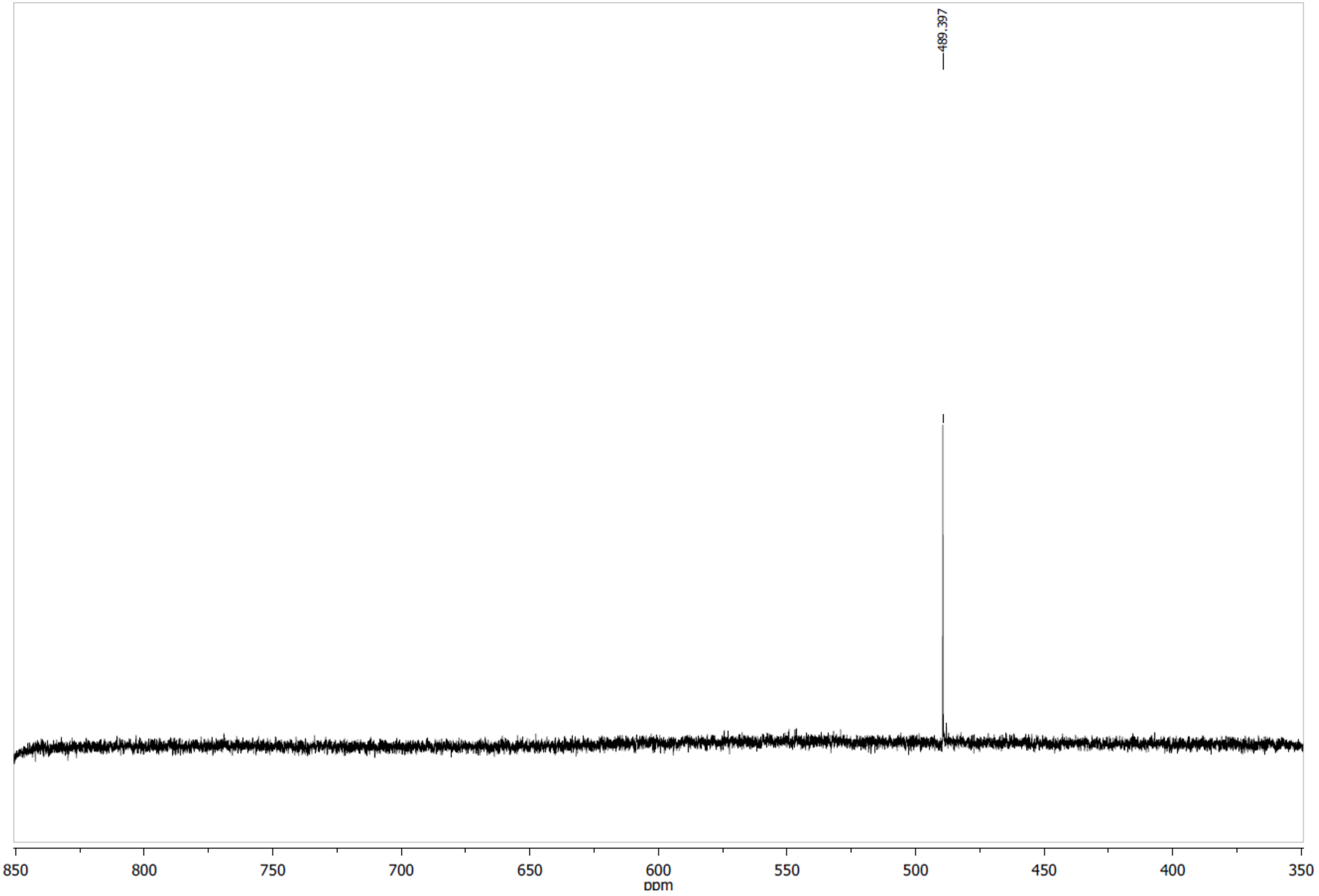

^1H NMR in $CDCl_3$

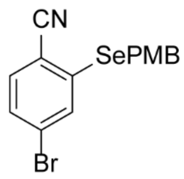

S32

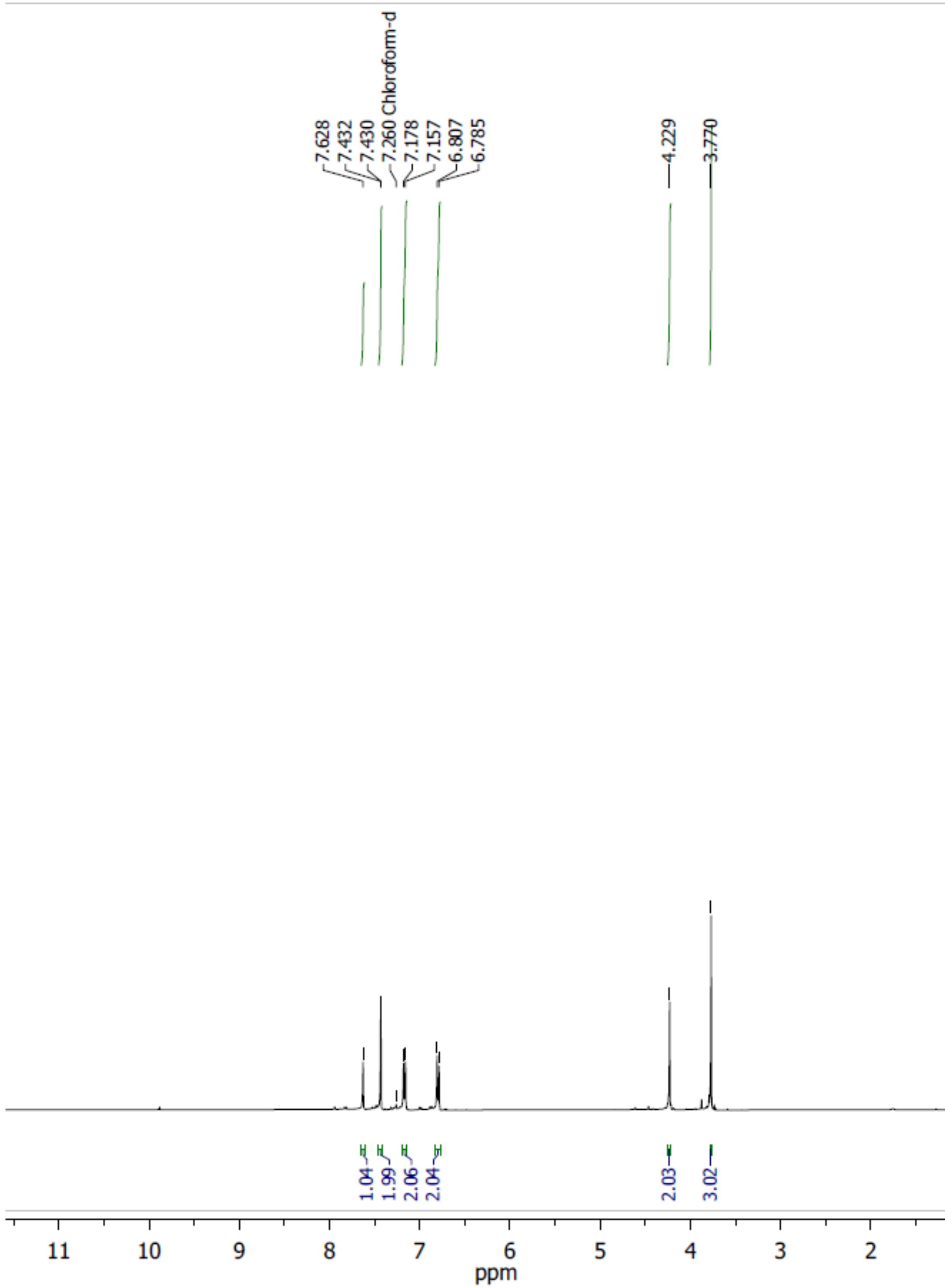

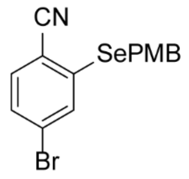

S32

^{13}C NMR in $CDCl_3$

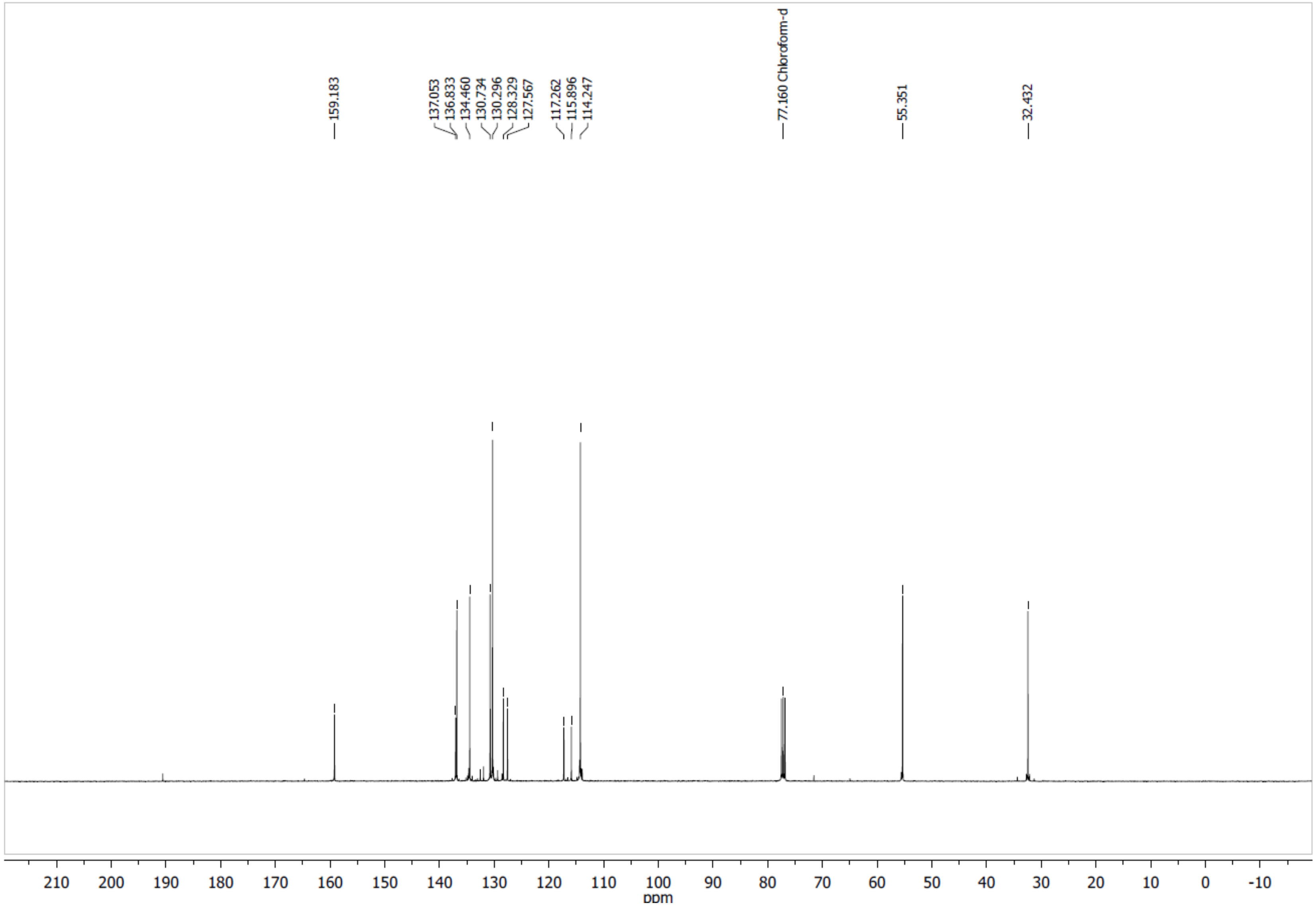

^{77}Se NMR in $CDCl_3$

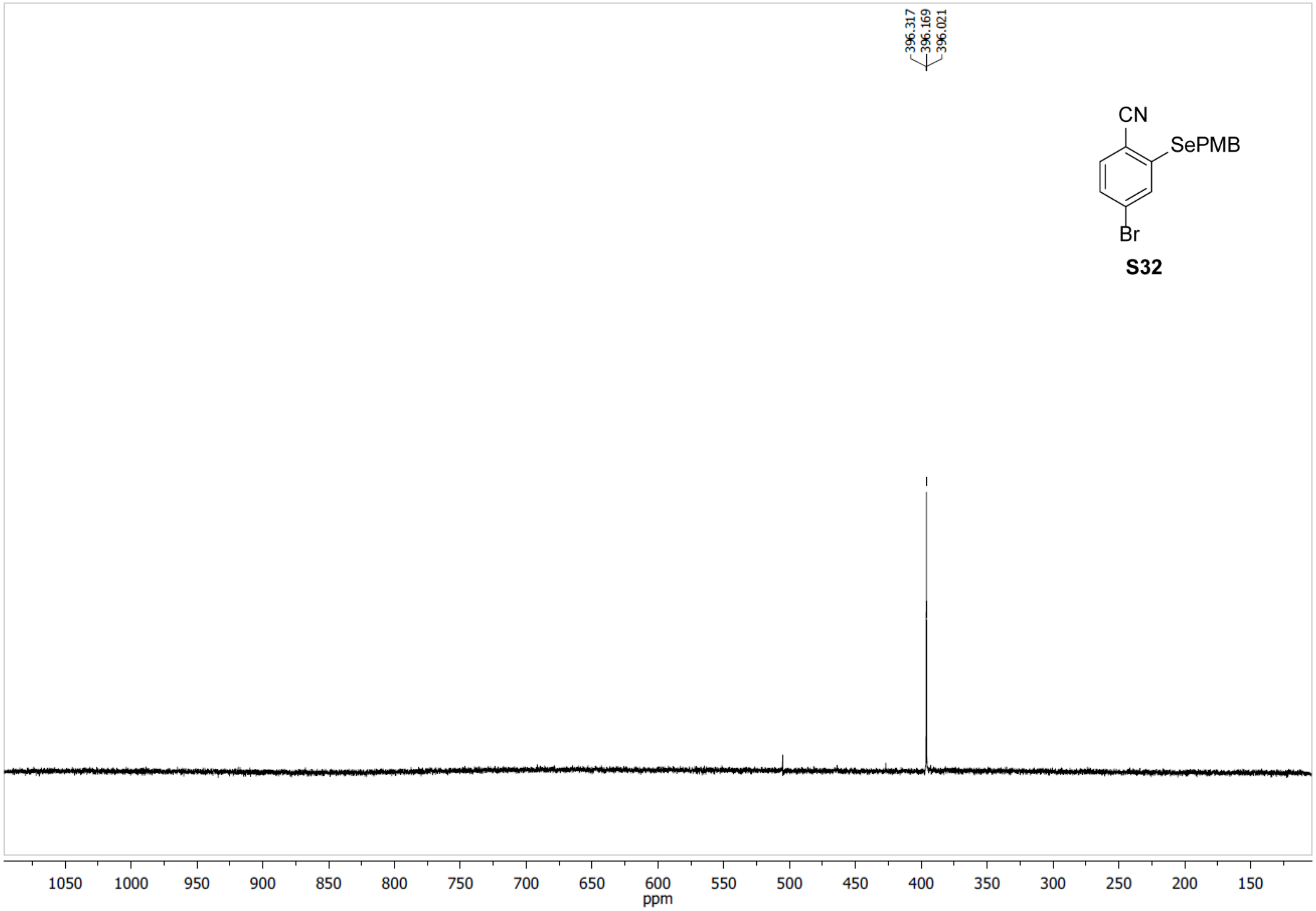

^{1}H NMR in $CDCl_3$

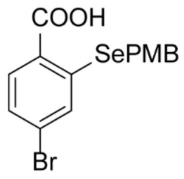

S33

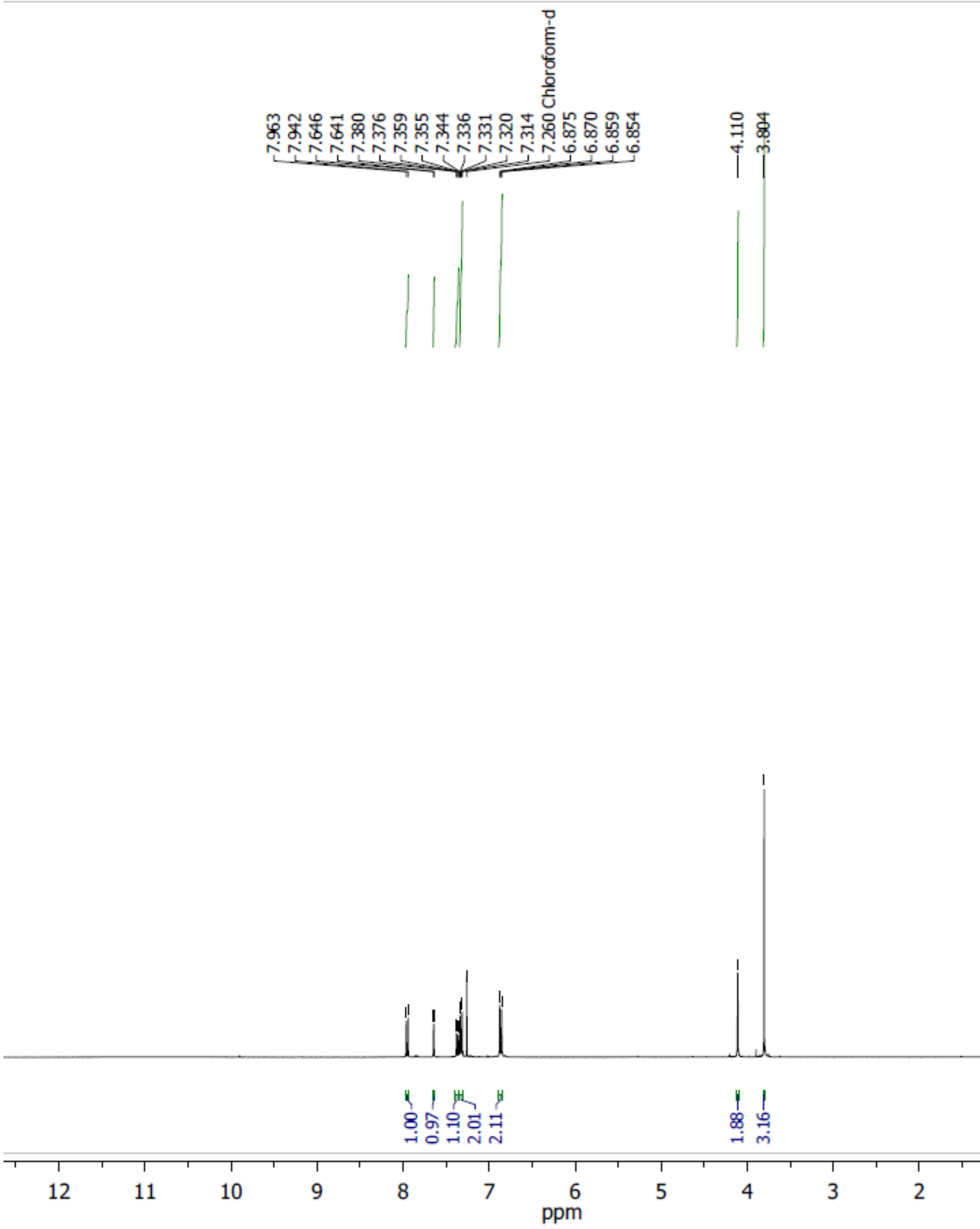

^{13}C NMR in $CDCl_3$

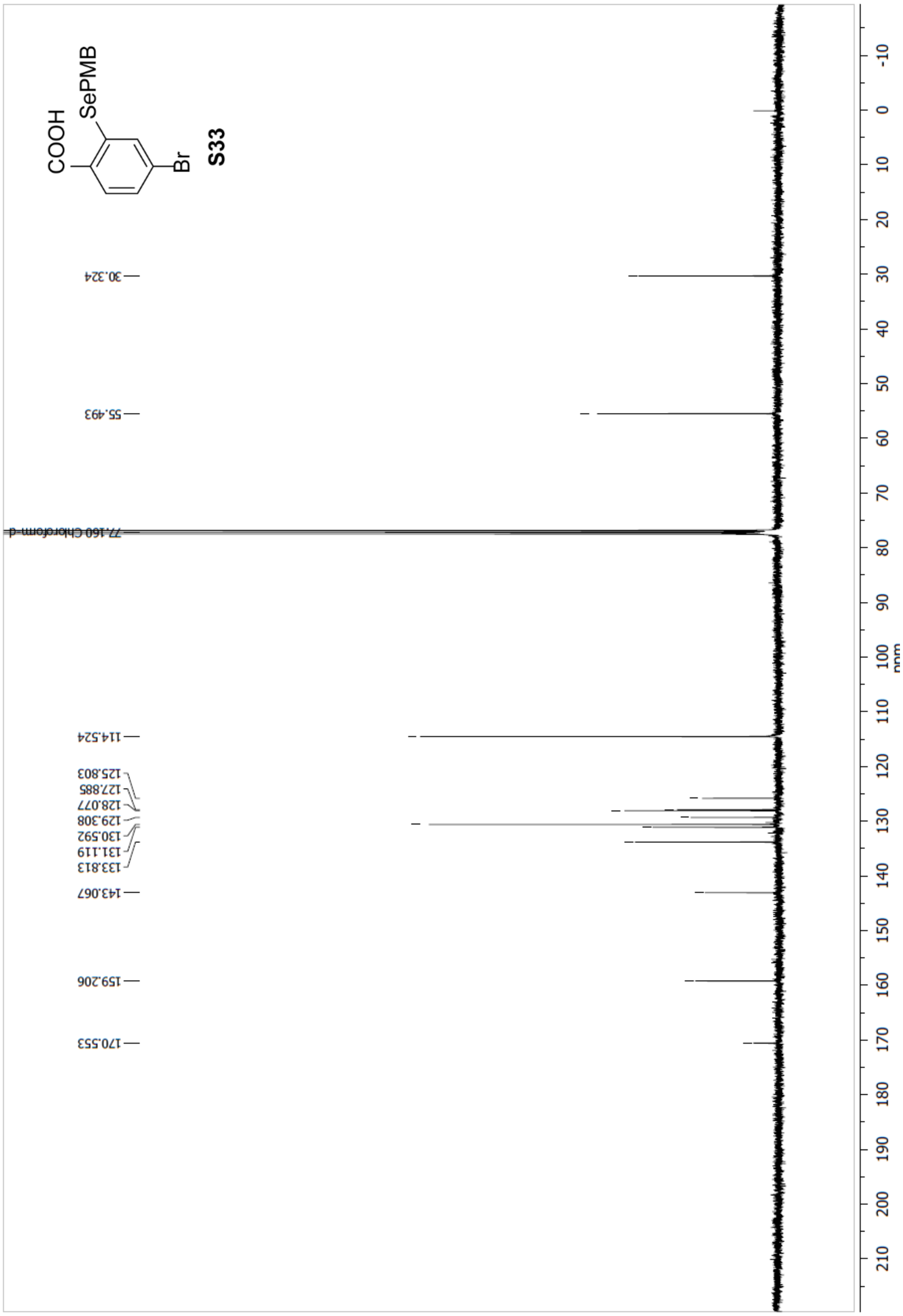

^{77}Se NMR in $CDCl_3$

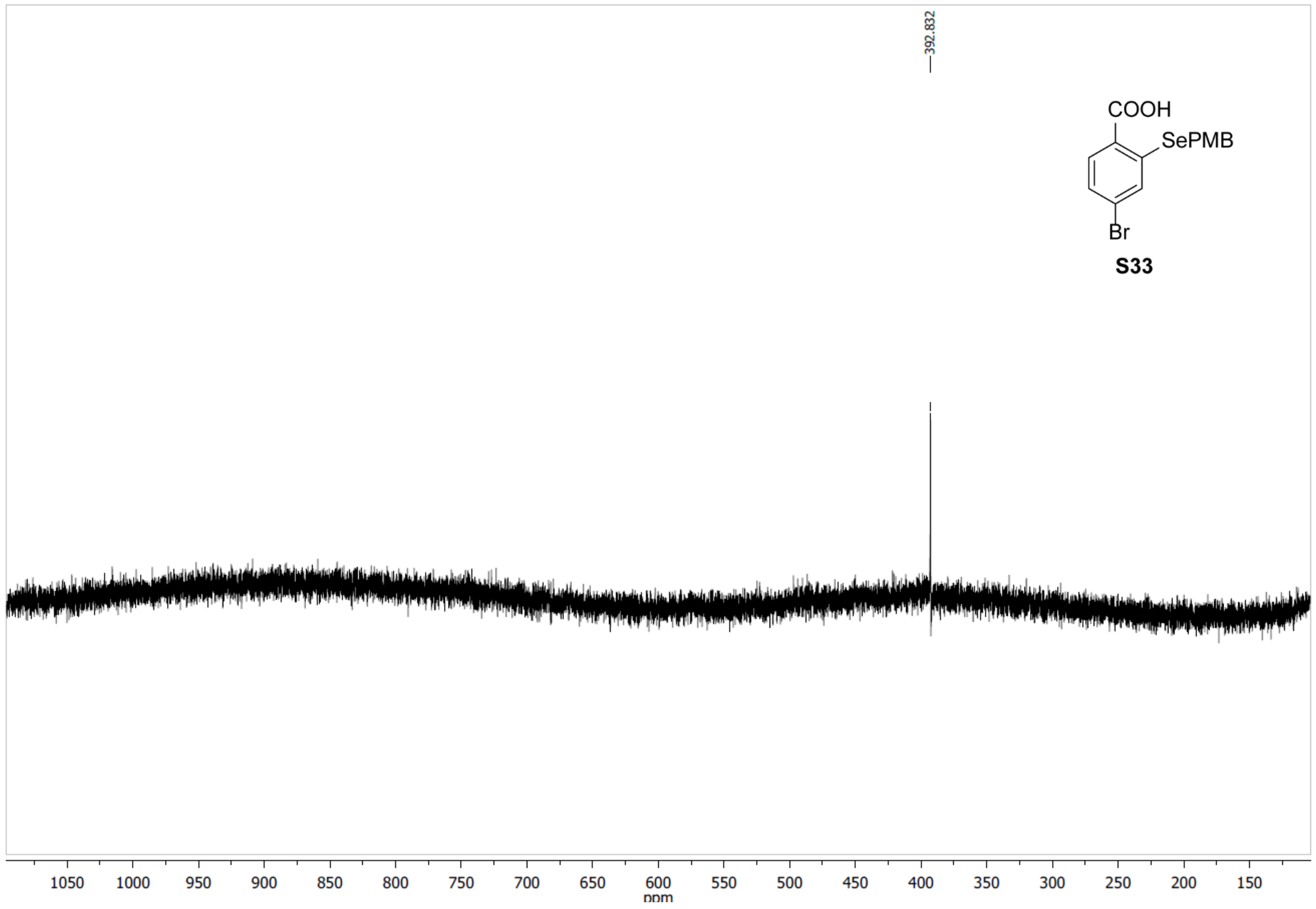

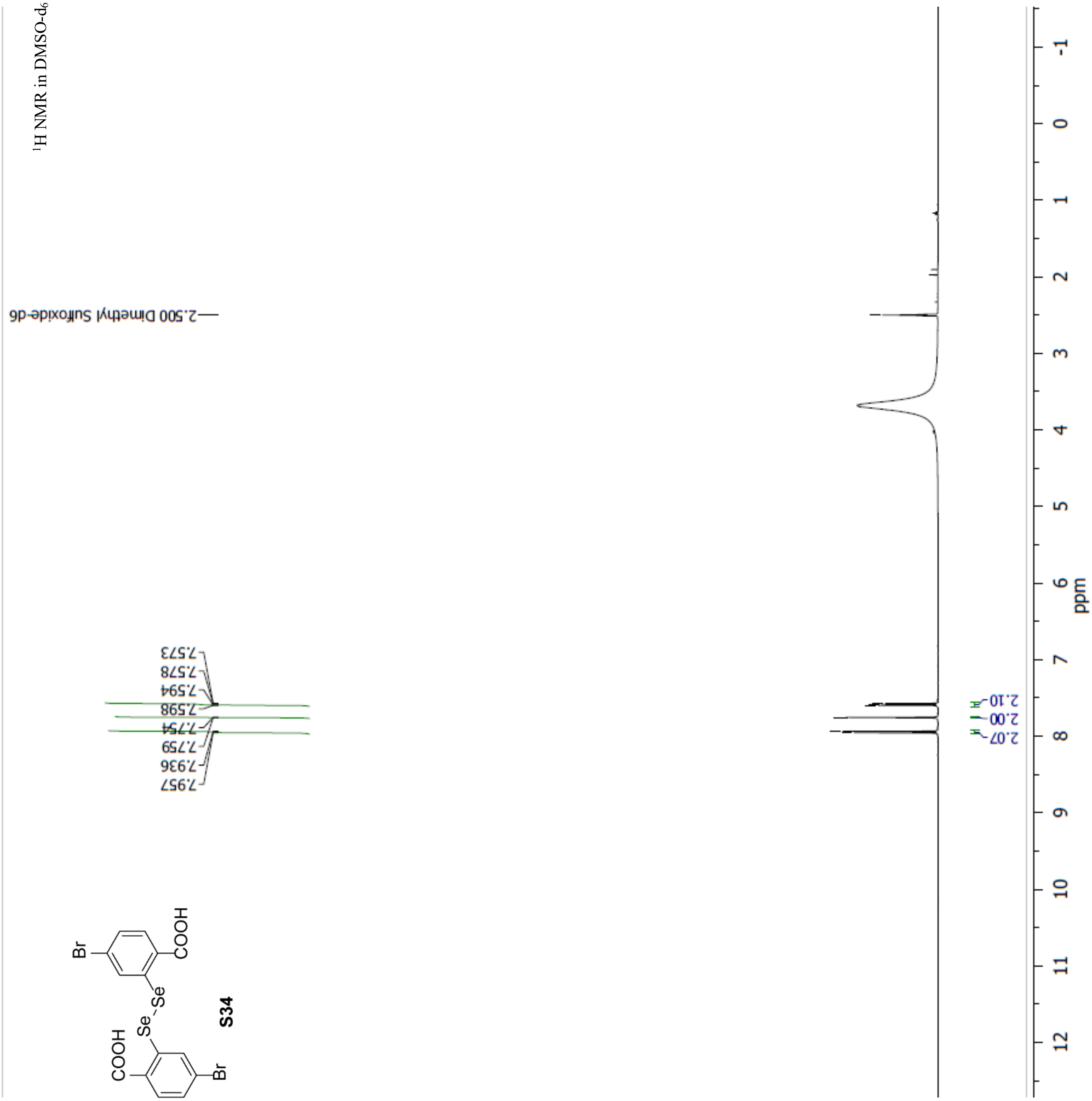

1H NMR in DMSO-d6
2.500 Dimethyl Sulfoxide-d6
7.573
7.578
7.594
7.598
7.754
7.759
7.936
7.957
2.10
2.00
2.07
ppm
S34

^{13}C NMR in DMSO-d_6

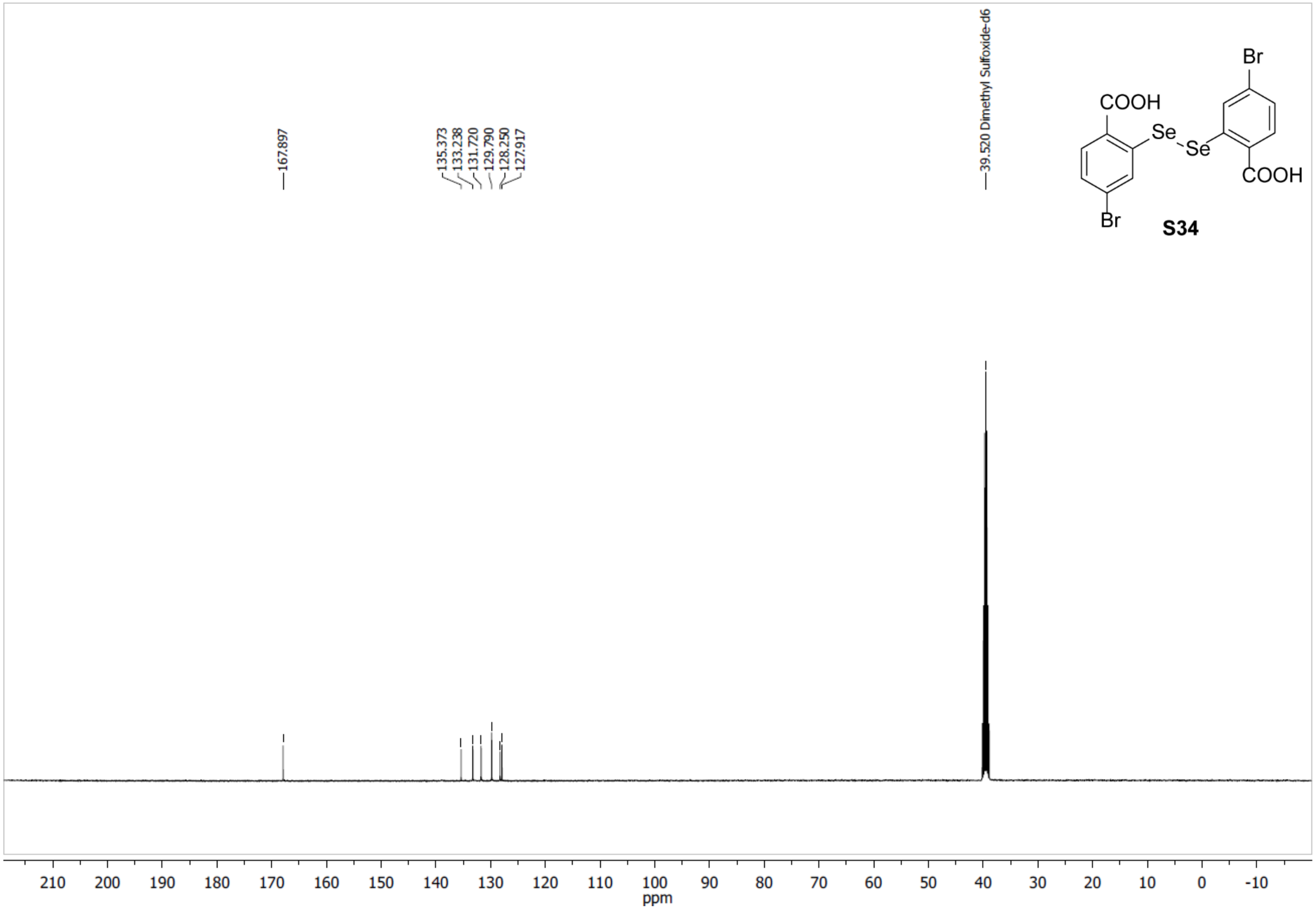

S34

^{77}Se NMR in DMSO-d_6

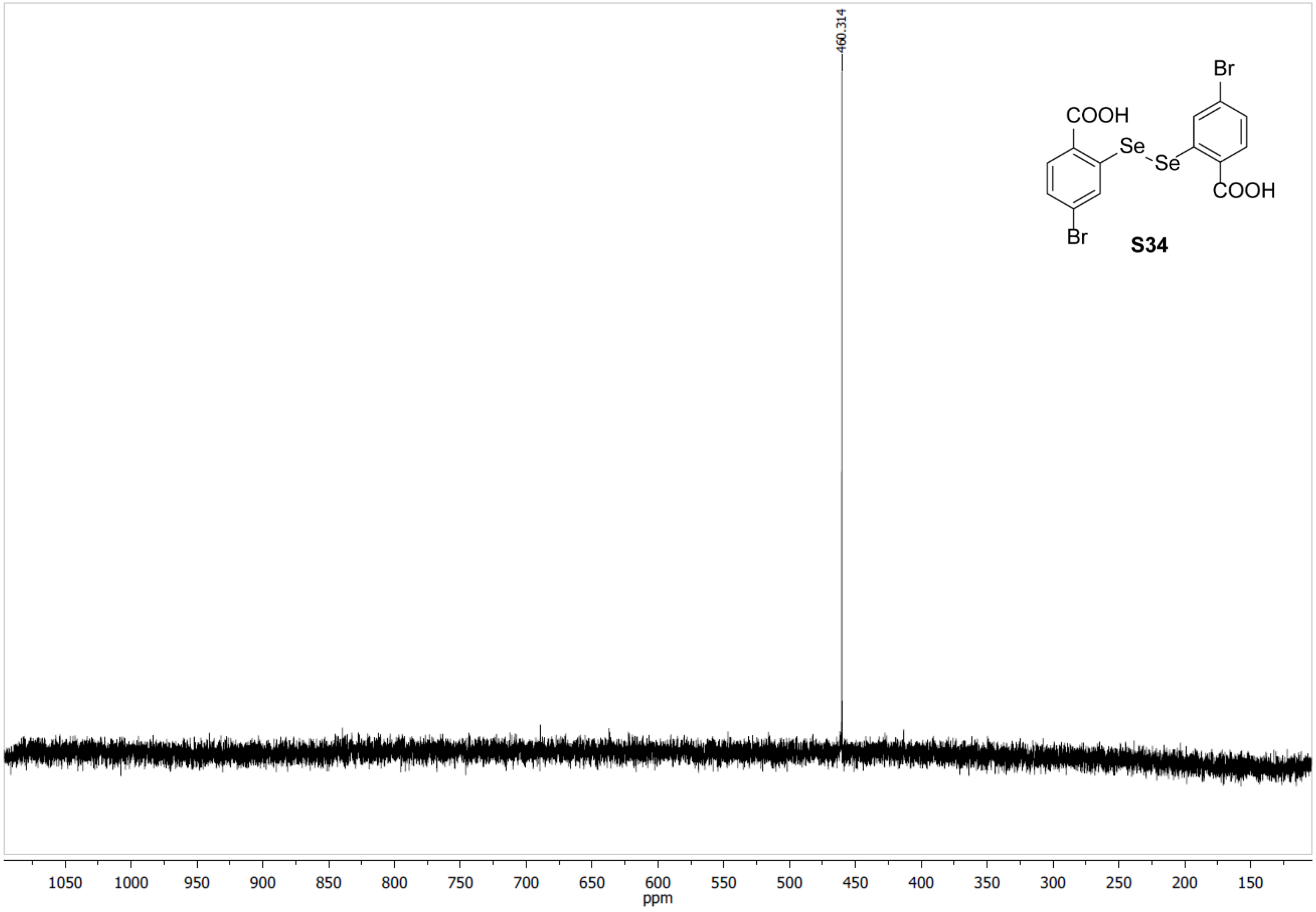

^{1}H NMR in $CDCl_3$

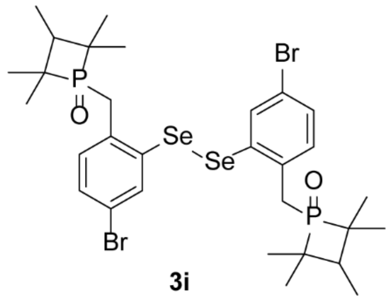

3i

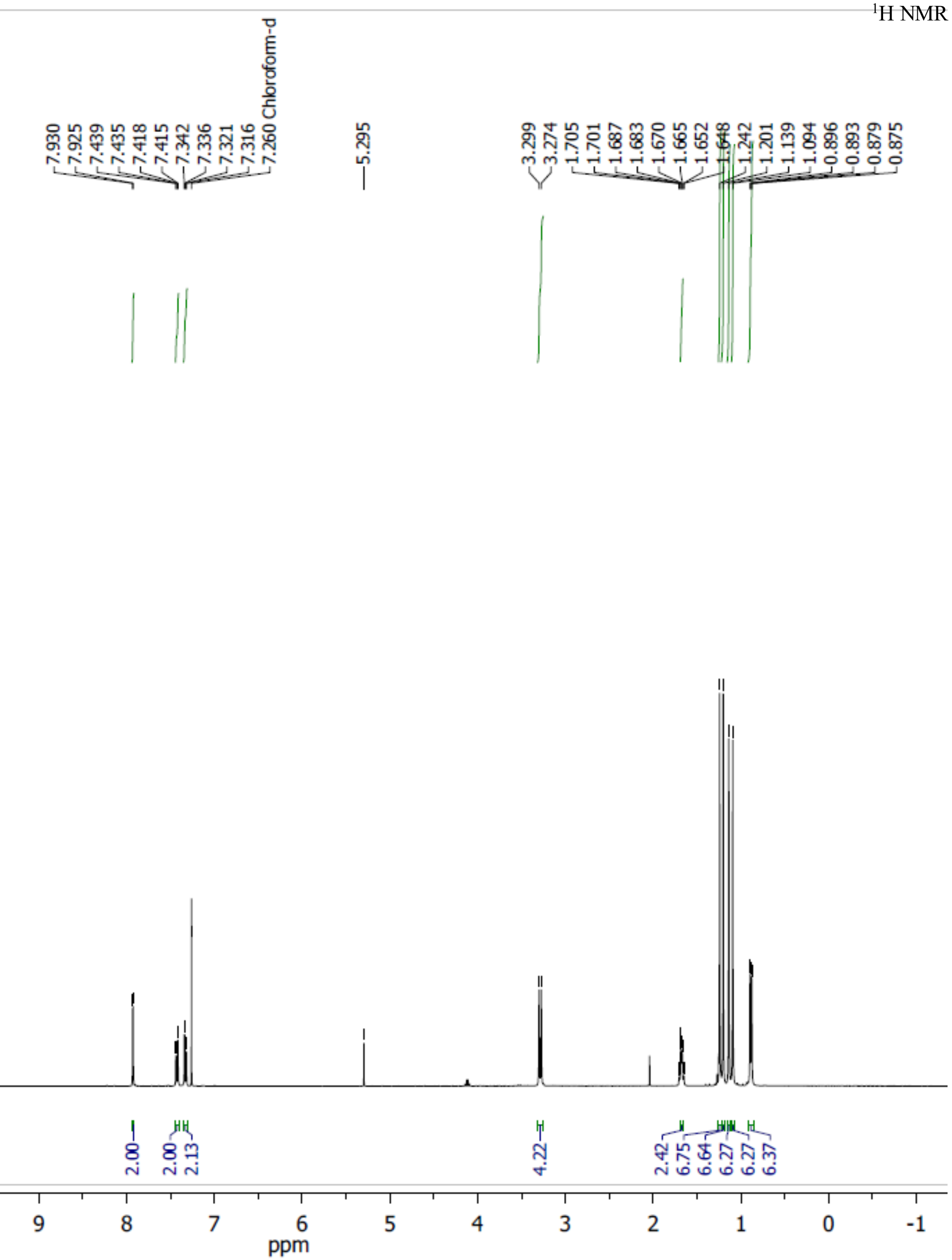

^{13}C NMR in $CDCl_3$

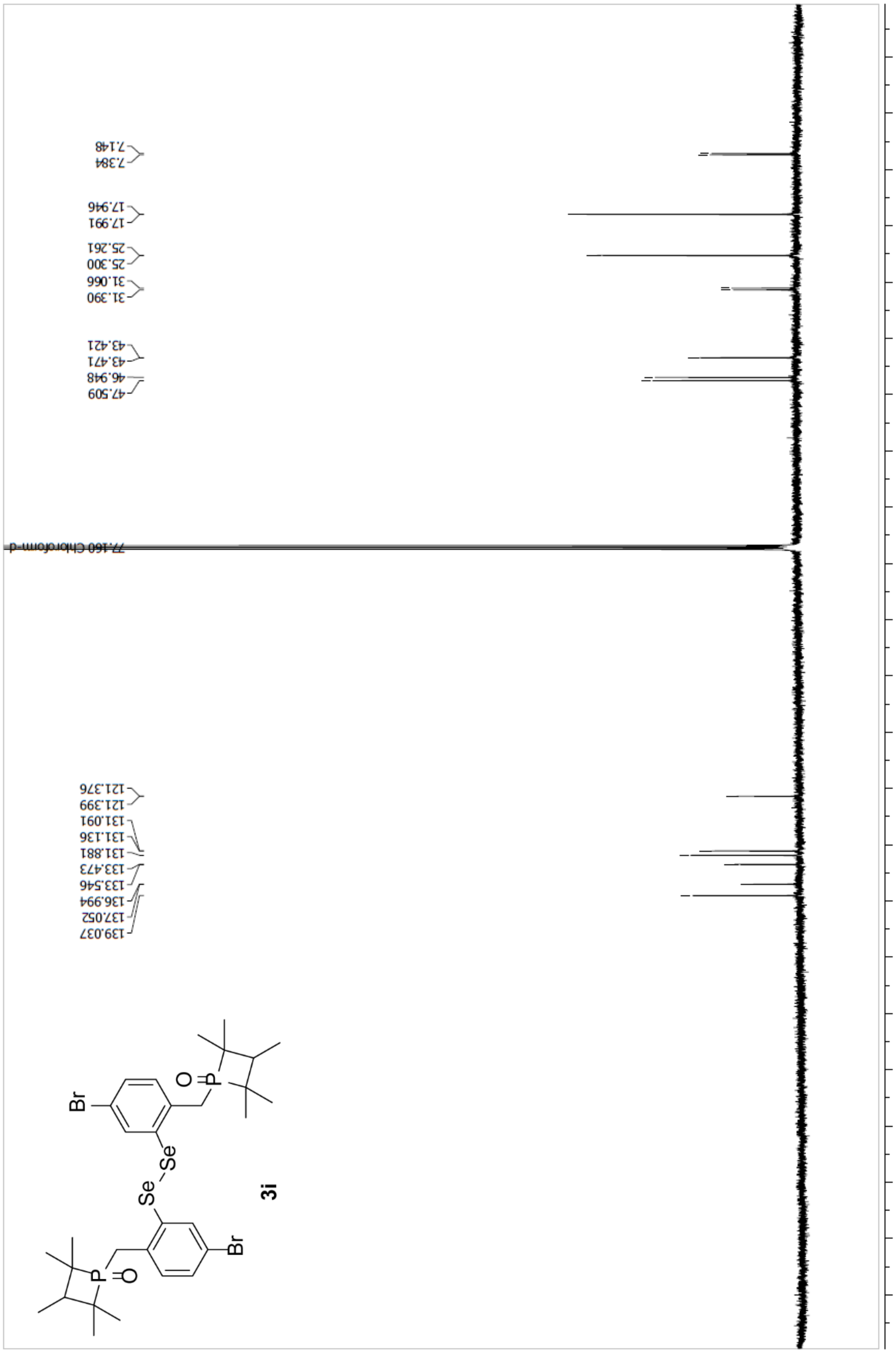

^{31}P NMR in $CDCl_3$

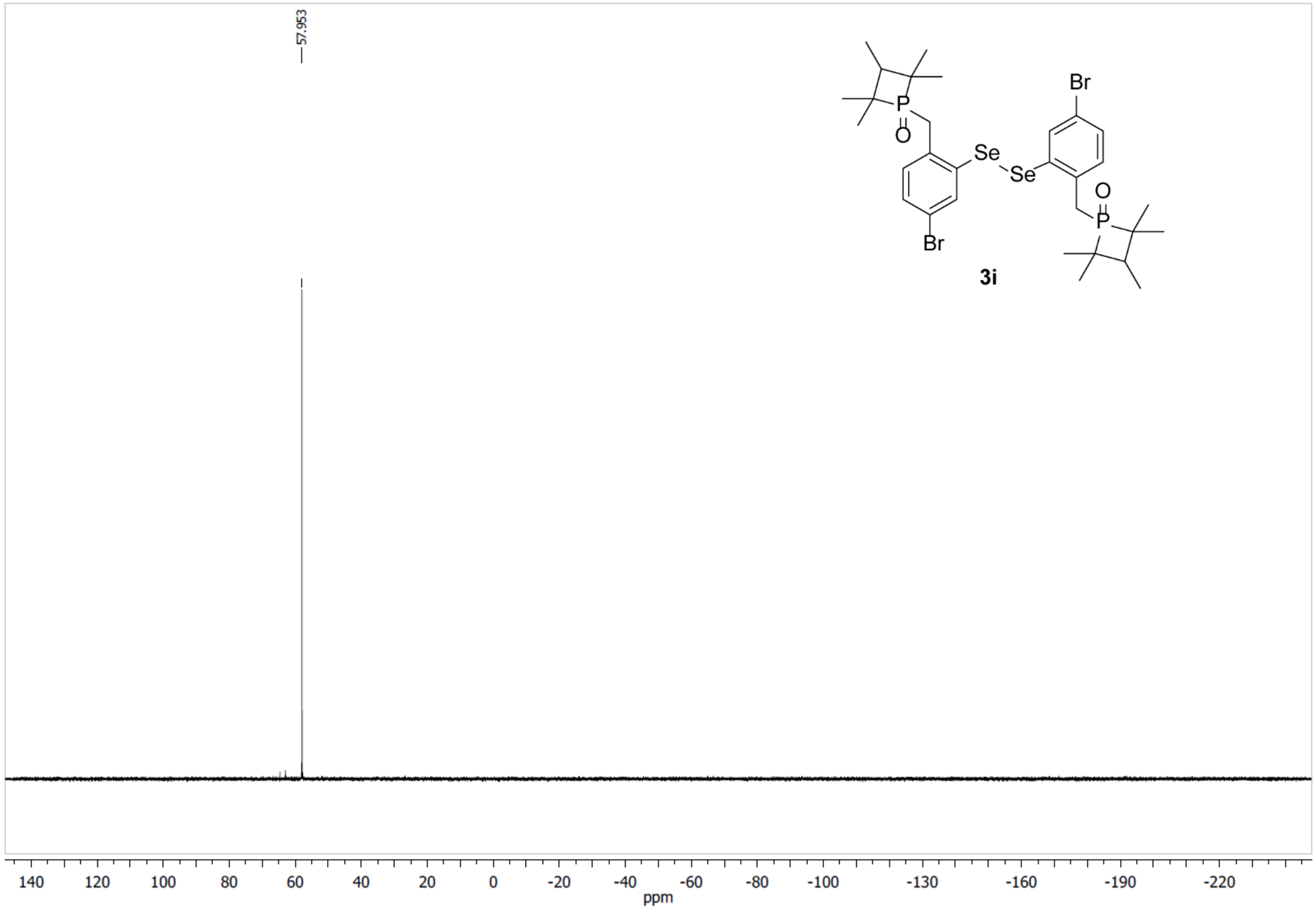

^{77}Se NMR in $CDCl_3$

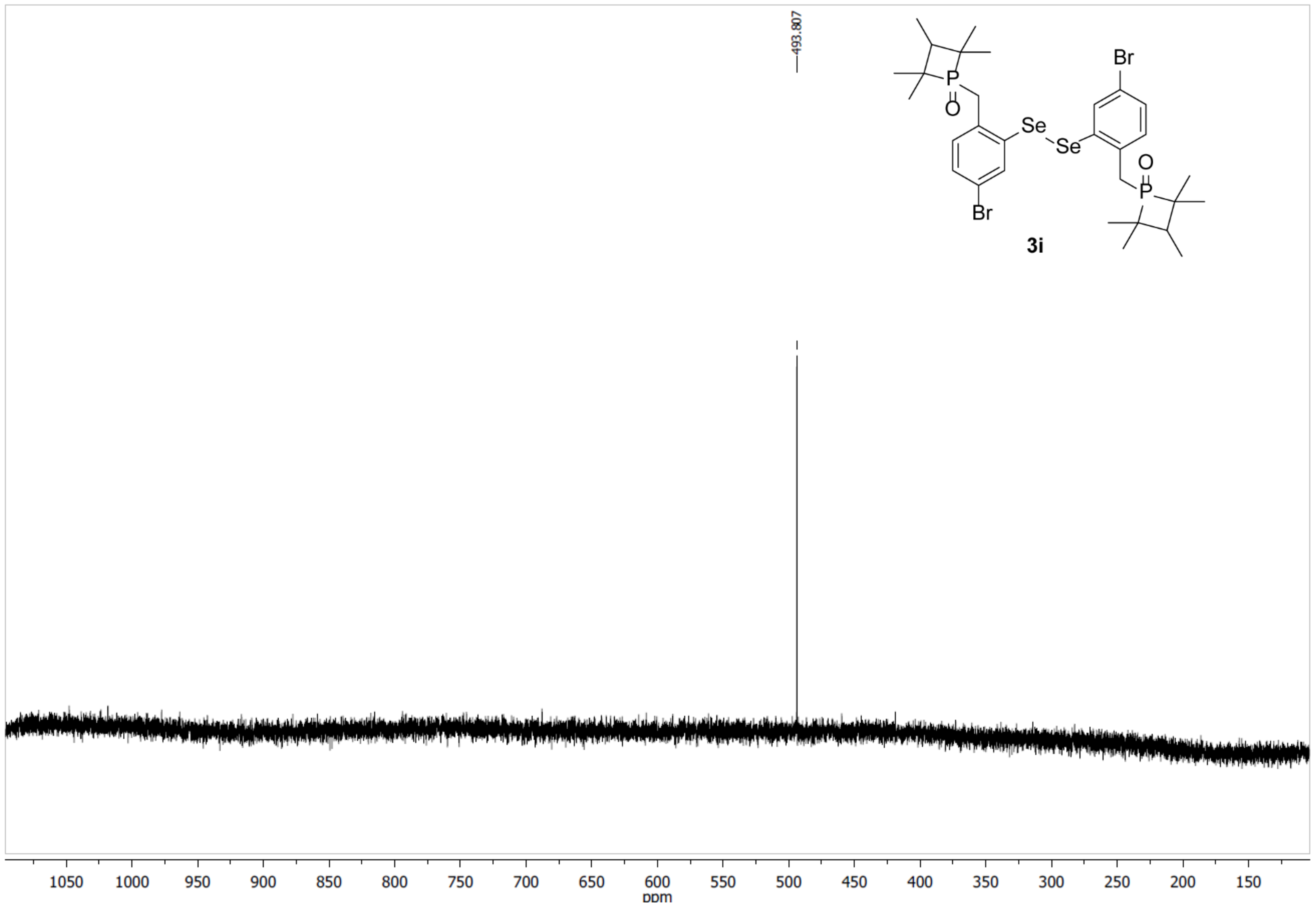

^{1}H NMR in $CDCl_3$

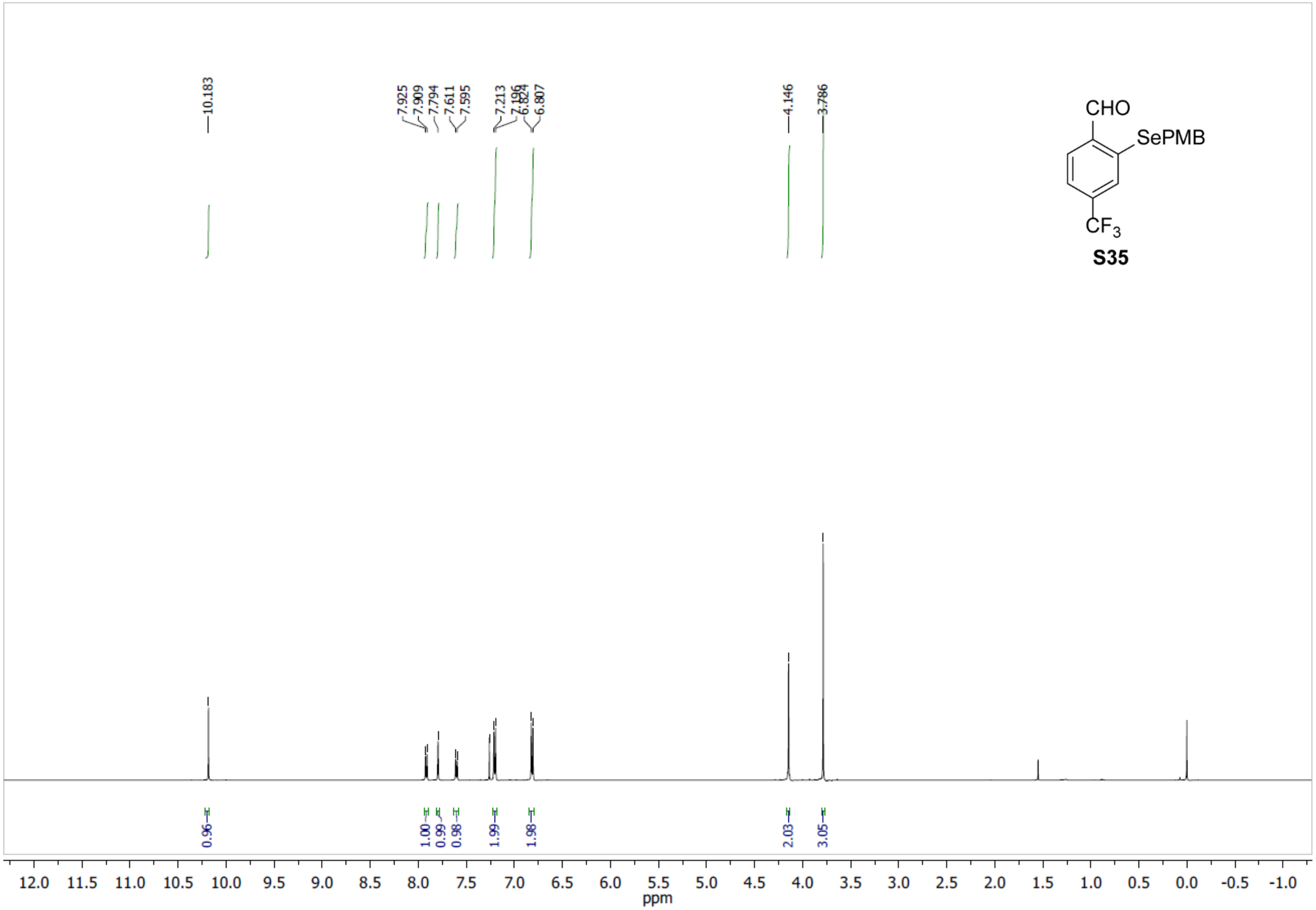

S35

^{13}C NMR in $CDCl_3$

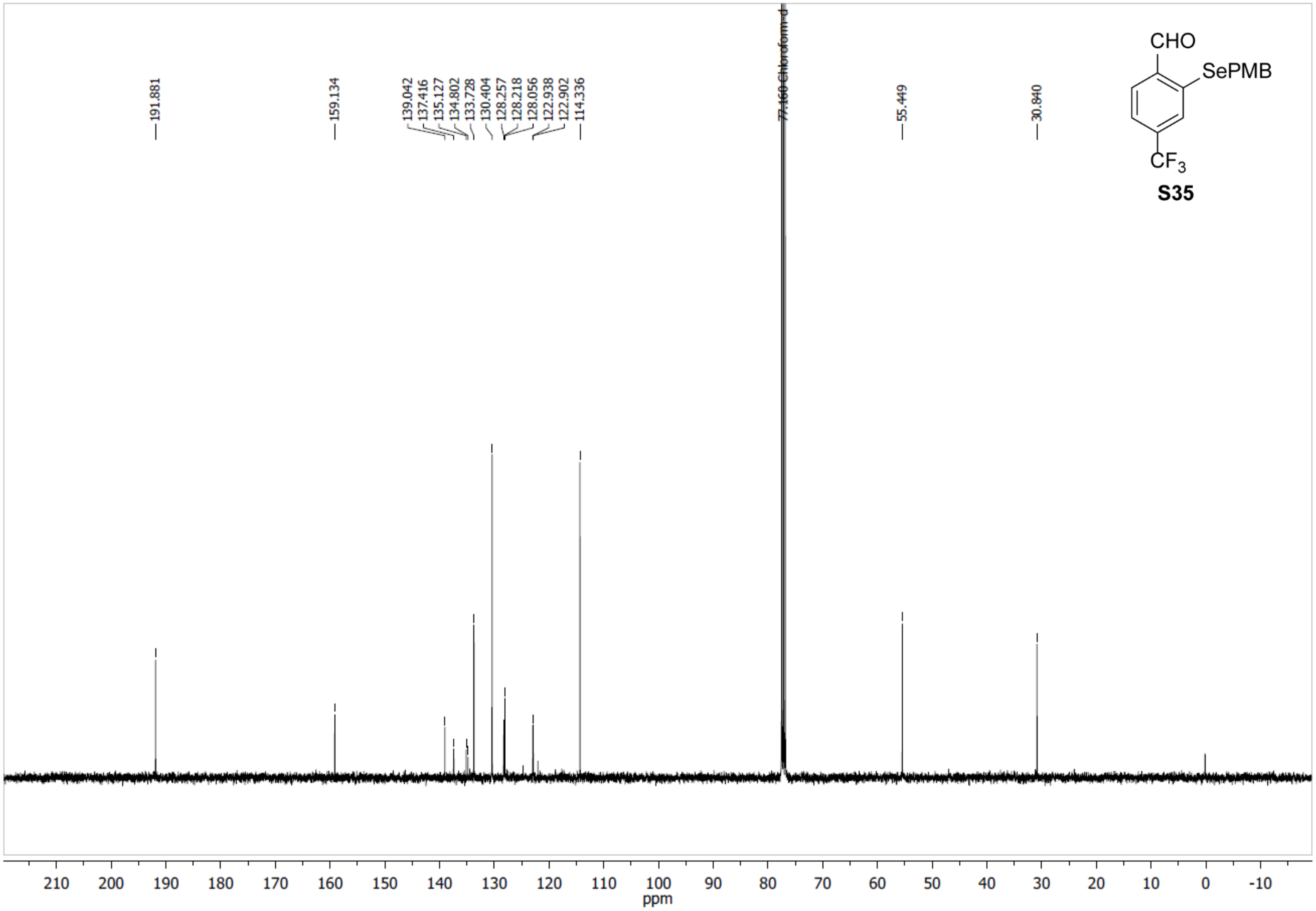

^{19}F NMR in $CDCl_3$

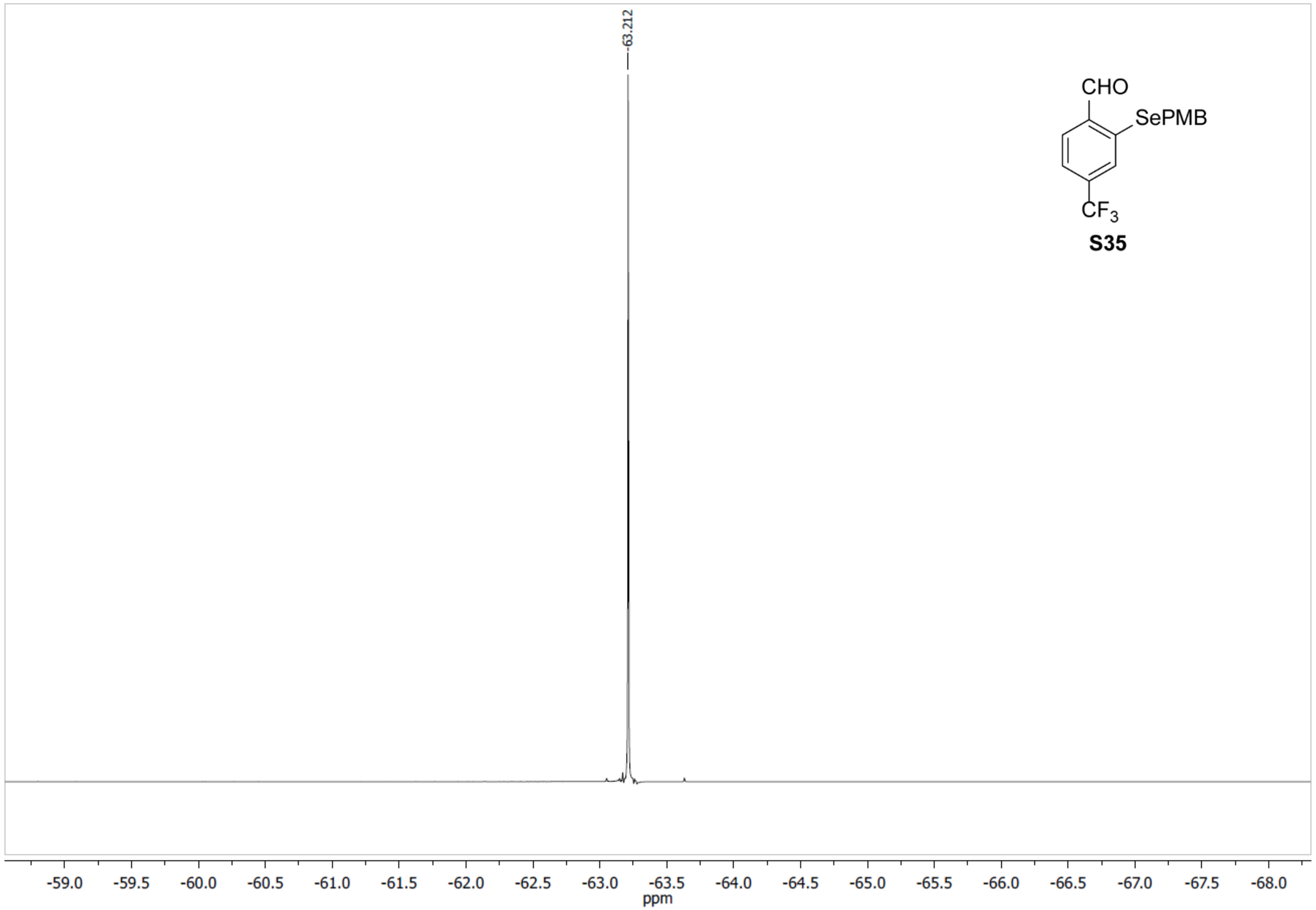

^{77}Se NMR in $CDCl_3$

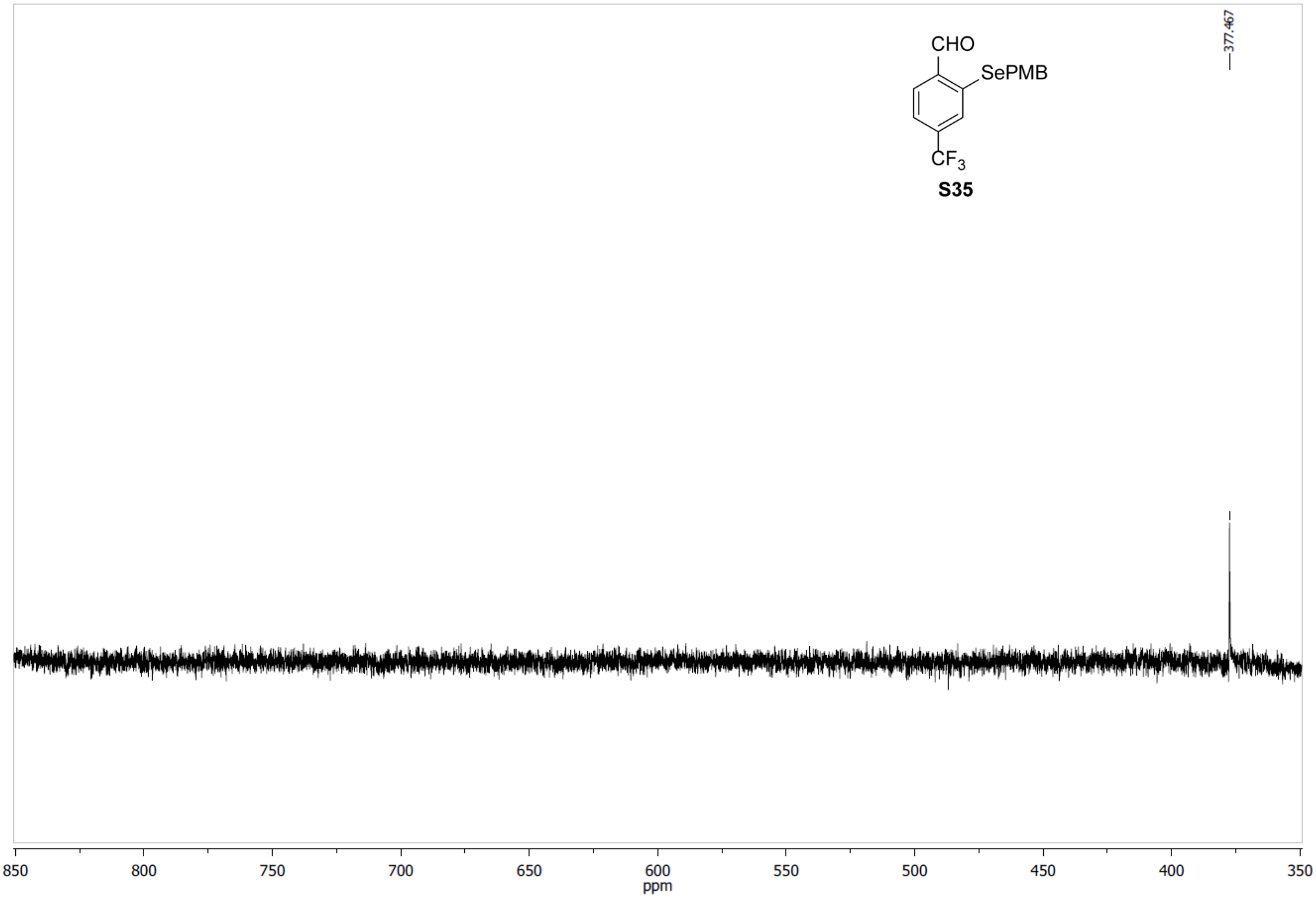

^{1}H NMR in $CDCl_3$

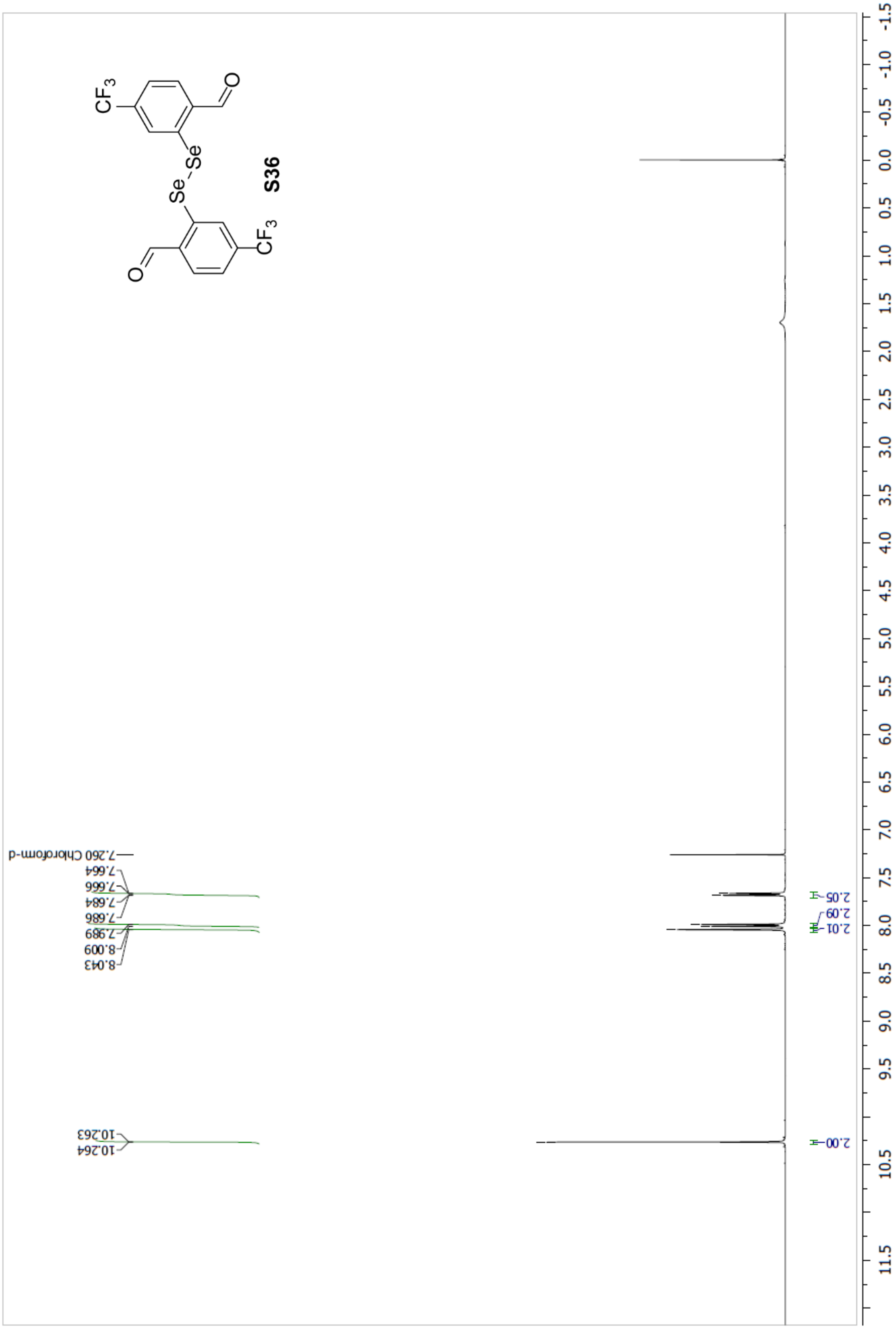

^{13}C NMR in $CDCl_3$

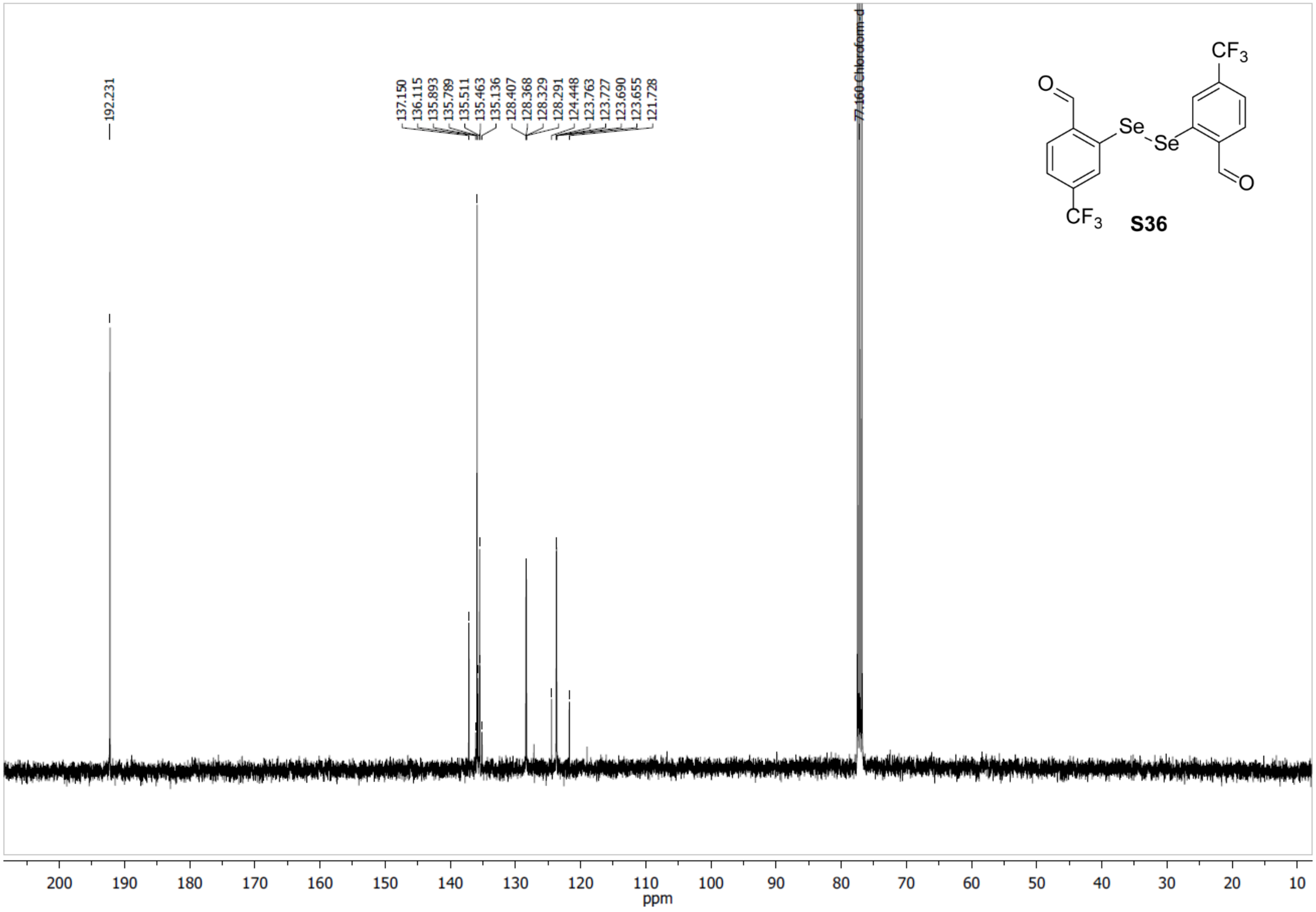

S36

^{19}F NMR in $CDCl_3$

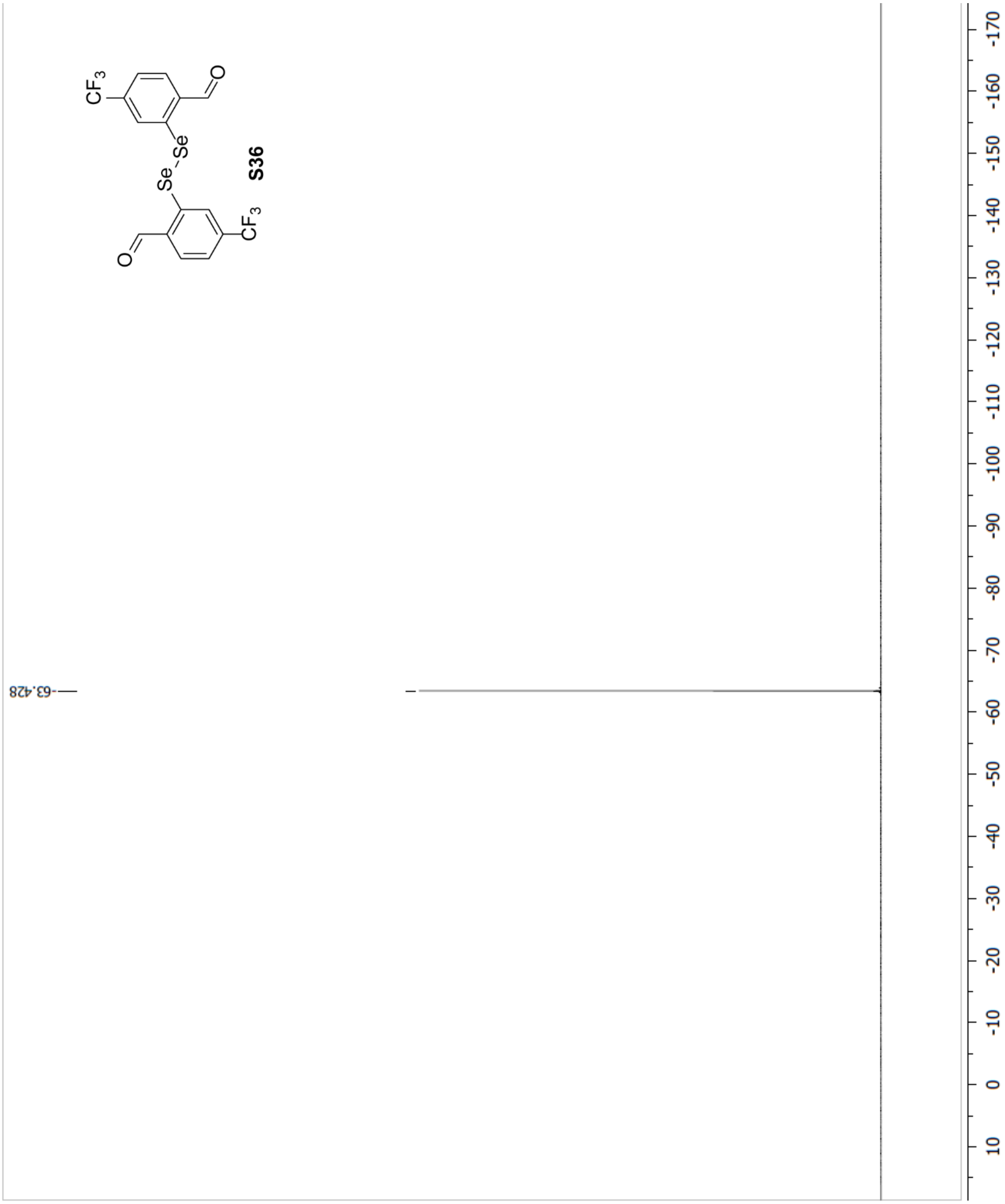

^{77}Se NMR in $CDCl_3$

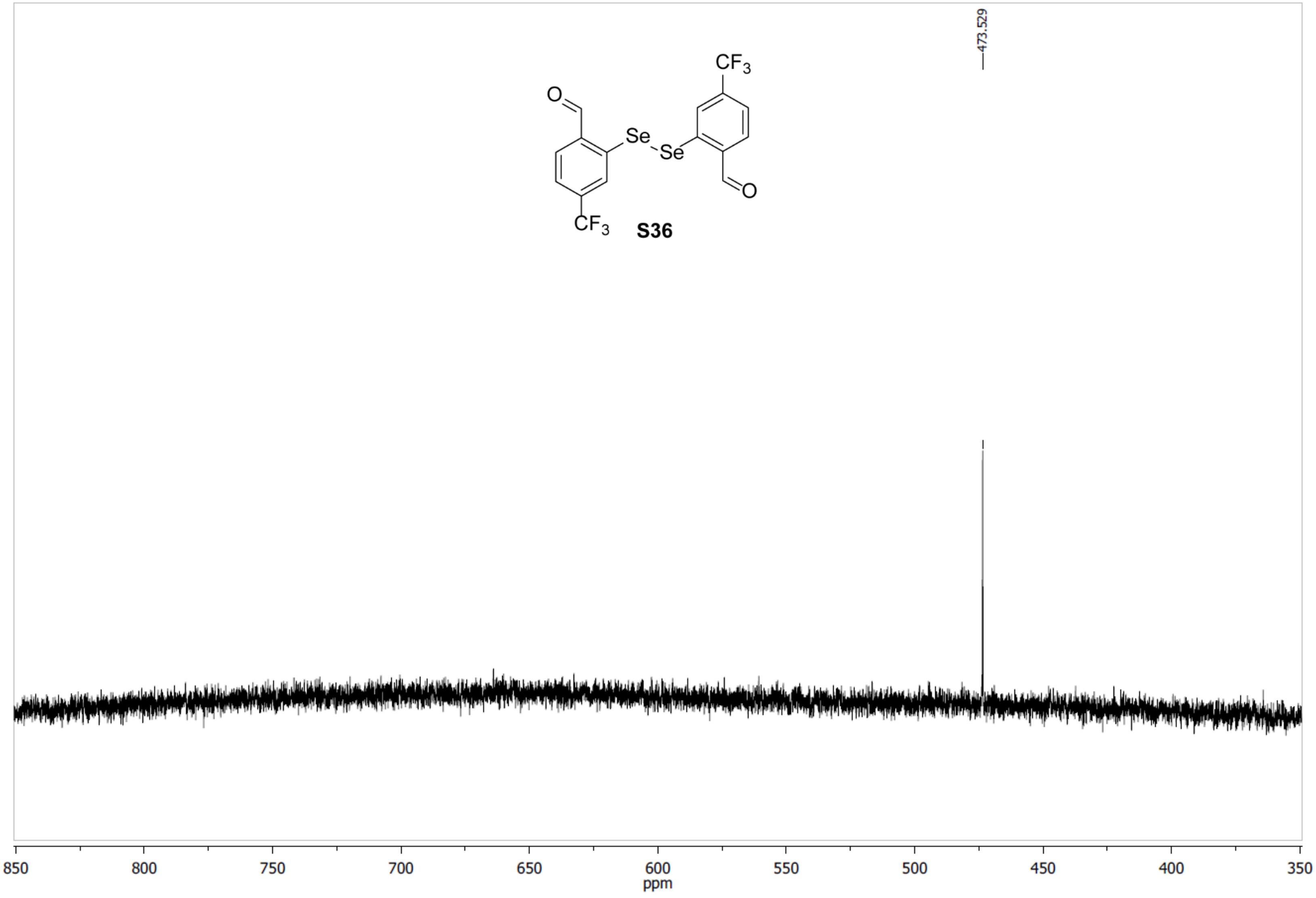

^{1}H NMR in DMSO-d_6

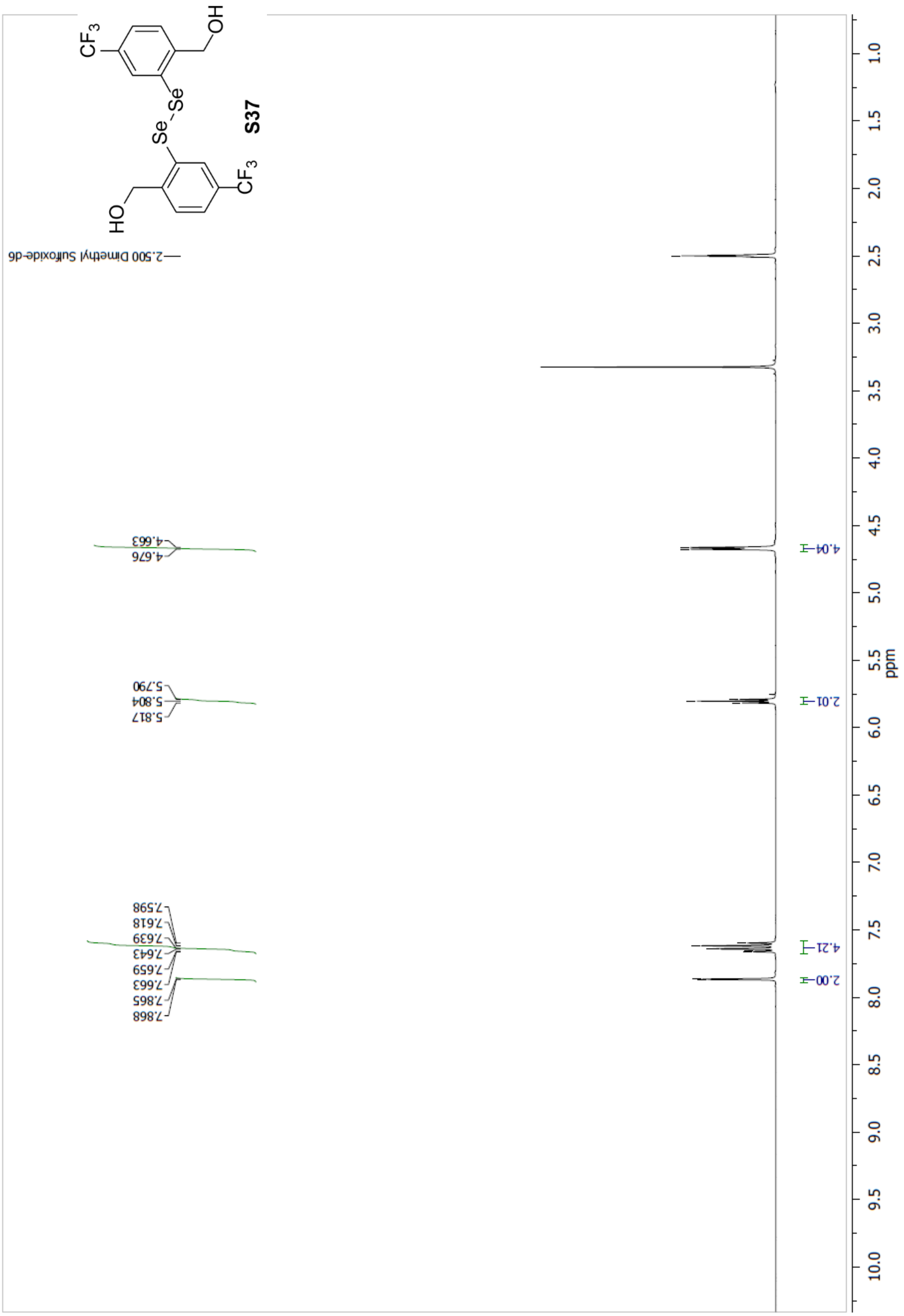

^{13}C NMR in DMSO-d_6

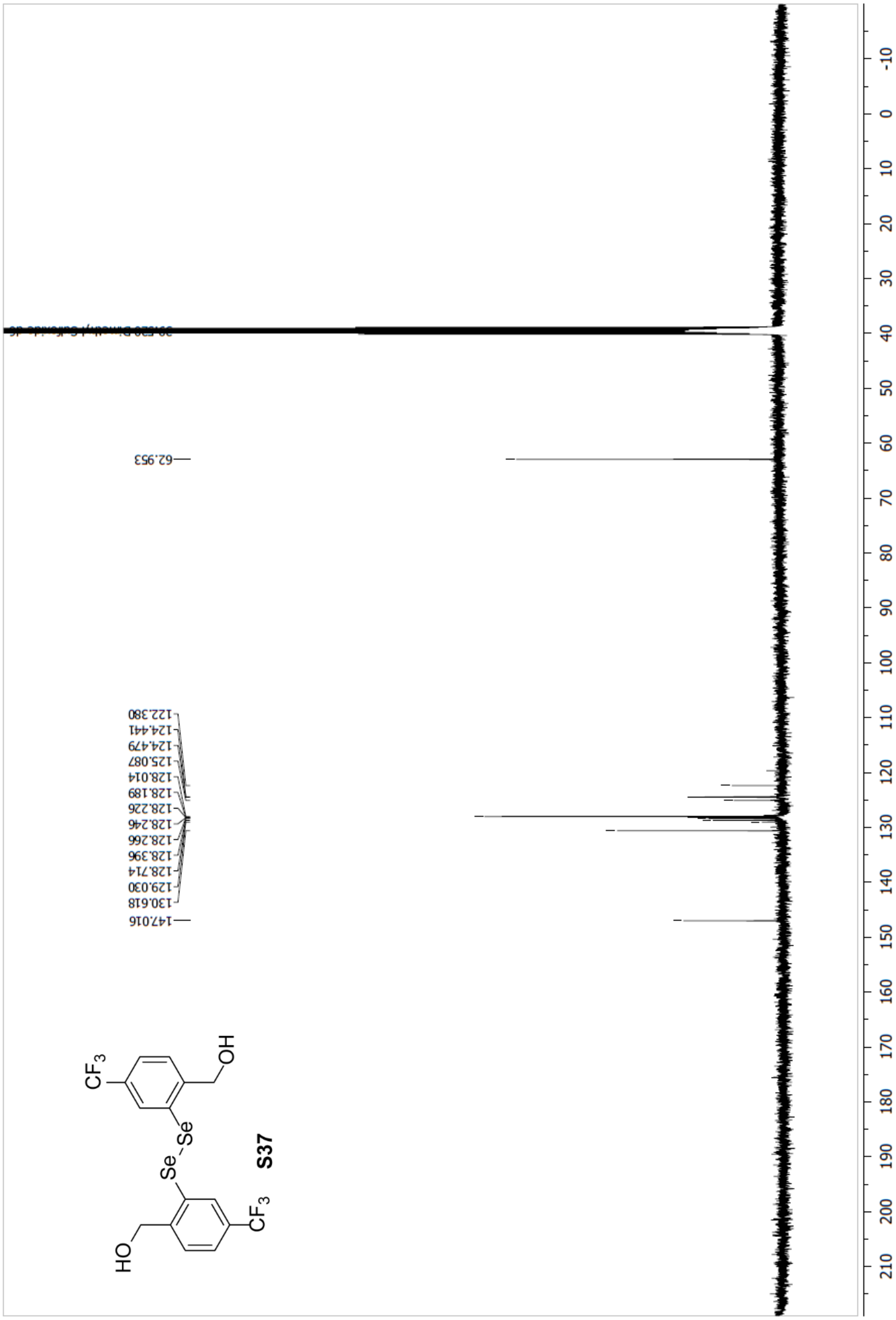

^{19}F NMR in DMSO-d_6

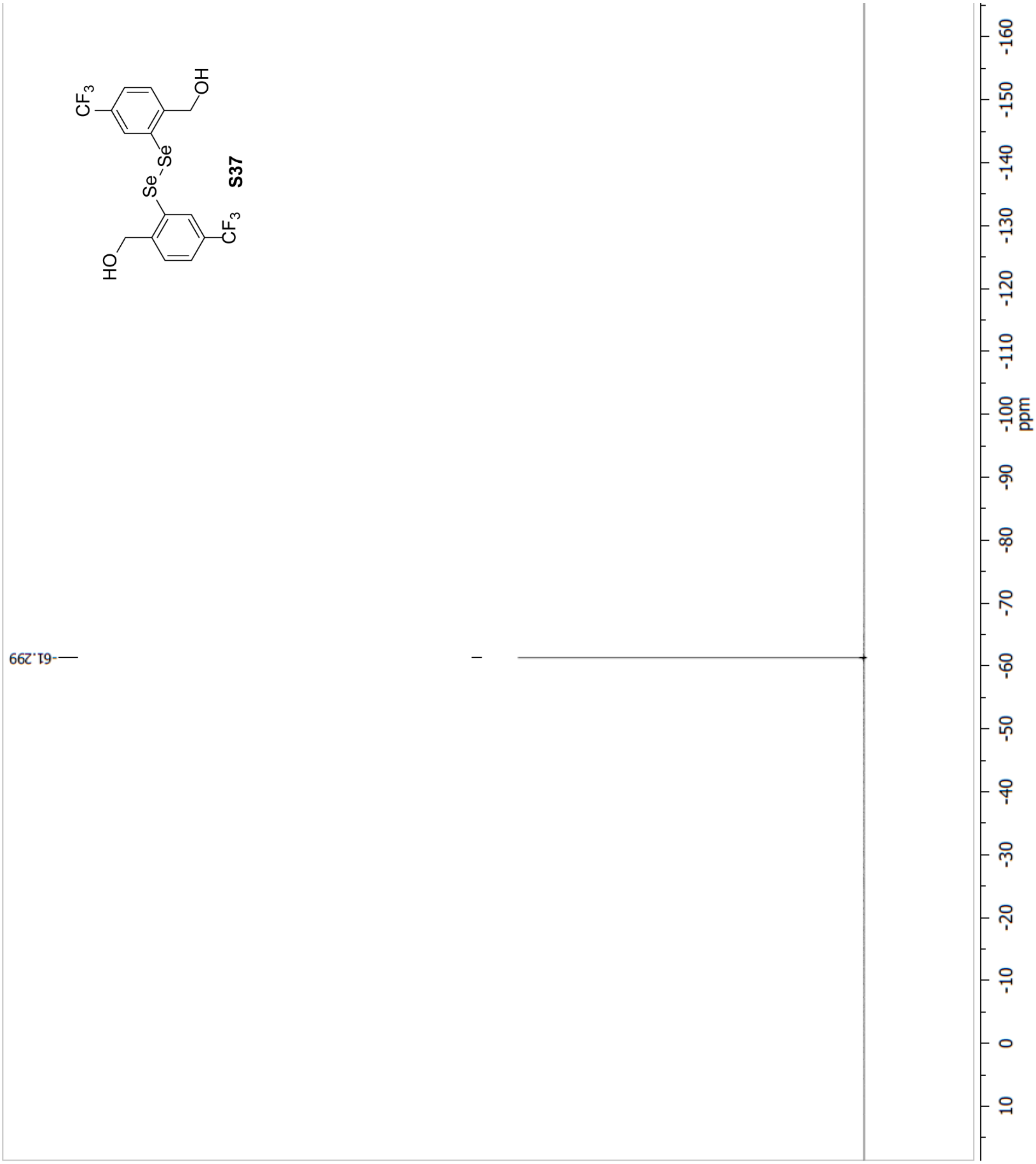

^{77}Se NMR in DMSO-d_6

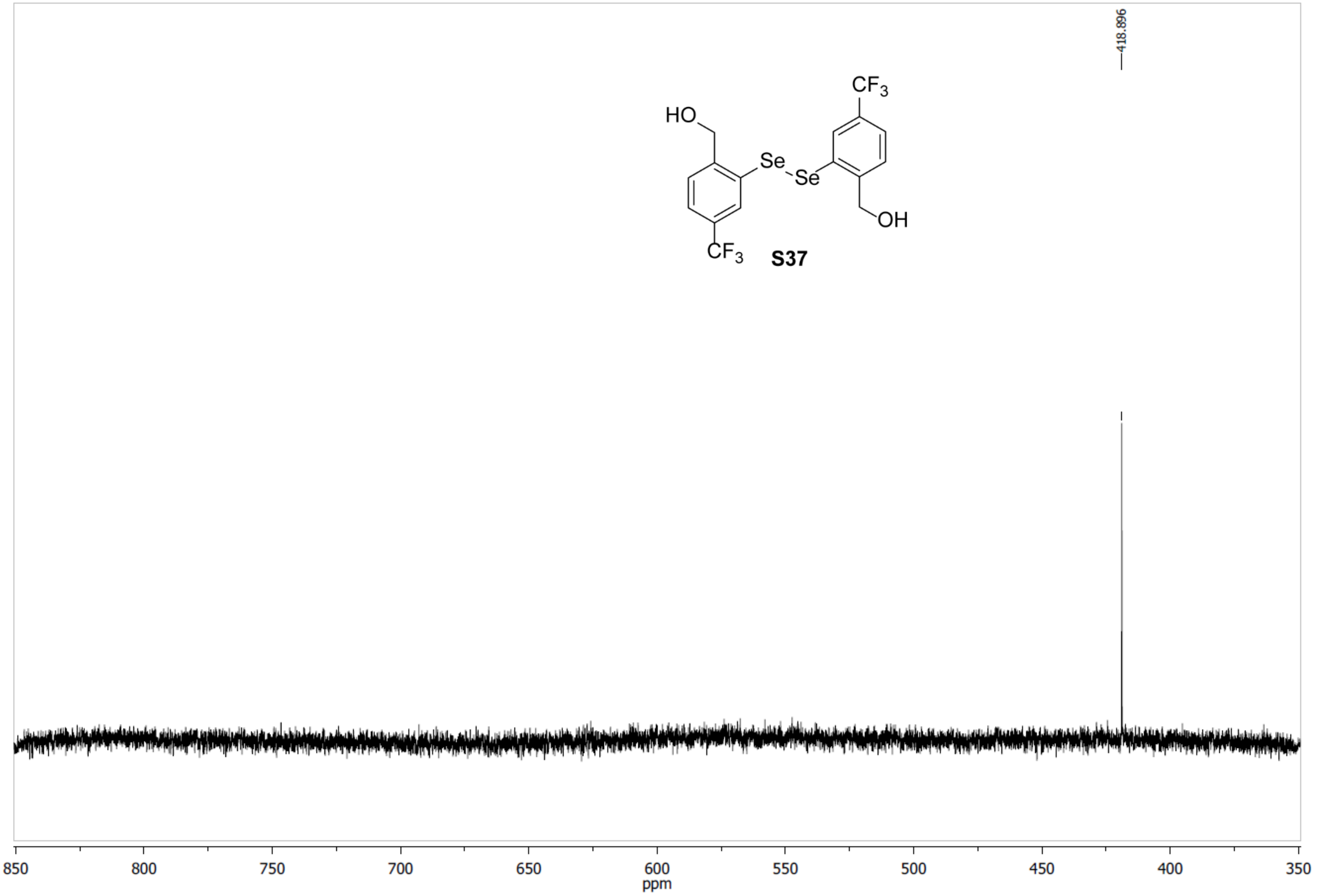

^{1}H NMR in $CDCl_3$

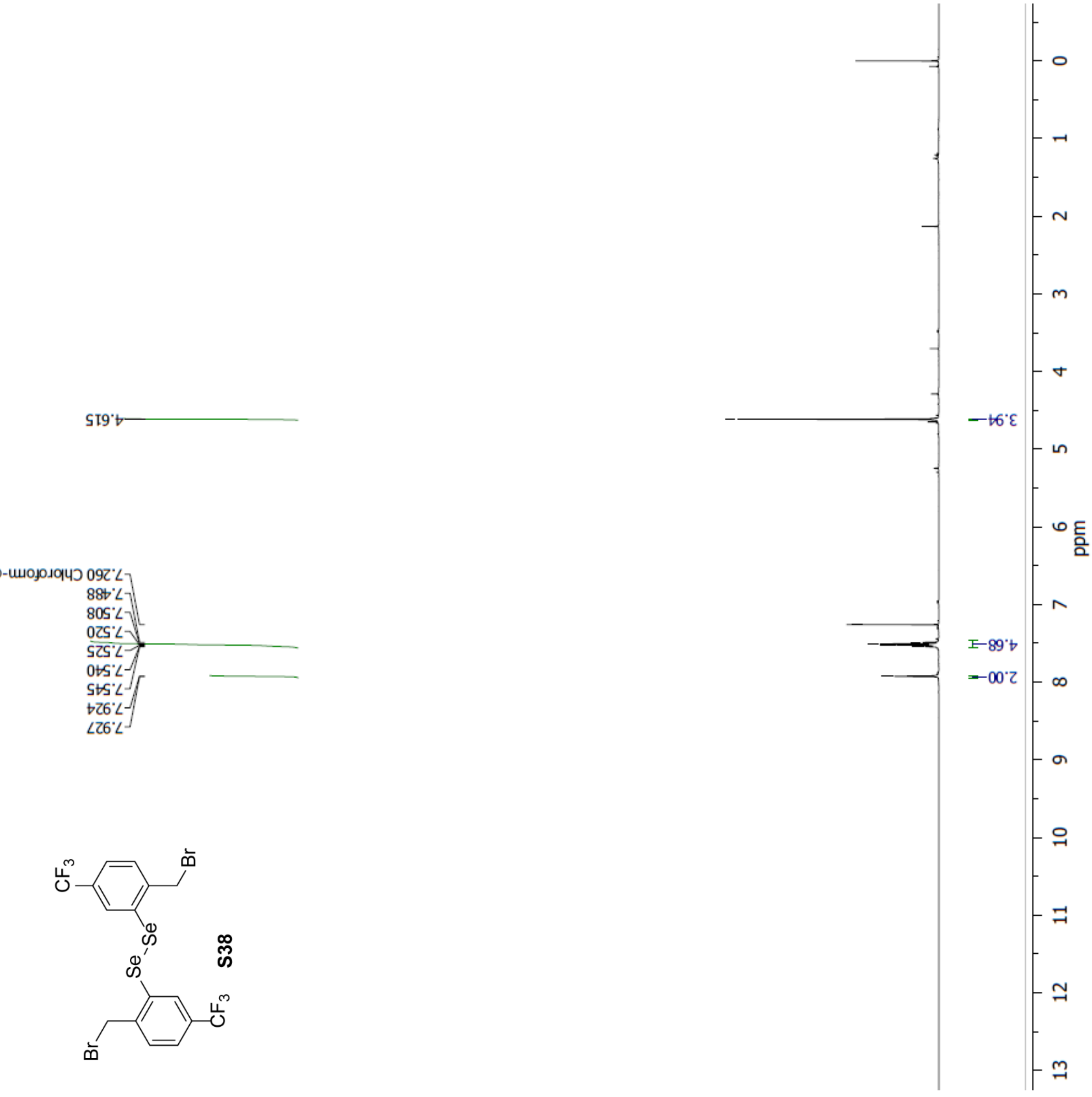

S38

^{13}C NMR in $CDCl_3$

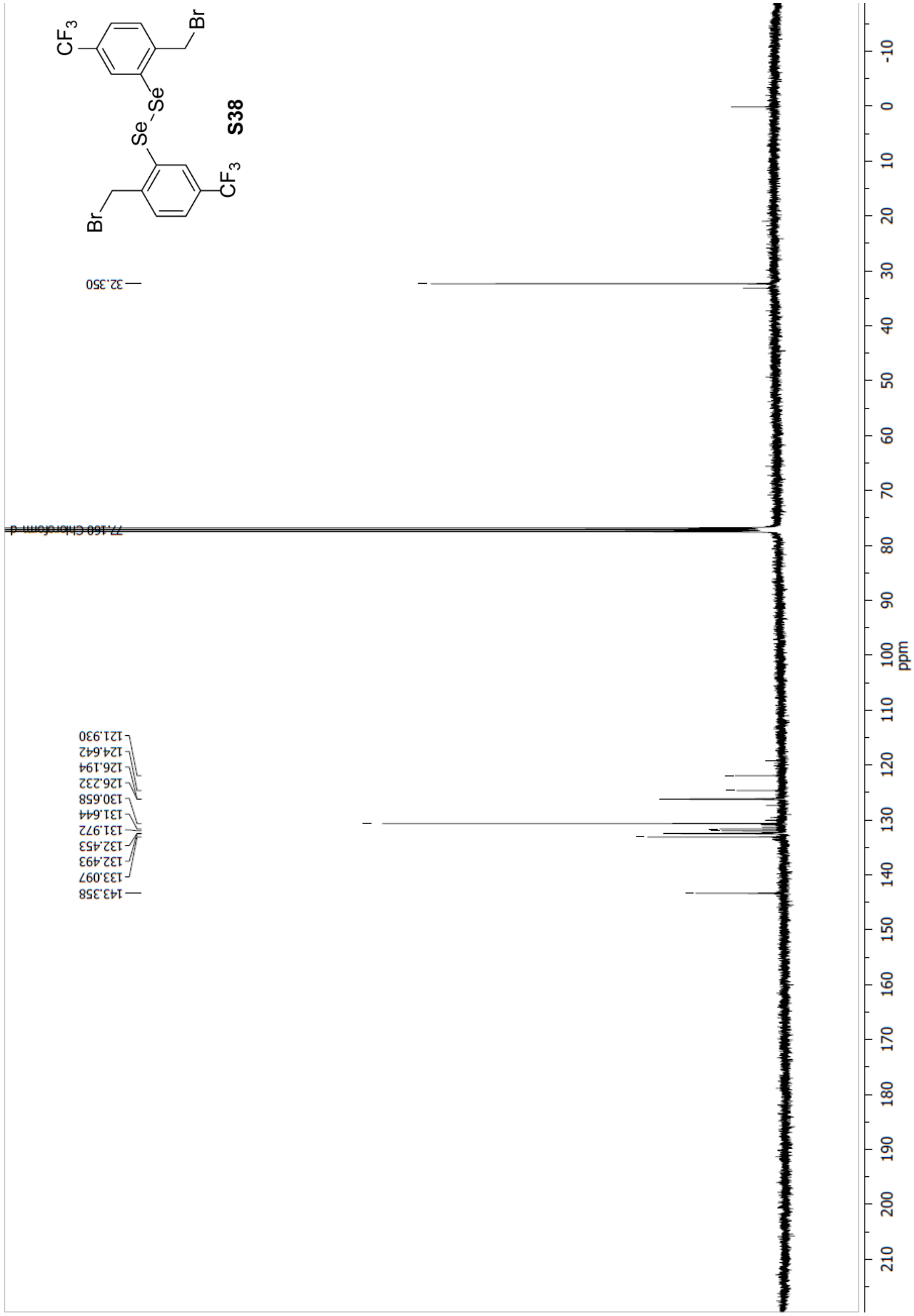

^{19}F NMR in $CDCl_3$

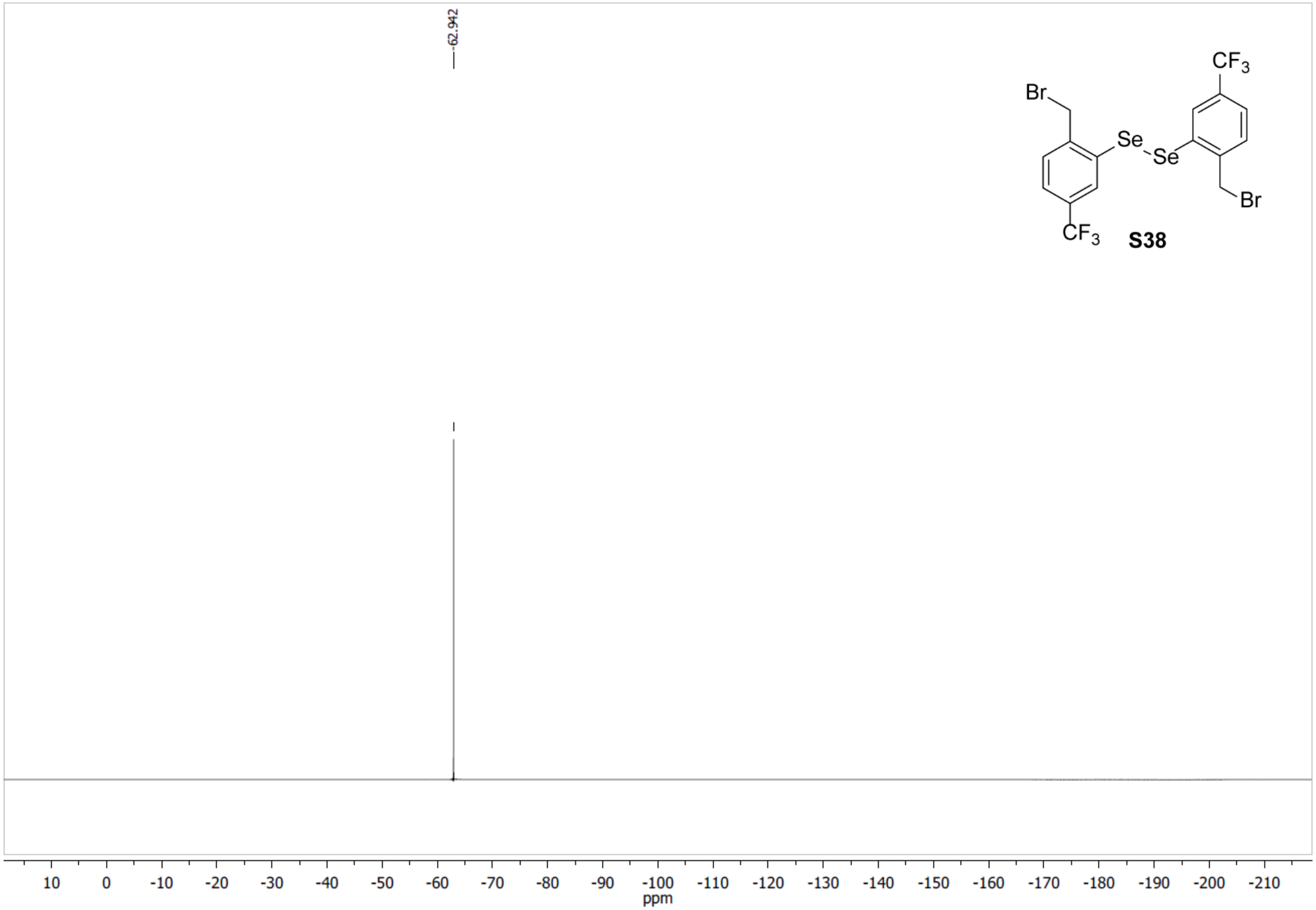

^{77}Se NMR in $CDCl_3$

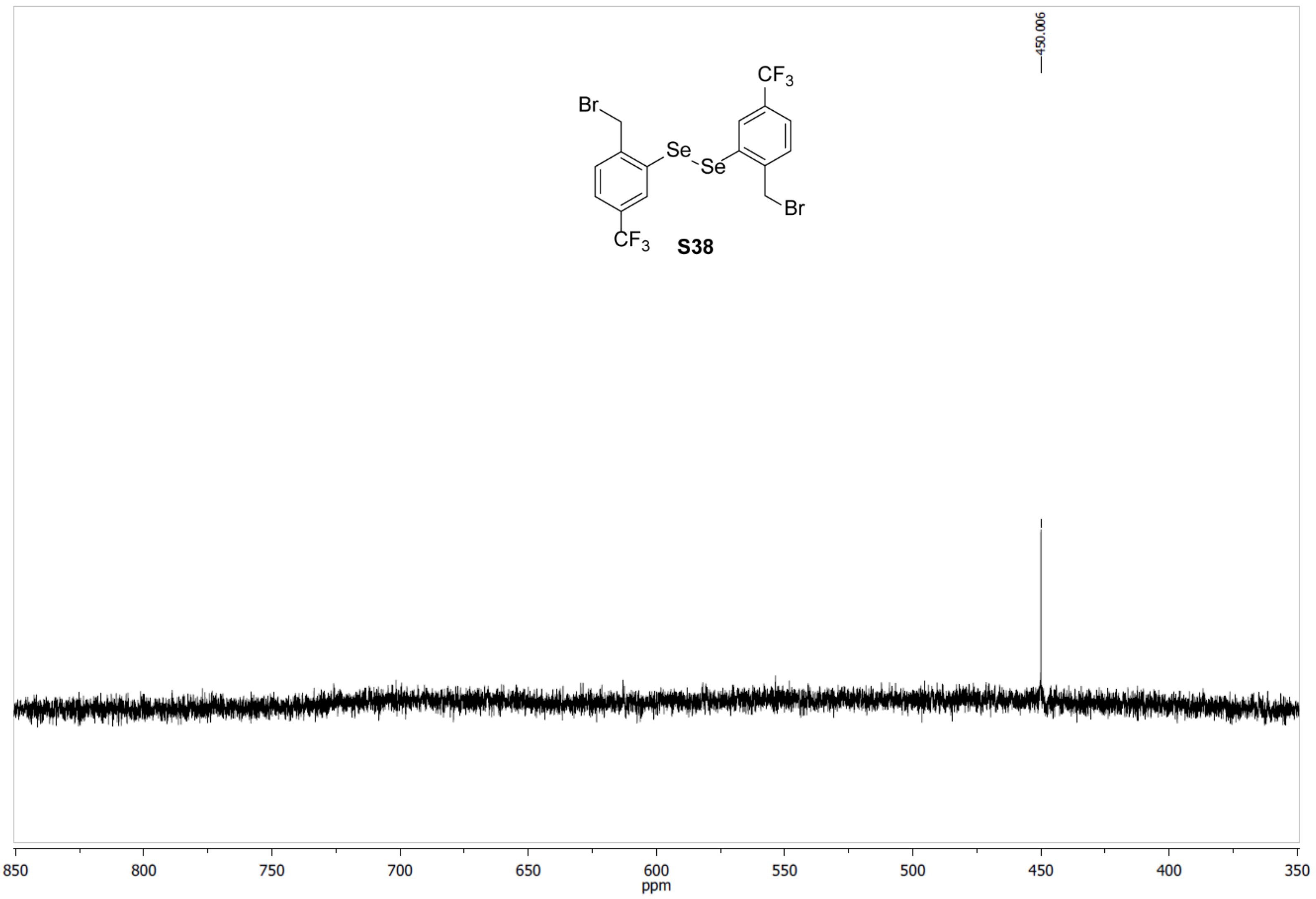

^{1}H NMR in $CDCl_3$

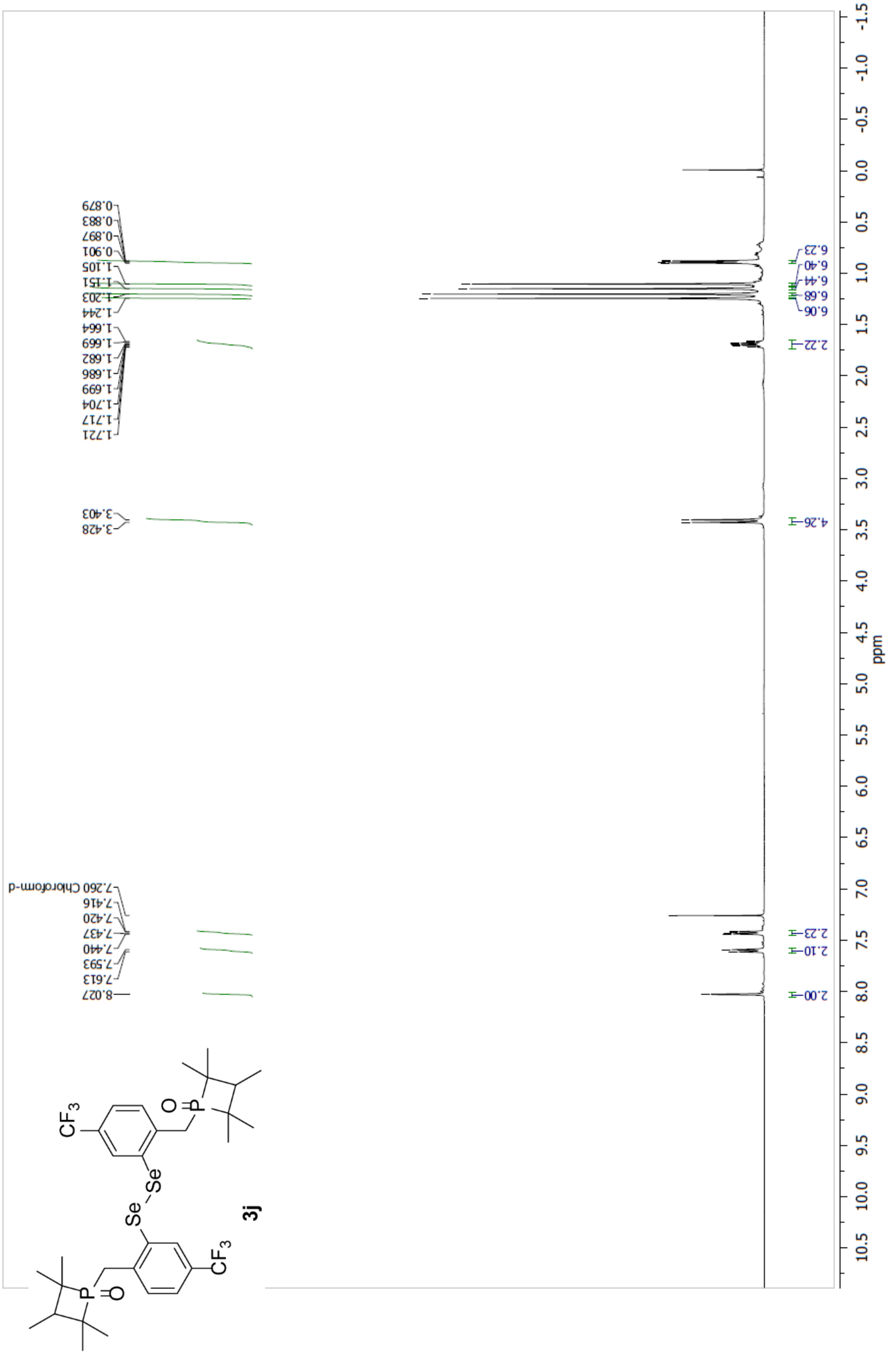

^{13}C NMR in $CDCl_3$

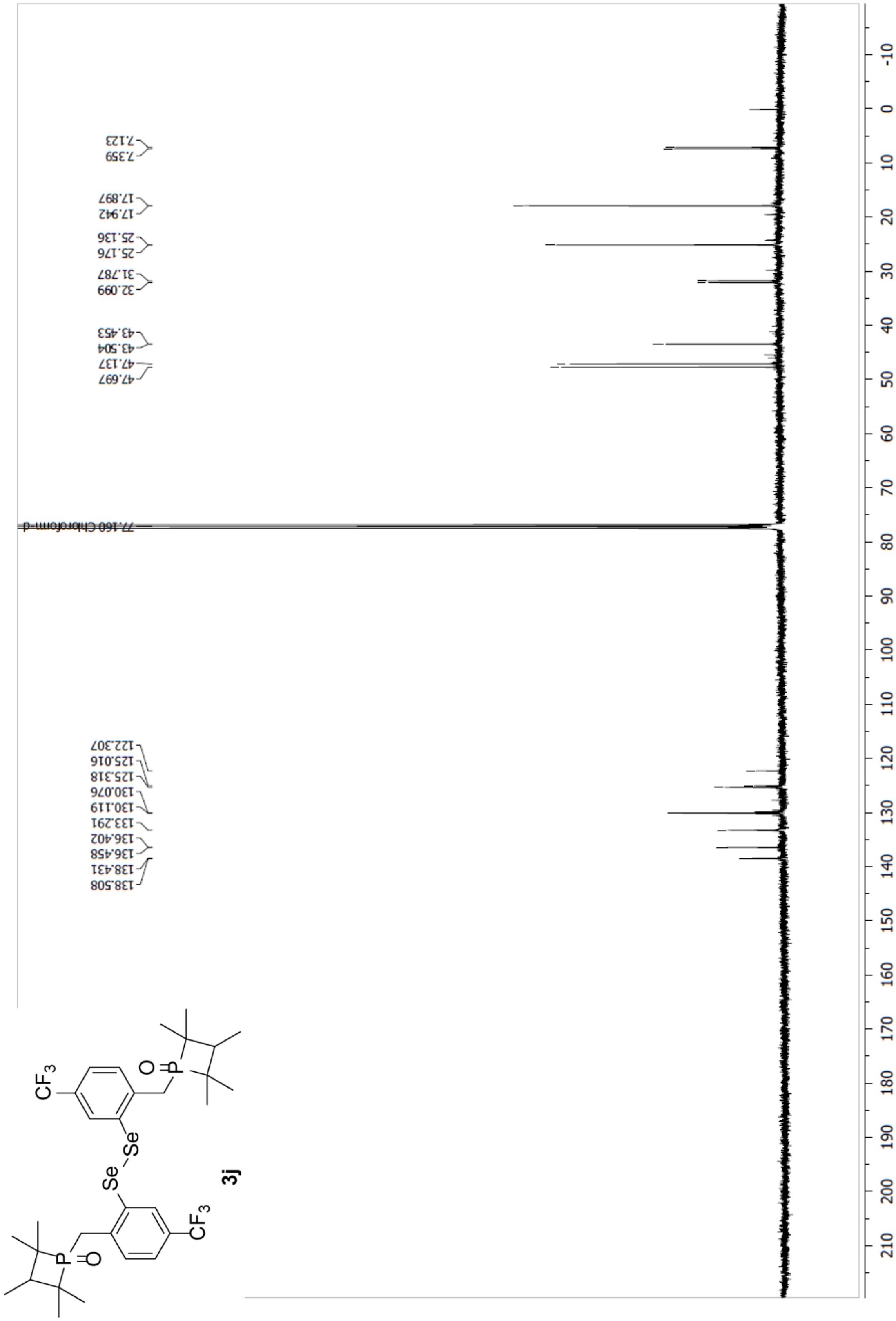

^{19}F NMR in $CDCl_3$

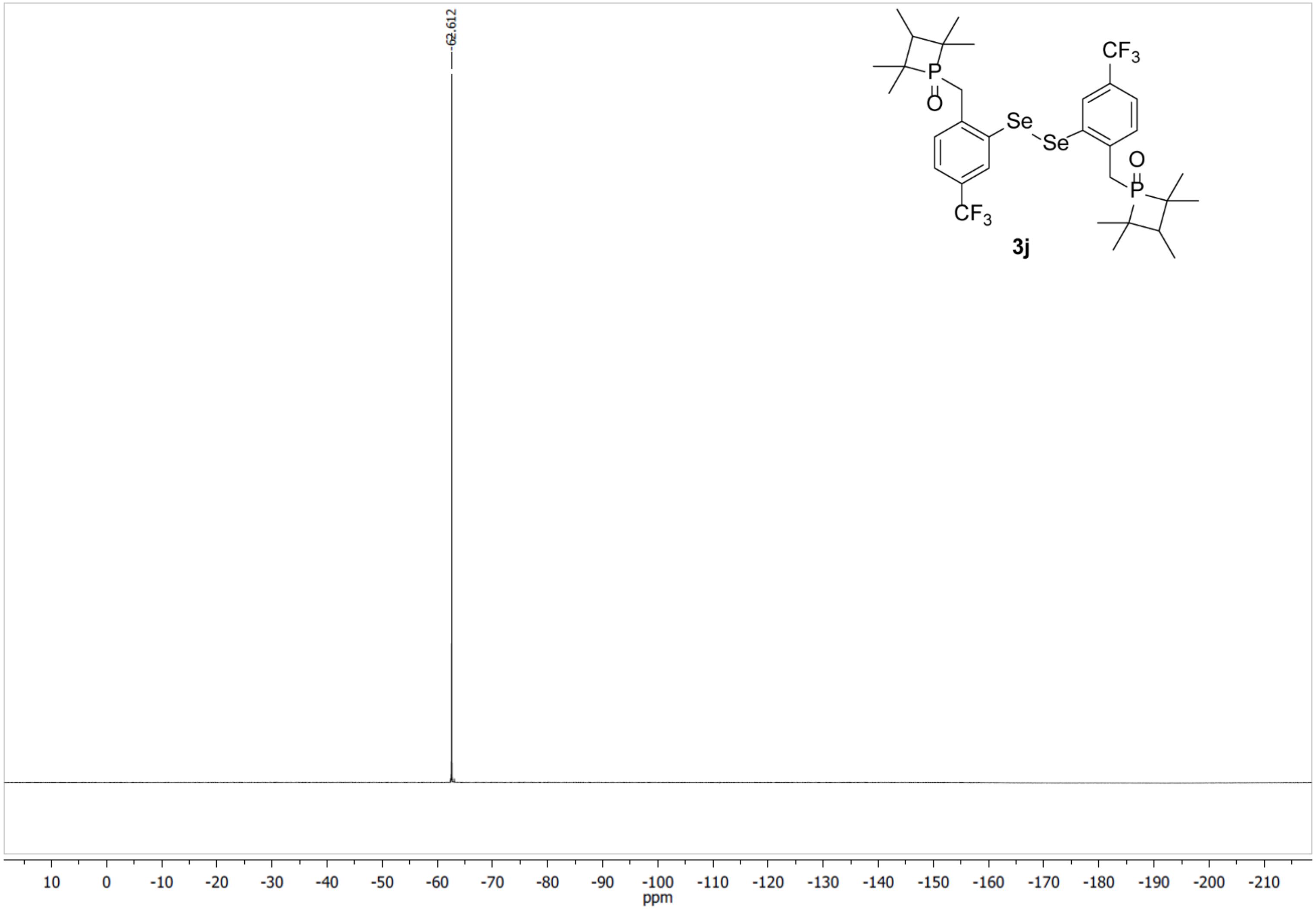

^{31}P NMR in $CDCl_3$

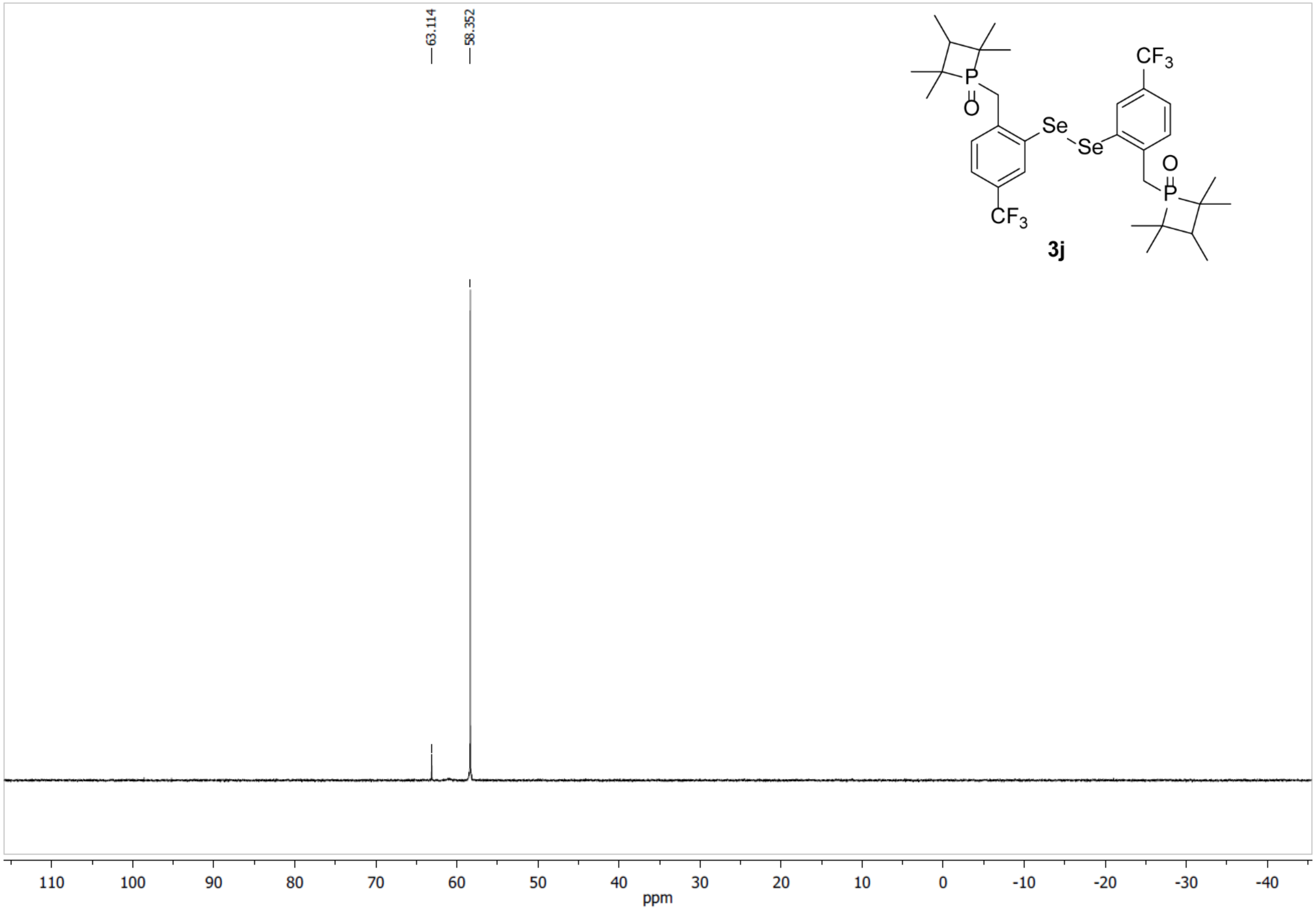

^{77}Se NMR in $CDCl_3$

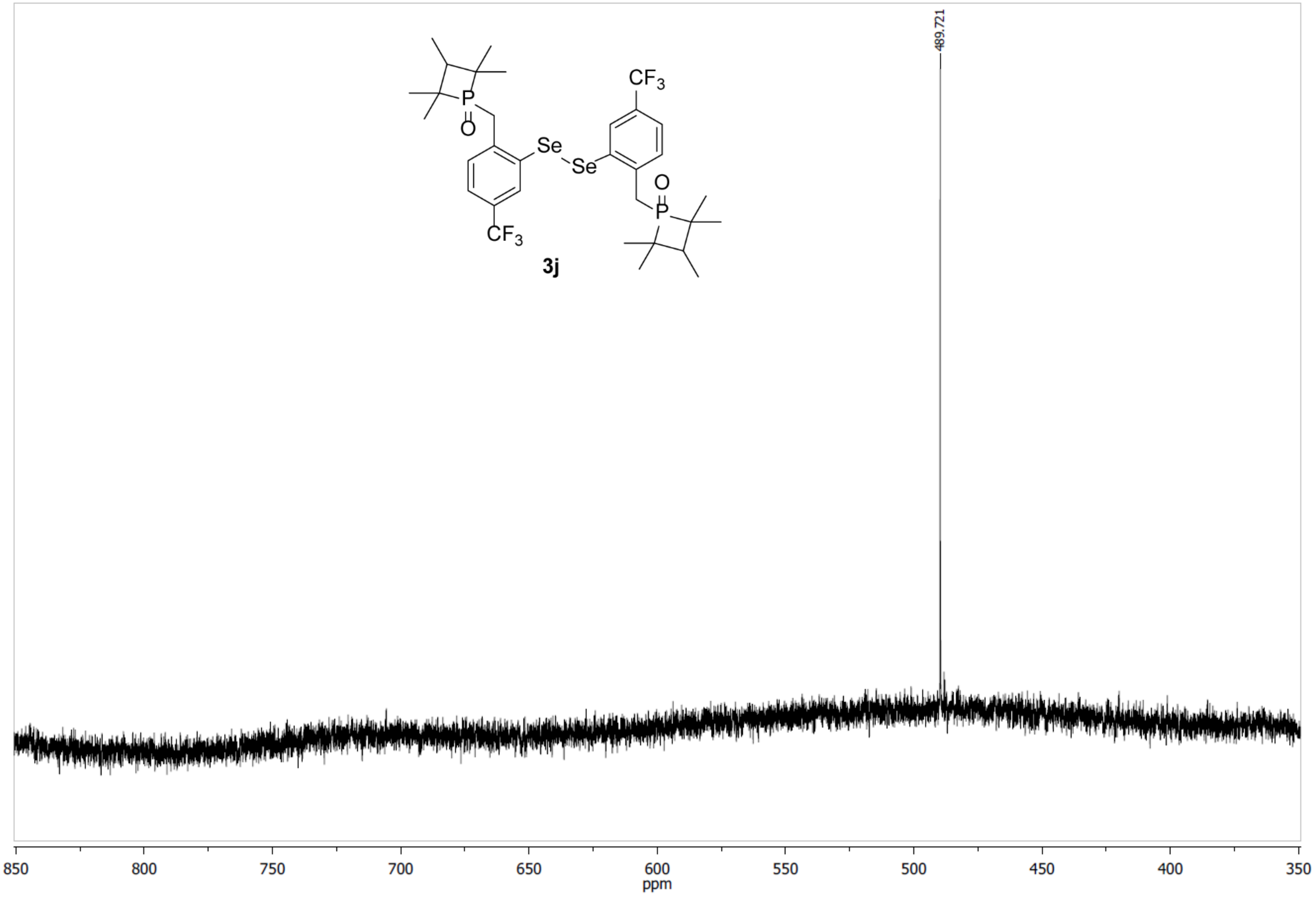

^1H NMR in DMSO-d_6

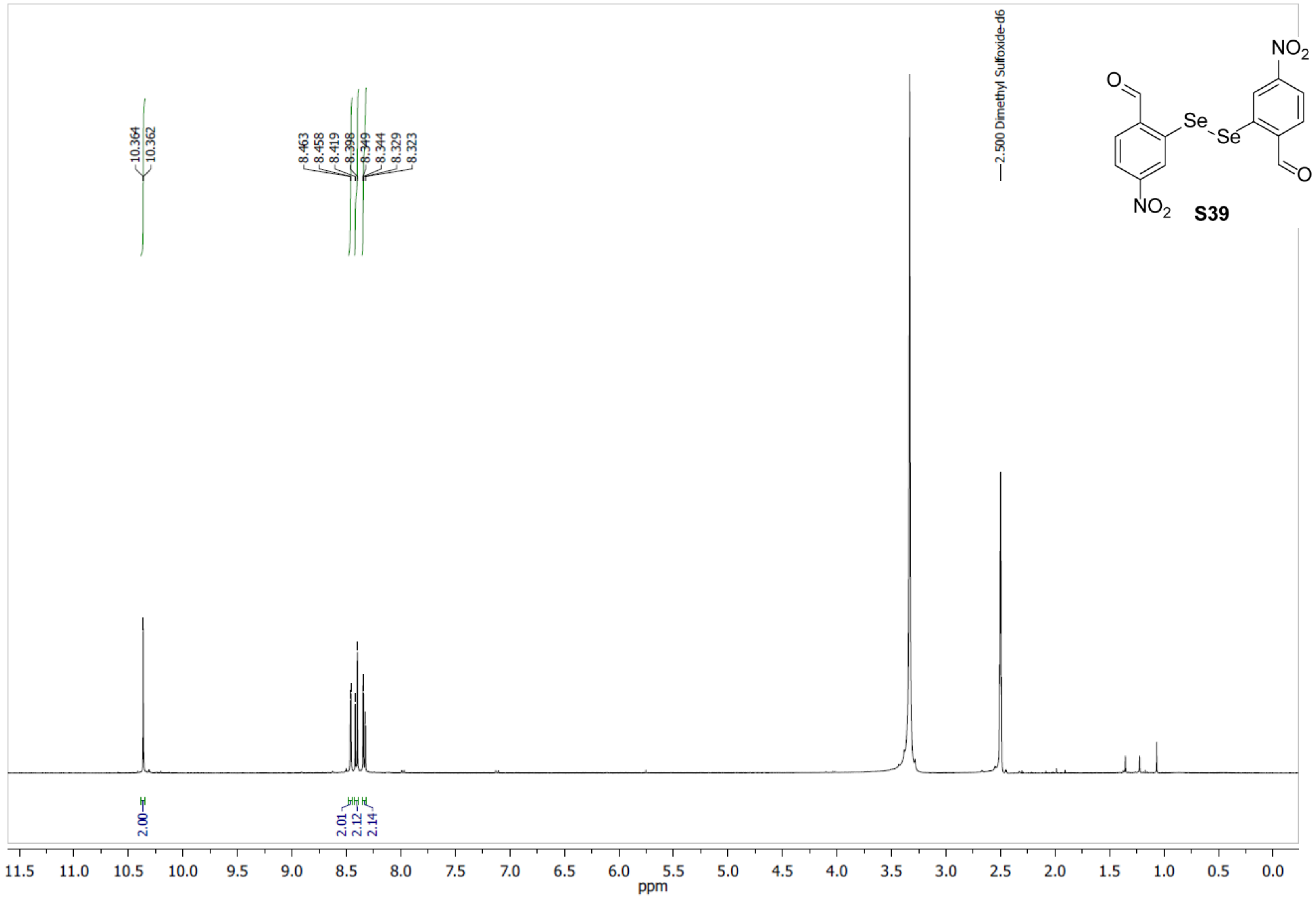

S39

^{13}C NMR in DMSO-d_6

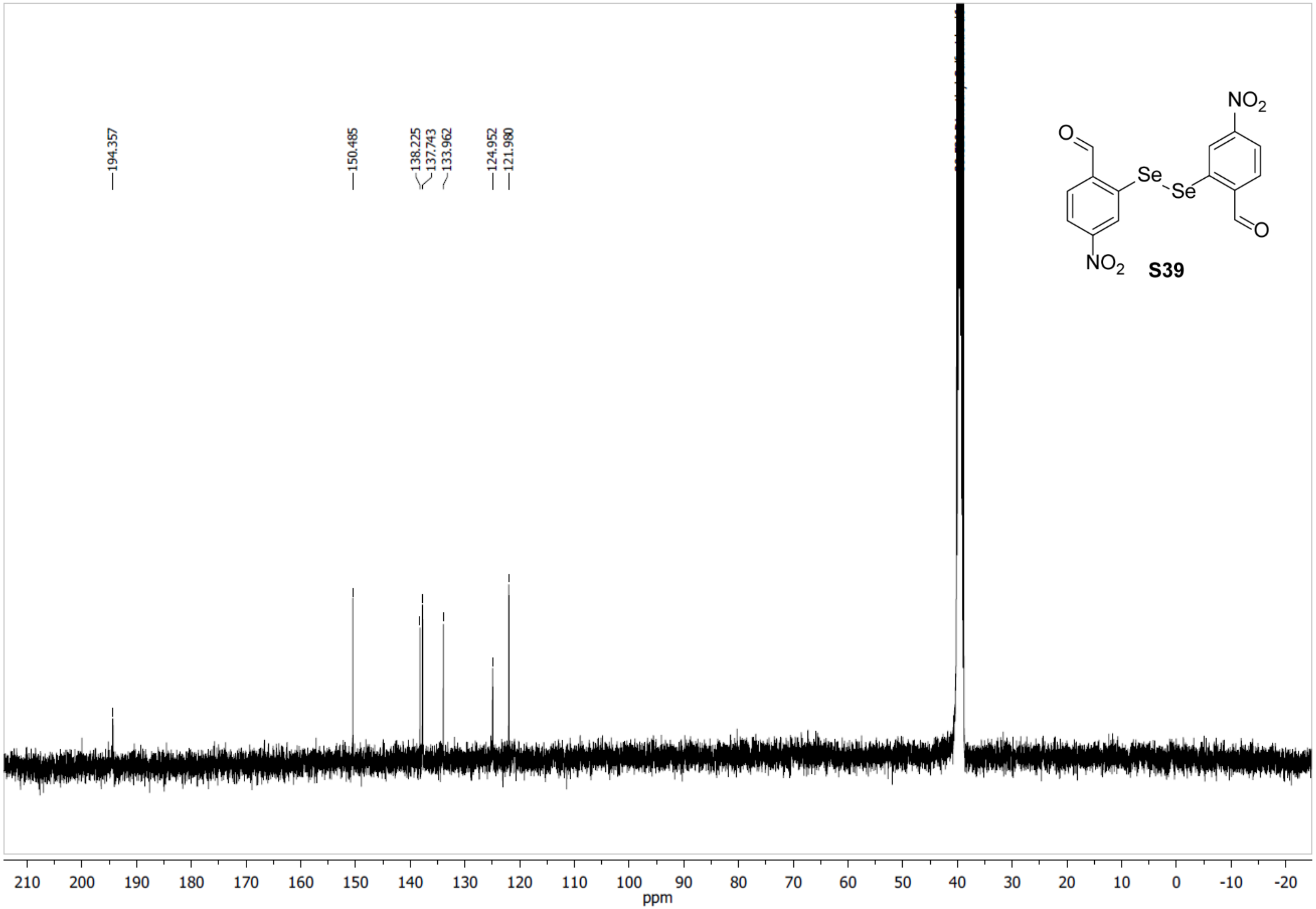

^{77}Se NMR in DMSO-d$_6$

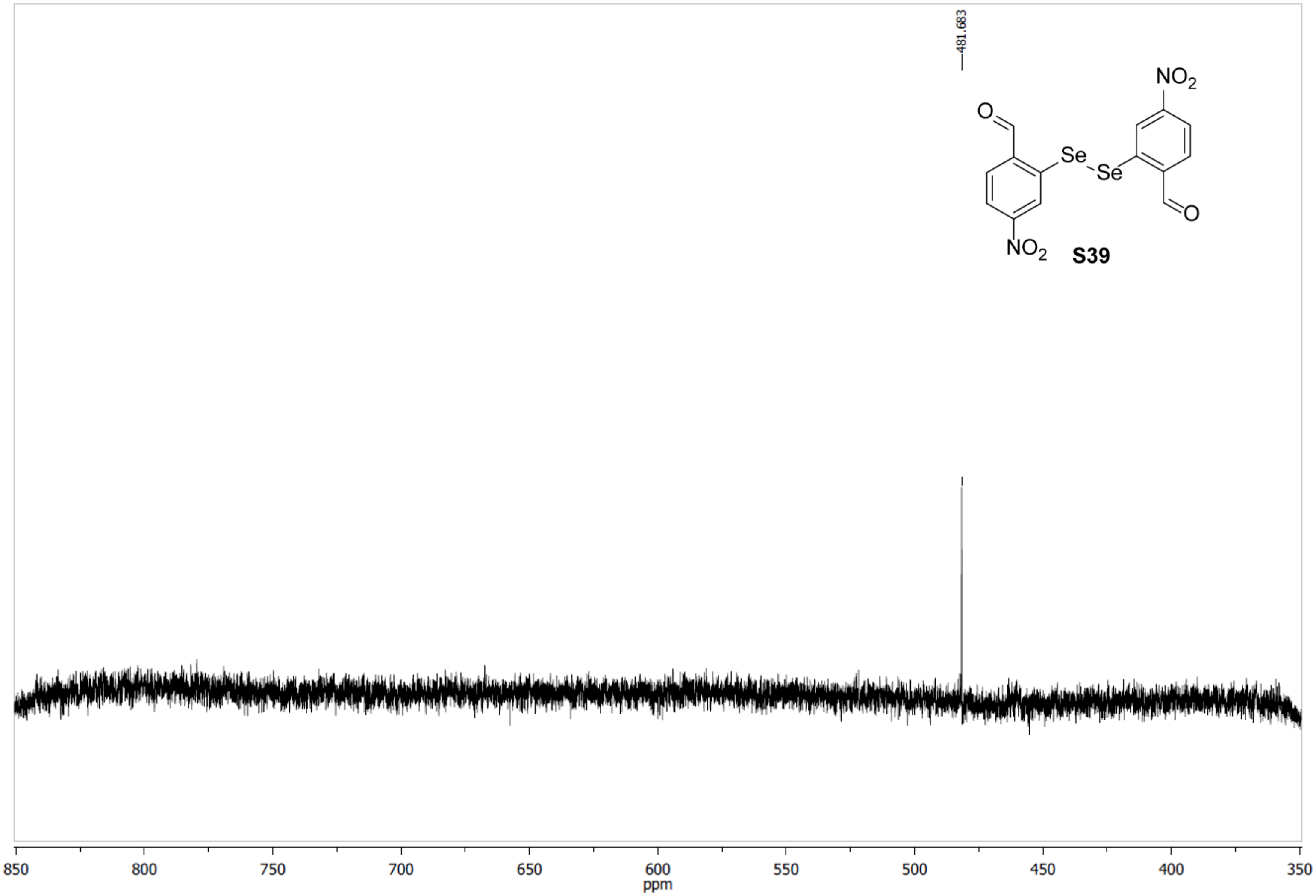

^1H NMR in DMSO-d_6

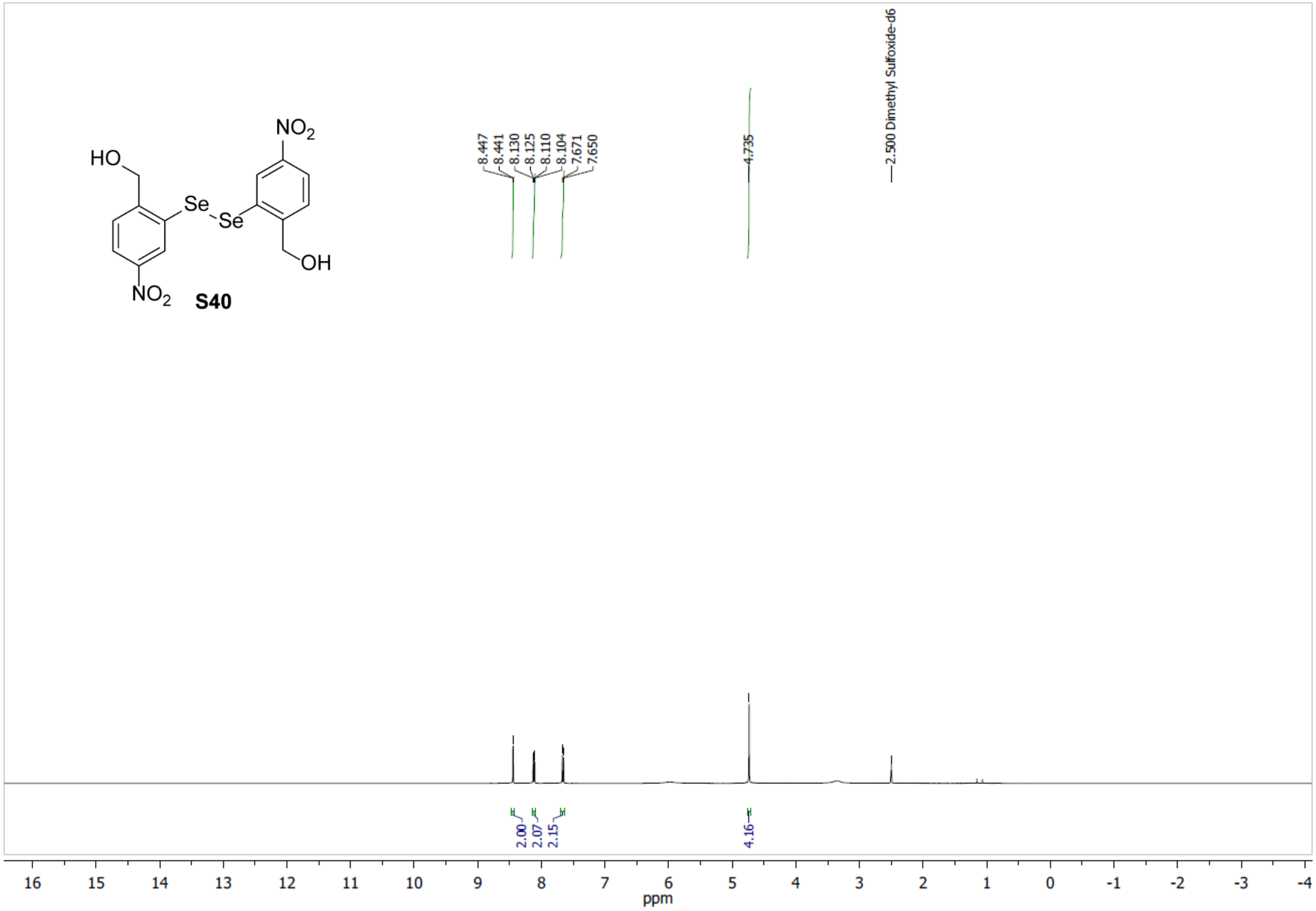

^{13}C NMR in DMSO-d_6

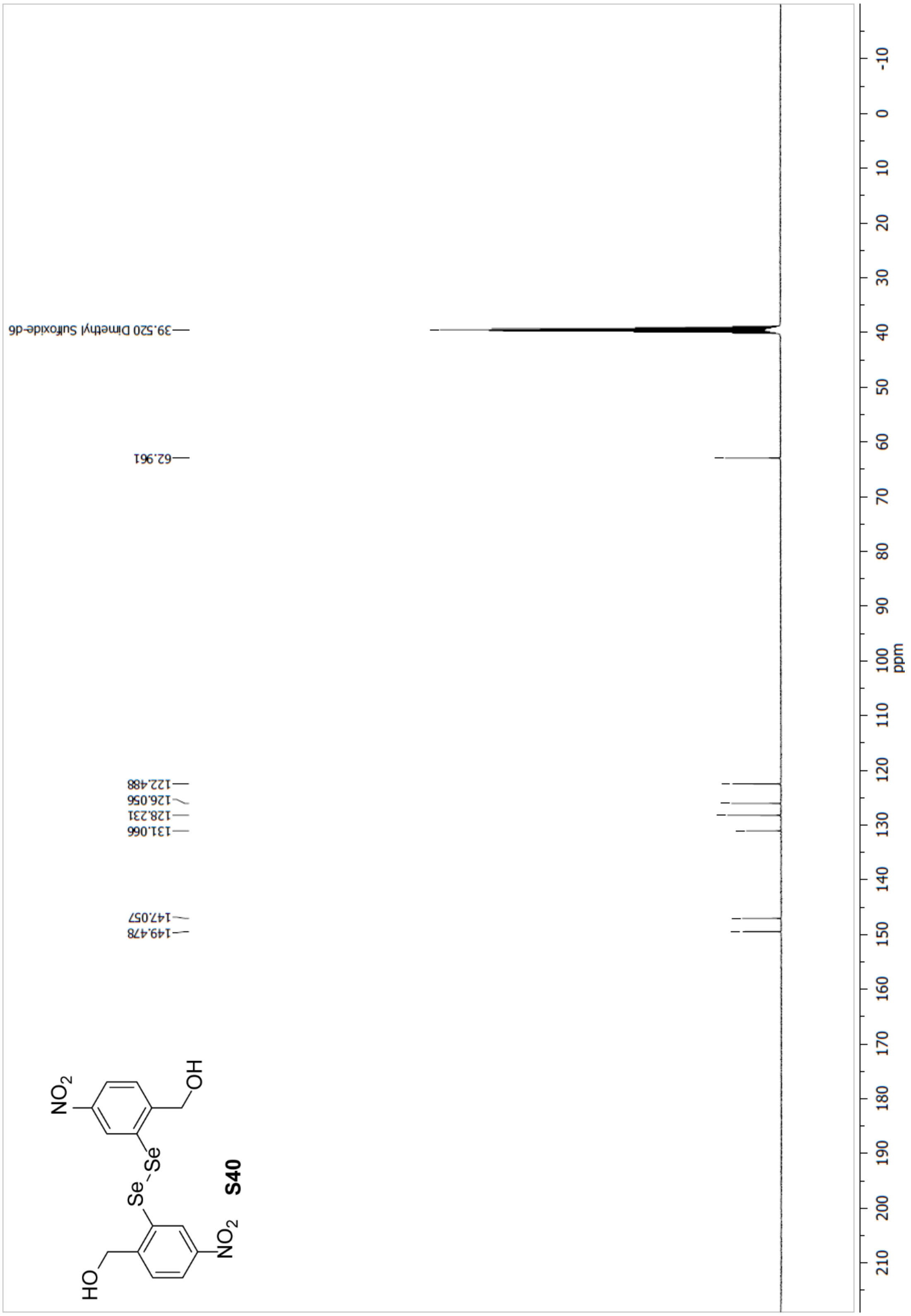

^{77}Se NMR in DMSO-d$_6$

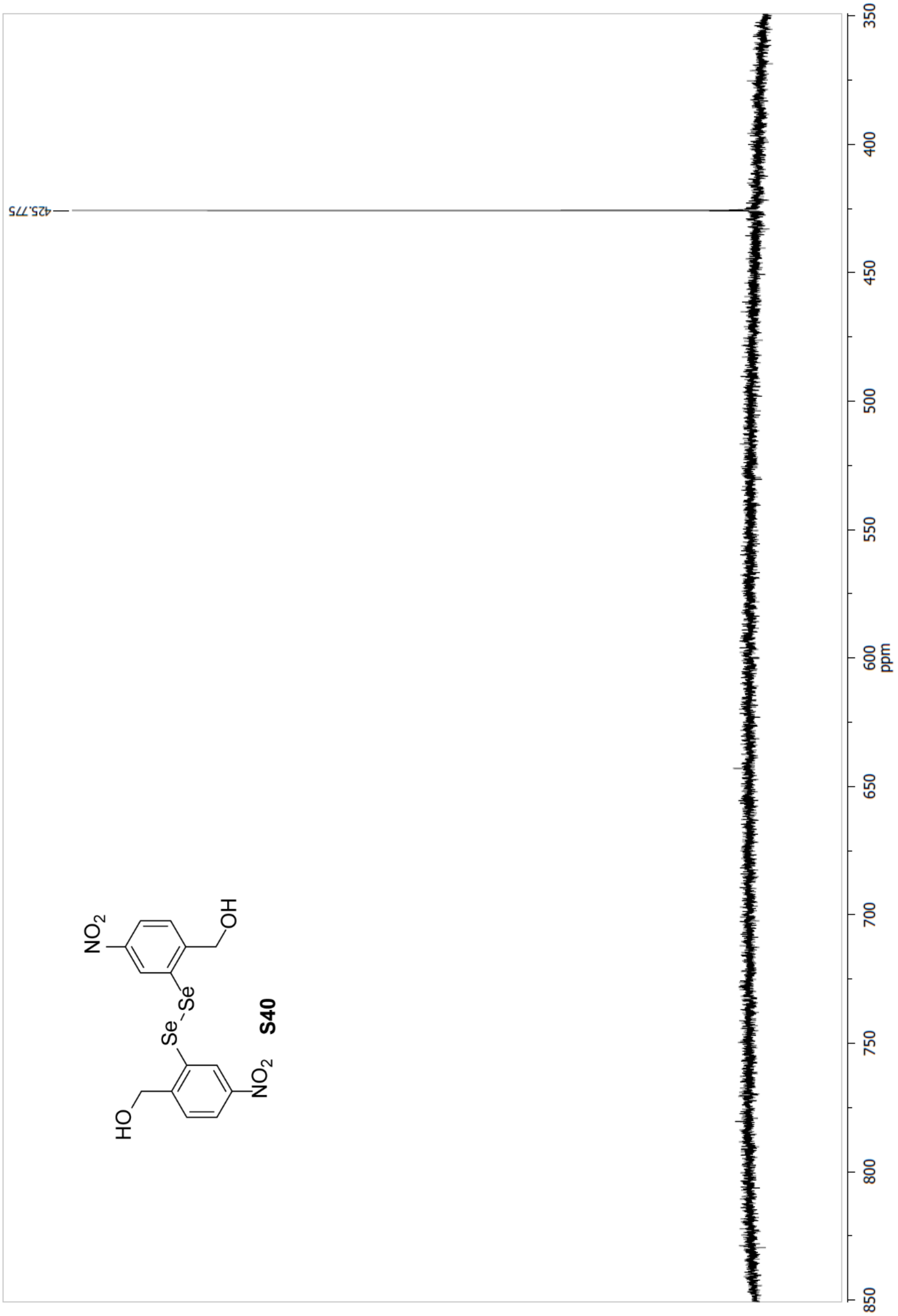

^{1}H NMR in $CDCl_3$

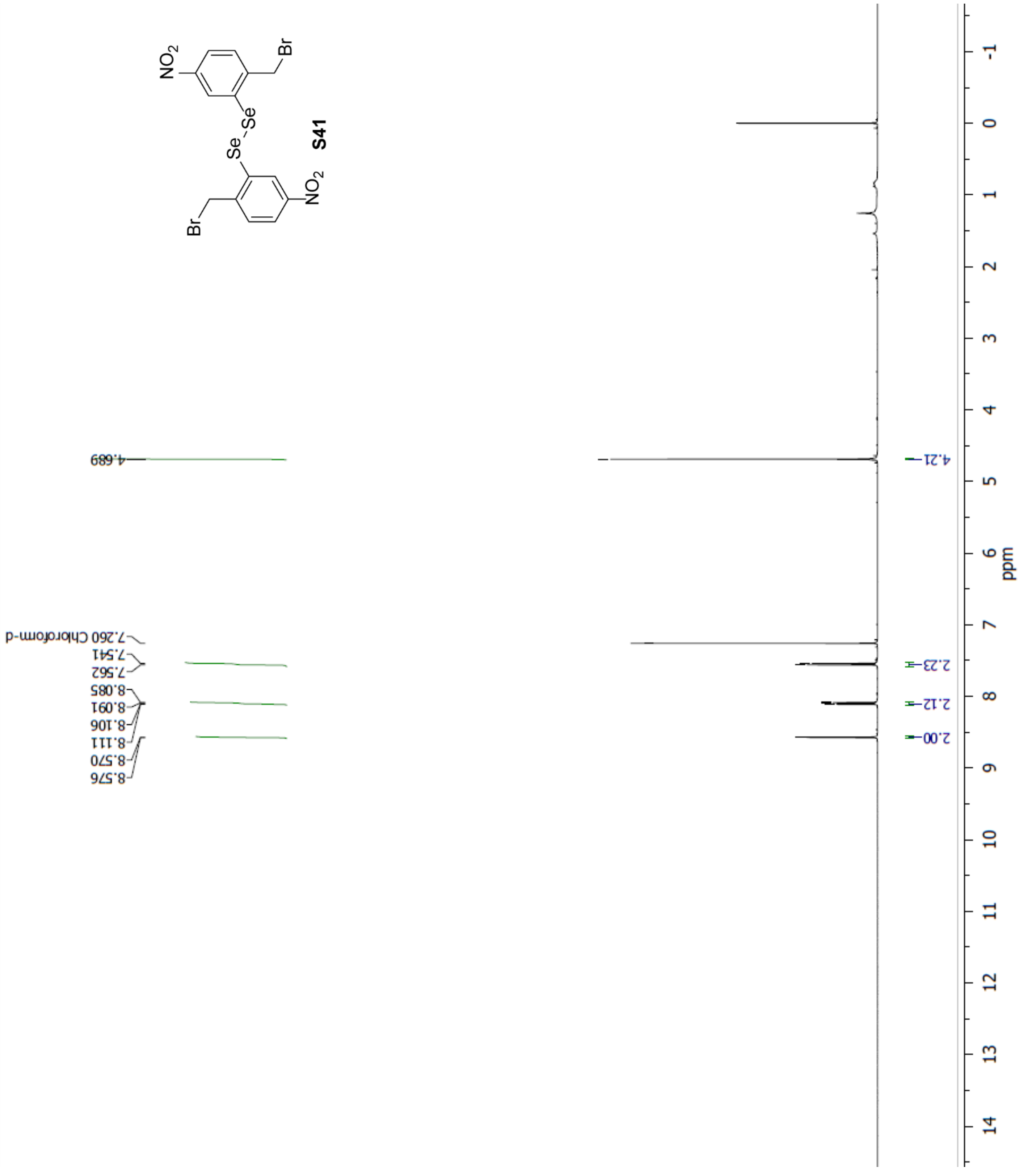

^{13}C NMR in $CDCl_3$

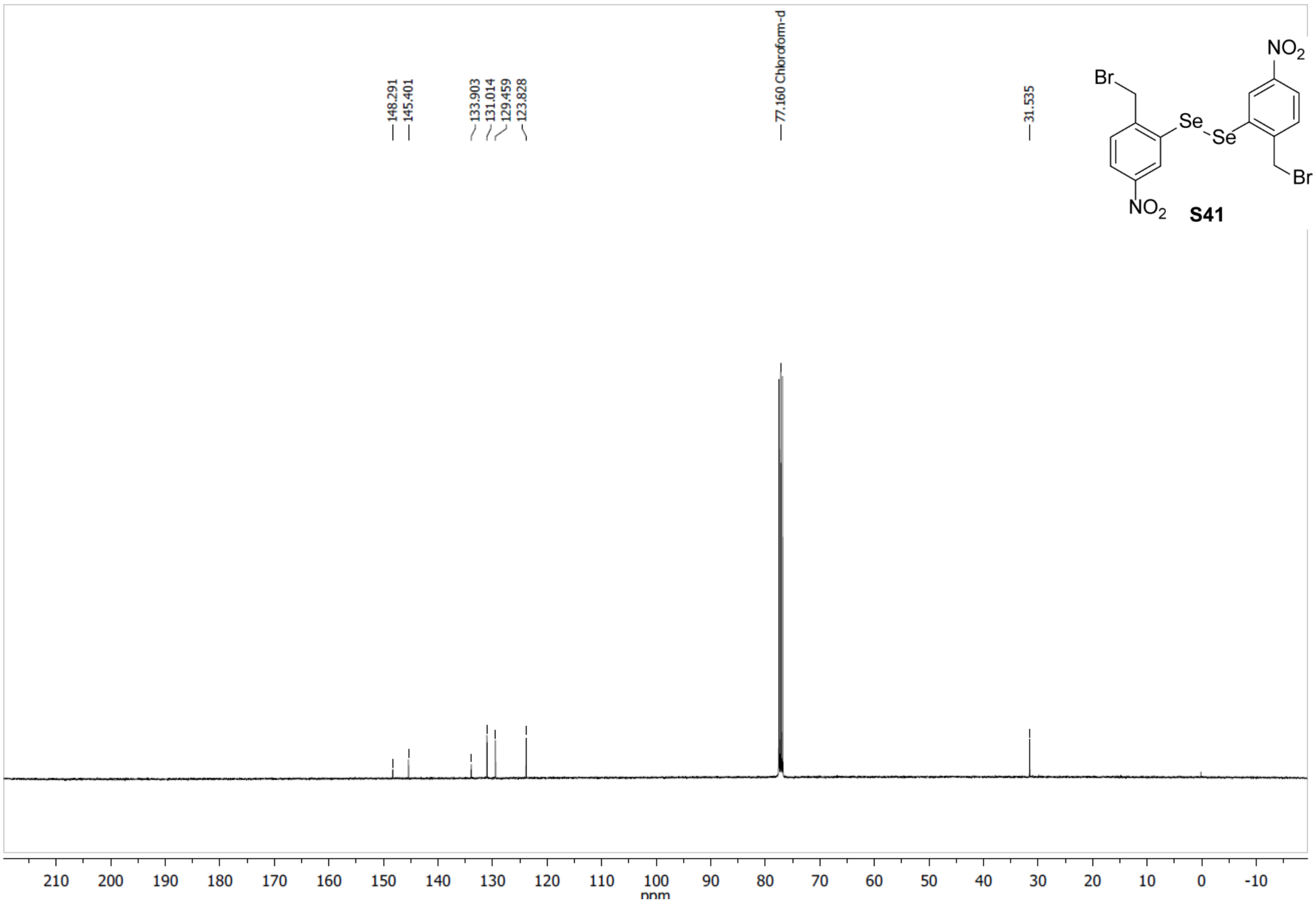

^{77}Se NMR in $CDCl_3$

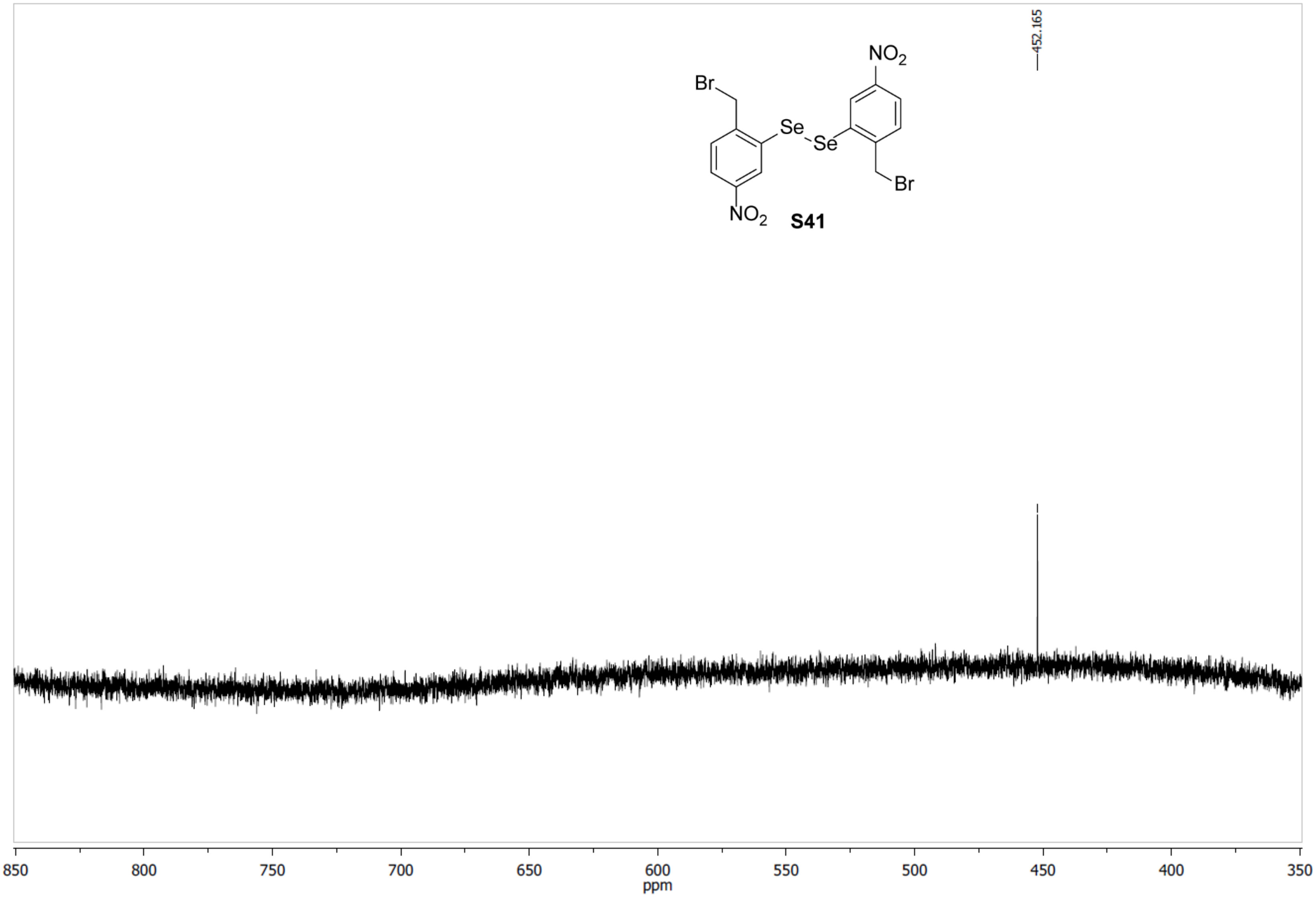

^{1}H NMR in $CDCl_3$

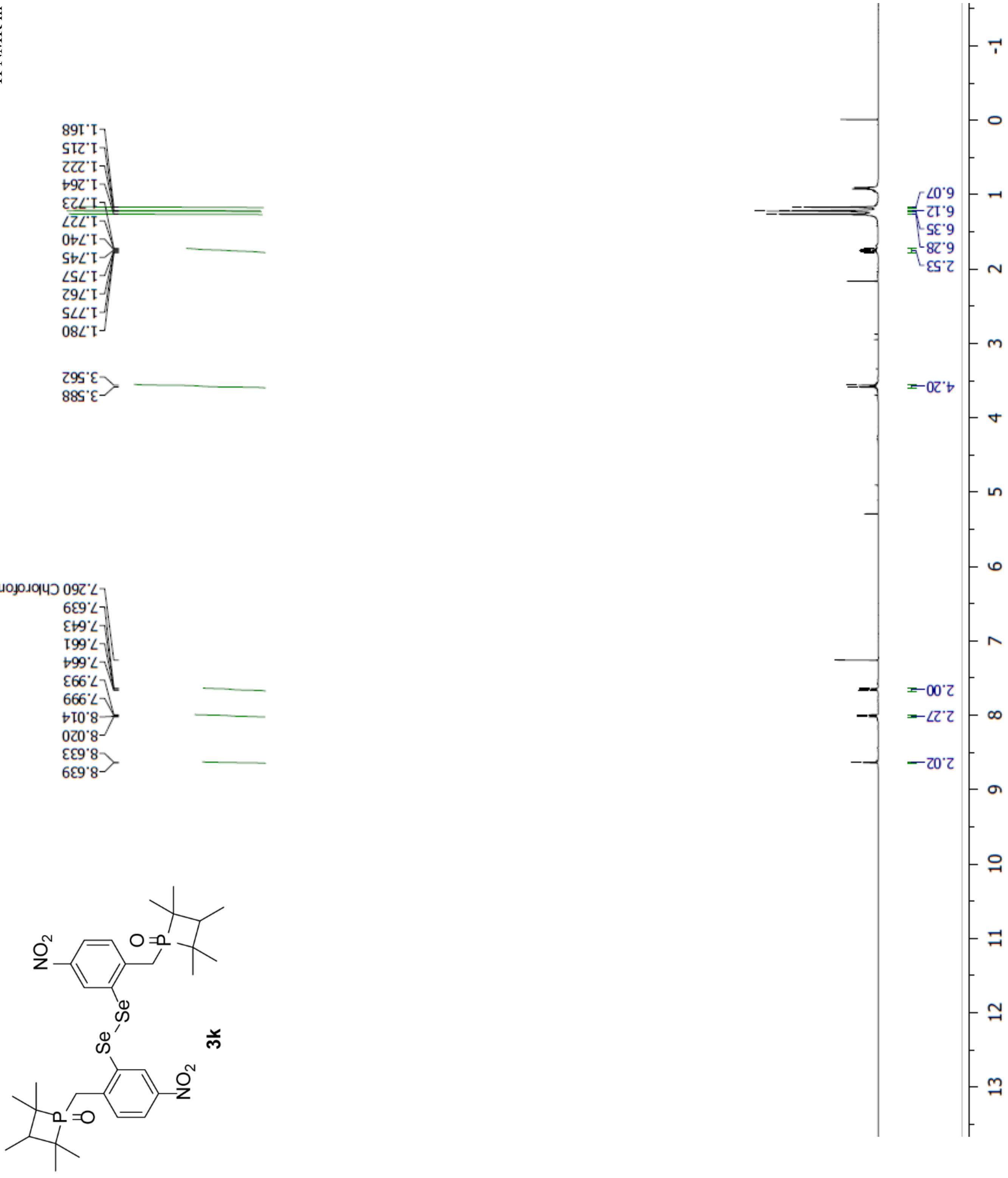

^{13}C NMR in $CDCl_3$

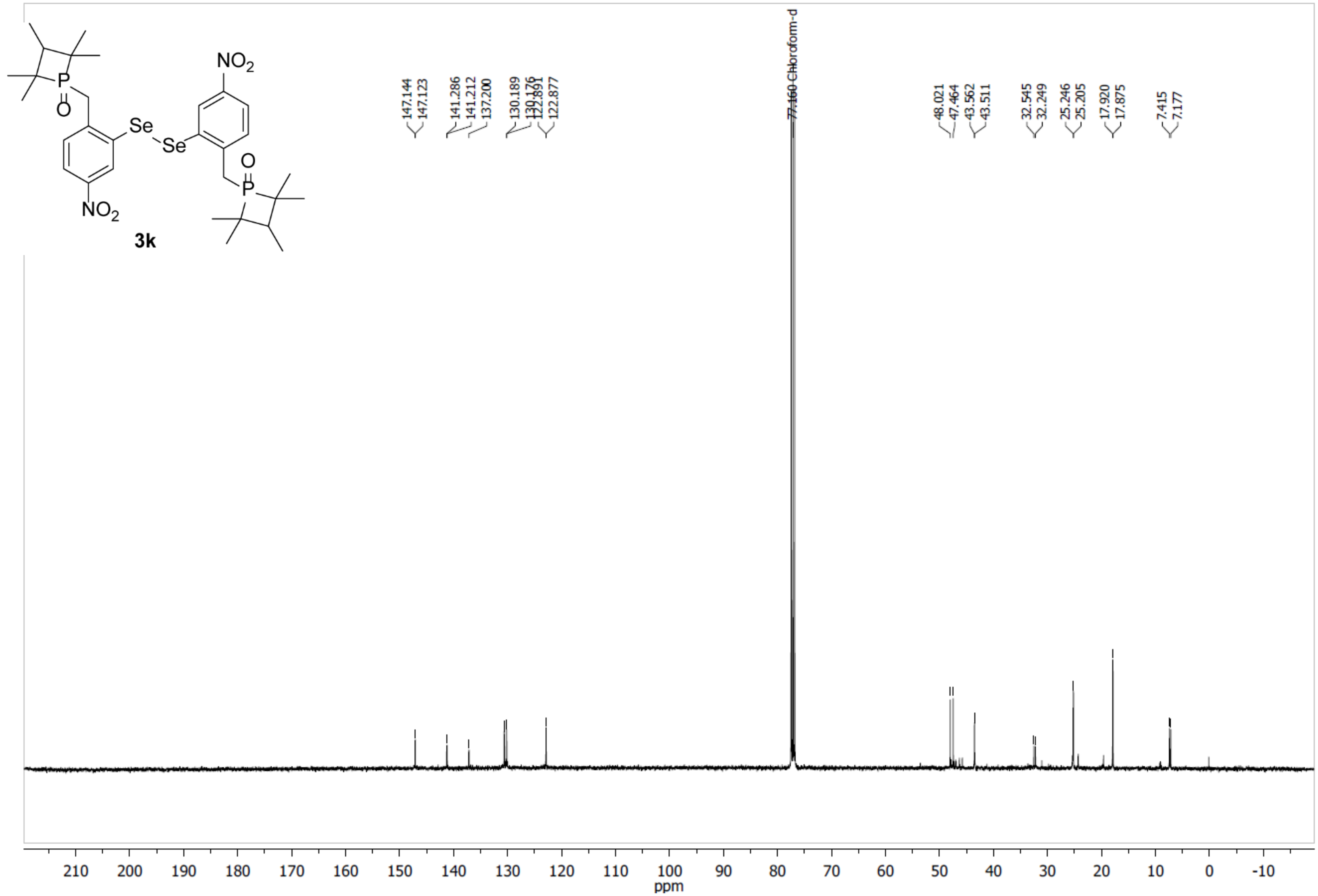

^{31}P NMR in $CDCl_3$

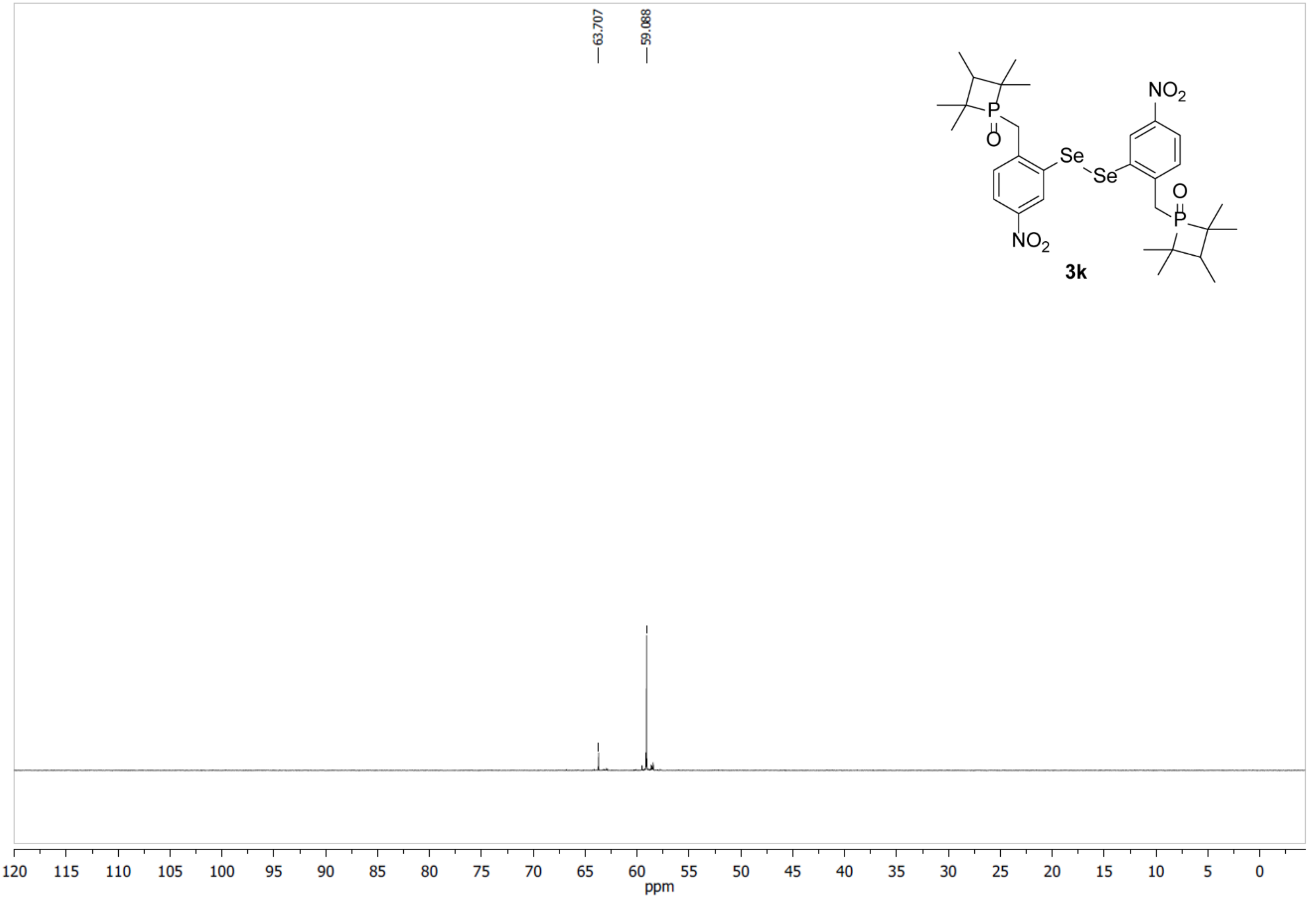

3k

^{77}Se NMR in $CDCl_3$

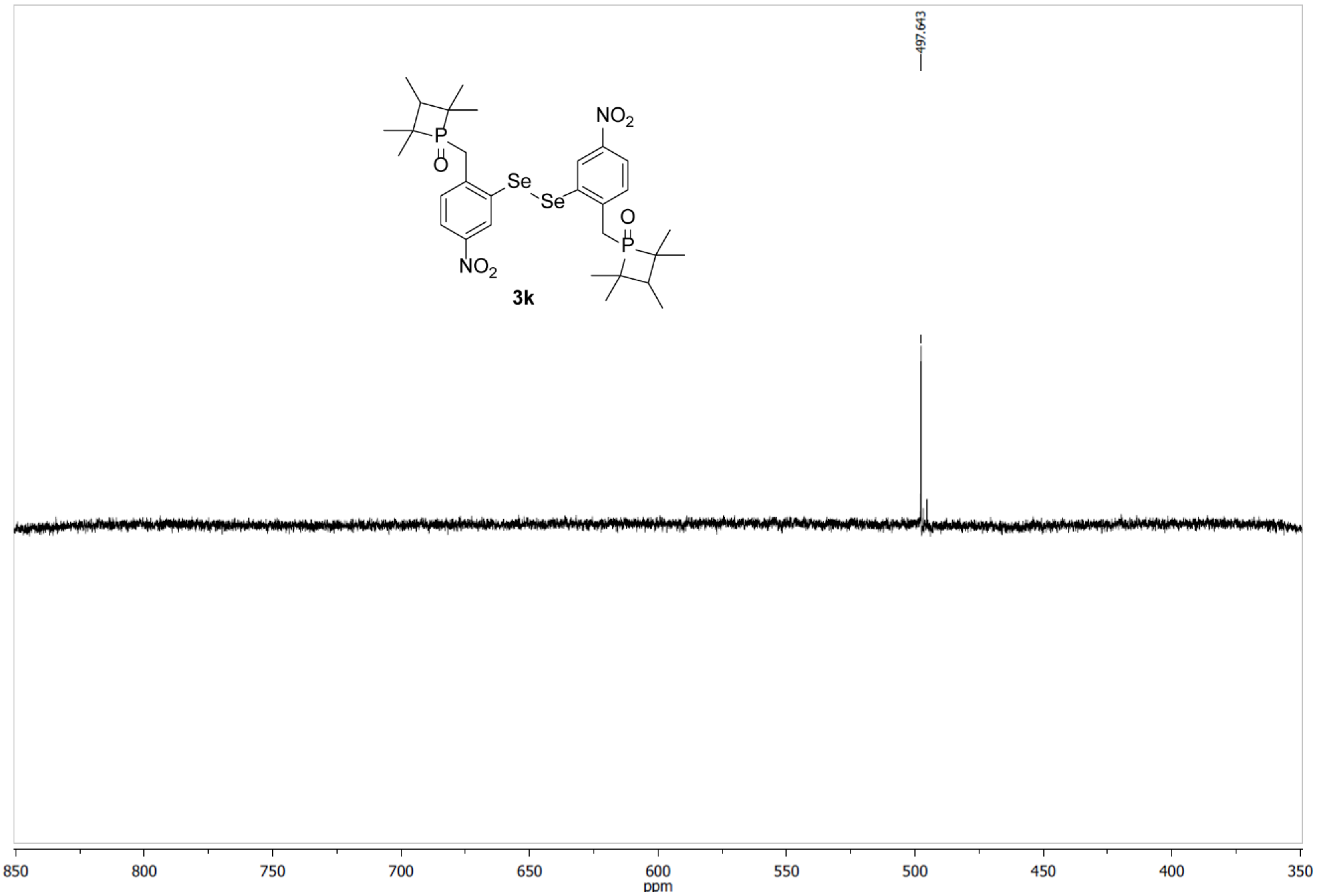

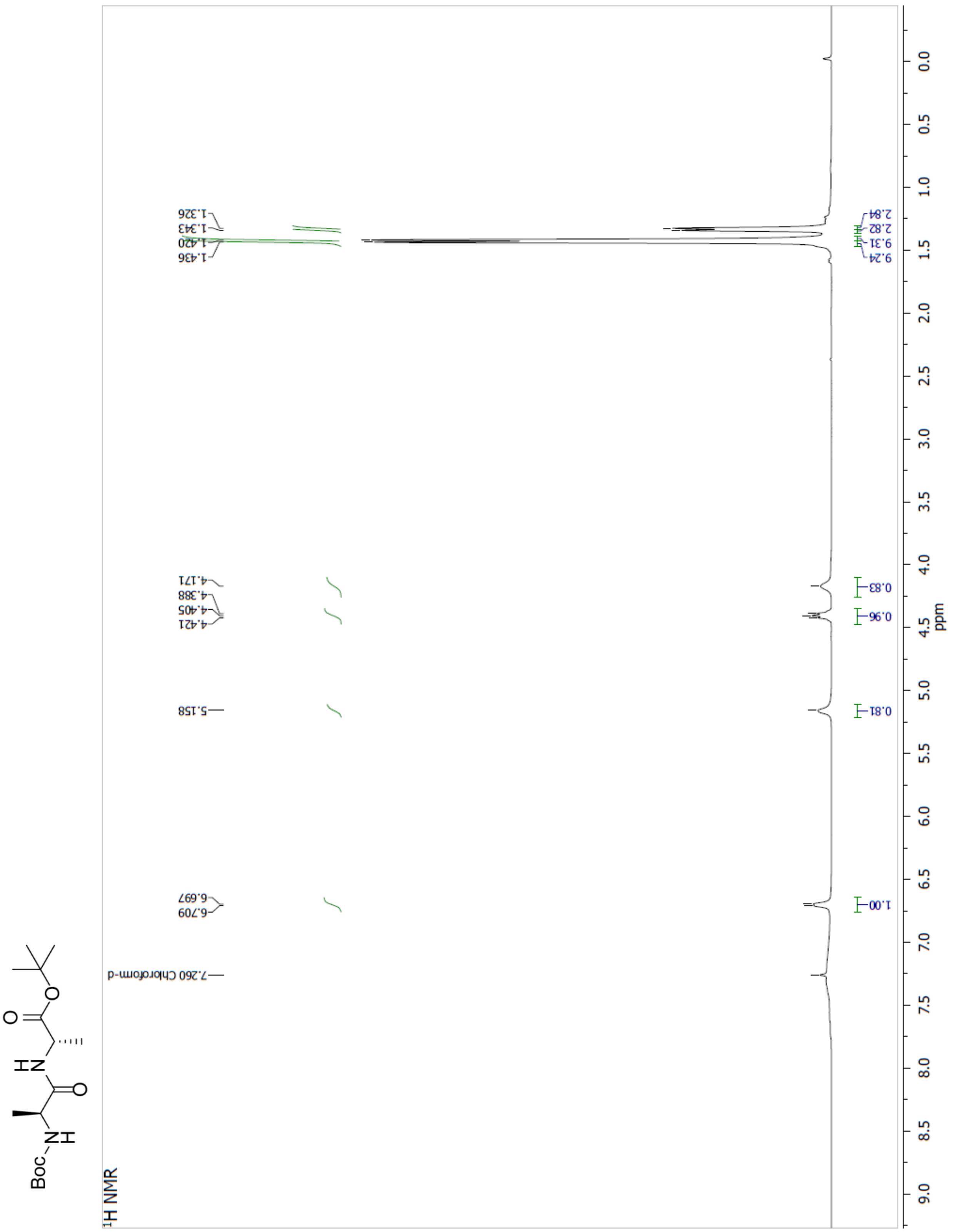
1H NMR
Boc
N
H
O
H
N
O
O
7.260 Chloroform-d
6.709
6.697
5.158
4.421
4.405
4.388
4.171
1.436
1.420
1.343
1.326
1.00
0.81
0.96
0.83
9.24
9.31
2.82
2.84
ppm

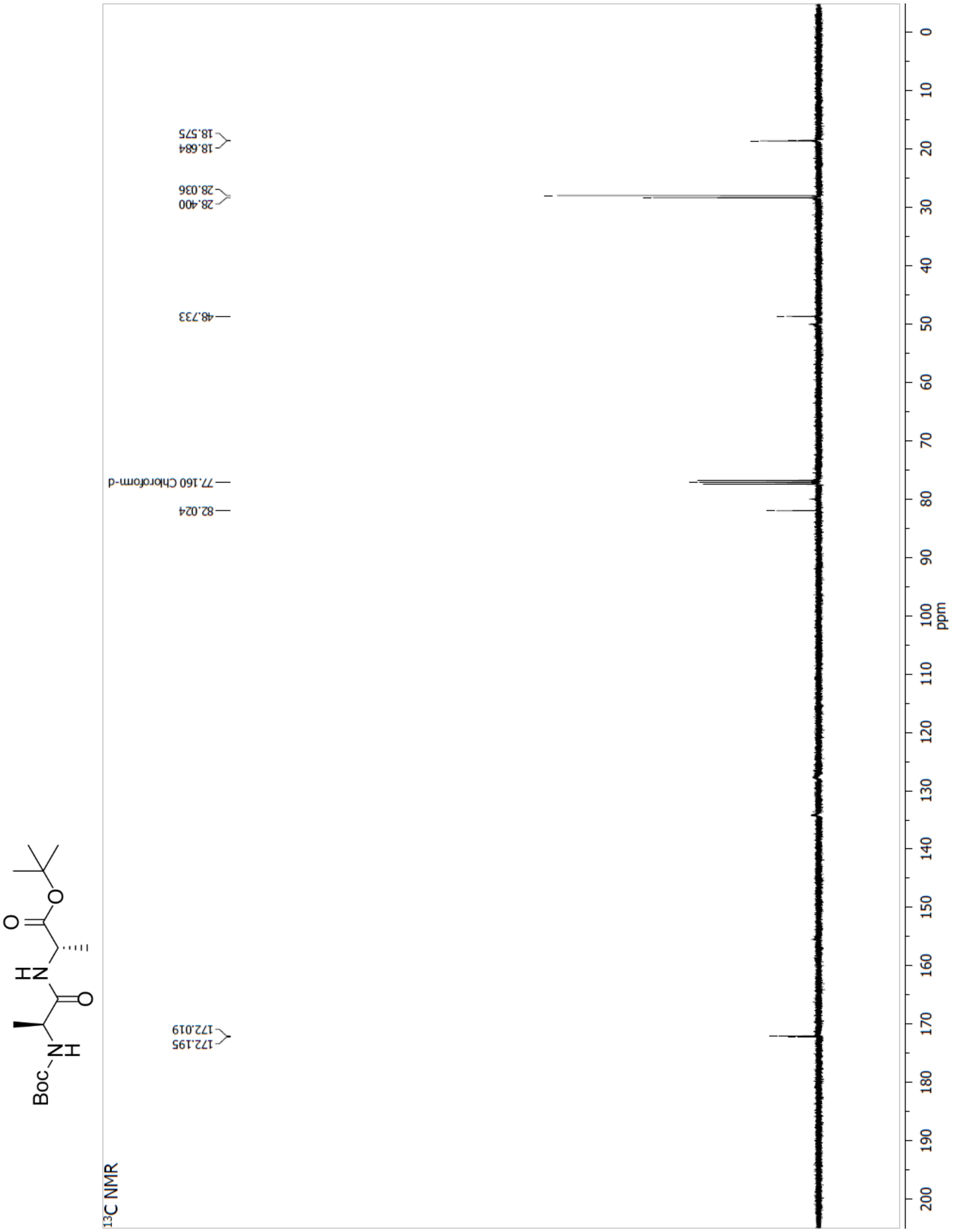
13C NMR
Boc
N
H
O
18.575
18.684
28.036
28.400
48.733
77.160 Chloroform-d
82.024
172.019
172.195
ppm
0
10
20
30
40
50
60
70
80
90
100
110
120
130
140
150
160
170
180
190
200

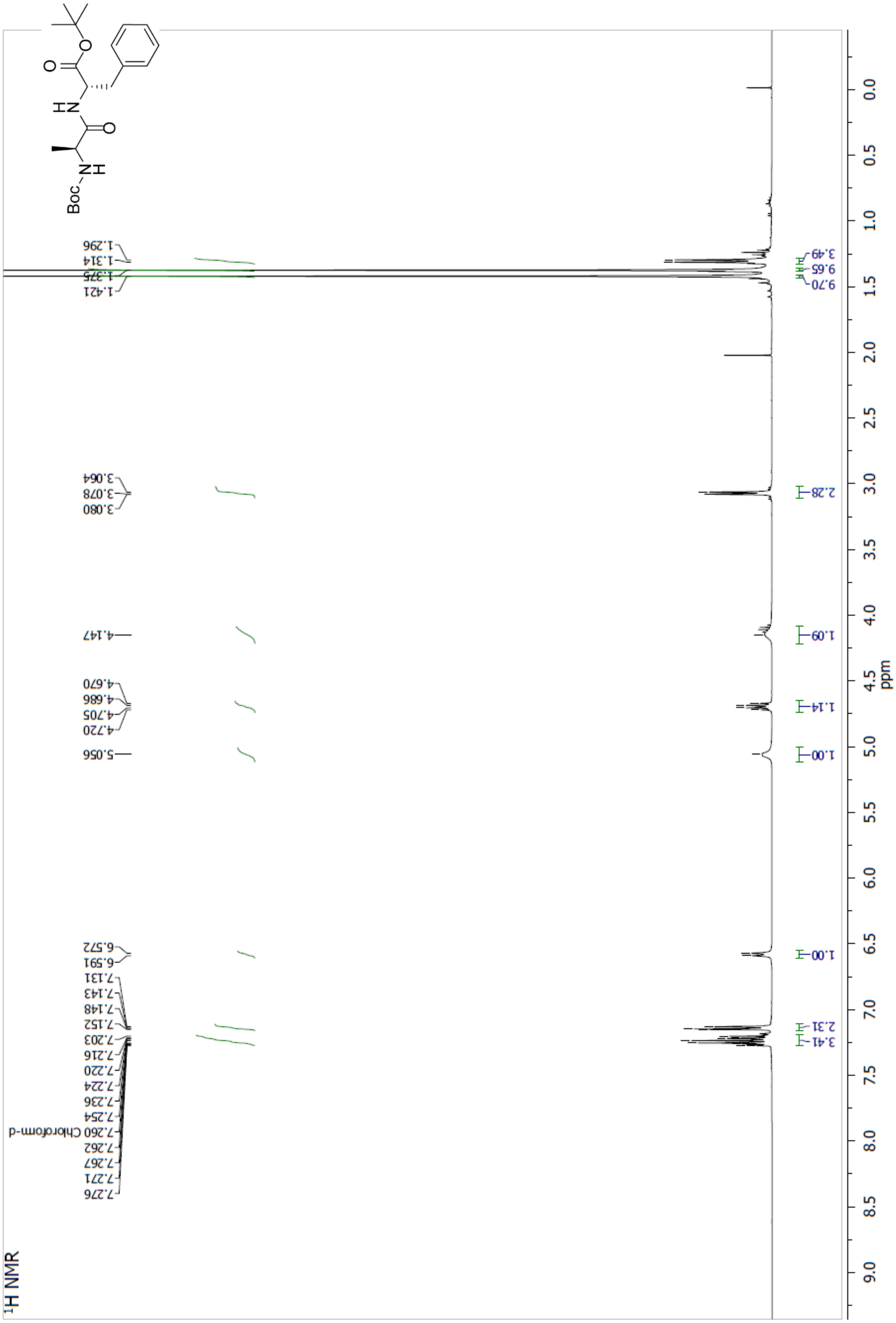
1H NMR
ppm
Boc
NH
O
Chlorofom-d
1.296
1.314
1.375
1.421
3.064
3.078
3.080
4.147
4.670
4.686
4.705
4.720
5.056
6.572
6.591
7.131
7.143
7.148
7.152
7.203
7.216
7.220
7.224
7.236
7.254
7.260
7.262
7.267
7.271
7.276
3.49
9.65
9.70
2.28
1.09
1.14
1.00
1.00
2.31
3.41

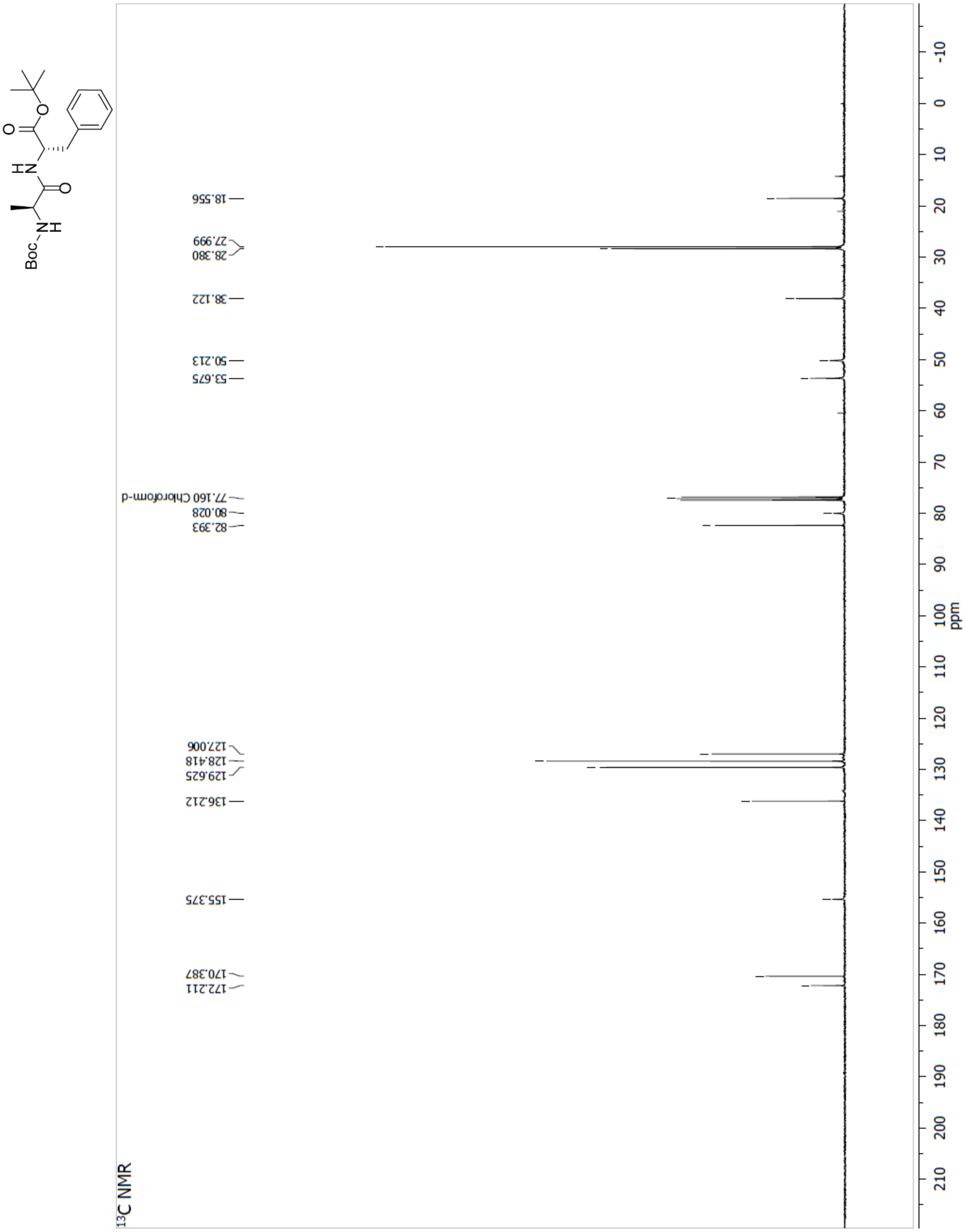

13C NMR
18.556
27.999
28.380
38.122
50.213
53.675
77.160 Chloroform-d
80.028
82.393
127.006
128.418
129.625
136.212
155.375
170.387
172.211
ppm
Boc

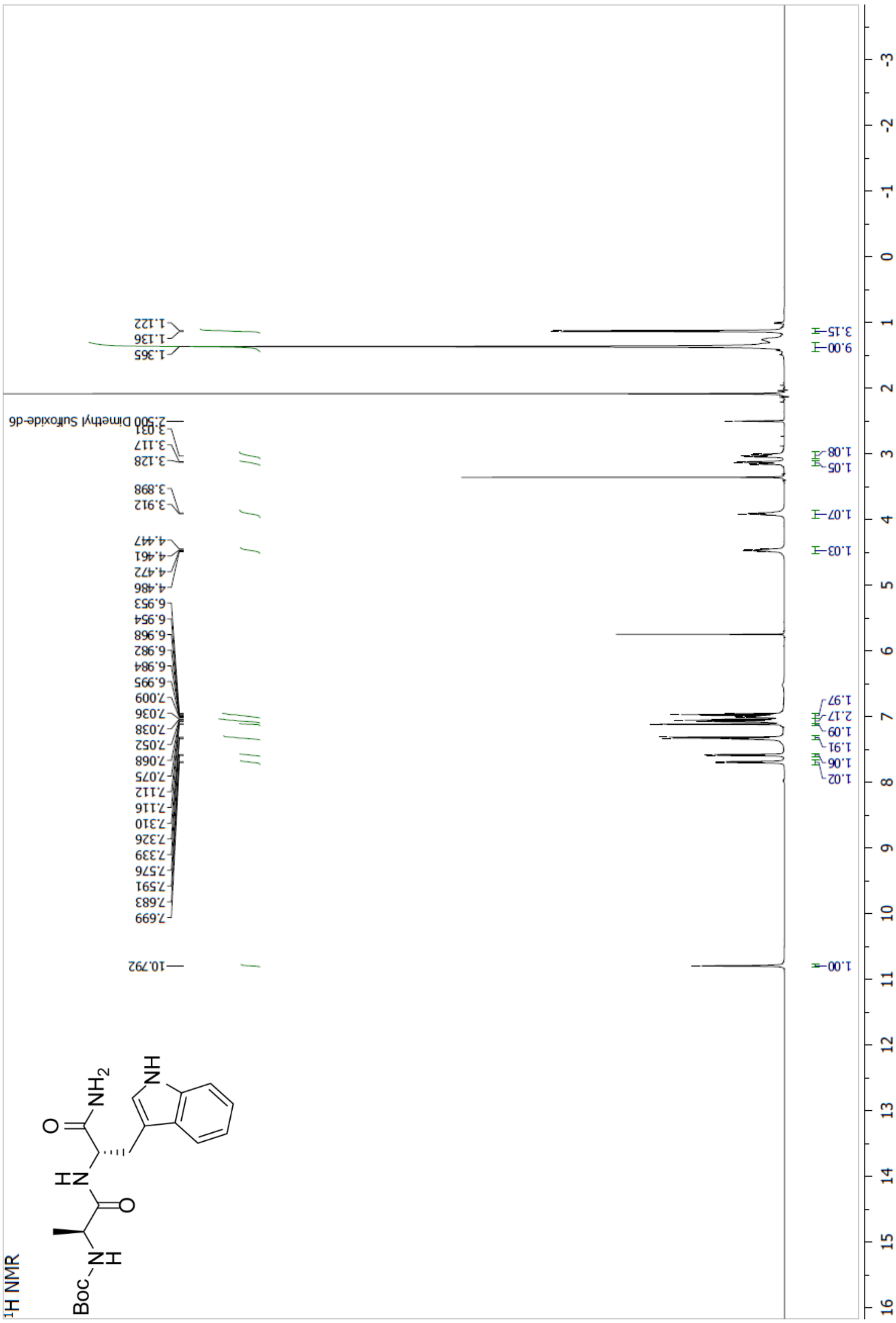
1H NMR
Boc
NH2
NH
2.500 Dimethyl Sulfoxide-d6
1.122
1.136
1.365
3.031
3.117
3.128
3.898
3.912
4.447
4.461
4.472
4.486
6.953
6.954
6.968
6.982
6.984
6.995
7.009
7.036
7.038
7.052
7.068
7.075
7.112
7.116
7.310
7.326
7.339
7.576
7.591
7.683
7.699
10.792
3.15
9.00
1.08
1.05
1.07
1.03
1.97
2.17
1.09
1.91
1.06
1.02
1.00
ppm

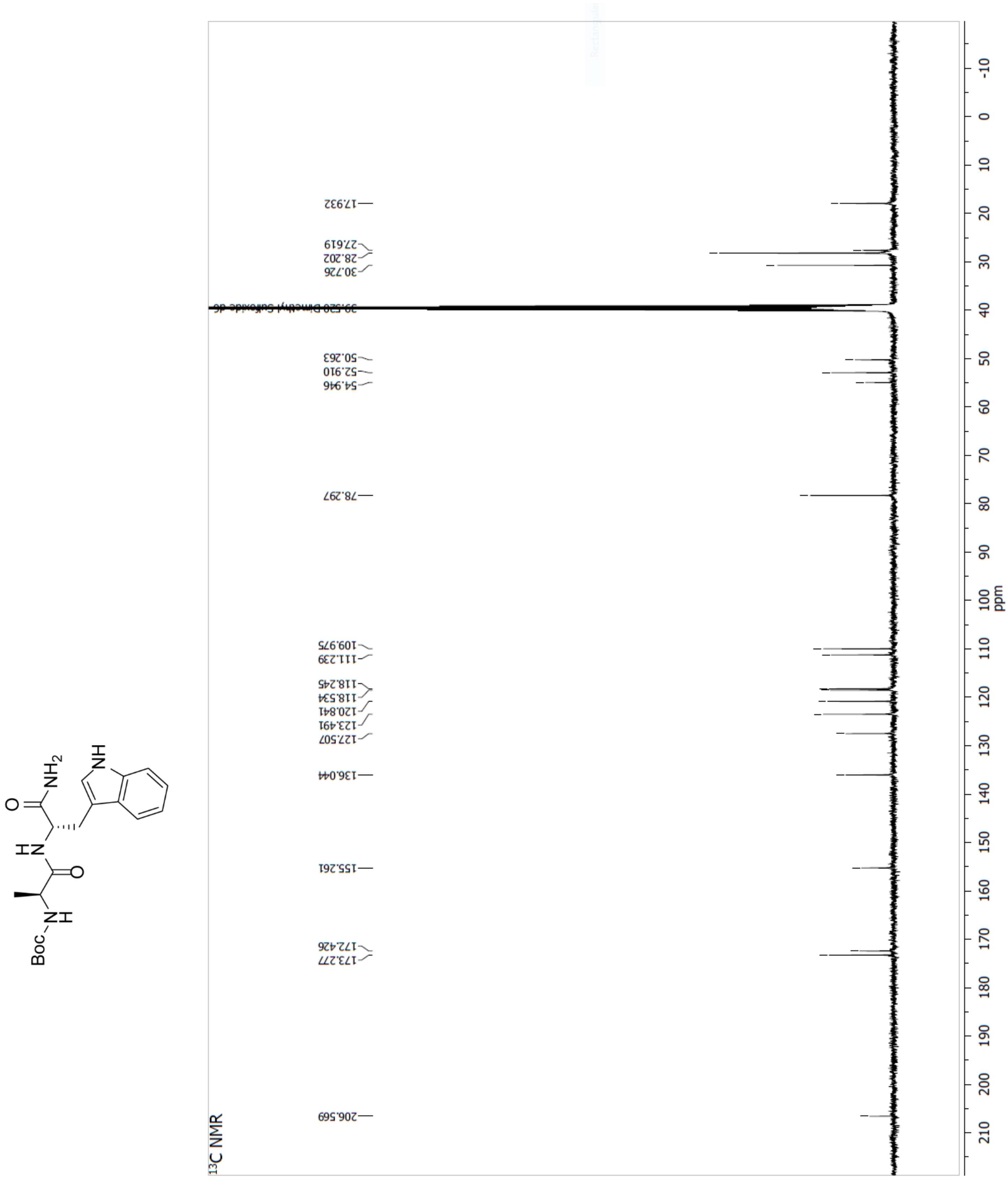
13C NMR
17.932
27.619
28.202
30.726
39.520 Dimethyl Sulfoxide-d6
50.263
52.910
54.946
78.297
109.975
111.239
118.245
118.534
120.841
123.491
127.507
136.044
155.261
172.426
173.277
206.569
ppm
Boc
NH
NH2
O
NH

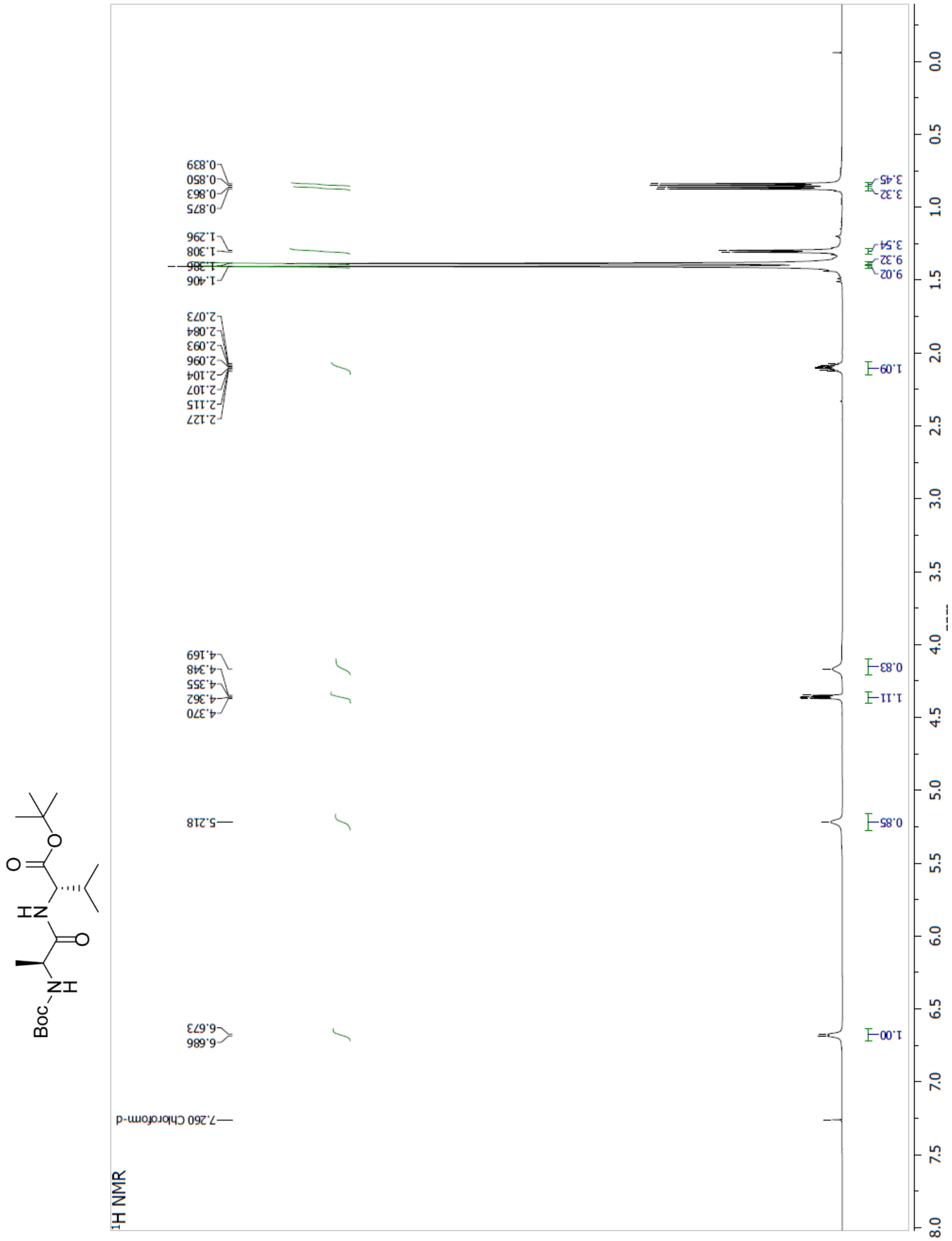
1H NMR
Boc
N
H
O
0.839
0.850
0.863
0.875
1.296
1.308
1.386
1.406
2.073
2.084
2.093
2.096
2.104
2.107
2.115
2.127
4.169
4.348
4.355
4.362
4.370
5.218
6.673
6.686
7.260 Chloroform-d
3.45
3.32
3.54
9.32
9.02
1.09
0.83
1.11
0.85
1.00
0.0
0.5
1.0
1.5
2.0
2.5
3.0
3.5
4.0
4.5
5.0
5.5
6.0
6.5
7.0
7.5
8.0

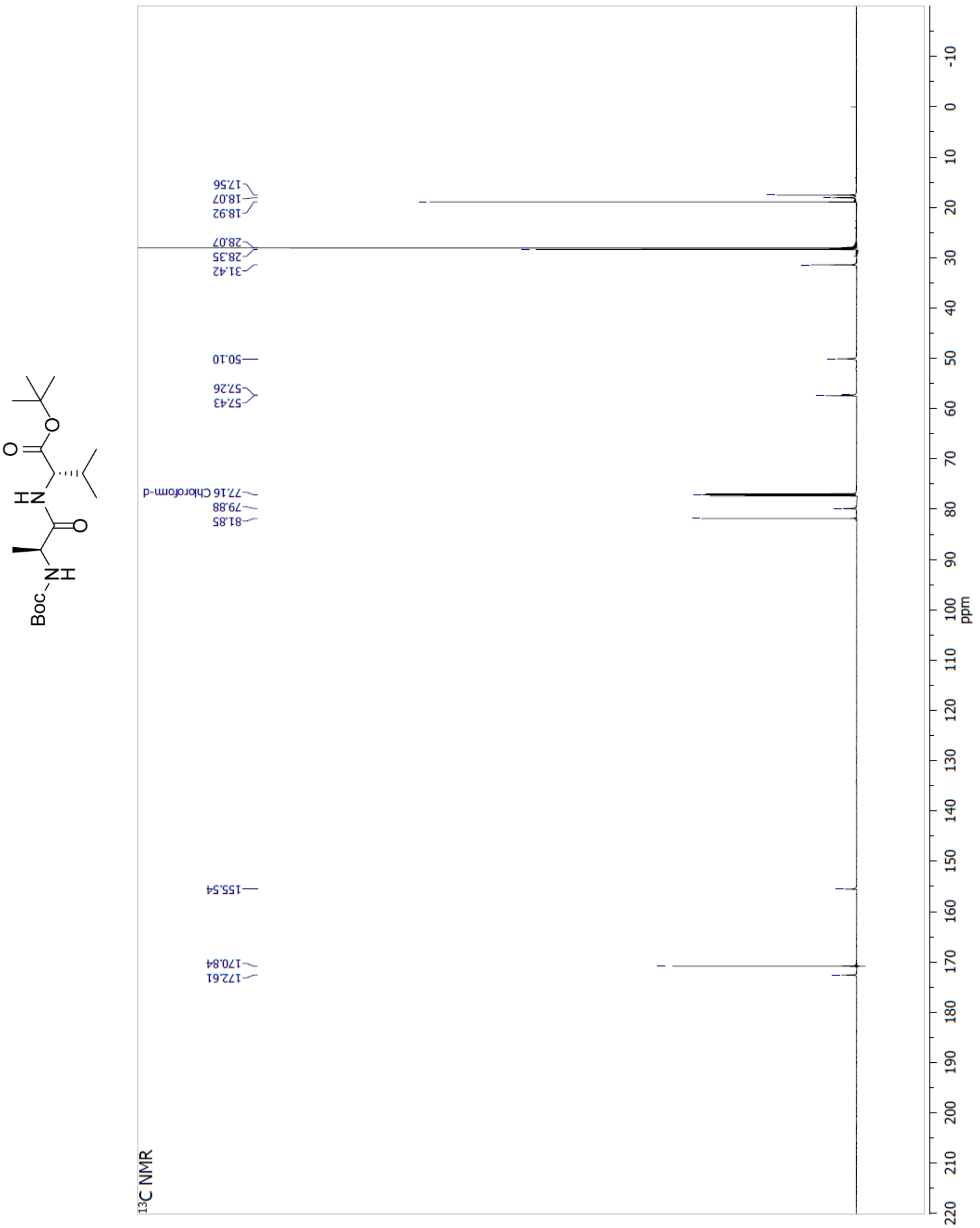

Boc
N
H
O
N
H
O
O
13C NMR
17.56
18.07
18.92
28.07
28.35
31.42
50.10
57.26
57.43
77.16 Chloroform-d
79.88
81.85
155.54
170.84
172.61
ppm

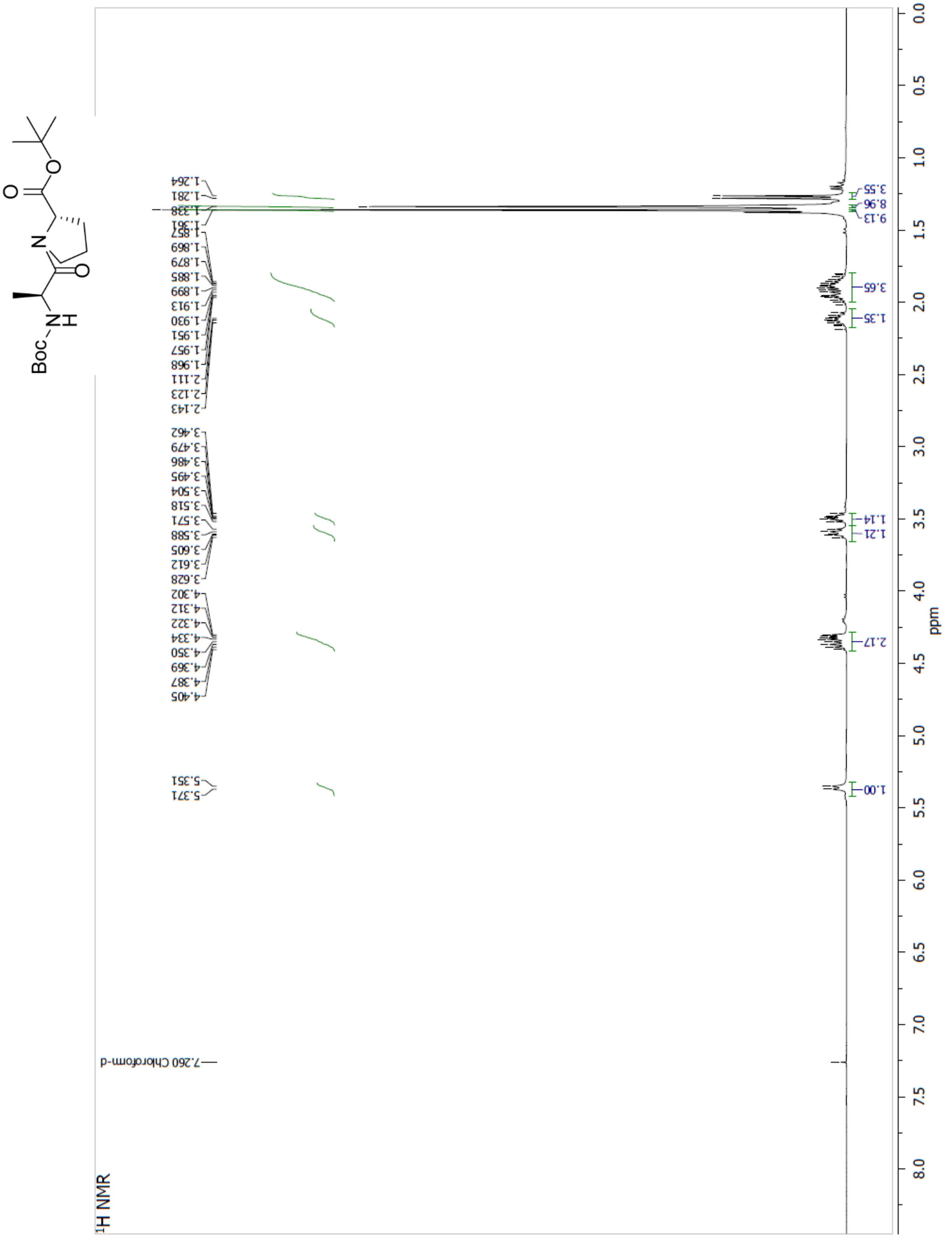

1H NMR
7.260 Chloroform-d
ppm

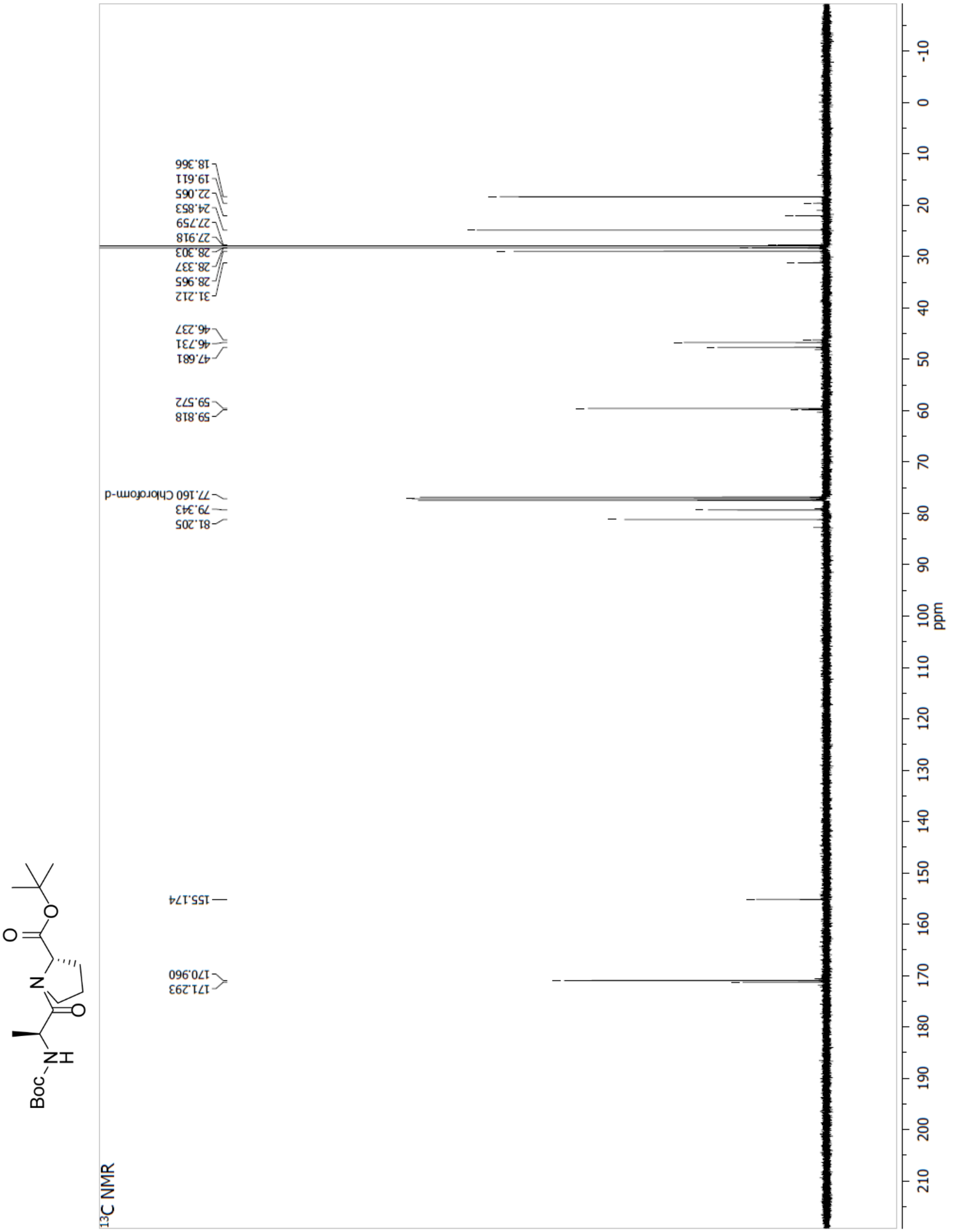
13C NMR
Boc
N
H
O
N
O
O
18.366
19.611
22.065
24.853
27.759
27.918
28.303
28.337
28.965
31.212
46.237
46.731
47.681
59.572
59.818
77.160 Chloroform-d
79.343
81.205
155.174
170.960
171.293
ppm

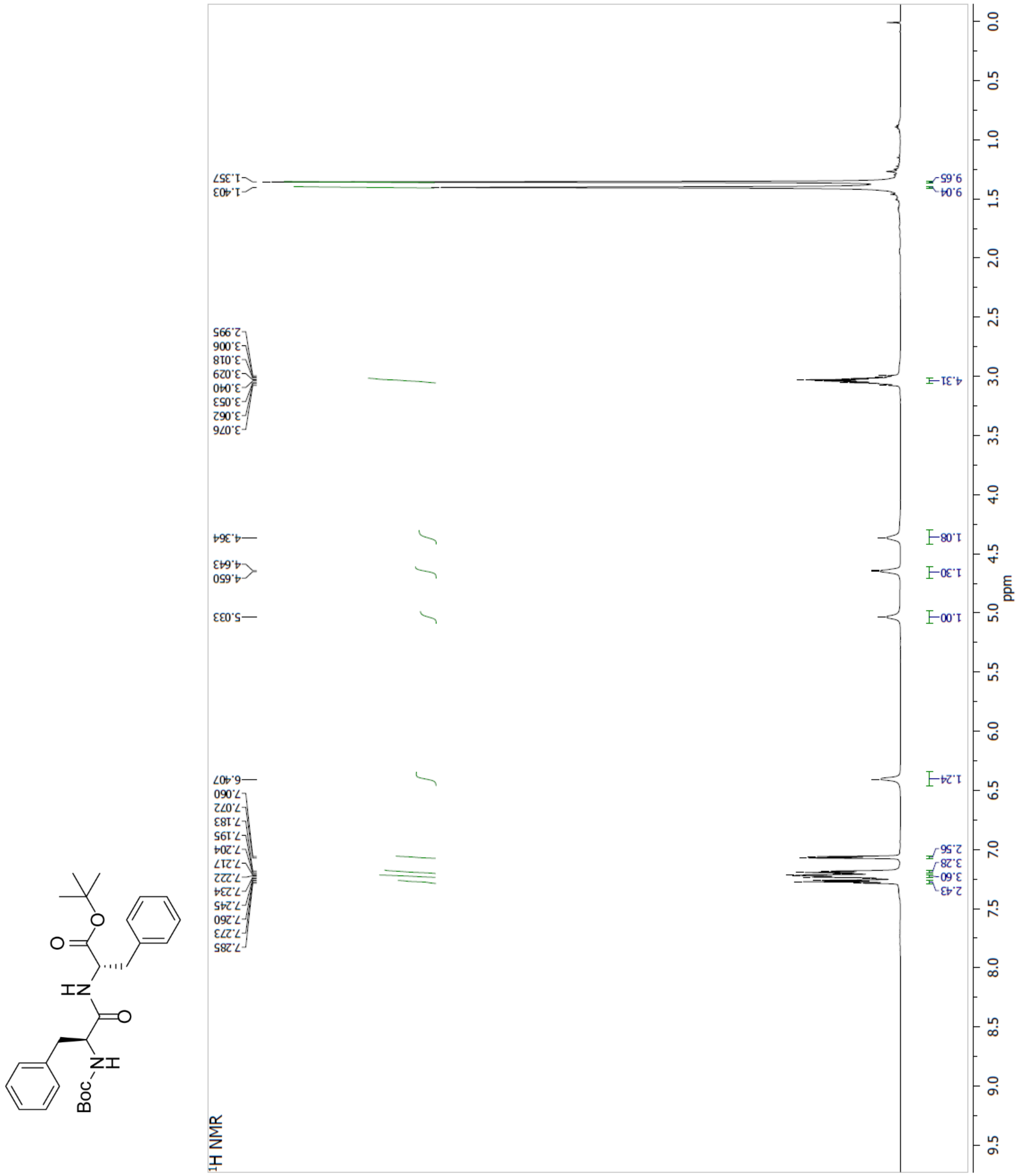
1H NMR
ppm
Boc
NH
HN
O
7.285
7.273
7.260
7.245
7.234
7.222
7.217
7.204
7.195
7.183
7.072
7.060
6.407
5.033
4.650
4.643
4.364
3.076
3.062
3.053
3.040
3.029
3.018
3.006
2.995
1.403
1.357
2.43
3.60
3.28
2.56
1.24
1.00
1.30
1.08
4.31
9.04
9.65
9.5
9.0
8.5
8.0
7.5
7.0
6.5
6.0
5.5
5.0
4.5
4.0
3.5
3.0
2.5
2.0
1.5
1.0
0.5
0.0

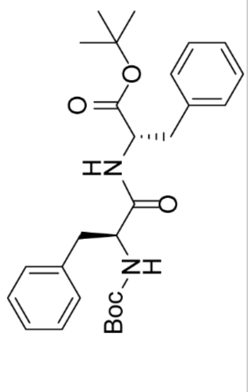

Boc
NH
O
NH
O
O

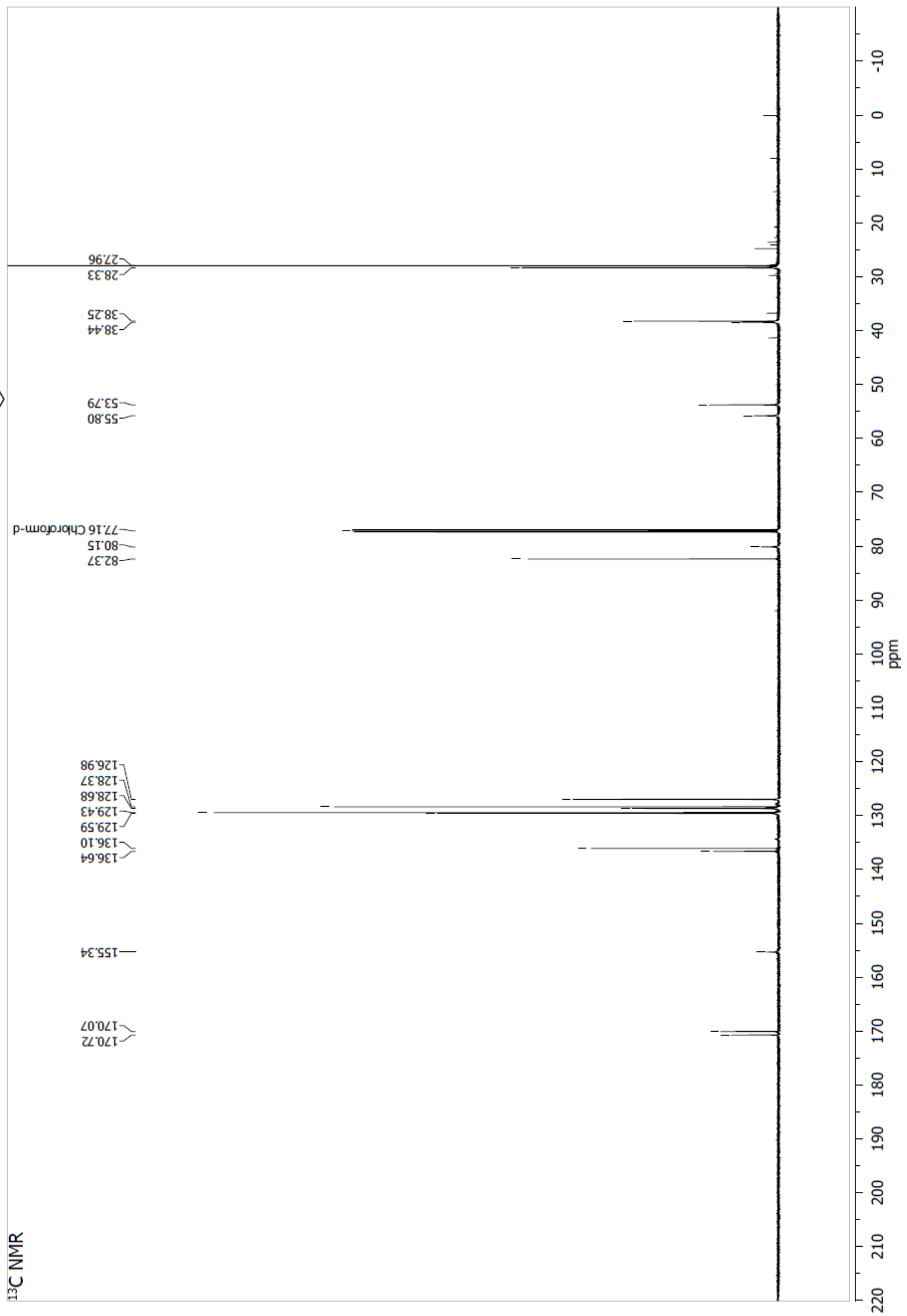

13C NMR
27.96
28.33
38.25
38.44
53.79
55.80
77.16 Chloroform-d
80.15
82.37
126.98
128.37
128.68
129.43
129.59
136.10
136.64
155.34
170.07
170.72
ppm

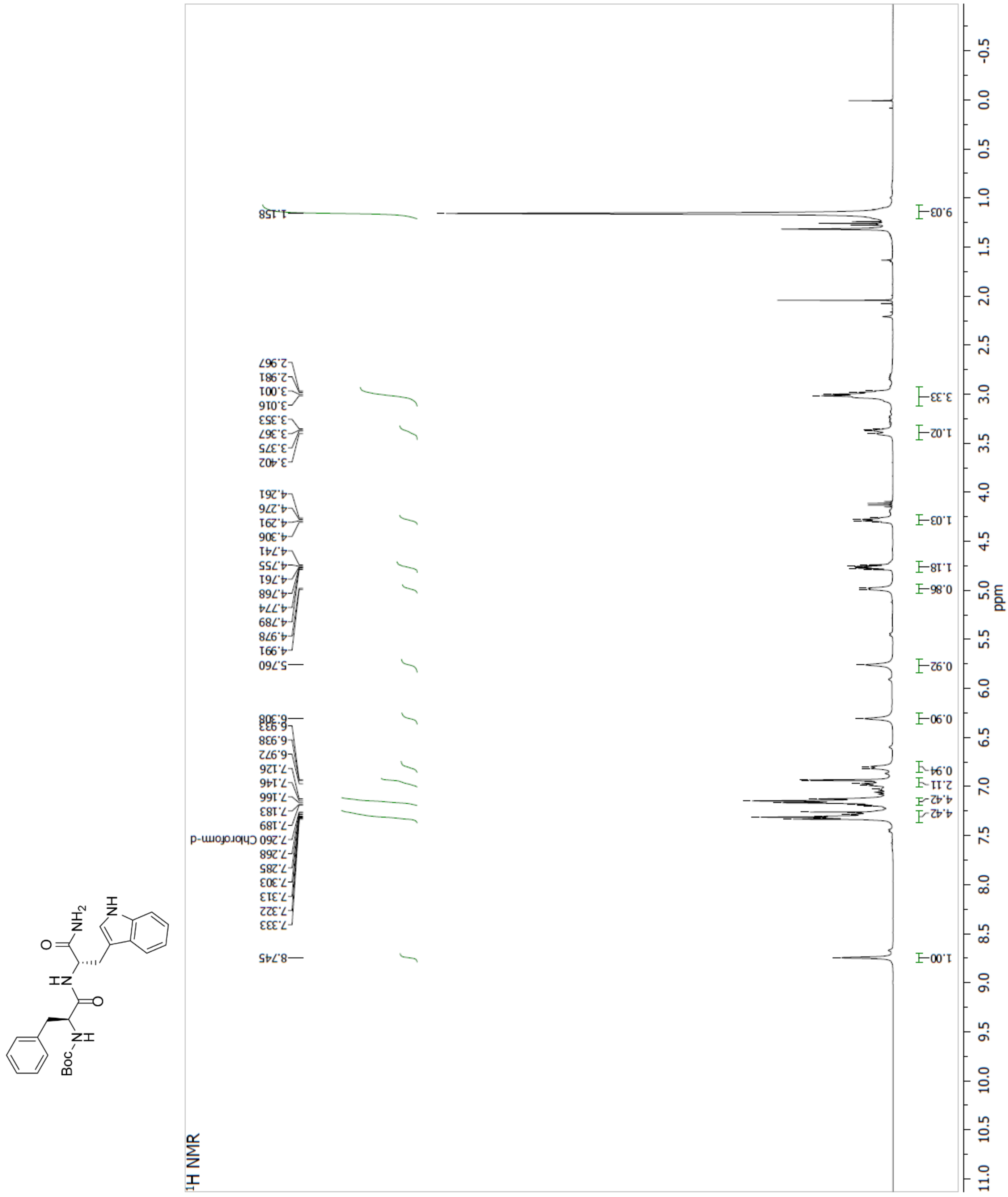
1H NMR
ppm

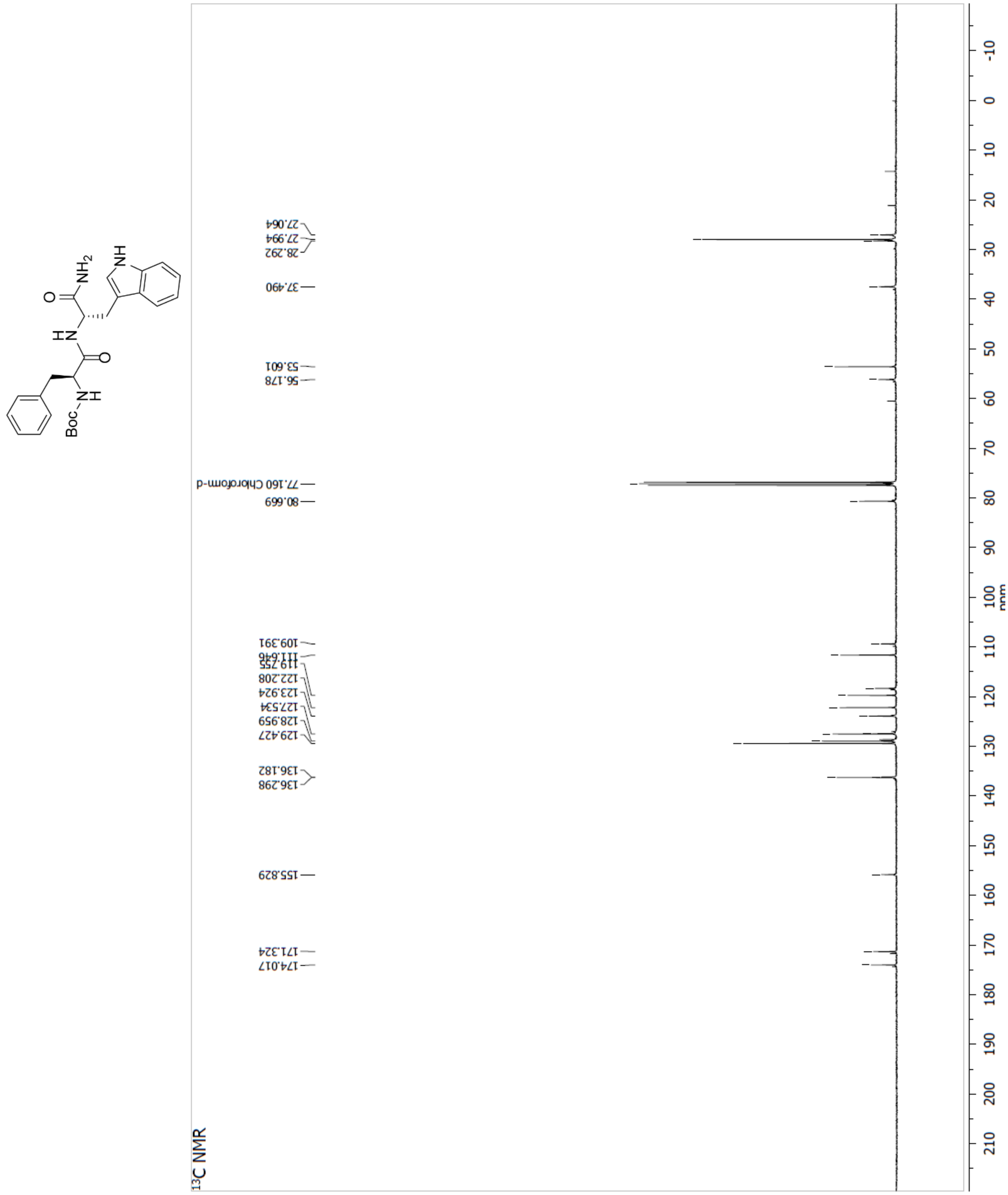

13C NMR
27.064
27.994
28.292
37.490
53.601
56.178
77.160 Chloroform-d
80.669
109.391
111.646
119.755
122.208
123.924
127.534
128.959
129.427
136.182
136.298
155.829
171.324
174.017
ppm
Boc
NH2
NH

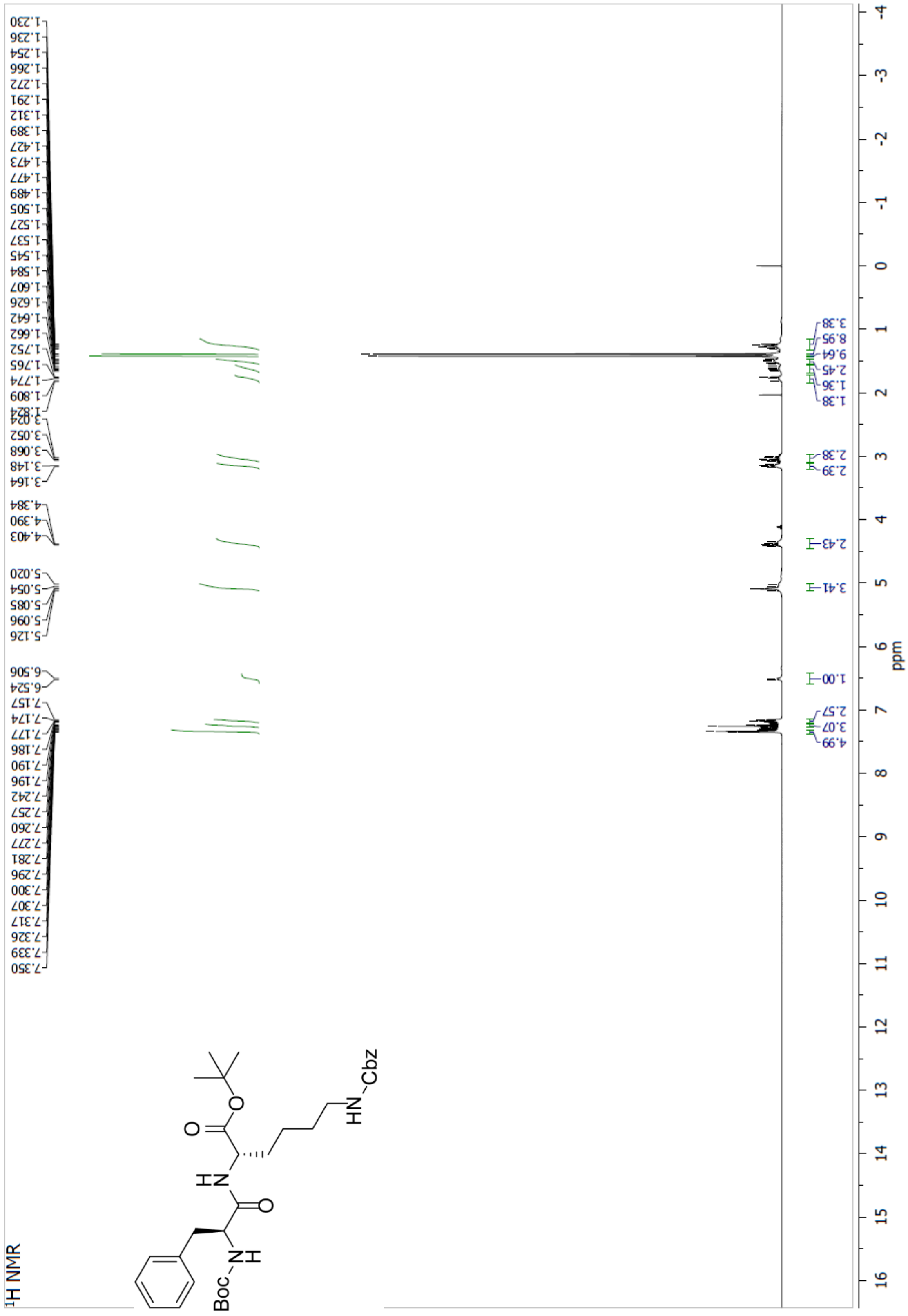
1H NMR
Boc
Cbz
ppm

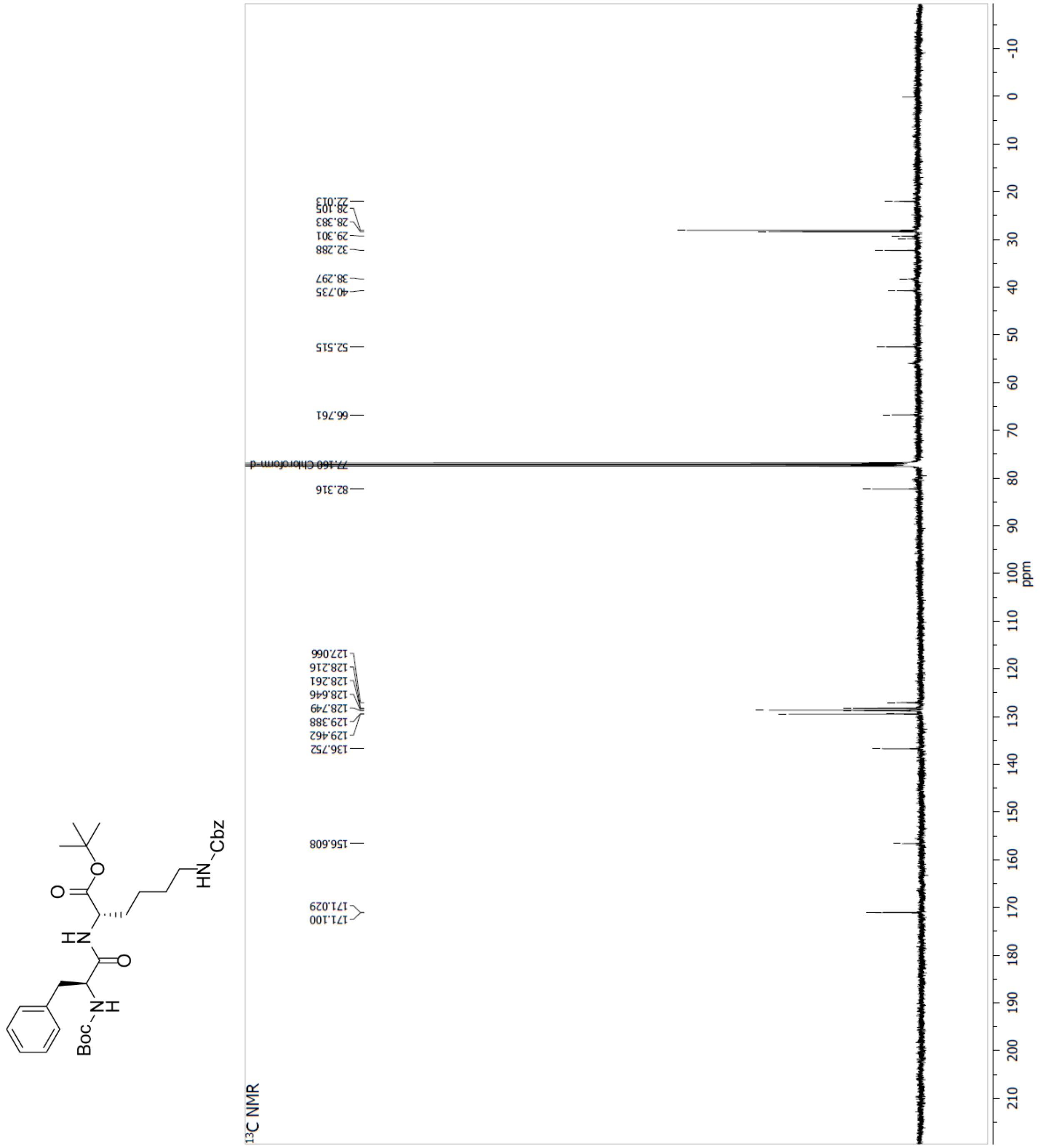
Boc
HN
Cbz
13C NMR
22.013
28.105
28.383
29.301
32.288
38.297
40.735
52.515
66.761
77.160 Chloroform-d
82.316
127.066
128.216
128.261
128.646
128.749
129.388
129.462
136.752
156.608
171.029
171.100
ppm

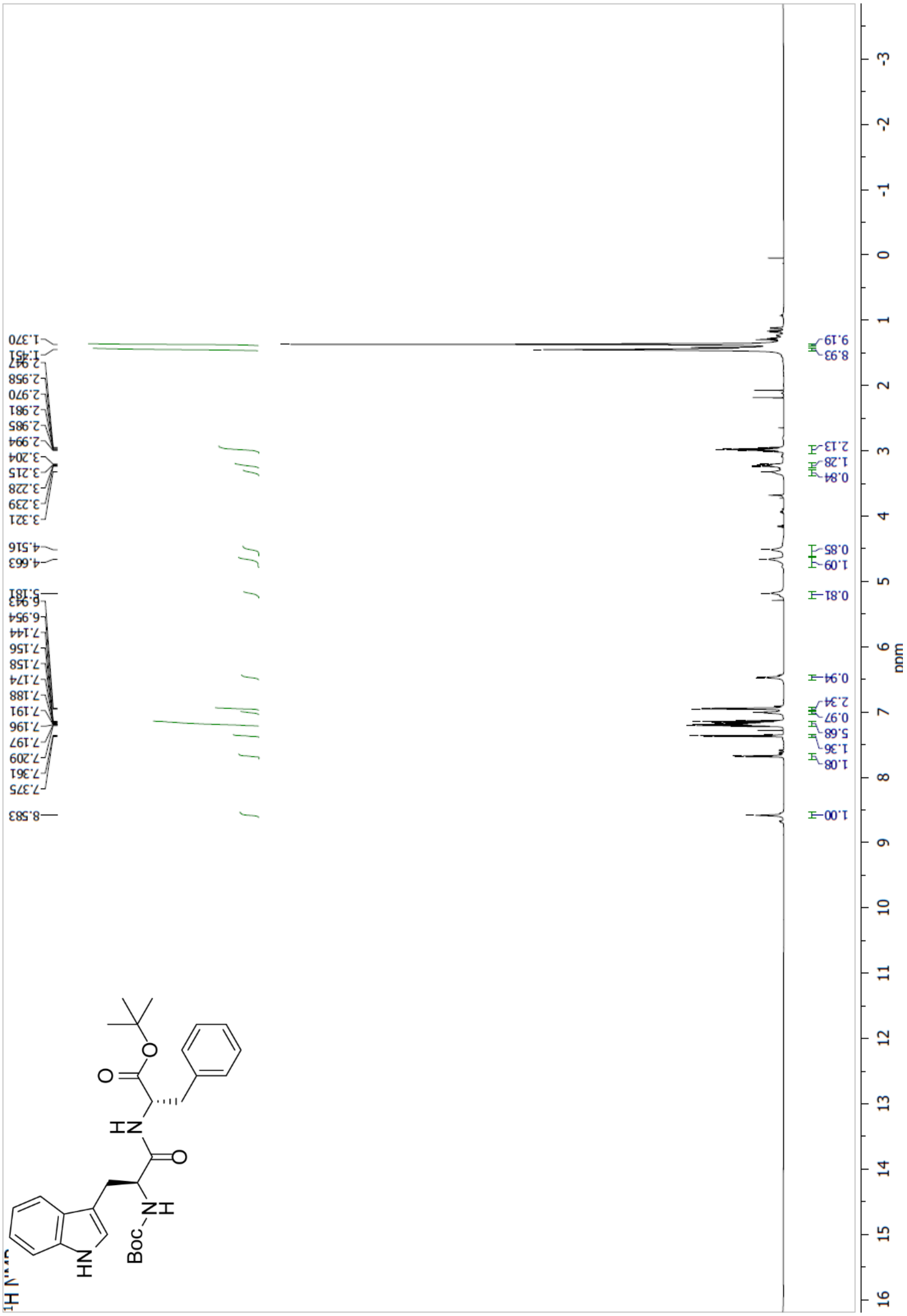
1H NMR
HN
Boc
NH
HN
O
O
O
1.370
1.451
2.947
2.958
2.970
2.981
2.985
2.994
3.204
3.215
3.228
3.239
3.321
4.516
4.663
5.181
6.943
6.954
7.144
7.156
7.158
7.174
7.188
7.191
7.196
7.197
7.209
7.361
7.375
8.583
9.19
8.93
2.13
1.28
0.84
0.85
1.09
0.81
0.94
2.34
0.97
5.68
1.36
1.08
1.00
ppm

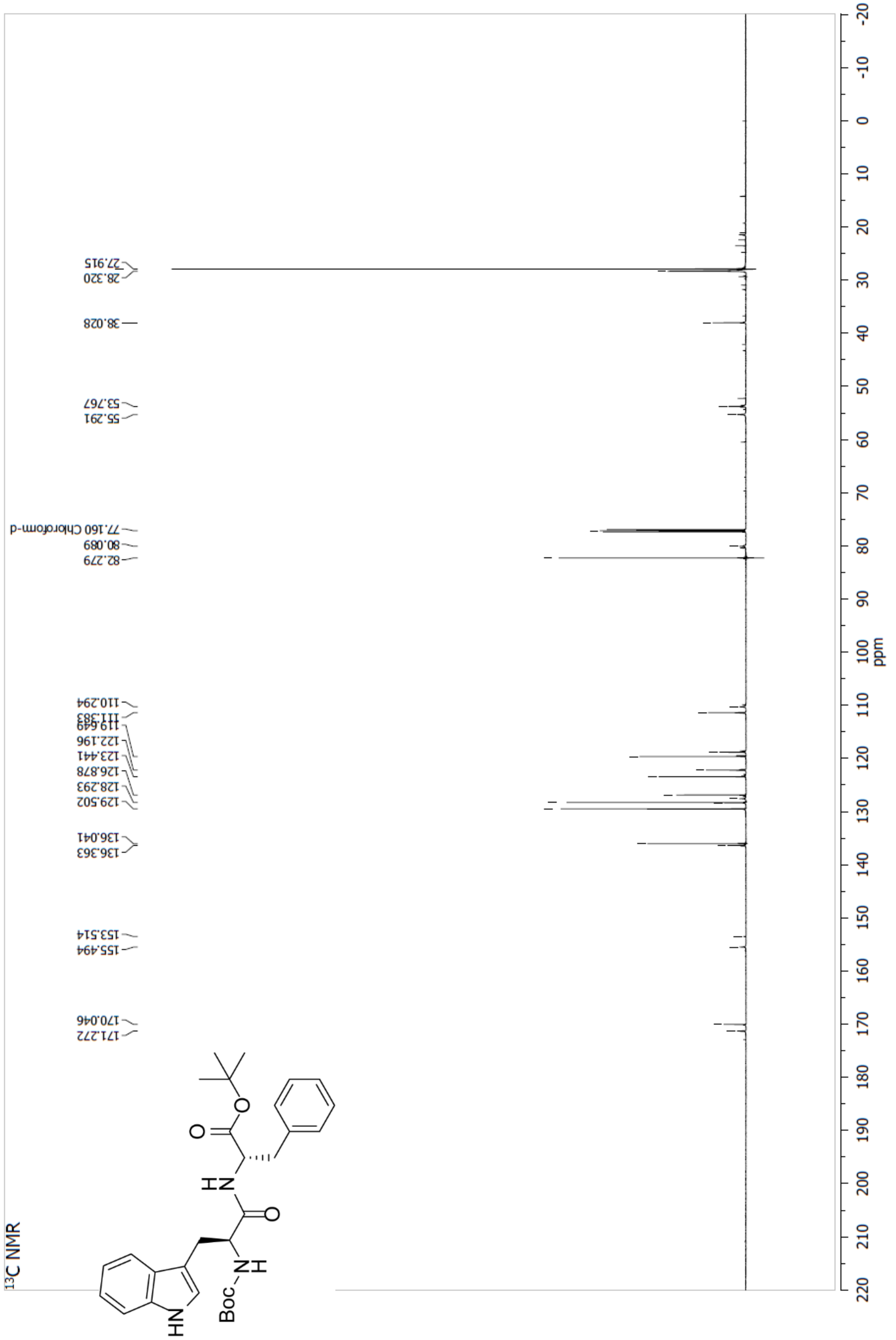
13C NMR
27.915
28.320
38.028
53.767
55.291
77.160 Chloroform-d
80.089
82.279
110.294
111.383
119.649
122.196
123.441
126.878
128.293
129.502
136.041
136.363
153.514
155.494
170.046
171.272
Boc
HN
ppm

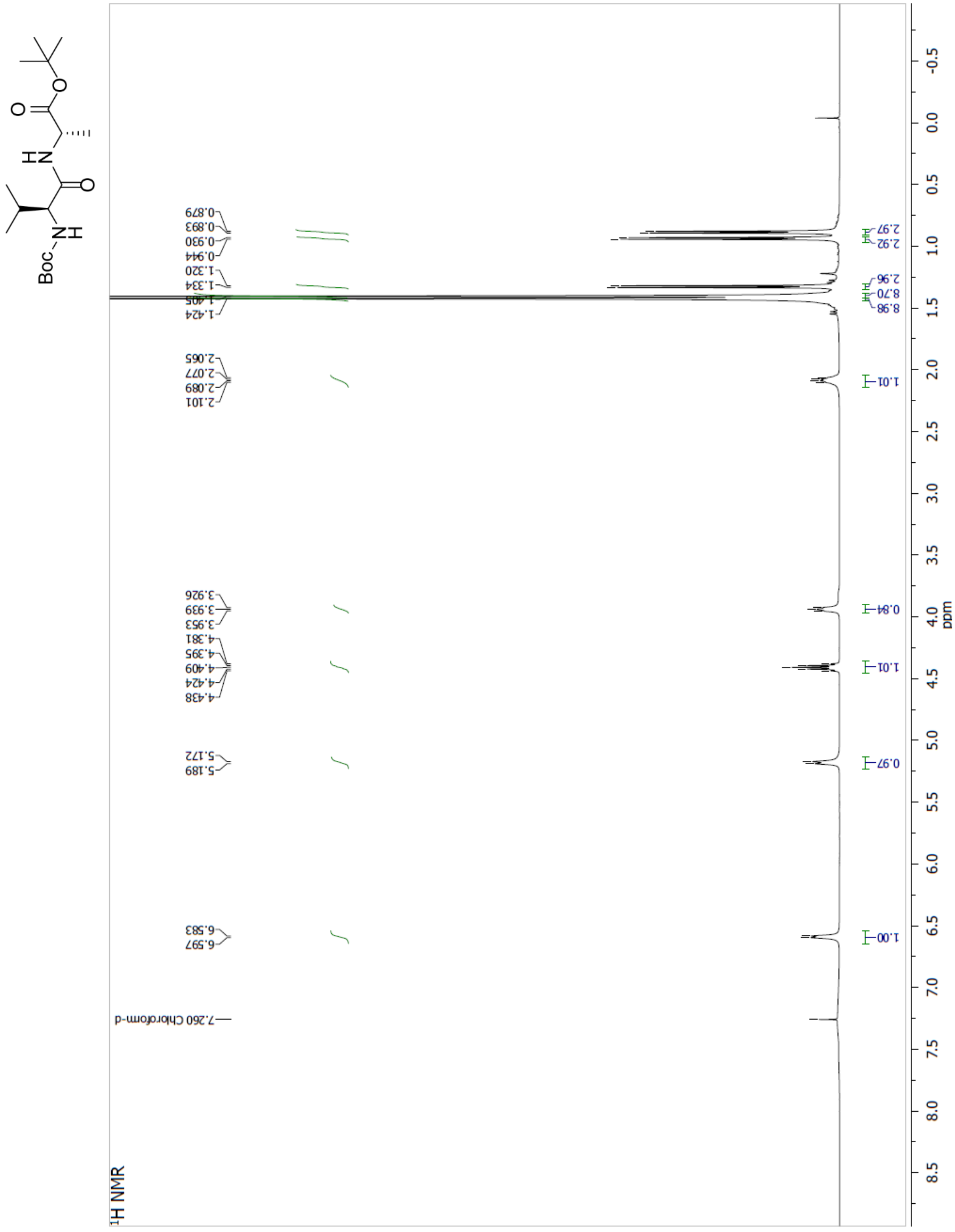
Boc
1H NMR
0.879
0.893
0.930
0.944
1.320
1.334
1.405
1.424
2.065
2.077
2.089
2.101
3.926
3.939
3.953
4.381
4.395
4.409
4.424
4.438
5.172
5.189
6.583
6.597
7.260 Chloroform-d
2.97
2.92
2.96
8.70
8.98
1.01
0.84
1.01
0.97
1.00
ppm

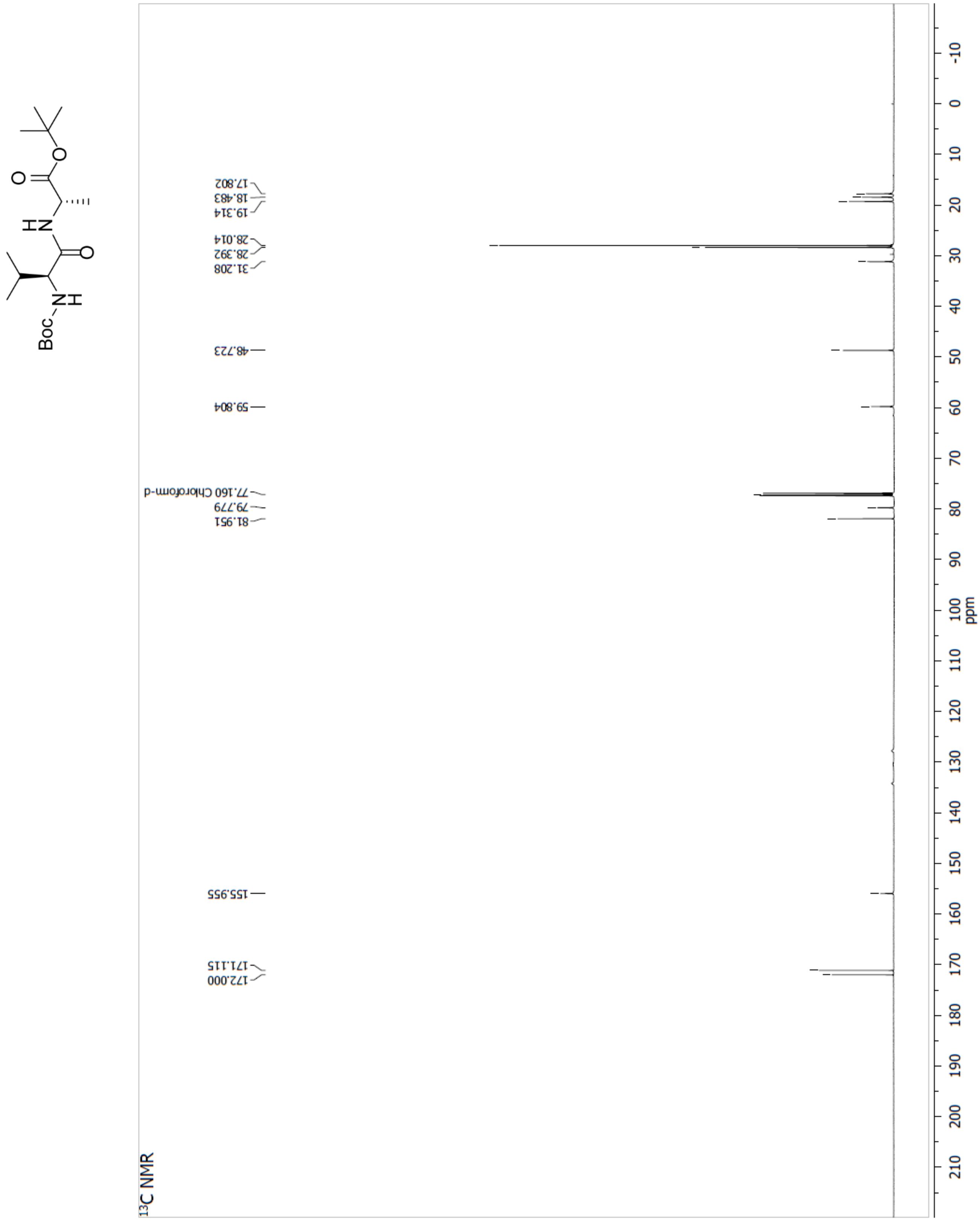
13C NMR
17.802
18.483
19.314
28.014
28.392
31.208
48.723
59.804
77.160 Chloroform-d
79.779
81.951
155.955
171.115
172.000
ppm
Boc
N
H
O

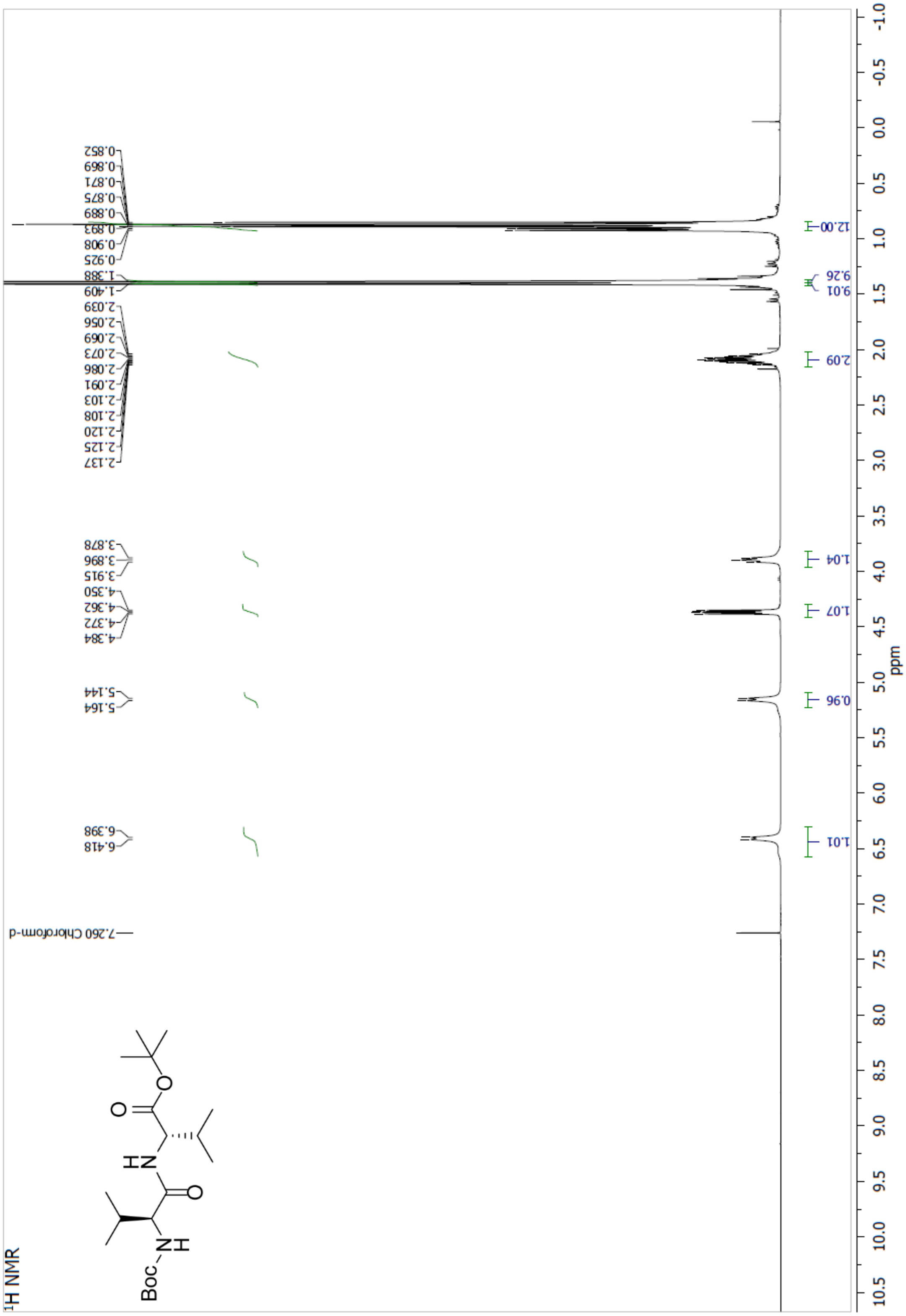

1H NMR
Boc
NH
O
7.260 Chloroform-d
0.852
0.869
0.871
0.875
0.889
0.893
0.908
0.925
1.388
1.409
2.039
2.056
2.069
2.073
2.086
2.091
2.103
2.108
2.120
2.125
2.137
3.878
3.896
3.915
4.350
4.362
4.372
4.384
5.144
5.164
6.398
6.418
12.00
9.26
9.01
2.09
1.04
1.07
0.96
1.01
ppm

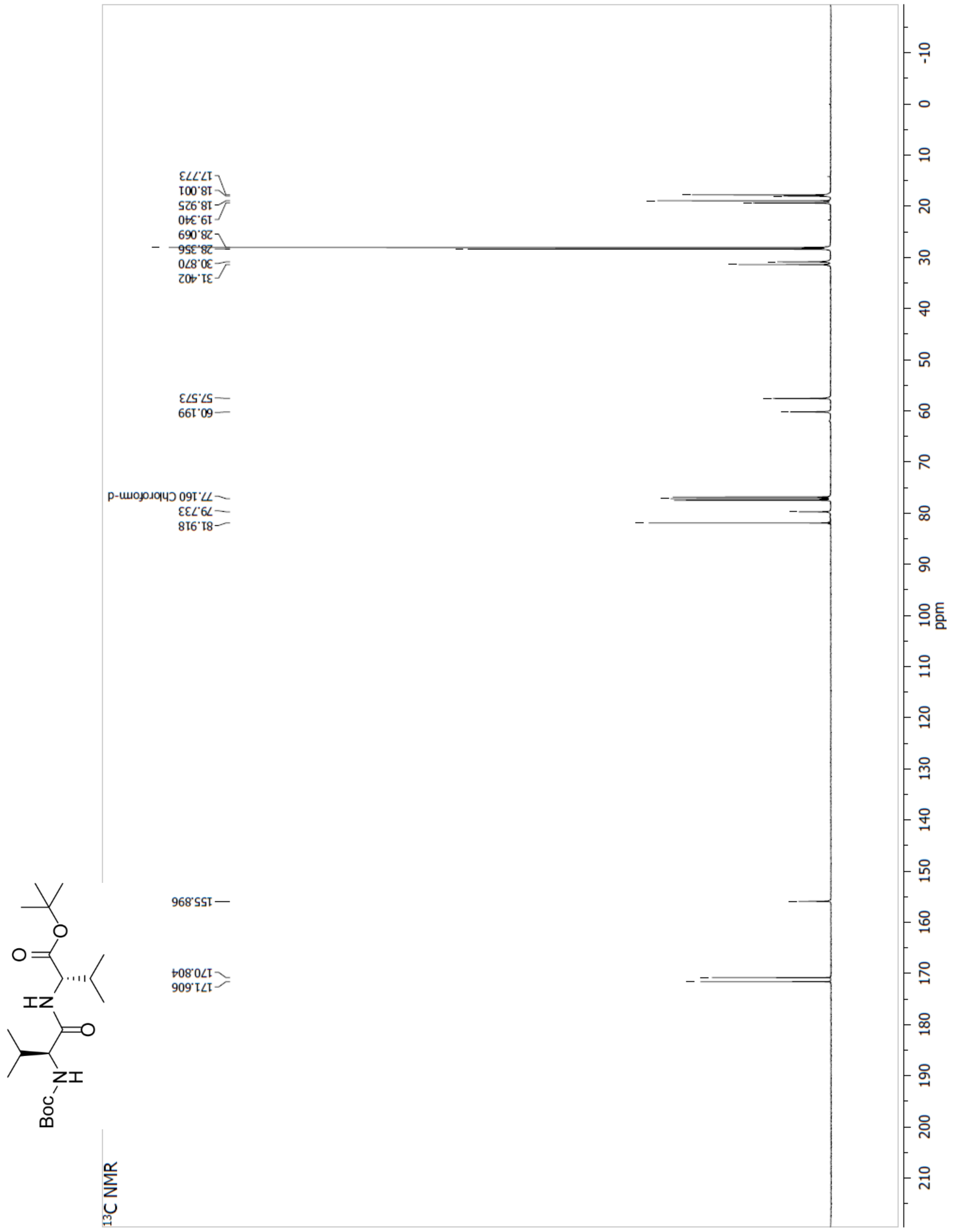

13C NMR
17.773
18.001
18.925
19.340
28.069
28.356
30.870
31.402
57.573
60.199
77.160 Chlorofom-d
79.733
81.918
155.896
170.804
171.606
ppm
Boc

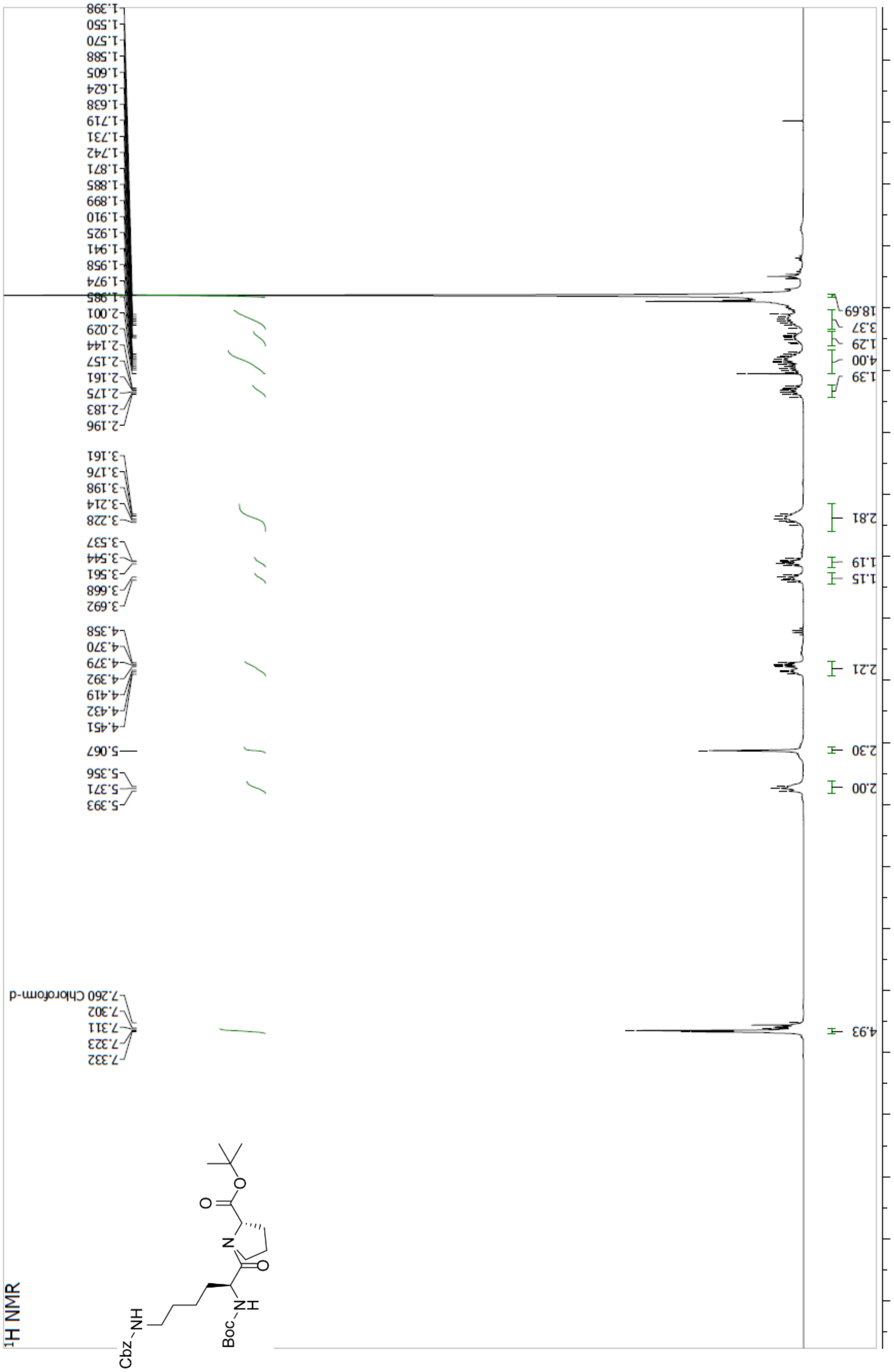

1H NMR
1.398
1.550
1.570
1.588
1.605
1.624
1.638
1.719
1.731
1.742
1.871
1.885
1.899
1.910
1.925
1.941
1.958
1.974
1.985
2.001
2.029
2.144
2.157
2.161
2.175
2.183
2.196
3.161
3.176
3.198
3.214
3.228
3.537
3.544
3.561
3.668
3.692
4.358
4.370
4.379
4.392
4.419
4.432
4.451
5.067
5.356
5.371
5.393
7.260 Chloroform-d
7.302
7.311
7.323
7.332
18.69
3.37
1.29
4.00
1.39
2.81
1.19
1.15
2.21
2.30
2.00
4.93
Cbz
NH
Boc
N
H
O
-0.5
0.0
0.5
1.0
1.5
2.0
2.5
3.0
3.5
4.0
4.5
5.0
5.5
6.0
6.5
7.0
7.5
8.0
8.5
9.0
9.5

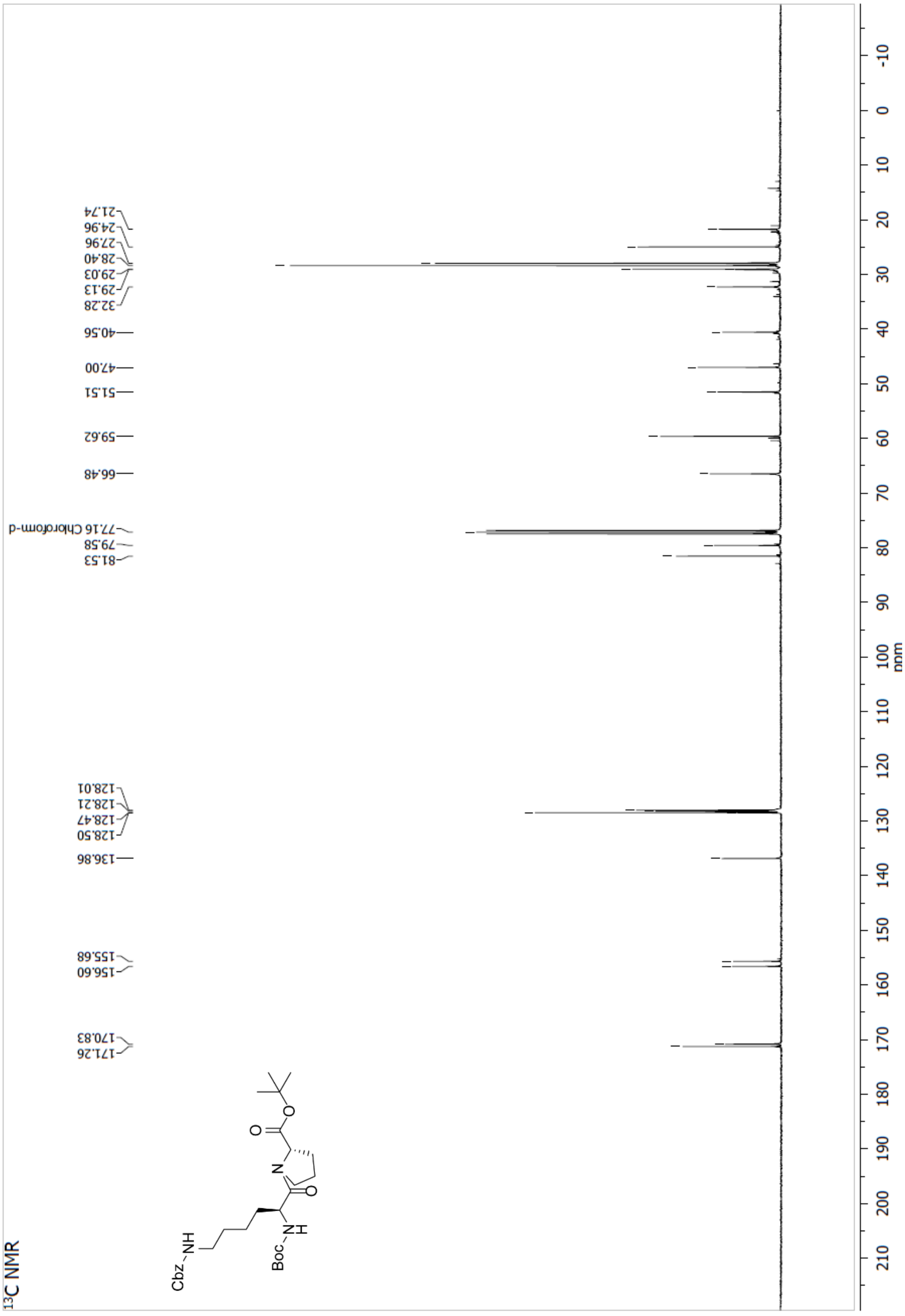

13C NMR
Cbz
NH
Boc
N
H
N
O
O
O
171.26
170.83
156.60
155.68
136.86
128.50
128.47
128.21
128.01
81.53
79.58
77.16 Chloroform-d
66.48
59.62
51.51
47.00
40.56
32.28
29.13
29.03
28.40
27.96
24.96
21.74
ppm

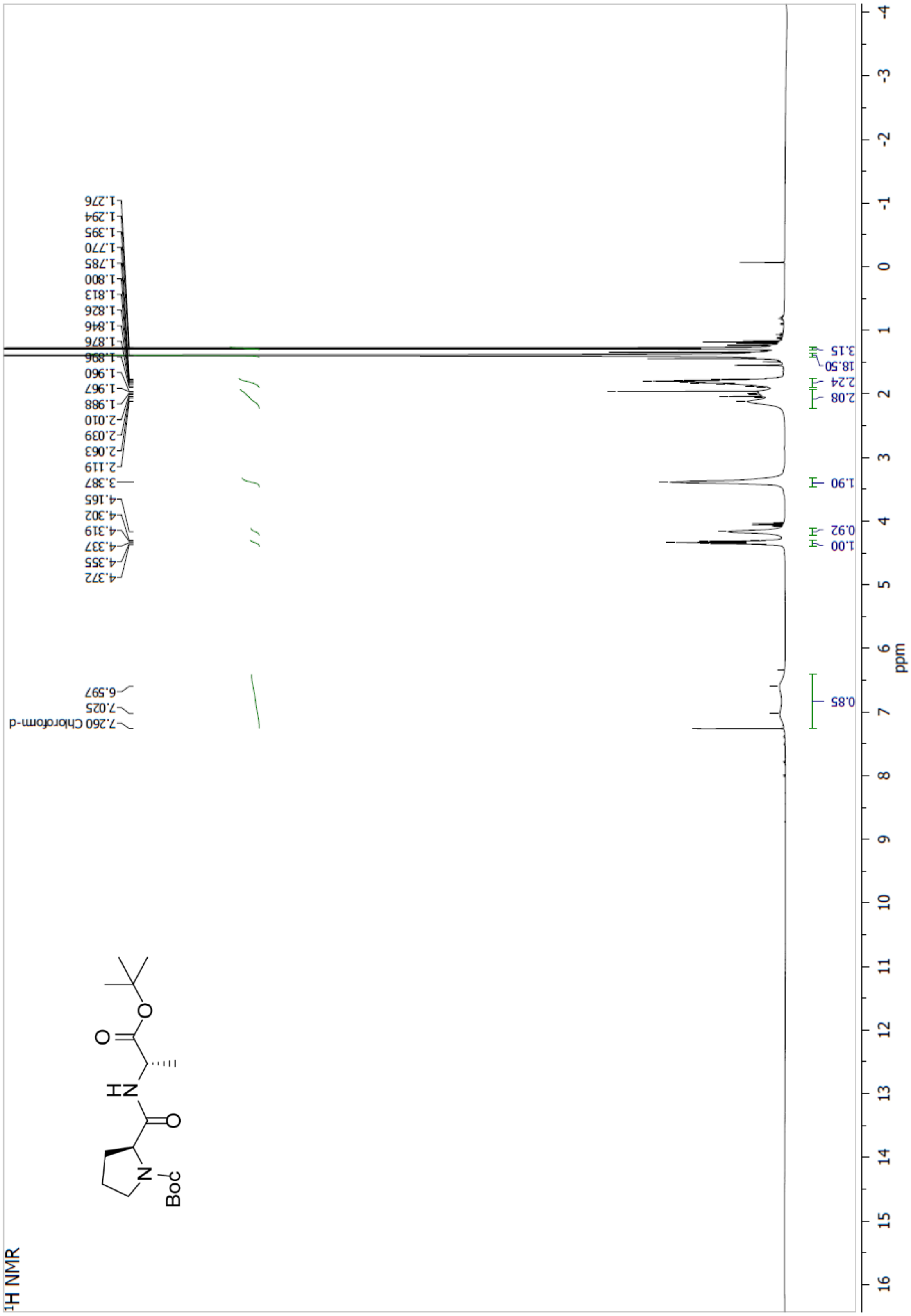

1H NMR
Boc
N
H
O
O
O
7.260 Chloroform-d
7.025
6.597
4.372
4.355
4.337
4.319
4.302
4.165
3.387
2.119
2.063
2.039
2.010
1.988
1.967
1.960
1.896
1.876
1.846
1.826
1.813
1.800
1.785
1.770
1.395
1.294
1.276
0.85
1.00
0.92
1.90
2.08
2.24
18.50
3.15
ppm

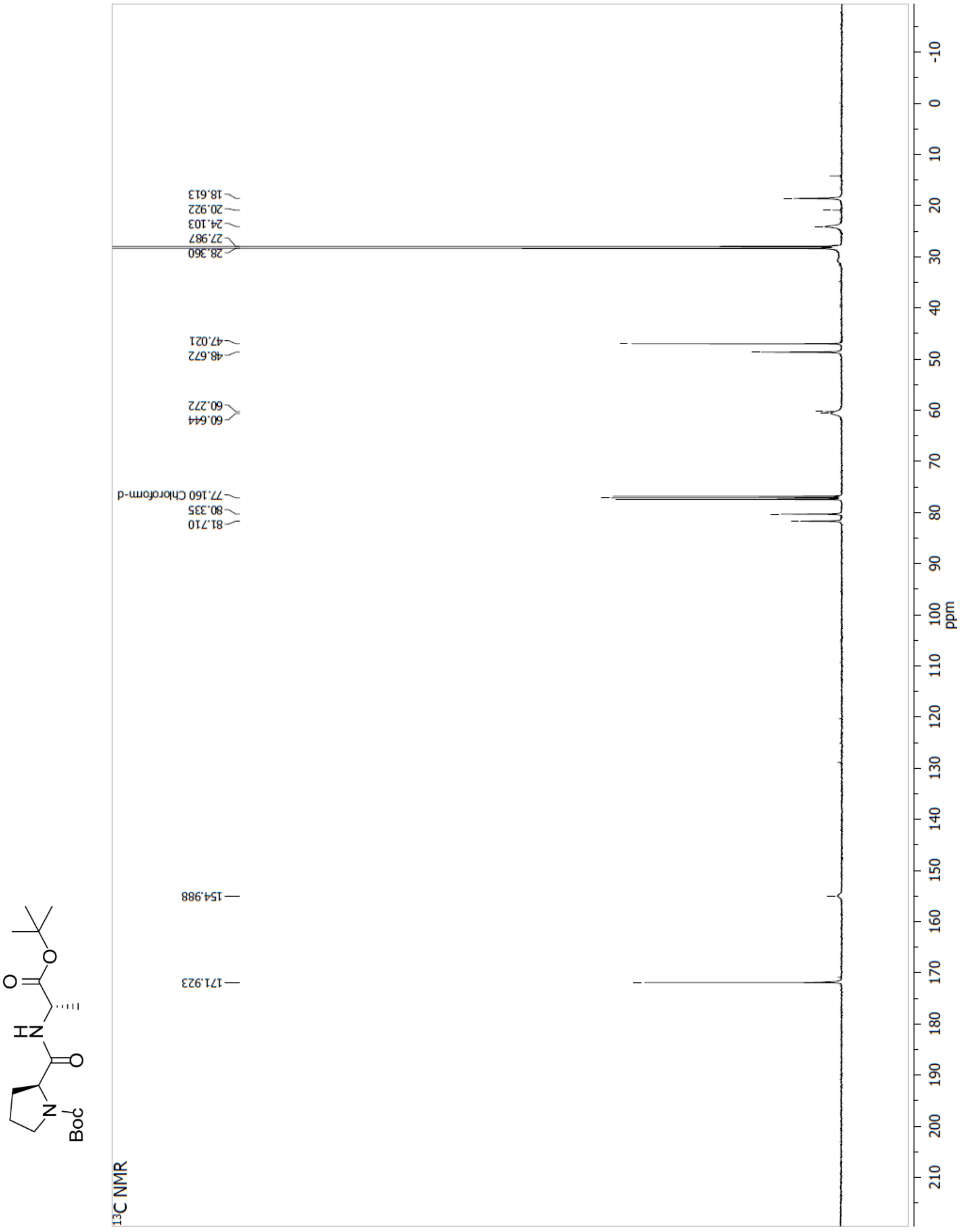
13C NMR
18.613
20.922
24.103
27.987
28.360
47.021
48.672
60.272
60.644
77.160 Chloroform-d
80.335
81.710
154.988
171.923
ppm
Boc
N
H
O

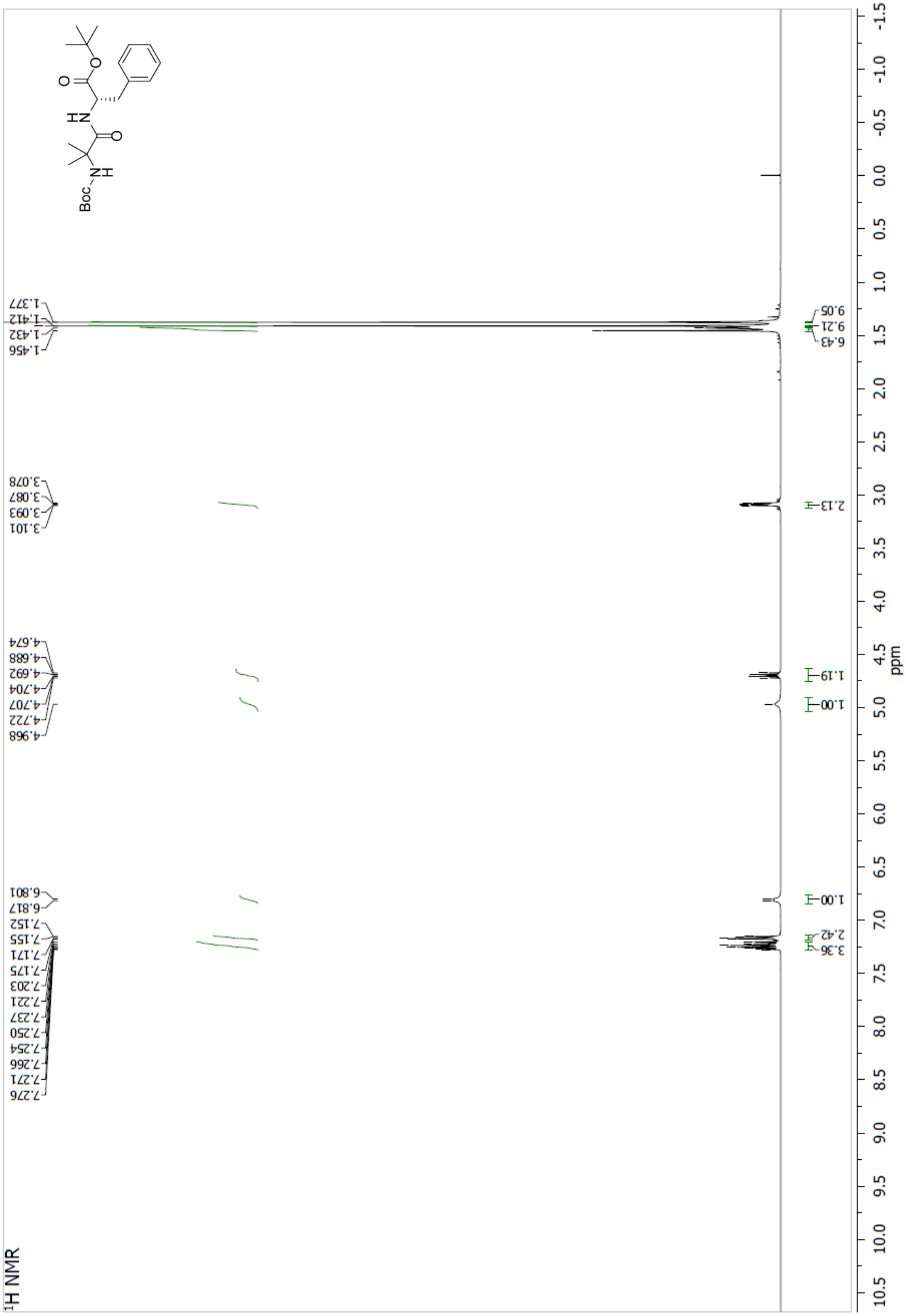
1H NMR
Boc
N
H
H
N
O
O
O
1.377
1.412
1.432
1.456
3.078
3.087
3.093
3.101
4.674
4.688
4.692
4.704
4.707
4.722
4.968
6.801
6.817
7.152
7.155
7.171
7.175
7.203
7.221
7.237
7.250
7.254
7.266
7.271
7.276
9.05
9.21
6.43
2.13
1.19
1.00
1.00
2.42
3.36
ppm

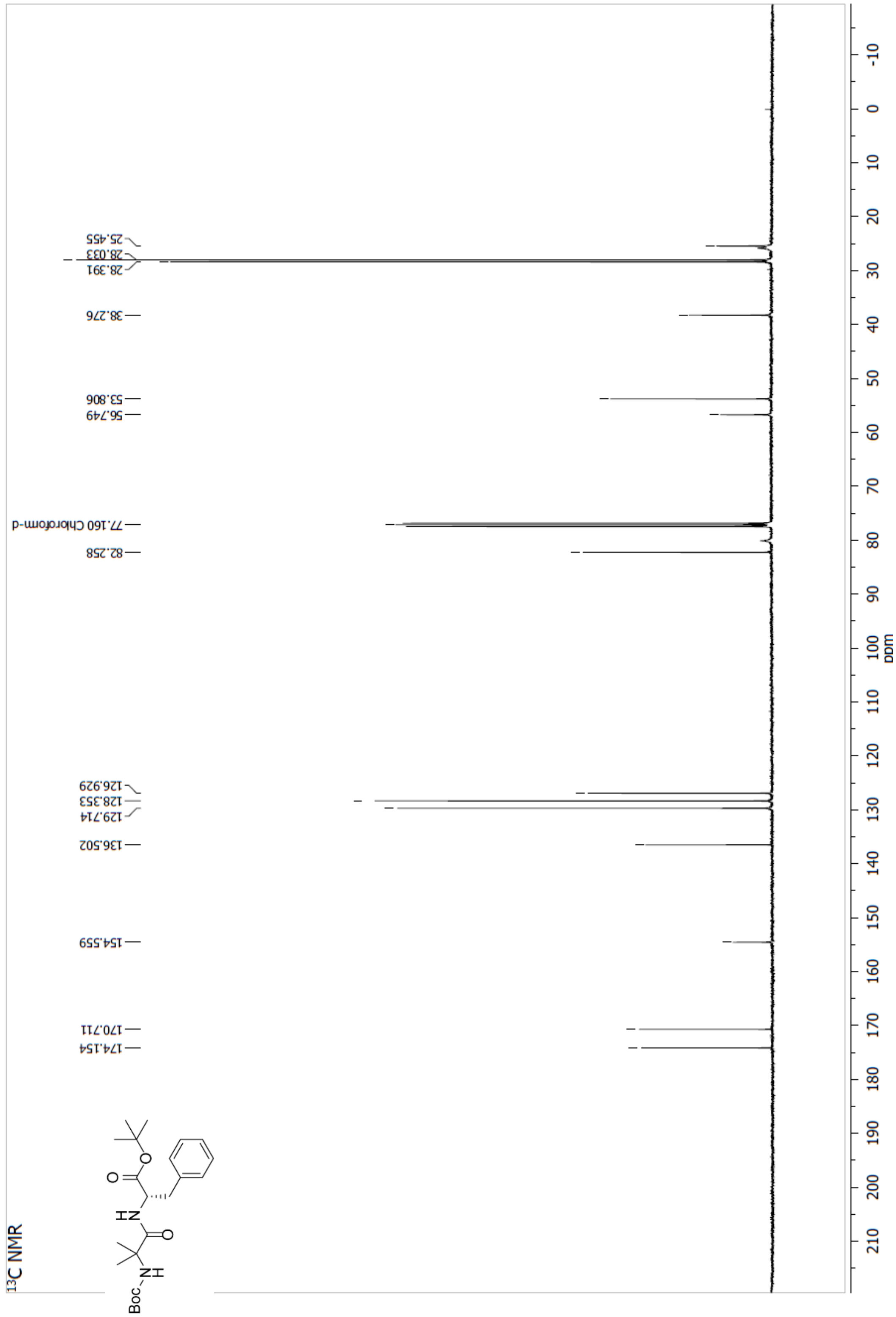

13C NMR
Boc
NH
HN
O
25.455
28.033
28.391
38.276
53.806
56.749
77.160 Chloroform-d
82.258
126.929
128.353
129.714
136.502
154.559
170.711
174.154
ppm

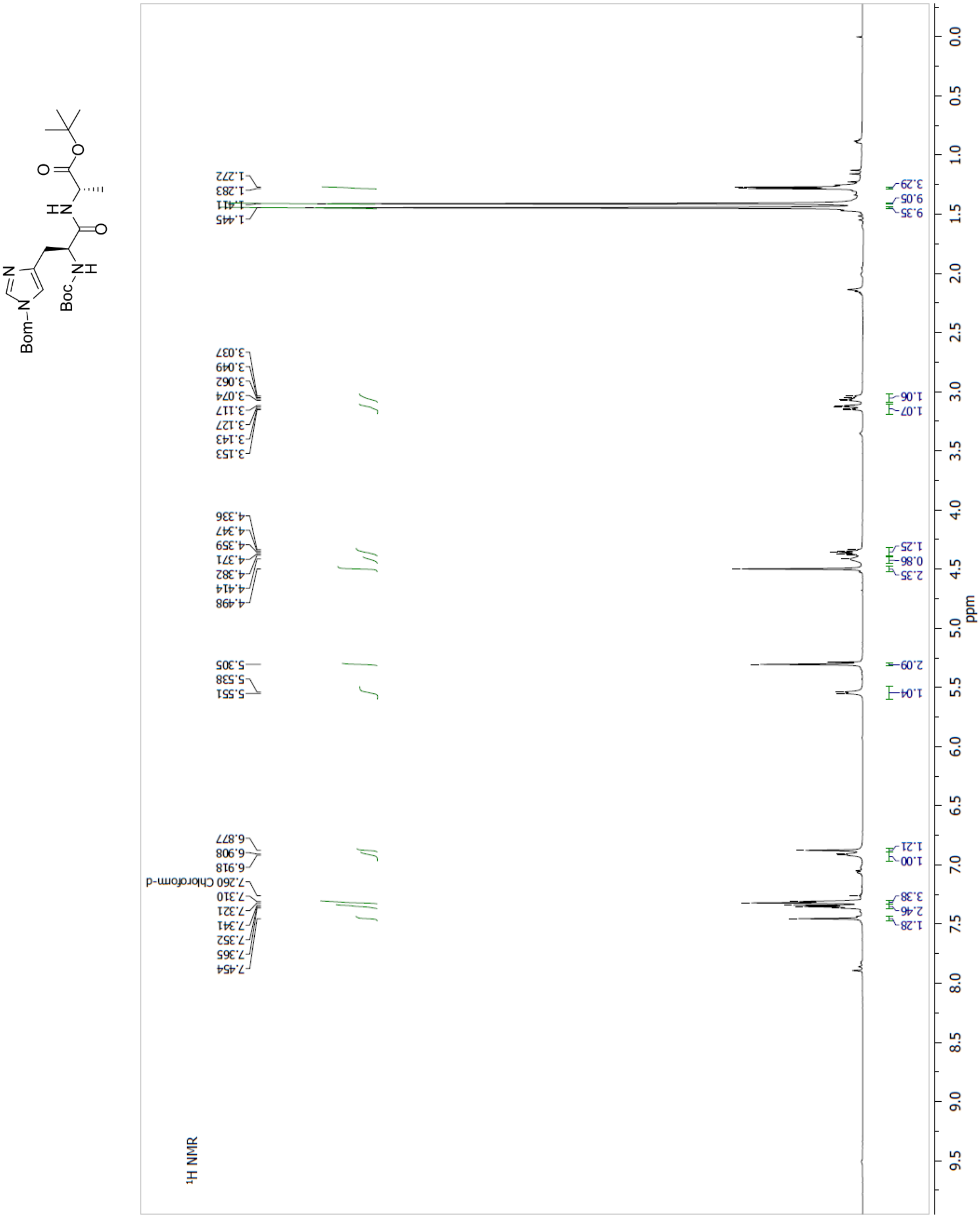
1H NMR
ppm
Chloroform-d
Bom
Boc

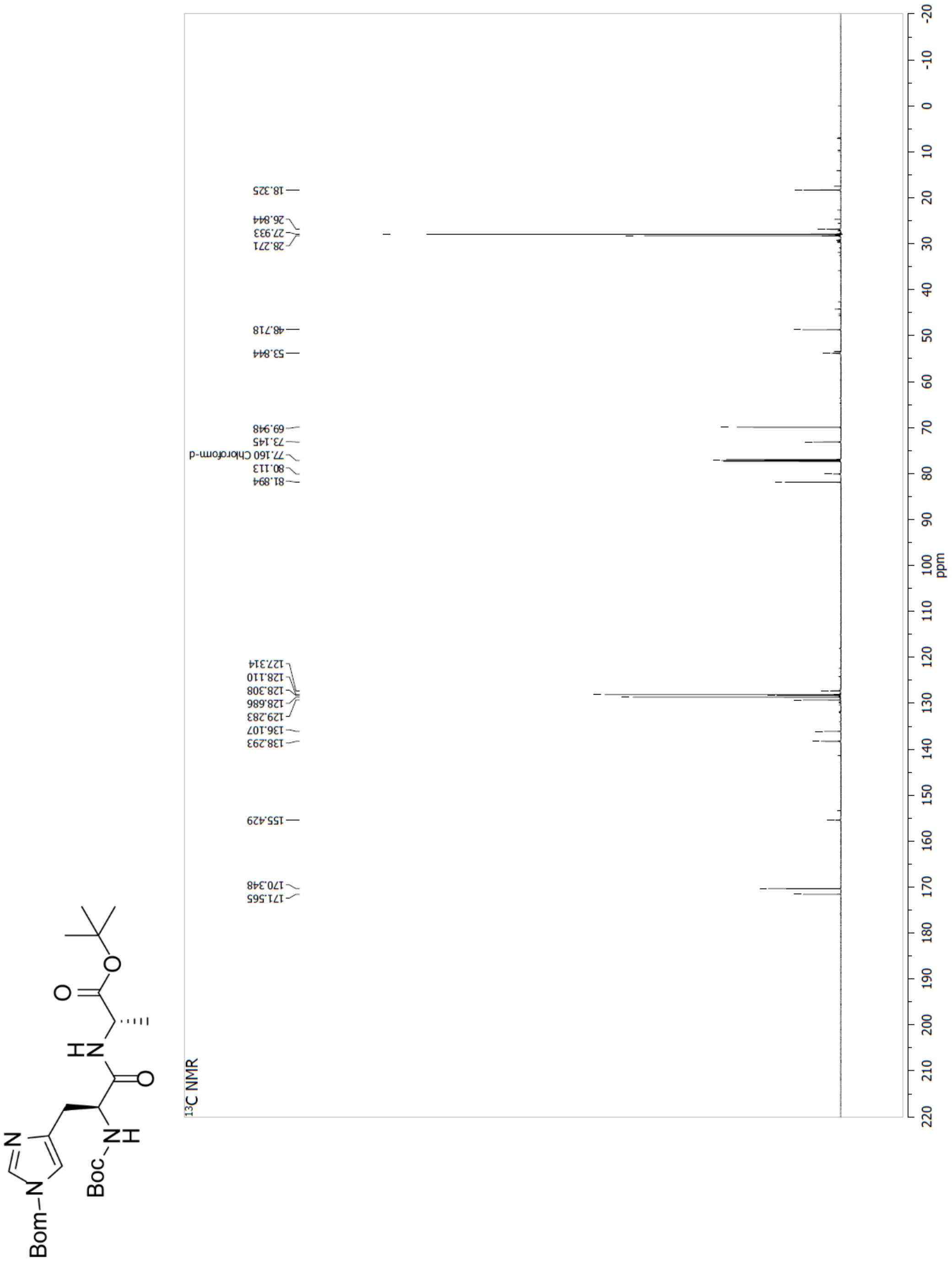

13C NMR
18.325
26.844
27.933
28.271
48.718
53.844
69.948
73.145
77.160 Chlorofom-d
80.113
81.894
127.314
128.110
128.308
128.686
129.283
136.107
138.293
155.429
170.348
171.565
ppm
Bom
Boc

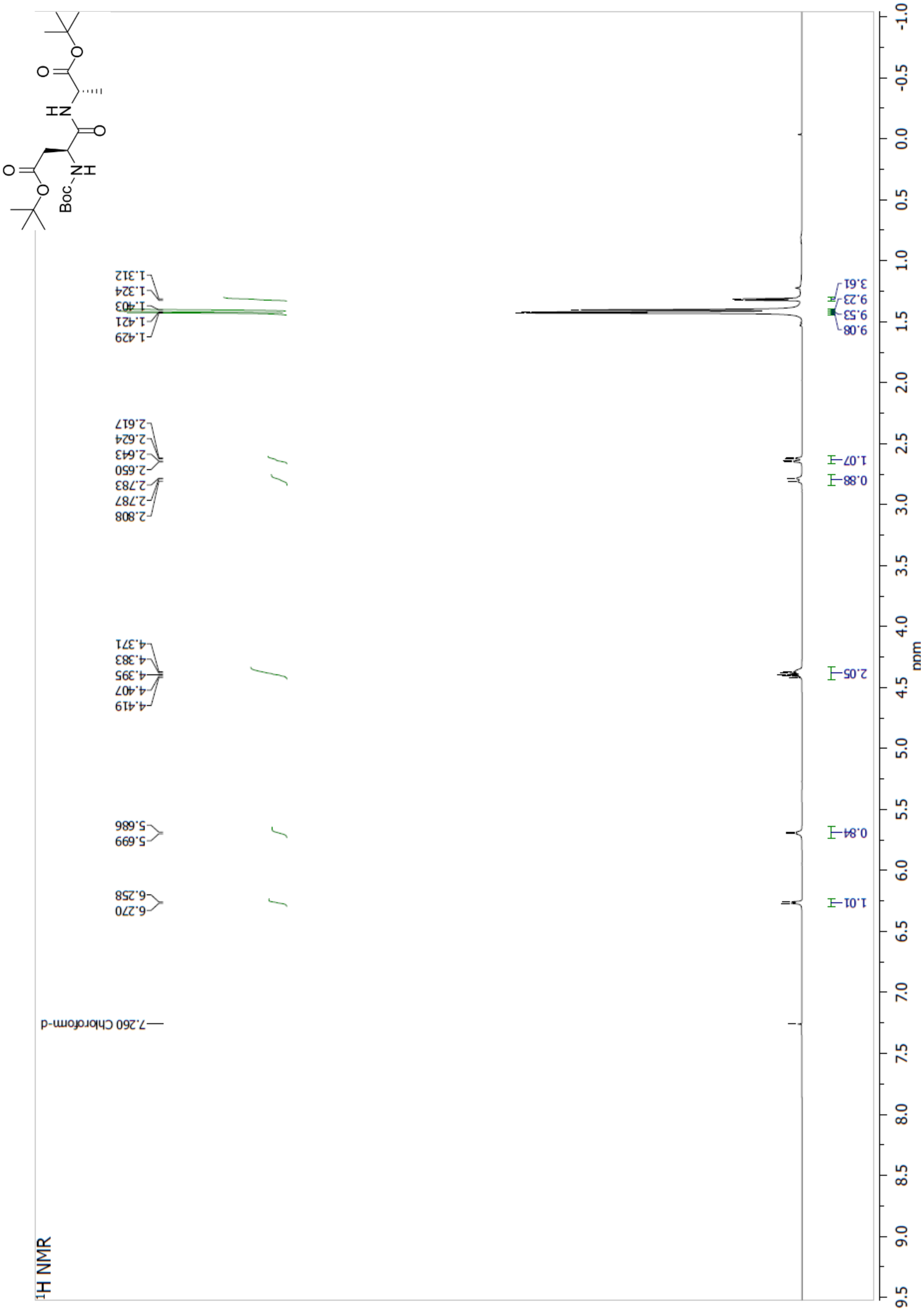
1H NMR
Boc
N
H
O
7.260 Chloroform-d
6.270
6.258
5.699
5.686
4.419
4.407
4.395
4.383
4.371
2.808
2.787
2.783
2.650
2.643
2.624
2.617
1.429
1.421
1.403
1.324
1.312
1.01
0.84
2.05
0.88
1.07
9.08
9.53
9.23
3.61
ppm

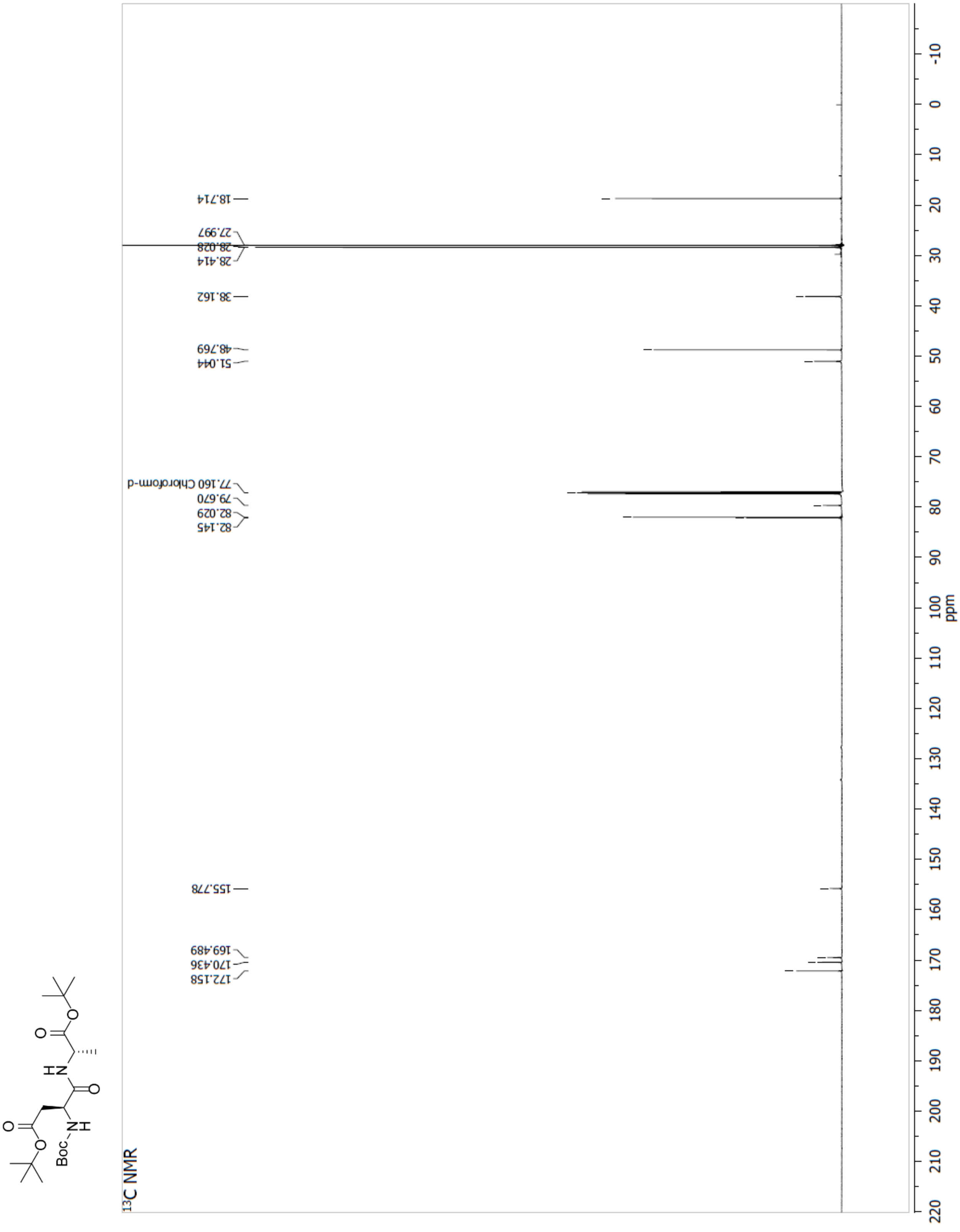

13C NMR
Boc
N
H
HN
O
18.714
27.997
28.028
28.414
38.162
48.769
51.044
77.160 Chloroform-d
79.670
82.029
82.145
155.778
169.489
170.436
172.158
ppm

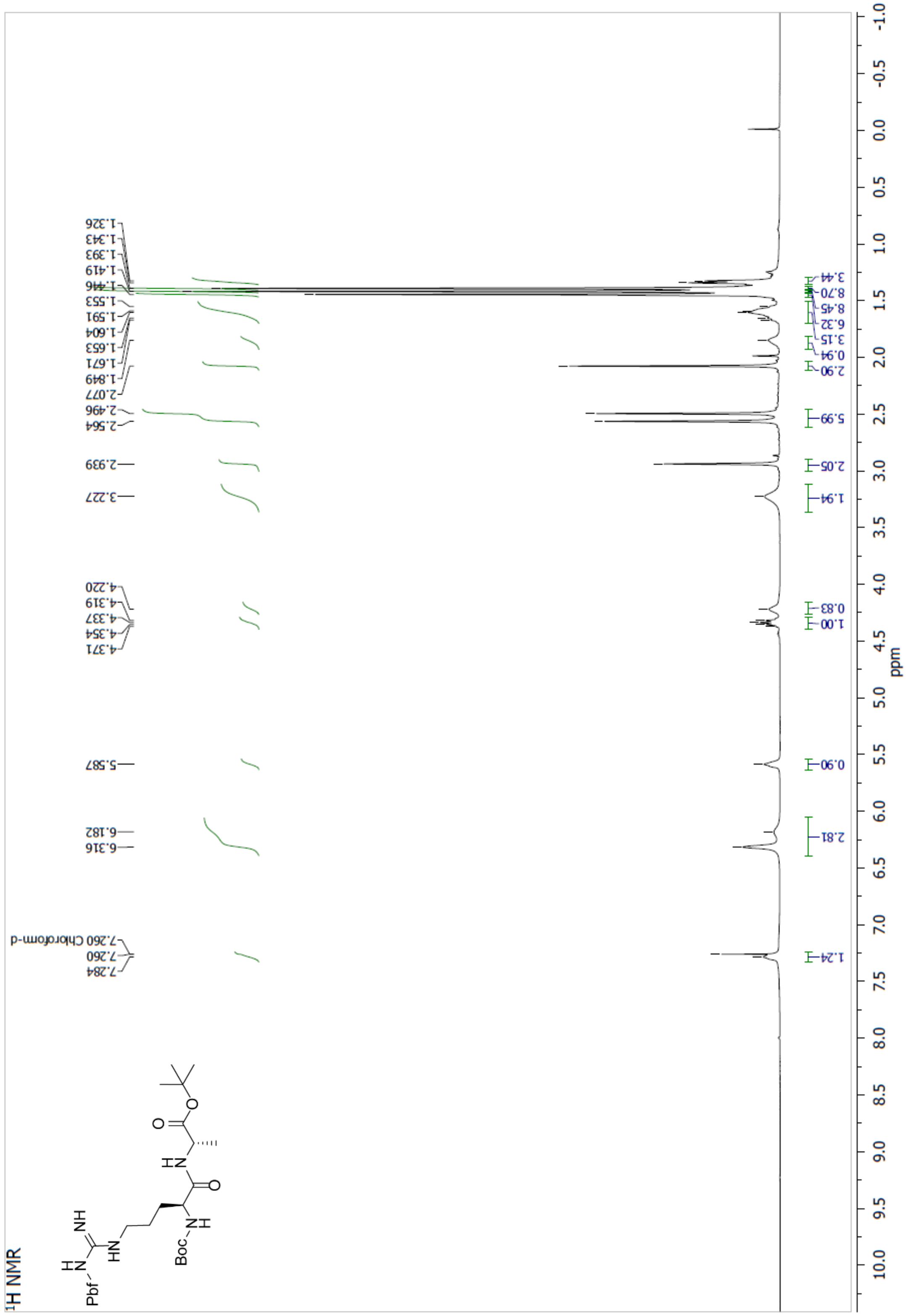
1H NMR
ppm
7.260 Chloroform-d

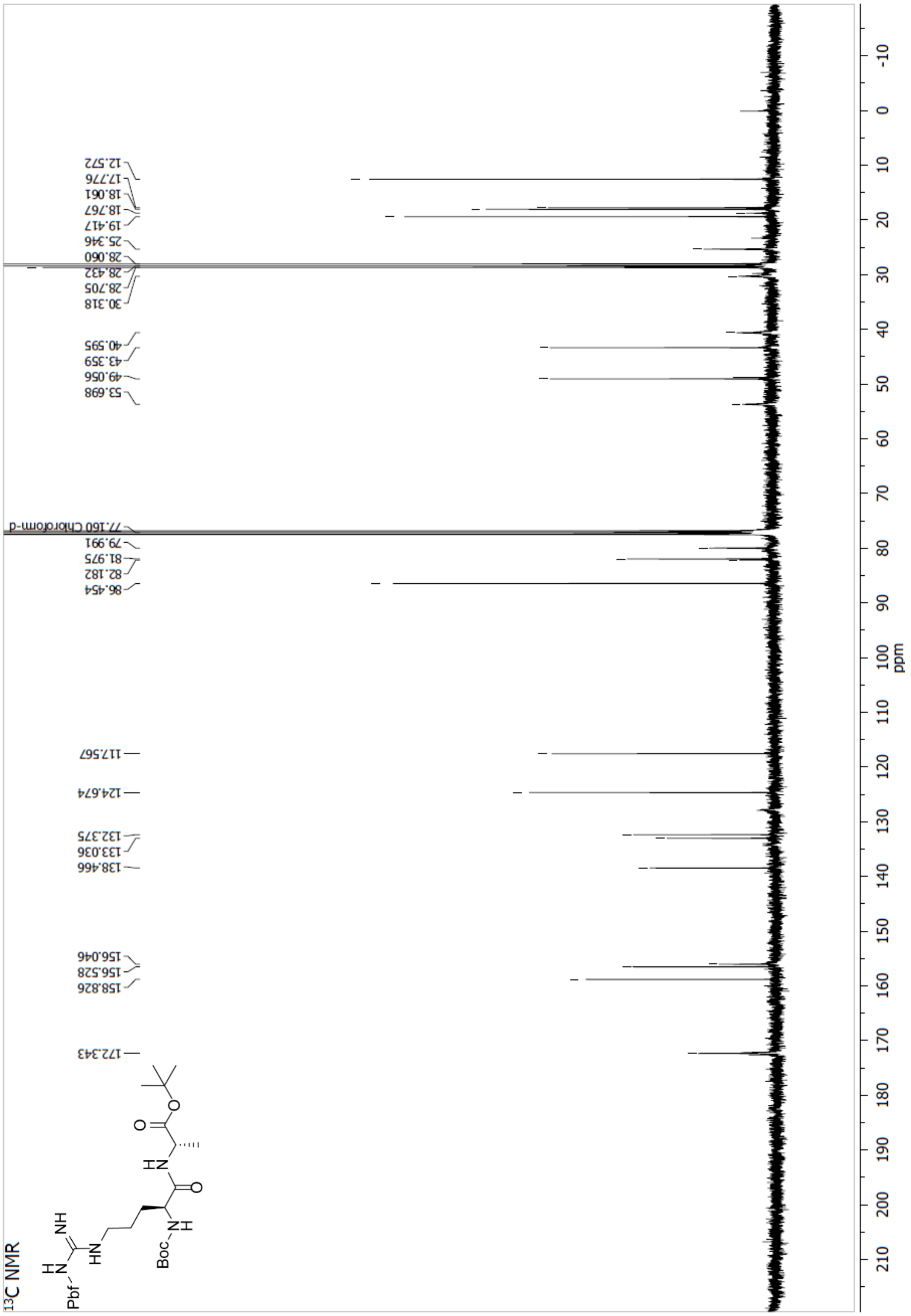
13C NMR
172.343
158.826
156.528
156.046
138.466
133.036
132.375
124.674
117.567
86.454
82.182
81.975
79.991
77.160 Chloroform-d
53.698
49.056
43.359
40.595
30.318
28.705
28.432
28.060
25.346
19.417
18.767
18.061
17.776
12.572
ppm
Pbf
Boc

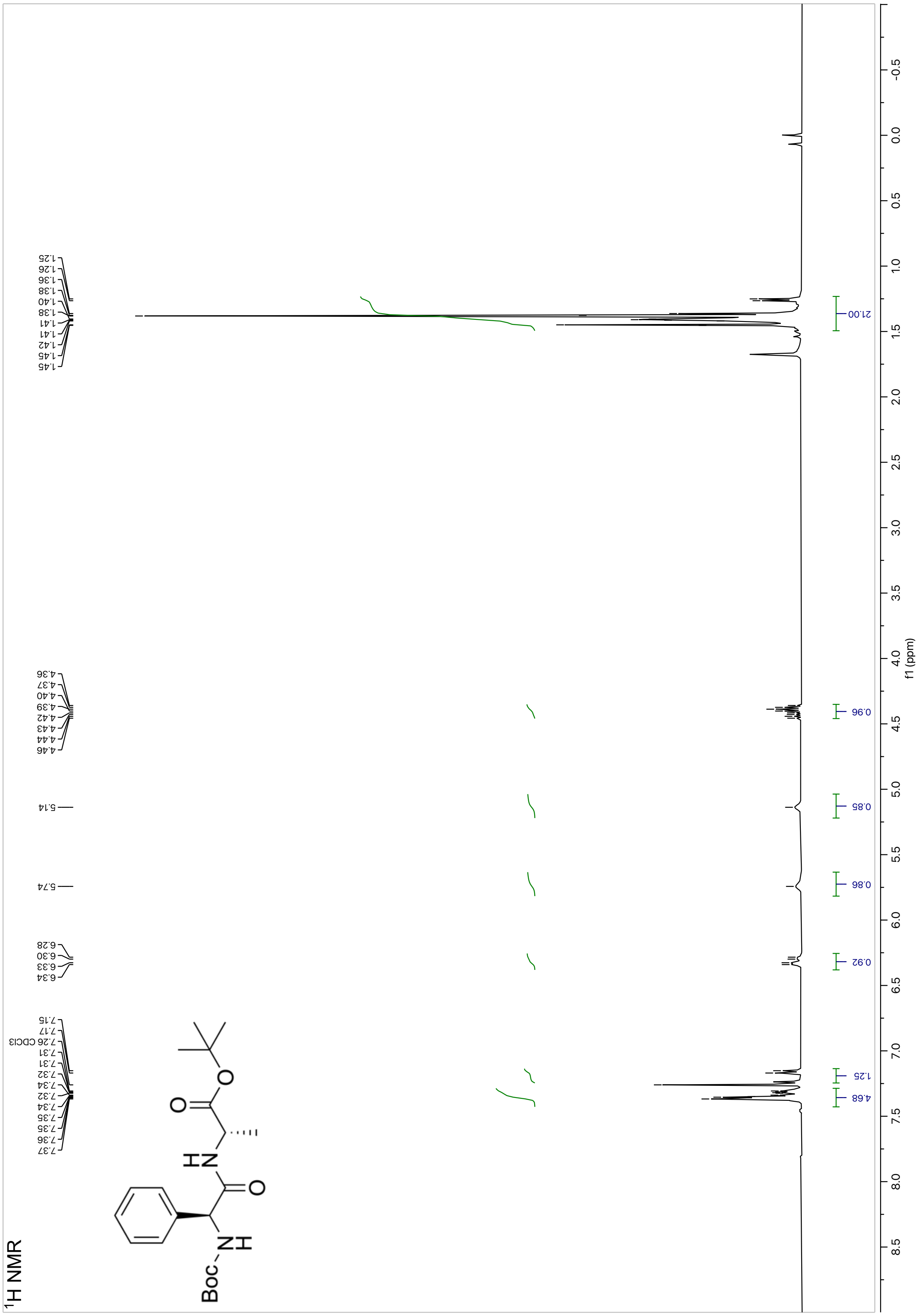

1H NMR
f1 (ppm)

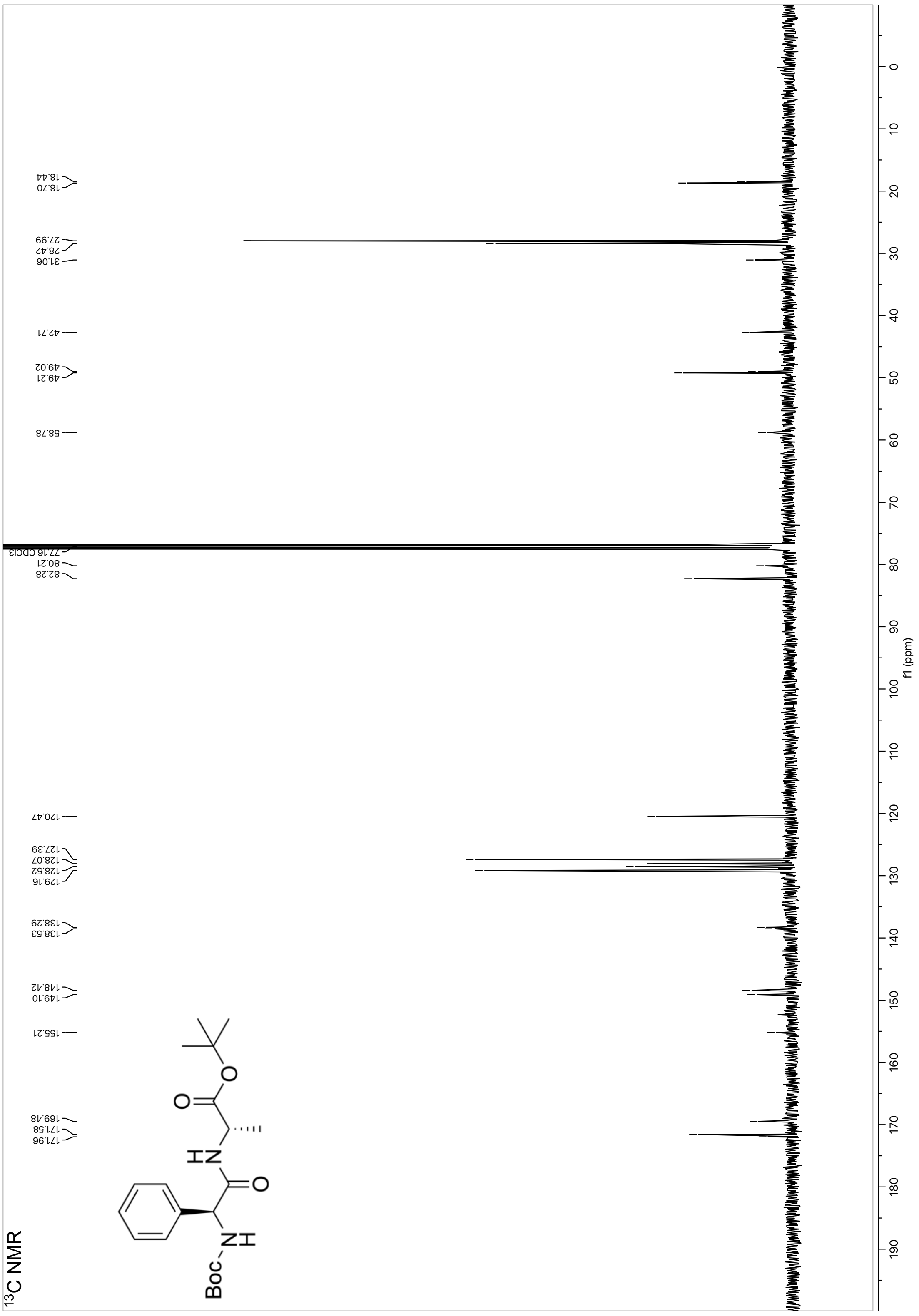

13C NMR
Boc
NH
O
NH
O
O
18.44
18.70
27.99
28.42
31.06
42.71
49.02
49.21
58.78
77.16 CDCl3
80.21
82.28
120.47
127.39
128.07
128.52
129.16
138.29
138.53
148.42
149.10
155.21
169.48
171.58
171.96
f1 (ppm)
